# Integrative analysis of transcriptomic data reveals a predictive gene signature for chemoradiotherapy response in rectal cancer

**DOI:** 10.1016/j.isci.2025.114455

**Published:** 2025-12-17

**Authors:** Claudia Corrò, Joao Victor Machado Carvalho, Melivoia Rapti, Paolo Angelino, Matthieu Tihy, Arnaud Bakaric, Giacomo Puppa, Pratyaksha Wirapati, André Durham, Frederic Ris, Stephanie Tissot, Jonathan Thevenet, Inti Zlobec, Valérie Dutoit, Mikael Pittet, Petros Tsantoulis, Thibaud Koessler

**Affiliations:** 1 Translational Research Center in Onco-Hematology (CRTOH), Department of Medicine, Faculty of Medicine, University of Geneva, 1205 Geneva, Switzerland; 2 Department of Pathology and Immunology (PATIM), Department of Medicine, Faculty of Medicine, University of Geneva, 1205 Geneva, Switzerland; 3 Swiss Cancer Center Léman, Lausanne, Switzerland; 4 Department of Oncology, Geneva University Hospital, 1205 Geneva, Switzerland; 5 SIB Swiss Institute of Bioinformatics, 1215 Lausanne, Switzerland; 6 Department of Pathology of Bichat and Beaujon AP-HP Hospitals, Université Paris Cité, Paris, France; 7 Department of Radio-oncology, Geneva University Hospital, 1205 Geneva, Switzerland; 8 Department of Visceral Surgery, Geneva University Hospital, 1205 Geneva, Switzerland; 9 Department of Oncology, Center of Experimental Therapeutics, Lausanne University Hospital (CHUV) and University of Lausanne (UNIL), Lausanne, Switzerland; 10 Institute of Tissue Medicine and Pathology, University of Bern, Bern, Switzerland

**Keywords:** Oncology, Molecular biology, Transcriptomics

## Abstract

Locally advanced rectal cancer (LARC) is treated with neoadjuvant chemoradiotherapy (nCRT), but only a minority of patients achieve a pathological complete response (pCR). Predictive biomarkers of response could help guide treatment decisions, yet none have reached clinical practice. In this exploratory study, we integrated six publicly available transcriptomic datasets and applied machine learning to derive a 186-gene signature predictive of nCRT response. The signature showed good performance in cross-validation (AUC 0.80) and was associated with consensus molecular (CMS4) and immune (iCMS3) subtypes enriched in responders. Gene set enrichment analyses highlighted pathways involved in tumor growth, immune regulation, and resistance. Spatial transcriptomic profiling of pre-treatment biopsies further identified compartment-specific markers, with tumor-associated genes showing greater predictive value. These results provide biological insights into response mechanisms and generate hypotheses for future validation. Larger prospective studies are required to assess the clinical utility of this approach.

## Highlights

• A 186-gene signature predicts response to nCRT in rectal cancer
• Signature shows consistent performance across six GEO datasets
• Associations observed with CMS4 and iCMS3 subtypes enriched in responders
• Spatial transcriptomics identifies compartment-specific candidate genes

## Graphical abstract

**Figure:**
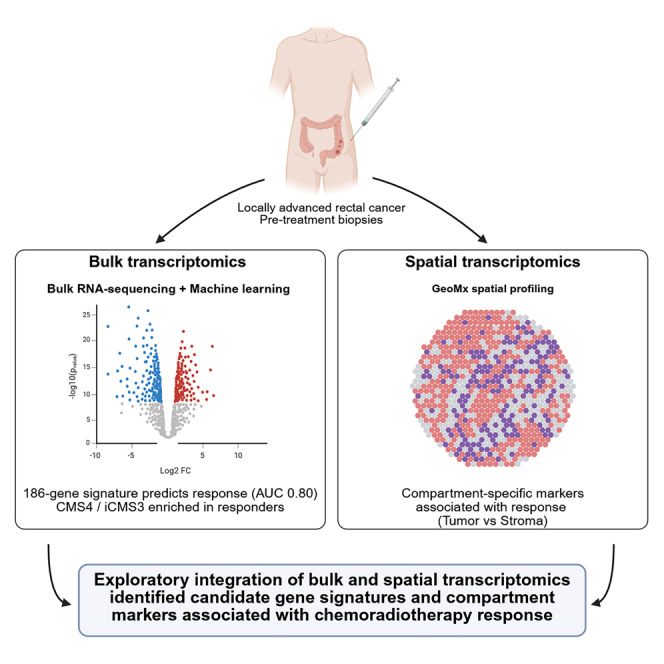

## Introduction

Rectal cancer (RC) accounts for approximately one-third of all colorectal cancer (CRC) cases, with an estimated 116,000 new cases and 47,000 deaths annually in the European Union.^1^ Locally advanced rectal cancer (LARC), classified as stage II or III according to the TNM system, is the most common stage at diagnosis in Europe.^2^ The primary treatment goal for LARC is curative, often involving neoadjuvant chemoradiotherapy (nCRT) or short-course radiotherapy (SCRT), followed by surgical resection.^2^

Approximately 15% of patients undergoing nCRT followed by surgery achieve a pathological complete response (pCR), defined as the absence of viable tumor cells upon pathological examination. Achieving pCR is associated with improved long-term prognosis and opens the possibility of non-surgical management, also known as the “watch and wait” strategy.^3^ In an effort to increase the likelihood of pCR, treatment intensification is frequently employed using the “Total Neoadjuvant Treatment” (TNT) approach, which combines nCRT with additional chemotherapy prior to surgery. TNT can increase the pCR rate to 30%, but this comes at the cost of higher treatment-related toxicity.^4^

Accurately predicting which patients will achieve pCR is crucial, as it can help tailor treatment plans by avoiding unnecessary treatment intensification for some patients and sparing others from undergoing nCRT. Numerous efforts and approaches have been used to identify predictive biomarkers for nCRT response. For example, Chatila et al. identified that the overexpression of IGF2 and L1CAM, through RNA sequencing, was associated with poor response to nCRT.^5^ El-Sissy and colleagues used the Immunoscore method and reported a strong correlation between higher densities of intratumoral CD3^+^ and CD8^+^ T cells and favorable treatment response, emphasizing the importance of spatial immune contexture.^6^ Lately, Zhang et al., using the GeoMx Digital Spatial Profiling (DSP) platform, found that high densities of HLA-DR/MHC-II+ cells within the tumor and CD20^+^ B cells in the stroma were associated with better responses to nCRT.^7^ Despite these promising results, none of these biomarkers have been replicated due to limitations such as small discovery cohort sizes, variability in technology platforms, and heterogeneity in neoadjuvant treatment protocols ultimately none of these biomarkers are used in clinical practice.

In this study, we aimed to address these challenges by conducting a comprehensive differential gene expression analysis across six Gene Expression Omnibus (GEO) datasets to identify a gene signature predictive of nCRT response in patients with LARC. A machine learning approach was employed to develop a robust cross-validated signature. Additionally, spatial transcriptomic analysis was performed on an in-house cohort, leveraging GeoMx Digital Spatial Profiling (DSP) to explore gene expression in tumor and stromal compartments. This spatial analysis provided deeper insights into the tissue-specific biological mechanisms underlying treatment response, complementing the findings from bulk transcriptomic data and offering potential compartment-specific biomarkers that should be further investigated. Overall, our goal was to develop a biologically informed and technically feasible approach that can be further tested in larger prospective studies.

## Results

### Identification of biomarkers of response using publicly available transcriptomic data

The primary objective of this study was to identify genes associated with the response to neoadjuvant chemoradiotherapy (nCRT) in pre-treatment biopsies of patients with locally advanced rectal cancer (LARC). To accomplish this, we analyzed six publicly available transcriptomic databases. We compiled a large and diverse patient cohort, ensuring that the identified potential biomarkers of response would not be overfit to any individual dataset.

An extensive search was conducted in the Gene Expression Omnibus (GEO) repository, resulting in the identification of six relevant gene expression series (GSE) datasets for analysis (Table S2). A differential expression analysis (DEA) was performed across these six datasets, and the results were further cross-referenced with data from four microarray datasets. These four microarray datasets, a subset of the original six, were selected based on their more homogeneous clinical characteristics, allowing for more refined analysis.

**Figure 1:**
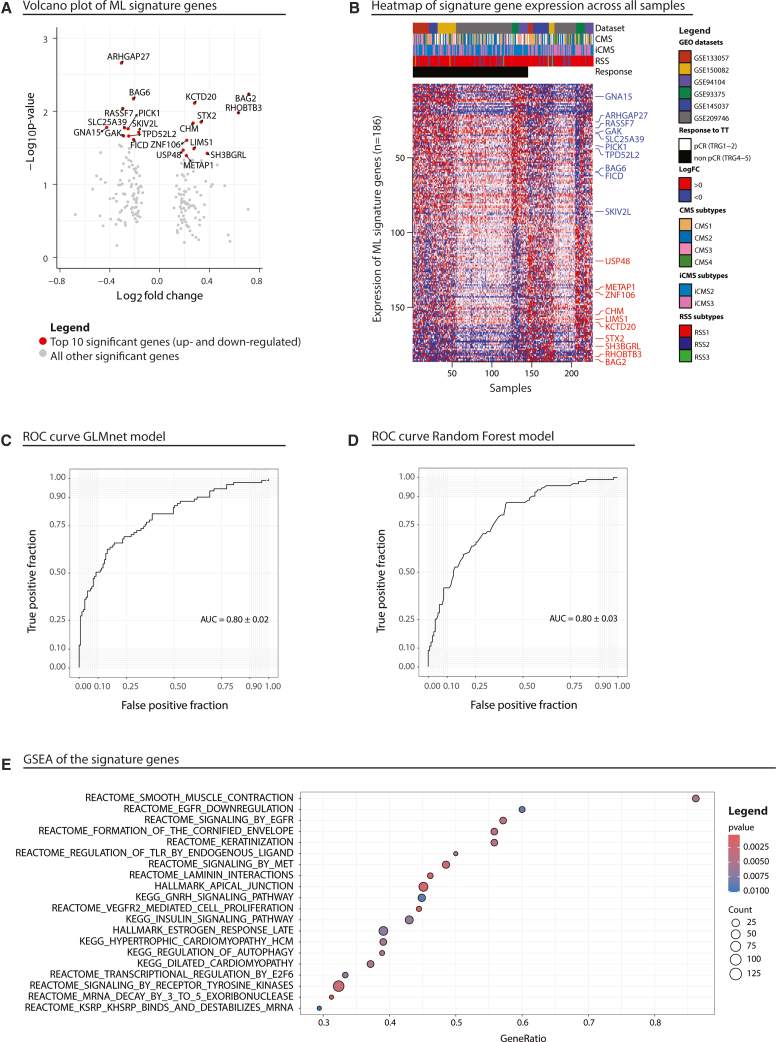
Transcriptomic analysis and development of a predictive gene signature (A) Volcano plot of differentially expressed genes between responders and non-responders across GEO datasets. (B) Heatmap shows expression patterns of significantly associated genes across datasets. (C) Performance of the 186-gene signature using GLMnet classifier. (D) Performance of the 186-gene signature using a Random Forest classifier. (E) Gene set enrichment analysis (GSEA) of pathways associated with treatment response.

The combined results from the differential expression analysis were subsequently utilized to train a machine learning model, which identified a set of 186 genes predictive of treatment - without batch correction (Figure S1). To validate this gene set, cross-validation was performed by randomly dividing the samples from the original six datasets into training and test sets. This procedure was repeated across the six datasets (Figure 1A). The model was built using scaled data repeated three times to evaluate the robustness and generalizability of the model.

A heatmap of the genes that were significantly (*p*-value <0.05) associated with response showed a consistent signal across datasets (Figure 1B). CMS3 subtype was more prevalent among non-responders, while CMS4 subtype was more frequent among responders (Figure S2A). This relationship between treatment response and CMS subtypes was statistically significant, *X*^2^ (1, *N* = 47) = 8.1384, *p* = 0.0043. When analyzing the immune classification of molecular subtypes (iCMS), the iCMS2 subtype was more prevalent among non-responders, whereas the iCMS3 subtype was predominant among responders (Figure S2B). This association was statistically significant, *X*^2^ (1, *N* = 226) = 11.647, *p* = 0.0006. To complement these classifications, we applied the response subtype stratification (RSS) classifier developed by Kisakol et al.,^8^ which defines three transcriptomic subtypes (RSS1–3) predictive of CRT response in rectal cancer. The majority of our samples, including many CMS4-classified tumors, were assigned to the RSS1 subtype, which is associated with immune activation and favorable treatment response. This may help explain the enrichment of CMS4 tumors among responders in our cohort (Figure 1B).

The predictive performance of the signature derived from this gene set achieved an area under the curve (AUC) of 0.80 with both the GLMnet and Random Forest models (Figures 1C and 1D). These findings provide a robust foundation for further exploration into the potential utility of these genes as biomarkers for predicting therapeutic response in clinical practice.

To further evaluate the robustness of the signature, we examined whether its predictive performance was independent of available clinicopathological variables. We reviewed all six GEO datasets used in the study for clinical annotations, but found that detailed metadata such as TNM staging or lymphovascular invasion were either inconsistently reported or absent. However, three datasets (GSE150082, GSE133057, and GSE145037) contained harmonized information for age and sex. Using these, we performed ANOVA to test for associations between the signature scores and these clinical variables. The results showed no significant associations (all *p*-values >0.05), suggesting that the predictive value of the signature is independent of patient age or sex in these cohorts. A summary of the available clinical information for each dataset is presented in Table 1.

**Table 1:** Clinical information is available across the six GEO datasets included in the study

Dataset	Gender	Age	Overall survival (days)	Dead/Alive	Molecular subtype	Clinical n positive	Clinical t stage
GSE94104	–	–	–	–	–	–	–
GSE93375	–	–	–	–	–	–	–
GSE150082	+	+	–	–	–	–	–
GSE133057	+	+	+	+	–	–	–
GSE209746	–	–	–	–	+	–	–
GSE145037	+	+	–	–	–	+	+

Summary of patient characteristics with harmonized clinical variables, including age and sex, where available.

Lastly, we conducted gene set enrichment analysis (GSEA) on the identified gene signature to uncover key involved biological pathways (Figure 1E). The analysis highlighted significant involvement of VEGFR and EGFR signaling, both critical in tumor growth, angiogenesis, and immune modulation in rectal cancer. Additionally, pathways related to cell junctions were enriched, emphasizing their role in tumor cell adhesion and metastasis. The involvement of Toll-like receptor (TLR) signaling, essential for immune regulation and innate immunity, suggests an impact on the tumor immune microenvironment. Finally, we observed enrichment in tyrosine kinase (TK) and E2F6 pathways, both linked to colorectal cancer progression and favorable responses to immune checkpoint blockade.

### Evaluating performance on an external dataset

We evaluated our model’s predictive performance on the recently published Domingo et al.^9^ dataset. Our model’s predictive performance on this dataset, yielding a modest AUC of 0.62 (Figure S3). This is likely due, at least in part, to the fact that this dataset was generated using a microarray platform, preventing us from re-aligning probe sequences to the same common annotation (GENCODE28) used in the other datasets. Consequently, individual genes were often represented by multiple probes, with some probes correlating positively with the response variable and others negatively. Additionally, we opted not to apply batch effect corrections to avoid potential bias in this test set; however, this choice introduced additional noise and variability, further reducing predictive accuracy.

### Comparison with previously published signatures

**Figure 2:**
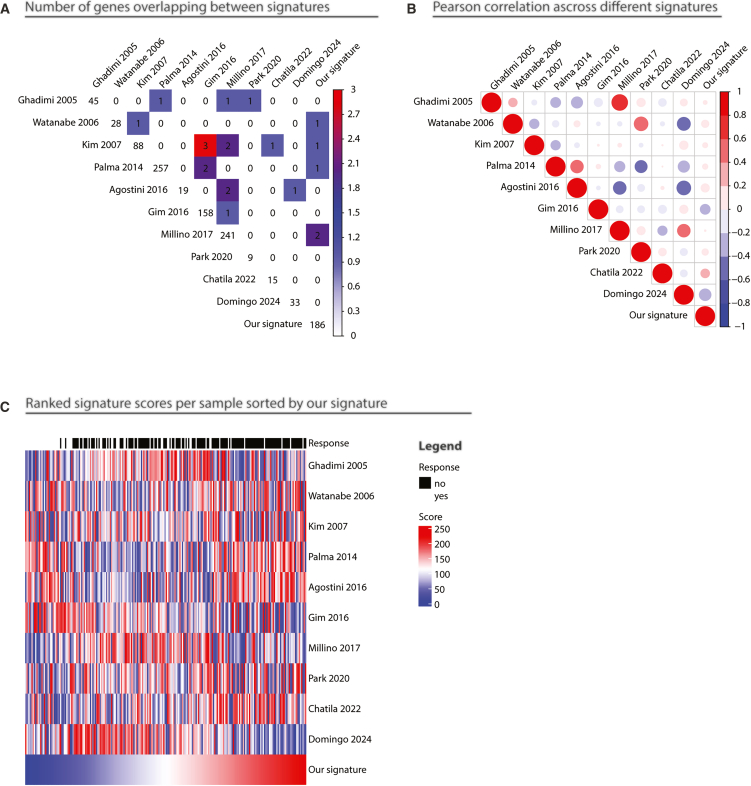
Comparison of the 186-gene signature with previously published predictive signatures (A) Overlap of genes between the 186-gene signature and published signatures. (B) Correlation matrix of signature scores across published signatures and the present study. (C) Comparison of patient ranking by predicted response across different signatures.

There was minimal overlap between our signature and previously published signatures (Figure 2A). Most of the signatures were weakly but positively correlated, possibly due to common underlying pathways (Figure 2B). Of note, our signature negatively correlated with Gim 2016 (rho −0.27) and Domingo 2024 (rho −0.37) but positively correlated with the other eight signatures (rho ranging between 0.01 and 0.21). Additionally, we compared sample rankings generated by each signature and found that none of the other signatures aligned closely with our rankings (Figure 2C). Overall, while the published signatures exhibit some overlap, they do not generate identical patient ranks, and the differences in clinical prediction are significant.

### Spatial transcriptomic profiling reveals compartment-specific biomarkers

**Figure 3:**
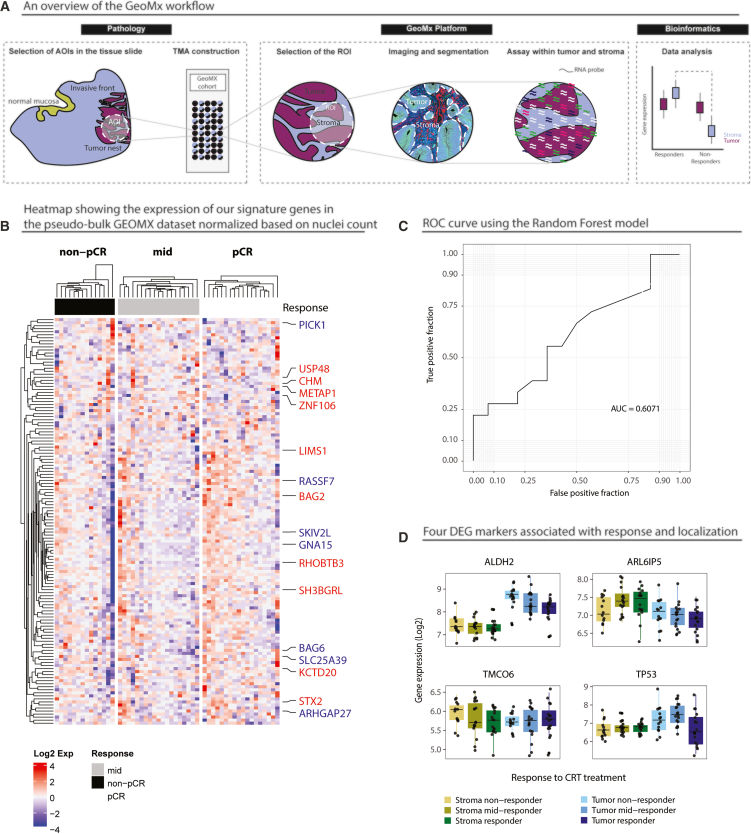
Spatial transcriptomic profiling of pre-treatment biopsies from patients with LARC (*n* = 53) (A) Representative workflow of GeoMx Digital Spatial Profiling (DSP) and selection of tumor (PanCK+) and stroma (PanCK−) compartments. (B) Pseudo-bulk analysis combining tumor and stromal compartments. (C) Predictive performance of the bulk-derived 186-gene signature applied to spatial data. (D) Compartment-specific analysis showing genes associated with response in tumor and stroma compartments. Data are represented as mean ± SEM.

We performed spatial transcriptomic analysis on 53 pre-treatment biopsies from patients with LARC treated at HUG, utilizing GeoMx technology (Figure 3A). As a quality control measure, we identified differentially expressed genes (DEGs) between the tumor and stroma compartments, uncovering 5,056 DEGs and 406 pathways that were consistently reproduced across five bootstrap runs. These genes and pathways were associated with well-established tumor- and stroma-specific characteristics, supporting the reliability of our analytical pipeline (Data S1).

We generated a synthetic pseudo-bulk expression profile by combining tumor and stromal compartments, weighted by nuclei counts, to assess our signature. The identified signature, however, showed limited performance in distinguishing between responder and non-responder patients, indicating that the pseudo-bulk GeoMx dataset may not fully capture the expected signature patterns (Figure 3B). The random forest model achieved an AUC of 0.6071 (Figure 3C). This limitation is likely due to challenges in applying a bulk RNA-seq-derived signature to the GeoMx platform, as several signature genes exhibited low or zero counts, and the precise tumor-to-stroma ratio remained unknown, with nuclei counts used as an approximation. In addition, the lack of overlap between genes identified in bulk and spatial transcriptomic analyses likely reflects the distinct biological resolution of each method, with spatial profiling capturing compartment-specific signals not discernible in bulk data.

A cause for this discrepancy could be that some genes behave differently in the epithelial and stromal compartments. Therefore, next, we focused on analyzing gene expression in the tumor and stroma compartments independently. Differential expression analysis between responders and non-responders identified eight genes significantly associated with treatment response in both compartments (Figure S4A). When assessing the two compartments separately, we identified 28 markers with significant interaction between their predictive role and their tissue localization (Figure S4B; Table S3). Thirteen of these genes were significantly associated with response in the tumor compartment, and seven were significant in the stroma compartment. The remaining eight genes exhibited opposing associations with response in the two compartments (opposite log fold change), but the association strength in each tissue was insufficient for them to be considered potential response markers. Four of these eight genes: *ALDH2*, *ARL6IP5*, *TMCO6,* and *TP53* have notable biological significance (Figure 3D).

The gene set enrichment analysis (GSEA) plots for both the tumor and stroma compartments reveal distinct biological pathways that are predictive of response to treatment in patients with rectal cancer (Figure S5). In the stroma compartment (Figure S5A), the enriched pathways include Fc-gamma receptor-dependent phagocytosis, complement cascade, and B cell receptor signaling, which underscore the role of humoral and phagocytic immune mechanisms. Similar to the tumor, stroma pathways involving Toll-like receptor cascades and interferon signaling were predictive of treatment outcomes. Additionally, cell surface interactions and phospholipid involvement in phagocytosis were prominent, further emphasizing the importance of immune-modulatory interactions in the stroma. Epigenetic regulation pathways, such as DNA methylation and histone post-translational modifications, were also enriched, reflecting the stroma’s active participation in the treatment response via transcriptional control.

In the tumor compartment (Figure S5B), key enriched pathways predictive of response include interferon signaling (both alpha and gamma responses), neutrophil degranulation, and Toll-like receptor cascades, indicating an active immune response. A point worth noting is that our tumor compartment also includes intra-tumoral immune cells, as the segmentation was performed solely based on PanCK staining. Additionally, pathways such as hypoxia, xenobiotic metabolism, and lysosome activity were also associated with treatment response, suggesting a role of metabolic and stress-response processes in the tumor microenvironment. Several pathways linked to chromatin modification (e.g., histone acetylation and DNA methylation) were also activated, highlighting the importance of epigenetic regulation in mediating response.

Our analysis also revealed a higher frequency of tumor-associated genes linked to treatment response compared to stroma-associated genes, indicating that the tumor compartment in our cohort holds greater predictive value for response. These results indicate that both immune-related and epigenetic mechanisms are central to predicting response to treatment in rectal cancer, with distinct but overlapping contributions from the tumor and stroma compartments.

A recent study by Zhang et al. examined 19 pre-treatment biopsies from patients with LARC receiving nCRT treatment and identified three DEGs (*KRT17*, *CD55*, and *PPBP*) associated with a favorable response in simulated bulk data. In their study, responders were classified as TRG0-1, and non-responders as TRG2-3, according to the AJCC scale. Notably, none of these genes were significantly different between responders and non-responders in our cohort (*p*-values are 0.21, 0.66, and 0.70, respectively). Moreover, Zhang et al. reported upregulated tumor compartment genes, including *IFI6*, *MMP7*, and *HLA-DR/MHC-II*-related genes (*CD74*, *HLA-DRA*, *HLA-DRB1*), and stroma-specific genes such as S100A9 and *MMP9* in major responders. In contrast, we observed no significant differential expression of these genes in our cohort.

The discrepancies between our findings and those of Zhang et al. could be attributed to the relatively small sample sizes in both studies, as well as differences in patient classification criteria for responders and non-responders. Furthermore, Zhang et al. utilized a smaller cancer transcriptome atlas of only 1,812 genes.

These findings underscore the importance of examining tumor and stroma compartments separately when searching for response biomarkers, as biologically relevant predictive markers may be masked in bulk-type analyses. Nevertheless, larger studies incorporating spatial transcriptomic data are crucial to validate these observations and determine whether spatial biomarkers can formally enhance the predictive power of signatures derived from bulk transcriptomic data.

## Discussion

While promising, our findings should be interpreted as exploratory due to the absence of functional validation.

In this study, we identified a gene signature associated with the response to neoadjuvant chemoradiotherapy (nCRT) in patients with locally advanced rectal cancer (LARC) by leveraging publicly available transcriptomic data. Our analysis across multiple transcriptomic datasets resulted in a gene signature predictive of treatment response.

The identified gene signature shows an association with consensus molecular subtypes (CMSs) (*p* = 0.0043) and immune classifications (iCMS) (*p* = 0.0006), reinforcing its biological relevance in colorectal cancer (CRC). This correlation highlights the interconnectedness of various molecular classification systems and their potential to provide complementary insights into tumor biology and treatment response. Additionally, the application of the response subtype stratification (RSS) classifier, as developed by Kisakol et al.,^8^ assigned the majority of samples, including many CMS4 tumors, to the RSS1 subtype, which correlates with immune activation and favorable CRT response. This further supports the observed enrichment of CMS4 tumors among responders in our cohort.

Notably, the CMS4 subtype, characterized by mesenchymal features and stromal infiltration, was more prevalent among responders.^10^ This aligns with previous findings suggesting that CMS4 tumors may be more responsive to certain therapies due to their unique microenvironment and cellular plasticity.^11^ Conversely, the CMS3 subtype, known for its metabolic dysregulation and KRAS mutations, was more frequent among non-responders.^10^ This observation is consistent with prior studies indicating that CMS3 tumors often exhibit resistance to standard therapies, possibly due to their altered metabolic profiles.^12^

The immune classification molecular subtypes (iCMS) results further supported these findings, with iCMS3 (immune-inflamed) more common in responders and iCMS2 (immune-excluded) more frequent in non-responders.^5^^,^^13^ This pattern underscores the critical role of the tumor microenvironment in shaping treatment response. The immune-inflamed phenotype of iCMS3 tumors may create a more favorable environment for therapeutic interventions, particularly immunotherapies.^14^ In contrast, the immune-excluded nature of iCMS2 tumors could contribute to treatment resistance by limiting immune cell infiltration and activation.^15^The correlation between the gene signature, CMS, and iCMS classifications suggests a complex interplay between tumor intrinsic factors and the surrounding microenvironment in determining treatment outcomes. These observations suggest that integrating molecular subtyping with the identified gene signature could provide a more comprehensive approach to predicting therapeutic outcomes in CRC. Future studies should focus on elucidating the mechanistic links between these classification systems and treatment response, potentially leading to more personalized and effective therapeutic strategies.^15^

The biological relevance of the identified gene set was further supported by the gene set enrichment analysis (GSEA), which pointed to critical pathways such as VEGFR and EGFR signaling, as well as Toll-like receptor (TLR) signaling. These pathways are well known for their roles in tumor growth, angiogenesis, and immune modulation, particularly in colorectal cancer. For instance, VEGFR-1 has been shown to regulate EGF-R expression to promote proliferation in colon cancer cells.^16^ Similarly, the TLR9 agonist IMO has demonstrated the ability to synergize with cetuximab in KRAS mutant colorectal cancer models, highlighting the interconnectedness of these pathways.^17^The enrichment of cell junction-related pathways suggests a possible role in tumor adhesion and metastasis, further highlighting the multifaceted impact of the gene signature in LARC progression and treatment response. This aligns with recent findings that EGFR activation during acute bouts of colitis may reduce the long-term burden of colitis-associated cancer, emphasizing the complex and context-dependent role of these pathways.^18^

The spatial transcriptomic analysis added another layer of depth to our findings. By evaluating gene expression in distinct tissue compartments (tumor and stroma), we identified eight genes significantly associated with treatment response in both compartments. This is particularly relevant, as it provides a clearer understanding of the tumor microenvironment’s role in modulating response to nCRT. This approach is supported by recent studies using the fluorescence-Raman endoscopic system (FRES) for the simultaneous detection of EGFR and VEGF in colorectal cancer, which demonstrated the importance of considering both tumor cells and their microenvironment.^19^

Notably, a greater number of tumor-associated genes were linked to treatment response compared to stroma-associated genes, indicating that the tumor compartment holds more predictive value in our cohort. This finding is consistent with the current understanding of EGFR-mediated signaling pathways in colorectal cancer, which play a crucial role in tumor cell proliferation and influence cellular activities such as nuclear transcription and mitochondrial metabolism.^20^

The weak correlations between our gene set and previously published signatures further emphasize the distinctiveness and potentially novel insights provided by our approach. This difference could be attributed to variations in the datasets and methodological approaches used in developing these signatures, highlighting the challenges in developing universally applicable biomarkers in oncology. It also underscores the complexity of resistance mechanisms to anti-EGFR therapies in metastatic colorectal cancer, which involve multiple signaling pathways and molecular alterations.^21^This underscores the need for future research to explore the therapeutic targeting of these pathways, particularly in the context of immune checkpoint inhibitors. Such investigations could potentially lead to more effective combination therapies and personalized treatment strategies for patients with locally advanced rectal cancer.

The genes identified in our analysis are known to be associated with cancer and, in some instances, also with therapeutic sensitivity. Genes associated with an immunosuppressive state (*ALDH2*, *TP53,* or *DHRS3*) are associated with poor response, whereas genes associated with increased tumor activity (*UAP1L1*) are associated with a better response to treatment. Half of rectal cancers present a mutation in the *TP53* gene.^22^ In addition to playing important roles in tumor development and progression, *TP53* appears to be linked to response to treatment.^23^^,^^24^ In response to chemotherapy and radiation, most cancer cells harboring p53 mutations show a reduced sensitivity compared to cells lacking p53 or those with wild type p53.^25^ *TP53* was found to be highly expressed among non-responders in the tumor compartment. *TMCO6* was found to be associated with a lack of response to nCRT in the stroma compartment. *TMCO6* is associated with poor prognosis in several cancers including CRC.^26^ In addition, in hepatocellular carcinoma, *TMCO6* interacts with NET-DNA and suppressed T cell receptor signaling, thereby impairing anti-tumor immunity and promoting cancer progression.^27^ Other genes, such as *ARPC3, POLR2H, WSB2,* and *ARL6IP5,* show opposite association with response in tumor and stroma compartments, respectively. The role of ARL6IP5 in the renewal and regeneration of intestinal epithelium is well described.^28^ Literature indicates that ARL6IP5 is also associated with angiogenesis, cell proliferation, apoptosis, metastasis, and resistance to chemotherapy in gastric, ovarian, and breast cancer.^29^ Yet, its prognostic and predictive role might depend on whether it is expressed by tumor cells or within the stroma compartment. Lastly, ALDH2 is found to be highly expressed by tumor cells compared to the stroma. In our cohort, it is associated with non-responders. ALDH2 was found to be associated with cancer stemness and metastasis in CRC through activating β-catenin signaling.^30^ β-catenin (*CTNNB1*) is one of the markers that came up from this analysis (Figure S4). Studies show a positive correlation between *ALDH2* expression and immune cell infiltration, in particular of CD3^+^ T cells and CD8^+^ T cells in the tumor microenvironment of melanoma, hepatocellular carcinoma and colon cancer.^31^^,^^32^^,^^33^ ALDH2 was also found to be positively correlated with PD-L1 expression in tumor tissue, indicating that these immune cells have suppressive features.

The genes *KRT17*, *CD55*, and *PPBP*, previously identified by Zhang et al.,^7^ have emerged as potential predictors of treatment response in rectal cancer, each offering unique insights into tumor biology and treatment efficacy. However, our data did not show significant correlations between these genes and treatment response, highlighting the potential variability in transcriptomic signatures across studies.

KRT17’s association with T-lymphocyte infiltration suggests its role in enhancing anti-tumor immune responses.^34^ In colorectal cancer, KRT17 has been linked to better clinical outcomes and increased T-lymphocyte infiltration, potentially reversing immune escape mechanisms.^34^ CD55, a regulator of complement activation, is frequently overexpressed in colorectal cancers and linked to poor prognosis, with its inhibition showing promise as a treatment strategy.^35^^,^^36^ While PPBP’s involvement in platelet activation hints at its potential relevance, its specific predictive value in rectal cancer is less established.

This discrepancy between our findings and previous studies underscores the importance of larger, more comprehensive investigations to validate potential biomarkers. The variability observed may arise from differences in sample size, classification criteria, and transcriptomic coverage across studies. These factors emphasize the need for standardized approaches and robust validation efforts in biomarker discovery for rectal cancer treatment response.

Despite the promising findings, there are several limitations to this study. First, despite the careful curation of multiple datasets, technical variation may limit the generalizability of our results. Indeed, the predictive performance was lower in an unseen dataset, and prospective clinical validation is necessary. Although the spatial data demonstrated very interesting interactions between the role of some genes and the compartment of their expression, they should be explored in larger patient populations to increase descriptive and predictive power. In particular, *in vitro* and *in vivo* validation would further elucidate the mechanistic links. Our integrative approach provides a biologically grounded and clinically motivated framework.

In conclusion, this study identified a gene signature predictive of response to nCRT in patients with LARC based on a large collection of patients. Incorporating a broader range of datasets allowed us to develop a signature with potentially wider applicability. Its association with key biological pathways and better performance over existing signatures highlight its promise as a tool for guiding personalized treatment strategies. However, further validation and exploration of spatial transcriptomics in larger cohorts are necessary to fully realize the clinical applicability of these findings.

### Conclusion

This study presents a gene signature predictive of response to neoadjuvant chemoradiotherapy (nCRT) in locally advanced rectal cancer (LARC), leveraging an integrative analysis across multiple transcriptomic datasets. The predictive performance of this gene signature, with an AUC of 0.80, demonstrates its potential utility in stratifying patients with LARC for personalized treatment approaches. Importantly, this signature shows significant associations with molecular subtypes, notably the consensus molecular subtype (CMS4) and immune classification subtype (iCMS3), both of which were enriched in responders, suggesting a robust connection to the biological characteristics of therapy response.

Spatial transcriptomic profiling added valuable insights by identifying compartment-specific biomarkers, with tumor-associated genes exhibiting greater predictive value than those in the stroma, underscoring the importance of intratumoral heterogeneity in therapeutic outcomes. These findings not only complement the bulk transcriptomic results but also highlight the potential of spatial transcriptomics in refining biomarker-based predictions.

While this study provides a promising framework for predicting nCRT response in LARC, further validation in larger, independent cohorts and continued exploration of spatial transcriptomics are essential to establish the clinical applicability of these findings. Ultimately, this approach could support more effective, tailored treatment strategies, reducing unnecessary interventions for non-responders while promoting intensified therapies for those likely to benefit.

### Limitations of the study

While this study identifies a promising gene signature predictive of response to neoadjuvant chemoradiotherapy in locally advanced rectal cancer, certain aspects warrant further consideration. The integration of publicly available datasets, although expanding cohort size and diversity, introduced technical variation and batch effects that could not be fully resolved. Nevertheless, the consistency of results across independent datasets and the use of robust cross-validation provide confidence in the findings. The spatial transcriptomic analysis was conducted in a moderate-sized cohort, which, while offering valuable compartment-specific insights, would benefit from replication in larger studies. Finally, functional validation of the identified biomarkers was beyond the scope of this work, but represents an important next step to establish mechanistic links. Overall, these limitations highlight opportunities for future research and do not diminish the strength of the exploratory framework presented here.

## Resource availability

### Lead contact

Further information and requests for resources, data, or materials should be directed to the Lead Contact: Thibaud Koessler (thibaud.koessler@hcuge.ch).

### Materials availability

This study did not generate new unique reagents or materials.

### Data and code availability

• Data: Raw sequencing data derived from human samples cannot be publicly shared due to institutional ethical regulations and patient privacy restrictions. De-identified processed single-cell and spatial transcriptomic files generated in this study have been deposited in the Gene Expression Omnibus (GEO) and are publicly available as of the date of publication. The spatial transcriptomic dataset is available under the accession number GSE279942, and additional transcriptomic datasets analyzed in this study are available under the accession numbers listed in the Supplementary Tables and referenced in the key resources table.
• Code: Custom code used for data preprocessing, clustering, and machine-learning analyses has been made publicly available at Zenodo and is accessible without restriction at the following DOI: https://doi.org/10.5281/zenodo.17207884.
• Other: This study did not generate additional datasets or software requiring availability statements.

## Acknowledgments

The authors would like to acknowledge the GTF UNIL core facility for their service. Ms. Garance Gutnecht, Mr. Sebastien Bugeia, and Ms. Karen Ducoli for their help with sample collection and processing. The authors would also like to thank the patients and their families for participating in this research project.

This work was supported by the Research Fund of the Department of Internal Medicine (HUG) and the Faculty of Medicine (UNIGE), the Fondation pour l’innovation sur le cancer et la biologie, and the Swiss Cancer League KFS-5786-02-2023.

## Author contributions

Conceptualization: C.C., J.V.M.C., P.T., and T.K. Methodology and data curation: C.C., J.V.M.C., P.A., M.T., A.B., and P.W. Investigation: C.C., J.V.M.C., M.R., P.A., S.T., and J.T. Formal analysis and visualization: C.C., J.V.M.C., M.R., P.A., P.W., and J.T. Resources and clinical samples: M.T., A.B., G.P., I.Z., A.D., F.R., and V.D. Writing – original draft: C.C., J.V.M.C., and M.R. Writing – review and editing: All authors. Supervision and funding acquisition: C.C., P.T., T.K., and M.P.

## Declaration of interests

The authors declare no competing interests.

## STAR★Methods

### Key resources table

**Table:** 

REAGENT or RESOURCE	SOURCE	IDENTIFIER
**Deposited data**		

RNA data	GEO repository	GSE94104
RNA data	GEO repository	GSE93375
RNA data	GEO repository	GSE150082
RNA data	GEO repository	GSE133057
RNA data	GEO repository	GSE279942
RNA data	GEO repository	GSE145037
RNA data	GEO repository	GSE209746
RNA data	GEO repository	GSE279942
RNA data	Domingo et al., EBioMedicine 2024	https://doi.org/10.1016/j.ebiom.2024.105228

**Software and algorithms**		

ComBat sva v.3.42.0	CRAN	https://doi.org/10.32614/CRAN.package.COMBAT
RUVcorr v.1.26.0	Bioconductor	https://doi.org/10.18129/B9.bioc.RUVcorr
limma v.3.50.3	Bioconductor	https://doi.org/10.18129/B9.bioc.limma
glmnet	CRAN	https://doi.org/10.32614/CRAN.package.glmnet
Random forest	CRAN	https://doi.org/10.32614/CRAN.package.randomForest

#### Public dataset collection and curation

##### Overview and process

We conducted a comprehensive search of the relevant literature in PubMed and GEO to identify publicly available datasets containing transcriptomic data from treatment-naive tumor samples in LARC patients. Out of the 11 studies identified, 6 met our criteria and were selected for further analysis. The datasets comprise 338 patients in total, who have undergone neoadjuvant chemoradiotherapy (nCRT) and were profiled by array or high throughput sequencing. Patients included in this cohort might have received different nCRT regimens.

Some samples were collected pre-treatment, while others post-treatment, resulting in non-technical sources of variation between those patients. To enhance the biological signal, we opted to exclude the post-treatment samples as we decided to focus on treatment-naïve tumors. Samples for which response information was unavailable were also excluded. The outcome of this filtering process resulted in a conclusive selection of 266 patients from the 6 studies.

Pathologic response after neoadjuvant radiation-based therapy was determined by three different TRG systems throughout the different studies; Mandard et al.,^37^ Edge et al. (AJCC)^38^ and Dworak et al.^39^ We suggested a TRG classification (Table S1) based on the relevant information of our datasets and pathologic response classification proposed by Trakarnsanga et al.^40^

All the studies employed for this project are publicly available and can be downloaded from NCBI GEO database with their accession number (Table S2).

### Experimental model and study participant details (GeoMx)

Fifty-three LARC patients were included into this study. All patients received nCRT as a standard of care treatment. Nevertheless, patients included in this cohort might have received different nCRT regimens. Tissue biopsies from these patients were collected during diagnostic procedure and underwent routine tissue processing and rapid sectioning for diagnostic purposes, including formalin fixation. Formalin-fixed and paraffin embedded (FFPE) blocks which were then used to build a next generation TMA (ngTMA). Two independent pathologists (M.T. and A.B.) in the Pathology Department at the University Hospital of Geneva reviewed all the tissue specimens and were involved in the region of interest (ROI) selection process for the ngTMA construction and GeoMx DSP. Clinical information for these patients was collected into the RedCap database. Response to treatment was classified as TRG according to Mandard scale. The study was approved by the ethics committee of the Geneva Canton (#2021-00700), and written informed consent was obtained from all participants. The median age of the cohort was 64 years (range 22–89 years), with 32 males and 21 females. All participants were of European ancestry. Information regarding race and ethnicity beyond ancestry was not collected in accordance with Swiss regulations and ethical guidelines.

### Method details

#### ngTMA construction

When FFPE blocks had a thickness below 3-4mm, samples were re-embedded. For tissue block re-embedding, the blocks were melted at 60°C into an adapted mould and additional paraffin was then added. Finally, after the sample was oriented properly, the block was cooled down to 4°C. Tumor regions were annotated on corresponding hematoxylin and eosin (H&E)-stained sections by a board-certified pathologist (M.T.). For each specimen, 1 representative tissue core of 0.6 mm diameter was assembled into next-generation TMAs (ngTMAs) using a Grand Master automated TMA (3DHISTECH). In addition, three tissue cores from normal rectal epithelia were included into each ngTMA as internal controls. TMA cores were digitally annotated by J.V. and L.D., under the supervision of P.B and I.Z. TMAs were sectioned at 2.5 um thickness, stained with H&E, and digitized using a Pannoramic P250 digital slide scanner (3DHistech). IHC was performed on a BOND-RX automated immunostainer (Leica Biosystems).

#### Transcriptomic analyses (GeoMx)

Transcriptomic analysis of the GeoMx cohort has been conducted in collaboration with the ILL Platform at the University of Lausanne using the GeoMx DSP technology. The FFPE tissue sections were incubated with the DNA probes Whole Human Transcriptome Atlas (WTA) 18′677 targeted genes). The region of interest (ROI) was selected using up to 4 morphologic antibodies combined with fluorochromes. These were: PanCytokeratin, CD45, Syto 83, CD31. Compartments were chosen for high-resolution multiplex profiling, and oligonucleotide tags from the selected region were released upon exposure to UV light. Photocleaved oligos were then collected via microcapillary tube inspiration into in microplates and analyzed using Ncounter platform or sequencing. Digital counts were then imported back into the GeoMx DSP instrument for QC and data analyses using the GeoMx DSP analysis suite version (v2.4). All raw data have also been exported for downstream analysis within R statistical language.

#### RNA digital profiling - Slides preparation

DNA oligonucleotide probes were designed to bind mRNA targets. From 5′ to 3′, they are each comprised of a 35- to 50-nucleotide target complementary sequence, an ultraviolet (UV) photocleavable linker and a 66-nucleotide indexing oligonucleotide sequence containing a unique molecular identifier (UMI), RNA ID sequence and primer binding sites.

FFPE tissue section of 5-μm were baked at 60°C for 1 hour. Slides were deparaffinized in 3 xylol baths of 5 min, then rehydrated in ethanol gradient from 100% EtOH, 2 baths of 5 min followed by 95% EtOH, 5 min. Slides were then washed in PBS 1X. Antigen retrieval was done in Tris-EDTA pH 9.0 buffer at 100°C for 15 min at low pressure. Slides were first dipped into hot water 10 seconds to be then dipped into Tris-EDTA buffer. Cooker vent stays open during the procedure to ensure low pressure and reach 100°C. Slides were then washed in PBS 1X, and incubated in proteinase K in PBS (1 ug/mL) for 15 min at 37°C and washed again in PBS 1X. Tissues were post-fixed in 10% neutral-buffered formalin 5 min, washed 2 times 5 min in NBF stop buffer (0.1M Tris Base, 0.1M Glycine) and finally one time in PBS 1X. The mix of Whole Transcriptome Atlas (WTA) probes was dropped on each section and covered with HybriSlip Hybridization Covers. Slides were then put for hybridization overnight at 37°C in a Hyb EZ II hybridization oven (Advanced cell Diagnostics). The day after, HybriSlip covers were gently removed and 25-min stringent washes were performed twice in 50% formamide and 2X SSC at 37°C. Tissues were washed for 5 min in 2× SSC, then blocked in Buffer W (Nanostring Technologies) for 30 min at room temperature in a humidity chamber. Next, 500 nM Syto13 and antibodies targeting PanCK and CD45 (Nanostring Technologies) in Buffer W were applied to each section for 1 h at room temperature. Slides were washed twice in fresh 2× SSC then loaded on the GeoMx Digital Spatial Profiler (DSP).

#### DSP collection

Entire slides were imaged at ×20 magnification and morphologic markers were used to select the Region Of Interest (ROI) either using a circle or organic shapes. Automatic segmentation of ROI based on PanCK+ markers were used to defined Areas of Illumination (AOIs). This allowed to separate tumor cells (PanCK+) and cells around tumor (stroma, PanCK-). AOIs were exposed to 385 nm light (UV), releasing the indexing oligonucleotides which were collected with a microcapillary and deposited in a 96-well plate for subsequent processing. The indexing oligonucleotides were dried down overnight and resuspended in 10 μl of DEPC-treated water.

#### DSP readout

For RNA profiling, NGS sequencing platform was used as readout. Sequencing libraries were generated by PCR from the photo-released indexing oligos and AOI-specific Illumina adapter sequences, and unique i5 and i7 sample indices were added. Each PCR reaction used 4 μl of indexing oligonucleotides, 4 μl of indexing PCR primers, 2 μl of Nanostring 5X PCR Master Mix. Thermocycling conditions were 37°C for 30 min, 50°C for 10 min, 95°C for 3 min; 18 cycles of 95°C for 15 s, 65°C for 1 min, 68°C for 30 s; and 68°C for 5 min. PCR reactions were pooled and purified twice using AMPure XP beads (Beckman Coulter, A63881), according to the manufacturer’s protocol. Pooled libraries were paired-sequenced at 2 × 27 base pairs and with the single-index workflow on an Illumina HiSeq 3000/4000 instrument. FastQ files were converted into Digital Count Conversion (DCC) files according to manufacturer’s pipeline. DCC files are imported back into the GeoMx DSP instrument for QC and data analyses.

### Quantification and statistical analysis

#### Estimate and correct batch effect

Strong batch effects were found in an exploratory analysis. A Principal Components Analysis (PCA) showed that the samples are mostly grouped based on the expression profiling platform that was used in each study (Figure S1).

First, we re-annotated microarray probes by aligning probe sequences against a recent common annotation (gencode28). It has been shown that manufacturer’s annotation can vary over time and may differ from recent sources, making this a necessary step to reduce technical variation.^41^ The expression of each gene was mean-centered and an algorithm for removal of unwanted variance (RUV) was applied with CRC specific control genes, before putting all datasets together.^42^

This approach successfully attenuated the batch effect within the microarray datasets. However, merging these with the datasets profiled by high throughput sequencing (RNAseq) proved to be hard, and a batch effect correction was not possible without excessive loss of information. Different approaches (ComBat sva v.3.42.0,^43^ RUVcorr v.1.26.0,^44^^,^^45^ limma v.3.50.3^46^) were tested to attenuate technical variation, but did not fully resolve the batch effect, or produced inacceptable loss of information. Indeed, the distribution of the expression in the high throughput sequencing data is very different from that of the microarray data (Figure S1). We resolved to a more conservative approach, and we applied a log2(x+1) transformation to the raw counts followed by mean centering and standardization.

#### Unsupervised clustering and biological annotation

We performed hierarchical clustering based on correlation distance (https://gitlab.com/pwirapati/nclust) on the six datasets. The reference CMS classifier (https://github.com/Sage-Bionetworks/CMSclassifier) was used to predict the CMS subtypes in the samples (method=“SSP”; function SSP.predictedCMS). The results were plotted on heatmaps (Figure 1B) and annotated with the intrinsic CMS (iCMS) class of each sample retrieved from the iCMS classifier algorithm (SSC.DQ).^47^

#### Differential gene expression and feature selection

Amongst the 6 expression datasets selected, only four (GSE94104, GSE93375, GSE150082, GSE133057) had an annotation system enabling an accurate correlation with response. We applied the procedure described in the following paragraphs, at first to these 4 datasets, then we extended the analysis to all 6 datasets, in the attempt to obtain a list of potential markers that was both comprehensive and accurate.

We have run a differential gene expression analysis as a first exploratory analysis, and to select a reduced number of genes (feature selection) to supply to the machine learning models, we compared responders *versus* non-responders. For increasing the separation power between the two groups, we have excluded the pathological mid responders (pMR; Tumor Regression Grade (TRG) 3 according to Mandard) from the analysis and retained 226 samples for final analysis.

To minimize overfitting, all the following steps were performed with cross-validation. To test for differential gene expression, we have run a GLM model from the ‘limma’ R package^46^ inside a cross-validation loop. The data was split in 10 folds using the function createFolds from the ‘caret’ R package.^48^ The random sampling was performed within the two groups of the response variable, in an attempt to balance the class distributions between the splits). In each run, we combined 8 out of 10 folds (for a total of 45 runs). We included the batch as a covariate in the formula for the GLM model (∼ batch + response). We computed the maximum and average p-value of all the runs, for each gene. Imposing a threshold on the maximum p-value is equivalent to computing the intersection all the significant genes in each run. This method provides a set of high confidence differentially expressed genes. As input for the machine learning models, we opted for a less stringent selection of genes, imposing a threshold of 0.05 on the average (over the cross-validation runs) p-value. This choice is motivated by the idea of giving a larger search space to ML algorithms like glmnet,^49^ which provide an internal feature selection mechanism. Finally, we intersected the lists of differential expression genes from the two analyses (6 datasets and 4 datasets), obtaining a list of 186 genes that we fed to two ML algorithms to generate a signature predicting response to treatment in the chosen datasets.

#### Machine learning

We tested two classifier algorithms to predict the response to treatment. L2-regularized logistic regression glmnet and Random Forest^50^ methods have been evaluated with Area Under the ROC Curve (AUC) as a measure of the model performance. As input to the ML algorithms, we provided the mean centered and standardized expression of the 186 genes selected with the differential expression analysis. pMR (TRG 3) were also excluded. For each method, we have run a 5 folds cross-validation. The patients were split in 5 folds, and at each iteration 4 folds were used as a training set, and the out of the bag fold was used as a test set. For each algorithm, hyperparameters were tuned over 20 iterations (tuneLength = 20) on the training set to maximize the Area Under the ROC Curve (AUC). We obtained 5 models and 5 measures of the performance per method. We estimated the performance of each method and of our selected set of genes taking the average and standard deviation (for the confidence interval) of the AUC from each cross-validation iteration (Figures 1C and 1D). Clinicopathological variables were excluded to assess the standalone predictive value of the gene signature, though future multivariate analyses are needed to determine its independence from clinical factors.

#### GeoMx data analysis

GeoMX transcriptomic data was used to identify genes associated with response to treatment according or independently from their spatial localization (tumor or stroma segments). Raw DCC files were processed into a GeoMxSet object using the GeomxTools R package (v 3.6.2). Segment Quality Control: Segments were flagged and removed if they failed to meet the following study-specific criteria: minimum of 1000 raw reads, >80% reads trimmed, >80% reads stitched, >75% reads aligned, >50% sequencing saturation, minimum negative control count of 1, maximum NTC count of 9000, a minimum of 20 nuclei, and a minimum area of 1000 μm^2^.

Probe Quality Control: Probes were flagged for removal based on global and local outlier detection using Grubb’s test. A probe was removed globally if its geometric mean count was less than 10% of the mean for all probes for that target or if it was flagged as an outlier in over 20% of segments. Local outliers were also removed.

Aggregation and Filtering: Gene-level counts were generated by calculating the geometric mean of the remaining probes for each target using the aggregateCounts function. The Limit of Quantification (LOQ) was established for each segment as 2 geometric standard deviations above the geometric mean of negative control probes. Segments with a gene detection rate (genes with counts > LOQ) of less than 10% were removed. Subsequently, genes detected in fewer than 10% of the remaining segments were filtered from the dataset. Normalization: The final dataset was normalized using the third-quartile (Q3) method via the normalize function in GeomxTools.

We fitted a mixed linear model for gene expression looking for association to the response alone or together with localization. Patient IDs were added as random effects to account for patient and batch technical variability. We used the DREAM method from the R package VariancePartition^51^ to fit the mixed linear model, and to extract the statistical test result table. Reported p-values are adjusted for multiple testing with the Benjamini-Hochberg procedure. The bootstrap method was used to reduce overfitting, and to obtain a more robust list of genes. We resampled the patients five times and re-fit the random effect model for each resampling. The lists of significant genes obtained with the five runs were compared, and only the genes that were present in all five lists were reported as associated to response, alone or together with localization. In each bootstrap run, more than 400 genes had significant difference in responder vs non-responder, yet only 28 genes were consistently reported in all five bootstrap runs (Figure S4B). The low number of consistent genes suggests that the results are quite sensitive to patient sampling, possibly as a consequence of limited sample size. The averaged measurements of tumor and stroma segments were calculated to give a pseudo bulk estimate of the data. Expression of the samples that had information on either one or the other segment didn’t change. A log2 transformation was applied afterwards. The GeoMx expression data are available in the GEO repository (accession number GSE279942), where CK+ refers to the tumor segment and CK- to the stroma segment. Genes with low expression, including some genes from previously published signatures^5^ were filtered according to the Nanostring guidelines. Currently, lack of publicly available spatial transcriptomic datasets in LARC limits external validation opportunities; however, our findings provide a unique reference point for future comparative studies.
