# Supplementary material for: Integrative analysis of transcriptomic data reveals a predictive gene signature for chemoradiotherapy response in rectal cancer
Source: iScience. 2025 Dec 17;29(1):114455. doi: 10.1016/j.isci.2025.114455 (PMC12803930; doi:10.1016/j.isci.2025.114455)
Supplement: Document S1. Figures S1–S5, Tables S1–S3, and Data S1 [file mmc1.pdf]

iScience, Volume *29*

Supplemental information

Integrative analysis of transcriptomic data reveals a predictive gene signature for chemoradiotherapy response in rectal cancer

Claudia Corrò, Joao Victor Machado Carvalho, Melivoia Rapti, Paolo Angelino, Matthieu Tihy, Arnaud Bakaric, Giacomo Puppa, Pratyaksha Wirapati, André Durham, Frederic Ris, Stephanie Tissot, Jonathan Thevenet, Inti Zlobec, Valérie Dutoit, Mikael Pittet, Petros Tsantoulis, and Thibaud Koessler

Supplemental items

Supplemental figure legends

Supplementary Figure 1. Batch effect assessment and normalization across transcriptomic datasets. Principal component analysis (PCA) plots showing sample clustering before (a) and after normalization (b). Comparison of expression distributions across microarray and RNA-seq datasets illustrating platform-related variation.

Supplementary Figure 2. Molecular subtype distribution across responders and non-responders. (a) Frequency of consensus molecular subtypes (CMS) among responders and non-responders. (b) Frequency of immune subtypes (iCMS) among responders and non-responders.

Supplementary Figure 3. External validation of the 186-gene signature. Receiver operating characteristic (ROC) curve showing predictive performance of the gene signature on the independent dataset published by Domingo et al. (2024).

Supplementary Figure 4. Compartment-specific analysis of spatial transcriptomic data. (a) Genes significantly associated with response across tumor and stroma compartments. (b) Interaction analysis showing genes with compartment-dependent associations with treatment response. Statistical analysis was performed using an unpaired two-tailed Student's t-test. Data are presented as mean ± SEM. *p < 0.05, **p < 0.01, ***p < 0.001, ****p < 0.0001.

Supplementary Figure 5. Gene set enrichment analysis (GSEA) of spatial transcriptomic compartments. (a) Enriched pathways associated with treatment response in the stroma compartment. (b) Enriched pathways associated with treatment response in the tumor compartment.

Supplementary Table 1. Tumor regression grade (TRG) classification systems. Overview of the TRG systems (Mandard, AJCC, Dworak) used across datasets and the unified classification applied in this study.

Supplementary Table 2. Publicly available datasets included in the analysis. List of GEO accession numbers and study details for the datasets used in this study.

Supplementary Table 3. Compartment-specific genes associated with response. Genes identified as significantly associated with treatment response in tumor and stroma compartments based on spatial transcriptomic analysis.

Supplementary Data 1. Pathways identified in compartment-specific analyses. Complete list of pathways enriched in tumor and stroma compartments based on bootstrap-supported differential expression analysis.

Supplementary Figure 1

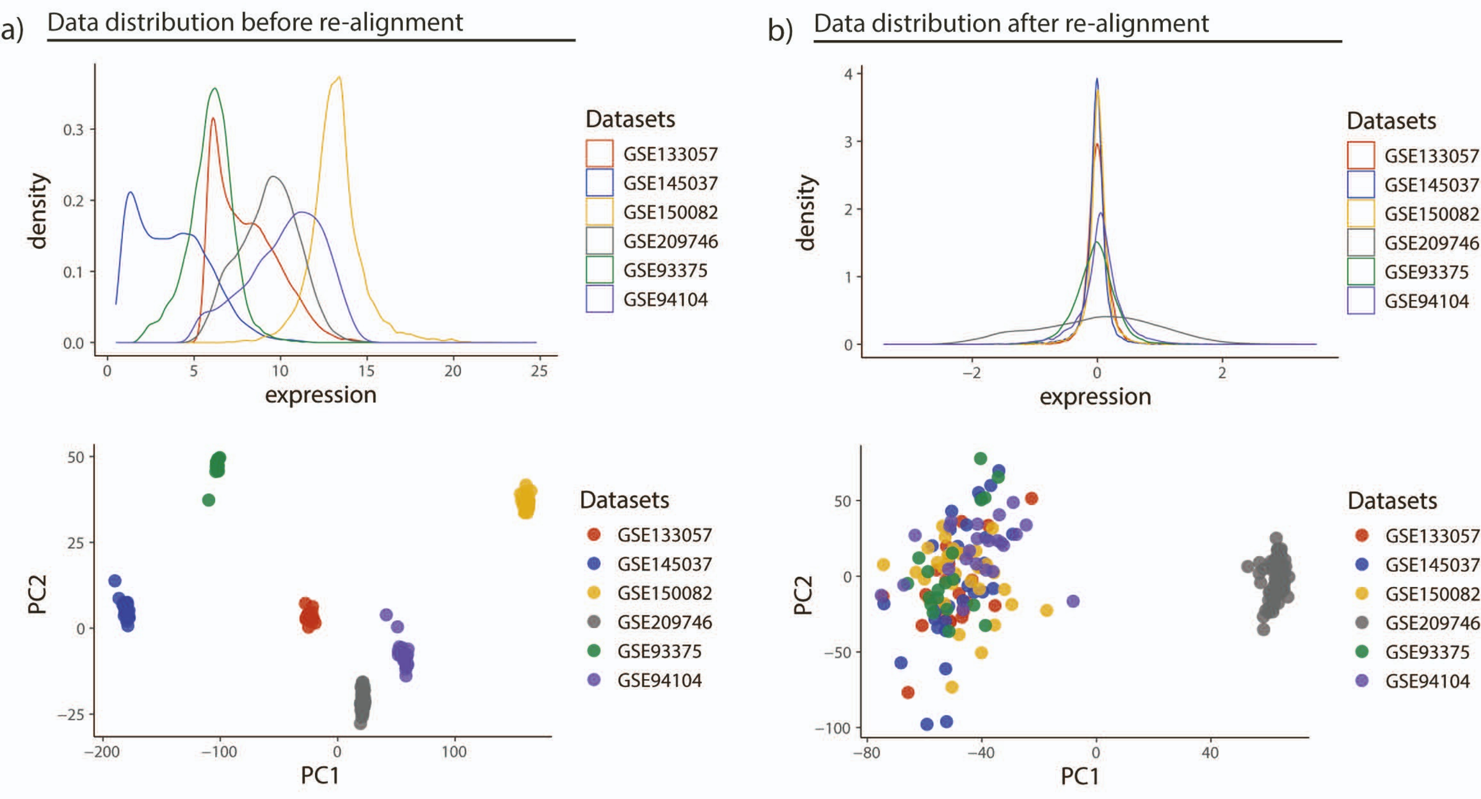

Supplementary Figure 2

a) Proportion of the four CMS subtypes in responders vs non-responders

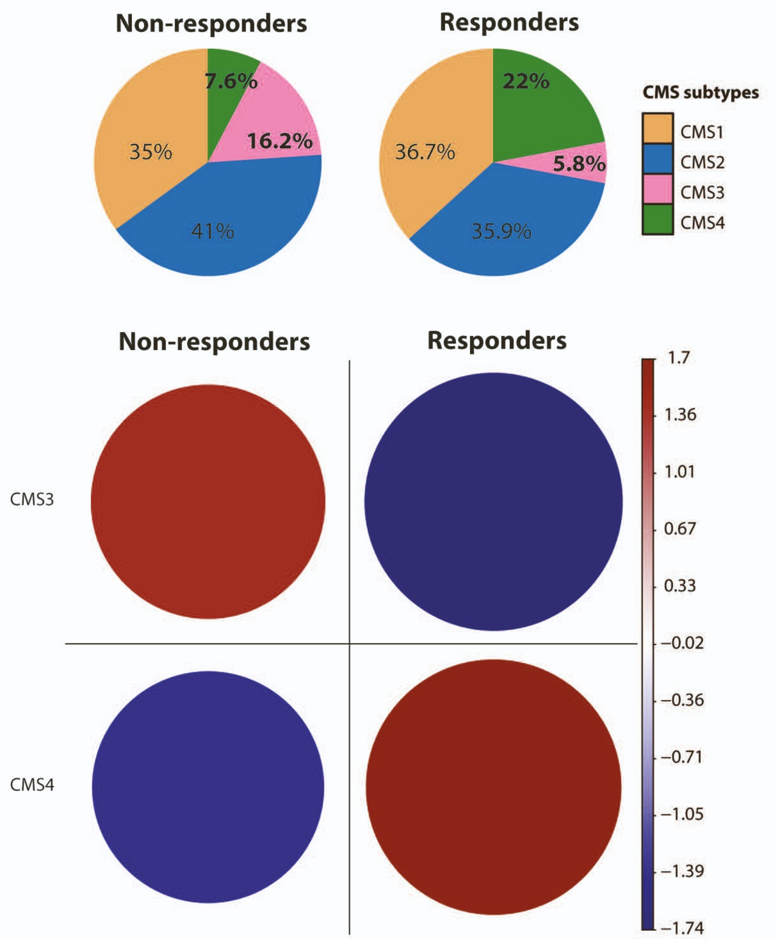

b) Proportion of the two iCMS subtypes in responders vs non-responders

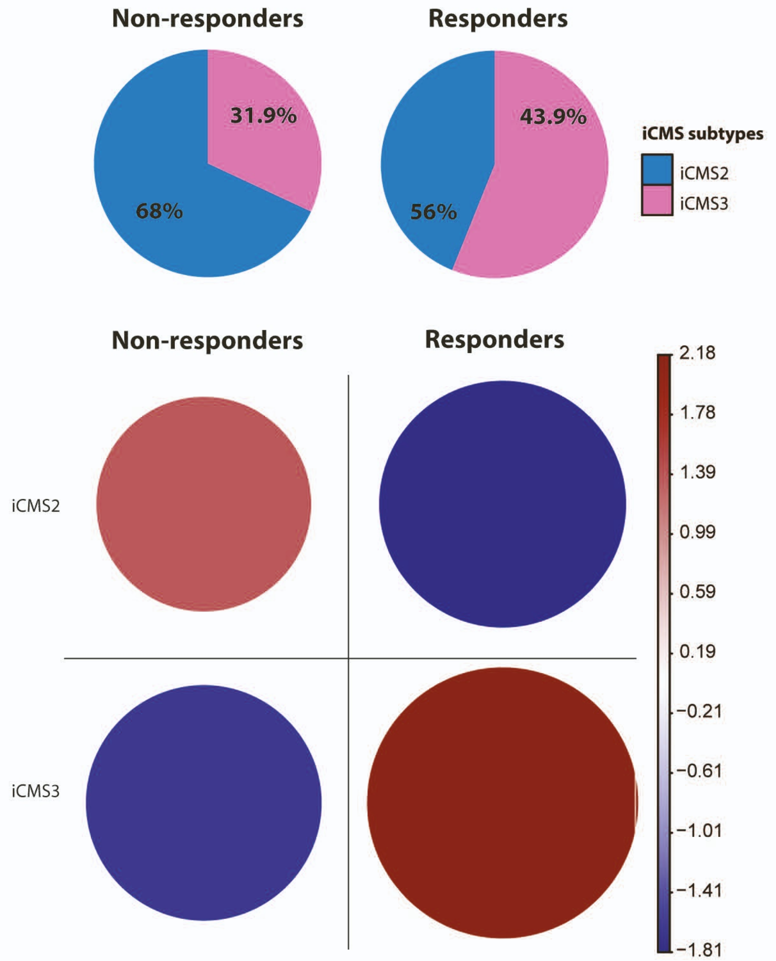

Supplementary Figure 3

a) ROC curve using the Random Forest model for the Domingo et al., 2024 signature

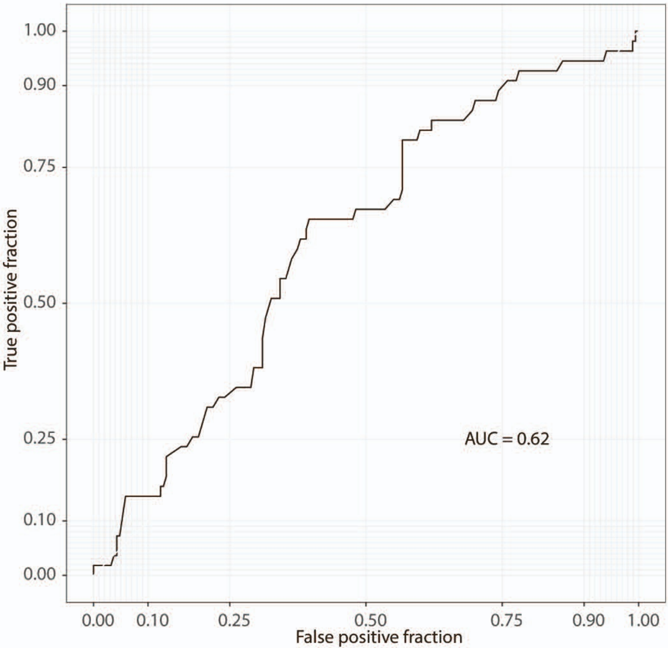

Supplementary Figure 4

a) Differential expression analysis of the GeoMx data for the genes that are associated with response

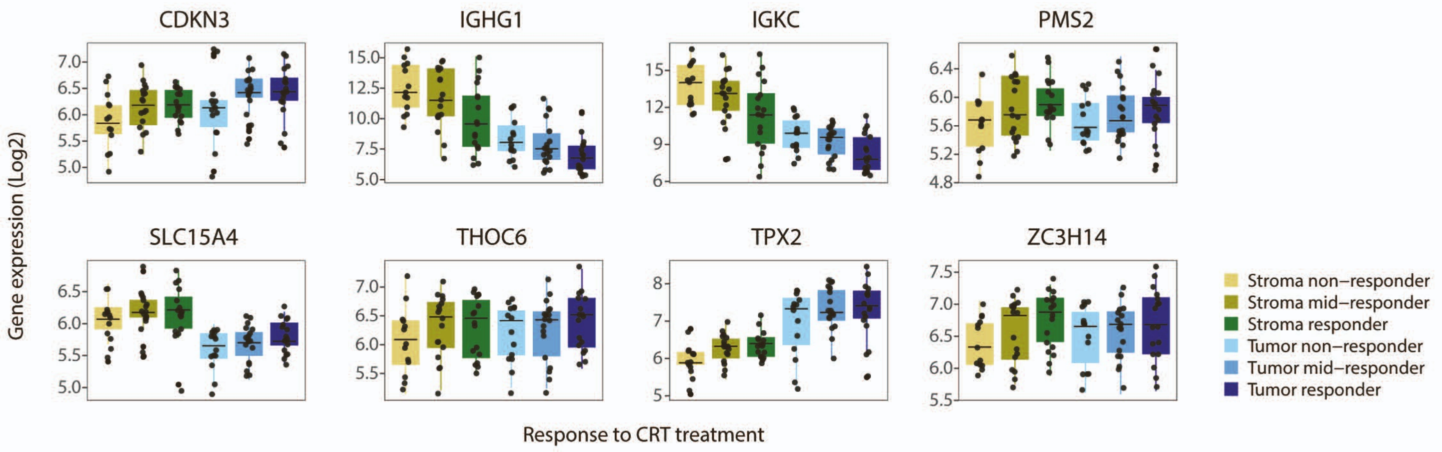

b) Differential expression analysis of the GeoMx data for the genes that are associated with response and localization

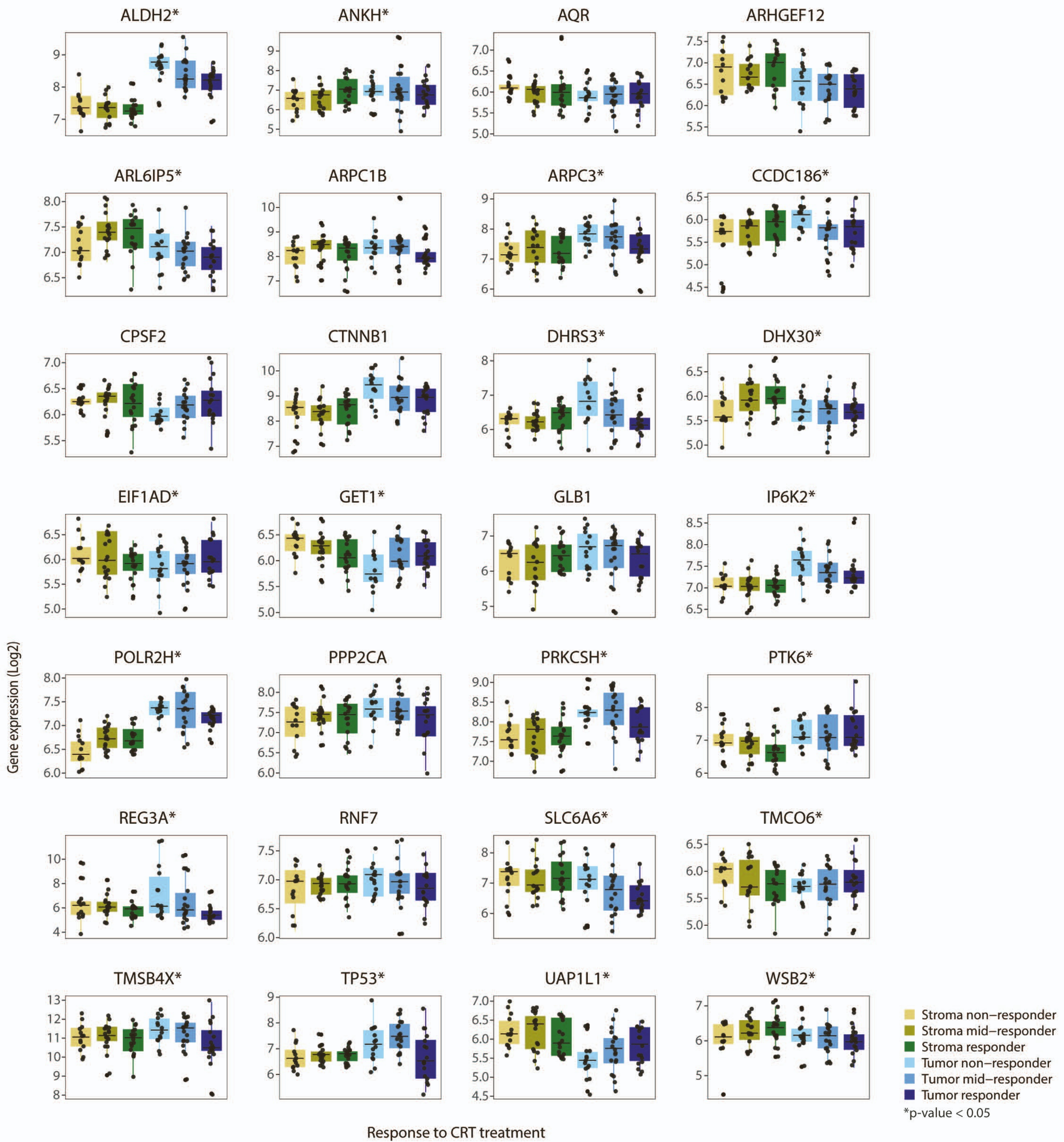

Supplementary Figure 5

a) GSEA analysis of the stroma compartment

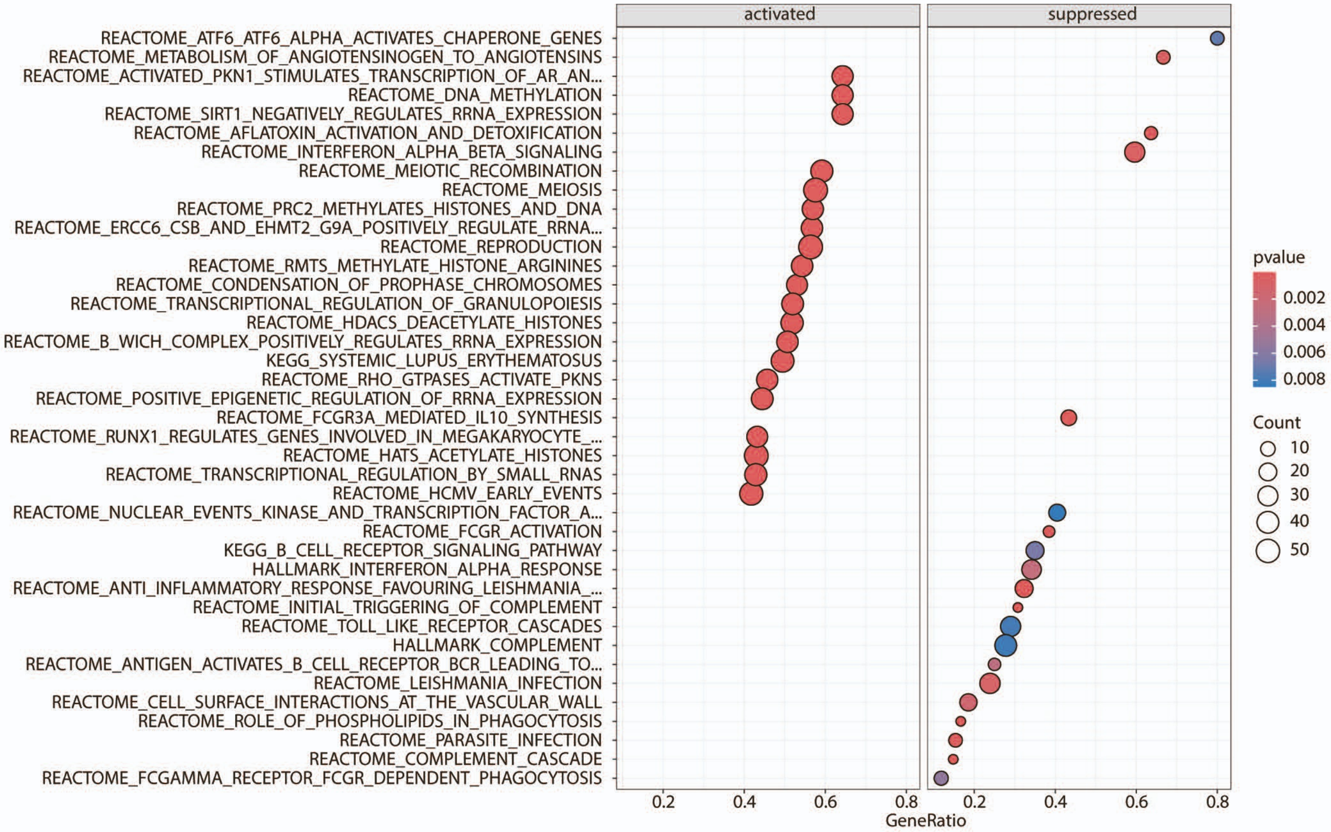

b) GSEA analysis of the tumor compartment

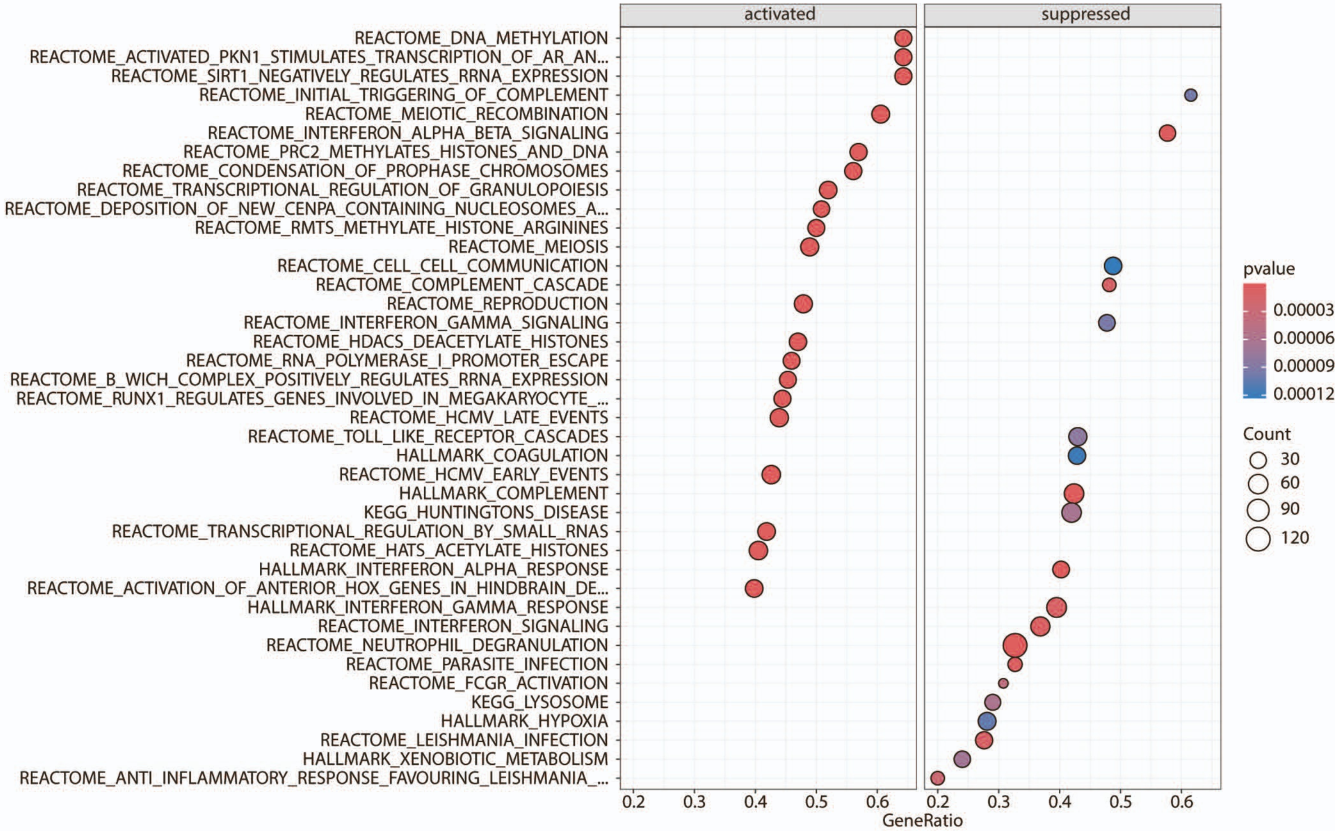

Supplementary Table 1

Tumor regression grade (TRG) classification systems.

	Non-pCR	Partial response	pCR
Mandard	4,5	3	1.2
Dworak	0,1	2	3,4
AJCC	2,3	1	0

Supplementary Table 2.
Publicly available datasets included in the analysis.

Accession Number	Initial Number of patients	Neoadjuvant Th	Sequencing Platform	TRG Score
GSE133057	33	CRT	Illumina	AJCC
GSE150082	38	CRT	Agilent	AJCC
GSE94104	80	CRT	Illumina	Dworak
GSE93375	42	CRT	Affymetrix	Mandard
GSE145037	31	CRT	Affymetrix	AJCC
GSE209746	114	CRT	HTS	-

Supplementary Table 3.
Compartment-specific genes associated with response.

gene	direction_in_tumor	direction_in_stroma	significant_in_tumor	significant_in_stroma
ALDH2	-	-	yes	no
ANKH	-	+	no	yes
AQR	+	-	no	no
ARHGEF12	-	+	no	no
ARL6IP5	-	+	yes	no
ARPC1B	-	+	no	no
ARPC3	-	+	yes	no
CCDC186	-	+	yes	no
CPSF2	+	-	no	no
CTNNB1	-	+	no	no
DHRS3	-	+	yes	no
DHX30	-	+	no	yes
EIF1AD	+	-	yes	no
GET1	+	-	no	yes
GLB1	-	+	no	no
IP6K2	-	-	yes	no
POLR2H	-	+	no	yes
PPP2CA	-	+	no	no
PRKCSH	-	-	yes	no
PTK6	-	-	no	yes
REG3A	-	-	yes	no
RNF7	-	+	no	no
SLC6A6	-	+	yes	no
TMCO6	+	-	no	yes
TMSB4X	-	-	yes	no
TP53	-	+	yes	no
UAP1L1	+	-	yes	no
WSB2	-	+	no	yes

Legend
- = downregulated in responders
+ = upregulated in responders

	logFC_1	logFC_2	logFC_3	logFC_4	logFC_5	adj.P.Val_	adj.P.Val_	adj.P.Val_	adj.P.Val_	adj.P.Val_	Rank_1	Rank_2	Rank_3	Rank_4	Rank_5
COL4A2	-7.00055	-7.05455	-6.70832	-7.03686	-6.68921	2.12E-28	3.82E-30	2.28E-31	1.71E-33	1.93E-26	1	7	2	6	29
ELF3	4.317301	3.939161	4.25971	4.006069	4.205182	2.32E-28	4.09E-27	2.83E-29	6.90E-29	1.85E-26	2	19	7	22	28
EPCAM	5.019488	5.015456	5.175731	5.111503	4.766508	4.42E-28	3.62E-27	6.91E-30	1.58E-29	1.06E-28	3	18	4	12	11
EVL	-3.82973	-3.68732	-3.8452	-3.84512	-3.56964	1.17E-27	1.24E-27	1.32E-27	2.47E-27	2.73E-26	4	14	23	40	31
TRBC1	-3.63664	-3.35879	-3.55964	-3.74524	-3.20893	1.17E-27	1.59E-21	1.91E-23	6.14E-27	7.35E-15	5	145	140	46	955
ACSL5	2.772493	2.83411	2.914387	3.073882	2.950457	1.58E-27	7.94E-21	1.28E-25	7.04E-22	1.03E-25	6	178	70	215	40
SPARC	-7.20744	-7.12386	-6.62866	-7.29504	-7.15866	1.59E-27	2.50E-30	9.75E-30	8.71E-35	6.25E-31	7	4	5	3	2
VIM	-7.33779	-7.44621	-6.89698	-7.62625	-7.47577	1.69E-27	4.75E-30	6.79E-32	3.37E-35	9.23E-31	8	8	1	1	3
PECAM1	-4.90475	-4.95699	-5.05489	-4.99241	-4.91084	2.57E-27	1.36E-27	3.95E-29	1.58E-29	4.61E-25	9	15	9	14	58
LSP1	-3.77177	-3.30007	-3.86714	-3.58257	-3.99751	2.83E-27	8.32E-25	1.23E-27	3.04E-25	2.18E-25	10	36	20	78	49
COL3A1	-6.76806	-6.7862	-6.20054	-6.76658	-6.73013	7.84E-27	1.46E-29	3.26E-29	1.09E-33	1.54E-29	11	10	8	4	7
COL4A1	-7.36511	-7.63378	-7.1586	-7.69379	-7.06781	9.05E-27	9.42E-33	4.00E-29	8.71E-35	4.01E-27	12	1	12	2	17
BGN	-6.50092	-6.55762	-5.8159	-6.65009	-6.70778	1.04E-26	1.21E-29	2.21E-29	2.21E-31	6.36E-29	13	9	6	8	10
CDH1	4.987827	4.547297	4.740102	4.73798	4.535992	1.12E-26	3.73E-24	2.10E-25	7.89E-24	2.51E-26	14	52	74	118	30
TMPRSS4	3.601032	3.613239	3.829173	3.855867	3.648322	5.42E-26	1.00E-27	1.07E-28	7.40E-26	1.29E-29	15	11	13	66	5
CEACAM1	5.079471	4.900363	5.225148	5.248385	4.629607	5.42E-26	1.03E-22	1.28E-25	2.83E-27	6.35E-24	16	100	69	41	87
FXYD3	4.478553	4.383086	4.432957	4.586371	4.262777	1.34E-25	5.38E-23	3.05E-25	8.62E-27	1.94E-20	17	88	79	49	230
CD24	4.347584	4.150416	4.520615	4.324056	3.859062	1.34E-25	4.82E-24	2.24E-26	5.23E-24	4.52E-20	18	56	47	108	261
SRGN	-4.03129	-4.0064	-4.33675	-4.0753	-4.01885	1.39E-25	7.83E-22	2.15E-28	9.19E-23	3.92E-20	19	132	16	168	250
IGFBP7	-7.35699	-7.44714	-6.87404	-7.54959	-7.15438	1.53E-25	7.49E-31	3.95E-29	1.71E-33	2.44E-29	20	2	10	5	8
CEACAM5	5.745833	5.630886	6.173987	5.931222	5.594916	2.17E-25	1.68E-24	1.41E-27	7.93E-27	4.56E-22	21	43	24	47	127
NNMT	-3.90323	-3.71492	-3.98275	-3.64671	-3.98007	4.33E-25	4.47E-19	2.76E-25	1.23E-22	3.32E-23	22	310	76	173	101
C1QC	-5.27798	-5.56041	-5.29845	-5.59319	-5.37958	5.33E-25	4.67E-25	1.60E-25	3.76E-26	1.65E-24	23	34	73	59	70
CLDN4	4.860573	4.810846	4.782299	4.790571	4.959388	5.33E-25	2.33E-25	3.95E-26	9.73E-28	7.47E-26	24	30	50	34	38
COL6A3	-6.38475	-6.18462	-5.79525	-6.28708	-6.48426	5.97E-25	1.35E-23	4.95E-26	1.26E-26	2.24E-27	25	67	55	51	14
POF1B	3.610653	3.507432	3.559717	3.679959	3.525379	5.97E-25	3.43E-24	3.14E-26	3.33E-29	5.20E-29	26	51	48	18	9
EMP3	-3.74211	-3.77889	-4.11262	-3.88205	-4.2042	6.05E-25	2.61E-23	1.65E-26	1.92E-25	5.77E-27	27	78	44	74	18
FGL2	-3.66934	-3.65483	-4.17548	-3.95279	-3.48233	6.76E-25	1.18E-20	2.91E-23	1.20E-20	4.68E-15	28	195	150	295	915
PHGR1	4.965375	4.952754	5.113008	4.904338	5.103339	7.08E-25	9.05E-25	8.09E-26	5.31E-27	6.02E-26	29	38	63	44	35
KLF5	4.308353	3.897156	4.462831	4.174811	4.484616	8.33E-25	1.55E-22	1.04E-25	1.02E-23	6.25E-31	30	108	65	126	1
PTPRC	-3.58273	-3.30579	-3.68567	-3.55775	-3.52695	8.49E-25	6.59E-24	7.39E-27	4.08E-23	5.81E-20	31	59	36	147	267
LLGL2	3.149895	3.182265	2.933423	3.118487	2.913601	1.37E-24	1.24E-27	2.78E-27	1.59E-26	1.95E-23	32	13	26	53	99
A2M	-5.91056	-5.8973	-5.70576	-6.29682	-6.18717	1.37E-24	2.50E-30	1.01E-26	6.06E-33	1.29E-29	33	5	39	7	6
PPP1R18	-2.68936	-2.63088	-2.8206	-2.61457	-2.78206	2.00E-24	3.92E-21	1.41E-21	1.59E-22	6.23E-20	34	163	247	180	268
HNF4A	3.383321	3.161997	3.508467	3.328851	3.162388	2.05E-24	2.49E-22	1.09E-27	1.31E-23	4.24E-21	35	115	19	133	183
IGKC	-8.14324	-7.50756	-7.29557	-7.42698	-6.66943	2.27E-24	6.41E-20	5.09E-21	4.58E-20	4.29E-16	36	247	305	327	743
RAB34	-3.21381	-2.8707	-3.07257	-2.95307	-3.15187	2.37E-24	7.98E-18	2.09E-24	1.18E-20	1.84E-24	37	420	101	293	72
PARVG	-2.4353	-2.10877	-2.761	-2.26578	-2.423	2.37E-24	4.76E-23	1.65E-26	7.01E-21	4.33E-18	38	86	43	279	443
GMFG	-2.95831	-2.87831	-3.21094	-3.07882	-2.94245	2.81E-24	2.28E-23	2.12E-26	2.94E-24	1.81E-19	39	74	46	98	314
PLEKHO1	-3.68942	-3.44913	-3.71049	-3.81224	-3.61576	2.83E-24	7.16E-23	4.03E-24	5.33E-27	4.09E-22	40	92	111	45	125
JAK3	-2.68375	-2.59175	-3.25147	-2.86559	-3.05843	3.26E-24	3.19E-23	5.84E-27	1.60E-24	2.14E-24	41	82	34	93	74
ADGRL2	-3.04389	-2.88598	-2.88946	-2.88525	-2.7771	3.29E-24	6.78E-23	6.39E-26	2.41E-25	3.17E-21	42	89	59	75	178
TCF4	-4.29738	-3.73683	-4.0007	-3.89794	-3.95283	3.29E-24	1.74E-19	4.16E-26	6.59E-26	1.97E-21	43	275	51	65	166
LGALS1	-5.40385	-5.53403	-5.17498	-5.59049	-5.43135	3.29E-24	1.80E-24	1.32E-27	2.83E-29	2.02E-25	44	47	21	17	48
AKAP1	2.839153	2.617638	2.773806	2.781545	2.824677	3.45E-24	3.67E-20	5.18E-22	3.39E-20	3.19E-24	45	226	218	319	78
STAB1	-4.46454	-4.63504	-4.73295	-4.59089	-4.18078	3.45E-24	2.65E-23	2.80E-27	7.71E-25	4.34E-20	46	79	27	86	257
SLC12A4	-2.43411	-2.34226	-2.22506	-2.31796	-2.26319	3.94E-24	1.31E-22	3.44E-22	2.59E-19	6.98E-20	47	105	209	397	278
PLXNC1	-2.44394	-2.36977	-2.92282	-2.60867	-2.5951	4.48E-24	3.07E-20	1.82E-23	8.77E-20	3.51E-20	48	219	136	345	249
TFF3	3.96106	3.611061	4.101298	4.129219	4.404187	4.51E-24	8.61E-20	2.02E-21	4.49E-19	1.94E-14	49	260	257	426	1053
COX6A1	2.754179	2.627425	2.726236	2.707793	2.614289	4.52E-24	3.52E-26	1.34E-24	9.44E-26	1.20E-26	50	24	96	69	25
LSR	3.951917	4.048324	3.950552	4.014082	3.708209	5.15E-24	5.92E-27	1.32E-27	4.55E-28	2.73E-24	51	20	22	31	75
SPI1	-3.38874	-3.60637	-3.57707	-3.82415	-3.65926	5.41E-24	2.61E-23	6.27E-25	5.29E-25	4.30E-25	52	76	89	83	57
PDPN	-4.45539	-4.14057	-4.27189	-4.27791	-4.46891	5.73E-24	4.16E-19	4.15E-23	2.28E-23	3.06E-22	53	306	159	137	117
LCP1	-3.77516	-3.70765	-3.87176	-3.98551	-3.97941	5.98E-24	7.03E-19	1.84E-28	5.30E-25	6.09E-24	54	328	15	84	85
COL5A2	-5.33805	-5.45567	-4.90135	-5.4872	-5.61517	6.37E-24	4.38E-25	1.43E-27	2.15E-28	5.11E-26	55	33	25	29	33
LAPTM5	-4.53237	-4.7407	-4.5596	-4.65384	-4.66342	6.37E-24	1.74E-24	7.54E-27	3.68E-25	1.25E-25	56	46	37	80	43
COL1A2	-6.98534	-7.28249	-6.24239	-7.37038	-7.15821	6.41E-24	1.11E-30	6.72E-27	2.78E-31	8.49E-30	57	3	35	10	4
CD3E	-2.13859	-1.802	-2.202	-2.12517	-2.42641	7.82E-24	9.68E-17	3.43E-19	8.31E-18	7.30E-19	58	573	443	566	365
C1QB	-5.049	-5.39413	-4.9189	-5.43439	-4.92154	8.38E-24	9.09E-24	2.51E-22	8.93E-24	7.53E-21	59	62	196	122	202
ENG	-5.09337	-5.26161	-5.1756	-5.2882	-4.98971	1.02E-23	1.44E-24	4.47E-26	2.24E-31	3.93E-22	60	39	53	9	124
LGALS4	4.540448	4.308198	4.865306	4.337272	4.546129	1.02E-23	4.44E-23	2.05E-26	7.13E-24	2.97E-26	61	84	45	114	32
ESRP1	2.765409	2.928818	3.390293	2.978983	3.102214	1.02E-23	2.18E-26	1.12E-26	1.10E-22	1.12E-26	62	22	40	170	24
SIPA1	-2.75164	-2.3764	-2.9766	-2.69646	-2.79319	1.13E-23	3.13E-23	1.17E-21	7.62E-24	1.74E-19	63	81	242	116	311
FMNL1	-2.7057	-2.51455	-2.81447	-2.65864	-2.34501	1.13E-23	2.71E-22	5.17E-27	2.65E-22	1.07E-09	64	116	33	191	2439
PLPP3	-2.60421	-2.54613	-2.62346	-2.45115	-2.43706	1.13E-23	2.61E-23	5.69E-25	3.67E-23	7.00E-20	65	77	85	145	279
C1S	-5.67173	-5.61834	-5.30382	-6.00986	-5.69375	1.18E-23	2.40E-26	4.95E-26	5.55E-29	1.11E-27	66	23	56	20	13
DCN	-5.4193	-5.2664	-5.00154	-5.46507	-5.70129	1.31E-23	3.85E-24	3.72E-26	2.55E-26	9.11E-27	67	53	49	57	21
FOXF1	-4.61711	-4.34039	-4.42556	-4.57481	-4.59828	1.39E-23	2.71E-22	6.47E-25	7.77E-25	7.93E-22	68	117	90	87	142
KRT18	4.130082	4.072435	4.042425	4.172296	4.374232	1.54E-23	1.10E-20	4.67E-24	1.62E-21	3.29E-27	69	190	114	243	16
HCLS1	-2.69308	-2.84845	-3.42362	-3.15103	-3.11008	1.59E-23	1.03E-22	6.47E-25	7.74E-21	1.88E-21	70	99	91	282	164
ACTA2	-5.95246	-5.91697	-5.85328	-5.95142	-5.85207	1.70E-23	4.79E-24	9.01E-27	1.50E-26	6.06E-26	71	55	38	52	36
MYL9	-4.57431	-4.83282	-4.86854	-4.86761	-4.95718	1.70E-23	2.17E-23	5.17E-27	1.40E-27	1.97E-25	72	73	32	36	45

GIMAP4	-2.1922	-2.14854	-2.24849	-2.54884	-2.17242	1.78E-23	3.77E-19	3.85E-18	4.66E-21	2.24E-18	73	302	574	270	413
PLVAP	-5.45347	-5.46965	-5.6672	-5.40662	-5.31587	1.95E-23	4.06E-24	5.96E-26	1.58E-29	8.84E-21	74	54	58	13	209
CLDN7	4.4988	4.313493	4.634807	4.291598	4.490577	2.07E-23	6.87E-25	1.07E-25	9.73E-28	6.17E-24	75	35	66	33	86
MEF2C	-2.18522	-2.01009	-2.37431	-2.30531	-2.40502	2.08E-23	9.46E-19	1.55E-18	6.92E-18	5.50E-21	76	334	511	556	192
SERPING1	-4.97864	-4.92154	-4.55033	-5.26323	-4.9209	2.15E-23	1.24E-27	6.49E-24	6.84E-29	1.75E-26	77	12	120	21	27
COL6A1	-6.14249	-6.25705	-5.63419	-6.4539	-6.63625	2.31E-23	6.26E-26	1.20E-25	1.82E-28	2.31E-27	78	26	68	27	15
EPS8L3	3.355136	3.204695	3.610753	3.303564	2.99928	2.32E-23	9.07E-23	3.21E-25	2.12E-26	1.20E-19	79	97	80	56	298
RIPOR1	-2.32271	-1.98467	-2.15859	-2.06852	-2.17492	2.58E-23	4.64E-22	2.76E-25	5.41E-22	2.21E-18	80	127	77	210	409
CD9	3.268941	3.247411	3.225753	3.423857	2.990062	2.81E-23	2.38E-22	8.98E-22	4.80E-26	9.50E-21	81	114	235	63	214
TSPAN8	4.948991	4.350521	5.195868	4.67702	4.795343	3.18E-23	1.43E-20	1.32E-26	1.78E-24	1.57E-26	82	206	42	94	26
AEBP1	-5.28737	-5.04535	-4.85006	-5.35578	-5.6616	3.33E-23	1.45E-23	3.75E-25	1.31E-23	2.71E-25	83	68	83	131	52
EVI2B	-2.68959	-2.5083	-3.04691	-2.70549	-2.95799	3.34E-23	1.54E-21	1.28E-22	1.39E-18	1.46E-16	84	144	181	470	669
EGFL7	-3.21782	-2.95452	-3.5598	-3.18415	-2.94191	3.82E-23	1.53E-17	7.45E-26	4.82E-23	9.46E-18	85	453	61	155	495
SOX9	3.632236	3.49676	3.765291	3.556748	3.653447	4.63E-23	2.39E-18	1.68E-24	9.79E-22	1.66E-25	86	369	98	222	44
MMP2	-5.77807	-5.88708	-5.21091	-6.08275	-6.3335	4.64E-23	7.12E-24	2.44E-25	1.86E-28	2.00E-25	87	61	75	28	47
SLC8A1	-3.25334	-2.96393	-2.99802	-3.17068	-2.84499	5.18E-23	4.16E-19	1.41E-18	1.10E-21	6.75E-18	88	305	506	227	472
CD93	-3.87493	-3.89331	-4.20279	-3.94578	-3.6179	5.44E-23	2.56E-20	2.96E-27	2.36E-24	4.22E-18	89	216	29	96	442
ITGAX	-2.42799	-2.54174	-2.65822	-2.78027	-2.77979	5.59E-23	4.65E-23	8.85E-21	8.63E-21	5.92E-22	90	85	319	285	132
KRT8	4.85547	4.752327	4.932598	4.754927	5.087468	6.00E-23	3.13E-23	3.44E-25	4.40E-23	5.72E-26	91	80	81	148	34
THY1	-5.78583	-5.99897	-5.1843	-6.00554	-5.76761	6.27E-23	2.64E-27	3.67E-25	6.90E-29	4.61E-25	92	17	82	23	59
AIF1	-2.85528	-2.88484	-3.13522	-2.99408	-2.95058	6.78E-23	8.82E-23	6.69E-24	1.02E-23	4.48E-21	93	95	121	125	185
LPXN	-2.1838	-1.86563	-2.26841	-2.19578	-2.01718	7.24E-23	3.62E-17	1.19E-19	5.77E-23	1.64E-13	94	510	399	159	1277
ITGAL	-1.92528	-1.61288	-2.06764	-1.80858	-1.9402	7.24E-23	4.06E-20	4.81E-21	2.23E-18	8.78E-19	95	228	298	492	372
MDH2	2.416366	2.160814	2.403552	2.291432	2.698434	7.24E-23	1.68E-24	6.22E-25	4.82E-21	1.80E-21	96	42	87	271	163
MCAM	-5.00398	-5.09784	-5.21231	-5.18807	-4.94291	7.24E-23	3.31E-24	7.41E-25	1.09E-27	6.45E-22	97	50	93	35	136
CLEC14A	-2.85519	-2.84115	-2.77697	-2.97581	-2.6611	7.24E-23	5.10E-21	3.24E-24	3.79E-24	3.58E-23	98	168	110	102	102
CLDN3	3.918115	3.907033	3.976682	3.837061	4.043692	8.64E-23	2.19E-22	4.18E-24	7.81E-23	6.39E-26	99	113	113	165	37
COL1A1	-6.57591	-6.90477	-5.96534	-7.00207	-6.8914	9.24E-23	3.36E-30	5.25E-24	5.79E-30	9.66E-27	100	6	117	11	22
LBH	-3.45859	-3.33064	-3.43762	-3.64064	-3.25146	9.94E-23	2.62E-21	9.46E-23	9.61E-27	7.51E-21	101	154	172	50	201
EMILIN1	-4.63446	-4.41838	-4.16804	-4.57315	-4.79059	1.06E-22	2.11E-21	4.47E-26	1.12E-24	1.19E-23	102	149	52	89	93
LCP2	-3.61871	-3.63074	-3.63168	-3.74865	-3.82809	1.08E-22	9.78E-21	4.80E-26	2.18E-22	8.84E-22	103	188	54	187	150
CAVIN1	-3.88068	-3.78285	-3.6337	-3.81666	-3.89561	1.24E-22	1.43E-20	1.86E-21	7.37E-24	5.40E-21	104	205	254	115	190
IGFBP3	-4.5632	-4.28964	-4.09319	-4.62325	-4.23821	1.37E-22	2.17E-22	3.03E-22	1.67E-26	2.49E-22	105	112	204	54	115
QKI	-3.38109	-3.34866	-3.46591	-3.35363	-3.37976	1.39E-22	6.82E-23	2.21E-28	6.85E-24	1.16E-23	106	90	17	113	92
LUM	-6.32266	-6.22344	-5.61675	-6.60311	-6.33694	1.40E-22	1.43E-25	6.27E-25	7.94E-29	7.91E-27	107	28	88	24	20
VASH1	-2.96871	-2.68593	-2.67698	-2.8298	-2.70676	1.81E-22	2.54E-25	2.42E-24	3.77E-27	3.73E-21	108	31	104	42	181
CRISPLD2	-3.44546	-3.5852	-3.86327	-3.82042	-3.64575	1.90E-22	1.25E-20	1.24E-26	3.99E-21	1.02E-24	109	200	41	266	67
EHF	3.080074	3.221037	3.257426	3.495017	3.292214	2.00E-22	1.20E-23	1.06E-22	1.81E-24	6.77E-20	110	66	174	95	276
CD3D	-2.03274	-1.83378	-2.03247	-2.06373	-2.23499	2.45E-22	1.71E-17	1.22E-21	1.47E-19	1.58E-23	111	460	244	372	96
FCGR3A	-2.86085	-3.27437	-3.2935	-3.39619	-3.28036	2.53E-22	8.93E-22	2.53E-21	4.73E-23	2.92E-20	112	135	264	151	243
ATP1A1	3.024386	2.587734	3.068258	2.92729	3.120197	2.72E-22	8.21E-21	7.36E-26	2.08E-21	1.94E-22	113	182	60	247	111
GALNT3	2.765506	2.932405	2.828908	3.10685	2.85897	2.73E-22	3.44E-21	6.82E-23	4.49E-22	7.80E-27	114	161	168	202	19
ITGB4	2.685796	2.437885	2.938602	2.685223	2.395148	2.81E-22	1.79E-17	3.92E-21	4.28E-17	1.45E-15	115	465	279	653	835
IL10RA	-2.54788	-2.07415	-2.96793	-2.45461	-2.8202	3.02E-22	5.71E-18	9.72E-22	3.41E-15	7.40E-16	116	407	237	940	779
LAD1	3.280931	3.176047	3.487581	3.193899	3.297255	3.02E-22	6.82E-23	3.95E-29	1.69E-26	1.03E-25	117	91	11	55	41
CBX6	-2.74673	-2.36746	-2.77867	-2.65842	-2.62761	3.04E-22	3.41E-19	1.24E-22	1.67E-22	2.45E-21	118	297	180	181	173
TRAC	-2.83872	-2.47758	-2.96443	-2.84594	-3.02289	3.06E-22	1.60E-18	2.98E-21	3.67E-23	9.08E-17	119	348	272	146	633
HDAC7	-1.57871	-1.69784	-1.69983	-1.64416	-1.38372	3.65E-22	2.00E-22	7.60E-24	2.03E-20	3.61E-13	120	109	125	307	1356
ARHGDIB	-3.43756	-3.51537	-3.74043	-3.62369	-3.50863	3.71E-22	1.70E-17	5.79E-22	1.32E-20	5.61E-19	121	457	221	297	352
SPON2	-5.06272	-5.10952	-4.84052	-5.33366	-5.40476	3.72E-22	4.82E-24	3.93E-21	7.29E-28	2.31E-25	122	57	283	32	50
MSN	-4.60563	-4.71972	-4.37174	-4.8517	-4.51794	3.74E-22	1.68E-24	5.15E-24	8.69E-29	7.35E-22	123	44	115	25	139
ENTPD1	-3.3431	-3.27548	-3.56209	-3.26095	-3.04626	3.74E-22	9.78E-21	2.96E-27	1.25E-21	3.57E-18	124	187	28	230	434
PXDN	-4.29915	-4.44534	-4.27586	-4.38202	-4.0215	4.98E-22	2.74E-21	5.71E-24	1.93E-23	6.48E-18	125	156	118	135	468
DTX3	-1.74868	-1.495	-1.57973	-1.61526	-1.63709	5.01E-22	3.27E-18	9.25E-23	9.19E-19	6.38E-21	126	381	171	455	197
VIL1	3.583709	3.720718	3.764596	3.736893	3.777659	5.01E-22	4.90E-20	4.95E-26	1.84E-19	5.02E-28	127	240	57	384	12
COL6A2	-6.20957	-6.68036	-5.72847	-6.81884	-6.77022	5.50E-22	1.86E-27	8.67E-23	1.58E-29	9.61E-26	128	16	170	15	39
C1R	-5.20106	-5.13492	-4.79861	-5.15059	-5.21238	5.60E-22	1.21E-21	1.59E-24	1.29E-23	6.09E-24	129	139	97	130	84
CAV1	-4.64797	-4.30026	-4.33453	-4.65495	-4.37074	5.80E-22	7.88E-20	1.06E-21	4.98E-22	4.73E-20	130	254	238	208	263
SRPK1	2.138206	2.142181	1.89527	2.094288	2.082566	5.81E-22	1.01E-22	2.51E-22	7.79E-23	8.69E-23	131	98	195	164	107
TGFB1I1	-3.08958	-3.01784	-2.99285	-3.08939	-2.7952	5.89E-22	8.32E-25	3.11E-22	4.73E-23	6.70E-20	132	37	205	153	274
ARHGAP9	-2.44671	-2.33571	-2.80419	-2.4989	-2.58906	5.94E-22	5.37E-19	2.06E-24	4.70E-19	2.13E-18	133	316	100	430	407
JUP	2.327389	2.178514	2.373115	2.645873	2.30486	5.99E-22	8.14E-17	1.35E-18	3.26E-21	8.22E-18	134	557	503	256	485
DSG2	3.292647	2.665059	3.234352	2.888713	3.063001	6.14E-22	5.15E-18	7.60E-23	3.54E-22	1.88E-22	135	402	169	198	110
NRP1	-3.15348	-3.15084	-3.16549	-3.23619	-3.33692	6.21E-22	1.31E-22	3.49E-22	3.90E-24	2.27E-23	136	106	211	104	100
IFFO1	-2.65768	-2.71177	-2.85656	-2.81066	-2.83489	6.21E-22	2.20E-21	2.31E-24	4.60E-24	2.00E-25	137	151	103	106	46
MPEG1	-3.36414	-3.35178	-3.57835	-3.46059	-3.25621	6.74E-22	5.82E-20	2.83E-25	2.01E-22	2.81E-17	138	244	78	185	556
ITGA4	-3.28433	-3.26401	-3.55155	-3.71796	-3.58811	6.74E-22	3.89E-22	1.72E-20	1.51E-25	2.11E-20	139	122	341	72	232
DDR1	3.39235	3.190106	3.336904	3.303335	3.281476	7.05E-22	3.00E-22	5.39E-31	6.51E-24	3.50E-25	140	119	3	112	53
GMDS	2.455389	2.318923	2.528253	2.69496	2.751282	7.55E-22	4.51E-20	4.80E-21	6.09E-23	1.02E-21	141	234	297	161	156
MAF	-3.35073	-3.307	-3.46021	-3.61745	-3.52912	8.11E-22	4.63E-21	4.73E-18	9.31E-23	6.68E-21	142	166	583	169	199
CGN	2.88894	2.697771	2.973678	2.60057	2.35956	8.31E-22	7.01E-19	7.44E-24	2.34E-22	6.40E-16	143	326	124	190	769
EPS8L2	3.306337	3.299503	3.229058	3.289191	2.914064	8.43E-22	1.01E-25	2.85E-24	1.38E-28	1.04E-18	144	27	108	26	380
MXRA8	-4.20205	-4.02975	-4.00807	-4.04074	-4.46757	8.54E-22	8.91E-20	3.65E-23	5.65E-22	8.16E-25	145	261	157	211	65

VCAN	-4.73406	-4.81102	-4.27493	-4.75546	-4.66258	8.61E-22	9.61E-22	3.38E-22	1.82E-25	1.25E-21	146	136	208	73	161
ARHGAP2!	-2.28042	-2.01915	-2.37086	-2.3248	-1.99743	8.70E-22	4.41E-19	3.86E-21	3.61E-20	1.03E-14	147	309	277	322	986
DOCK2	-2.4646	-2.24212	-2.85113	-2.41989	-2.63018	8.70E-22	6.78E-21	2.25E-24	3.76E-19	1.88E-16	148	174	102	416	683
CCDC88A	-2.05344	-1.94025	-2.12713	-2.14071	-2.05158	8.77E-22	5.81E-21	1.07E-19	5.08E-20	1.02E-19	149	172	393	330	294
GNB4	-2.7934	-3.06888	-3.00277	-3.1195	-2.68366	1.05E-21	2.08E-19	5.13E-21	3.83E-20	2.51E-20	150	283	307	324	238
CSRP2	-2.93205	-2.9402	-2.90795	-3.24343	-2.94913	1.14E-21	1.11E-22	2.00E-23	2.99E-25	3.46E-22	151	102	141	77	121
NCKAP1L	-2.37655	-1.97745	-2.65408	-2.27407	-2.32968	1.16E-21	5.01E-16	6.63E-21	2.23E-18	1.76E-14	152	655	314	494	1041
DSP	2.902091	3.000826	3.357752	2.948678	3.150662	1.18E-21	3.42E-19	5.60E-22	3.03E-20	7.34E-24	153	300	219	316	88
IKZF1	-2.25692	-1.89023	-2.46365	-2.31308	-2.58493	1.18E-21	1.33E-15	5.85E-15	2.19E-14	2.60E-16	154	715	1080	1105	714
FERMT1	2.778085	2.869888	2.942434	3.08762	3.072494	1.21E-21	1.44E-20	2.48E-22	2.94E-24	6.29E-25	155	207	194	99	63
FYN	-2.06318	-1.84844	-2.10119	-2.00797	-1.74541	1.38E-21	3.93E-17	1.65E-22	3.24E-17	6.92E-14	156	514	183	637	1188
FPR3	-2.46857	-2.35015	-2.47362	-2.5803	-2.33505	1.48E-21	3.17E-20	3.93E-21	8.03E-21	3.39E-15	157	221	281	283	882
SPOCK2	-1.71147	-1.6609	-2.04593	-1.87016	-2.23229	1.50E-21	1.21E-21	8.62E-18	5.58E-19	4.95E-18	158	138	618	434	452
FMNL3	-3.2187	-3.02997	-2.87202	-3.16028	-2.75993	1.52E-21	1.11E-22	1.86E-23	1.87E-23	2.53E-20	159	103	138	134	240
ITGA5	-4.35572	-4.32852	-4.48668	-4.52118	-4.66583	1.52E-21	8.16E-22	7.10E-24	5.51E-24	8.41E-25	160	133	122	109	66
EIF6	2.857388	2.8492	2.83058	2.893344	2.749786	1.53E-21	1.93E-20	9.92E-24	1.71E-19	8.29E-21	161	212	130	380	207
PDGFRA	-4.60405	-4.32477	-4.07907	-4.70461	-4.08535	1.53E-21	1.19E-20	1.04E-22	4.78E-25	3.73E-19	162	197	173	82	331
SLA	-1.91958	-1.78251	-2.07036	-2.04317	-2.10393	1.54E-21	6.79E-17	1.53E-18	2.14E-19	3.67E-17	163	545	510	389	570
LHFPL6	-3.25911	-3.14019	-3.13772	-3.30844	-3.00849	1.59E-21	1.99E-23	3.44E-23	5.84E-26	9.77E-22	164	72	153	64	155
TUBB6	-3.15883	-3.00467	-3.3511	-2.99088	-3.32357	1.71E-21	8.69E-17	1.91E-23	4.88E-21	1.22E-21	165	560	139	272	159
TAGLN	-4.81339	-4.87697	-4.56446	-5.19403	-4.95637	1.87E-21	4.42E-22	1.18E-22	3.28E-26	6.25E-25	166	125	178	58	62
SPINT2	3.261125	2.965361	3.563613	2.971702	3.146261	1.93E-21	4.13E-18	2.38E-21	2.98E-19	1.62E-20	167	394	263	400	224
NID2	-4.16717	-4.00749	-3.95503	-3.9443	-3.84701	2.05E-21	1.28E-21	1.72E-23	1.37E-25	2.33E-20	168	140	134	71	234
TNC	-5.05397	-4.96487	-5.16227	-5.26514	-6.1077	2.08E-21	4.54E-17	3.08E-18	4.88E-20	5.78E-24	169	521	549	329	83
VWF	-3.76898	-3.69647	-4.09866	-3.68349	-3.49197	2.08E-21	1.87E-18	1.45E-23	8.50E-23	1.55E-15	170	356	133	166	839
SERPINF1	-3.47419	-3.27682	-3.43908	-3.53773	-3.60465	2.13E-21	3.20E-20	1.47E-21	3.65E-24	4.87E-24	171	223	250	101	80
FERMT3	-2.04859	-1.99544	-2.11624	-2.22551	-2.26594	2.15E-21	3.36E-19	8.17E-22	9.07E-21	1.57E-20	172	291	228	288	223
ST14	3.729698	3.424643	4.013392	3.661104	3.406918	2.22E-21	1.72E-19	3.49E-23	3.06E-21	8.32E-20	173	272	154	254	285
ESAM	-3.33756	-3.43627	-3.45718	-3.45334	-3.1788	2.24E-21	3.04E-20	2.60E-23	1.10E-23	9.56E-19	174	218	145	128	378
ANTXR1	-4.93804	-4.89984	-4.89317	-5.23085	-5.43394	2.26E-21	2.67E-21	6.21E-25	7.97E-24	3.51E-25	175	155	86	119	54
STARD10	2.329603	2.461592	2.316534	2.261103	1.553811	2.35E-21	2.07E-22	9.74E-21	4.53E-17	5.32E-09	176	111	322	655	2695
ADAMDEC	-3.31973	-2.96586	-3.37277	-3.35356	-3.84952	2.36E-21	1.37E-19	8.67E-19	5.14E-20	4.01E-21	177	268	479	331	182
COL5A1	-5.94773	-5.92298	-5.00248	-6.11736	-5.75449	2.37E-21	5.92E-27	2.01E-22	4.05E-29	1.25E-24	178	21	187	19	68
CD52	-2.17906	-1.92617	-2.14216	-2.00302	-1.92418	2.69E-21	9.35E-20	1.07E-20	7.80E-20	7.41E-14	179	262	328	342	1193
MAN1C1	-1.83398	-1.3604	-1.70291	-1.62962	-1.67489	2.69E-21	3.65E-14	1.09E-16	8.50E-17	6.87E-20	180	944	775	691	277
EHD2	-3.10912	-2.8676	-2.82381	-3.09073	-3.24037	2.76E-21	1.89E-23	4.84E-25	1.97E-27	1.06E-25	181	70	84	39	42
PDGFRB	-4.92436	-5.04611	-4.27346	-5.08111	-5.03635	2.76E-21	1.68E-24	5.18E-22	7.95E-27	2.07E-22	182	40	216	48	113
C1QA	-4.34941	-4.92438	-4.4848	-4.93198	-4.72234	3.08E-21	2.61E-23	2.23E-21	2.81E-23	4.91E-22	183	75	262	142	128
RFTN1	-3.89528	-3.82431	-3.76699	-4.01964	-3.68535	3.09E-21	5.83E-22	8.11E-24	4.57E-26	3.76E-22	184	131	126	60	123
SH2D2A	-2.03523	-1.94143	-2.10283	-2.18705	-2.19946	3.59E-21	2.62E-14	1.55E-17	9.80E-17	1.72E-16	185	917	656	697	680
LIMD2	-2.79106	-2.41754	-2.92226	-2.53973	-2.99433	3.64E-21	1.84E-18	2.84E-23	1.21E-19	5.27E-19	186	355	149	361	346
WIPF1	-3.81951	-3.71628	-4.06793	-3.91509	-3.98722	4.01E-21	7.78E-23	1.68E-28	6.12E-23	1.81E-23	187	93	14	162	97
LCK	-1.66284	-1.52934	-1.59908	-1.75221	-1.53194	4.05E-21	3.83E-17	5.91E-20	8.80E-21	2.71E-14	188	512	382	287	1085
RASAL3	-2.26575	-1.67134	-2.4179	-1.96478	-2.38536	4.15E-21	6.85E-15	4.65E-17	3.19E-15	5.96E-18	189	811	707	931	464
RFLNB	-2.87059	-2.53392	-2.73701	-2.71811	-2.60883	4.17E-21	1.81E-19	2.98E-22	3.88E-24	4.99E-16	190	276	202	103	754
IGHA1	-7.41276	-6.42925	-6.29698	-6.96546	-5.58715	4.37E-21	3.73E-14	3.24E-13	4.79E-17	1.11E-11	191	946	1469	660	1783
CD34	-3.3431	-3.34709	-3.44335	-3.56135	-3.00917	4.48E-21	4.58E-20	2.45E-19	3.26E-22	3.46E-15	192	238	431	195	887
COL18A1	-4.2908	-4.21232	-3.87203	-4.35635	-4.08433	5.03E-21	5.01E-26	9.07E-25	1.97E-27	1.84E-24	193	25	95	38	73
FRMD6	-2.54153	-2.27518	-2.55701	-2.25127	-2.18777	5.04E-21	3.12E-17	6.09E-19	1.15E-14	1.56E-14	194	496	468	1049	1026
CALD1	-5.75421	-5.73488	-5.15174	-6.0364	-5.81418	5.04E-21	2.70E-25	3.65E-23	2.00E-29	6.25E-25	195	32	156	16	61
FAM20C	-4.41364	-4.29804	-4.04375	-4.71875	-4.14747	5.08E-21	2.24E-20	5.05E-23	3.28E-21	4.24E-19	196	213	161	259	341
THBD	-2.43949	-2.04029	-2.43992	-2.24545	-2.55178	5.13E-21	1.12E-20	1.35E-20	7.41E-22	8.76E-22	197	192	333	218	148
HIC1	-3.53945	-3.14783	-3.18079	-3.48106	-2.88536	5.28E-21	1.38E-21	1.91E-20	1.26E-22	1.53E-16	198	141	346	174	674
TMEM54	3.165273	3.063807	3.500936	3.16222	2.728523	5.47E-21	2.92E-21	2.30E-22	3.78E-25	3.52E-18	199	157	192	81	432
PERP	3.875992	3.630875	3.968483	3.761777	3.845277	5.53E-21	2.07E-18	3.65E-20	5.79E-18	2.84E-21	200	361	367	544	177
SDC2	-4.60187	-4.54217	-4.0653	-4.51113	-4.29714	5.56E-21	3.35E-22	2.84E-23	1.11E-25	2.45E-20	201	120	148	70	237
CLIC4	-3.01186	-2.84196	-3.11117	-3.13836	-2.96499	5.73E-21	3.02E-18	5.18E-22	3.15E-19	6.14E-22	202	380	217	401	133
TYROBP	-3.68772	-3.7054	-3.72298	-3.88674	-3.60907	7.05E-21	9.93E-21	1.23E-22	5.51E-23	9.17E-20	203	189	179	158	289
ZEB2	-3.49296	-3.5813	-3.59054	-3.59464	-3.7866	7.28E-21	4.04E-23	1.10E-25	3.57E-25	5.73E-25	204	83	67	79	60
GPRC5A	3.902051	3.699755	4.208987	4.059001	3.742515	7.47E-21	3.51E-17	1.96E-21	6.55E-22	1.91E-23	205	507	256	213	98
USH1C	3.171574	2.883286	3.363195	3.013064	2.876855	7.85E-21	3.87E-18	2.72E-22	1.68E-19	5.81E-19	206	389	199	379	354
TIMP2	-4.73482	-4.41821	-4.24842	-4.67966	-4.93588	8.10E-21	1.74E-20	1.86E-23	6.74E-25	8.19E-24	207	209	137	85	91
CXCL14	-4.97467	-4.14052	-5.21606	-4.56273	-5.02422	8.62E-21	3.69E-12	4.22E-22	2.22E-15	4.24E-19	208	1394	213	911	340
ZEB1	-2.44897	-2.33809	-2.53716	-2.5102	-2.57006	8.62E-21	4.05E-20	7.17E-24	4.51E-22	2.44E-21	209	227	123	205	172
CTLA4	-2.13819	-2.07704	-2.2588	-2.3432	-1.85149	8.70E-21	1.88E-19	1.26E-21	2.13E-22	2.82E-08	210	279	245	186	3013
SLAMF8	-2.20793	-2.32604	-2.43568	-2.30886	-2.44539	9.16E-21	1.07E-21	4.97E-21	1.19E-20	2.88E-20	211	137	302	294	242
LILRB3	-2.70872	-2.53912	-2.97009	-2.73741	-2.69196	9.16E-21	4.06E-18	2.16E-19	1.82E-19	8.19E-16	212	392	424	383	792
CSF1R	-2.67481	-2.55221	-2.83094	-2.67607	-2.96974	1.14E-20	7.02E-20	1.25E-19	2.03E-21	9.34E-21	213	250	401	246	212
MYO6	1.729239	1.729018	1.824211	1.906455	1.858188	1.24E-20	1.89E-17	1.14E-19	3.50E-18	1.31E-19	214	468	396	509	302
NXN	-2.55218	-2.04575	-2.16355	-2.5361	-2.25763	1.30E-20	1.84E-10	2.50E-10	7.30E-19	1.26E-11	215	1911	2357	442	1795
COL12A1	-5.56746	-5.39169	-4.93237	-5.64641	-5.77096	1.52E-20	3.21E-19	4.12E-21	2.75E-23	4.34E-22	216	290	287	141	126
ANKRD44	-1.9009	-1.6752	-2.22219	-1.89279	-1.80282	1.63E-20	2.36E-17	3.74E-20	1.90E-15	2.17E-13	217	478	368	895	1307
NID1	-4.25031	-4.4307	-4.43856	-4.42666	-4.09423	1.67E-20	6.65E-24	1.41E-23	1.54E-22	3.73E-19	218	60	132	178	332

SCARF1	-2.27032	-2.03142	-2.18165	-2.2932	-1.92463	1.68E-20	3.87E-18	2.96E-17	4.73E-23	2.11E-14	219	388	686	152	1060
FYB1	-2.44877	-2.49111	-2.59898	-2.81714	-2.48254	1.75E-20	2.81E-22	5.86E-20	2.08E-23	2.07E-19	220	118	381	136	317
TBC1D9	-2.09835	-1.95672	-2.47949	-2.14227	-2.56189	1.78E-20	1.02E-17	2.10E-18	2.88E-17	1.24E-19	221	434	530	624	300
PRR15L	2.726472	2.668435	3.001052	2.85666	2.721188	1.83E-20	3.35E-22	2.50E-24	4.69E-26	1.74E-24	222	121	105	61	71
GPNMB	-3.80547	-4.19573	-3.83525	-4.15132	-4.25856	1.90E-20	1.13E-23	1.33E-25	6.43E-24	1.08E-26	223	63	72	111	23
BAIAP2L1	2.713952	2.434164	3.102295	2.586574	2.987844	1.96E-20	1.13E-17	2.72E-24	1.44E-21	2.86E-24	224	439	106	235	76
STOM	-3.11691	-2.95695	-2.94421	-3.19812	-2.7123	2.25E-20	1.27E-19	9.61E-24	3.61E-24	5.98E-19	225	267	129	100	356
JAM3	-2.43486	-2.03002	-2.16881	-2.21596	-2.04677	2.25E-20	9.44E-15	4.88E-19	4.79E-17	4.81E-19	226	828	462	658	344
PLPP2	2.619627	2.500551	2.658724	2.838962	2.400513	2.34E-20	5.11E-19	1.15E-22	1.40E-21	3.64E-14	227	314	176	234	1121
SYDE1	-3.03263	-2.92375	-2.79781	-3.05916	-2.33439	2.36E-20	1.72E-19	1.28E-22	4.50E-21	1.56E-13	228	274	182	269	1270
ADAP1	2.692046	2.567283	2.556705	2.390912	2.509127	2.40E-20	5.56E-21	2.34E-20	1.48E-19	4.38E-21	229	169	351	373	184
RGL1	-2.0812	-1.81204	-2.03398	-1.89598	-1.73036	2.41E-20	6.31E-17	2.77E-21	1.22E-18	2.80E-11	230	537	269	464	1888
BIN2	-1.87166	-1.63878	-2.02969	-1.77055	-2.2037	2.53E-20	2.63E-16	2.81E-18	4.63E-16	2.06E-17	231	614	544	796	539
PRRT2	-1.37257	-1.00077	-1.25277	-1.19582	-1.37161	2.53E-20	3.14E-09	1.30E-13	4.42E-10	3.44E-13	232	2355	1355	2265	1352
IGFBP5	-5.06228	-4.53256	-4.65573	-4.4452	-5.12851	2.81E-20	6.67E-17	5.97E-23	1.42E-19	7.92E-21	233	540	165	370	205
CORO1A	-2.87849	-2.97723	-3.35039	-3.257	-3.03702	2.83E-20	4.25E-20	1.21E-21	1.19E-21	8.53E-13	234	232	243	228	1463
COX5B	2.56188	2.359386	2.695847	2.51664	2.618383	2.95E-20	7.18E-20	3.54E-22	1.41E-19	1.61E-24	235	251	212	369	69
MFGE8	-4.48652	-4.17639	-4.31173	-4.40799	-4.64542	3.00E-20	7.97E-23	5.25E-24	9.42E-26	5.00E-24	236	94	116	68	82
OAZ2	-2.03606	-1.78778	-1.81527	-1.75691	-1.59706	3.33E-20	9.68E-17	4.26E-21	9.33E-16	5.05E-13	237	572	290	841	1396
HLA-DMB	-2.179	-2.10126	-1.91273	-1.91025	-1.93429	3.36E-20	4.86E-18	6.93E-15	9.72E-16	4.21E-14	238	399	1092	843	1141
VCAM1	-2.1369	-1.6654	-2.05972	-2.06993	-2.09106	3.53E-20	1.94E-16	3.21E-17	1.47E-18	5.17E-17	239	606	689	475	593
GBP5	-1.95483	-2.06743	-2.31806	-2.37026	-2.23817	3.56E-20	6.91E-17	9.56E-18	3.57E-17	3.36E-20	240	546	624	646	248
HEG1	-3.19183	-3.2797	-3.15118	-3.25659	-2.91721	3.66E-20	4.12E-22	3.93E-21	4.08E-22	6.04E-17	241	124	282	199	604
MRC2	-3.68913	-3.38015	-3.40334	-3.52231	-3.97352	3.66E-20	6.41E-20	8.62E-20	1.87E-20	1.25E-21	242	246	386	304	160
CD53	-2.70013	-2.31181	-2.65993	-2.52402	-2.66925	3.74E-20	3.69E-17	9.69E-19	1.74E-18	1.03E-17	243	511	490	482	502
IL6ST	-2.62685	-2.54204	-2.27666	-2.62094	-2.75352	3.74E-20	1.90E-24	3.10E-21	3.18E-23	1.05E-19	244	48	274	143	297
GGT5	-3.77274	-3.60889	-3.65662	-3.8108	-3.91699	3.74E-20	5.66E-18	4.12E-21	2.13E-21	2.07E-22	245	406	286	249	114
CD4	-2.75572	-2.73767	-2.82563	-2.85935	-2.75417	3.90E-20	1.72E-19	4.47E-20	1.73E-20	5.35E-17	246	273	369	303	596
LAMA4	-4.41028	-4.62517	-4.74756	-4.57878	-4.308	3.94E-20	1.59E-18	6.16E-22	2.17E-20	1.83E-18	247	346	223	308	402
TOB1	2.576101	2.134655	2.690035	2.300492	2.318931	4.15E-20	4.73E-16	1.34E-19	3.07E-17	6.34E-16	248	646	407	627	768
FSTL1	-5.14999	-5.01715	-4.53366	-5.13447	-4.69839	4.38E-20	5.78E-22	1.02E-20	5.12E-24	5.07E-20	249	130	323	107	264
PLCB2	-2.14624	-1.85679	-2.05792	-2.22067	-1.7911	4.40E-20	4.03E-17	8.34E-16	4.40E-19	1.33E-15	250	515	921	423	826
MUC13	4.104633	3.831323	4.244121	4.21144	4.003587	4.73E-20	2.68E-19	2.77E-21	2.20E-22	4.57E-21	251	284	270	188	188
SELPLG	-2.20954	-1.96209	-2.20006	-2.23371	-2.21552	4.76E-20	1.63E-11	2.25E-16	1.86E-11	5.56E-15	252	1586	826	1855	928
KDR	-3.22622	-3.15299	-3.35602	-3.17276	-2.7593	5.07E-20	9.95E-18	4.68E-22	1.98E-20	5.86E-14	253	432	215	306	1172
ZNF532	-2.36841	-2.35977	-2.47043	-2.59034	-2.31156	5.07E-20	4.48E-19	5.16E-23	2.94E-21	5.11E-22	254	311	162	252	129
LST1	-2.49232	-2.58994	-2.91942	-2.6397	-2.61941	5.58E-20	3.52E-21	2.96E-24	6.46E-21	2.26E-16	255	162	109	276	702
CCN2	-4.64002	-4.37399	-4.30073	-4.51479	-4.53545	5.81E-20	3.95E-22	4.51E-21	4.14E-27	4.49E-21	256	123	294	43	186
SAMSN1	-2.73719	-2.62462	-2.92239	-2.82198	-2.96764	5.94E-20	1.29E-17	1.13E-22	3.54E-18	3.22E-20	257	448	175	510	247
CCDC102B	-2.60916	-2.73775	-2.68735	-2.59443	-2.53411	5.94E-20	5.37E-17	3.56E-18	5.92E-18	8.91E-18	258	529	559	548	489
LDB2	-3.02983	-2.90918	-2.95553	-3.14354	-2.79802	6.00E-20	7.47E-21	1.15E-21	1.29E-23	1.01E-19	259	175	241	129	293
AXL	-3.34372	-3.41251	-3.52344	-3.6337	-3.70997	6.45E-20	4.58E-20	5.85E-22	6.26E-21	1.40E-18	260	237	222	275	395
ARL4C	-2.26743	-2.03751	-2.29296	-2.14041	-2.17187	6.72E-20	1.25E-18	1.86E-22	1.34E-18	9.61E-20	261	342	185	468	290
LAYN	-1.4519	-1.27257	-1.61901	-1.30859	-1.193	6.73E-20	1.27E-11	1.52E-18	2.19E-11	1.44E-10	262	1554	509	1877	2106
PLEKHO2	-2.32238	-2.35138	-2.67557	-2.42981	-2.44376	6.92E-20	1.59E-18	1.70E-20	3.42E-19	1.40E-18	263	347	340	403	394
ACAN	-2.62925	-3.00992	-3.16994	-3.40652	-3.10014	7.19E-20	1.28E-15	5.02E-18	3.41E-21	9.49E-18	264	712	588	262	496
FNBP1	-2.24932	-2.16133	-2.27537	-2.35507	-2.13155	7.43E-20	5.73E-15	3.80E-18	9.66E-17	2.79E-19	265	797	572	695	325
AFDN	1.480965	1.505528	1.619611	1.670537	1.543085	7.46E-20	1.11E-20	8.51E-19	2.45E-20	1.49E-15	266	191	476	312	837
RNF43	3.802896	4.016264	3.4793	4.048455	3.640672	7.84E-20	3.13E-24	4.97E-21	2.66E-25	8.52E-22	267	49	303	76	146
CCN1	-4.30323	-4.09415	-4.2794	-4.31212	-4.40414	8.14E-20	8.85E-17	1.19E-19	4.18E-18	2.32E-18	268	562	398	519	414
CRIP2	-3.66009	-3.74617	-3.95782	-3.71565	-3.92788	8.26E-20	1.21E-17	5.88E-21	1.54E-22	8.49E-22	269	444	311	179	144
CALHM2	-1.66648	-1.52838	-1.80351	-1.74661	-1.67392	8.33E-20	1.25E-17	9.70E-18	8.49E-19	1.83E-17	270	446	627	451	530
CYBB	-2.78377	-2.99996	-2.92963	-3.15481	-3.09202	8.74E-20	7.88E-21	4.77E-20	1.46E-22	3.95E-20	271	177	372	176	251
MGP	-4.36923	-4.03915	-4.03527	-4.31835	-4.12517	8.81E-20	8.04E-21	3.68E-27	5.36E-23	1.25E-21	272	179	30	157	162
ADGRA2	-3.48956	-3.59994	-3.40425	-3.60401	-3.63532	8.92E-20	8.17E-21	9.47E-21	8.09E-24	2.89E-22	273	180	320	120	116
IL2RB	-2.1577	-2.06299	-2.22853	-2.43521	-2.04792	9.33E-20	3.22E-17	5.24E-18	1.07E-19	2.74E-14	274	500	592	353	1086
PRKCH	-2.2085	-2.19804	-2.11771	-2.28424	-2.04435	9.71E-20	2.73E-20	1.72E-19	4.49E-22	1.26E-18	275	217	414	204	391
TNS1	-3.42833	-3.38632	-3.32452	-3.45069	-3.48526	9.90E-20	2.09E-18	2.79E-23	4.02E-20	2.75E-21	276	362	147	326	176
C19orf48	1.98454	2.2374	1.918812	2.113129	1.894525	1.03E-19	5.13E-23	2.99E-20	1.05E-23	6.60E-20	277	87	359	127	271
CDH17	3.425135	3.230034	3.918999	3.38984	3.304847	1.08E-19	3.35E-21	7.13E-28	2.51E-21	2.41E-20	278	159	18	250	235
CMTM3	-3.16952	-3.001	-2.98922	-3.10513	-3.06393	1.12E-19	1.14E-23	2.72E-24	2.93E-24	7.29E-21	279	65	107	97	200
AP1M2	2.928633	2.92181	3.044775	2.83934	3.272693	1.13E-19	9.23E-21	4.04E-24	1.32E-21	3.56E-25	280	186	112	231	55
RAB25	2.724155	2.528195	2.850626	2.536664	2.829205	1.17E-19	5.96E-20	5.60E-22	6.04E-21	3.76E-23	281	245	220	274	104
POSTN	-4.94616	-5.35693	-4.59401	-5.23986	-5.29102	1.17E-19	4.33E-20	1.32E-19	1.53E-21	7.88E-20	282	233	406	240	281
FERMT2	-3.41955	-3.36279	-3.21162	-3.47055	-3.51642	1.24E-19	3.08E-19	2.81E-20	1.69E-22	7.92E-21	283	288	355	182	206
GIMAP8	-1.88439	-1.49223	-1.74809	-1.68154	-1.75807	1.24E-19	4.60E-17	5.09E-17	1.98E-16	5.14E-14	284	523	714	740	1157
ITGB2	-3.44773	-3.48567	-3.35148	-3.78921	-3.56254	1.24E-19	5.83E-21	6.40E-17	1.39E-22	1.77E-18	285	173	730	175	399
CHST15	-2.50735	-2.11328	-2.34979	-2.10645	-2.50514	1.38E-19	6.02E-19	3.38E-19	5.87E-16	9.42E-17	286	319	440	815	637
TP53INP1	-1.9616	-1.75736	-2.49614	-2.46348	-2.33023	1.50E-19	9.54E-17	1.55E-18	4.74E-15	1.38E-12	287	569	512	972	1525
FCER1G	-3.60873	-3.79362	-3.78341	-3.95676	-3.62894	1.71E-19	2.38E-20	9.26E-22	4.97E-22	1.87E-17	288	214	236	207	531
PLAU	-4.40067	-4.3451	-4.17885	-4.6494	-4.24919	1.79E-19	1.42E-21	4.39E-19	1.76E-21	6.23E-18	289	142	456	244	465
GIMAP7	-1.95477	-1.93085	-2.37191	-2.03235	-2.0477	1.88E-19	2.58E-17	2.07E-23	5.21E-18	1.38E-13	290	483	143	537	1256
MAP7	2.184323	2.499896	2.710077	2.449122	1.994883	1.92E-19	9.34E-17	3.31E-23	3.42E-16	2.84E-14	291	565	152	778	1094

SULF1	-4.92694	-5.05364	-4.62412	-5.09977	-4.99694	1.99E-19	5.80E-20	2.71E-22	3.09E-22	2.07E-22	292	243	198	194	112
LSM7	1.944524	2.029904	1.838188	2.08777	2.041448	2.01E-19	6.42E-19	2.02E-18	7.30E-23	7.79E-17	293	323	526	163	621
PODN	-2.564	-1.99715	-2.0852	-2.06844	-2.04996	2.04E-19	2.02E-14	3.46E-16	2.80E-14	8.81E-19	294	887	856	1121	373
WAS	-2.37257	-2.1478	-2.47739	-2.49967	-2.36415	2.15E-19	1.06E-16	9.75E-18	2.11E-19	3.71E-16	295	578	628	388	735
B3GNT3	3.219282	3.04857	3.376338	3.109202	3.001606	2.16E-19	3.21E-20	1.04E-20	2.71E-21	1.45E-16	296	224	324	251	667
ITIH5	-3.40339	-2.98557	-2.9386	-2.75229	-2.6345	2.17E-19	4.34E-14	3.65E-16	4.83E-15	2.44E-12	297	959	860	978	1585
GNAI2	-3.45659	-3.54815	-3.47425	-3.29207	-3.27271	2.18E-19	5.31E-22	8.19E-25	1.05E-20	4.21E-19	298	128	94	290	337
S100P	3.986151	3.701005	4.422887	3.790228	3.75447	2.22E-19	4.62E-22	1.07E-23	4.44E-22	2.18E-17	299	126	131	201	543
PRDM1	-3.34863	-3.16212	-3.54267	-3.20312	-3.24801	2.22E-19	2.44E-20	7.12E-25	3.89E-21	8.17E-17	300	215	92	265	626
RAB5IF	2.420485	2.141633	2.354663	2.181554	2.136659	2.26E-19	1.72E-16	2.08E-21	3.07E-17	7.67E-16	301	600	258	629	786
ARHGEF25	-2.26727	-2.14162	-2.52183	-2.30063	-2.35588	2.29E-19	1.76E-16	1.09E-20	8.42E-20	9.73E-20	302	602	329	343	291
COL16A1	-2.32104	-2.15987	-2.09321	-2.30468	-2.48538	2.33E-19	2.32E-18	3.79E-18	2.41E-20	1.18E-21	303	365	570	311	157
CHSY1	-1.96186	-2.03341	-2.06972	-1.93384	-1.86394	2.37E-19	3.42E-19	1.00E-18	3.11E-17	8.42E-15	304	298	491	630	968
EZR	2.588048	2.053191	2.627605	2.181788	2.244237	2.37E-19	3.60E-14	7.55E-18	7.54E-15	2.02E-16	305	941	609	1008	691
SLC12A2	3.425376	3.609349	3.088234	3.920046	3.636702	2.42E-19	2.15E-21	1.82E-18	1.59E-24	1.04E-16	306	150	518	92	648
TGFB1	-3.95731	-3.97594	-3.83292	-4.15669	-3.86308	2.43E-19	1.68E-24	5.89E-24	2.33E-28	1.21E-18	307	45	119	30	388
LRRC32	-3.51121	-3.30986	-3.10082	-3.06131	-3.20973	2.59E-19	5.66E-21	1.23E-20	4.43E-21	1.25E-18	308	171	331	268	389
RARRES2	-4.28262	-3.80658	-3.78307	-4.04874	-4.35817	2.73E-19	6.34E-19	3.16E-18	8.67E-19	1.23E-23	309	322	552	452	94
NLRP1	-2.29886	-1.86057	-2.52019	-2.37785	-2.1322	2.74E-19	2.66E-17	1.51E-18	7.56E-19	4.25E-15	310	485	508	444	908
SVEP1	-1.958	-1.65852	-1.78118	-1.71854	-1.74846	2.80E-19	4.56E-17	2.53E-21	8.74E-20	1.00E-17	311	522	265	344	500
DHCR24	2.880184	2.909365	3.261037	3.022253	2.883007	2.83E-19	1.75E-17	4.74E-23	1.45E-19	9.00E-22	312	461	160	371	151
DPYSL3	-3.62588	-3.41449	-3.25276	-3.38153	-3.29357	2.85E-19	2.52E-17	1.46E-20	3.45E-19	4.86E-18	313	481	334	405	449
FBLN1	-4.8926	-4.25655	-3.842	-4.39442	-4.28357	2.91E-19	3.52E-17	1.92E-17	1.23E-18	7.12E-19	314	508	664	465	361
TSPAN4	-2.62047	-2.63697	-2.73438	-2.73529	-2.62954	3.02E-19	2.07E-22	3.85E-21	1.14E-20	1.82E-19	315	110	276	291	315
EVA1B	-3.24389	-3.08396	-2.80663	-3.24062	-3.43707	3.23E-19	1.14E-23	8.25E-24	1.54E-27	7.86E-22	316	64	128	37	141
IRF2BP2	2.056147	2.185201	1.89916	2.259119	2.093593	3.43E-19	5.20E-18	5.02E-20	6.14E-18	4.31E-16	317	404	374	549	744
TMEM238	2.631016	2.819517	2.709209	2.935301	2.969867	3.43E-19	1.82E-20	1.65E-21	3.39E-22	6.45E-22	318	211	252	196	135
NR2F1	-2.17115	-2.01493	-2.27091	-2.1722	-2.242	3.45E-19	9.94E-15	3.06E-17	8.68E-19	6.60E-20	319	836	688	453	272
JAML	-2.07171	-1.8581	-2.41869	-1.97058	-2.11894	3.74E-19	2.58E-14	5.03E-18	4.39E-16	6.05E-14	320	916	589	793	1178
ROBO4	-2.5252	-2.34582	-2.77018	-2.19932	-2.25387	3.79E-19	2.50E-13	1.17E-17	1.95E-15	9.65E-12	321	1116	641	898	1757
SRGAP2B	-2.19104	-2.22871	-2.0679	-2.28802	-2.1865	3.79E-19	1.59E-23	4.13E-21	3.46E-20	2.59E-21	322	69	288	320	175
LAIR1	-2.31407	-2.39218	-2.62124	-2.69849	-2.17664	3.80E-19	1.05E-16	2.15E-17	6.48E-20	3.52E-12	323	577	672	335	1632
VAMP5	-1.73092	-1.52267	-1.61345	-1.65601	-1.5365	3.80E-19	5.81E-17	1.11E-19	5.50E-18	6.07E-17	324	533	394	541	606
PKP3	3.276695	3.312716	3.976163	3.324788	3.276669	3.89E-19	4.03E-21	9.58E-26	2.65E-22	5.00E-24	325	164	64	192	81
RGS1	-3.20975	-3.57213	-3.67689	-3.89868	-3.40992	3.89E-19	1.74E-18	6.09E-23	1.38E-18	5.30E-16	326	351	167	469	757
PSTPIP1	-1.83042	-1.60237	-1.86264	-1.75732	-2.06398	3.90E-19	2.14E-16	1.02E-19	1.67E-17	9.40E-23	327	611	392	597	108
CD2	-2.10454	-1.76106	-2.3644	-2.22635	-2.64991	3.97E-19	1.40E-15	6.64E-19	1.22E-16	7.62E-21	328	719	469	712	203
IL1R1	-2.87347	-2.71457	-2.8609	-2.78046	-2.77319	4.05E-19	2.84E-18	2.15E-22	8.80E-20	1.39E-16	329	377	191	346	666
SH2B3	-2.14292	-2.2031	-2.44863	-2.38707	-2.43695	4.07E-19	5.11E-15	1.42E-21	4.51E-22	1.49E-17	330	787	248	206	522
YWHAZ	1.639174	1.701192	1.894156	1.802355	1.871723	4.73E-19	1.98E-21	1.06E-20	4.31E-19	4.57E-18	331	148	327	421	445
HDHD3	2.602266	2.438825	2.719319	2.396781	2.427153	4.78E-19	3.31E-17	1.71E-22	2.47E-19	6.67E-20	332	503	184	393	273
ADAMTS1	-2.8472	-2.71717	-3.1392	-2.988	-2.81536	4.78E-19	5.11E-15	9.67E-21	2.55E-18	3.42E-16	333	786	321	503	729
CD248	-4.32329	-4.39919	-3.93382	-4.28899	-4.26085	4.90E-19	1.30E-22	1.77E-19	8.43E-26	2.27E-19	334	104	418	67	319
CCL2	-4.47334	-3.63334	-3.61726	-3.73148	-4.32112	4.96E-19	3.74E-14	1.56E-16	3.63E-15	1.91E-19	335	948	805	944	316
GPR35	2.312072	2.498249	2.492384	2.456024	2.725872	5.08E-19	6.56E-20	1.02E-19	1.07E-19	6.15E-21	336	248	391	354	194
ZNF703	2.380962	2.349693	2.340312	2.669997	2.479203	5.08E-19	8.23E-17	5.25E-18	5.41E-17	1.91E-17	337	558	593	670	532
NREP	-2.76856	-2.94675	-2.61737	-2.77395	-2.37338	5.08E-19	5.21E-18	1.78E-15	3.46E-20	2.12E-12	338	405	986	321	1562
PIP4K2A	-1.81083	-1.58277	-1.77806	-1.89695	-1.59371	5.12E-19	1.44E-17	2.02E-20	5.96E-19	1.73E-11	339	450	348	435	1834
MEF2A	-1.43893	-1.38538	-1.63027	-1.43614	-1.36521	5.13E-19	6.15E-18	4.37E-22	1.71E-17	1.51E-16	340	410	214	598	673
LTB	-2.3311	-1.98548	-2.55487	-2.18861	-2.07752	5.38E-19	4.12E-16	1.34E-21	1.85E-18	3.40E-12	341	638	246	486	1628
FCGR2C	-3.05791	-3.10939	-3.26913	-3.14726	-3.15216	5.55E-19	1.84E-18	8.60E-20	4.58E-20	7.57E-18	342	353	385	328	478
TAMALIN	-2.17843	-1.95965	-1.96423	-2.05388	-1.81972	5.80E-19	9.81E-18	8.42E-18	3.06E-20	6.00E-14	343	431	616	317	1176
ENTPD6	2.614211	2.685289	2.502999	2.850159	2.476751	5.84E-19	2.48E-21	9.08E-19	1.32E-21	7.86E-22	344	152	484	232	140
PHLDB1	-2.96122	-2.94402	-2.88867	-3.10271	-3.18612	6.28E-19	1.19E-17	2.40E-20	4.49E-22	2.70E-20	345	442	352	203	241
PDLIM3	-2.87525	-2.65806	-2.64255	-2.78511	-3.08627	6.48E-19	8.11E-17	5.77E-19	6.50E-20	9.40E-22	346	556	467	336	153
CAVIN3	-3.07579	-3.19427	-2.90932	-3.03172	-3.08814	6.52E-19	4.73E-20	1.31E-19	1.22E-21	8.29E-20	347	239	404	229	284
SPARCL1	-4.28894	-3.67425	-3.85582	-3.72378	-4.11674	6.58E-19	5.66E-16	1.86E-20	4.30E-18	1.41E-20	348	666	345	524	221
PKD2	-2.29517	-2.17335	-1.82949	-2.29033	-1.95271	6.74E-19	4.30E-18	1.34E-16	9.36E-20	1.18E-15	349	395	789	348	816
LGALS3	3.57954	2.950922	3.958639	3.056689	3.355266	6.83E-19	1.43E-20	1.17E-22	2.59E-19	5.61E-19	350	204	177	395	353
PLPP1	-3.26069	-3.24004	-3.12401	-3.35408	-3.34801	6.96E-19	4.05E-18	1.81E-21	1.57E-19	6.25E-20	351	391	253	375	269
CDX2	3.134165	3.718995	3.230337	3.860123	3.783582	6.97E-19	1.53E-25	4.39E-21	2.44E-23	7.61E-24	352	29	292	140	89
FBLN5	-2.0919	-1.89585	-2.47274	-2.29009	-2.21923	7.15E-19	2.16E-14	7.72E-17	2.10E-15	9.29E-18	353	894	751	902	493
PLEKHG2	-2.65549	-2.46671	-2.58071	-2.65597	-2.39951	7.29E-19	8.89E-21	1.15E-21	4.41E-23	6.80E-17	354	185	240	149	612
TRIM22	-2.39564	-2.2792	-2.61332	-2.44962	-2.2358	7.57E-19	7.13E-16	1.69E-20	8.63E-18	3.40E-15	355	676	339	567	884
IL17RA	-1.29731	-1.19621	-1.20849	-1.07864	-1.06686	7.61E-19	7.86E-15	1.46E-15	9.71E-15	3.93E-12	356	823	967	1029	1645
DRAM1	-2.20231	-2.17862	-2.23627	-2.1611	-2.03113	7.65E-19	1.15E-17	4.68E-21	2.04E-18	3.73E-13	357	441	295	490	1364
PKP2	2.641792	2.528085	2.464799	2.408466	2.314384	7.65E-19	7.21E-18	5.47E-16	4.92E-17	1.91E-18	358	417	884	662	406
RUNX1T1	-2.1605	-1.79305	-1.68346	-1.98256	-1.76565	7.87E-19	7.82E-18	6.31E-18	1.17E-19	9.03E-19	359	418	601	357	376
CDH5	-2.95507	-2.99326	-3.04627	-3.01532	-2.61397	8.43E-19	3.39E-19	2.04E-23	5.18E-19	5.56E-14	360	294	142	432	1163
GLIPR1	-3.07592	-2.84124	-3.14136	-2.97676	-3.2505	8.43E-19	1.12E-17	3.34E-20	1.83E-18	8.62E-17	361	438	363	485	630
EIF3B	2.124836	2.058636	2.018952	2.075772	2.024818	8.51E-19	7.34E-20	1.05E-17	1.32E-20	3.98E-19	362	252	634	298	334
MYO1G	-1.92231	-1.70568	-1.93466	-1.88938	-1.72192	8.94E-19	1.39E-14	1.61E-18	9.94E-15	9.28E-15	363	860	513	1034	978
CSGALNA(	-2.5245	-2.4545	-2.71046	-2.63875	-2.63884	9.09E-19	8.77E-17	4.51E-27	1.88E-22	6.21E-22	364	561	31	183	134

FCGR1A	-1.50795	-1.40584	-1.87353	-1.50708	-1.7888	9.20E-19	2.30E-17	7.00E-19	1.91E-15	5.05E-17	365	477	470	896	591
POLI	-1.32449	-1.34554	-1.35664	-1.37039	-1.36682	9.27E-19	1.51E-18	8.59E-19	3.37E-17	9.70E-15	366	345	477	638	980
CDH11	-4.31135	-4.25726	-4.02661	-4.14385	-4.25505	9.34E-19	4.34E-21	2.67E-23	1.31E-19	8.49E-22	367	165	146	365	145
PLP2	2.144313	2.254672	1.858435	2.593115	1.932076	9.43E-19	1.71E-13	3.57E-14	5.89E-18	3.94E-16	368	1086	1211	547	738
MSC	-2.49943	-2.1905	-2.28006	-2.42892	-2.28682	9.75E-19	7.73E-12	9.17E-17	8.87E-14	5.34E-15	369	1503	759	1232	925
F2R	-3.84066	-3.46182	-3.34092	-3.64553	-3.6274	1.00E-18	2.54E-21	1.29E-20	9.34E-25	2.84E-19	370	153	332	88	326
CFH	-2.56288	-2.37111	-2.38705	-2.38483	-2.72486	1.09E-18	2.06E-19	7.00E-22	2.82E-20	6.39E-18	371	281	226	314	467
SUPT7L	-1.79244	-1.68971	-1.6657	-1.89356	-1.50972	1.10E-18	2.10E-14	5.73E-17	3.67E-16	3.87E-16	372	892	723	782	736
SERPINH1	-3.28234	-3.5573	-2.87589	-3.50294	-3.18791	1.12E-18	1.37E-20	4.93E-18	4.10E-22	2.34E-16	373	203	587	200	708
ETS1	-3.49608	-3.52173	-3.42077	-3.37506	-3.37656	1.16E-18	1.16E-20	2.85E-22	3.91E-24	8.35E-16	374	194	201	105	795
EPHB2	2.742366	2.754682	2.623738	2.867904	2.818569	1.21E-18	3.36E-19	5.34E-17	1.07E-21	2.32E-19	375	292	720	226	320
VSIR	-2.43342	-2.01757	-2.60547	-2.42217	-2.56167	1.22E-18	1.10E-13	2.77E-17	1.86E-16	3.30E-14	376	1043	685	737	1111
SNX18	-1.63258	-1.56487	-1.79601	-1.85839	-1.59831	1.28E-18	4.05E-17	3.94E-21	3.31E-21	1.70E-19	377	516	284	261	310
HGF	-2.16064	-1.73585	-2.3613	-1.98591	-2.39376	1.31E-18	4.94E-13	1.88E-18	1.37E-12	3.98E-17	378	1198	520	1542	575
GPX3	-1.45311	-1.52347	-0.89529	-1.54324	-1.17183	1.32E-18	5.10E-16	3.76E-07	1.53E-12	1.91E-09	379	659	3806	1558	2531
ADGRF5	-2.75372	-2.72499	-2.59484	-2.89357	-2.38677	1.32E-18	7.69E-20	2.38E-16	5.68E-24	5.49E-14	380	253	834	110	1162
PMP22	-2.77625	-2.67742	-2.47489	-2.90031	-2.89236	1.32E-18	8.21E-21	3.18E-16	8.84E-24	1.85E-20	381	181	853	121	228
FOXF2	-2.83011	-2.53892	-2.6131	-2.62064	-2.48675	1.33E-18	1.45E-17	1.90E-18	1.63E-19	3.90E-16	382	451	522	377	737
AHCY	2.749245	2.866515	2.674017	2.986367	2.761251	1.34E-18	1.64E-19	3.81E-18	3.49E-22	1.15E-22	383	270	573	197	109
LRRC8C	-1.72136	-1.51819	-1.90376	-1.61952	-1.76864	1.38E-18	1.58E-13	2.69E-18	2.03E-13	1.41E-15	384	1069	538	1315	830
HLA-DPB1	-4.02754	-4.44328	-3.51852	-3.94878	-3.92447	1.39E-18	1.68E-24	2.84E-16	2.05E-19	2.08E-17	385	41	845	386	540
CTSK	-4.30773	-3.86104	-3.38081	-4.13443	-3.96214	1.39E-18	1.34E-20	1.35E-18	1.20E-22	7.30E-19	386	202	504	172	364
TNFAIP2	-2.55135	-2.35435	-2.13412	-2.39195	-2.30647	1.41E-18	1.15E-20	2.00E-14	3.31E-15	2.72E-13	387	193	1167	936	1325
PLXND1	-3.79804	-3.89888	-3.93033	-3.69349	-3.8357	1.48E-18	8.33E-20	3.38E-22	1.19E-17	2.51E-20	388	259	207	586	239
DKK3	-4.21161	-4.1181	-3.84356	-4.53375	-4.36864	1.50E-18	9.75E-20	1.76E-20	8.53E-23	9.93E-18	389	264	343	167	497
NOTCH4	-2.62382	-2.59851	-2.64432	-2.62609	-2.41983	1.51E-18	2.59E-18	8.91E-22	3.60E-19	1.11E-13	390	374	234	409	1230
ARHGEF17	-3.81079	-4.0443	-3.88576	-4.23707	-3.73565	1.60E-18	9.86E-20	2.48E-23	2.20E-22	1.53E-19	391	265	144	189	308
SLC2A3	-3.2642	-3.07886	-3.25493	-3.2725	-3.2086	1.61E-18	1.80E-18	2.18E-20	7.39E-20	1.04E-16	392	352	350	339	651
MISP	3.592208	3.242784	3.751962	3.194669	3.285419	1.67E-18	1.28E-16	1.27E-19	2.18E-17	4.89E-21	393	585	402	612	189
SDC4	2.230507	2.494006	2.504873	2.750839	2.119307	1.68E-18	6.19E-15	4.65E-17	9.31E-19	3.13E-14	394	804	708	456	1106
OLFML2B	-3.18152	-3.42583	-3.17383	-3.28413	-3.23166	1.70E-18	7.89E-18	3.30E-18	2.33E-20	3.11E-17	395	419	553	309	560
OCIAD2	2.601761	2.272959	2.732155	2.320421	2.7783	1.72E-18	2.13E-17	5.97E-23	7.39E-21	6.58E-23	396	470	166	280	106
DPM1	1.667249	1.59697	1.625255	1.726629	1.236505	1.74E-18	3.24E-17	6.74E-17	4.15E-17	2.78E-12	397	502	734	651	1607
GPX2	3.263579	3.328786	3.328078	3.606831	3.762001	1.76E-18	3.16E-17	1.90E-18	1.94E-19	1.29E-19	398	497	521	385	301
HSPA1B	2.442091	2.225416	2.688547	2.368115	2.741425	1.79E-18	1.46E-14	1.33E-25	4.32E-16	2.81E-17	399	861	71	790	555
POLR2H	1.432438	1.337288	1.640146	1.480853	1.219972	1.80E-18	2.25E-14	2.96E-16	1.44E-15	1.96E-14	400	903	849	869	1055
HSPG2	-3.94766	-4.13407	-3.71771	-4.15874	-3.81386	1.80E-18	5.78E-22	7.00E-22	5.82E-23	6.61E-22	401	129	227	160	137
LMOD1	-1.91974	-1.39618	-2.10255	-1.69242	-2.09901	1.81E-18	3.32E-10	1.84E-14	1.49E-12	4.91E-18	402	2011	1160	1556	451
IGSF6	-2.00454	-2.09133	-2.20093	-2.29351	-2.09863	1.84E-18	9.96E-18	2.63E-20	1.67E-17	4.74E-19	403	433	353	596	343
FGFR1	-3.45891	-2.96162	-2.7117	-3.26515	-2.89233	1.88E-18	1.67E-19	3.22E-19	5.92E-21	6.38E-18	404	271	439	273	466
ADAMTS9	-3.47426	-3.17334	-3.17483	-3.15025	-2.76505	1.88E-18	1.33E-14	7.48E-21	2.90E-14	1.02E-13	405	852	316	1126	1222
SYPL1	1.36174	1.297752	1.493332	1.548685	1.468652	1.92E-18	1.68E-15	7.40E-17	5.55E-19	5.62E-12	406	732	746	433	1685
ERBB3	3.367382	3.092167	2.756435	3.381485	2.76164	1.93E-18	1.95E-23	1.87E-21	1.02E-23	2.40E-18	407	71	255	124	416
ISLR	-3.932	-3.78217	-3.44908	-3.77901	-3.90654	1.95E-18	3.60E-18	5.69E-21	2.56E-19	6.07E-21	408	386	310	394	193
SNRPD2	2.071826	2.001317	1.998152	2.009059	1.720071	2.08E-18	5.11E-18	7.04E-17	8.36E-19	4.97E-18	409	401	741	448	456
CD163	-2.33916	-2.44978	-2.46866	-2.31632	-2.36211	2.09E-18	4.93E-20	1.12E-19	7.68E-21	3.32E-15	410	241	395	281	880
FILIP1L	-3.2969	-3.58343	-3.23559	-3.54145	-3.48568	2.17E-18	5.27E-20	1.94E-19	5.98E-22	8.90E-19	411	242	420	212	374
DEFA1B	-1.25547	-1.00666	-1.35465	-1.18225	-0.95748	2.19E-18	2.68E-12	5.27E-17	7.79E-14	8.51E-08	412	1354	717	1220	3243
NDST1	-1.7851	-1.71608	-1.93287	-1.88763	-1.74825	2.24E-18	2.20E-17	3.92E-21	1.19E-18	3.51E-17	413	471	280	463	566
IRAG1	-3.08504	-2.9158	-3.25415	-3.32624	-3.25384	2.25E-18	1.33E-17	2.85E-18	9.92E-22	5.99E-19	414	449	545	223	357
SLC25A5	2.88075	2.585036	3.109971	2.864812	2.906645	2.30E-18	7.12E-18	2.05E-19	2.44E-17	2.37E-21	415	415	423	617	171
ATP2B4	-2.07413	-1.9039	-1.95911	-2.07861	-1.91715	2.37E-18	3.08E-19	3.03E-22	2.80E-19	3.31E-18	416	287	203	398	429
PTPRM	-2.94024	-2.91307	-2.57735	-2.98339	-2.91608	2.44E-18	1.44E-22	6.02E-21	1.36E-24	9.95E-21	417	107	312	91	216
TIE1	-1.58638	-1.48241	-1.50776	-1.41326	-0.99293	2.47E-18	1.49E-13	6.79E-15	7.43E-16	1.51E-05	418	1067	1090	828	4398
C1orf162	-1.81579	-1.86389	-2.01334	-1.95654	-1.68207	2.56E-18	8.51E-18	4.48E-19	4.89E-17	2.63E-12	419	424	458	661	1597
UGCG	-1.54583	-1.50462	-1.3788	-1.52225	-1.35898	2.62E-18	9.41E-20	1.86E-18	7.04E-22	2.62E-14	420	263	519	216	1079
ACP5	-3.79477	-4.11967	-4.27876	-4.36806	-4.54828	2.66E-18	1.38E-18	8.80E-22	9.19E-18	3.05E-20	421	343	233	573	245
NDUFB9	1.981775	2.090302	2.174095	2.053807	1.858611	2.71E-18	1.47E-21	6.41E-22	1.31E-19	2.29E-16	422	143	225	366	705
KRTCAP3	2.213461	2.329073	2.194797	2.28605	2.364995	2.79E-18	8.28E-19	6.31E-18	1.37E-19	4.61E-20	423	332	602	368	262
TSPAN11	-1.87108	-1.66421	-1.83538	-1.75659	-1.83542	2.82E-18	2.27E-18	1.75E-19	9.18E-19	5.93E-17	424	364	417	454	602
OLFML3	-1.42756	-1.35056	-1.55832	-1.50352	-1.60765	2.85E-18	5.09E-16	2.44E-19	9.64E-17	1.99E-20	425	658	430	694	231
GRK5	-2.56371	-2.4805	-2.41412	-2.43228	-2.37173	2.87E-18	5.15E-24	8.83E-20	3.28E-21	1.04E-18	426	58	388	260	381
PCOLCE	-3.58869	-3.48692	-3.50834	-3.48955	-3.79906	2.87E-18	2.77E-16	9.62E-18	3.53E-17	9.93E-20	427	620	626	642	292
ADA2	-2.2921	-2.05607	-2.5975	-2.25475	-2.75498	2.87E-18	8.94E-16	3.29E-20	3.47E-13	6.88E-17	428	690	362	1379	613
HSPB8	-1.61126	-1.24131	-1.53557	-1.36184	-1.47247	2.87E-18	2.70E-13	1.92E-15	4.93E-15	3.05E-15	429	1122	989	980	874
WNT5A	-3.56876	-3.26066	-3.48344	-3.44595	-3.54343	2.88E-18	5.91E-14	5.67E-20	4.26E-18	1.69E-16	430	993	379	523	677
PLEKHJ1	1.438947	1.697266	1.606501	1.620076	1.377904	2.94E-18	7.60E-17	9.63E-17	6.05E-17	4.06E-20	431	551	766	675	254
OSMR	-2.62846	-2.27513	-2.60171	-2.58241	-2.52137	2.96E-18	2.29E-13	1.77E-17	4.25E-18	4.67E-16	432	1108	661	522	750
ACTG2	-4.84092	-4.68968	-4.87055	-4.71084	-4.59446	2.97E-18	9.68E-17	5.40E-20	5.21E-18	1.92E-16	433	575	378	536	686
FKBP4	1.631927	1.780484	1.823278	1.705995	1.876091	3.02E-18	4.57E-20	4.94E-21	9.67E-20	6.56E-19	434	236	300	351	358
DOCK10	-2.01883	-1.73405	-1.94371	-2.00963	-2.15657	3.05E-18	4.91E-16	8.72E-17	9.51E-19	2.22E-18	435	653	755	458	410
LOXL2	-3.96041	-3.9448	-3.7942	-4.2392	-4.03461	3.06E-18	2.79E-17	3.72E-19	1.33E-21	2.57E-17	436	491	447	233	550
LAMB2	-2.63262	-2.59223	-2.19849	-2.60897	-2.37645	3.07E-18	9.05E-23	2.73E-18	1.48E-21	2.96E-19	437	96	541	237	327

NR3C1	-2.3113	-2.08765	-2.13986	-2.31805	-2.26315	3.17E-18	8.48E-16	5.76E-19	3.81E-17	5.31E-19	438	684	465	650	347
TRABD2A	2.180146	2.37277	2.351495	2.304567	2.419319	3.24E-18	8.10E-17	1.21E-17	1.24E-18	9.15E-21	439	555	643	466	210
ALPL	-1.5927	-1.26576	-1.45245	-1.33302	-1.20412	3.45E-18	9.66E-16	3.31E-15	2.66E-16	2.48E-10	440	697	1035	767	2195
HNRNPAB	1.990312	1.81814	2.174862	1.926037	2.085643	3.56E-18	1.19E-18	4.13E-21	9.88E-17	1.18E-21	441	339	289	699	158
RAC2	-2.17745	-1.88948	-2.49924	-2.07444	-2.10079	3.64E-18	9.10E-18	5.11E-21	8.60E-15	6.42E-15	442	426	306	1015	941
ZNF385A	-2.00116	-1.71103	-1.90348	-2.17899	-1.97637	3.73E-18	1.89E-11	9.09E-13	4.94E-18	7.08E-13	443	1607	1576	529	1445
CYGB	-3.85958	-3.96161	-3.63972	-4.08703	-4.11566	3.76E-18	1.80E-21	6.67E-21	1.31E-23	1.06E-20	444	146	315	132	217
RIPOR2	-1.50706	-1.23381	-1.66081	-1.48391	-1.67674	3.83E-18	4.68E-14	1.75E-15	1.66E-14	2.20E-14	445	963	983	1076	1063
COX6C	2.42708	2.319028	2.656027	2.409709	2.539333	3.85E-18	1.10E-18	4.90E-18	2.25E-18	1.78E-18	446	337	584	495	400
SELENOM	-2.73999	-2.90383	-2.79369	-2.69202	-2.82241	4.04E-18	8.75E-16	1.79E-16	1.19E-16	9.13E-16	447	686	809	710	802
PLXNB1	1.80977	2.080156	2.119442	1.848293	2.076541	4.05E-18	6.42E-19	1.86E-20	5.77E-16	3.58E-23	448	324	344	813	103
SNRPF	2.206302	2.19983	2.30086	2.303848	2.16958	4.11E-18	2.02E-19	5.74E-20	6.42E-20	1.72E-20	449	280	380	334	226
TIGIT	-1.2899	-1.00976	-1.19578	-1.15292	-1.50086	4.13E-18	7.44E-15	7.11E-16	1.86E-15	9.34E-17	450	819	905	894	636
SYNGR2	2.38009	2.378087	2.403968	2.373804	2.286958	4.15E-18	2.66E-17	4.73E-16	1.48E-15	1.25E-17	451	486	874	870	511
ADAMTS2	-3.70485	-3.44905	-3.56146	-3.42148	-3.53058	4.20E-18	2.42E-17	1.05E-17	1.19E-18	1.01E-16	452	480	631	462	641
STAT2	-1.90345	-2.07328	-2.1192	-2.03462	-2.00234	4.20E-18	2.98E-16	2.20E-21	1.98E-17	1.13E-18	453	624	261	602	383
PGF	-1.71351	-1.54875	-1.57679	-1.51102	-1.24617	4.29E-18	1.35E-14	6.37E-16	2.20E-15	1.21E-08	454	857	891	909	2857
LEF1	-2.55464	-2.2549	-2.20735	-2.763	-2.21376	4.34E-18	3.50E-14	5.05E-17	5.92E-17	3.62E-13	455	940	713	672	1359
TRAP1	2.84836	2.728883	2.68161	2.988884	2.788242	4.51E-18	3.50E-18	1.48E-19	1.48E-21	9.34E-21	456	385	409	239	213
LTBP2	-2.89254	-2.34067	-2.43548	-2.67824	-2.89108	4.58E-18	2.20E-14	2.90E-18	1.01E-17	2.22E-18	457	900	546	578	411
ZNHIT1	1.081049	0.992274	1.129664	1.154549	1.053702	4.61E-18	2.55E-14	6.53E-17	2.47E-18	1.34E-14	458	915	731	500	1016
MFAP4	-3.50128	-3.17394	-3.53855	-3.26431	-3.86888	4.65E-18	9.29E-14	7.82E-19	2.90E-15	8.20E-22	459	1028	474	922	143
KRT19	4.058845	3.370054	4.380045	3.705884	3.641696	4.68E-18	3.44E-18	3.07E-20	2.59E-19	2.13E-18	460	384	360	396	408
MCTP1	-1.96533	-1.66455	-2.2199	-1.75675	-2.34975	5.07E-18	1.37E-09	2.45E-12	1.49E-08	3.98E-20	461	2225	1686	2831	252
FLT1	-3.07519	-2.98527	-3.29824	-3.21932	-2.78069	5.14E-18	2.64E-16	1.86E-17	1.38E-20	1.29E-11	462	615	662	299	1800
PLCG2	-2.02542	-1.83492	-2.37467	-2.2898	-2.04857	5.35E-18	3.26E-15	1.53E-17	1.67E-15	1.90E-14	463	759	653	886	1050
HNRNPU	1.512465	1.399589	1.408422	1.496622	1.446454	5.38E-18	1.71E-17	2.37E-15	2.28E-18	1.29E-14	464	459	1003	496	1014
MYLK	-4.07537	-3.92402	-3.74313	-4.25445	-4.05278	5.41E-18	2.96E-19	2.14E-17	4.75E-26	4.47E-20	465	285	671	62	260
ANXA4	2.651278	2.203585	2.943875	2.591996	2.619897	5.43E-18	8.21E-18	2.09E-22	8.03E-21	5.26E-22	466	422	189	284	131
SHANK3	-2.46111	-2.13597	-2.36356	-2.21016	-2.3556	5.48E-18	7.79E-17	4.58E-20	3.66E-20	1.33E-19	467	552	371	323	303
BMP8A	-2.7716	-2.65861	-2.88074	-2.85823	-2.74619	5.54E-18	3.77E-19	3.08E-20	1.70E-20	8.66E-20	468	303	361	302	288
MAL2	2.705848	2.709813	3.469713	2.696326	3.430249	5.74E-18	2.88E-16	1.72E-23	8.78E-18	1.41E-23	469	622	135	569	95
TXN	3.318777	2.975475	3.324596	3.094744	2.958674	6.00E-18	1.29E-20	1.13E-21	1.93E-18	3.78E-23	470	201	239	488	105
C11orf96	-4.33763	-4.59326	-4.54283	-5.10027	-4.96661	6.33E-18	1.92E-16	2.02E-20	1.23E-19	5.61E-19	471	605	347	364	351
MAN2B1	-1.80538	-2.00045	-1.89983	-1.93169	-1.63016	6.42E-18	3.50E-19	2.43E-19	2.86E-17	1.90E-14	472	301	429	623	1049
SWAP70	-1.51865	-1.33425	-1.5197	-1.23708	-1.36422	6.65E-18	6.63E-17	8.31E-18	1.18E-15	2.92E-16	473	538	615	858	723
CD27	-2.05328	-1.54587	-2.2051	-1.98224	-1.9372	6.68E-18	3.00E-10	4.78E-12	1.41E-09	1.19E-09	474	1990	1774	2461	2453
PTPRK	1.640441	1.445479	1.969078	1.674112	1.540806	6.68E-18	1.71E-16	5.40E-20	4.95E-17	2.20E-14	475	599	377	664	1064
HSD17B14	-1.26178	-1.01417	-1.14777	-1.13931	-1.17099	6.68E-18	2.18E-13	7.33E-17	2.10E-15	3.14E-15	476	1104	745	901	877
CTSL	-3.11082	-3.08836	-3.37257	-3.53516	-3.56941	6.78E-18	2.57E-18	3.71E-17	6.94E-21	2.00E-21	477	373	695	278	167
PDXK	1.434253	1.467459	1.558062	1.453332	1.209875	6.79E-18	8.47E-17	2.00E-16	3.01E-13	1.30E-15	478	559	817	1359	824
COL5A3	-2.4561	-2.64295	-2.60937	-2.69993	-2.73107	7.08E-18	2.03E-18	3.73E-18	5.13E-19	3.52E-21	479	360	568	431	179
COX7A2	1.890522	1.894611	2.169581	1.950572	2.123453	7.25E-18	3.40E-21	3.18E-23	1.53E-19	8.19E-24	480	160	151	374	90
CD48	-1.95011	-1.99533	-2.26775	-2.36413	-2.09207	7.29E-18	6.91E-17	2.31E-14	2.43E-16	9.74E-13	481	547	1176	759	1480
RIC1	-1.14071	-0.97387	-1.04085	-1.03195	-0.90169	7.39E-18	3.15E-12	8.35E-15	4.69E-13	7.90E-09	482	1371	1109	1416	2762
PREX1	-1.48271	-1.37788	-1.46577	-1.51171	-1.6119	7.48E-18	2.38E-15	3.57E-18	2.20E-16	4.45E-18	483	749	560	748	444
SEC14L1	-1.92015	-2.1256	-2.41891	-2.21621	-2.24079	7.48E-18	6.09E-17	3.34E-21	2.39E-16	5.41E-19	484	536	275	758	348
PTPN7	-1.68533	-1.22975	-1.96143	-1.68145	-1.62896	7.61E-18	3.12E-13	1.56E-16	3.21E-14	1.17E-12	485	1138	804	1140	1503
C3AR1	-2.14188	-2.1062	-2.14712	-2.16029	-2.16329	7.63E-18	1.58E-16	2.54E-18	5.31E-18	1.01E-16	486	596	535	539	642
KLHDC8B	-1.58892	-1.57956	-1.64875	-1.62915	-1.41274	7.63E-18	5.15E-16	8.72E-22	5.67E-16	7.95E-14	487	660	232	811	1200
RAMP2	-2.7377	-2.59354	-2.81403	-2.52808	-2.53095	7.68E-18	7.44E-13	1.11E-14	2.58E-16	3.68E-14	488	1227	1125	766	1124
SH3BGRL2	1.897875	2.098529	2.526423	2.206134	2.150093	7.96E-18	7.88E-20	5.27E-23	8.21E-22	1.90E-21	489	255	163	221	165
TLR2	-2.0024	-1.98777	-2.15375	-2.08378	-1.95672	7.97E-18	1.29E-16	8.74E-21	5.58E-18	4.18E-18	490	587	318	542	440
HSPA6	-2.0803	-1.7815	-2.20142	-2.06777	-1.83612	8.23E-18	2.18E-14	3.92E-21	2.69E-16	2.75E-14	491	897	278	770	1088
PLAT	-4.01036	-3.91147	-3.9098	-4.31433	-4.10202	8.23E-18	3.02E-19	9.16E-20	5.37E-22	1.26E-18	492	286	389	209	390
HLA-DQB1	-2.71489	-2.89186	-2.42225	-2.503	-2.75234	8.38E-18	4.43E-18	9.96E-13	1.40E-13	1.22E-16	493	396	1592	1272	658
HNRNPL	1.229077	1.066828	1.328508	1.07718	1.353832	8.49E-18	3.93E-11	1.61E-13	2.77E-10	5.82E-18	494	1712	1388	2193	463
PTBP3	1.482537	1.510516	1.628992	1.545439	1.667447	8.58E-18	3.04E-15	8.79E-18	5.51E-16	8.95E-18	495	756	621	808	491
COX6B1	2.124247	1.775198	2.23007	1.821622	2.061829	8.82E-18	7.35E-13	1.66E-15	1.89E-12	2.14E-19	496	1225	976	1582	318
RAB11FIP1	2.250495	2.363089	2.229715	2.391154	2.064501	9.09E-18	9.26E-18	2.70E-21	5.85E-18	3.63E-15	497	427	268	546	892
CBL	-1.08549	-0.86434	-1.26424	-0.99165	-1.1326	9.44E-18	2.74E-14	1.23E-16	9.24E-13	4.01E-12	498	923	781	1487	1647
DYNLL2	1.224827	1.200563	1.392229	1.32449	1.361581	9.61E-18	5.77E-18	1.07E-14	1.11E-15	6.84E-16	499	408	1121	854	772
JCHAIN	-6.24063	-5.83759	-6.17609	-6.00549	-6.07424	9.68E-18	6.63E-17	5.08E-16	1.16E-17	1.05E-16	500	539	880	583	652
LSM4	2.047991	2.087136	2.147909	2.176287	1.543103	9.90E-18	5.59E-15	3.80E-17	6.80E-17	1.61E-11	501	793	696	679	1828
CACNA1H	-3.26788	-2.81473	-2.90392	-3.19669	-2.92648	1.02E-17	1.19E-20	8.83E-19	1.20E-22	5.50E-15	502	196	481	171	927
FAM83H	2.415508	2.552878	2.888982	2.594747	2.578218	1.04E-17	4.24E-20	3.03E-21	4.04E-18	4.79E-24	503	231	273	517	79
WWTR1	-3.57059	-3.48078	-3.5053	-3.67668	-3.46009	1.04E-17	4.63E-21	5.15E-21	3.28E-21	4.34E-20	504	167	308	258	259
ABHD11	2.518396	2.429519	2.48553	2.474567	2.702052	1.06E-17	4.29E-19	1.53E-20	1.18E-19	9.70E-22	505	308	337	359	154
IGHG4	-7.48331	-7.49874	-7.43015	-7.32533	-6.87906	1.06E-17	1.84E-18	1.68E-20	2.46E-16	1.35E-14	506	354	338	761	1018
RGS3	-1.54542	-1.58781	-1.58357	-1.61409	-1.44198	1.06E-17	2.37E-14	3.52E-16	1.15E-16	1.50E-13	507	910	858	708	1266
PPP1R1B	2.937314	2.673504	2.932285	2.769329	3.119238	1.11E-17	1.10E-16	2.42E-19	2.28E-23	7.71E-20	508	579	428	138	280
ITPR3	2.194016	2.152325	2.230299	2.211929	2.114232	1.11E-17	8.53E-16	1.50E-20	4.16E-17	1.07E-18	509	685	336	652	382
PREX2	-1.9617	-1.78998	-1.8789	-1.99507	-2.09701	1.12E-17	4.27E-16	8.27E-17	7.23E-22	1.02E-19	510	641	752	217	295

ATP5MF	2.10065	1.945265	2.070469	1.961787	2.282122	1.17E-17	5.36E-19	9.34E-19	1.23E-17	2.57E-21	511	315	485	588	174
SMIM22	2.894004	2.722256	3.286723	2.952696	3.207692	1.17E-17	4.16E-17	7.89E-26	3.67E-21	8.76E-22	512	518	62	263	147
HEBP2	1.324324	1.408512	1.49186	1.506908	1.413392	1.17E-17	1.24E-20	1.08E-17	5.58E-20	1.03E-16	513	198	636	332	645
HMGA1	3.424818	3.332451	3.265719	3.319941	3.672875	1.18E-17	1.06E-18	3.55E-19	1.02E-19	3.06E-22	514	335	445	352	118
ST3GAL2	-1.25194	-1.06034	-1.40691	-1.01791	-1.09679	1.21E-17	1.95E-15	2.09E-22	7.96E-13	6.65E-13	515	736	190	1472	1438
EDNRA	-2.97932	-2.94897	-2.7435	-3.02132	-2.73091	1.21E-17	3.02E-18	4.48E-18	2.11E-21	8.41E-17	516	379	578	248	627
GPA33	2.637938	2.773604	2.806098	2.494881	2.062054	1.22E-17	4.16E-19	5.40E-19	5.65E-17	8.43E-12	517	307	463	671	1738
DLL4	-2.05465	-1.79501	-2.0958	-1.89498	-1.63634	1.22E-17	3.88E-12	7.53E-16	1.09E-14	6.17E-08	518	1401	912	1043	3167
RDX	-1.86843	-1.44258	-1.87784	-1.46331	-1.92865	1.29E-17	3.86E-09	2.40E-14	6.26E-10	1.05E-14	519	2394	1178	2318	990
SH3BP5	-1.85528	-1.2991	-1.7721	-1.59403	-1.63916	1.30E-17	3.75E-12	5.02E-17	1.46E-16	7.48E-15	520	1397	712	726	957
TBX2	-3.84098	-3.80056	-3.9723	-4.09124	-4.11025	1.31E-17	1.08E-22	2.35E-22	9.01E-24	1.52E-19	521	101	193	123	307
PDE4DIP	-1.78727	-1.62181	-2.05342	-1.84302	-1.78775	1.33E-17	7.93E-15	3.63E-18	3.71E-14	1.40E-12	522	824	565	1155	1527
RIMKLB	-1.56465	-1.31901	-1.50898	-1.43525	-1.18958	1.33E-17	9.59E-16	3.16E-18	9.50E-14	5.67E-10	523	695	551	1241	2325
ADAMTS4	-2.48007	-2.36096	-2.50193	-2.61512	-2.37397	1.36E-17	1.34E-14	2.48E-14	6.82E-18	1.33E-14	524	853	1181	555	1015
CHD7	1.331065	1.376524	1.433119	1.461685	1.501141	1.36E-17	1.56E-16	2.58E-16	1.90E-14	7.77E-16	525	594	838	1092	787
C19orf33	2.548753	2.748441	2.935938	2.536178	2.564582	1.38E-17	2.97E-17	4.58E-19	3.93E-20	8.04E-15	526	495	459	325	965
CAPN15	1.911634	1.651019	1.915027	1.669429	1.727834	1.38E-17	1.08E-15	8.25E-24	1.66E-17	7.13E-18	527	703	127	595	475
MYH11	-3.35409	-3.07872	-3.40968	-3.33708	-3.02791	1.41E-17	1.45E-16	8.68E-22	1.80E-18	1.37E-17	528	592	230	484	515
THBS1	-4.12844	-3.77501	-4.26334	-3.72811	-4.17599	1.41E-17	2.78E-14	9.64E-16	2.51E-18	1.99E-17	529	926	928	502	537
IGHG2	-7.25336	-7.35538	-7.30675	-7.07739	-6.74743	1.42E-17	1.10E-18	5.03E-21	6.44E-16	1.25E-14	530	336	304	823	1012
COX5A	1.890563	1.654211	1.987478	1.728991	1.959203	1.46E-17	4.87E-17	3.36E-18	1.40E-14	1.44E-20	531	528	555	1064	222
CBLC	1.983676	2.206587	2.073454	2.122298	2.095216	1.52E-17	3.13E-19	1.55E-19	2.42E-19	8.83E-22	532	289	411	391	149
TXNDC15	-1.77084	-1.42288	-1.56015	-1.59491	-1.8276	1.53E-17	1.30E-16	4.28E-15	4.61E-13	3.65E-16	533	588	1051	1415	734
LAMC1	-2.82378	-3.0183	-2.5922	-2.95075	-2.67191	1.55E-17	8.26E-21	3.41E-18	1.23E-19	1.50E-17	534	183	558	363	524
GJC1	-2.00049	-1.97836	-1.88265	-2.13515	-1.87358	1.61E-17	4.57E-20	1.06E-16	2.97E-21	9.53E-17	535	235	774	253	639
HLA-DPA1	-4.19368	-4.48987	-3.46665	-3.92157	-4.36086	1.64E-17	8.75E-22	4.83E-14	1.04E-15	3.50E-17	536	134	1238	846	565
CEBPG	1.258979	1.134544	1.331405	1.138397	1.2729	1.65E-17	1.31E-10	6.73E-15	6.87E-11	5.91E-17	537	1864	1089	2018	601
CH25H	-1.9488	-1.57798	-1.91205	-1.75045	-1.62649	1.65E-17	6.53E-12	1.39E-14	8.02E-14	1.03E-14	538	1473	1138	1221	985
CLEC2D	-1.23012	-1.04456	-0.98095	-1.14545	-1.18866	1.66E-17	1.44E-12	7.70E-19	3.93E-14	5.05E-15	539	1286	473	1159	921
TMEM119	-2.84376	-2.53099	-2.47071	-2.75192	-3	1.67E-17	2.13E-15	5.73E-17	3.21E-19	7.23E-18	540	744	724	402	476
TNS2	-2.72456	-2.83707	-2.63706	-2.98489	-2.75337	1.72E-17	7.96E-20	1.66E-18	1.46E-22	4.53E-21	541	257	515	177	187
HCST	-1.58354	-1.40669	-1.4862	-1.70427	-1.79481	1.79E-17	5.51E-13	5.05E-13	3.83E-15	7.96E-16	542	1204	1515	953	790
MS4A7	-2.13018	-2.12593	-2.50493	-2.26944	-2.51974	1.79E-17	1.66E-16	3.65E-23	4.79E-17	1.92E-17	543	597	158	659	534
ADAP2	-2.03929	-2.01399	-2.22425	-2.07766	-2.515	1.83E-17	1.65E-15	2.78E-18	1.92E-15	3.15E-18	544	729	543	897	425
PLK2	-2.2594	-2.05809	-2.04323	-2.32838	-2.25754	1.90E-17	1.30E-15	1.32E-13	8.16E-16	7.48E-16	545	713	1359	836	780
FASN	2.452803	2.765961	2.597584	2.835166	2.455213	1.91E-17	6.22E-19	1.06E-17	4.18E-21	7.99E-20	546	321	635	267	282
SEPTIN1	-1.77187	-1.38613	-2.3402	-1.82663	-1.72144	1.92E-17	2.75E-16	2.72E-18	4.77E-14	6.35E-10	547	618	540	1171	2349
PKMYT1	1.63201	1.500415	1.500347	1.454909	1.624617	1.95E-17	6.33E-16	5.69E-17	1.90E-16	1.63E-18	548	671	722	738	398
INHBA	-4.12741	-4.06306	-4.29957	-4.31546	-4.32525	1.98E-17	1.34E-14	4.08E-19	2.72E-16	6.10E-17	549	855	450	771	609
PCBD1	2.032376	2.113231	1.968991	2.060259	2.148565	1.98E-17	2.87E-17	8.27E-18	2.10E-17	2.18E-17	550	493	614	607	542
PRR16	-2.09198	-1.69842	-1.77565	-1.81304	-1.84283	2.00E-17	3.61E-14	1.81E-16	2.87E-14	2.89E-15	551	943	813	1123	868
WNT2	-1.90529	-1.64607	-1.77253	-2.01239	-1.91953	2.07E-17	1.66E-14	1.58E-16	1.76E-17	5.45E-16	552	874	806	599	762
GAS7	-2.28264	-1.72651	-1.76004	-1.88673	-1.86435	2.15E-17	1.57E-14	2.08E-18	2.51E-16	4.62E-17	553	870	529	763	586
OSTM1	-1.29417	-1.18158	-1.28618	-1.37338	-1.50274	2.23E-17	3.60E-14	6.77E-17	1.54E-16	2.04E-16	554	942	735	731	692
MYO9B	-1.41343	-1.55385	-1.78164	-1.55046	-1.827	2.24E-17	4.86E-14	5.63E-15	8.94E-15	2.23E-14	555	965	1078	1020	1066
IQSEC1	-1.91596	-1.70646	-1.75073	-1.79294	-1.66079	2.24E-17	2.13E-15	7.92E-18	6.19E-16	6.02E-15	556	741	612	821	935
TRIM2	1.903097	1.760596	2.196729	1.887396	2.422469	2.28E-17	2.20E-14	1.32E-19	7.75E-15	7.21E-17	557	899	405	1010	617
COL8A1	-3.85377	-3.23416	-2.88063	-3.34832	-3.76208	2.28E-17	2.60E-13	5.47E-16	9.05E-15	7.90E-17	558	1120	885	1022	625
ADAMTS1	-2.97927	-2.78386	-2.79581	-2.73615	-2.80919	2.38E-17	2.68E-18	2.59E-21	1.34E-19	7.95E-18	559	375	267	367	483
HERC1	-1.5286	-1.40482	-1.63259	-1.38975	-1.39063	2.40E-17	2.78E-18	1.16E-18	2.07E-16	3.66E-14	560	376	497	744	1122
PLSCR3	-1.77597	-1.52642	-1.74288	-1.67078	-1.58039	2.42E-17	4.36E-13	8.14E-16	1.34E-16	6.06E-13	561	1180	918	721	1424
RAB31	-3.52864	-3.46016	-3.38139	-3.67249	-3.84917	2.46E-17	4.71E-18	1.17E-18	8.73E-21	3.42E-22	562	398	499	286	119
NOTCH3	-3.54613	-3.63963	-3.33502	-3.50605	-3.39357	2.47E-17	1.54E-19	5.25E-21	1.45E-21	1.14E-17	563	269	309	236	506
PTGES3	1.879571	1.591508	1.625473	1.812577	1.811054	2.51E-17	3.43E-16	3.23E-17	1.21E-17	1.31E-17	564	631	690	587	512
IGHG3	-7.21614	-7.45049	-7.34482	-7.17938	-6.88896	2.52E-17	2.39E-18	8.67E-21	5.05E-16	8.79E-16	565	368	317	798	798
CHCHD2	2.116113	1.93254	2.080961	2.063178	2.244883	2.59E-17	1.81E-17	2.91E-18	1.97E-17	8.83E-21	566	466	547	600	208
ZNF706	1.299536	1.362586	1.345676	1.446479	1.027132	2.60E-17	1.01E-15	5.01E-13	5.05E-17	1.85E-09	567	699	1512	665	2527
RBM47	1.899072	1.837092	2.111351	1.736458	1.645521	2.60E-17	6.81E-15	8.98E-18	4.24E-13	2.48E-12	568	810	622	1403	1589
RASGRF2	-2.38033	-2.09625	-1.83231	-2.18512	-2.18585	2.67E-17	1.14E-16	1.25E-17	4.64E-19	3.61E-16	569	582	645	429	732
RORA	-1.79819	-1.38134	-2.0418	-1.5999	-1.86455	2.75E-17	6.81E-14	1.45E-21	1.05E-15	9.89E-17	570	1005	249	848	640
TIMP1	-3.37884	-3.84328	-3.41307	-3.3329	-3.92017	2.82E-17	6.99E-16	1.95E-18	1.04E-17	6.07E-17	571	674	524	579	605
PTPRS	-2.39603	-2.00229	-2.01274	-2.34056	-2.39635	2.85E-17	1.87E-17	3.39E-18	1.21E-19	4.23E-17	572	467	556	362	580
CFTR	2.511524	2.962268	2.487928	3.158521	2.894785	2.85E-17	4.11E-20	4.44E-17	3.88E-21	3.62E-21	573	229	705	264	180
TNFAIP6	-1.52845	-1.4372	-1.50507	-1.53029	-1.53559	2.90E-17	5.63E-12	6.50E-14	6.04E-13	8.50E-15	574	1454	1272	1446	969
AGPAT2	2.157136	2.188608	2.31457	2.15703	2.274305	2.94E-17	3.41E-19	3.45E-22	1.46E-18	1.11E-12	575	295	210	473	1499
ZBTB46	-2.00525	-2.01198	-2.08115	-2.06348	-2.05667	2.98E-17	1.25E-17	3.96E-21	1.70E-18	6.76E-22	576	447	285	481	138
SLC36A1	-1.21965	-0.91788	-1.10069	-1.18277	-1.15006	2.98E-17	3.67E-11	6.41E-16	5.03E-15	2.64E-12	577	1696	892	981	1599
ATP1B1	3.31205	2.741981	3.619923	2.913181	3.396811	2.98E-17	2.93E-17	1.01E-19	1.48E-17	3.48E-22	578	494	390	592	122
TIMP3	-4.06065	-3.69102	-4.11102	-3.88793	-4.89667	3.04E-17	4.80E-19	9.53E-19	9.62E-19	5.23E-22	579	312	488	459	130
TMC5	2.67771	2.566241	3.427902	2.793869	3.238158	3.05E-17	2.67E-16	1.73E-24	5.85E-18	7.39E-25	580	616	99	545	64
GLIS2	-1.66021	-1.55854	-1.66407	-1.761	-1.62975	3.08E-17	3.38E-17	1.33E-18	1.13E-19	2.29E-16	581	504	502	355	706
FLNA	-3.65139	-3.77	-3.76291	-3.92054	-4.08277	3.08E-17	2.56E-16	3.93E-19	2.23E-18	3.07E-24	582	613	448	493	77
ICAM1	-2.84788	-2.76692	-2.48217	-2.7814	-2.86028	3.10E-17	5.59E-19	3.58E-19	1.57E-21	4.23E-19	583	317	446	242	338

FHL1	-3.0647	-2.67496	-3.23493	-2.84598	-3.2363	3.16E-17	3.26E-15	3.64E-23	1.77E-18	5.31E-20	584	760	155	483	265
TPSAB1	-3.69197	-3.04008	-3.20931	-3.40664	-3.41998	3.18E-17	1.04E-13	5.96E-14	1.64E-15	2.34E-14	585	1039	1264	882	1069
CALHM6	-1.92431	-2.05733	-2.14145	-2.43059	-1.80314	3.21E-17	3.30E-13	5.81E-12	1.09E-16	6.52E-12	586	1149	1803	703	1707
CERCAM	-3.07147	-3.02852	-2.91944	-3.25209	-3.34457	3.23E-17	1.22E-18	9.50E-19	1.53E-21	2.34E-21	587	340	486	241	170
SPINK1	3.508008	3.458925	3.69262	3.968823	3.750456	3.33E-17	7.12E-19	1.72E-19	7.92E-22	1.04E-16	588	329	416	219	650
GATA6	1.868215	1.737475	2.353288	1.772874	2.087209	3.40E-17	3.15E-13	4.18E-19	3.13E-17	2.79E-19	589	1142	451	631	324
ITGA6	2.116933	2.429375	2.313411	2.825653	2.082609	3.42E-17	7.30E-17	1.45E-16	3.45E-23	4.76E-15	590	548	794	144	920
MAT2A	1.565954	1.415444	1.459556	1.592466	1.556537	3.45E-17	1.45E-15	4.91E-14	2.08E-15	7.53E-16	591	723	1244	900	782
HCK	-2.72699	-2.69824	-2.70386	-2.65961	-2.73844	3.52E-17	4.73E-17	3.92E-17	8.71E-18	2.34E-16	592	526	700	568	707
GPR183	-2.01363	-1.89634	-2.44897	-2.42867	-2.57901	3.55E-17	3.08E-14	2.07E-16	2.67E-13	1.95E-16	593	932	818	1349	688
FGD2	-2.23136	-1.98952	-2.78774	-2.18824	-2.84003	3.58E-17	7.98E-18	1.48E-21	1.18E-15	7.57E-18	594	421	251	856	479
NAGK	-1.45057	-1.44159	-1.4438	-1.49079	-1.18301	3.60E-17	4.81E-13	3.67E-15	1.10E-13	2.15E-12	595	1194	1041	1249	1565
MYH14	3.06536	3.074073	3.415132	3.107943	3.00424	3.63E-17	1.06E-17	1.64E-19	7.63E-24	6.76E-19	596	436	413	117	359
CD300A	-2.50221	-2.19549	-2.72898	-2.34273	-2.24169	4.04E-17	6.39E-13	2.01E-17	9.08E-13	2.67E-10	597	1214	666	1485	2210
TUBB4B	2.295977	2.176819	2.570261	2.20071	2.40829	4.14E-17	4.09E-18	4.51E-20	3.91E-16	2.96E-20	598	393	370	787	244
ALDH1A3	-2.44869	-2.08743	-2.15975	-2.41151	-2.12779	4.16E-17	5.04E-14	7.34E-15	6.52E-19	1.01E-12	599	971	1096	439	1484
ACKR3	-1.61139	-1.65477	-1.73768	-1.95644	-1.69949	4.30E-17	6.91E-15	2.42E-17	4.33E-18	1.78E-13	600	812	680	526	1284
EDIL3	-3.20446	-2.79542	-3.34519	-2.8501	-3.46404	4.33E-17	2.03E-12	3.88E-18	1.62E-16	9.29E-18	601	1325	575	733	494
CPQ	-2.16163	-1.77121	-2.12983	-1.9892	-2.18527	4.39E-17	3.05E-13	6.66E-14	2.00E-13	1.12E-16	602	1134	1274	1312	655
EFEMP2	-3.33161	-3.30458	-2.95929	-3.36194	-3.56011	4.39E-17	1.82E-19	5.55E-19	3.26E-21	2.43E-20	603	277	464	257	236
PIK3CG	-1.93748	-2.12816	-2.21343	-2.39371	-2.30195	4.54E-17	1.03E-14	1.80E-16	8.94E-15	1.29E-16	604	838	812	1019	663
NCKAP5L	-1.56929	-1.44171	-1.38946	-1.44793	-1.38362	4.67E-17	1.54E-10	1.49E-14	5.30E-12	1.09E-13	605	1890	1143	1680	1227
PTPRF	2.461444	2.32205	2.55675	2.378724	2.339413	4.72E-17	9.25E-19	4.39E-21	4.41E-23	2.36E-15	606	333	293	150	859
CHST2	-2.3243	-1.81688	-2.07188	-2.27783	-1.81057	4.75E-17	7.43E-12	1.08E-13	5.00E-13	9.36E-10	607	1495	1326	1423	2420
UQCRHL	1.946236	2.134053	2.13541	2.187765	2.193798	4.78E-17	5.66E-21	2.90E-19	3.74E-19	1.95E-17	608	170	436	413	535
EBF1	-1.50466	-1.38382	-1.56816	-1.57724	-1.28054	4.86E-17	1.29E-16	1.90E-19	1.69E-18	4.66E-14	609	586	419	480	1146
C4B	-2.42043	-2.46763	-2.47534	-2.8204	-2.33591	4.86E-17	6.73E-17	2.85E-21	6.65E-19	4.56E-19	610	542	271	440	342
AMD1	1.156947	1.42948	1.418538	1.509899	1.321539	4.94E-17	8.51E-18	9.76E-16	1.60E-19	1.12E-16	611	423	929	376	653
FPR1	-1.65977	-1.32803	-1.89135	-1.56152	-1.64721	4.97E-17	1.17E-12	1.56E-14	1.27E-14	2.21E-12	612	1263	1144	1058	1571
LIMS3	-1.72743	-1.78074	-2.08929	-2.11282	-1.82158	5.28E-17	1.78E-13	5.62E-18	2.93E-15	1.88E-14	613	1089	595	924	1047
LCN2	4.496936	4.254715	5.21128	4.131034	4.820236	5.30E-17	7.79E-15	7.55E-19	7.21E-17	3.55E-17	614	822	472	682	567
ALDH2	2.117663	1.952433	2.236892	2.242954	2.045865	5.44E-17	1.58E-16	1.34E-15	1.31E-18	7.57E-19	615	595	959	467	367
VAV1	-1.69199	-1.23816	-1.66281	-1.40913	-1.67306	5.45E-17	6.79E-12	9.33E-12	2.42E-11	2.03E-13	616	1480	1863	1890	1297
MRPS35	1.733912	1.654749	1.879896	1.597946	1.632905	5.65E-17	5.04E-16	4.92E-18	9.06E-18	3.20E-18	617	656	585	571	428
PDE4D	-1.61919	-1.2024	-1.93731	-1.42033	-1.44554	5.65E-17	5.43E-14	2.54E-18	2.55E-10	7.36E-09	618	981	536	2180	2747
PER1	-1.36966	-1.36982	-1.55365	-1.49958	-1.60132	5.67E-17	1.17E-13	1.13E-14	1.18E-15	1.72E-16	619	1048	1126	857	679
NOS3	-2.5075	-2.47038	-2.69315	-2.40596	-2.4459	5.80E-17	6.11E-15	2.13E-21	2.25E-15	1.11E-14	620	802	259	912	998
ESM1	-3.39302	-3.33623	-3.47582	-3.91552	-3.18608	5.88E-17	9.68E-17	1.18E-15	8.29E-18	1.02E-12	621	571	950	565	1485
OLFML2A	-3.56192	-3.67347	-3.34748	-3.76767	-3.48136	5.88E-17	3.23E-21	4.32E-19	2.33E-23	1.11E-17	622	158	455	139	505
ARID5A	-1.48933	-1.44413	-1.27722	-1.51738	-1.12186	5.89E-17	6.11E-15	3.58E-18	6.46E-16	1.82E-11	623	801	561	824	1848
CTHRC1	-3.93189	-3.60784	-3.41686	-3.3746	-4.13211	5.93E-17	2.67E-14	1.11E-15	1.71E-15	5.04E-17	624	922	945	889	590
TNRC18	1.57159	1.345528	1.608399	1.706651	1.727017	6.13E-17	7.11E-15	3.94E-15	5.32E-16	3.93E-15	625	814	1046	805	897
AAK1	-1.34421	-1.11465	-1.26688	-1.32565	-1.28367	6.23E-17	2.08E-11	1.68E-13	1.70E-14	1.12E-11	626	1621	1396	1078	1784
HDGF	2.152926	1.847238	2.099491	1.951749	2.07103	6.34E-17	3.35E-15	6.26E-18	6.92E-15	9.25E-22	627	763	600	1002	152
HBM	-0.98668	-0.84074	-1.06768	-0.99813	-0.89833	6.50E-17	6.03E-12	4.39E-15	1.51E-13	1.48E-13	628	1465	1053	1281	1265
CD247	-1.35575	-1.21449	-1.43472	-1.5122	-1.69895	6.52E-17	4.56E-14	1.40E-15	3.80E-15	1.61E-14	629	961	964	950	1028
GALE	2.178137	2.160106	2.403002	2.341865	1.953686	6.77E-17	9.64E-18	1.21E-19	3.50E-19	1.18E-13	630	430	400	406	1240
MMP12	-4.66639	-5.13978	-4.68741	-5.2486	-5.2003	6.87E-17	7.96E-20	7.66E-18	3.67E-18	1.48E-17	631	256	611	512	521
ETHE1	2.211269	1.959302	2.516502	1.997405	2.020958	6.88E-17	1.15E-15	1.54E-17	2.49E-15	3.62E-16	632	708	654	916	733
MRGPRE	-1.20521	-1.15191	-1.15634	-1.25984	-1.02495	6.90E-17	2.68E-17	2.30E-16	2.83E-17	9.21E-14	633	487	831	622	1211
ADAM12	-1.95243	-2.05476	-1.85116	-2.07393	-1.90169	6.93E-17	4.04E-14	3.66E-12	1.63E-13	1.39E-15	634	951	1747	1292	828
HTRA3	-3.47932	-3.50033	-3.36542	-3.53875	-3.78631	7.10E-17	3.42E-19	5.68E-18	1.14E-24	2.13E-20	635	299	596	90	233
AGT	-2.88213	-2.76353	-2.76213	-2.88018	-2.72235	7.14E-17	1.11E-15	2.20E-17	7.78E-15	5.85E-16	636	705	674	1011	763
TCN2	-1.73765	-1.37299	-1.45703	-1.57565	-1.70757	7.36E-17	1.65E-11	8.77E-13	9.87E-13	2.03E-10	637	1588	1572	1500	2157
MRGPRF	-2.49464	-1.91429	-2.08263	-2.08712	-2.37435	7.47E-17	2.94E-12	9.20E-16	4.87E-15	1.13E-20	638	1364	927	979	219
SSBP1	1.730049	1.844941	1.706879	1.848447	1.793577	7.54E-17	6.64E-19	1.21E-16	5.01E-23	1.01E-17	639	325	779	156	501
CD46	2.272164	1.965554	2.235856	2.397295	2.143718	7.56E-17	6.48E-14	5.94E-14	4.79E-17	4.74E-13	640	999	1262	657	1387
HIP1	-1.76596	-1.39072	-1.83637	-1.57585	-1.60578	7.57E-17	4.16E-14	3.16E-18	2.55E-16	1.01E-15	641	952	550	764	806
FGD5	-2.49985	-2.51705	-2.27328	-2.53276	-2.36916	7.66E-17	1.65E-18	9.62E-18	1.02E-21	2.40E-16	642	349	625	225	710
EID1	-2.18811	-2.36245	-2.18791	-2.23452	-2.25396	8.10E-17	3.17E-20	6.39E-18	3.57E-19	7.30E-19	643	222	605	407	366
PDZRN3	-1.68842	-1.3287	-1.4449	-1.35113	-1.08557	8.35E-17	8.62E-12	8.41E-13	1.97E-10	1.32E-06	644	1512	1568	2147	3798
AGR2	4.376085	3.699758	4.866237	3.77119	3.822572	8.35E-17	9.64E-18	1.74E-20	7.46E-18	1.16E-15	645	429	342	558	813
UQCRC2	1.797637	1.396047	1.724504	1.728668	1.650453	8.41E-17	7.23E-15	1.42E-14	8.63E-17	5.41E-17	646	815	1140	692	598
BACE1	-1.89995	-1.54623	-1.63392	-1.49353	-1.56653	8.41E-17	6.00E-11	1.06E-12	1.83E-10	5.61E-13	647	1768	1604	2135	1409
APBB3	-1.44948	-1.36493	-1.72199	-1.49042	-1.33793	8.60E-17	5.21E-15	1.78E-18	8.33E-13	2.76E-10	648	788	517	1478	2216
IGHG1	-6.95047	-7.93973	-7.80433	-7.69265	-7.3068	8.60E-17	4.16E-20	5.39E-23	1.46E-18	3.06E-19	649	230	164	471	328
DAPK1	-1.90734	-1.73388	-1.87217	-1.94936	-1.72189	8.60E-17	1.94E-13	2.53E-14	1.96E-18	5.84E-13	650	1100	1184	489	1417
AFAP1L1	-1.83731	-1.45915	-1.75352	-1.58045	-1.57521	8.78E-17	5.74E-14	4.97E-21	6.23E-15	2.02E-15	651	989	301	996	850
TFRC	2.122475	2.232015	2.452052	2.027368	1.836376	8.87E-17	4.63E-17	6.04E-21	7.63E-14	4.81E-13	652	524	313	1216	1389
COX7C	1.659545	1.558717	1.714281	1.641959	1.663548	8.91E-17	2.56E-18	2.27E-17	1.17E-19	1.29E-15	653	372	675	358	823
TMBIM6	2.078879	1.536879	1.956091	1.77296	1.803859	8.98E-17	1.30E-12	1.69E-15	2.26E-13	1.46E-18	654	1276	977	1326	396
HLA-DRB1	-4.05416	-4.28437	-3.36427	-3.80395	-4.15079	9.01E-17	1.25E-20	1.09E-13	1.19E-15	1.49E-16	655	199	1328	859	670
CD40	-2.19052	-2.17424	-2.3421	-1.78884	-2.26868	9.24E-17	6.52E-12	4.83E-15	2.67E-13	3.35E-11	656	1472	1062	1347	1909

PCNX1	-1.5452	-1.24231	-1.34832	-1.28277	-1.51878	9.25E-17	6.74E-14	1.57E-15	1.06E-13	1.87E-16	657	1004	972	1247	682
S100A10	3.141105	2.871014	3.227819	2.811329	2.715142	9.40E-17	3.41E-19	3.75E-18	3.46E-18	2.69E-19	658	296	569	508	323
EFEMP1	-3.02957	-2.61968	-2.43056	-2.84094	-2.66999	9.62E-17	9.22E-16	1.56E-15	2.44E-17	2.78E-15	659	692	970	616	867
H2BC8	2.814278	2.399025	2.837223	2.677032	2.845095	9.89E-17	3.27E-15	8.65E-19	2.05E-16	6.38E-21	660	761	478	743	198
VDAC1	1.659135	1.43851	1.806811	1.638962	1.700455	1.01E-16	5.60E-15	4.33E-18	1.65E-16	4.98E-14	661	794	576	736	1152
PATJ	1.739263	2.015634	1.954571	2.04993	1.988	1.04E-16	2.01E-16	7.55E-18	3.57E-19	1.80E-18	662	608	610	408	401
IL7R	-3.54837	-3.24743	-3.43482	-3.78151	-3.49439	1.04E-16	4.48E-13	5.15E-17	1.90E-14	6.07E-13	663	1188	715	1091	1426
SCARF2	-2.44055	-2.08545	-2.21824	-2.3023	-2.64018	1.05E-16	1.46E-15	7.06E-16	3.74E-19	8.98E-19	664	724	904	415	375
EHD3	-1.91823	-1.75963	-1.85008	-1.95875	-2.01682	1.11E-16	6.92E-15	1.17E-14	3.63E-17	8.06E-15	665	813	1130	648	966
ITGA1	-3.80267	-3.93843	-3.24363	-3.86767	-3.32328	1.12E-16	1.61E-20	9.04E-17	4.45E-19	3.23E-13	666	208	757	425	1344
LDLRAD4	-1.32963	-0.96164	-1.24152	-1.15906	-1.0636	1.13E-16	1.49E-07	4.00E-10	1.41E-08	8.05E-08	667	3085	2432	2820	3230
KLHL6	-1.65633	-1.72748	-2.33376	-2.08579	-2.00376	1.14E-16	5.15E-18	3.59E-18	2.41E-13	1.31E-12	668	403	562	1337	1515
ATP10B	3.096056	3.52356	3.581577	3.582717	2.874889	1.20E-16	3.71E-15	1.37E-17	8.38E-16	4.76E-13	669	769	648	837	1388
IFI30	-3.61797	-4.27604	-3.907	-4.24938	-4.22411	1.22E-16	1.97E-21	2.15E-21	7.98E-22	5.50E-21	670	147	260	220	191
APOE	-3.26648	-3.66689	-3.42974	-3.38906	-3.79801	1.25E-16	3.37E-19	2.87E-19	9.41E-20	7.09E-19	671	293	435	349	360
PPDPF	2.822403	2.273664	3.011635	2.697687	2.670543	1.26E-16	4.81E-16	2.84E-20	2.82E-15	6.30E-21	672	651	356	921	196
HOMER3	-1.68367	-1.77402	-2.07564	-1.89469	-2.00021	1.36E-16	2.97E-14	8.91E-19	5.63E-16	4.06E-17	673	930	482	810	577
SCD5	-1.20723	-1.00003	-1.18649	-1.21972	-1.18028	1.48E-16	8.87E-13	8.14E-14	7.91E-16	2.44E-15	674	1245	1299	830	861
ENSA	1.071965	0.964815	0.842654	0.900418	0.889525	1.49E-16	1.15E-13	1.31E-13	1.22E-12	1.99E-13	675	1047	1357	1526	1294
SENP7	-1.3293	-1.29594	-1.319	-1.27876	-1.23201	1.54E-16	2.28E-17	2.00E-15	1.57E-14	1.01E-14	676	475	992	1071	982
PLS1	2.359861	2.355282	2.649268	2.44383	2.490251	1.58E-16	1.23E-16	2.57E-22	9.57E-21	6.70E-20	677	584	197	289	275
UBE2I	1.501449	1.368947	1.441686	1.473664	1.224353	1.58E-16	2.06E-14	1.17E-19	2.30E-16	1.34E-12	678	889	397	755	1518
ALOX5AP	-2.75093	-2.34082	-2.26434	-2.23542	-2.24073	1.61E-16	1.05E-14	1.65E-14	2.49E-18	9.51E-12	679	839	1149	501	1755
CSF1	-1.60433	-1.54454	-1.32568	-1.54704	-1.32339	1.61E-16	2.28E-17	2.49E-15	3.47E-17	8.52E-16	680	476	1009	641	797
EIF3H	1.791501	1.849158	2.155048	1.913611	1.909978	1.62E-16	8.86E-21	2.41E-17	7.78E-19	3.88E-19	681	184	678	445	333
DCHS1	-2.24526	-2.03313	-2.26312	-2.20289	-1.93963	1.65E-16	6.28E-18	6.18E-22	3.03E-20	1.53E-17	682	411	224	315	525
RGS10	-1.98255	-2.07429	-2.25094	-2.22427	-2.22	1.68E-16	4.63E-14	1.32E-18	3.62E-16	1.39E-20	683	962	501	781	220
COL14A1	-3.00386	-2.69206	-2.26834	-2.66987	-2.61631	1.68E-16	4.03E-19	2.32E-16	2.83E-18	4.22E-15	684	304	832	507	906
C5AR1	-1.4985	-1.27522	-1.58407	-1.38562	-1.58021	1.69E-16	4.42E-12	6.61E-17	1.64E-13	8.45E-18	685	1418	732	1294	488
BMP1	-3.12127	-2.95694	-2.92776	-3.04126	-2.73343	1.72E-16	1.40E-15	3.94E-19	1.78E-19	1.51E-14	686	721	449	382	1024
GYPC	-2.14576	-2.37268	-2.46577	-2.36935	-2.41143	1.80E-16	2.19E-18	8.34E-22	6.04E-16	4.15E-20	687	363	229	817	255
CXADR	2.633752	2.710989	2.505558	2.619095	2.595013	1.81E-16	3.62E-18	1.93E-18	3.53E-17	7.23E-19	688	387	523	643	362
CPXM1	-2.52224	-2.83792	-2.68184	-2.93125	-2.81477	1.84E-16	2.22E-16	1.05E-16	1.41E-16	8.42E-18	689	612	773	723	487
EMILIN2	-2.61883	-2.5087	-2.83144	-2.58462	-2.73989	1.90E-16	2.14E-14	6.23E-17	9.72E-15	1.81E-14	690	893	727	1030	1045
ADAM28	-1.81218	-1.58592	-2.24422	-1.85601	-1.7184	1.96E-16	3.58E-10	1.05E-13	2.65E-12	5.12E-09	691	2024	1321	1611	2684
EIF1AX	1.533712	1.542716	1.496211	1.82078	1.577555	2.02E-16	5.90E-14	1.45E-12	2.69E-17	2.97E-14	692	992	1635	619	1099
NRP2	-2.26961	-2.08184	-2.60554	-2.24314	-2.50902	2.04E-16	4.56E-13	2.36E-16	5.08E-15	1.16E-14	693	1191	833	985	1003
TMEM140	-1.77083	-1.56481	-1.77217	-1.86799	-1.71293	2.08E-16	1.03E-13	2.82E-16	2.16E-17	4.28E-14	694	1038	844	611	1143
TMEM244	-0.89451	-0.68492	-0.89725	-0.8102	-0.91278	2.08E-16	5.81E-12	6.23E-14	4.24E-13	7.32E-15	695	1459	1267	1404	954
PHF21A	-1.62297	-1.39063	-1.36883	-1.43749	-1.23846	2.11E-16	2.87E-15	2.30E-16	4.58E-16	2.45E-09	696	754	830	795	2581
COL15A1	-4.09025	-3.92805	-3.5106	-3.83903	-4.09397	2.11E-16	3.85E-16	7.40E-16	6.48E-18	5.18E-18	697	635	911	553	457
CYB5B	1.935884	2.137725	2.139923	2.495553	2.402368	2.12E-16	4.90E-18	1.03E-18	1.53E-18	6.25E-20	698	400	492	476	270
DUS1L	1.776759	1.750545	1.661407	1.861277	1.516649	2.13E-16	4.27E-16	8.20E-15	6.09E-19	6.69E-15	699	640	1107	437	946
CD2AP	1.276981	1.132279	1.680317	1.257955	1.530582	2.13E-16	1.65E-11	2.09E-17	1.42E-12	1.15E-17	700	1587	668	1547	509
C1QTNF5	-3.35558	-3.50381	-3.30285	-3.33092	-3.78315	2.17E-16	6.77E-18	4.77E-20	1.83E-21	1.55E-18	701	412	373	245	397
GNG11	-3.09662	-2.89148	-3.08218	-2.83313	-3.17834	2.18E-16	3.31E-13	8.75E-17	4.02E-15	4.88E-16	702	1151	756	960	753
SLC44A4	2.724186	2.382389	3.072693	2.61473	3.01246	2.21E-16	3.40E-14	1.04E-20	2.07E-17	1.36E-17	703	937	325	605	514
MAP3K3	-1.68735	-1.50848	-1.48594	-1.49463	-1.35539	2.22E-16	1.20E-15	2.07E-15	5.79E-15	4.76E-14	704	710	995	993	1148
CNRIP1	-1.45956	-1.17881	-1.2693	-1.31513	-1.37364	2.26E-16	4.50E-13	1.87E-13	9.12E-16	1.71E-15	705	1189	1409	839	842
TYMP	-2.96789	-3.03147	-2.8178	-2.88936	-3.10765	2.30E-16	5.16E-16	7.57E-16	3.65E-19	5.30E-18	706	661	913	411	458
EML3	-1.26186	-0.90647	-1.18888	-0.94768	-1.15817	2.41E-16	8.90E-12	3.40E-18	1.26E-12	3.40E-14	707	1517	557	1530	1114
CD74	-4.10929	-4.61353	-3.68367	-3.82575	-4.44139	2.46E-16	4.89E-19	6.45E-12	6.63E-15	6.08E-17	708	313	1810	1000	608
RGS5	-4.19683	-4.35378	-4.11161	-4.82369	-4.34563	2.47E-16	1.61E-17	4.50E-18	1.44E-20	6.65E-18	709	455	579	300	471
VAPB	1.366932	1.255487	1.125086	1.340902	1.005148	2.51E-16	5.71E-16	6.66E-16	4.38E-16	1.32E-11	710	667	896	792	1803
PPA1	2.300434	2.019174	2.365723	2.417155	2.598018	2.53E-16	4.29E-16	6.08E-18	3.66E-15	4.18E-19	711	642	599	946	336
BTBD19	-2.17105	-1.76205	-1.85001	-1.93334	-2.11268	2.58E-16	2.80E-12	4.41E-13	3.54E-16	1.43E-15	712	1361	1500	779	831
VSIG4	-2.62084	-2.51758	-2.23358	-2.27059	-2.54674	2.61E-16	1.78E-17	2.93E-12	2.52E-17	9.82E-13	713	463	1717	618	1482
ANXA6	-3.13208	-2.92124	-2.64974	-2.57454	-2.50338	2.63E-16	7.89E-19	6.99E-15	9.81E-15	8.92E-13	714	331	1093	1033	1468
SPAG11A	-1.14698	-0.81229	-1.22022	-0.9313	-1.14971	2.65E-16	9.79E-08	1.28E-10	2.91E-07	8.85E-12	715	2991	2257	3369	1747
ATP8B2	-1.76771	-1.75818	-2.11379	-2.12557	-2.1125	2.66E-16	2.72E-17	3.14E-15	8.09E-16	3.30E-15	716	488	1026	835	879
DYSF	-2.51603	-2.52236	-2.68107	-2.67022	-2.7456	2.70E-16	8.00E-17	1.53E-15	1.74E-19	3.04E-16	717	554	969	381	726
FZD5	1.849741	1.971822	1.991462	1.869278	1.74511	2.75E-16	2.31E-14	4.47E-15	7.92E-16	2.72E-16	718	907	1055	832	716
C16orf86	-1.04761	-0.91864	-0.89896	-0.9852	-1.08521	2.77E-16	4.23E-14	1.97E-11	6.21E-13	6.89E-12	719	956	1972	1448	1712
PRRX1	-2.86489	-2.67272	-2.51768	-2.68859	-3.00217	2.85E-16	2.56E-12	1.00E-13	5.46E-14	1.19E-18	720	1344	1315	1184	387
NDUFA1	1.875933	1.789886	2.029542	1.994824	1.381979	2.89E-16	9.23E-16	1.23E-16	1.25E-16	1.12E-10	721	693	780	715	2069
PODXL	-1.94836	-1.98196	-1.65938	-1.84281	-1.83104	2.91E-16	3.84E-16	1.84E-14	3.76E-16	1.12E-13	722	634	1159	784	1232
SH3PXD2B	-1.83342	-1.78861	-1.74959	-1.58853	-1.7322	3.04E-16	5.71E-14	4.92E-18	1.02E-12	5.66E-14	723	988	586	1506	1169
SLIRP	1.605028	1.854092	1.917977	1.876167	2.081355	3.05E-16	1.71E-17	5.67E-15	2.42E-19	2.74E-18	724	458	1079	392	420
RCAN1	-1.22906	-0.97148	-1.04681	-1.01995	-1.10318	3.06E-16	1.20E-09	1.56E-15	2.90E-14	5.24E-13	725	2198	971	1127	1401
EBNA1BP2	1.378699	1.71825	1.601537	1.644394	1.955689	3.07E-16	4.05E-18	2.84E-16	1.14E-19	9.72E-13	726	390	846	356	1479
TMPRSS2	2.771984	3.292963	3.569268	3.124752	3.385023	3.07E-16	1.05E-17	1.04E-20	5.66E-18	3.72E-25	727	435	326	543	56
CDH13	-2.15003	-1.8741	-2.31462	-1.91058	-2.2693	3.16E-16	1.28E-09	1.03E-15	9.20E-14	1.10E-13	728	2212	939	1238	1229
SP140	-1.37234	-1.15005	-1.57344	-1.53534	-1.59577	3.20E-16	7.98E-14	1.66E-14	3.07E-13	3.16E-13	729	1019	1150	1363	1340

CD14	-3.27532	-3.70549	-3.65849	-3.6596	-3.39592	3.21E-16	1.87E-18	2.85E-22	6.38E-20	4.56E-15	730	357	200	333	914
GIPC1	2.013019	1.954309	2.455377	1.988202	2.00995	3.27E-16	3.44E-18	2.08E-22	7.22E-19	7.67E-16	731	383	188	441	785
CHST1	-1.5814	-1.41311	-1.6842	-1.54521	-1.64757	3.33E-16	4.36E-13	2.57E-18	7.28E-15	3.98E-16	732	1181	537	1005	739
CAMSAP3	1.938123	2.321669	2.372375	2.290827	1.995569	3.34E-16	2.37E-17	5.39E-20	4.40E-19	4.71E-15	733	479	376	424	917
NDUFAB1	1.617327	1.406406	1.624091	1.541807	1.346121	3.46E-16	7.83E-17	1.54E-17	4.67E-17	2.83E-13	734	553	655	656	1329
PRR15	2.46058	2.563365	2.509577	2.594055	2.477923	3.61E-16	1.75E-17	9.74E-15	4.28E-19	5.24E-17	735	462	1115	420	595
AXIN2	2.735655	2.890572	2.585988	3.02891	3.003379	3.76E-16	1.77E-20	5.18E-18	6.22E-19	7.36E-18	736	210	591	438	477
HLA-DRA	-4.28868	-4.70452	-3.54442	-3.93754	-4.52004	3.77E-16	1.10E-18	2.04E-11	7.68E-14	1.09E-15	737	338	1980	1218	811
P2RY8	-1.28098	-1.21821	-1.58865	-1.4943	-1.59671	3.77E-16	5.71E-16	8.47E-14	1.85E-13	6.58E-14	738	668	1305	1307	1184
BCAP31	2.135584	1.819975	2.036769	2.172462	1.647758	3.83E-16	3.12E-15	7.57E-17	5.29E-18	7.62E-16	739	757	748	538	784
TMEM204	-1.57138	-1.39196	-1.69709	-1.6068	-1.72534	3.87E-16	1.62E-13	2.72E-15	1.53E-17	3.50E-16	740	1073	1013	593	730
PPP1R14A	-1.89976	-2.01243	-1.76824	-2.11586	-1.39498	3.92E-16	1.87E-13	2.97E-15	9.18E-16	4.99E-09	741	1095	1016	840	2683
GUCY2C	2.047591	2.047041	2.207122	2.187421	2.356113	3.97E-16	2.37E-18	4.57E-18	2.72E-17	2.26E-21	742	367	581	620	169
ANGPTL2	-3.90069	-3.49651	-3.55736	-3.61235	-4.27699	4.11E-16	2.05E-12	2.02E-15	7.39E-15	1.41E-19	743	1326	993	1006	305
CLEC11A	-2.17431	-1.83194	-1.8662	-1.8549	-2.14652	4.19E-16	4.11E-13	2.59E-15	1.56E-14	1.01E-15	744	1176	1010	1070	807
TOM1L1	2.077005	1.834665	2.236396	1.879843	1.919125	4.22E-16	1.65E-13	3.37E-20	1.93E-16	5.30E-16	745	1078	365	739	758
FDFT1	2.014018	2.166007	2.496524	2.350738	2.02062	4.28E-16	1.10E-16	4.18E-19	1.60E-16	1.26E-15	746	580	452	732	821
TLE4	-1.9605	-1.86316	-1.96388	-1.74841	-1.75308	4.38E-16	1.32E-13	2.14E-17	2.75E-14	5.88E-11	747	1058	670	1119	1969
DERL3	-3.07995	-3.01692	-3.64895	-3.43424	-3.82213	4.47E-16	2.37E-15	1.51E-16	1.52E-11	1.04E-16	748	748	799	1829	649
CYTH4	-1.76563	-1.76988	-1.88685	-2.16299	-2.02616	4.47E-16	1.33E-13	2.71E-15	2.10E-16	7.84E-17	749	1061	1012	747	622
COX7A1	-2.49406	-2.45188	-2.61667	-2.58607	-2.75462	4.60E-16	7.50E-16	1.45E-18	7.39E-20	1.02E-19	750	678	507	341	296
F2RL3	-2.02624	-1.51483	-1.594	-1.62132	-1.96089	5.00E-16	6.02E-10	3.50E-13	3.92E-12	3.50E-18	751	2099	1472	1653	431
RPS6KA2	-1.72821	-1.63451	-1.71048	-1.73298	-1.45377	5.04E-16	4.73E-15	2.20E-16	9.33E-15	9.33E-12	752	781	824	1024	1752
TNS3	-2.18564	-2.22753	-1.84656	-2.22108	-1.79005	5.07E-16	9.07E-16	6.96E-14	3.07E-17	6.74E-12	753	691	1278	626	1710
CISD3	1.979543	1.754579	2.017911	1.770863	1.738055	5.08E-16	8.92E-16	5.48E-15	1.57E-15	2.10E-17	754	689	1077	876	541
ITGB7	-1.97274	-1.52998	-1.95366	-1.75265	-2.03752	5.11E-16	3.85E-13	2.29E-15	4.00E-18	1.04E-14	755	1168	998	516	988
NDUFS6	1.814917	1.624769	1.898041	1.763314	1.885836	5.15E-16	4.33E-13	3.14E-15	4.57E-14	4.96E-18	756	1179	1024	1169	454
SLC7A1	1.964809	1.958916	2.22505	1.842178	1.84322	5.17E-16	7.85E-16	2.01E-22	8.03E-16	6.42E-12	757	683	186	834	1704
ABI3	-1.50014	-1.46376	-1.53147	-1.52881	-1.20555	5.19E-16	3.37E-15	1.67E-14	2.17E-15	3.30E-08	758	764	1151	908	3037
FN1	-4.43809	-4.56482	-4.45867	-4.15019	-4.44909	5.19E-16	1.05E-15	2.34E-15	2.83E-16	4.15E-12	759	701	1001	772	1652
STARD9	-1.33304	-1.24542	-1.17686	-1.38952	-1.2733	5.22E-16	1.11E-13	1.09E-12	6.04E-16	2.17E-13	760	1044	1608	816	1306
NDUFB3	1.478617	1.523492	1.574544	1.646225	1.506768	5.22E-16	2.24E-17	1.49E-20	9.05E-18	6.17E-21	761	474	335	570	195
CS	1.673629	1.589872	1.756846	1.733258	1.757419	5.23E-16	2.38E-12	5.09E-14	3.50E-13	9.47E-17	762	1336	1249	1380	638
ELK3	-1.66812	-1.41798	-1.60022	-1.45794	-1.63512	5.23E-16	5.29E-14	1.64E-18	1.73E-14	3.18E-19	763	978	514	1079	330
CTSO	-1.54615	-1.49001	-1.22705	-1.56683	-1.2565	5.30E-16	6.98E-12	1.42E-10	1.60E-11	2.36E-11	764	1483	2270	1834	1873
FAM20A	-1.73957	-1.5827	-1.85107	-1.82605	-1.4748	5.34E-16	6.09E-17	2.39E-13	2.24E-16	5.76E-10	765	535	1439	750	2330
ITGA11	-3.28669	-2.72056	-2.92653	-2.82054	-3.60537	5.37E-16	5.59E-13	1.62E-17	9.80E-15	7.57E-16	766	1206	658	1031	783
CKMT1A	2.1507	2.355921	2.281546	2.096096	2.341947	5.48E-16	3.40E-18	1.80E-11	1.67E-12	2.90E-15	767	382	1950	1567	869
FZD4	-2.51793	-2.37045	-2.46635	-2.51105	-2.28718	5.48E-16	1.19E-17	4.70E-21	9.67E-20	2.59E-17	768	443	296	350	551
SMAGP	1.645261	1.730011	1.354013	1.824211	1.569675	5.60E-16	1.14E-15	7.52E-17	6.31E-18	9.61E-14	769	706	747	551	1218
S100A8	-1.61688	-1.54721	-2.18291	-1.62572	-2.02869	5.60E-16	4.97E-12	8.60E-17	1.57E-14	1.18E-13	770	1437	753	1072	1239
TSPAN6	1.951909	1.913111	1.797426	2.0705	1.508267	5.82E-16	3.42E-15	4.90E-15	1.11E-13	3.35E-10	771	765	1064	1251	2236
SYAP1	1.120769	1.078668	1.17925	1.137422	0.791226	5.82E-16	2.09E-15	6.31E-16	7.40E-12	8.09E-06	772	740	888	1715	4224
GM2A	-1.70341	-1.77707	-1.63292	-1.71028	-1.74229	5.89E-16	4.67E-18	8.36E-15	4.17E-13	2.09E-13	773	397	1110	1402	1301
GABARAPI	-2.07528	-1.87217	-1.87558	-1.85786	-2.10627	5.93E-16	3.23E-16	2.07E-18	1.15E-16	1.92E-16	774	627	527	706	687
DGAT1	2.258191	2.350391	2.426222	2.3561	1.97301	6.02E-16	2.35E-18	1.09E-15	3.74E-19	1.36E-14	775	366	942	414	1019
KRT20	3.30785	3.121136	3.538198	3.122287	3.292392	6.24E-16	3.69E-15	1.01E-16	4.40E-19	8.91E-18	776	767	770	422	490
SLC1A5	1.738401	1.714927	1.47196	1.814085	1.833456	6.34E-16	2.71E-16	7.26E-17	1.34E-13	1.29E-11	777	617	743	1267	1802
IRF4	-2.21039	-1.73146	-2.81571	-2.50205	-2.59489	6.34E-16	4.05E-12	4.14E-15	4.38E-13	1.51E-11	778	1408	1048	1407	1816
AMOTL1	-2.6107	-2.3202	-2.34409	-2.56673	-2.5336	6.68E-16	1.94E-18	3.48E-17	8.93E-20	1.39E-18	779	358	693	347	393
MAGED4	-1.73922	-1.50653	-1.66526	-1.64542	-1.48971	6.80E-16	2.33E-11	8.85E-13	5.67E-14	3.06E-12	780	1635	1574	1188	1616
RHOQ	-1.70557	-1.86532	-2.08219	-1.93977	-1.95183	6.84E-16	5.74E-17	2.87E-20	2.30E-16	4.56E-17	781	532	357	754	583
PLXDC2	-3.37689	-3.4943	-3.24882	-3.22814	-3.66369	6.84E-16	1.01E-19	1.37E-16	3.74E-18	4.03E-19	782	266	791	513	335
PIK3IP1	-1.29243	-0.96331	-1.01528	-0.90043	-1.14569	7.04E-16	1.19E-08	3.83E-08	5.86E-09	3.34E-09	783	2585	3321	2679	2621
GLT8D2	-2.24117	-1.54015	-1.72359	-1.64866	-1.85005	7.17E-16	4.10E-10	5.55E-12	3.47E-11	3.50E-13	784	2042	1797	1928	1354
NDUFA4L2	-2.28838	-2.60212	-2.56224	-2.78417	-2.47656	7.77E-16	2.69E-12	1.98E-17	2.62E-18	1.77E-14	785	1355	665	505	1042
MKI67	1.830787	1.770327	1.87622	1.727862	1.549227	8.19E-16	2.76E-13	8.08E-18	8.70E-15	1.29E-12	786	1123	613	1016	1512
SEM1	1.732235	1.526644	1.696014	1.397123	1.840164	8.33E-16	3.56E-12	6.57E-16	8.55E-12	5.69E-17	787	1387	894	1736	600
CYTIP	-2.49207	-2.19781	-3.31874	-2.46578	-2.50061	8.37E-16	5.36E-13	5.28E-17	1.41E-09	1.42E-09	788	1200	718	2460	2484
TMEM45B	2.894026	2.688563	3.247739	2.826608	2.617816	8.40E-16	2.13E-15	2.17E-19	1.37E-16	1.99E-12	789	742	425	722	1554
CENPX	1.586016	1.714488	1.762488	1.714243	1.679496	8.58E-16	6.00E-17	1.41E-17	8.03E-18	5.56E-18	790	534	650	563	460
BMPR2	-1.79051	-1.69773	-1.46482	-1.52198	-1.44383	8.67E-16	9.60E-17	4.83E-14	1.75E-14	2.72E-11	791	570	1237	1082	1885
ANO9	2.544469	2.719455	2.412346	2.887699	2.427744	8.68E-16	4.96E-16	1.70E-14	2.83E-17	2.34E-17	792	654	1153	621	546
NUDT18	-1.03731	-0.71043	-1.13254	-1.00939	-1.20743	8.87E-16	4.59E-08	6.79E-17	8.53E-12	1.07E-13	793	2829	737	1735	1226
RAB12	-1.54194	-1.25202	-0.99393	-1.2469	-1.49346	9.00E-16	1.44E-13	5.49E-12	6.30E-13	2.18E-16	794	1065	1796	1450	700
GBP4	-1.49284	-1.38346	-1.55154	-1.84022	-1.66079	9.03E-16	3.00E-13	1.24E-10	7.93E-17	1.33E-09	795	1132	2248	686	2473
HSPE1	2.457966	2.621589	2.481629	2.731368	2.388393	9.04E-16	1.14E-17	6.33E-16	2.74E-20	8.21E-19	796	440	889	313	370
MEIS1	-2.15878	-1.85549	-1.94438	-2.11232	-2.27615	9.33E-16	3.38E-12	3.62E-15	3.97E-14	1.65E-14	797	1383	1039	1161	1033
RABL6	1.216681	1.338629	1.402582	1.167645	1.505488	9.33E-16	2.09E-14	3.38E-19	9.00E-14	3.03E-18	798	891	441	1236	423
ITPKB	-1.45806	-1.25886	-1.43849	-1.213	-1.16783	9.33E-16	5.82E-14	6.62E-16	7.41E-14	6.33E-11	799	990	895	1210	1987
CSPG4	-2.86711	-2.96876	-3.02814	-3.10891	-3.37885	9.37E-16	6.06E-18	5.77E-19	4.82E-23	1.81E-19	800	409	466	154	312
CD163L1	-2.02997	-1.91173	-2.09436	-2.02849	-1.84383	9.41E-16	1.44E-15	4.41E-15	1.53E-15	1.06E-12	801	722	1054	873	1493
MYO7B	2.278958	2.208661	2.610342	2.288978	2.139361	9.49E-16	4.91E-14	1.95E-19	9.21E-15	2.02E-17	802	967	422	1023	538

MGAT4B	1.956295	1.95584	1.873645	1.797246	2.051376	9.55E-16	3.50E-17	8.63E-18	3.37E-15	2.71E-25	803	505	620	938	51
PLEK	-1.64742	-1.65985	-1.79878	-1.79334	-1.70493	9.77E-16	1.76E-14	2.03E-17	1.73E-13	1.02E-13	804	880	667	1299	1221
ELOB	1.566278	1.370155	1.612542	1.639708	1.430642	9.77E-16	3.61E-13	4.58E-14	1.42E-14	9.93E-14	805	1163	1234	1065	1220
PTPRE	-2.151	-2.22858	-2.0669	-2.37541	-1.98103	9.85E-16	1.68E-17	4.69E-18	4.26E-19	2.16E-12	806	456	582	419	1566
IFITM10	-1.34845	-1.03691	-1.21861	-1.09902	-1.40775	9.90E-16	1.70E-13	1.22E-15	2.22E-16	1.59E-13	807	1085	954	749	1272
OLR1	-1.56092	-1.27081	-1.33792	-1.42541	-1.61968	1.00E-15	8.54E-13	5.44E-13	3.03E-13	8.81E-15	808	1240	1524	1360	973
PKN1	-1.47802	-1.28738	-1.37205	-1.28898	-1.13702	1.01E-15	2.68E-11	1.35E-13	2.70E-12	5.18E-09	809	1660	1362	1613	2687
HOOK2	1.343528	1.556179	1.474403	1.623199	1.359437	1.06E-15	7.21E-18	4.19E-19	6.14E-18	1.15E-18	810	416	453	550	384
CLIP3	-2.00302	-1.65041	-1.65772	-1.91499	-1.84347	1.07E-15	4.47E-12	1.24E-14	1.04E-14	7.55E-15	811	1420	1131	1037	960
SYNPO2	-1.7969	-1.39088	-1.79965	-1.54999	-1.42831	1.08E-15	8.58E-14	9.45E-18	3.51E-13	4.22E-16	812	1024	623	1381	742
SLC39A13	-1.47937	-1.35494	-1.51061	-1.26292	-1.49267	1.09E-15	9.51E-14	5.98E-18	4.75E-15	3.29E-17	813	1030	597	973	564
SEMA6B	-2.30503	-2.0023	-2.27371	-1.98937	-2.28404	1.09E-15	7.90E-12	1.83E-15	9.45E-13	3.07E-14	814	1506	988	1491	1103
UBE2Q2L	-1.25682	-0.99483	-0.99355	-1.31166	-1.16044	1.13E-15	3.96E-11	6.81E-12	8.49E-19	6.24E-11	815	1713	1823	449	1980
F2RL2	-2.87365	-2.88815	-2.62479	-3.19657	-2.65878	1.13E-15	7.65E-16	1.05E-15	4.53E-19	2.78E-16	816	679	940	428	719
ODC1	2.144447	1.999616	1.738038	1.973488	1.563457	1.18E-15	2.77E-16	2.53E-13	2.46E-16	1.07E-11	817	621	1446	762	1775
ADCY7	-1.24994	-1.07675	-1.41078	-1.44078	-1.17682	1.18E-15	6.92E-12	3.01E-12	2.16E-11	1.25E-05	818	1481	1722	1871	4349
KIRREL1	-2.31077	-2.27004	-2.13339	-2.49516	-2.24789	1.18E-15	8.77E-16	6.78E-16	5.03E-18	9.08E-17	819	687	897	532	632
DIO2	-2.92252	-2.39087	-2.38162	-2.76865	-2.57257	1.20E-15	1.08E-12	2.21E-18	5.24E-16	1.94E-15	820	1258	533	802	848
SNRPN	-2.16601	-1.7001	-1.62656	-1.56111	-1.14655	1.20E-15	1.31E-13	2.08E-14	1.30E-09	1.59E-05	821	1057	1168	2435	4411
MET	2.425002	2.868636	2.706282	2.912642	2.464915	1.23E-15	3.13E-20	1.61E-17	3.07E-17	4.98E-14	822	220	657	628	1151
EMID1	-2.34115	-2.07062	-2.09594	-2.04469	-2.02333	1.27E-15	4.75E-16	4.42E-18	5.37E-18	1.71E-12	823	648	577	540	1538
DUX5	-1.09331	-0.97962	-1.01901	-0.85559	-1.00734	1.28E-15	1.15E-13	2.50E-17	3.18E-13	2.61E-12	824	1046	682	1368	1595
HTATIP2	1.516295	1.640166	1.671506	1.507385	1.713329	1.30E-15	5.05E-16	1.11E-17	1.98E-17	8.62E-15	825	657	637	601	971
PYHIN1	-1.35189	-1.27352	-1.54473	-1.48983	-1.44879	1.31E-15	9.22E-14	5.77E-13	3.01E-14	2.67E-08	826	1027	1531	1134	2994
CTSC	-3.12199	-3.32032	-2.95223	-3.11053	-2.99691	1.32E-15	4.80E-16	2.34E-15	4.50E-16	6.49E-18	827	650	1002	794	469
PDHA1	1.603175	1.776208	1.629671	1.764576	1.457007	1.33E-15	6.74E-15	1.16E-16	1.14E-14	5.59E-15	828	809	777	1047	930
LMCD1	-2.35996	-2.44868	-2.65215	-2.63418	-2.82132	1.36E-15	8.31E-13	4.67E-19	5.28E-16	1.46E-17	829	1234	461	803	520
APOBEC3(	-1.84651	-1.63764	-1.86425	-1.80542	-2.00953	1.36E-15	8.81E-14	2.24E-19	8.01E-15	9.76E-21	830	1025	426	1012	215
UQCRQ	2.094762	1.999902	2.177352	2.186857	2.224839	1.36E-15	1.79E-17	2.25E-19	7.99E-19	4.34E-20	831	464	427	447	258
MAP7D1	-1.79751	-1.7474	-1.62151	-1.90999	-1.90225	1.36E-15	3.49E-16	5.08E-16	3.61E-19	4.05E-17	832	632	879	410	576
RELL1	-1.7146	-1.63646	-1.55806	-1.76556	-1.71806	1.39E-15	2.28E-15	1.90E-12	9.84E-17	6.66E-15	833	746	1660	698	945
CCN4	-2.53503	-2.09987	-1.93074	-2.29403	-2.08115	1.40E-15	2.64E-15	3.46E-15	1.15E-16	2.03E-14	834	752	1037	707	1056
ATP5F1B	2.145699	1.690758	2.072887	1.795881	2.048089	1.41E-15	1.76E-11	3.91E-13	3.30E-11	2.01E-16	835	1599	1487	1920	690
CLTA	1.527471	1.378891	1.634639	1.732125	1.321743	1.45E-15	3.02E-11	2.82E-12	5.70E-15	1.38E-12	836	1670	1714	991	1524
DENND3	-1.688	-1.41614	-1.59312	-1.55533	-1.65947	1.46E-15	1.81E-14	7.32E-16	3.54E-18	2.46E-15	837	881	909	511	862
SYNM	-1.56251	-1.38788	-1.68874	-1.45501	-1.71571	1.47E-15	1.31E-16	1.17E-20	1.63E-16	7.92E-21	838	589	330	734	204
EBP	1.952352	2.261293	2.02739	2.281276	2.201924	1.47E-15	7.03E-19	9.08E-19	1.30E-20	1.69E-20	839	327	483	296	225
KIAA1522	1.952785	2.002889	2.273673	2.000852	2.007946	1.47E-15	6.85E-18	3.38E-19	1.46E-20	1.29E-18	840	413	442	301	392
ARHGAP1!	-1.38301	-1.60345	-1.94074	-1.75229	-1.78048	1.47E-15	9.42E-17	3.50E-16	3.46E-14	6.10E-15	841	566	857	1147	937
ZMIZ1	-2.36003	-2.38401	-1.89518	-2.18338	-1.83771	1.52E-15	6.98E-20	5.02E-16	1.34E-15	9.79E-12	842	249	877	865	1762
DPYSL2	-2.42999	-2.69458	-2.36971	-2.56547	-2.23027	1.55E-15	3.65E-20	1.29E-16	4.21E-15	9.32E-14	843	225	786	962	1215
PAWR	1.879033	1.527918	1.825918	1.682311	1.572399	1.58E-15	1.29E-10	1.01E-15	4.81E-11	2.62E-14	844	1863	934	1969	1080
PAICS	1.451961	1.461358	1.714055	1.388152	1.137607	1.58E-15	7.39E-15	8.73E-19	7.39E-14	4.20E-08	845	818	480	1208	3087
FHL3	-1.98804	-1.55609	-1.64361	-1.71065	-1.84574	1.59E-15	7.21E-12	5.34E-13	8.27E-13	2.51E-17	846	1488	1521	1477	548
COX4I1	2.134239	1.891726	2.13379	2.142112	2.485219	1.62E-15	8.66E-18	1.13E-17	6.22E-16	8.12E-20	847	425	639	822	283
UNC5B	-3.21676	-3.16448	-2.89819	-3.28241	-3.12856	1.62E-15	2.94E-18	4.79E-17	1.48E-21	9.19E-17	848	378	710	238	635
GEM	-2.32387	-1.69559	-2.03338	-2.02802	-2.59531	1.63E-15	1.39E-08	1.64E-11	5.82E-09	2.49E-16	849	2614	1933	2677	712
SPINT1	2.354502	1.974484	2.306904	2.159335	2.442368	1.68E-15	2.34E-13	1.85E-16	1.57E-15	1.27E-15	850	1111	815	878	822
CSF2RB	-2.21025	-2.23529	-2.45258	-2.61742	-2.44769	1.69E-15	9.15E-14	1.68E-18	1.28E-14	1.81E-15	851	1026	516	1059	846
SUN2	-1.14205	-0.98587	-1.12093	-1.06384	-0.81216	1.69E-15	2.42E-13	3.76E-13	6.44E-08	0.000283	852	1115	1484	3081	5221
TMEM63A	2.107096	1.89933	2.100579	2.278448	2.245686	1.72E-15	4.48E-13	8.69E-16	2.66E-18	4.45E-15	853	1187	925	506	913
MGST1	2.316895	2.049835	2.096251	2.064696	1.520073	1.72E-15	2.08E-09	2.70E-10	1.02E-09	8.12E-08	854	2282	2367	2398	3236
MMP24OS	1.772434	1.676887	1.48223	1.78214	1.387181	1.72E-15	1.00E-16	3.35E-13	1.25E-16	6.93E-15	855	576	1470	714	949
RNASE6	-1.60053	-1.48093	-1.84896	-1.54314	-1.22898	1.74E-15	2.70E-15	6.59E-20	1.48E-13	8.65E-09	856	753	384	1277	2783
DBN1	-2.33493	-2.29695	-1.9626	-2.44902	-1.8774	1.74E-15	2.07E-19	1.25E-13	2.15E-17	2.13E-13	857	282	1348	608	1305
LILRB4	-2.20677	-2.1167	-2.32373	-2.33965	-2.64199	1.74E-15	2.24E-12	2.49E-15	1.28E-12	3.19E-18	858	1330	1008	1534	426
PIK3CD	-1.74031	-1.53218	-1.84212	-1.83369	-1.59333	1.78E-15	1.42E-10	1.67E-13	5.35E-13	2.10E-07	859	1881	1394	1428	3423
SH3RF3	-1.72738	-1.5201	-1.83518	-1.64266	-1.47397	1.78E-15	7.70E-14	1.94E-19	1.12E-14	3.35E-10	860	1015	421	1046	2235
MAP9	-1.24213	-1.1991	-1.15111	-1.23007	-1.23011	1.78E-15	9.92E-12	3.17E-13	1.03E-12	3.29E-14	861	1531	1466	1508	1110
NCF4	-1.62396	-1.51629	-1.8855	-1.77681	-1.57581	1.78E-15	4.43E-13	1.72E-14	4.85E-14	1.77E-09	862	1183	1155	1173	2519
FAM162A	1.467217	1.460247	1.812427	1.585935	1.384545	1.81E-15	1.01E-13	2.51E-18	1.20E-14	7.34E-11	863	1036	534	1053	2011
GPR161	-1.49254	-1.20626	-1.27916	-1.30152	-1.26262	1.81E-15	7.39E-12	5.16E-13	2.58E-13	1.14E-12	864	1493	1517	1340	1500
ABCA1	-2.27683	-2.14909	-2.47285	-2.1936	-2.4229	1.81E-15	3.73E-14	2.47E-19	7.17E-16	4.96E-18	865	947	432	827	453
ADAM19	-3.37874	-3.05776	-3.7956	-3.00106	-3.7621	1.84E-15	8.87E-12	5.02E-17	4.58E-15	9.94E-18	866	1516	711	968	499
SLC15A3	-1.92257	-2.16987	-2.26576	-2.22835	-2.31124	1.90E-15	1.15E-15	2.13E-18	4.04E-15	5.60E-19	867	707	531	961	350
UCHL3	2.286661	2.24255	2.210586	2.275364	2.345206	1.90E-15	1.23E-18	6.82E-17	2.06E-19	6.63E-18	868	341	738	387	470
HOXB2	-1.61234	-1.16001	-1.29511	-1.40189	-1.29683	1.92E-15	1.95E-12	1.24E-16	2.49E-15	1.05E-14	869	1317	784	915	991
MAP1A	-1.86087	-1.25942	-1.66266	-1.56434	-1.95936	1.94E-15	1.69E-08	2.38E-10	4.55E-10	1.02E-14	870	2636	2351	2269	983
LRPPRC	1.887993	1.906699	1.848803	1.870315	1.814996	1.98E-15	4.19E-17	9.82E-17	1.52E-15	2.65E-18	871	519	767	872	418
CMKLR1	-2.16686	-2.16686	-2.34298	-2.55602	-2.35495	1.99E-15	5.68E-15	1.15E-14	3.46E-17	1.22E-14	872	795	1128	640	1009
HSPD1	2.794047	2.943417	2.838727	3.151109	2.747173	2.02E-15	6.80E-16	1.26E-13	3.19E-20	7.57E-18	873	673	1350	318	480
HSD17B12	1.466881	1.627818	1.916424	1.987318	1.640783	2.11E-15	8.85E-12	3.74E-12	5.25E-14	3.98E-13	874	1514	1749	1179	1370
LRATD1	2.479087	2.994169	2.958646	3.192576	2.930418	2.30E-15	1.99E-17	1.17E-17	9.40E-19	1.94E-20	875	469	640	457	229

DLG4	-1.88128	-1.74053	-1.94247	-1.76297	-1.83593	2.34E-15	4.36E-12	3.65E-18	1.12E-14	1.37E-12	876	1416	566	1045	1522
C1orf216	-1.36647	-1.06793	-1.06917	-1.14656	-1.4766	2.36E-15	4.82E-12	6.26E-14	4.25E-12	3.28E-13	877	1432	1268	1659	1346
PRSS3	2.598671	2.462383	2.477212	2.533339	2.233011	2.40E-15	4.02E-16	3.62E-16	1.49E-15	6.12E-12	878	636	859	871	1693
GLIPR2	-2.08924	-1.57514	-2.06099	-1.76227	-2.24181	2.45E-15	1.61E-11	9.79E-16	5.79E-13	1.15E-16	879	1583	930	1440	656
GGTA1	-1.27513	-1.04114	-1.18664	-1.11738	-1.39181	2.48E-15	2.79E-09	1.19E-12	1.80E-10	4.42E-16	880	2336	1615	2131	747
BLVRA	-1.1994	-1.07616	-1.38185	-1.25	-1.13722	2.48E-15	2.50E-12	1.97E-13	1.65E-12	1.54E-10	881	1340	1414	1566	2112
DCTPP1	1.645336	1.624735	1.927758	1.884187	1.862031	2.55E-15	1.16E-14	6.68E-18	2.61E-15	2.91E-14	882	842	606	917	1098
ILVBL	1.426843	1.383492	1.623415	1.66407	1.410347	2.63E-15	9.52E-14	1.01E-15	3.54E-17	4.47E-16	883	1031	933	645	748
KLHL5	-2.75585	-2.39148	-2.56322	-2.73381	-3.01798	2.64E-15	2.55E-14	9.18E-17	8.97E-15	8.27E-16	884	914	761	1021	794
HSPA9	1.994275	1.880824	2.037343	1.915655	1.865142	2.66E-15	5.72E-17	2.59E-17	3.93E-18	1.73E-16	885	531	683	514	681
ATP5MC3	2.112247	1.861674	2.329409	2.287703	2.116762	2.69E-15	2.75E-17	1.97E-18	1.01E-18	3.13E-20	886	489	525	460	246
EPB41L4B	2.576414	2.798158	2.627521	2.87036	2.154649	2.71E-15	7.47E-15	1.70E-15	5.03E-15	1.73E-12	887	820	978	982	1541
CNN1	-3.33657	-2.97208	-3.44527	-3.13902	-3.41262	2.71E-15	2.40E-11	1.38E-15	1.11E-12	1.07E-17	888	1642	962	1518	503
LRATD2	1.359157	1.283282	1.478145	1.381862	1.318481	2.74E-15	1.56E-10	1.46E-16	9.31E-13	2.08E-14	889	1894	795	1488	1059
EIF2AK1	1.625301	1.398087	1.390019	1.332353	1.244429	2.80E-15	1.67E-12	9.22E-14	5.50E-11	5.97E-14	890	1305	1312	1985	1173
NES	-2.12267	-1.87163	-2.01222	-1.91582	-1.7745	2.83E-15	9.46E-18	1.53E-17	7.79E-17	7.73E-14	891	428	651	685	1195
ARRDC1	1.728299	1.779092	1.838131	1.986839	1.791548	2.85E-15	1.89E-16	1.39E-19	2.39E-18	6.08E-17	892	604	408	499	607
TAF10	1.233672	1.115878	1.228746	1.361757	0.920766	2.89E-15	6.01E-13	2.91E-13	5.23E-16	1.14E-08	893	1210	1458	800	2845
SLC43A3	-1.8275	-1.56101	-1.74291	-1.65483	-1.66997	2.92E-15	3.49E-13	4.53E-12	2.33E-13	1.38E-10	894	1157	1768	1330	2100
HTRA1	-3.81311	-3.77238	-3.66608	-3.51031	-4.14722	2.94E-15	1.66E-15	9.22E-17	2.67E-16	8.50E-19	895	730	762	769	371
PLXDC1	-2.89544	-2.71008	-2.67711	-2.97228	-3.01836	3.02E-15	4.35E-16	7.63E-16	9.85E-18	2.06E-16	896	643	914	577	693
DPYD	-2.04935	-1.92418	-2.0679	-2.0724	-2.02659	3.14E-15	1.76E-16	1.78E-14	1.33E-17	4.05E-15	897	601	1157	591	902
SLFN11	-2.61529	-2.22849	-2.84497	-2.33879	-2.93023	3.27E-15	1.18E-11	2.18E-14	1.37E-10	3.78E-17	898	1552	1171	2099	572
FBN1	-3.14573	-3.03389	-2.8984	-3.03579	-3.43761	3.31E-15	1.64E-15	1.80E-16	1.07E-17	1.88E-18	899	728	810	581	405
MZB1	-3.56979	-3.34043	-4.54346	-3.89287	-4.42655	3.39E-15	4.01E-15	1.40E-17	2.10E-12	6.97E-17	900	772	649	1588	614
LIMS2	-2.27525	-2.01276	-2.46823	-2.22651	-2.34826	3.42E-15	1.76E-12	2.74E-18	4.10E-13	5.85E-15	901	1310	542	1399	933
WARS1	-1.30989	-1.10519	-1.15778	-1.07892	-1.14713	3.47E-15	1.79E-12	7.52E-15	1.04E-14	2.40E-13	902	1312	1099	1038	1314
ATL2	1.193813	1.154192	1.02897	1.349176	1.044521	3.50E-15	2.93E-14	5.33E-14	3.19E-14	1.25E-07	903	929	1253	1139	3320
CYP51A1	2.083352	1.965924	2.038651	2.207468	2.128818	3.58E-15	2.65E-14	5.42E-15	1.32E-15	2.65E-18	904	919	1075	864	419
HES4	-1.71613	-1.41636	-1.61864	-1.55743	-1.48112	3.61E-15	1.34E-14	2.25E-16	1.22E-14	4.39E-13	905	854	827	1054	1380
JPT2	1.666366	1.635638	1.834275	1.795031	1.834287	3.70E-15	1.13E-14	1.27E-15	1.58E-18	4.71E-17	906	841	956	478	589
GAL3ST4	-1.42514	-1.57986	-1.71556	-1.73116	-1.56288	3.70E-15	5.16E-14	7.16E-14	3.37E-14	2.02E-11	907	974	1285	1144	1860
MYOCD	-1.68159	-1.17209	-1.79462	-1.30172	-1.3681	3.79E-15	3.03E-09	1.11E-13	1.15E-11	8.11E-08	908	2352	1329	1783	3235
RALY	1.644974	1.377442	1.589708	1.595091	1.541277	3.84E-15	3.57E-11	7.52E-15	1.19E-11	2.22E-13	909	1689	1098	1793	1309
AOC1	2.521471	2.625545	2.728934	2.542966	2.571282	3.99E-15	6.32E-16	5.71E-14	1.46E-18	1.69E-14	910	670	1259	474	1035
NCF1	-2.01671	-1.92218	-2.66245	-2.447	-2.39853	4.00E-15	1.59E-13	4.03E-17	1.08E-12	9.41E-13	911	1071	703	1517	1475
MRPS34	1.379822	1.365036	1.310928	1.378833	0.981704	4.01E-15	1.58E-15	1.41E-13	1.09E-16	3.85E-08	912	727	1370	702	3063
PABPC1	2.350297	2.405085	2.388344	2.476953	2.615324	4.08E-15	1.58E-17	3.41E-14	4.53E-19	1.46E-17	913	454	1209	427	519
RPS21	3.219222	3.222201	2.934642	3.548094	3.11672	4.12E-15	3.18E-17	3.19E-15	1.02E-21	2.41E-19	914	498	1030	224	321
SH3D19	1.367794	1.07963	1.430795	1.307101	1.051494	4.12E-15	9.08E-10	1.40E-13	3.19E-08	6.17E-07	915	2162	1367	2962	3645
GADD45B	-1.59379	-1.52041	-1.58985	-1.71216	-1.6752	4.13E-15	2.96E-13	8.39E-14	1.57E-18	1.15E-17	916	1130	1303	477	507
TTBK2	-1.02345	-0.79613	-1.00086	-0.91663	-0.99282	4.26E-15	1.87E-10	3.14E-12	1.36E-09	1.28E-11	917	1913	1730	2451	1798
RASSF2	-2.12718	-1.73675	-1.63697	-1.84686	-2.01674	4.33E-15	1.29E-09	2.10E-12	4.96E-10	8.36E-14	918	2213	1673	2276	1205
LILRB2	-1.91703	-1.58373	-1.79087	-1.6862	-1.86051	4.35E-15	4.57E-10	3.47E-15	1.08E-11	5.57E-13	919	2052	1038	1773	1406
PDGFB	-1.87033	-1.74097	-2.0112	-1.76865	-1.77788	4.36E-15	3.11E-13	2.79E-20	9.81E-15	7.89E-14	920	1137	354	1032	1199
DISC1	-1.25916	-1.05957	-1.40017	-1.30092	-1.15293	4.38E-15	4.83E-13	1.27E-14	5.75E-14	7.14E-12	921	1195	1133	1189	1715
RGCC	-2.23686	-2.03869	-2.22677	-2.18901	-1.64658	4.44E-15	1.73E-13	2.13E-17	1.74E-14	5.23E-08	922	1088	669	1080	3138
SLC25A39	1.695736	1.729635	1.637081	1.856789	1.8133	4.46E-15	2.01E-16	3.31E-15	1.03E-18	5.88E-16	923	607	1034	461	764
GFPT2	-1.50991	-1.73483	-1.77488	-1.93908	-1.76017	4.52E-15	5.28E-12	3.90E-15	1.63E-14	1.70E-16	924	1448	1043	1075	678
TBC1D10C	-1.52821	-1.13482	-1.65073	-1.34881	-1.53884	4.54E-15	2.34E-11	1.24E-16	5.44E-13	2.24E-12	925	1637	782	1431	1573
ATP9A	1.82373	1.797745	1.903406	2.022534	2.020099	4.54E-15	1.17E-14	2.67E-16	6.67E-18	1.36E-17	926	844	840	554	513
GASK1B	-2.39219	-2.39095	-2.53547	-2.36912	-2.58006	4.58E-15	8.50E-13	3.58E-20	1.76E-14	1.04E-14	927	1239	366	1084	987
SH3TC1	-1.36417	-1.1823	-1.49907	-1.23852	-1.40371	4.58E-15	1.00E-12	1.04E-17	8.28E-15	1.64E-14	928	1252	630	1013	1032
TPM3	1.346385	0.951358	1.185814	1.093796	1.388317	4.78E-15	1.14E-10	3.66E-14	9.73E-11	3.63E-17	929	1848	1214	2062	568
ZBTB18	1.193882	0.973741	1.286373	1.183596	1.025641	4.78E-15	4.19E-08	2.00E-13	1.54E-12	6.49E-07	930	2812	1417	1562	3653
SRI	2.037693	1.713892	2.118658	1.895135	2.069017	4.84E-15	2.54E-11	1.56E-13	3.19E-14	1.93E-11	931	1654	1384	1138	1856
DLD	1.243426	1.223293	1.237202	1.171654	1.284818	4.84E-15	6.78E-17	1.12E-17	5.09E-18	2.80E-14	932	544	638	533	1092
CDX1	2.208124	2.221695	2.24005	2.194106	2.341015	4.84E-15	1.37E-15	3.37E-20	1.32E-17	1.08E-20	933	717	364	590	218
CCL3	-1.72596	-2.12082	-2.23584	-2.135	-2.00746	4.84E-15	2.61E-12	2.31E-15	1.43E-12	5.38E-12	934	1348	1000	1551	1681
ELL2	-1.47024	-1.35344	-1.54266	-1.46777	-1.52831	4.92E-15	1.24E-12	4.88E-14	7.74E-10	5.37E-12	935	1272	1243	2358	1680
TOX2	-1.09753	-1.023	-1.04858	-1.06832	-0.76661	5.11E-15	5.33E-15	3.21E-15	2.13E-15	4.44E-05	936	790	1032	903	4686
MRC1	-1.71598	-1.84501	-1.85139	-1.8318	-1.9805	5.46E-15	2.53E-15	1.21E-13	5.06E-15	4.26E-13	937	751	1340	984	1376
SNURF	-1.51659	-1.31321	-1.30473	-1.44131	-0.82825	5.46E-15	3.43E-15	4.86E-14	2.59E-12	3.02E-05	938	766	1239	1604	4569
ANXA3	2.390991	2.343697	2.820602	2.157048	2.533018	5.49E-15	6.91E-14	1.55E-19	1.34E-13	5.78E-18	939	1009	410	1268	462
MACF1	-1.99872	-1.82133	-1.82131	-1.63393	-1.63547	5.53E-15	9.48E-17	6.56E-16	1.07E-14	3.12E-11	940	567	893	1042	1900
YWHAB	1.816011	1.633908	1.748842	1.785715	2.044658	5.53E-15	1.58E-13	4.46E-16	1.05E-14	3.98E-16	941	1070	871	1039	740
PJA2	-1.50295	-1.4109	-1.36848	-1.3921	-1.56793	5.58E-15	8.88E-16	2.24E-15	5.29E-15	2.22E-17	942	688	996	990	545
LTBP3	-2.00962	-1.73255	-2.20268	-1.91715	-2.56613	5.93E-15	8.26E-12	3.71E-16	3.09E-12	7.93E-15	943	1509	861	1630	963
PEA15	-1.6721	-1.69796	-1.66023	-1.69363	-1.63925	5.94E-15	3.20E-17	1.69E-17	1.16E-17	4.22E-15	944	499	659	582	905
ACY1	1.574929	1.303528	1.374561	1.401426	1.684709	5.95E-15	2.33E-14	1.33E-15	3.98E-15	7.87E-17	945	908	958	958	623
FGR	-1.48279	-1.29658	-1.40148	-1.51303	-1.59032	6.10E-15	7.57E-11	2.07E-11	3.10E-13	2.60E-13	946	1789	1985	1366	1320
GSPT1	1.317236	1.06719	1.269864	1.308133	1.010693	6.21E-15	1.94E-09	9.59E-12	2.41E-13	1.69E-09	947	2270	1869	1338	2508
CD79A	-2.42844	-2.38085	-3.22648	-3.07785	-2.7491	6.23E-15	2.27E-14	1.10E-15	6.40E-14	1.78E-10	948	905	944	1199	2133

NTM	-2.33344	-1.80365	-1.49996	-1.83779	-1.70206	6.27E-15	6.80E-13	3.68E-13	5.16E-13	3.64E-12	949	1217	1480	1426	1634
TMEM123	1.430731	1.432619	1.540468	1.640783	1.277107	6.49E-15	2.36E-15	1.65E-13	1.80E-15	1.98E-12	950	747	1393	893	1553
CST2	-2.41113	-2.07417	-1.98235	-2.12293	-1.75558	6.52E-15	1.28E-11	1.21E-12	5.39E-13	2.15E-11	951	1558	1619	1430	1866
LAMB1	-3.22118	-3.7517	-3.43046	-3.8685	-2.8454	6.60E-15	1.82E-16	1.95E-13	8.02E-18	1.02E-10	952	603	1413	562	2056
SLC52A2	1.84263	2.160072	2.00123	2.216961	1.683169	6.71E-15	1.10E-17	3.25E-15	1.92E-22	1.04E-13	953	437	1033	184	1223
PDGFD	-1.57485	-1.56681	-1.89087	-1.752	-1.54793	6.78E-15	3.85E-12	3.62E-18	3.46E-14	1.18E-11	954	1399	564	1148	1788
PPP2R1A	0.991234	0.840305	1.062569	0.929815	1.190414	6.80E-15	4.12E-11	3.66E-12	1.37E-09	1.15E-17	955	1719	1746	2452	508
PDLIM7	-2.88731	-3.03395	-2.72544	-3.04758	-2.96334	6.83E-15	2.22E-17	1.20E-15	2.33E-17	9.25E-19	956	473	952	615	377
MFAP2	-3.0438	-2.53649	-2.31466	-2.7	-2.77798	6.84E-15	6.08E-13	2.05E-13	3.50E-14	8.02E-18	957	1211	1421	1150	484
NUMBL	-1.4696	-1.05146	-0.99648	-1.11781	-1.42624	6.90E-15	1.85E-11	1.52E-11	8.78E-12	2.14E-15	958	1605	1925	1740	853
CYLD	-1.14257	-1.23383	-1.16939	-1.23199	-1.33082	7.05E-15	1.40E-15	2.41E-17	7.07E-14	7.24E-16	959	720	679	1206	776
SOX18	-2.883	-3.00775	-2.99751	-2.98889	-2.69423	7.10E-15	2.64E-17	1.16E-18	3.17E-17	3.04E-13	960	484	498	632	1338
PXDC1	-1.61527	-1.34282	-1.39533	-1.49932	-1.4436	7.12E-15	3.57E-13	4.55E-13	1.76E-15	1.14E-13	961	1162	1503	891	1235
TNFRSF18	-1.6156	-1.36124	-1.72704	-1.63092	-1.83076	7.20E-15	5.01E-10	4.92E-13	9.73E-12	3.10E-14	962	2067	1509	1756	1104
MYH10	-1.7162	-1.27771	-1.40104	-1.39772	-1.63687	7.22E-15	1.81E-11	6.94E-13	2.67E-13	3.44E-14	963	1602	1553	1348	1115
CCT6A	1.738431	1.682292	1.859959	1.881097	1.846284	7.38E-15	2.02E-16	7.99E-16	2.35E-19	2.26E-15	964	609	916	390	856
CDC42SE1	-1.00512	-0.98904	-1.16858	-1.11515	-0.99796	7.44E-15	1.69E-13	2.44E-14	1.29E-11	6.05E-13	965	1084	1179	1806	1423
PLD3	-2.24428	-2.49827	-2.15056	-2.58736	-2.34562	7.51E-15	3.09E-13	1.77E-12	1.51E-16	5.17E-14	966	1136	1656	728	1158
TMEM141	1.741685	1.760759	2.013894	1.765775	2.034218	7.55E-15	4.46E-16	4.24E-14	4.21E-18	1.20E-14	967	644	1230	520	1006
MMP9	-3.60556	-4.07388	-4.00941	-3.82752	-4.55771	7.74E-15	4.42E-15	5.00E-15	5.11E-14	4.23E-17	968	776	1067	1177	581
ANKDD1A	-1.26439	-0.75187	-1.02943	-0.94236	-0.93437	7.79E-15	0.000109	2.57E-08	3.89E-06	2.37E-07	969	4724	3221	3960	3453
FXYD6	-1.95078	-2.05687	-1.97075	-2.13242	-1.98385	7.79E-15	6.05E-15	8.37E-14	3.02E-15	1.24E-17	970	800	1302	927	510
AP1M1	-1.211	-0.75137	-1.0942	-0.95897	-1.36617	7.94E-15	5.69E-07	1.40E-12	3.67E-08	2.54E-15	971	3340	1629	2989	864
IFI16	-2.31993	-2.73301	-2.84642	-2.91633	-2.55752	7.95E-15	1.56E-15	1.80E-16	6.70E-22	5.14E-13	972	726	811	214	1398
SEC61G	1.235589	0.86479	1.167539	0.881152	1.253886	7.96E-15	2.02E-07	3.89E-10	6.78E-07	2.29E-12	973	3152	2427	3570	1578
ADAMTSL4	-1.49084	-1.45495	-1.58724	-1.74008	-1.70603	8.03E-15	2.67E-12	1.67E-12	1.82E-14	1.61E-11	974	1352	1651	1088	1827
PTPN9	-1.15807	-1.06162	-0.99286	-1.21292	-0.71937	8.11E-15	3.63E-12	9.87E-12	3.80E-16	3.90E-08	975	1391	1874	785	3065
EPPK1	2.292695	2.187753	2.504351	2.354503	2.202222	8.14E-15	1.12E-16	8.76E-20	6.60E-17	3.46E-22	976	581	387	676	120
COL7A1	-4.04962	-4.05719	-3.8556	-4.39424	-4.36676	8.23E-15	2.35E-12	1.72E-14	4.37E-15	1.81E-15	977	1334	1156	965	845
CCL4L2	-1.82955	-2.08024	-2.33126	-2.20486	-2.2595	8.30E-15	1.95E-12	1.02E-16	5.74E-13	1.50E-16	978	1318	771	1439	672
HAVCR2	-1.68352	-1.52585	-1.90066	-1.86348	-1.48476	8.38E-15	6.21E-12	1.68E-16	3.31E-14	8.73E-07	979	1467	807	1142	3704
HAS2	-1.77528	-1.52385	-1.62586	-1.62326	-1.46514	8.42E-15	9.86E-12	2.63E-19	4.84E-13	1.49E-12	980	1530	433	1419	1532
CREM	-1.43111	-1.23539	-1.37307	-1.30137	-1.66802	8.71E-15	2.79E-12	2.99E-15	1.01E-13	5.81E-13	981	1360	1020	1245	1416
UBL5	1.121343	1.16758	1.106514	1.225	1.083759	9.03E-15	2.72E-12	1.08E-13	1.39E-14	2.04E-13	982	1357	1327	1063	1298
CRYBG3	-0.97537	-1.02495	-1.11402	-1.088	-0.95199	9.04E-15	1.62E-13	3.11E-13	8.64E-13	2.46E-10	983	1075	1463	1482	2193
ADPRH	-1.22868	-1.14568	-1.46716	-1.23539	-1.02346	9.18E-15	4.69E-11	1.38E-16	1.46E-12	2.96E-08	984	1742	792	1553	3023
CST7	-1.80188	-1.22203	-1.71016	-1.59335	-1.70296	9.27E-15	3.31E-09	1.03E-12	2.01E-10	2.82E-10	985	2369	1599	2152	2220
CANT1	2.054181	1.852899	2.126654	2.031917	1.753294	9.30E-15	4.16E-14	2.34E-17	3.96E-14	1.62E-14	986	954	676	1160	1029
EIF3E	1.781671	1.875579	1.945002	1.975672	1.588968	9.37E-15	5.45E-17	2.02E-13	8.50E-17	3.85E-12	987	530	1419	690	1642
GTF2IRD1	1.799719	1.910547	2.446382	1.980813	1.966967	9.42E-15	1.04E-15	6.98E-17	1.76E-13	4.91E-14	988	700	739	1304	1150
GPC1	-2.06923	-1.30783	-1.61569	-1.73076	-2.15553	9.68E-15	5.08E-08	1.59E-12	1.85E-10	1.58E-14	989	2849	1644	2137	1027
PLA2G7	-2.14854	-2.09606	-2.29082	-2.22834	-2.59938	9.68E-15	8.32E-13	3.91E-17	1.21E-11	1.43E-17	990	1236	699	1794	516
CCT5	1.747552	1.70127	1.771865	1.841749	1.695278	1.01E-14	6.91E-14	2.48E-13	4.95E-18	4.37E-15	991	1008	1444	530	911
BRI3BP	1.575473	1.829049	1.601561	1.838262	1.6978	1.03E-14	3.72E-16	6.14E-16	7.84E-19	7.81E-18	992	633	887	446	482
MST1R	2.269254	2.520315	2.778227	2.5177	2.068301	1.03E-14	9.53E-16	2.76E-19	2.16E-17	2.31E-15	993	694	434	610	858
KANK2	-2.09022	-2.19547	-1.93141	-2.24844	-1.91092	1.04E-14	1.67E-15	4.67E-16	2.31E-18	1.05E-14	994	731	873	497	993
POLR2I	1.097335	1.092183	1.13103	0.917257	1.011803	1.06E-14	1.07E-11	1.02E-13	4.66E-11	5.29E-11	995	1542	1317	1966	1954
LRRC59	1.232351	1.129496	1.362752	1.372155	1.311754	1.07E-14	4.36E-15	7.05E-14	6.40E-18	4.07E-11	996	774	1281	552	1929
SNAI2	-2.43271	-2.25054	-2.06502	-2.30505	-2.29058	1.07E-14	6.60E-14	1.21E-12	3.41E-15	6.33E-15	997	1001	1617	939	940
TCF21	-2.60981	-2.12158	-2.35367	-2.31962	-2.52261	1.10E-14	1.14E-10	2.27E-14	3.55E-13	1.23E-13	998	1845	1175	1384	1244
GSTP1	2.671066	2.290564	2.820213	2.6298	2.577471	1.15E-14	4.05E-16	1.72E-15	3.21E-17	1.45E-17	999	637	980	634	518
ALDH1L2	-1.28525	-0.83253	-1.11882	-1.12595	-0.93473	1.17E-14	2.18E-06	5.29E-10	6.85E-10	3.38E-06	1000	3681	2469	2336	4005
HSP90AA1	1.995667	1.835489	2.148751	2.073731	2.112069	1.17E-14	1.29E-13	1.75E-13	1.06E-15	5.58E-15	1001	1056	1400	851	929
SLC22A18	1.790711	1.471409	1.616897	1.626651	1.621309	1.17E-14	1.68E-13	1.62E-13	4.83E-15	9.73E-15	1002	1083	1391	977	981
MBNL1	-0.96232	-0.82535	-0.89259	-1.09335	-0.80236	1.18E-14	1.88E-11	1.08E-12	3.65E-15	2.19E-12	1003	1606	1607	945	1570
CLEC7A	-2.0594	-1.74106	-2.17604	-1.90648	-2.28629	1.19E-14	3.18E-11	3.67E-14	5.62E-11	2.73E-17	1004	1673	1215	1992	554
GPI	1.723155	1.809372	2.015452	1.948799	1.732227	1.22E-14	6.86E-18	1.52E-16	5.15E-18	2.92E-16	1005	414	803	535	722
IGDCC4	-2.01222	-1.43185	-1.50041	-1.7443	-1.53462	1.23E-14	1.23E-10	9.24E-12	2.93E-16	1.64E-10	1006	1857	1860	773	2121
TM9SF3	1.729038	1.415563	1.814612	1.713707	1.732999	1.27E-14	7.80E-16	6.88E-14	1.28E-14	2.44E-12	1007	681	1276	1061	1586
SERINC5	1.666336	1.74809	1.852689	2.044914	1.933421	1.28E-14	4.48E-15	1.13E-14	2.99E-15	2.65E-19	1008	778	1127	925	322
CACNA2D1	-1.62739	-1.3438	-1.40349	-1.39597	-1.279	1.30E-14	7.26E-15	1.37E-14	1.09E-15	1.58E-11	1009	817	1137	853	1822
DBNDD2	1.41157	1.32034	1.449417	1.338177	1.442315	1.31E-14	9.44E-11	2.99E-12	2.22E-10	2.14E-12	1010	1824	1721	2165	1563
LATS2	-1.60413	-1.34626	-1.61406	-1.71059	-1.22653	1.34E-14	9.91E-13	1.05E-13	2.09E-16	1.91E-08	1011	1250	1320	746	2932
TMEM273	-1.45437	-1.2839	-1.23746	-1.72242	-1.10666	1.36E-14	9.84E-11	9.63E-09	7.39E-20	1.50E-06	1012	1833	3004	338	3819
TRAF3IP3	-1.6237	-1.17666	-1.99791	-1.51633	-1.71302	1.38E-14	2.89E-10	3.72E-16	9.74E-13	1.07E-13	1013	1975	863	1496	1225
NR1I2	1.541482	1.383174	1.181694	1.27365	1.061522	1.41E-14	3.01E-12	1.71E-07	7.01E-08	0.000123	1014	1366	3628	3093	4997
FBL	1.820771	1.904731	1.932071	1.872454	1.972083	1.42E-14	1.62E-14	4.56E-14	4.24E-16	1.28E-16	1015	872	1233	789	661
U2AF2	1.075149	0.936858	1.064704	0.915591	1.114726	1.44E-14	1.35E-09	1.43E-12	3.13E-09	1.10E-14	1016	2220	1632	2591	997
APH1A	1.298971	0.839013	1.026669	1.148676	1.165967	1.44E-14	2.28E-07	3.50E-09	5.70E-10	4.64E-10	1017	3175	2820	2298	2297
CEP170B	1.453268	1.733345	1.683475	1.401343	1.308905	1.47E-14	3.38E-16	2.48E-16	6.53E-13	5.59E-10	1018	630	835	1455	2322
RBMS1	-2.22592	-1.67892	-1.96855	-1.73126	-2.135	1.52E-14	7.02E-14	1.23E-18	6.71E-14	2.88E-18	1019	1010	500	1204	421
CRB3	1.719839	1.673457	1.979922	1.648684	1.665776	1.54E-14	6.61E-13	1.98E-15	9.94E-14	2.06E-12	1020	1216	991	1244	1561
ARHGAP29	-1.96672	-1.39315	-1.50853	-1.42885	-1.80742	1.56E-14	2.62E-08	4.53E-11	9.42E-10	2.02E-13	1021	2712	2091	2389	1296

SMC1A	1.243483	1.181539	1.034277	1.283348	1.230351	1.56E-14	2.77E-14	1.91E-12	2.39E-12	2.92E-15	1022	925	1661	1599	870
PPM1F	-1.07024	-0.94579	-0.83582	-0.92609	-0.97996	1.58E-14	2.09E-15	1.86E-11	6.18E-14	5.43E-16	1023	739	1964	1196	761
CNTRL	-0.89514	-0.65593	-0.82344	-0.69649	-0.90332	1.58E-14	2.51E-10	8.41E-13	1.43E-11	7.89E-14	1024	1962	1569	1813	1198
WNT2B	-2.07357	-1.53445	-1.54757	-1.67186	-1.84348	1.64E-14	1.73E-12	5.34E-13	1.32E-13	7.13E-14	1025	1307	1522	1266	1192
KIFC3	-2.19669	-2.00443	-2.23159	-2.5362	-2.27699	1.68E-14	2.78E-13	1.63E-12	3.19E-17	1.94E-14	1026	1126	1648	633	1054
PIK3R3	-1.2947	-1.09753	-1.32567	-1.16226	-0.82287	1.68E-14	2.41E-13	4.64E-15	3.82E-13	3.39E-05	1027	1114	1058	1394	4601
TFPI	-2.29394	-2.44628	-2.34837	-2.63158	-2.7153	1.69E-14	1.35E-16	1.40E-13	2.96E-16	2.37E-17	1028	590	1368	774	547
ERGIC3	1.970536	1.912117	1.915528	2.011649	1.688942	1.78E-14	7.19E-14	3.71E-16	6.49E-14	3.50E-15	1029	1012	862	1200	888
ELOA3P	-1.33237	-1.2336	-1.78005	-1.38449	-1.73477	1.79E-14	9.68E-17	2.15E-20	7.47E-15	3.88E-17	1030	574	349	1007	573
RPL8	2.49227	2.597718	2.538141	2.710843	2.641366	1.83E-14	2.80E-17	2.48E-13	1.64E-19	2.22E-17	1031	492	1445	378	544
CD36	-1.5391	-1.38067	-1.54681	-1.23771	-1.18857	1.86E-14	2.52E-13	2.78E-14	6.83E-13	6.50E-08	1032	1117	1193	1459	3175
UQCR11	1.485096	1.561964	1.579041	1.565822	1.218495	1.87E-14	1.35E-16	1.34E-15	2.20E-18	6.74E-12	1033	591	960	491	1711
HOXB9	2.800845	3.080006	3.015488	3.185037	3.537665	1.91E-14	8.30E-20	6.19E-17	8.49E-19	6.75E-18	1034	258	726	450	473
IL3RA	-1.67426	-1.38627	-1.85145	-1.51305	-1.68227	1.94E-14	2.18E-09	5.03E-13	7.59E-10	8.03E-10	1035	2292	1513	2354	2391
NDUFB10	1.240875	1.059355	1.258282	1.161167	1.233035	1.95E-14	1.67E-11	2.01E-13	3.91E-12	3.90E-15	1036	1591	1418	1652	896
SLC35A3	1.896198	1.604528	2.026959	1.711317	1.701558	1.96E-14	1.35E-14	1.16E-18	7.57E-15	3.98E-18	1037	856	495	1009	436
LDB1	-1.51154	-1.34521	-1.5222	-1.2517	-1.1233	2.01E-14	6.15E-15	1.24E-16	2.73E-12	2.14E-10	1038	803	783	1615	2173
DNM1	-2.16565	-1.79067	-1.24676	-1.60502	-1.99017	2.02E-14	5.67E-10	1.24E-08	8.71E-09	7.89E-12	1039	2093	3069	2735	1727
PHC1	-1.11367	-1.08277	-1.17699	-1.10763	-0.99936	2.02E-14	7.31E-14	1.02E-15	5.32E-13	1.75E-10	1040	1014	935	1427	2132
RSL1D1	1.555061	1.357992	1.494554	1.518217	1.251975	2.04E-14	1.43E-13	3.11E-14	5.33E-14	2.93E-11	1041	1063	1198	1181	1892
CAV2	-2.56191	-2.16807	-2.27466	-2.53198	-2.35	2.05E-14	2.52E-14	8.12E-15	1.89E-18	4.23E-13	1042	913	1106	487	1373
CCDC71L	-1.80927	-1.42126	-1.68312	-1.69732	-1.63926	2.06E-14	4.82E-10	3.17E-14	1.61E-11	1.95E-13	1043	2060	1200	1835	1292
NDST2	-1.15981	-0.79374	-0.97634	-0.90357	-1.02546	2.08E-14	1.01E-06	4.91E-12	1.58E-09	1.07E-11	1044	3481	1782	2481	1773
MSR1	-1.82983	-1.94141	-2.04056	-1.94458	-2.27299	2.09E-14	1.65E-13	1.05E-17	8.42E-15	4.91E-18	1045	1079	633	1014	450
DIXDC1	-1.01458	-0.67213	-0.92831	-0.89291	-0.98062	2.10E-14	3.27E-10	7.66E-11	1.10E-11	1.56E-13	1046	2007	2166	1776	1269
ATP5F1E	1.371942	1.444454	1.154199	1.446109	1.418491	2.14E-14	5.42E-14	2.49E-15	1.52E-16	3.40E-15	1047	980	1007	730	885
PREP	1.473384	1.511029	1.512327	1.713735	1.522427	2.14E-14	4.33E-14	3.76E-14	7.61E-17	3.39E-14	1048	958	1217	684	1113
SPN	-1.19831	-1.02078	-1.39324	-1.37889	-1.39388	2.16E-14	7.43E-10	7.01E-16	9.34E-15	5.56E-18	1049	2128	903	1025	459
B3GALNT1	-1.15761	-0.99391	-1.2306	-1.16628	-1.09864	2.19E-14	3.22E-13	4.28E-10	2.55E-11	7.37E-10	1050	1147	2441	1896	2377
TUFM	1.812396	1.561853	1.7996	1.803295	1.888382	2.21E-14	4.16E-14	5.36E-15	7.46E-17	1.67E-15	1051	953	1072	683	841
SUN1	1.013629	0.967834	1.068859	1.124662	0.766472	2.24E-14	2.09E-10	1.99E-12	1.27E-12	6.14E-09	1052	1934	1665	1531	2717
ATP5MK	1.841852	1.702266	2.064757	1.876492	1.601955	2.28E-14	5.21E-16	1.35E-15	2.76E-15	6.03E-13	1053	662	961	920	1421
SNED1	-2.13855	-1.4491	-1.85212	-1.70629	-2.15014	2.31E-14	7.94E-10	7.43E-14	7.12E-11	3.09E-15	1054	2143	1291	2027	876
CD83	-1.07952	-1.04232	-1.0556	-1.00487	-0.9726	2.40E-14	8.91E-10	1.45E-10	1.01E-09	1.79E-08	1055	2156	2274	2394	2924
POU2AF1	-2.19408	-1.67549	-2.75786	-2.45944	-2.47233	2.40E-14	7.26E-10	5.45E-13	1.10E-09	6.24E-11	1056	2120	1525	2414	1981
C1QTNF6	-1.63926	-1.77976	-1.88535	-1.94585	-2.13459	2.42E-14	7.43E-17	5.71E-13	1.28E-16	1.70E-19	1057	549	1530	717	309
NAXE	1.385729	1.575108	1.345262	1.734162	1.245571	2.45E-14	1.34E-12	3.19E-11	5.74E-16	1.89E-10	1058	1278	2055	812	2146
SSH1	-1.48789	-1.26903	-1.35873	-1.26558	-1.70405	2.45E-14	1.20E-12	3.81E-14	8.52E-14	1.44E-17	1059	1268	1224	1229	517
SNRK	-1.22064	-1.1172	-1.04122	-0.99445	-1.19751	2.49E-14	1.32E-13	5.29E-15	2.73E-12	2.47E-15	1060	1059	1070	1614	863
ROMO1	1.703495	1.749373	1.545995	1.906511	1.853705	2.54E-14	3.90E-17	7.99E-15	6.75E-20	2.14E-16	1061	513	1102	337	698
BMF	-1.586	-1.49907	-1.69098	-1.67383	-1.55763	2.58E-14	1.97E-10	2.14E-14	2.31E-11	4.05E-14	1062	1921	1169	1885	1137
MMP19	-1.9289	-1.8685	-1.69418	-2.1219	-2.03328	2.58E-14	5.87E-12	5.37E-15	1.76E-14	1.77E-14	1063	1460	1073	1083	1044
NCF2	-2.0393	-1.93208	-1.86267	-1.99379	-1.83611	2.59E-14	1.29E-11	1.01E-14	9.83E-13	1.32E-10	1064	1559	1118	1499	2090
SEPTIN6	-1.89778	-1.74052	-2.10661	-2.0372	-2.15557	2.59E-14	5.89E-15	4.60E-19	3.73E-19	5.30E-16	1065	798	460	412	756
ATP5MC1	1.81529	1.981109	1.777695	1.879385	1.841969	2.62E-14	2.78E-17	1.40E-13	1.99E-17	1.84E-18	1066	490	1369	603	403
WNK2	2.182609	2.742758	1.937722	2.507383	2.105185	2.64E-14	4.68E-17	3.14E-11	5.04E-15	3.65E-13	1067	525	2051	983	1361
TPRN	1.900769	2.007046	2.304577	1.931223	1.826719	2.66E-14	4.25E-17	1.61E-19	3.82E-15	8.49E-12	1068	520	412	951	1741
ATP5MC2	1.265864	1.011714	0.99239	1.01197	1.204817	2.66E-14	3.60E-10	4.16E-12	5.57E-08	9.92E-11	1069	2025	1757	3051	2052
MRPL14	1.379345	1.292126	1.37511	1.310859	1.480732	2.70E-14	5.06E-14	6.56E-15	6.00E-15	8.08E-19	1070	972	1087	995	369
DDR2	-3.13314	-2.68034	-2.46022	-2.79119	-3.15127	2.70E-14	8.86E-12	2.17E-11	9.45E-13	9.15E-16	1071	1515	1993	1492	803
UBE2E2	-1.96984	-1.57874	-1.73278	-1.85496	-1.91535	2.71E-14	7.91E-10	2.94E-12	9.47E-14	4.03E-14	1072	2140	1718	1240	1136
RPL19	2.160225	1.919219	1.956999	2.020472	1.997845	2.72E-14	3.40E-14	1.13E-15	1.57E-13	1.23E-16	1073	936	949	1287	659
SUCLG1	1.616459	1.770186	1.885936	1.825628	1.690138	2.73E-14	4.50E-15	3.94E-17	5.79E-15	1.86E-18	1074	779	701	992	404
ARF5	1.084096	1.190653	1.293346	1.34871	1.385638	2.75E-14	6.70E-11	6.87E-16	9.60E-13	4.00E-14	1075	1779	899	1493	1135
MYO5A	-1.40113	-0.87415	-1.20054	-1.19778	-1.51605	2.75E-14	2.27E-07	9.79E-10	1.10E-08	1.41E-13	1076	3174	2574	2777	1260
MNDA	-1.85899	-1.83574	-1.75337	-1.75505	-1.8363	2.75E-14	2.65E-11	2.83E-11	1.14E-10	1.15E-14	1077	1658	2035	2075	1001
HSP90AB1	2.213951	1.895216	2.114627	2.159011	2.238858	2.78E-14	3.17E-14	1.24E-13	5.24E-16	4.62E-17	1078	935	1346	801	587
CDK17	-1.28304	-1.46812	-1.55636	-1.48486	-1.43044	2.80E-14	9.64E-15	1.33E-16	3.99E-14	1.75E-14	1079	833	787	1162	1040
TPD52	2.169521	2.040474	2.697543	2.031263	1.954785	2.84E-14	2.90E-14	1.16E-18	1.67E-12	5.77E-18	1080	927	496	1568	461
PRKDC	1.353975	1.656536	1.682106	1.743454	1.672006	2.88E-14	3.53E-17	6.03E-15	4.95E-18	4.22E-17	1081	509	1081	531	579
SMS	1.472687	1.391851	1.654284	1.634209	1.52425	2.94E-14	7.85E-16	3.55E-12	5.31E-16	3.44E-13	1082	682	1744	804	1353
ARHGAP2:	1.416039	1.331152	1.071993	1.202024	1.420561	2.99E-14	3.13E-12	6.49E-10	3.88E-10	9.71E-12	1083	1368	2509	2241	1759
LAMB3	2.026445	1.689653	2.174286	1.77339	1.876742	3.04E-14	1.57E-11	1.57E-15	7.99E-18	3.22E-13	1084	1582	973	560	1342
KRTAP9-1	-0.95583	-0.65274	-1.02123	-0.81272	-0.61854	3.08E-14	1.22E-09	3.33E-14	2.16E-10	0.000287	1085	2202	1204	2161	5225
NANS	1.27375	1.298948	1.50647	1.525176	0.967299	3.11E-14	1.05E-11	1.82E-16	4.79E-15	3.93E-06	1086	1540	814	976	4046
GUCY1A2	-1.51075	-1.11312	-1.28672	-1.24884	-1.54398	3.12E-14	3.26E-10	7.14E-11	1.68E-12	1.23E-11	1087	2005	2157	1570	1793
WDR5	1.055638	1.117979	0.915082	1.031482	0.804531	3.19E-14	8.96E-17	9.01E-15	3.03E-14	2.51E-08	1088	563	1113	1135	2981
STOML2	1.551874	1.567648	1.887543	1.661163	1.398114	3.20E-14	1.21E-12	5.15E-14	7.66E-12	1.42E-11	1089	1270	1250	1720	1811
TMEM158	-3.20388	-2.70706	-3.00429	-2.73308	-3.22748	3.20E-14	2.91E-13	6.68E-17	5.63E-14	7.06E-17	1090	1127	733	1186	615
DENND5A	-1.52404	-1.48347	-1.41329	-1.80533	-1.80267	3.21E-14	9.66E-14	3.51E-13	1.16E-16	8.43E-16	1091	1032	1474	709	796
RNF144A	-1.49556	-1.36079	-1.48227	-1.42197	-1.45348	3.28E-14	1.48E-14	2.57E-21	3.87E-15	3.79E-14	1092	862	266	955	1125
SNRPB	1.870533	1.738055	1.839133	1.701613	1.753403	3.31E-14	4.75E-16	2.73E-16	2.30E-16	4.20E-16	1093	647	842	752	741
AKAP5	-1.09846	-0.81294	-0.95597	-0.94832	-1.08778	3.33E-14	9.58E-09	9.47E-12	1.53E-09	4.89E-12	1094	2547	1865	2476	1671

MMP14	-3.93206	-4.38345	-3.46736	-4.1922	-4.23147	3.34E-14	7.62E-21	4.65E-15	1.16E-20	4.57E-18	1095	176	1060	292	446
BCAT1	-1.8135	-1.48643	-1.78368	-1.60706	-1.9813	3.35E-14	3.35E-11	7.77E-13	7.58E-11	3.55E-15	1096	1681	1563	2032	890
SRSF1	1.435307	1.313902	1.381266	1.520569	1.435364	3.35E-14	5.57E-14	9.43E-13	1.64E-15	2.34E-14	1097	986	1587	881	1068
ATAD3A	1.213193	1.209189	1.084209	1.228289	1.506112	3.43E-14	3.28E-13	5.89E-11	3.22E-14	1.20E-15	1098	1148	2133	1141	820
BOLA2B	1.37018	1.155247	1.144701	1.513631	1.344407	3.47E-14	7.40E-10	7.27E-10	1.78E-13	1.67E-09	1099	2126	2528	1306	2507
FCHSD2	-1.53048	-1.28944	-1.94954	-1.54521	-1.8477	3.54E-14	3.58E-11	2.08E-18	3.82E-13	6.56E-17	1100	1692	528	1395	610
CDCA7	2.407845	2.624352	2.350559	2.691585	2.676823	3.59E-14	1.70E-16	4.63E-15	6.96E-17	3.20E-18	1101	598	1057	681	427
WDR7	-1.47473	-1.33403	-1.6641	-1.53695	-1.55933	3.65E-14	7.59E-12	3.33E-22	2.37E-13	2.98E-14	1102	1501	206	1333	1100
NSDHL	1.395705	1.315042	1.388989	1.411361	0.893585	3.68E-14	6.60E-11	6.33E-14	1.64E-16	5.86E-08	1103	1776	1269	735	3157
NDUFA10	0.880618	1.117687	1.230431	1.239745	1.165027	3.70E-14	2.99E-11	2.97E-14	6.42E-13	4.47E-13	1104	1669	1195	1453	1384
A4GALT	-2.03489	-1.47708	-1.64794	-1.62858	-1.76568	3.70E-14	3.31E-11	3.25E-14	1.55E-13	1.37E-12	1105	1680	1201	1284	1523
ZNF134	-1.19815	-1.02314	-1.19572	-1.40869	-1.08365	3.72E-14	8.01E-10	2.04E-11	6.10E-16	2.05E-11	1106	2144	1981	818	1862
SUSD6	-1.72464	-1.65527	-1.50327	-1.55348	-1.61783	3.74E-14	1.65E-12	1.24E-13	1.18E-11	6.47E-14	1107	1304	1347	1788	1182
PALM2AK/	-1.61844	-1.43875	-1.75919	-1.4326	-1.63145	3.85E-14	1.05E-09	1.76E-13	4.20E-11	2.28E-12	1108	2182	1402	1954	1577
CSE1L	1.945076	2.128816	1.879941	1.911537	1.355166	3.87E-14	6.78E-17	9.46E-17	1.41E-13	2.40E-07	1109	543	764	1273	3457
CLMP	-2.08303	-1.82452	-1.62692	-1.84607	-2.27971	3.87E-14	8.62E-11	1.70E-11	1.30E-11	1.05E-14	1110	1809	1939	1807	992
PRDX2	1.998146	1.85688	1.95411	2.301412	1.984135	3.96E-14	3.74E-13	1.45E-11	2.41E-20	5.14E-15	1111	1166	1918	310	922
NOP58	1.587827	1.754712	1.533286	1.827781	1.328903	4.03E-14	3.16E-16	9.21E-14	6.71E-21	5.58E-14	1112	626	1311	277	1167
H2AC6	1.756102	1.57126	1.962239	1.704707	1.902226	4.05E-14	2.37E-13	7.26E-17	4.09E-13	7.26E-19	1113	1113	742	1398	363
RPL18	2.292482	2.178797	2.124319	2.279251	2.219616	4.13E-14	1.99E-14	2.47E-12	1.23E-16	1.71E-14	1114	886	1689	713	1037
USP17L3	-1.39699	-1.00317	-1.62008	-1.22913	-1.10952	4.14E-14	5.26E-09	1.51E-13	2.27E-10	5.14E-08	1115	2447	1380	2166	3132
GBP1	-1.81595	-2.31318	-2.05083	-2.15054	-2.11402	4.18E-14	4.81E-16	6.84E-15	6.91E-15	2.43E-15	1116	652	1091	1001	860
MYO1D	1.719711	1.456938	1.743613	1.750918	1.533261	4.20E-14	1.85E-11	2.18E-14	5.39E-17	5.86E-12	1117	1604	1172	669	1689
DOCK11	-1.2356	-0.77085	-0.95779	-1.0626	-0.85805	4.23E-14	5.37E-05	3.71E-10	1.98E-08	1.20E-07	1118	4506	2418	2886	3318
TSPAN9	-2.11916	-1.86433	-1.84674	-1.84617	-2.27585	4.24E-14	1.18E-15	5.28E-15	1.24E-13	1.18E-18	1119	709	1069	1260	386
SELENBP1	2.363294	2.350429	2.426982	2.278066	2.414498	4.25E-14	1.82E-14	8.09E-14	2.61E-12	5.60E-13	1120	882	1298	1607	1408
ADAMTS1	-2.4658	-2.41487	-2.3843	-2.4795	-2.50191	4.27E-14	5.27E-14	1.39E-14	2.26E-17	1.39E-14	1121	977	1139	614	1020
NDUFA9	1.192448	1.01907	1.145805	1.213952	0.91267	4.36E-14	2.50E-10	1.02E-12	1.44E-12	3.87E-07	1122	1961	1595	1552	3562
GPR68	-1.60373	-1.28673	-1.53583	-1.60352	-1.6968	4.47E-14	1.42E-12	2.51E-14	4.65E-15	3.48E-14	1123	1283	1183	971	1118
ATP5IF1	1.518008	1.259202	1.690529	1.435324	1.529438	4.48E-14	1.73E-18	6.31E-18	1.39E-15	4.16E-18	1124	350	604	868	439
CCL11	-3.48562	-2.28707	-2.6634	-2.49775	-2.85416	4.48E-14	1.93E-07	6.50E-10	2.24E-09	7.98E-12	1125	3141	2510	2536	1728
ELN	-2.70027	-1.77047	-2.40385	-2.32278	-2.60247	4.52E-14	7.35E-10	8.59E-16	8.51E-12	2.06E-14	1126	2123	924	1734	1058
RUNX3	-1.74454	-1.52905	-1.4188	-1.7761	-1.65668	4.59E-14	1.76E-12	1.20E-13	4.79E-15	3.02E-13	1127	1311	1339	975	1336
CNOT1	1.441329	1.552837	1.478011	1.506902	1.555429	4.61E-14	1.00E-13	1.65E-13	2.44E-15	5.40E-16	1128	1035	1392	913	760
BICDL2	2.435974	2.255234	2.686363	2.219458	2.722577	4.61E-14	1.24E-14	1.05E-18	7.39E-20	3.98E-20	1129	848	493	340	253
MRPL12	1.727995	1.740908	1.892472	1.970134	1.798947	4.63E-14	4.78E-16	8.01E-14	2.15E-17	4.88E-16	1130	649	1296	609	752
ATP5PB	1.476566	1.408187	1.314754	1.514332	1.384842	4.64E-14	3.31E-16	4.23E-12	2.09E-17	4.59E-16	1131	628	1760	606	749
NDUFB2	1.536862	1.267069	1.527376	1.416257	1.635527	4.66E-14	4.14E-12	4.33E-15	6.36E-13	3.93E-14	1132	1410	1052	1451	1129
RAB8B	-1.68446	-1.83129	-1.64536	-1.92111	-1.81064	4.66E-14	1.60E-13	3.96E-16	1.07E-14	6.29E-13	1133	1072	867	1041	1431
SLCO4A1	2.266715	2.842186	2.407806	2.964842	2.121812	4.70E-14	3.24E-15	1.28E-16	9.19E-18	1.09E-14	1134	758	785	572	995
DKC1	1.937388	2.018136	1.976665	2.107569	1.723269	4.72E-14	7.82E-14	9.36E-13	2.43E-16	8.49E-12	1135	1017	1585	760	1740
PAPLN	-1.93074	-1.51112	-2.12881	-1.81268	-2.09472	4.85E-14	1.05E-09	9.96E-15	1.66E-10	5.14E-15	1136	2180	1116	2114	923
IER2	1.038442	0.762006	0.736699	1.012654	0.683927	4.93E-14	2.34E-06	1.21E-05	6.99E-11	6.57E-05	1137	3697	4648	2021	4801
EDNRB	-2.49174	-2.3834	-2.66765	-2.73509	-2.65652	4.93E-14	7.71E-14	8.34E-16	1.02E-16	4.29E-13	1138	1016	920	700	1379
P3H1	-2.31638	-2.29081	-2.44449	-2.29208	-2.69747	4.93E-14	4.25E-16	9.51E-19	2.38E-16	4.22E-20	1139	639	487	757	256
SASH3	-1.73186	-1.34639	-1.90284	-1.52239	-1.81087	4.97E-14	3.80E-11	7.24E-18	2.42E-12	2.46E-16	1140	1707	608	1601	711
CYBRD1	-2.03643	-1.91688	-1.81438	-1.99759	-2.01726	4.99E-14	1.90E-14	3.76E-14	6.63E-15	3.55E-18	1141	884	1220	999	433
ID3	-3.26628	-2.98096	-2.87058	-2.9405	-2.66514	5.11E-14	1.49E-11	6.88E-14	4.50E-14	2.63E-09	1142	1576	1277	1167	2586
HSPH1	1.685281	1.560065	1.928926	1.740267	1.940174	5.21E-14	8.16E-14	2.57E-14	1.32E-14	8.04E-15	1143	1022	1187	1062	964
ZDHHC18	-1.21367	-1.17852	-1.55816	-1.25237	-1.62663	5.21E-14	7.24E-15	4.56E-18	1.89E-13	5.58E-19	1144	816	580	1309	349
LPAR6	-1.91977	-2.36507	-2.26236	-2.46824	-1.82381	5.22E-14	1.74E-14	1.86E-14	1.34E-16	4.44E-11	1145	877	1162	720	1941
BMP8B	-2.22369	-1.88508	-2.03576	-1.98201	-2.205	5.24E-14	1.05E-14	1.72E-19	1.65E-15	1.10E-17	1146	840	415	883	504
ZBTB20	-1.51124	-1.21213	-1.29639	-1.20977	-1.56897	5.25E-14	9.70E-12	4.05E-11	4.09E-12	1.16E-13	1147	1526	2082	1656	1236
TAFA5	-2.05719	-1.49792	-1.76991	-1.66503	-1.98694	5.38E-14	3.39E-10	3.86E-16	1.04E-11	1.93E-14	1148	2015	866	1765	1051
IPO7	1.223646	1.160144	1.072885	1.288662	0.920686	5.51E-14	6.72E-14	1.40E-12	5.10E-17	1.87E-07	1149	1003	1627	666	3403
KANK3	-1.1713	-0.88959	-1.39567	-1.07233	-1.15014	5.76E-14	3.21E-08	2.40E-13	3.87E-08	1.83E-10	1150	2749	1440	3000	2139
SLC25A30	-0.77881	-0.55276	-0.67512	-0.61583	-0.74987	5.76E-14	1.64E-07	9.55E-11	6.14E-08	4.36E-12	1151	3098	2210	3071	1657
GFPT1	1.787956	1.667661	2.043207	1.877991	1.932932	5.78E-14	4.37E-15	2.21E-18	2.08E-16	2.58E-18	1152	775	532	745	417
MINDY2	-1.48239	-1.13682	-1.37831	-1.34922	-1.47293	5.80E-14	7.16E-09	6.87E-13	3.25E-13	3.97E-14	1153	2497	1550	1371	1132
RPN1	1.443759	1.183434	1.465817	1.362528	1.120513	5.80E-14	3.11E-11	4.59E-10	1.26E-10	4.51E-09	1154	1671	2454	2091	2674
NRBF2	-0.84092	-0.54125	-0.56174	-0.60732	-0.63993	5.93E-14	3.25E-07	7.88E-08	8.44E-09	4.56E-10	1155	3235	3459	2728	2295
SLCO2A1	-1.90759	-1.32171	-1.55552	-1.23796	-1.46728	6.01E-14	2.12E-09	3.73E-10	6.66E-09	3.84E-10	1156	2286	2420	2697	2259
SERBP1	1.588368	1.57293	1.611564	1.813823	1.569326	6.04E-14	2.91E-16	9.34E-13	3.06E-22	2.07E-15	1157	623	1583	193	852
FSCN1	-3.45422	-3.55995	-3.22814	-3.41233	-3.59994	6.11E-14	7.68E-19	1.51E-16	3.23E-17	1.81E-19	1158	330	801	636	313
SRSF6	1.460604	1.372771	1.404236	1.681607	1.473796	6.11E-14	2.91E-12	9.48E-13	8.38E-14	1.41E-14	1159	1363	1588	1227	1021
CLEC2B	-1.58669	-1.4851	-1.65671	-1.6341	-1.58496	6.12E-14	2.16E-14	1.15E-18	8.48E-16	4.74E-15	1160	895	494	838	919
INTS6L	-0.94217	-0.71723	-0.5888	-0.70989	-0.87471	6.13E-14	2.78E-06	1.25E-05	3.95E-06	3.58E-08	1161	3739	4663	3966	3053
ARL6IP1	1.799668	1.40912	1.617911	1.598219	1.618765	6.19E-14	7.84E-10	1.04E-12	4.42E-10	3.18E-12	1162	2136	1600	2263	1620
STX2	-1.12833	-0.55924	-0.84747	-0.72747	-0.84221	6.24E-14	5.15E-05	4.82E-08	1.47E-07	1.14E-05	1163	4495	3363	3237	4318
PSMB3	1.304729	1.062056	1.520835	1.292686	1.121412	6.31E-14	3.90E-13	2.20E-14	6.31E-14	2.60E-13	1164	1171	1173	1197	1321
PACS1	-1.2491	-1.15233	-1.16805	-1.07869	-1.40906	6.34E-14	2.37E-12	7.13E-16	8.33E-12	3.14E-17	1165	1335	906	1731	561
TWSG1	-1.85575	-1.7857	-1.70558	-1.88703	-1.8658	6.37E-14	2.40E-15	3.40E-14	3.39E-17	6.17E-15	1166	750	1208	639	939
SPHK1	-2.07958	-2.08159	-1.90545	-2.09732	-2.87445	6.41E-14	4.49E-11	2.10E-12	2.27E-12	2.66E-17	1167	1732	1674	1594	553

FAM120A	1.255112	1.13366	1.466164	1.280168	1.225748	6.45E-14	5.33E-10	1.49E-12	2.76E-10	5.14E-13	1168	2076	1637	2192	1399
PTGS1	-2.34445	-2.3413	-2.50562	-2.58227	-2.23691	6.56E-14	2.59E-12	1.10E-15	2.02E-13	1.08E-08	1169	1346	943	1313	2831
SCNN1A	2.355722	2.513035	2.196202	2.54019	2.574765	6.56E-14	3.23E-17	1.92E-16	9.63E-15	1.53E-11	1170	501	816	1027	1818
PCDH17	-2.55992	-2.35461	-2.22088	-2.49793	-2.34367	6.64E-14	4.31E-15	1.28E-14	4.45E-18	3.83E-14	1171	773	1134	528	1127
PLAGL2	1.826032	1.760416	1.363124	1.673497	1.595163	6.70E-14	6.12E-19	7.52E-14	2.82E-12	9.12E-08	1172	320	1294	1619	3262
TUBA4A	1.715249	1.948765	1.733828	1.811244	1.581095	6.71E-14	1.06E-15	1.51E-16	4.28E-17	3.86E-14	1173	702	798	654	1128
CDH6	-1.54053	-1.52208	-1.58495	-1.57611	-1.52223	6.78E-14	1.49E-14	3.01E-17	2.47E-15	4.56E-17	1174	864	687	914	584
TOMM5	1.380522	1.616423	1.112482	1.605591	1.238586	6.81E-14	1.52E-14	2.76E-11	1.57E-15	1.50E-11	1175	867	2029	877	1815
JTB	1.280184	1.029446	0.916518	1.075543	1.192672	7.08E-14	3.44E-11	8.83E-10	1.75E-10	1.36E-11	1176	1685	2560	2123	1806
RTF2	1.141146	0.971092	0.925282	1.10055	0.917551	7.11E-14	2.12E-10	2.73E-12	4.56E-11	2.05E-10	1177	1937	1713	1961	2160
KRTAP5-8	-1.09322	-0.66783	-1.10299	-0.85847	-1.15468	7.12E-14	7.36E-07	2.28E-10	2.30E-07	4.08E-10	1178	3403	2344	3323	2272
B4GALNT3	1.598179	1.737005	1.737545	1.695653	1.532342	7.16E-14	2.19E-14	1.79E-13	2.01E-14	2.09E-10	1179	898	1403	1096	2165
TSPAN13	2.03014	1.730643	1.858898	1.656921	2.201434	7.19E-14	2.24E-13	4.55E-12	4.94E-09	3.61E-13	1180	1107	1769	2656	1357
CLEC3B	-2.15203	-1.85744	-2.0146	-2.09038	-1.92965	7.27E-14	1.14E-16	8.68E-22	3.95E-18	2.77E-14	1181	583	231	515	1090
HEY1	-1.32981	-1.3104	-1.44191	-1.36999	-1.35109	7.44E-14	4.72E-11	1.04E-14	7.22E-13	2.56E-14	1182	1745	1120	1463	1077
SSC5D	-1.95823	-1.735	-2.23015	-1.98301	-2.3687	7.45E-14	3.20E-11	4.86E-12	6.04E-12	1.19E-15	1183	1675	1780	1691	819
KCNJ5	-1.04019	-0.67784	-1.014	-0.87019	-1.05336	7.77E-14	1.81E-06	1.86E-09	2.62E-07	8.80E-12	1184	3632	2683	3351	1745
JPT1	2.034046	1.674745	2.351877	1.658071	1.765696	7.77E-14	6.88E-13	1.41E-16	3.63E-11	1.62E-13	1185	1219	793	1939	1276
AFAP1L2	-2.20183	-2.2078	-1.72696	-2.27811	-1.32373	7.86E-14	8.19E-15	6.06E-11	3.75E-14	7.86E-06	1186	825	2135	1156	4218
F11R	1.872995	1.618269	2.239923	1.919304	2.005669	7.97E-14	2.93E-10	3.81E-16	6.61E-12	4.32E-14	1187	1980	864	1707	1144
HNRNPF	1.644547	1.169773	1.732419	1.501585	1.5666	8.08E-14	4.67E-12	8.34E-14	6.12E-12	2.93E-13	1188	1427	1301	1695	1332
LAG3	-1.74092	-1.37824	-1.64138	-1.9804	-1.55172	8.23E-14	5.43E-09	1.11E-10	5.26E-14	8.58E-10	1189	2455	2230	1180	2405
TCEA2	-1.58191	-1.23731	-1.35719	-1.38399	-1.41483	8.28E-14	3.51E-11	3.53E-13	7.87E-12	8.60E-15	1190	1687	1475	1725	970
RTL8C	-2.77653	-2.75577	-2.14996	-2.32726	-2.69117	8.37E-14	3.34E-16	3.66E-12	8.42E-12	1.41E-13	1191	629	1748	1732	1259
SCPEP1	-1.63615	-1.69949	-1.73422	-1.59645	-2.12053	8.50E-14	5.21E-14	5.41E-15	1.34E-13	2.88E-17	1192	976	1074	1270	558
TRIM31	2.446347	2.498243	2.673919	2.726146	2.70099	9.00E-14	3.30E-15	4.92E-14	7.26E-18	1.05E-15	1193	762	1246	557	809
ARPC1A	1.225102	1.006991	1.253692	0.970267	1.090112	9.02E-14	1.32E-09	2.12E-13	5.25E-10	1.19E-12	1194	2218	1424	2285	1506
CASK	1.643193	2.067608	1.687284	2.167765	1.610743	9.36E-14	9.92E-15	1.83E-13	2.36E-18	9.31E-13	1195	835	1407	498	1471
TRIB2	-2.28964	-1.84274	-1.59327	-1.72617	-1.53722	9.36E-14	9.82E-13	1.01E-10	1.09E-11	6.80E-10	1196	1249	2220	1775	2364
GUCY1B1	-2.2303	-1.98035	-1.96309	-2.08896	-2.27193	9.43E-14	2.39E-18	1.01E-17	3.18E-21	1.97E-17	1197	370	629	255	536
ADGRG1	1.97113	1.763626	1.772427	1.919624	1.920149	9.49E-14	3.72E-13	1.68E-13	2.58E-14	8.29E-15	1198	1164	1397	1117	967
RUSC2	-1.59795	-1.17292	-1.28138	-1.17352	-1.34939	9.62E-14	1.54E-11	2.69E-12	2.13E-13	6.54E-12	1199	1579	1708	1322	1709
MICOS10	1.179547	1.101646	1.253814	1.370398	1.443663	9.70E-14	1.45E-12	8.37E-13	7.26E-14	2.78E-16	1200	1287	1567	1207	720
FZD8	-2.07411	-1.75568	-1.9339	-1.97408	-2.06575	9.71E-14	3.26E-11	2.72E-12	4.78E-15	3.34E-13	1201	1678	1712	974	1348
MCRIP1	-1.48334	-1.40182	-1.385	-1.27759	-1.50466	9.88E-14	5.57E-14	4.37E-10	1.73E-12	1.79E-13	1202	984	2442	1573	1285
ZFP82	-1.10236	-0.73733	-0.97257	-0.86911	-0.71985	9.98E-14	2.15E-08	3.54E-11	4.29E-10	2.96E-05	1203	2677	2068	2257	4561
PRR13	1.586159	1.451088	1.728391	1.528533	1.65471	1.00E-13	1.87E-13	9.94E-17	1.15E-15	4.63E-18	1204	1096	768	855	447
WFDC1	-2.46346	-2.42976	-2.57434	-2.54211	-2.58417	1.01E-13	1.10E-15	1.14E-16	2.90E-19	3.41E-15	1205	704	776	399	886
ECM1	-1.98251	-2.05285	-2.08865	-2.2141	-2.13577	1.01E-13	2.20E-17	1.71E-13	5.31E-17	2.47E-13	1206	472	1398	667	1317
MXRA5	-3.65843	-3.67842	-2.35799	-3.53762	-3.44886	1.01E-13	1.88E-19	3.78E-10	7.96E-16	6.71E-14	1207	278	2423	833	1187
KITLG	-0.98963	-0.86008	-1.01308	-1.0116	-1.00847	1.02E-13	8.53E-10	4.72E-13	9.43E-13	4.90E-13	1208	2149	1507	1490	1391
SFXN3	-1.29357	-1.19342	-1.11652	-1.26587	-1.37511	1.02E-13	1.76E-14	4.82E-11	1.32E-15	2.73E-13	1209	879	2104	863	1326
ELOA2	-1.25883	-1.18909	-1.51999	-1.31303	-1.46033	1.03E-13	4.05E-17	3.26E-17	1.05E-15	9.03E-16	1210	517	691	850	800
H3-3A	1.441443	1.239877	1.489609	1.462733	1.850567	1.05E-13	2.03E-12	1.02E-15	6.81E-14	7.79E-17	1211	1324	937	1205	620
TOMM20	1.548774	1.459388	1.494135	1.683752	1.781685	1.06E-13	4.85E-12	2.62E-11	1.09E-15	1.05E-11	1212	1433	2023	852	1769
PVR	0.998788	1.243019	0.649267	0.984682	0.839018	1.06E-13	5.32E-16	3.60E-07	3.93E-09	6.16E-08	1213	663	3796	2631	3165
MOXD1	-1.66198	-1.35276	-1.51889	-1.72241	-1.51158	1.07E-13	3.17E-12	5.19E-12	6.91E-16	1.35E-14	1214	1374	1788	826	1017
HNRNPM	1.043245	0.994071	0.998819	1.139086	0.951832	1.08E-13	3.80E-11	9.67E-10	4.37E-15	1.12E-10	1215	1706	2572	964	2068
ABHD2	1.519753	1.791679	1.598173	1.789598	1.399459	1.08E-13	1.69E-14	5.55E-13	1.86E-12	3.71E-10	1216	876	1526	1580	2247
AKNA	-1.35486	-1.31837	-1.66388	-1.50953	-1.63161	1.11E-13	2.53E-13	4.26E-15	1.73E-13	1.62E-13	1217	1118	1050	1301	1273
FAM135A	1.354579	1.411909	1.598411	1.548249	1.130996	1.11E-13	8.42E-11	1.60E-15	2.37E-13	2.76E-09	1218	1804	975	1332	2588
VWA5A	-1.55448	-1.1348	-1.0657	-1.13703	-1.1277	1.13E-13	5.21E-09	5.93E-10	1.35E-09	6.62E-11	1219	2446	2495	2450	1993
PKIG	-2.13927	-1.76208	-1.79226	-1.84037	-1.58337	1.14E-13	1.30E-11	7.47E-14	5.91E-11	5.65E-09	1220	1561	1292	1997	2701
ARFGEF2	1.266895	1.472764	1.294699	1.584711	1.116171	1.15E-13	1.78E-15	1.94E-12	1.49E-16	6.75E-10	1221	734	1662	727	2358
SOCS3	-2.38606	-2.52002	-2.55359	-2.52737	-2.79994	1.16E-13	4.46E-13	5.98E-18	5.53E-14	3.68E-15	1222	1185	598	1185	894
ZIK1	-0.99438	-0.86976	-1.09827	-1.16043	-1.09214	1.17E-13	3.07E-10	1.17E-09	2.15E-13	3.18E-12	1223	1993	2614	1324	1621
THBS2	-3.05224	-2.89827	-2.54586	-2.8777	-3.43494	1.17E-13	1.81E-11	6.75E-13	2.53E-12	1.03E-16	1224	1601	1547	1602	646
CDR2L	-1.10748	-0.97943	-0.93805	-0.99158	-0.99322	1.18E-13	3.88E-07	1.01E-07	1.88E-07	1.68E-12	1225	3267	3519	3289	1537
GUCY1A1	-2.67875	-2.36086	-2.18971	-2.31633	-2.48938	1.20E-13	1.40E-15	2.17E-16	1.11E-16	1.12E-16	1226	718	822	704	654
BSPRY	1.323375	1.636705	1.700695	1.624261	1.639983	1.21E-13	1.68E-13	1.12E-15	4.61E-15	5.65E-13	1227	1081	948	969	1411
KPNA2	1.240861	1.029799	1.283151	1.176814	0.732699	1.25E-13	4.88E-11	2.61E-12	1.83E-10	6.90E-05	1228	1748	1700	2132	4818
MRPL18	1.315635	1.211097	1.351949	1.226005	1.015023	1.25E-13	2.63E-13	7.73E-16	3.55E-14	5.43E-10	1229	1121	915	1151	2318
FAP	-2.4292	-2.28143	-2.44702	-2.35496	-2.6084	1.26E-13	3.86E-13	2.56E-16	1.25E-14	5.17E-17	1230	1170	836	1056	592
FNDC1	-2.45298	-2.04958	-2.34634	-2.24177	-2.52699	1.26E-13	3.71E-11	3.35E-14	3.82E-13	1.17E-15	1231	1700	1205	1393	814
SLC31A2	-1.29113	-1.09577	-1.18428	-1.38957	-1.39637	1.26E-13	6.02E-10	1.82E-11	7.22E-13	2.34E-14	1232	2097	1955	1464	1070
KIAA0930	-2.04096	-1.36821	-1.39641	-1.61666	-1.68104	1.26E-13	1.34E-12	1.68E-14	1.76E-14	2.82E-14	1233	1277	1152	1085	1093
LAMC2	2.458251	2.320702	2.763553	2.474944	2.768674	1.28E-13	1.85E-09	2.81E-13	6.16E-12	2.37E-16	1234	2261	1455	1696	709
CCT2	1.565396	1.471816	1.588394	1.738047	1.700661	1.28E-13	3.05E-14	9.38E-13	2.24E-17	4.88E-16	1235	931	1586	613	751
RGS16	-1.21652	-1.3436	-1.40578	-1.38502	-1.45996	1.30E-13	4.57E-09	2.28E-10	8.47E-09	4.36E-14	1236	2417	2345	2730	1145
CRYAB	-1.8978	-1.78866	-2.04835	-1.85258	-2.27394	1.30E-13	1.25E-11	6.98E-17	1.16E-13	1.92E-12	1237	1553	740	1255	1548
C1orf198	-0.94609	-0.97044	-0.80372	-1.05076	-0.8887	1.31E-13	2.53E-12	3.47E-10	7.45E-14	1.56E-11	1238	1342	2412	1212	1821
EIF2S2	1.725867	1.932529	1.717137	2.00373	1.218238	1.31E-13	4.45E-15	4.64E-15	2.18E-14	1.17E-07	1239	777	1059	1104	3311
TCEAL3	-1.32256	-0.93584	-0.93818	-0.83623	-1.23107	1.33E-13	4.29E-11	1.51E-11	1.09E-10	1.67E-13	1240	1726	1923	2072	1278

SPSB1	-2.06131	-1.49675	-1.76242	-1.56195	-1.94976	1.40E-13	1.76E-10	1.03E-11	8.66E-11	2.11E-14	1241	1908	1877	2048	1061
TIMM13	1.430197	1.560005	1.472014	1.612513	1.480674	1.43E-13	6.62E-12	4.40E-12	3.26E-15	1.46E-14	1242	1475	1764	932	1022
FAM219A	-1.02595	-0.74084	-0.65103	-0.64935	-0.74715	1.45E-13	3.20E-08	2.37E-07	1.33E-09	5.96E-08	1243	2748	3695	2447	3161
CDC37	1.079401	1.004812	1.057464	1.155765	1.13341	1.46E-13	3.15E-10	1.65E-10	3.97E-15	1.88E-11	1244	2000	2295	957	1851
BZW2	2.00915	2.106168	2.174922	2.195763	1.925575	1.47E-13	2.30E-13	3.46E-15	1.15E-13	9.09E-15	1245	1109	1036	1254	976
MAPK13	1.789944	1.302809	1.756228	1.287304	1.972926	1.50E-13	9.26E-11	3.38E-17	1.24E-09	5.41E-13	1246	1818	692	2424	1404
GREM1	-3.71188	-3.32092	-2.71996	-3.77457	-3.61579	1.52E-13	1.36E-11	9.22E-13	3.53E-15	3.98E-14	1247	1565	1578	941	1133
TCP10L3	-0.78997	-0.67003	-0.95395	-0.91147	-1.14395	1.54E-13	3.90E-11	8.21E-08	2.36E-09	1.39E-09	1248	1711	3466	2544	2481
ADAMTSL	-1.50864	-1.01885	-1.54787	-1.14279	-1.62399	1.58E-13	4.53E-06	6.12E-11	2.11E-06	2.84E-11	1249	3839	2137	3816	1891
RAD21	1.310766	1.307001	1.674502	1.317381	1.354053	1.59E-13	1.96E-12	1.02E-14	8.86E-11	7.42E-15	1250	1319	1119	2054	956
ACVRL1	-2.17436	-2.13746	-1.9272	-2.14172	-1.4384	1.60E-13	9.10E-15	4.51E-12	3.07E-14	1.22E-07	1251	827	1767	1137	3319
SHROOM3	1.416372	1.226181	1.488509	1.310129	1.485034	1.60E-13	7.72E-11	4.88E-14	1.32E-09	1.92E-11	1252	1792	1242	2440	1854
RBPMS	-2.51124	-2.60446	-2.37952	-2.61434	-2.3254	1.60E-13	1.99E-18	2.67E-14	9.53E-18	1.03E-14	1253	359	1189	574	984
TTN	-1.06589	-0.97958	-0.95623	-0.90169	-1.10067	1.60E-13	6.36E-11	2.62E-11	2.74E-09	9.86E-14	1254	1773	2022	2565	1219
PAG1	-2.52548	-2.1207	-1.96571	-2.44276	-2.2322	1.61E-13	1.91E-12	6.45E-13	1.46E-18	1.02E-11	1255	1315	1543	472	1766
FADS3	-1.71834	-1.20121	-1.647	-1.47987	-1.88179	1.67E-13	6.34E-12	1.53E-17	3.90E-14	5.96E-15	1256	1469	652	1158	934
TMPO	1.253796	1.10502	1.267361	1.148705	0.94985	1.67E-13	1.36E-11	5.68E-14	8.71E-12	4.17E-09	1257	1566	1258	1737	2657
SREBF2	1.281881	1.4687	1.768721	1.478982	0.942588	1.70E-13	7.72E-12	9.33E-17	1.95E-11	1.71E-05	1258	1502	763	1861	4432
MYC	2.881396	3.187365	3.016195	3.201771	2.764739	1.72E-13	1.52E-16	1.20E-13	1.52E-16	1.41E-12	1259	593	1338	729	1528
ZCCHC24	-2.08498	-1.91084	-1.98386	-1.82936	-2.14321	1.73E-13	2.12E-13	5.15E-13	3.53E-11	7.83E-15	1260	1103	1516	1933	962
FUT4	1.865584	1.680468	2.023062	1.979238	1.880125	1.74E-13	1.64E-13	2.86E-16	8.34E-17	2.94E-13	1261	1076	847	689	1333
DDX39A	1.401511	1.511449	1.424948	1.426839	0.925987	1.74E-13	5.15E-14	2.21E-13	1.03E-14	3.29E-07	1262	973	1428	1036	3524
MACROH2	1.712523	1.423212	1.77417	1.750346	1.76209	1.74E-13	2.43E-14	1.72E-14	1.45E-16	4.44E-17	1263	911	1154	724	582
CTSF	-1.66322	-1.31459	-1.57421	-1.55446	-1.7731	1.76E-13	2.09E-09	7.71E-17	2.84E-12	1.18E-15	1264	2284	750	1621	815
C4orf3	-1.38222	-1.36358	-0.94564	-1.21429	-0.918	1.80E-13	1.36E-15	2.84E-09	9.68E-12	6.95E-08	1265	716	2775	1755	3196
LSM5	1.726348	1.567213	1.845704	1.541783	1.507073	1.82E-13	6.00E-13	2.74E-16	9.92E-13	4.26E-13	1266	1209	843	1501	1378
ESRP2	2.130191	2.261008	1.650296	2.061639	1.961101	1.82E-13	2.12E-16	3.22E-07	1.24E-10	7.99E-10	1267	610	3775	2089	2388
MXRA7	-2.02718	-2.12867	-2.33943	-2.03094	-2.48672	1.82E-13	3.49E-13	8.24E-19	1.06E-14	8.62E-20	1268	1159	475	1040	286
ILF2	1.327026	1.114438	1.214102	1.263738	1.380085	1.87E-13	1.43E-11	5.48E-11	1.30E-15	5.65E-14	1269	1570	2120	861	1168
SKI	-1.40501	-1.4205	-1.05959	-1.3292	-1.09222	1.91E-13	3.17E-12	1.57E-09	2.94E-10	4.57E-11	1270	1373	2647	2205	1946
PSMD3	1.224776	1.349613	1.310967	1.419168	1.397006	1.92E-13	1.35E-11	1.92E-11	3.59E-17	9.54E-13	1271	1563	1969	647	1477
LURAP1	-1.16485	-0.95429	-1.1306	-1.3194	-1.19023	1.92E-13	3.54E-09	1.62E-08	8.04E-13	1.10E-09	1272	2379	3119	1474	2444
MRPS24	1.162894	1.028019	1.05029	1.072695	1.180826	1.94E-13	3.77E-10	1.85E-10	1.39E-12	7.72E-12	1273	2031	2311	1543	1723
H4C2	1.926477	1.726486	1.960183	1.921898	2.204917	1.94E-13	9.19E-13	2.97E-15	4.68E-14	1.69E-16	1274	1247	1017	1170	676
PPIA	2.064864	1.75427	1.985212	1.961975	2.177047	1.94E-13	4.46E-13	1.61E-12	3.91E-15	1.04E-16	1275	1184	1646	956	647
SMAP2	-1.53077	-1.17413	-1.62343	-1.32949	-1.98418	1.95E-13	5.94E-11	1.17E-16	3.85E-12	8.12E-16	1276	1767	778	1648	791
TPM2	-3.38867	-3.61766	-4.06182	-3.66593	-4.08012	1.96E-13	4.67E-15	4.32E-21	5.94E-17	9.28E-21	1277	780	291	673	211
PLD4	-0.92205	-0.65051	-0.96666	-0.8215	-0.88706	1.97E-13	3.20E-09	1.18E-13	1.83E-10	3.28E-11	1278	2363	1336	2133	1904
TRIM28	1.542276	1.541755	1.532805	1.615729	1.606775	1.99E-13	4.99E-14	4.28E-13	1.05E-15	1.41E-13	1279	969	1498	849	1258
CCT3	1.738939	1.543859	1.427241	1.678045	1.738975	2.00E-13	4.09E-12	3.36E-12	2.81E-13	9.28E-15	1280	1409	1734	1356	977
MCM3	1.516953	1.619211	1.458052	1.621934	1.617036	2.05E-13	2.13E-15	8.47E-15	1.20E-19	8.02E-19	1281	743	1111	360	368
HNRNPA2	1.567569	1.163895	1.388808	1.28064	1.606546	2.10E-13	8.97E-10	3.29E-11	6.91E-10	1.28E-13	1282	2158	2059	2337	1249
LBR	1.635182	1.899693	1.604433	1.662933	1.669309	2.16E-13	6.70E-16	5.95E-14	1.82E-14	6.02E-15	1283	672	1263	1089	936
VPS25	1.117172	1.158569	1.127853	1.26351	1.091829	2.18E-13	1.29E-14	3.98E-16	4.07E-19	1.30E-15	1284	849	869	418	825
FAM3D	2.849385	2.813579	3.252412	3.220896	3.273117	2.20E-13	8.10E-14	5.27E-17	7.78E-18	2.83E-13	1285	1021	716	559	1328
AK2	1.269655	1.606818	1.492906	1.682996	1.556052	2.26E-13	2.16E-14	5.35E-14	3.44E-19	2.28E-16	1286	896	1254	404	704
SCD	2.489477	2.459871	2.415297	2.556822	2.402683	2.26E-13	5.25E-12	3.80E-12	1.04E-12	3.88E-11	1287	1446	1750	1511	1925
SLC25A10	1.475723	1.469133	1.506548	1.548259	1.600369	2.27E-13	4.78E-15	3.15E-16	6.60E-17	1.46E-16	1288	782	852	678	668
DNPEP	1.25005	1.272474	1.450185	1.506182	1.405272	2.29E-13	1.69E-14	6.39E-17	3.16E-15	2.74E-16	1289	875	729	930	718
VAT1	-1.78377	-1.72344	-1.5477	-1.53394	-1.85116	2.29E-13	1.16E-14	8.66E-12	1.48E-13	3.15E-14	1290	843	1849	1278	1107
IL15RA	-1.12441	-0.85706	-0.86987	-0.91715	-1.01148	2.30E-13	2.22E-09	9.81E-12	1.01E-10	3.37E-13	1291	2294	1872	2065	1349
DEPP1	-1.90572	-1.92309	-1.89754	-2.05588	-1.37424	2.30E-13	5.48E-13	2.60E-16	2.75E-12	1.26E-06	1292	1203	839	1616	3785
RPS26	1.526499	1.367388	1.183985	1.398511	1.593078	2.31E-13	6.57E-12	9.37E-11	1.61E-13	4.29E-15	1293	1474	2203	1290	909
MS4A4A	-1.60838	-1.60084	-1.63623	-1.73218	-1.87358	2.33E-13	6.49E-13	7.04E-15	5.47E-13	5.98E-19	1294	1215	1094	1432	355
RAPGEFL1	1.819737	1.963973	2.101533	1.679547	1.919454	2.34E-13	1.26E-15	3.96E-16	6.85E-17	1.77E-13	1295	711	868	680	1283
S1PR3	-2.05657	-1.79771	-1.89041	-1.97934	-2.12587	2.35E-13	1.24E-10	2.54E-14	3.24E-11	1.34E-15	1296	1859	1186	1917	827
PPP1R14B	1.14423	1.342971	1.183509	1.424169	1.317116	2.35E-13	9.58E-15	5.24E-12	1.93E-20	7.22E-15	1297	832	1790	305	952
MOB3A	-1.76697	-1.64818	-1.82321	-1.76689	-1.97228	2.36E-13	1.62E-13	2.08E-16	7.00E-15	4.01E-18	1298	1074	819	1003	437
S100A6	3.063661	2.887767	3.471163	3.052159	2.886456	2.43E-13	2.54E-17	2.26E-16	4.24E-18	6.87E-13	1299	482	828	521	1442
TECR	1.633209	1.675312	1.684427	1.709782	1.612888	2.43E-13	2.36E-13	4.93E-13	2.31E-13	7.21E-17	1300	1112	1510	1329	618
ITGB3	-2.35775	-2.03063	-2.5201	-2.33562	-2.37252	2.43E-13	8.97E-10	1.13E-12	8.74E-11	2.65E-11	1301	2159	1611	2051	1881
LYSMD4	-0.75513	-0.69082	-0.92121	-0.84242	-1.08375	2.44E-13	1.30E-12	4.16E-10	7.65E-11	1.59E-12	1302	1275	2436	2034	1535
FUT3	2.502615	1.969706	2.621733	2.324371	2.33259	2.44E-13	1.69E-09	8.41E-16	1.54E-12	1.02E-16	1303	2245	923	1560	643
NFIC	-2.00402	-1.90324	-1.74396	-1.95056	-1.77323	2.45E-13	9.64E-16	1.02E-15	3.26E-15	1.10E-12	1304	696	936	933	1498
MPST	1.37919	1.614368	1.525728	1.745939	1.644879	2.46E-13	3.09E-14	2.77E-14	2.14E-15	9.02E-15	1305	933	1192	905	975
LILRA5	-1.557	-1.5218	-1.73275	-1.8196	-1.61802	2.48E-13	1.28E-11	6.91E-16	3.61E-16	1.06E-11	1306	1557	901	780	1771
CLIC1	2.064222	1.458885	2.113213	1.723311	2.113189	2.50E-13	1.34E-10	1.01E-15	1.85E-10	2.78E-16	1307	1869	932	2136	721
ACTN4	1.567187	1.183585	1.79368	1.43068	1.645583	2.51E-13	3.88E-06	2.89E-10	6.62E-07	9.28E-14	1308	3813	2377	3561	1213
REPIN1	1.477852	1.633678	1.474764	1.538557	1.101805	2.51E-13	5.23E-15	2.72E-13	2.14E-15	2.31E-09	1309	789	1452	904	2571
ABHD17C	1.719835	1.51086	1.889358	1.754142	1.813719	2.51E-13	5.34E-14	1.46E-15	1.25E-15	2.30E-15	1310	979	966	860	857
RAN	1.690097	1.670993	1.565589	1.787338	1.747578	2.51E-13	6.38E-14	6.93E-12	9.62E-18	1.22E-16	1311	998	1827	575	657
HOPX	-0.87461	-0.71674	-0.89495	-0.76171	-1.06814	2.51E-13	5.42E-10	3.77E-14	3.73E-10	7.29E-16	1312	2080	1222	2239	778
RPS15	2.076858	1.954907	1.813006	2.208903	1.801313	2.53E-13	9.47E-13	3.40E-11	1.16E-17	1.80E-11	1313	1248	2064	584	1843

SGPP1	-1.09977	-0.84094	-1.0409	-0.9809	-1.06284	2.56E-13	1.94E-11	8.95E-15	1.97E-12	3.41E-08	1314	1612	1112	1584	3044
TOP2A	1.71321	1.47294	1.46721	1.467608	0.998687	2.56E-13	1.45E-12	6.34E-14	2.41E-12	2.96E-06	1315	1288	1270	1600	3973
ECSCR	-2.50694	-2.07593	-2.2538	-2.27625	-2.40182	2.60E-13	2.01E-11	6.56E-15	4.26E-12	1.10E-11	1316	1613	1086	1661	1779
CCDC159	-1.08606	-0.81056	-1.27828	-1.06837	-1.27976	2.62E-13	8.76E-12	7.36E-12	1.48E-09	7.28E-12	1317	1513	1832	2471	1717
NDC1	1.051767	1.097019	1.164913	1.042709	0.772992	2.65E-13	1.46E-12	4.23E-16	1.10E-14	1.84E-07	1318	1290	870	1044	3398
NLGN2	-1.96115	-1.7222	-2.21738	-1.80202	-2.48768	2.69E-13	6.75E-10	6.26E-15	5.79E-10	9.94E-18	1319	2108	1083	2302	498
NAPSA	-1.13093	-0.71303	-1.0395	-0.90368	-0.83254	2.70E-13	2.43E-07	2.26E-10	2.41E-10	1.56E-06	1320	3182	2343	2177	3831
ECI1	1.094639	1.179748	1.300551	1.102541	0.983764	2.71E-13	1.04E-10	6.81E-12	9.94E-12	4.02E-09	1321	1837	1824	1759	2653
ZNF707	-1.71667	-1.51806	-1.37903	-1.37326	-1.48262	2.73E-13	9.47E-11	2.68E-10	1.22E-10	2.38E-10	1322	1827	2365	2086	2190
SLC39A5	1.949927	1.877915	1.79136	1.859823	1.671653	2.74E-13	1.96E-09	2.09E-11	1.30E-09	1.06E-12	1323	2271	1986	2437	1492
CXCL12	-1.46999	-1.08776	-1.31264	-1.3066	-1.07878	2.80E-13	2.71E-09	1.18E-12	4.77E-11	2.31E-07	1324	2328	1613	1967	3451
DEFB131A	-0.92266	-0.52183	-0.69365	-0.56536	-0.70548	2.81E-13	2.67E-05	2.86E-09	6.75E-06	1.98E-05	1325	4298	2776	4094	4462
FA2H	1.459454	1.290423	1.627872	1.543449	1.12453	2.83E-13	9.20E-11	1.51E-13	7.79E-12	1.52E-07	1326	1817	1379	1723	3353
TUB	-0.96414	-0.75505	-0.59126	-0.8345	-0.6745	2.85E-13	2.12E-08	8.54E-07	2.22E-10	4.66E-08	1327	2676	3996	2164	3113
SMG6	-1.09016	-0.82931	-0.84854	-1.01149	-1.01437	2.86E-13	1.01E-08	2.44E-08	2.83E-11	9.85E-07	1328	2554	3204	1904	3734
LMNB2	1.404725	1.620182	1.606816	1.656626	1.667251	2.86E-13	6.34E-14	2.00E-13	9.21E-17	1.38E-19	1329	997	1416	693	304
RUFY3	-1.10901	-0.94435	-0.79986	-0.9212	-0.68547	2.89E-13	1.66E-09	3.90E-06	4.31E-08	1.86E-07	1330	2243	4332	3018	3400
KIAA0754	-1.42511	-1.13054	-1.13882	-1.13508	-1.07603	2.90E-13	6.06E-12	2.64E-12	1.98E-10	4.14E-08	1331	1466	1702	2148	3082
PDZK1IP1	2.766919	2.574835	3.457929	2.797289	3.013057	2.92E-13	2.96E-13	6.05E-17	2.02E-17	2.10E-16	1332	1129	725	604	695
ZNF705A	-1.28739	-1.09457	-1.53323	-1.27255	-1.32295	2.95E-13	8.26E-12	4.92E-21	8.60E-13	3.60E-18	1333	1510	299	1481	435
DNAJB1	1.295314	1.519743	1.581522	1.67861	1.389702	2.98E-13	3.48E-14	7.32E-16	1.04E-16	8.15E-13	1334	938	910	701	1459
KAT2B	-1.1974	-1.01234	-1.59944	-1.3332	-1.45151	3.02E-13	2.71E-10	4.42E-14	2.37E-11	7.06E-12	1335	1968	1231	1887	1714
SPART	-2.18061	-1.65247	-1.95802	-1.7255	-2.23199	3.04E-13	5.00E-10	5.22E-14	1.49E-11	3.10E-16	1336	2066	1252	1824	727
COX8A	1.758549	1.585281	1.916471	1.81232	1.746296	3.04E-13	9.35E-12	1.15E-13	2.96E-13	3.60E-15	1337	1522	1333	1358	891
DIP2C	-1.35233	-0.90848	-1.03821	-0.92322	-1.30166	3.07E-13	2.19E-07	2.46E-09	8.28E-08	3.78E-13	1338	3166	2749	3131	1365
MCM4	1.777514	1.789124	1.910431	1.852935	1.654844	3.09E-13	1.72E-15	1.00E-16	8.51E-14	5.73E-11	1339	733	769	1228	1965
NSUN2	0.946212	1.062465	0.84271	0.997272	0.718792	3.10E-13	3.36E-13	6.41E-13	6.50E-12	3.44E-10	1340	1153	1541	1703	2239
ATIC	1.516377	1.960938	1.690748	2.068623	1.407984	3.11E-13	3.49E-13	2.02E-12	3.02E-15	6.57E-14	1341	1160	1668	926	1183
ATP5MJ	1.374858	1.501694	1.368715	1.40173	1.312673	3.11E-13	1.61E-12	1.07E-13	3.64E-14	3.20E-12	1342	1301	1325	1152	1623
CD37	-1.70847	-1.28031	-1.59425	-1.47647	-1.74346	3.11E-13	3.84E-08	1.32E-10	2.40E-08	3.05E-14	1343	2790	2260	2917	1102
EIF3C	1.69051	1.226704	1.37498	1.67329	1.663988	3.20E-13	4.79E-09	4.75E-09	5.67E-14	3.09E-11	1344	2428	2866	1187	1897
DENND1C	-1.28615	-1.16149	-1.7022	-1.38283	-1.9474	3.38E-13	3.40E-13	3.53E-19	4.72E-13	7.17E-17	1345	1155	444	1417	616
PIGT	1.778218	1.354015	1.842592	1.487042	1.653886	3.49E-13	2.38E-10	1.02E-15	1.72E-10	5.60E-13	1346	1953	938	2120	1407
HLA-DQA2	-1.85829	-1.64537	-2.06648	-1.74604	-1.86398	3.51E-13	1.01E-12	1.01E-15	8.77E-14	4.97E-13	1347	1254	931	1231	1394
CD68	-2.31603	-3.09212	-2.5529	-2.71681	-2.65269	3.55E-13	6.70E-17	1.54E-13	5.43E-16	9.63E-13	1348	541	1382	806	1478
TRAPPC9	1.258548	1.316958	1.269443	1.355986	1.169406	3.59E-13	8.62E-13	1.13E-13	7.66E-12	8.84E-16	1349	1241	1332	1719	799
PHB	1.864209	1.955616	1.863878	1.932645	1.98742	3.65E-13	3.02E-16	3.31E-14	8.02E-18	3.43E-18	1350	625	1202	561	430
H4C15	2.364582	1.876828	2.239208	2.189284	2.423095	3.70E-13	4.47E-12	1.40E-12	2.94E-14	9.10E-17	1351	1419	1630	1130	634
FECH	-0.98049	-0.72668	-0.89668	-0.8588	-0.9557	3.76E-13	9.00E-09	1.41E-11	1.28E-08	1.37E-13	1352	2535	1914	2806	1254
CHST14	-0.97787	-0.67186	-1.03502	-0.89988	-1.01697	3.80E-13	6.15E-09	2.73E-09	7.59E-10	2.83E-11	1353	2477	2768	2353	1890
HEPH	1.395421	1.381802	1.344393	1.561101	1.055439	3.80E-13	5.79E-11	4.54E-11	8.58E-13	8.86E-08	1354	1764	2093	1480	3254
CFL2	-1.42139	-1.48136	-1.52457	-1.78929	-1.54688	3.80E-13	4.12E-13	1.70E-15	1.57E-15	1.04E-14	1355	1177	979	875	989
SLC2A6	-1.34022	-1.05384	-1.37717	-1.23759	-1.18491	3.80E-13	3.58E-06	4.42E-09	3.35E-07	1.81E-07	1356	3790	2856	3405	3392
SNRPA	1.11639	1.242153	1.171698	1.179155	1.191466	3.86E-13	1.54E-12	2.48E-12	3.35E-12	2.14E-16	1357	1294	1690	1632	697
TRPM3	-0.8357	-0.67048	-0.9098	-0.83124	-0.84644	3.88E-13	4.04E-09	3.51E-12	1.17E-10	1.77E-11	1358	2401	1742	2080	1840
CXCL3	2.19833	1.624799	2.271499	2.010516	1.831545	4.01E-13	1.16E-06	1.82E-09	3.25E-11	7.57E-08	1359	3520	2679	1919	3220
DNAJA2	0.911795	0.704118	0.876126	0.76915	0.993981	4.02E-13	4.63E-09	5.26E-12	8.18E-10	1.34E-10	1360	2422	1791	2366	2094
ZNF768	1.024094	1.091961	1.14192	0.914559	1.102259	4.05E-13	1.84E-09	2.03E-12	6.26E-10	1.38E-09	1361	2260	1669	2319	2479
MROH1	1.620215	1.933273	1.855715	1.927761	1.450003	4.12E-13	1.16E-12	2.43E-15	3.88E-12	2.94E-13	1362	1260	1005	1650	1334
KLF3	1.163524	1.123205	1.620185	1.166163	1.108804	4.20E-13	1.48E-14	1.23E-17	6.29E-12	4.83E-09	1363	863	644	1700	2677
NELFCD	1.901476	1.977277	1.503477	2.127961	1.618567	4.20E-13	2.08E-14	4.97E-12	1.62E-17	1.27E-13	1364	890	1784	594	1247
EIF4G1	1.17793	0.930085	1.033991	1.005962	1.221238	4.31E-13	8.80E-09	3.16E-10	1.07E-08	2.30E-13	1365	2534	2390	2773	1311
SFN	2.462168	2.657565	3.009411	2.759755	2.217552	4.31E-13	2.24E-13	7.32E-16	3.52E-12	1.55E-14	1366	1106	908	1635	1025
OAS1	1.978066	2.172665	1.835877	2.420501	1.154095	4.32E-13	1.19E-12	1.26E-11	9.62E-16	0.0001	1367	1267	1903	842	4929
SGCB	-1.95166	-1.50815	-1.43827	-1.54905	-1.59184	4.37E-13	8.49E-13	1.69E-11	1.68E-12	6.95E-11	1368	1238	1937	1569	2004
NQO1	2.380355	2.227557	2.392017	2.654367	2.273792	4.38E-13	8.84E-10	1.19E-13	1.55E-13	1.35E-16	1369	2155	1337	1285	664
IQGAP3	1.442694	1.182997	0.673002	1.001512	0.820026	4.38E-13	1.36E-13	0.002229	2.61E-05	0.000254	1370	1062	6245	4468	5190
CST1	-3.0947	-2.571	-2.3531	-2.61649	-2.43338	4.41E-13	1.27E-08	7.39E-10	1.04E-10	8.67E-10	1371	2598	2531	2066	2407
PSMD8	0.754599	0.882074	0.863638	0.900312	0.821844	4.51E-13	6.25E-12	8.03E-14	4.16E-13	9.39E-12	1372	1468	1297	1401	1753
FAM89B	-1.03182	-0.97228	-0.84542	-0.88111	-1.1282	4.54E-13	3.21E-12	9.90E-11	2.11E-10	2.38E-14	1373	1376	2217	2158	1072
CD84	-1.54132	-1.56033	-1.60437	-1.73913	-2.08985	4.56E-13	2.00E-12	8.11E-15	1.60E-14	4.23E-19	1374	1322	1105	1074	339
PRKCSH	1.171694	0.841502	0.94685	0.821498	0.840203	4.64E-13	1.47E-07	8.32E-09	4.36E-07	1.13E-09	1375	3081	2980	3466	2447
PIM2	-2.45111	-2.00195	-3.26364	-2.3217	-2.80773	4.74E-13	5.96E-12	3.73E-18	4.29E-10	2.65E-13	1376	1464	567	2255	1324
TSC1	-0.76314	-0.68532	-0.6349	-0.78092	-0.70486	4.77E-13	7.32E-10	8.89E-09	1.59E-12	6.76E-10	1377	2122	2991	1563	2362
SINHCAF	1.281288	1.395625	1.303696	1.385675	1.172009	4.79E-13	4.80E-15	1.47E-13	1.81E-14	1.35E-12	1378	783	1373	1087	1519
MFSD1	-0.89891	-1.00629	-1.05425	-0.98593	-0.81041	4.82E-13	3.04E-12	7.29E-14	8.05E-11	5.31E-09	1379	1367	1286	2041	2694
APOL3	-1.28798	-1.10999	-1.5632	-1.33168	-1.60016	4.84E-13	5.12E-12	1.27E-19	3.08E-12	1.18E-18	1380	1443	403	1629	385
NOX1	2.656332	2.406122	2.507974	2.212782	2.357433	4.90E-13	3.19E-11	8.17E-10	4.43E-12	9.88E-11	1381	1674	2548	1667	2051
MCM7	1.577477	1.636115	1.523008	1.615973	1.724171	4.99E-13	2.68E-12	6.67E-12	1.27E-12	2.25E-15	1382	1353	1816	1533	855
BCAM	-1.80462	-1.50889	-1.31551	-1.6811	-1.21014	5.09E-13	9.32E-10	1.25E-10	3.27E-13	3.33E-08	1383	2165	2250	1373	3039
PLEKHM2	-1.40527	-1.3922	-1.12762	-1.1932	-1.27964	5.12E-13	5.55E-15	2.57E-12	5.09E-11	9.73E-12	1384	792	1695	1980	1760
ARID5B	-1.57988	-1.47382	-1.50303	-1.56498	-1.30914	5.14E-13	8.06E-11	2.70E-17	3.30E-11	5.68E-10	1385	1795	684	1921	2327
ACP1	1.138247	1.079749	1.082747	1.143383	1.221037	5.23E-13	5.99E-13	1.36E-13	2.33E-12	6.01E-16	1386	1208	1364	1595	766

CMTM2	-1.06027	-1.03292	-1.2237	-1.09713	-1.23105	5.41E-13	1.08E-12	1.51E-16	9.68E-12	4.33E-16	1387	1259	800	1754	745
PRSS53	-0.84717	-0.68693	-0.71771	-0.74147	-0.70451	5.41E-13	2.02E-11	1.39E-13	2.26E-12	2.43E-12	1388	1614	1366	1592	1582
IL18BP	-1.39495	-1.57788	-1.60416	-1.57866	-1.38987	5.44E-13	3.96E-13	7.43E-14	9.40E-13	4.79E-12	1389	1174	1290	1489	1667
PRELID1	1.351462	1.403358	1.246466	1.324472	1.126524	5.44E-13	3.51E-17	3.91E-17	6.60E-17	6.75E-11	1390	506	698	677	1997
CLUH	1.461977	1.711028	1.63766	1.705956	1.777645	5.46E-13	4.99E-15	1.11E-14	1.04E-17	4.71E-17	1391	785	1124	580	588
THEMIS2	-2.1816	-2.14219	-2.3223	-2.31938	-2.39999	5.46E-13	6.66E-15	2.70E-13	5.85E-12	2.41E-13	1392	808	1451	1689	1316
AIFM1	1.542027	1.78757	1.451142	2.035465	1.551656	5.47E-13	2.91E-14	2.36E-12	4.01E-19	4.70E-12	1393	928	1684	417	1663
PRELID3B	1.008302	1.070993	0.9084	1.212903	1.219925	5.61E-13	1.78E-13	3.18E-13	3.32E-16	4.05E-15	1394	1090	1467	777	903
EVPL	2.188686	2.122934	1.762085	2.133483	1.559732	5.63E-13	1.05E-13	2.70E-12	6.57E-14	2.58E-10	1395	1040	1710	1202	2202
PDCD6	1.361525	1.185224	1.640658	1.232967	1.415122	5.63E-13	1.11E-10	2.73E-15	1.79E-10	6.68E-16	1396	1843	1014	2130	770
PPP1R9B	-0.92262	-1.12703	-1.04716	-1.06322	-0.96094	5.74E-13	2.52E-12	3.68E-13	4.50E-11	1.57E-10	1397	1341	1479	1959	2115
C5orf24	-1.03745	-0.76405	-0.60218	-0.70334	-0.74297	5.75E-13	6.56E-08	1.78E-05	3.82E-06	2.60E-08	1398	2896	4751	3949	2989
OR52N4	-0.96614	-0.68752	-0.96813	-0.91101	-0.84626	5.80E-13	1.90E-06	1.64E-08	1.46E-08	1.16E-10	1399	3647	3121	2825	2073
ODF3B	-1.52934	-1.39225	-1.49819	-1.65284	-1.26097	5.84E-13	1.54E-10	3.11E-12	3.66E-15	2.51E-10	1400	1891	1729	947	2199
KIF7	-1.16786	-0.82342	-1.07651	-0.99352	-1.12813	5.90E-13	5.75E-09	4.42E-10	2.36E-08	2.26E-09	1401	2464	2446	2914	2562
OR4F6	-0.80827	-0.79976	-0.90824	-0.98805	-0.66883	5.93E-13	2.51E-09	2.87E-09	1.32E-10	3.44E-06	1402	2311	2777	2097	4015
SLFN12L	-1.13249	-0.68416	-1.27643	-0.91452	-0.83909	5.93E-13	8.10E-08	1.50E-13	2.33E-08	0.000253	1403	2946	1377	2911	5189
RENBP	-1.27255	-1.30636	-1.6819	-1.5655	-1.5301	5.93E-13	6.02E-19	1.38E-18	8.10E-18	2.38E-12	1404	318	505	564	1580
PARD6B	1.969257	1.919631	1.71416	1.924287	1.456469	5.93E-13	3.49E-14	3.71E-13	3.89E-13	1.25E-08	1405	939	1481	1396	2862
TTYH2	-1.11839	-0.66312	-1.14823	-0.88172	-1.28412	6.00E-13	2.47E-06	1.22E-11	1.52E-07	8.49E-11	1406	3707	1899	3245	2024
TSPYL2	-1.09015	-0.9252	-1.13583	-1.04882	-1.21593	6.02E-13	5.33E-12	2.02E-12	8.04E-12	4.39E-15	1407	1449	1667	1728	912
SSTR2	-0.75216	-0.51736	-0.68293	-0.65493	-0.68547	6.03E-13	8.87E-06	3.36E-08	2.53E-08	1.92E-08	1408	4008	3292	2923	2934
CARM1	0.895966	0.943643	1.005717	1.015105	0.920975	6.10E-13	1.80E-12	9.77E-14	6.17E-14	1.46E-10	1409	1313	1314	1195	2107
LOXL1	-2.56891	-2.31344	-2.14595	-2.34563	-2.57145	6.27E-13	4.01E-12	1.90E-11	2.41E-13	5.58E-14	1410	1407	1968	1336	1165
PELO	-1.13193	-1.03997	-1.00785	-1.04711	-0.80016	6.35E-13	1.17E-13	1.29E-11	2.18E-12	2.12E-08	1411	1049	1905	1591	2953
CBX3	1.397123	1.22985	1.413467	1.327399	1.525591	6.37E-13	5.94E-12	1.71E-11	2.38E-12	3.99E-14	1412	1463	1940	1597	1134
DLL1	-2.79522	-2.70022	-2.60627	-2.82552	-2.75578	6.44E-13	7.29E-14	3.17E-15	5.08E-13	5.95E-17	1413	1013	1028	1425	603
RARB	-1.42495	-1.36822	-1.35865	-1.57525	-1.59251	6.48E-13	1.86E-14	3.50E-11	5.77E-16	4.18E-14	1414	883	2067	814	1138
SORBS3	-1.77662	-1.70134	-1.91694	-1.76207	-1.57424	6.48E-13	4.92E-11	1.12E-15	3.43E-11	4.35E-12	1415	1751	946	1924	1656
H2AC19	2.277566	1.808531	2.009001	2.195577	2.116537	6.58E-13	2.27E-11	1.02E-12	2.87E-14	1.24E-13	1416	1631	1596	1124	1245
RECQL	-1.1115	-0.94795	-1.00115	-0.89534	-0.99268	6.73E-13	1.46E-13	5.93E-13	2.76E-12	9.01E-10	1417	1066	1535	1617	2415
TMEM132	-1.84062	-1.68154	-1.89331	-1.86778	-2.03783	6.74E-13	2.06E-11	1.65E-14	3.28E-15	6.35E-12	1418	1616	1148	934	1700
SLC35C2	1.194953	1.176305	0.96909	1.203538	0.91029	6.77E-13	2.50E-12	1.78E-11	5.50E-12	6.60E-10	1419	1338	1946	1684	2356
CXCR4	-1.27019	-1.21228	-1.5418	-1.41217	-1.14832	6.77E-13	1.98E-10	2.99E-15	1.69E-09	1.06E-08	1420	1923	1021	2494	2829
DCBLD1	-1.9041	-1.9289	-1.61197	-1.87698	-1.91961	6.80E-13	2.62E-14	7.84E-14	3.21E-17	3.44E-14	1421	918	1295	635	1116
NRG1	-2.79267	-2.51801	-3.07478	-2.88704	-3.42309	6.87E-13	2.87E-09	2.30E-12	3.05E-10	1.37E-12	1422	2338	1680	2208	1521
ITGAM	-1.02281	-0.75099	-1.15292	-1.02732	-1.27648	6.88E-13	6.75E-07	4.54E-07	4.40E-07	1.87E-09	1423	3380	3847	3469	2528
SNRPB2	1.110866	1.31419	1.141398	1.203571	0.874166	6.93E-13	2.17E-10	6.69E-12	3.03E-09	3.80E-07	1424	1943	1818	2588	3560
PKNOX2	-1.0846	-0.89968	-1.36501	-1.07168	-0.73506	6.95E-13	5.84E-11	4.21E-13	1.35E-08	0.000583	1425	1766	1497	2816	5451
CEP170	-1.72747	-1.83703	-1.66709	-1.74237	-2.2052	6.95E-13	4.73E-17	1.46E-16	1.34E-16	5.07E-19	1426	527	796	719	345
AQP1	-1.80261	-2.13487	-1.69069	-2.08485	-1.06121	7.00E-13	2.28E-15	2.61E-11	4.16E-16	0.000726	1427	745	2021	788	5539
OLA1	1.338847	1.47647	1.428568	1.676128	1.276394	7.22E-13	1.31E-15	4.68E-15	1.68E-18	9.09E-16	1428	714	1061	479	801
PTAFR	-1.49186	-1.29683	-1.54715	-1.22882	-1.56464	7.27E-13	1.35E-12	1.27E-12	9.57E-12	4.22E-12	1429	1279	1620	1752	1653
CYCS	2.254228	1.876426	2.323341	1.810591	2.253661	7.29E-13	8.61E-10	7.03E-14	1.58E-09	4.25E-15	1430	2151	1280	2482	907
RBMS2	-1.18826	-1.25579	-0.96713	-1.36289	-1.16588	7.34E-13	8.24E-11	1.22E-08	5.26E-11	1.02E-11	1431	1802	3064	1983	1767
ZNF331	-1.10737	-1.01959	-1.20767	-1.3222	-1.40204	7.38E-13	1.30E-09	1.16E-11	1.64E-15	1.18E-11	1432	2216	1891	880	1789
FUT11	-1.4002	-1.32824	-1.20715	-1.32537	-1.38378	7.38E-13	1.02E-13	9.24E-11	1.56E-11	9.06E-12	1433	1037	2202	1833	1749
RPS29	1.625615	1.824066	1.503013	1.816208	1.945632	7.44E-13	6.67E-14	2.14E-11	1.60E-15	4.02E-15	1434	1002	1991	879	899
GID8	1.204089	1.561665	1.587513	1.62907	1.268197	7.63E-13	3.70E-15	7.43E-11	1.69E-15	4.14E-10	1435	768	2160	888	2275
TSC22D3	-1.88514	-1.88168	-1.73385	-2.11064	-2.48281	7.65E-13	1.62E-12	4.67E-13	1.97E-15	2.37E-18	1436	1302	1504	899	415
KHSRP	1.295847	1.394194	1.241193	1.492602	1.390252	7.65E-13	4.14E-13	6.99E-14	1.31E-13	4.20E-14	1437	1178	1279	1265	1139
PGRMC1	1.365808	1.22368	1.450888	1.420254	1.034096	7.66E-13	1.10E-11	1.79E-11	7.46E-10	3.15E-07	1438	1545	1949	2351	3516
SLC49A3	-1.27363	-1.42178	-1.76339	-1.58509	-1.63063	7.69E-13	1.94E-13	5.09E-18	9.69E-15	1.36E-16	1439	1099	590	1028	665
NCBP1	0.882432	0.893245	0.956783	0.917683	0.722233	7.74E-13	5.60E-11	7.25E-13	2.22E-11	9.49E-07	1440	1760	1558	1882	3722
IFT172	-0.87063	-0.73076	-0.77063	-0.67534	-0.84717	7.81E-13	6.55E-11	1.21E-12	3.03E-10	7.72E-10	1441	1775	1618	2207	2380
PLEKHG1	-1.19708	-0.81105	-0.87779	-0.82939	-0.72508	7.81E-13	1.11E-06	9.55E-09	1.66E-07	1.06E-06	1442	3508	3003	3263	3753
MPC2	1.553204	1.758272	1.46144	1.784395	1.643164	7.83E-13	1.93E-12	1.06E-13	1.09E-13	4.62E-17	1443	1316	1323	1248	585
ROBO1	-2.40314	-2.05844	-1.68968	-1.89033	-2.21524	8.02E-13	2.34E-11	2.78E-11	5.89E-10	2.50E-14	1444	1636	2032	2305	1076
SPIRE2	1.862543	1.922428	1.78878	1.902144	1.804209	8.09E-13	3.15E-13	1.57E-14	2.94E-14	4.47E-13	1445	1141	1145	1129	1383
CSF3R	-1.83375	-1.76552	-2.05871	-2.02003	-2.01077	8.24E-13	1.07E-10	7.01E-11	3.66E-13	4.40E-10	1446	1839	2153	1387	2287
PTCD3	1.045205	1.142898	1.19943	1.266557	1.065905	8.25E-13	2.75E-16	9.63E-13	1.34E-13	1.39E-12	1447	619	1590	1269	1526
PNPLA6	-1.01386	-0.46549	-0.87217	-0.60733	-0.67543	8.25E-13	0.000395	5.12E-09	1.57E-05	1.22E-05	1448	5167	2879	4341	4338
BMERB1	-1.23869	-0.76924	-1.43921	-1.09381	-1.28264	8.27E-13	3.18E-10	2.47E-15	3.44E-09	2.32E-10	1449	2001	1006	2608	2188
CERS6	1.274295	1.614154	1.799915	1.678249	1.656856	8.35E-13	6.37E-13	1.52E-15	6.60E-14	1.77E-14	1450	1213	968	1203	1043
SYNPO	-2.04342	-2.13387	-2.82204	-2.17385	-2.31847	8.39E-13	2.82E-11	7.63E-15	3.39E-10	1.43E-13	1451	1664	1100	2223	1263
DEK	1.709888	1.381755	1.70964	1.610491	1.891083	8.44E-13	7.35E-10	3.55E-12	4.66E-11	6.73E-17	1452	2124	1743	1964	611
PSMA7	1.987775	1.610945	1.78178	1.920279	1.810085	8.69E-13	1.66E-11	2.78E-13	2.73E-13	7.10E-14	1453	1589	1454	1351	1191
RAPGEF1	-1.55378	-1.80697	-1.5497	-1.74702	-2.08676	8.71E-13	7.24E-16	5.95E-16	5.80E-14	3.18E-19	1454	677	886	1190	329
ADGRE2	-2.20678	-1.62882	-2.09828	-1.8106	-2.42898	8.81E-13	1.43E-07	4.29E-11	1.17E-08	1.77E-13	1455	3075	2088	2791	1282
DHX9	1.051693	0.894351	1.070855	1.004994	0.832134	8.82E-13	7.91E-10	9.84E-12	2.67E-12	6.13E-11	1456	2139	1873	1612	1973
MMP11	-3.79092	-3.78747	-3.37344	-3.72141	-4.12261	8.86E-13	4.28E-14	2.08E-16	7.91E-16	1.44E-15	1457	957	820	831	834
ZBTB7B	1.073138	1.161225	0.978465	1.030716	1.159884	8.86E-13	9.68E-14	8.14E-11	1.79E-12	2.85E-14	1458	1033	2174	1576	1095
GATA2	-1.38729	-0.75646	-1.17914	-1.05319	-0.66931	8.89E-13	7.44E-06	1.55E-09	5.81E-09	0.001192	1459	3951	2645	2674	5695

CNBP	1.044894	1.065058	1.109386	1.040687	1.039444	8.89E-13	8.73E-11	8.12E-12	8.36E-11	1.34E-11	1460	1811	1842	2044	1804
RASSF4	-1.93155	-2.5892	-2.14369	-2.47255	-1.8029	8.89E-13	1.24E-17	8.48E-18	9.66E-17	5.59E-10	1461	445	617	696	2321
MORF4L2	1.251667	0.894462	1.216812	1.175751	0.793575	8.95E-13	1.17E-08	2.85E-11	7.10E-11	8.69E-07	1462	2579	2036	2026	3703
OSBPL2	1.276016	1.40384	1.413452	1.590142	1.333689	9.02E-13	1.51E-14	4.99E-14	2.16E-14	1.72E-12	1463	865	1247	1102	1539
GDF15	2.078342	2.062148	1.618416	2.374792	1.258085	9.03E-13	1.68E-10	9.66E-09	2.16E-15	1.90E-06	1464	1901	3005	907	3871
UBE2C	2.836941	2.710448	2.845741	2.795124	2.681067	9.06E-13	1.08E-11	2.68E-13	1.52E-12	1.04E-12	1465	1544	1450	1557	1489
TNFAIP3	-1.82682	-1.79739	-2.20201	-1.56901	-2.21947	9.39E-13	8.64E-10	2.19E-16	2.49E-11	7.62E-13	1466	2152	823	1894	1451
GGH	2.351221	2.581275	2.358774	2.850335	2.259975	9.51E-13	3.72E-15	6.91E-13	5.99E-19	3.44E-13	1467	770	1552	436	1351
ACVR1	-1.09901	-1.01025	-1.09747	-1.02119	-1.18188	9.57E-13	1.72E-11	2.31E-12	2.39E-13	3.32E-12	1468	1596	1681	1335	1626
LTBP4	-2.09104	-1.88792	-1.79573	-1.96155	-1.93435	9.57E-13	7.90E-14	2.78E-11	3.10E-15	1.22E-13	1469	1018	2031	929	1243
LOX	-2.47161	-2.13738	-1.82501	-2.2046	-2.18841	9.57E-13	6.02E-10	2.00E-11	3.65E-10	1.05E-12	1470	2098	1976	2232	1490
OR10G4	-0.89441	-0.75745	-1.15361	-0.91239	-0.81293	9.80E-13	8.54E-11	1.98E-14	7.37E-10	1.23E-09	1471	1808	1166	2349	2458
MGAT1	-1.46553	-1.80355	-1.41837	-1.69561	-1.83772	9.82E-13	5.92E-14	3.81E-13	1.27E-12	1.55E-15	1472	995	1486	1532	838
UBA52	1.558781	1.345152	1.497549	1.577868	1.333461	9.83E-13	4.29E-11	5.79E-11	1.23E-13	3.04E-10	1473	1724	2129	1259	2230
HEXA	-1.11228	-1.30024	-1.26755	-1.38572	-1.11723	9.89E-13	3.40E-10	5.79E-11	1.81E-11	4.87E-10	1474	2016	2127	1850	2305
FBH1	-1.51166	-1.04612	-1.19135	-1.19177	-1.35147	9.92E-13	3.61E-09	6.97E-12	1.96E-10	3.82E-12	1475	2383	1829	2143	1640
NDUFB11	1.1221	1.170749	0.842358	1.278991	0.885627	1.01E-12	5.54E-15	1.88E-14	1.05E-15	5.50E-13	1476	791	1163	847	1405
ZSWIM6	-1.14854	-1.01616	-1.10023	-1.09618	-0.84145	1.01E-12	1.19E-07	6.18E-11	2.75E-10	4.06E-05	1477	3039	2140	2191	4660
PALLD	-2.94007	-3.08598	-2.37229	-3.11792	-2.6376	1.01E-12	6.24E-16	2.37E-14	2.32E-16	3.40E-12	1478	669	1177	756	1629
RHPN2	1.14212	1.315925	1.118736	1.31951	1.281464	1.02E-12	1.22E-12	3.57E-11	6.17E-10	1.32E-10	1479	1271	2069	2316	2091
SMARCD3	-1.47012	-1.15717	-1.25297	-1.44296	-1.20082	1.04E-12	3.17E-13	3.76E-13	3.35E-15	1.60E-10	1480	1144	1485	937	2117
GSTM2	-1.47552	-0.83144	-1.43703	-1.18579	-1.51787	1.04E-12	0.00021	2.48E-08	3.44E-06	1.28E-10	1481	4921	3209	3926	2085
SMURF2	-1.15794	-0.94871	-0.82794	-0.88429	-0.90599	1.04E-12	2.00E-08	1.63E-07	9.11E-09	3.84E-08	1482	2663	3619	2743	3062
BLCAP	1.788773	1.553989	1.7104	1.838127	1.661505	1.04E-12	3.67E-11	4.68E-13	1.29E-12	3.39E-14	1483	1697	1505	1535	1112
CRABP2	-0.79197	-0.61276	-0.84637	-1.00487	-0.84099	1.05E-12	4.28E-06	8.52E-08	2.19E-11	7.46E-08	1484	3830	3475	1878	3217
IMPDH2	1.761129	1.649987	1.906488	1.764512	1.661017	1.05E-12	3.91E-12	1.50E-13	3.25E-13	2.48E-14	1485	1403	1376	1372	1074
DAB2	-1.70077	-1.395	-2.35377	-1.64851	-2.31668	1.06E-12	1.86E-10	4.61E-16	1.30E-10	6.10E-15	1486	1912	872	2095	938
NME2	2.286714	2.018601	2.194672	2.204086	2.001079	1.07E-12	5.59E-14	2.10E-11	5.35E-17	1.68E-14	1487	987	1988	668	1034
AVL9	1.35292	1.218063	1.550155	1.276997	1.240407	1.08E-12	3.74E-12	4.98E-15	1.46E-11	1.81E-12	1488	1396	1066	1819	1542
CAMK1	-1.32406	-1.26249	-1.59154	-1.22939	-1.24774	1.08E-12	4.77E-13	4.82E-16	2.26E-14	4.93E-07	1489	1193	875	1108	3601
RAE1	1.541825	1.499049	1.302859	1.446298	1.033632	1.08E-12	1.07E-12	1.52E-10	5.82E-14	3.54E-06	1490	1257	2283	1191	4026
RASSF7	1.4965	1.159562	1.442392	1.25353	1.344356	1.10E-12	6.09E-08	1.31E-12	6.63E-10	1.07E-11	1491	2885	1622	2330	1774
OPCML	-0.89215	-0.69756	-0.90066	-0.91712	-0.86773	1.11E-12	1.91E-08	8.72E-12	5.31E-12	3.02E-11	1492	2651	1852	1681	1893
ARHGEF12	-0.90627	-0.91122	-0.77997	-0.71689	-0.74714	1.11E-12	4.86E-13	8.66E-11	2.05E-09	7.26E-08	1493	1197	2190	2520	3214
COPE	0.91672	0.882885	0.71858	0.973192	0.467202	1.12E-12	2.96E-10	2.61E-08	7.42E-14	0.000396	1494	1984	3223	1211	5322
RECK	-1.56175	-1.08861	-1.4172	-1.33353	-1.83391	1.12E-12	7.77E-07	5.65E-12	4.44E-08	7.79E-14	1495	3413	1799	3023	1196
RPN2	2.018286	1.701504	1.496381	1.986535	1.498139	1.12E-12	2.47E-11	1.16E-09	2.18E-13	5.49E-11	1496	1648	2609	1325	1957
ZFP90	-0.90708	-0.87041	-0.99411	-1.08397	-0.88929	1.12E-12	3.00E-10	4.08E-12	1.47E-12	8.55E-11	1497	1991	1755	1554	2026
TMED4	1.154903	1.105678	1.01978	0.985772	1.144185	1.13E-12	5.44E-13	1.04E-11	7.05E-10	6.26E-12	1498	1201	1880	2341	1696
FARSA	1.189932	1.225803	1.130573	1.390434	0.885369	1.14E-12	2.95E-12	1.46E-11	2.57E-18	1.20E-05	1499	1365	1920	504	4334
H4C5	2.178643	1.787823	2.257275	1.985364	2.091404	1.14E-12	3.52E-11	4.51E-14	3.24E-13	5.24E-14	1501	1688	1232	1369	1160
SLC25A1	1.397595	1.217055	1.499551	1.277781	1.349143	1.16E-12	7.33E-13	9.17E-17	2.85E-12	2.53E-17	1502	1224	760	1623	549
ICAM2	-1.72912	-1.71611	-1.88083	-2.12066	-1.92886	1.16E-12	3.33E-13	2.99E-15	1.46E-16	6.00E-14	1503	1152	1018	725	1175
RPS10	2.120334	2.110239	1.753218	2.162644	2.050459	1.18E-12	2.53E-13	6.12E-11	2.03E-16	8.83E-15	1504	1119	2138	742	974
MLEC	1.605333	1.433432	1.488613	1.650352	1.603268	1.18E-12	1.48E-11	9.18E-10	4.45E-18	1.36E-13	1505	1573	2566	527	1253
STAT5B	-0.91042	-0.83182	-1.18005	-0.95939	-0.85122	1.18E-12	2.00E-07	1.40E-11	6.82E-09	5.00E-06	1506	3149	1913	2702	4106
SCRIB	1.076241	1.442055	1.329295	1.485888	1.48638	1.19E-12	4.65E-16	1.18E-13	6.11E-16	3.15E-18	1507	645	1335	820	424
PLBD1	1.43792	1.266847	1.748826	1.34961	1.456708	1.21E-12	2.28E-10	2.87E-16	1.87E-12	2.55E-11	1508	1947	848	1581	1879
TRIM15	2.225062	2.444161	2.534055	2.415441	2.002522	1.21E-12	2.37E-11	4.99E-14	2.98E-14	1.57E-10	1509	1638	1248	1132	2114
ST20	-0.84604	-0.4954	-0.84158	-0.56774	-0.42932	1.22E-12	3.07E-06	2.86E-07	7.27E-05	0.006299	1510	3766	3736	4782	6328
CD164	1.564931	1.297088	1.828604	1.46665	1.53387	1.22E-12	4.69E-11	9.04E-17	8.71E-12	3.20E-17	1511	1741	758	1738	562
EIF3A	0.955316	0.995605	0.882123	0.932224	1.0634	1.22E-12	2.01E-13	2.57E-12	1.32E-09	2.41E-10	1512	1101	1697	2439	2192
LAPTM4B	1.60192	1.496675	1.577277	1.63197	1.614362	1.23E-12	1.08E-11	8.20E-10	6.85E-13	6.37E-13	1513	1543	2550	1460	1434
CCR1	-1.5003	-1.22633	-1.75218	-1.21307	-1.73949	1.23E-12	3.66E-10	1.82E-13	1.66E-08	2.61E-13	1514	2027	1406	2855	1322
PDXDC1	1.366964	0.971124	1.397979	1.369423	1.68544	1.26E-12	1.39E-09	6.13E-14	4.40E-10	9.45E-14	1515	2227	1265	2262	1217
TBRG4	1.451822	1.205117	1.432758	1.205505	1.390618	1.26E-12	1.68E-11	3.71E-13	3.10E-11	6.46E-15	1516	1593	1482	1911	943
TOMM7	1.211315	1.104226	1.037429	1.124128	1.090078	1.27E-12	9.68E-11	1.06E-11	4.72E-10	2.67E-10	1517	1829	1881	2273	2211
TRIM8	-1.38291	-1.2317	-0.87415	-1.15075	-1.12705	1.27E-12	1.12E-09	9.39E-08	1.95E-07	9.27E-07	1518	2191	3498	3297	3718
RASA3	-1.84962	-1.64718	-2.21485	-1.73799	-1.7521	1.28E-12	1.57E-12	4.97E-15	5.88E-14	1.80E-11	1519	1298	1065	1192	1844
DDX27	1.561013	1.51912	1.401115	1.747344	1.429514	1.30E-12	3.35E-12	4.02E-11	4.16E-13	1.54E-09	1520	1380	2080	1400	2494
SH3RF2	2.029497	2.232783	2.156498	2.0315	1.57724	1.30E-12	2.22E-14	2.19E-17	2.76E-12	4.96E-10	1521	901	673	1618	2310
PTPN3	1.352896	1.603526	1.480623	1.559756	1.166956	1.30E-12	5.98E-15	2.34E-13	6.31E-14	8.97E-09	1522	799	1437	1198	2789
GSTK1	1.428363	1.122617	1.219991	1.305475	1.535434	1.31E-12	7.42E-08	9.58E-10	1.04E-08	2.58E-12	1523	2930	2571	2770	1594
GBP2	-1.84696	-1.93228	-1.90775	-1.70227	-1.59346	1.33E-12	1.90E-14	2.58E-13	2.65E-12	6.27E-11	1524	885	1449	1610	1983
TCP1	1.451137	1.498546	1.52734	1.586333	1.301572	1.33E-12	6.54E-14	1.86E-13	1.68E-15	1.74E-11	1525	1000	1408	887	1836
SMG1	0.916348	0.977572	0.93985	1.137395	0.971679	1.33E-12	4.32E-12	1.86E-11	2.31E-14	3.62E-13	1526	1414	1962	1109	1360
TSTD1	1.380264	1.589762	1.595077	1.641062	1.914457	1.34E-12	7.18E-14	2.06E-15	5.49E-13	2.36E-14	1527	1011	994	1434	1071
SLC66A3	-0.85259	-0.9104	-1.09923	-0.90036	-1.04884	1.34E-12	1.93E-11	5.29E-17	3.50E-11	1.45E-13	1528	1611	719	1929	1264
TKT	1.805029	1.97601	1.867925	2.184744	2.156488	1.34E-12	9.51E-15	6.47E-15	1.19E-17	7.70E-18	1529	830	1085	585	481
NECTIN1	1.331389	1.418446	1.452168	1.699512	1.104031	1.35E-12	1.51E-11	5.50E-14	3.50E-14	2.10E-10	1530	1578	1256	1149	2166
ZC2HC1A	-1.16842	-0.73102	-0.72362	-0.90964	-0.65696	1.35E-12	3.14E-07	6.95E-08	2.35E-10	4.05E-06	1531	3227	3443	2172	4050
PDZD2	-1.69686	-1.36894	-1.64275	-1.50418	-1.24253	1.35E-12	6.84E-11	3.87E-14	1.39E-12	4.70E-07	1532	1783	1225	1544	3594
ZNF593	0.990296	1.037886	0.96411	1.044944	0.799347	1.36E-12	7.49E-12	1.01E-14	2.90E-13	1.99E-08	1533	1498	1117	1357	2941

MRPL37	1.122215	0.890074	1.066961	0.877078	0.723273	1.37E-12	2.46E-08	3.32E-14	1.06E-07	1.56E-06	1534	2699	1203	3176	3830
RPL41	1.912681	1.548251	1.473631	1.671906	1.860438	1.39E-12	2.36E-10	3.83E-10	2.17E-11	1.56E-13	1535	1952	2424	1874	1268
RNF114	1.12226	1.119309	0.933882	1.198695	0.968668	1.41E-12	2.06E-11	1.99E-11	1.23E-12	5.01E-14	1536	1618	1974	1527	1153
CAMK4	-1.03791	-0.70607	-0.81001	-0.85384	-0.82175	1.41E-12	7.26E-08	3.71E-10	2.74E-09	1.75E-08	1537	2921	2417	2567	2917
PUS1	1.208843	1.291471	1.141923	1.252668	1.160498	1.41E-12	2.17E-11	1.19E-11	1.24E-11	7.52E-13	1538	1626	1893	1798	1449
MARCKSL1	1.483962	1.14478	1.70959	1.535464	2.060261	1.41E-12	1.71E-10	5.47E-14	2.30E-11	1.08E-10	1539	1903	1255	1884	2064
RPL30	2.228191	2.22605	2.143324	2.290743	2.252965	1.41E-12	2.36E-14	1.05E-10	4.30E-18	3.39E-15	1540	909	2225	525	883
AKR1B1	-2.86921	-2.79208	-3.27189	-2.97575	-3.57456	1.45E-12	4.30E-12	1.35E-16	6.73E-13	9.74E-16	1541	1412	790	1458	804
YTHDF1	1.076195	1.298057	1.008397	1.183279	0.599326	1.45E-12	4.48E-13	1.04E-11	6.81E-10	0.000145	1542	1186	1878	2333	5038
ANXA1	-2.75737	-2.83803	-2.41717	-2.91617	-2.90258	1.58E-12	3.57E-12	3.94E-11	6.30E-13	1.16E-14	1543	1388	2077	1449	1004
UBE2V1	1.251841	1.179703	1.043158	1.281573	0.943705	1.61E-12	4.49E-12	9.56E-12	3.75E-13	1.65E-11	1544	1421	1868	1390	1830
PILRA	-1.46958	-1.33567	-1.35042	-1.24369	-1.59991	1.62E-12	5.59E-07	8.80E-09	6.72E-07	3.19E-10	1545	3334	2989	3568	2234
AP1S2	-1.07525	-0.89018	-1.12047	-0.93517	-1.26405	1.63E-12	3.21E-10	6.77E-17	5.63E-10	3.12E-14	1546	2003	736	2294	1105
SERPINB9	-1.03319	-0.97607	-0.97447	-1.07113	-0.83771	1.63E-12	6.65E-12	1.88E-14	8.28E-14	1.27E-05	1547	1476	1164	1226	4356
SLC16A1	1.572175	1.487636	1.571477	1.819614	1.322638	1.64E-12	2.78E-12	4.78E-11	1.49E-14	1.87E-10	1548	1359	2102	1067	2145
STARD7	1.155254	1.05242	1.132974	1.071118	1.086487	1.66E-12	9.42E-11	1.28E-13	6.77E-11	2.89E-13	1549	1823	1351	2017	1330
CD99	-1.53672	-1.39505	-1.27007	-1.31628	-1.11337	1.66E-12	3.97E-09	1.05E-09	1.37E-10	7.06E-07	1550	2397	2586	2100	3666
NAIP	-1.54675	-1.41218	-1.70764	-1.36254	-1.20866	1.69E-12	3.29E-08	4.82E-12	4.26E-08	1.04E-07	1551	2753	1777	3013	3288
NCR1	-0.90428	-0.90784	-0.74161	-0.90109	-0.64992	1.71E-12	2.63E-12	1.20E-09	2.60E-12	9.06E-07	1552	1351	2618	1606	3712
NR2F6	1.478237	1.365465	1.821479	1.312539	1.482987	1.74E-12	3.10E-10	6.79E-16	2.03E-10	4.96E-13	1553	1998	898	2154	1392
CCDC80	-2.04198	-1.41921	-1.5267	-1.72589	-1.69155	1.74E-12	6.68E-07	2.29E-07	8.34E-08	5.14E-11	1554	3376	3687	3136	1952
ADRM1	1.709501	1.594875	1.676462	1.723172	1.708445	1.75E-12	7.46E-13	2.22E-12	2.07E-13	2.03E-10	1555	1228	1678	1319	2158
EBPL	1.778986	2.095053	1.750537	2.259176	2.048036	1.76E-12	5.46E-16	2.96E-11	8.25E-17	1.28E-14	1556	664	2048	688	1013
GSS	1.482586	1.3761	1.186004	1.520194	1.264638	1.78E-12	3.79E-11	2.28E-11	2.60E-11	2.74E-10	1557	1703	1997	1899	2214
PTPN14	-1.08756	-0.88471	-1.1444	-0.94301	-1.29177	1.78E-12	1.06E-09	1.50E-10	2.49E-08	3.81E-13	1558	2183	2280	2922	1366
CLEC10A	-1.14272	-0.87029	-1.47481	-1.13357	-1.4879	1.81E-12	7.43E-11	8.27E-12	2.21E-09	2.62E-10	1559	1787	1845	2533	2209
NUDT4B	1.429033	1.584877	1.367558	1.853102	1.223309	1.83E-12	6.19E-15	7.55E-11	8.03E-17	8.70E-12	1560	805	2163	687	1744
FGF2	-2.10286	-2.17653	-2.23498	-2.41164	-2.32681	1.84E-12	2.50E-12	3.97E-13	7.73E-13	1.30E-12	1561	1339	1488	1468	1514
DUX1	-1.01498	-0.68982	-1.12915	-0.82641	-0.97829	1.86E-12	6.06E-08	3.97E-10	8.04E-07	1.72E-11	1562	2881	2431	3592	1833
TWF1	0.99803	0.734696	1.093401	0.899507	1.251953	1.88E-12	5.43E-08	4.84E-12	1.07E-09	3.75E-10	1563	2862	1779	2407	2251
TPRKB	0.902594	0.93767	0.698104	1.012077	0.80941	1.91E-12	2.67E-14	4.24E-10	5.45E-14	3.43E-11	1564	921	2439	1183	1911
LILRA6	-0.92551	-0.62306	-1.00395	-0.79069	-0.86174	1.93E-12	3.98E-08	1.66E-10	1.14E-08	2.95E-08	1565	2800	2296	2788	3021
SLC9A3R2	-2.26502	-2.30247	-2.67917	-2.21684	-2.36631	1.93E-12	4.91E-12	1.65E-14	1.18E-11	3.42E-12	1566	1435	1147	1789	1631
TFG	1.067784	0.859152	1.255234	1.052688	1.05813	1.94E-12	6.47E-09	1.54E-11	1.31E-09	4.56E-10	1567	2485	1928	2438	2293
IGFBP4	-2.85089	-2.55189	-2.48838	-2.58552	-2.76995	1.94E-12	1.24E-13	5.44E-15	2.30E-16	1.89E-15	1568	1052	1076	753	847
ALDOA	1.901448	1.369588	2.188091	1.730773	1.766576	1.95E-12	2.26E-10	1.92E-14	2.26E-12	3.42E-13	1569	1945	1165	1593	1350
TGFBR1	-1.46432	-1.41259	-0.81575	-1.21549	-1.15535	1.97E-12	3.40E-13	2.48E-05	1.33E-09	3.03E-07	1570	1154	4842	2443	3508
LRP5	1.067717	1.271727	1.665138	1.478135	1.642804	1.98E-12	2.67E-14	1.76E-17	2.16E-15	8.77E-13	1571	920	660	906	1466
C11orf65	-0.95511	-0.90949	-0.96539	-1.15076	-0.81321	1.99E-12	1.27E-09	6.69E-12	1.01E-12	2.05E-06	1572	2209	1819	1504	3880
DDAH1	1.359725	1.386574	1.726552	1.511865	1.652256	1.99E-12	9.63E-12	6.92E-18	2.64E-13	2.66E-17	1573	1524	607	1346	552
CRMP1	-1.40581	-1.13992	-1.20764	-1.52848	-1.70739	2.00E-12	1.75E-10	3.88E-10	1.61E-13	6.58E-13	1574	1906	2426	1289	1436
CERS2	1.226103	0.984077	1.134229	1.006984	1.075366	2.06E-12	2.04E-08	1.49E-11	2.10E-08	2.08E-09	1575	2669	1921	2898	2545
PPP1R16A	1.285989	1.553176	1.58815	1.532038	1.409837	2.08E-12	9.48E-17	5.57E-18	3.80E-15	4.13E-17	1576	568	594	949	578
METTL5	1.060079	1.070059	1.291325	1.092462	1.002373	2.08E-12	4.50E-11	1.32E-15	2.91E-12	9.02E-11	1577	1733	957	1625	2034
CNPPD1	0.957166	1.019455	1.003521	1.006302	1.010281	2.10E-12	1.17E-12	3.10E-14	2.90E-14	7.83E-16	1578	1265	1197	1128	788
ROCK2	1.208997	1.163958	1.215619	1.295224	1.347142	2.14E-12	1.76E-10	4.11E-12	8.32E-11	1.73E-15	1579	1907	1756	2043	843
IRF2BPL	-0.99882	-0.61727	-0.82988	-0.87454	-1.08378	2.14E-12	4.88E-05	1.83E-07	1.05E-07	3.53E-10	1580	4478	3643	3175	2243
STAT6	1.307528	1.163143	1.252909	1.252248	1.2271	2.16E-12	7.33E-09	3.01E-13	4.07E-09	1.13E-13	1581	2499	1462	2634	1233
ITGAV	-2.0366	-2.31869	-2.12403	-2.0751	-2.34204	2.16E-12	5.01E-14	3.76E-14	2.21E-14	1.92E-13	1582	970	1218	1106	1289
SERPINE1	-3.15219	-3.13778	-3.1275	-3.42114	-3.49715	2.19E-12	6.55E-09	5.05E-11	6.37E-11	6.46E-11	1583	2487	2109	2006	1988
DCAF13	0.891685	1.060858	1.398932	1.1439	0.678325	2.24E-12	1.25E-12	1.42E-13	1.68E-09	0.000414	1584	1274	1371	2491	5344
DNM2	0.90737	1.072809	0.972676	1.042506	0.967709	2.25E-12	1.92E-11	9.29E-13	3.34E-13	2.55E-11	1585	1608	1579	1376	1878
TPX2	1.968007	1.992292	2.108513	2.112034	1.662142	2.28E-12	1.14E-10	4.95E-13	1.65E-11	2.86E-07	1586	1850	1511	1836	3490
ARHGEF10	-1.19329	-0.73752	-1.18766	-0.9287	-1.18911	2.29E-12	3.64E-06	5.90E-13	2.99E-07	9.31E-10	1587	3797	1534	3371	2419
ADCY9	-1.36225	-1.18745	-1.12377	-1.14754	-1.15341	2.29E-12	1.22E-10	5.99E-11	1.03E-09	3.35E-07	1588	1854	2134	2402	3526
TM9SF2	1.807158	1.307699	1.907685	1.591076	2.088347	2.30E-12	1.59E-09	1.98E-13	2.03E-13	3.53E-16	1589	2239	1415	1316	731
CAMK2N1	1.38574	1.239929	1.384183	1.406929	0.887348	2.30E-12	1.96E-10	2.46E-13	1.35E-12	1.41E-05	1590	1918	1442	1539	4381
TNRC6B	-1.2597	-1.01101	-0.81576	-1.10052	-1.00556	2.30E-12	2.40E-10	9.43E-11	1.22E-11	1.70E-10	1591	1954	2206	1797	2127
TMEM97	1.869103	1.858938	1.632683	2.061954	1.518725	2.30E-12	4.31E-12	8.68E-12	2.03E-16	1.81E-12	1592	1413	1850	741	1543
CHMP4B	1.225272	1.464679	1.51852	1.513155	1.59966	2.30E-12	3.83E-14	1.04E-16	4.37E-16	7.65E-15	1593	949	772	791	961
H2AC17	2.336179	2.058891	2.4653	2.147319	2.594236	2.34E-12	3.67E-12	1.33E-17	2.02E-12	8.62E-17	1594	1393	646	1585	629
BPGM	-1.73174	-1.42487	-1.47296	-1.45841	-1.57697	2.37E-12	6.86E-10	1.78E-15	4.62E-11	3.08E-11	1595	2109	987	1962	1895
PPP3CC	-1.48336	-1.26368	-1.95751	-1.51959	-1.74549	2.38E-12	3.25E-09	5.34E-20	1.51E-11	5.34E-16	1596	2366	375	1828	759
BSG	1.393165	1.2826	1.630763	1.46663	1.406975	2.42E-12	8.92E-13	1.56E-13	3.06E-13	1.53E-12	1598	1246	1386	1362	1534
CCT7	1.724914	1.652152	1.926436	1.733049	1.708411	2.49E-12	2.98E-15	6.18E-14	1.21E-16	5.08E-16	1599	755	1266	711	755
PTP4A1	1.340256	1.024332	1.424726	1.182658	1.01226	2.50E-12	1.04E-07	1.03E-12	1.19E-09	3.13E-09	1600	3007	1598	2421	2613
IRAK1	1.520151	1.348293	1.043521	1.387239	1.10199	2.51E-12	2.72E-10	2.08E-08	3.90E-12	1.97E-08	1601	1969	3171	1651	2940
H4C4	2.153557	1.877209	2.357661	1.988215	2.402246	2.54E-12	4.73E-12	1.44E-15	1.59E-13	1.23E-16	1602	1429	965	1288	660
GHITM	1.320528	1.355936	1.773127	1.553969	1.548531	2.57E-12	3.86E-15	2.15E-16	1.08E-12	3.40E-16	1603	771	821	1515	728
IHH	1.851979	2.087877	1.870901	2.186843	1.965509	2.57E-12	5.11E-12	8.79E-12	2.50E-14	7.16E-15	1604	1442	1854	1114	951
FBN2	-1.35979	-1.38461	-1.44145	-1.40187	-1.63312	2.59E-12	8.19E-10	3.50E-13	7.38E-12	2.01E-11	1605	2145	1473	1714	1859
POM121C	0.970938	1.026203	0.771037	1.047569	0.755536	2.65E-12	3.21E-12	1.99E-11	6.04E-12	1.29E-07	1606	1375	1973	1692	3329
MYCBP	0.98772	0.864674	0.840956	0.858622	0.714977	2.66E-12	1.11E-08	5.22E-10	9.53E-09	1.00E-06	1607	2569	2468	2754	3738

RNF166	-1.41274	-1.30593	-1.84812	-1.40543	-1.25237	2.68E-12	8.35E-12	3.00E-16	1.67E-10	2.58E-07	1608	1511	850	2115	3463
BEX4	-1.79256	-1.84903	-1.79218	-1.63852	-2.04633	2.71E-12	1.37E-14	1.43E-12	9.39E-12	1.62E-17	1609	858	1631	1750	526
SAMD4A	-1.53839	-1.43481	-1.82735	-1.51066	-1.51872	2.79E-12	2.28E-12	3.85E-15	9.91E-16	1.50E-12	1610	1332	1042	845	1533
HCFC1R1	-1.22638	-1.10882	-0.86977	-1.06084	-1.14665	2.81E-12	9.32E-14	9.82E-11	7.52E-14	1.86E-11	1611	1029	2215	1215	1850
CD8A	-1.39869	-0.71703	-1.31819	-1.06126	-1.08468	2.83E-12	0.000882	5.21E-08	2.28E-05	0.000176	1612	5459	3385	4420	5086
IFNAR2	-1.17995	-0.8996	-1.08738	-1.14624	-0.97361	2.83E-12	2.52E-08	2.43E-09	1.33E-09	1.21E-09	1613	2704	2744	2446	2455
PEAK1	-1.90317	-1.78297	-1.5066	-1.74949	-1.79643	2.84E-12	6.99E-16	1.36E-13	6.05E-17	1.74E-13	1614	675	1365	674	1281
HK3	-1.68485	-1.23692	-1.56095	-1.47533	-1.60623	2.87E-12	2.98E-06	3.96E-10	1.10E-07	3.30E-11	1615	3758	2430	3186	1905
ALDH18A1	1.078282	1.16685	1.225658	1.432051	1.296708	2.88E-12	6.86E-14	1.84E-10	2.75E-15	5.99E-13	1616	1007	2310	919	1419
APOBEC3C	-1.39107	-1.42609	-1.96108	-1.62833	-1.49201	2.99E-12	5.76E-10	1.25E-14	1.20E-09	2.04E-10	1617	2094	1132	2422	2159
LIX1L	-2.18198	-2.01726	-2.00507	-2.06254	-2.21919	3.04E-12	1.49E-18	3.62E-18	7.52E-19	1.45E-19	1618	344	563	443	306
OCM	-0.87457	-0.55366	-0.9912	-0.77706	-0.51613	3.05E-12	2.83E-08	2.31E-12	7.17E-10	0.000411	1619	2729	1682	2344	5341
CACYBP	1.411161	1.340566	1.254269	1.246105	1.391179	3.09E-12	1.33E-13	1.08E-12	1.58E-14	3.35E-15	1620	1060	1606	1073	881
NAPRT	1.82899	1.708901	2.249874	1.667585	1.624389	3.12E-12	3.97E-13	2.92E-18	4.52E-09	1.80E-11	1621	1175	548	2646	1845
HIF1A	-2.05159	-2.24672	-1.44492	-2.10003	-1.79769	3.16E-12	8.40E-15	7.05E-09	8.21E-13	2.15E-10	1622	826	2943	1476	2176
TNFAIP8	-1.22915	-1.15676	-1.1304	-1.21358	-1.43431	3.19E-12	3.87E-12	2.54E-11	1.51E-11	3.88E-17	1623	1400	2013	1827	574
PHLDA3	-2.07182	-1.63731	-2.11493	-1.68341	-2.11311	3.22E-12	1.03E-06	7.20E-13	2.55E-08	4.74E-14	1624	3485	1557	2925	1147
TRAF7	0.818222	0.590616	0.841009	0.734392	0.669977	3.25E-12	7.96E-08	4.41E-13	7.20E-10	2.59E-10	1625	2942	1501	2345	2204
PCGF6	-0.92436	-0.74409	-0.8936	-0.84474	-0.65211	3.25E-12	7.71E-09	2.13E-11	2.65E-09	2.09E-07	1626	2509	1990	2561	3422
STAT5A	-1.14078	-0.91344	-1.20872	-1.20975	-1.2017	3.26E-12	3.25E-08	8.87E-14	2.89E-11	7.83E-14	1627	2750	1309	1905	1197
PDE4B	-1.71747	-1.25434	-1.65348	-1.68818	-1.92138	3.28E-12	7.03E-08	1.35E-10	2.22E-09	1.93E-12	1628	2910	2262	2535	1549
PSENEN	0.870929	0.837096	0.865373	0.844636	0.914705	3.28E-12	5.18E-09	9.94E-11	3.77E-09	1.37E-12	1629	2444	2218	2625	1520
ZNF83	-1.09631	-0.90264	-1.07648	-0.95906	-0.85193	3.31E-12	1.21E-08	1.22E-13	4.32E-09	1.46E-08	1630	2590	1343	2641	2889
PRDX1	1.474449	1.085987	1.53113	1.437737	1.457599	3.31E-12	4.23E-11	7.41E-13	1.72E-12	1.19E-12	1631	1722	1560	1572	1505
ASL	1.777563	1.415669	1.199893	1.417181	1.571226	3.32E-12	3.57E-10	5.85E-10	5.47E-10	8.03E-12	1632	2023	2490	2292	1729
RNF215	-0.91633	-0.67661	-0.90035	-0.92996	-0.75354	3.34E-12	1.27E-08	2.23E-09	1.96E-10	3.60E-09	1633	2599	2726	2144	2631
IDH3B	1.405791	1.438919	1.091571	1.249466	1.084622	3.36E-12	1.76E-11	7.84E-10	1.01E-09	2.11E-07	1634	1598	2542	2396	3424
H2BC5	2.275709	1.89231	2.44082	1.965185	2.562183	3.39E-12	6.98E-12	3.79E-18	9.94E-12	2.94E-18	1635	1482	571	1758	422
MGST2	1.61615	0.892809	1.489472	1.408555	1.216756	3.39E-12	0.000213	7.86E-07	7.39E-14	1.01E-05	1636	4928	3975	1209	4278
RPL7	2.011486	2.179052	1.981923	2.279261	2.292486	3.44E-12	1.52E-14	3.19E-11	1.31E-17	1.71E-14	1637	868	2054	589	1036
ACE2	2.133223	2.147153	1.596499	2.253237	1.346235	3.50E-12	3.14E-13	2.00E-08	9.60E-13	1.09E-07	1638	1140	3168	1494	3297
UBE2V2	0.913156	1.105747	1.255478	1.069471	0.898639	3.53E-12	1.37E-14	2.22E-16	4.33E-12	7.31E-12	1639	859	825	1664	1718
COX7B	1.731873	1.670908	1.817495	1.869333	1.840901	3.56E-12	1.75E-14	8.20E-15	3.33E-13	4.96E-18	1640	878	1108	1375	455
KRTAP5-1	-0.96557	-0.53945	-0.9738	-0.68049	-0.53959	3.58E-12	8.53E-05	3.42E-09	2.60E-05	0.000242	1641	4649	2814	4463	5178
ATP5PO	1.214639	1.220333	1.398574	1.263215	1.357801	3.59E-12	9.31E-12	9.32E-13	5.92E-13	1.72E-13	1642	1520	1581	1442	1280
PLEKHH3	-0.94211	-0.73927	-0.94613	-0.76737	-1.09762	3.59E-12	1.89E-09	3.59E-14	1.96E-10	6.00E-14	1643	2266	1212	2145	1174
WDFY4	-1.07314	-0.76078	-1.21436	-1.05675	-1.46065	3.63E-12	4.77E-06	1.07E-08	2.00E-07	1.57E-13	1644	3853	3026	3302	1271
AOC3	-1.9529	-1.45223	-1.80904	-1.75832	-2.167	3.67E-12	5.44E-07	1.66E-09	2.49E-08	2.20E-15	1645	3326	2656	2921	854
H4C3	1.805915	1.740787	2.067511	1.774199	2.090373	3.67E-12	2.01E-12	2.68E-16	1.30E-13	6.69E-16	1646	1323	841	1262	771
SMARCC1	1.112599	1.220595	1.258614	1.356892	1.552485	3.71E-12	2.56E-12	2.13E-10	9.83E-14	3.95E-13	1647	1345	2338	1243	1368
S100A4	-2.69781	-2.91633	-3.26598	-2.68794	-3.00074	3.73E-12	7.45E-17	3.08E-15	6.18E-12	5.81E-14	1648	550	1023	1697	1170
FZD1	-1.54294	-1.13392	-1.15941	-1.24579	-1.37413	3.75E-12	2.53E-06	2.97E-07	3.42E-07	2.99E-10	1649	3715	3750	3409	2228
UQCRC1	1.485277	1.233233	1.682506	1.359204	1.692853	3.90E-12	1.27E-13	7.29E-13	6.38E-11	7.04E-16	1650	1055	1559	2008	775
DNTTIP1	1.355847	1.473676	1.45975	1.832687	1.441523	3.97E-12	5.67E-12	1.20E-08	3.83E-14	1.86E-10	1651	1457	3060	1157	2141
STK10	-1.11107	-1.40389	-1.50863	-1.37362	-1.30728	3.98E-12	4.75E-12	2.28E-16	2.15E-11	1.17E-14	1652	1430	829	1870	1005
SLC39A6	-0.80913	-0.78579	-0.91899	-0.96847	-0.81314	3.98E-12	1.17E-12	1.74E-15	1.67E-15	6.22E-14	1653	1264	981	885	1181
DDX21	1.429287	1.474865	1.513838	1.587898	1.416627	4.02E-12	1.81E-13	1.83E-11	3.87E-16	3.97E-13	1654	1093	1957	786	1369
NISCH	-0.97396	-0.74434	-0.86312	-0.78869	-0.84186	4.03E-12	5.32E-08	2.69E-12	6.70E-08	6.92E-09	1655	2855	1706	3086	2737
DNAJC28	-0.95435	-0.83952	-0.79802	-1.03564	-0.65338	4.09E-12	6.29E-10	5.31E-11	1.18E-14	1.18E-08	1656	2101	2116	1050	2851
RASSF8	-1.94389	-1.59539	-1.73633	-1.8941	-1.82928	4.09E-12	2.50E-09	4.78E-12	4.72E-12	8.05E-12	1657	2310	1775	1671	1731
FBXO34	1.033685	1.113006	1.375035	1.035098	1.076812	4.17E-12	3.73E-09	1.49E-16	1.18E-07	5.36E-17	1658	2387	797	3197	597
RPL36	2.006728	2.08554	1.80363	2.374433	2.020877	4.18E-12	2.53E-12	8.19E-11	1.33E-16	4.03E-12	1659	1343	2178	718	1649
MEF2D	-1.2176	-1.24955	-1.34189	-1.33746	-1.56864	4.18E-12	6.81E-14	1.62E-13	5.94E-14	3.78E-17	1660	1006	1390	1194	571
PTK6	0.877369	0.939694	0.857916	0.980804	0.812789	4.18E-12	2.14E-10	2.68E-12	1.46E-11	1.34E-10	1661	1939	1704	1820	2092
SLC38A1	1.654385	1.709287	1.351827	1.81452	1.399602	4.19E-12	1.65E-13	5.39E-11	2.55E-16	2.98E-12	1662	1077	2117	765	1615
IGLL5	-5.02383	-5.13505	-6.46718	-5.80356	-5.38034	4.21E-12	2.76E-13	6.71E-15	8.98E-14	2.43E-12	1663	1124	1088	1234	1583
SIRPA	-1.81628	-1.65225	-1.87954	-1.87778	-2.28526	4.25E-12	5.84E-09	1.51E-16	1.73E-10	4.04E-15	1664	2468	802	2121	900
MAOB	-0.85102	-0.65762	-0.70449	-0.8124	-0.76908	4.33E-12	1.02E-09	9.93E-10	2.17E-11	2.58E-10	1665	2175	2578	1872	2203
ZNF831	-0.88477	-0.57654	-0.6287	-0.76625	-1.08934	4.34E-12	8.59E-07	4.32E-08	3.26E-10	3.94E-09	1666	3435	3344	2216	2649
VAMP2	-1.22872	-1.19706	-1.51495	-1.42363	-1.067	4.40E-12	1.18E-11	1.13E-13	2.61E-10	3.35E-07	1667	1551	1331	2185	3525
LYST	-1.34449	-1.04898	-1.40129	-1.22472	-1.75357	4.42E-12	8.83E-13	4.04E-14	1.62E-13	1.19E-15	1668	1244	1227	1291	817
TBC1D2B	-1.60318	-1.46463	-1.54195	-1.318	-1.38928	4.43E-12	2.45E-10	3.63E-15	7.31E-13	5.80E-10	1669	1956	1040	1465	2333
HLA-DMA	-1.98833	-1.67935	-1.46871	-1.40485	-1.96235	4.43E-12	9.91E-09	5.96E-06	1.46E-05	5.91E-09	1670	2552	4446	4318	2713
ERP29	1.201253	1.166335	0.969401	1.415088	1.131627	4.47E-12	7.97E-13	7.18E-10	1.34E-15	7.15E-10	1671	1231	2527	866	2370
PON2	1.235633	1.229032	1.218649	1.420254	1.335154	4.50E-12	4.71E-10	1.01E-13	8.23E-14	7.93E-11	1672	2056	1316	1222	2018
PTPRCAP	-1.8529	-1.74706	-2.21553	-1.9787	-2.0416	4.54E-12	3.15E-12	3.13E-16	3.81E-12	2.04E-12	1673	1369	851	1646	1559
DTX2	1.036582	1.080004	1.045194	1.105162	0.717126	4.57E-12	4.64E-12	1.15E-10	6.58E-12	1.24E-05	1674	1426	2235	1706	4344
TARS3	-1.01508	-0.7733	-1.13566	-0.9359	-1.15875	4.58E-12	2.42E-08	1.54E-10	5.03E-10	6.99E-11	1675	2696	2284	2283	2005
NUAK1	-1.96842	-1.383	-1.70575	-1.83328	-1.83887	4.66E-12	2.07E-08	2.86E-11	9.54E-11	4.82E-13	1676	2672	2038	2060	1390
ECT2	1.552993	1.573082	1.545355	1.595451	1.636848	4.66E-12	1.56E-12	9.31E-13	3.39E-14	1.09E-14	1677	1295	1580	1145	994
RAB40C	0.998542	1.128661	1.44733	1.408762	0.796946	4.69E-12	5.57E-06	1.02E-08	3.13E-09	1.66E-06	1678	3887	3014	2590	3847
IFI27	2.496895	1.737415	2.765311	2.483445	2.386067	4.71E-12	3.45E-07	6.62E-12	1.50E-14	1.70E-10	1679	3243	1814	1069	2126
RAI14	-2.15202	-1.63861	-1.89998	-1.7672	-2.13878	4.80E-12	2.57E-08	1.12E-09	3.70E-08	1.37E-13	1680	2710	2604	2990	1255

ACSS2	1.488638	1.472999	1.620927	1.595725	1.525259	4.82E-12	4.23E-11	4.75E-14	2.68E-13	5.58E-14	1681	1723	1235	1350	1166
ARHGAP4!	-1.14984	-0.76364	-1.45186	-0.90144	-1.16306	4.86E-12	1.30E-08	1.75E-13	1.12E-06	1.74E-07	1682	2601	1399	3669	3386
CHFR	-1.0828	-0.93334	-1.37115	-1.06636	-1.11368	4.88E-12	3.48E-08	1.09E-14	3.26E-09	1.69E-10	1683	2771	1123	2602	2125
ZNF781	-0.94068	-0.61863	-0.81599	-0.77421	-0.52287	4.91E-12	8.40E-07	8.29E-10	2.27E-09	0.000265	1684	3431	2551	2538	5204
RPL36A	2.485532	2.59253	2.117176	2.777535	2.551406	4.96E-12	4.89E-14	2.26E-11	4.04E-18	1.24E-14	1685	966	1995	518	1011
SIGMAR1	1.24147	1.476857	1.261096	1.554209	1.051411	5.01E-12	8.69E-13	3.91E-11	1.75E-13	2.80E-08	1686	1242	2076	1302	3011
FAM114A2	-0.94816	-0.92797	-0.83624	-0.7194	-0.81518	5.04E-12	3.44E-10	2.45E-09	5.11E-08	2.11E-10	1687	2019	2747	3040	2169
HMGB3	1.282284	1.283947	1.388018	1.393225	1.174946	5.12E-12	4.38E-12	2.16E-14	2.78E-13	6.92E-10	1688	1417	1170	1352	2368
PSME3	1.269572	1.171214	1.171783	1.484385	1.194711	5.17E-12	5.19E-10	1.72E-09	3.27E-16	4.84E-11	1689	2071	2661	776	1948
ZNF664	1.16344	1.138709	1.199371	1.16562	1.279313	5.22E-12	3.83E-11	3.29E-11	7.06E-12	2.68E-15	1690	1709	2058	1711	866
SALL3	-0.88211	-0.48397	-0.67546	-0.74736	-1.144	5.24E-12	0.000275	2.01E-07	3.90E-06	8.08E-10	1691	5029	3658	3961	2392
PRELID2	1.381674	1.222727	1.506354	1.421788	1.349715	5.26E-12	7.10E-12	1.85E-12	9.37E-15	1.07E-12	1692	1486	1658	1026	1494
CYTH3	-1.06904	-0.57763	-0.7873	-0.71298	-0.91912	5.29E-12	2.07E-05	8.27E-09	9.43E-07	1.23E-10	1693	4221	2979	3624	2080
DCXR	1.475173	1.401944	1.345102	1.429826	1.364239	5.35E-12	5.91E-14	1.50E-13	8.92E-14	7.99E-13	1694	994	1375	1233	1455
TIMM50	0.941575	1.286832	1.117751	1.110608	0.866384	5.41E-12	9.34E-17	1.26E-13	1.74E-14	1.38E-08	1695	564	1349	1081	2881
MXD4	-1.20624	-1.18956	-0.98919	-1.06129	-1.32846	5.45E-12	2.82E-12	1.38E-08	1.00E-09	1.35E-10	1696	1362	3087	2393	2096
MTMR11	1.526784	1.401362	1.858678	1.398929	1.547144	5.52E-12	1.74E-12	1.21E-17	8.00E-11	3.58E-14	1697	1308	642	2040	1120
HINT1	1.450757	1.452707	1.679777	1.509171	1.82031	5.57E-12	4.37E-14	1.36E-17	1.04E-12	1.83E-17	1698	960	647	1510	529
PDIA4	1.521994	1.058676	1.209631	1.375022	1.407509	5.57E-12	8.74E-08	1.59E-09	2.70E-10	2.15E-09	1699	2966	2649	2187	2553
DNAH8	-0.92403	-0.80929	-1.04358	-1.00828	-0.83566	5.58E-12	6.13E-10	1.81E-13	3.59E-11	9.13E-11	1700	2100	1405	1936	2037
KDELR1	1.166239	0.885483	1.228894	1.04304	0.915977	5.62E-12	2.37E-08	3.68E-13	4.34E-09	1.65E-07	1701	2693	1478	2642	3372
CBWD2	1.30545	1.168746	1.008116	1.313519	1.16519	5.74E-12	4.08E-11	3.96E-08	4.01E-13	4.87E-10	1702	1717	3329	1397	2303
ZNF362	-1.56207	-1.10029	-1.03492	-1.15372	-1.11253	5.78E-12	1.66E-07	6.06E-06	1.87E-06	3.03E-07	1703	3103	4454	3791	3507
PI4K2A	-0.99512	-0.88441	-0.75443	-0.90799	-1.03877	5.85E-12	3.29E-08	1.92E-09	2.38E-08	1.63E-16	1704	2751	2687	2916	675
PAMR1	-1.483	-1.35196	-1.48245	-1.60739	-1.09751	5.86E-12	1.32E-10	1.71E-10	8.26E-14	2.27E-06	1705	1867	2300	1223	3918
RPS5	2.202691	1.980055	1.91639	2.089008	2.230956	5.86E-12	1.24E-10	2.08E-09	8.69E-13	6.95E-13	1706	1858	2712	1483	1443
IKZF3	-1.24965	-0.83523	-1.42242	-1.24004	-1.15891	5.87E-12	8.76E-06	5.49E-11	9.19E-11	6.80E-08	1707	4000	2122	2057	3187
CARD16	-1.54588	-1.24156	-0.85968	-1.02695	-1.12304	5.95E-12	5.67E-08	0.000163	5.82E-06	1.30E-05	1708	2868	5356	4057	4362
PTPN6	-1.09907	-1.144	-1.35772	-1.29186	-1.01559	6.01E-12	1.54E-12	4.00E-14	5.67E-12	2.14E-07	1709	1293	1226	1688	3428
TRIR	0.939829	0.775221	0.991334	1.002273	0.888003	6.14E-12	3.11E-08	3.72E-09	1.19E-11	9.36E-08	1710	2740	2829	1792	3264
TALDO1	1.224602	1.193564	1.243601	1.339795	0.992669	6.28E-12	1.14E-10	6.77E-11	1.57E-13	1.56E-08	1711	1849	2150	1286	2898
CHST3	-1.37781	-1.02075	-1.19238	-1.15498	-1.24872	6.29E-12	7.10E-08	2.92E-10	1.30E-09	4.91E-12	1712	2911	2382	2436	1672
OSCAR	-1.27832	-1.03446	-1.15775	-1.29993	-1.19202	6.38E-12	7.26E-08	8.00E-10	2.87E-09	1.11E-11	1714	2922	2547	2574	1781
TOP1	1.437286	1.070445	1.344208	1.215078	1.27984	6.38E-12	1.78E-07	9.42E-11	5.62E-08	2.12E-11	1715	3127	2205	3052	1865
STAU1	1.148169	0.985842	0.91625	1.214603	0.7037	6.58E-12	7.07E-11	3.10E-09	5.88E-13	1.45E-07	1716	1784	2792	1441	3349
CMTM4	1.457818	1.362987	1.713424	1.754111	1.505151	6.67E-12	4.65E-09	4.18E-13	1.78E-12	4.84E-12	1717	2423	1496	1575	1669
SF3A2	1.058441	1.141897	1.009447	1.226601	0.845011	6.73E-12	1.00E-12	2.44E-14	1.24E-14	1.87E-07	1718	1251	1180	1055	3402
AHSA1	1.15046	1.391447	1.592453	1.266726	1.226482	7.00E-12	4.74E-14	3.36E-16	8.84E-11	8.55E-10	1719	964	854	2053	2404
PSMB7	1.111548	0.93906	1.196012	1.062577	1.048689	7.04E-12	1.12E-11	3.48E-11	3.77E-13	6.37E-12	1720	1546	2066	1391	1702
TACC2	1.412988	0.992532	1.458118	1.050276	1.222378	7.05E-12	2.93E-09	4.02E-17	2.80E-10	1.20E-10	1721	2343	702	2198	2076
POLR2K	1.233569	1.516585	1.622792	1.370759	1.369149	7.13E-12	9.67E-16	4.46E-17	8.71E-15	3.05E-15	1722	698	706	1017	875
FOXM1	1.306058	1.445361	1.283261	1.308742	1.130195	7.14E-12	1.42E-12	6.19E-15	3.04E-12	5.82E-10	1723	1284	1082	1627	2334
VSTM4	-1.82697	-1.5593	-2.09418	-1.83005	-1.87654	7.14E-12	9.81E-12	4.87E-14	4.09E-12	2.80E-14	1724	1528	1240	1655	1091
CHCHD3	1.074303	1.141053	1.097535	1.059169	1.111634	7.20E-12	9.86E-10	1.17E-11	6.14E-10	2.63E-12	1725	2172	1892	2315	1596
RUVBL2	1.021404	1.248461	1.013913	1.079577	0.968421	7.23E-12	2.72E-11	2.48E-10	6.76E-11	2.22E-09	1726	1661	2355	2016	2558
MRPL17	0.730458	0.69573	0.986238	0.96869	0.797834	7.29E-12	1.04E-08	1.06E-09	3.31E-10	2.21E-06	1727	2558	2587	2218	3908
RUNX1	-1.67363	-1.65652	-1.24748	-1.47997	-1.45967	7.32E-12	3.49E-13	9.69E-08	2.45E-11	4.52E-09	1728	1158	3505	1893	2675
TPI1	1.632241	1.266038	1.62269	1.451908	1.478853	7.34E-12	2.69E-08	4.24E-12	2.30E-10	4.09E-11	1729	2721	1761	2168	1931
ADI1	0.920498	0.894457	0.814287	0.932849	0.847016	7.34E-12	8.17E-11	5.26E-12	1.84E-12	3.29E-12	1730	1798	1794	1578	1625
ABCC3	2.063095	1.796332	2.571882	1.722929	2.253265	7.35E-12	1.00E-11	3.42E-16	3.12E-11	1.92E-17	1731	1533	855	1913	533
PBXIP1	-1.40754	-1.0926	-1.44476	-1.2387	-1.35901	7.39E-12	8.97E-10	4.12E-13	2.18E-09	7.04E-12	1732	2160	1492	2530	1713
PTH1R	-0.88206	-0.72515	-0.73926	-0.8892	-1.06461	7.53E-12	5.65E-10	1.17E-09	5.82E-11	1.17E-13	1733	2090	2613	1994	1237
CALM3	0.845365	0.742136	0.929782	0.669507	0.840346	7.58E-12	1.46E-07	5.64E-11	1.16E-06	4.72E-12	1734	3079	2125	3681	1664
FOXN3	-1.24564	-0.88182	-0.94961	-0.87587	-1.08018	7.65E-12	1.87E-05	4.89E-08	1.40E-05	3.58E-13	1735	4194	3366	4306	1355
CPZ	-2.47245	-2.00891	-2.49196	-2.00895	-2.5993	7.66E-12	4.51E-07	1.32E-09	3.81E-07	2.39E-13	1736	3294	2627	3433	1313
HLA-DOB	-1.02841	-0.7436	-1.36114	-0.99741	-1.0851	7.76E-12	5.85E-09	4.18E-14	5.14E-09	2.12E-07	1737	2470	1228	2662	3425
SECISBP2L	-1.22894	-1.16848	-1.33148	-1.01735	-1.38245	7.76E-12	3.66E-10	2.74E-14	2.07E-08	2.69E-08	1738	2029	1191	2895	2995
DUOXA1	-0.97734	-0.64733	-0.7733	-0.84002	-0.84225	7.87E-12	1.53E-06	2.96E-07	2.29E-09	8.52E-09	1739	3588	3746	2539	2778
TJP3	1.428166	1.54676	2.072278	1.758559	1.789187	7.92E-12	4.73E-12	8.02E-15	1.28E-11	4.88E-14	1740	1428	1104	1803	1149
SERPINB1	2.079021	1.69065	2.292888	1.796424	2.45458	8.04E-12	1.35E-09	2.87E-15	2.94E-10	9.14E-18	1741	2221	1015	2206	492
CYC1	1.560355	1.711682	1.901164	1.792198	1.716032	8.10E-12	2.08E-15	4.76E-14	3.71E-14	1.02E-16	1742	738	1236	1154	644
CARD8	-1.59604	-1.62336	-2.18709	-1.74355	-2.08728	8.10E-12	1.45E-10	2.99E-20	2.10E-11	5.35E-20	1743	1883	358	1867	266
TMEM107	-0.82645	-0.80359	-0.84801	-0.8134	-0.53747	8.17E-12	3.85E-10	1.27E-10	5.52E-07	0.001511	1744	2034	2253	3519	5798
PRNP	-1.70147	-1.2627	-1.37397	-1.44626	-1.72014	8.40E-12	9.64E-11	1.85E-12	3.64E-11	6.99E-13	1745	1828	1657	1940	1444
COPS5	0.894328	0.76115	1.09399	0.787868	0.494192	8.46E-12	4.13E-10	2.00E-12	1.17E-07	0.007476	1746	2043	1666	3196	6408
OR56A1	-0.87207	-0.54192	-0.8072	-0.77441	-0.95529	8.53E-12	0.000167	4.88E-07	2.06E-06	6.82E-11	1747	4853	3862	3810	2000
PHLDA2	1.581832	1.297704	1.809627	1.45434	1.326224	8.66E-12	3.02E-09	8.60E-12	5.17E-12	4.25E-12	1748	2350	1847	1679	1654
BOC	-1.99977	-1.27782	-1.79137	-1.48778	-2.0243	8.82E-12	2.45E-07	6.65E-13	7.21E-08	2.11E-16	1749	3183	1545	3099	696
ZFYVE16	-1.00273	-0.82064	-0.91873	-0.85931	-1.02757	9.03E-12	1.25E-09	5.26E-12	5.51E-09	7.98E-14	1750	2205	1793	2669	1202
FABP1	3.035244	3.362681	3.540835	3.07983	3.300412	9.11E-12	6.26E-15	3.99E-15	5.11E-14	2.31E-14	1751	807	1047	1178	1067
ENTPD4	-1.2645	-1.29921	-1.35838	-1.25604	-1.33141	9.20E-12	4.83E-13	1.67E-13	3.53E-11	7.51E-13	1752	1196	1395	1934	1448
IFRD2	1.667327	1.613503	1.51241	1.656934	1.543072	9.20E-12	5.15E-12	3.17E-11	1.78E-12	1.18E-13	1753	1444	2053	1574	1238
GMPS	1.063777	1.18828	1.227916	1.25356	1.030832	9.24E-12	3.75E-13	1.74E-15	1.53E-13	1.08E-10	1754	1167	982	1282	2063

PAPSS1	-1.00982	-0.95314	-0.91277	-0.99395	-0.92172	9.38E-12	8.06E-14	1.20E-10	8.90E-13	1.11E-08	1755	1020	2242	1484	2838
AP1S1	1.585946	1.62848	1.557951	1.608722	1.486061	9.52E-12	9.75E-12	1.45E-10	5.71E-13	5.87E-12	1756	1527	2275	1437	1692
LFNG	1.525257	1.456711	1.133253	1.500105	1.380197	9.58E-12	5.86E-14	4.43E-10	1.85E-14	4.59E-12	1757	991	2448	1090	1661
GAS2L1	-1.17285	-0.97237	-1.24898	-1.06495	-1.31289	9.60E-12	3.67E-09	3.64E-13	3.11E-10	2.22E-14	1758	2386	1477	2210	1065
MTRNR2L1	1.220541	1.393469	1.221073	1.492074	1.490912	9.64E-12	2.62E-12	3.38E-14	1.11E-12	3.02E-15	1759	1350	1207	1519	872
NDFIP2	1.608706	1.680981	1.54064	1.63244	1.875463	9.74E-12	2.30E-14	2.53E-13	3.86E-15	2.01E-16	1760	906	1447	954	689
PYGB	2.037157	1.877702	2.372823	2.213124	1.780974	9.75E-12	1.43E-11	7.17E-13	1.92E-14	1.55E-08	1761	1571	1556	1093	2895
FSTL3	-1.43403	-1.08554	-1.07805	-1.57889	-1.56875	9.82E-12	3.56E-08	2.31E-07	1.22E-09	6.23E-12	1762	2777	3689	2423	1695
OR9A2	-0.72966	-0.40401	-0.62574	-0.70853	-0.59681	9.82E-12	0.000496	1.72E-05	4.23E-07	6.26E-06	1763	5249	4744	3455	4160
SLFN5	-1.4048	-1.31987	-1.53772	-1.16341	-1.51464	9.84E-12	1.82E-10	1.93E-15	6.68E-09	4.08E-10	1764	1909	990	2698	2273
S100A14	2.5091	2.464355	3.017659	2.334078	2.181263	1.02E-11	6.21E-15	4.21E-15	3.34E-11	4.70E-11	1765	806	1049	1923	1947
BDH1	1.988146	1.782508	2.167743	1.735988	1.847709	1.03E-11	3.26E-10	2.58E-16	1.08E-11	1.37E-11	1766	2006	837	1774	1809
CDH3	2.001712	1.952658	1.970992	1.840063	1.624944	1.04E-11	3.54E-12	3.62E-14	1.01E-12	8.80E-08	1767	1386	1213	1505	3252
LAMA2	-1.22376	-0.98775	-0.94051	-1.09337	-1.16823	1.05E-11	3.75E-08	9.98E-09	1.33E-09	7.20E-10	1768	2784	3012	2444	2372
COL11A1	-2.64328	-2.65698	-2.35983	-2.53183	-2.7669	1.06E-11	4.23E-10	8.17E-11	7.75E-10	1.03E-11	1769	2046	2177	2359	1768
CDK11A	0.847639	1.06348	0.824666	0.920958	0.896444	1.06E-11	3.26E-11	8.26E-09	1.27E-09	1.01E-08	1770	1677	2978	2430	2818
HMOX1	-2.26523	-2.39944	-2.15602	-2.81685	-2.71372	1.09E-11	2.37E-11	5.66E-14	1.15E-14	2.63E-15	1771	1639	1257	1048	865
HNRNPK	1.139859	0.886373	1.179239	0.991407	1.081613	1.10E-11	1.05E-08	3.46E-11	1.40E-09	1.35E-13	1772	2561	2065	2457	1252
SGCD	-1.55749	-1.44487	-1.45632	-1.44316	-1.83749	1.12E-11	3.31E-10	3.35E-10	5.28E-10	1.24E-14	1773	2009	2407	2286	1010
CNN3	-1.97801	-2.01121	-2.03555	-1.70905	-1.8102	1.14E-11	7.59E-12	2.32E-13	1.07E-11	2.04E-08	1774	1500	1434	1768	2947
ZNF528	-0.89168	-0.70741	-0.80593	-0.87224	-0.71634	1.14E-11	1.84E-07	1.58E-08	2.60E-11	6.44E-06	1775	3132	3115	1898	4164
LCE1D	-1.00255	-0.69588	-1.1005	-1.10052	-1.02469	1.15E-11	1.05E-06	1.52E-11	4.01E-11	2.18E-09	1776	3493	1926	1947	2557
PRKCZ	1.184415	1.082583	1.124398	1.104001	1.238978	1.16E-11	3.56E-09	2.36E-11	6.15E-09	3.56E-11	1777	2381	2003	2686	1915
PDIA6	1.411181	1.074553	1.190632	1.428539	1.328527	1.16E-11	9.92E-10	1.12E-09	1.64E-13	1.04E-12	1778	2173	2602	1293	1488
UFSP2	-0.816	-0.79827	-0.99291	-0.90863	-1.08935	1.16E-11	2.76E-09	1.01E-12	4.03E-10	4.22E-14	1779	2333	1593	2249	1142
OR5K4	-1.42715	-1.06768	-1.69153	-1.46239	-1.89939	1.16E-11	1.53E-10	3.06E-12	7.22E-09	1.95E-13	1780	1889	1726	2707	1293
TAL1	-1.02065	-0.5431	-1.2064	-0.75592	-0.88536	1.16E-11	6.11E-06	1.31E-12	1.59E-06	2.54E-08	1781	3909	1624	3747	2983
WNK4	1.343379	1.161145	1.275999	1.351807	1.725455	1.16E-11	3.18E-09	1.16E-08	2.14E-11	1.09E-11	1782	2360	3048	1868	1776
CLDN12	1.628674	1.773295	2.163701	1.913361	1.856671	1.16E-11	1.02E-12	6.31E-18	3.07E-13	4.73E-18	1783	1255	603	1364	448
RAD23B	0.781709	0.856844	0.748374	0.748079	0.706292	1.18E-11	2.29E-11	2.24E-10	4.38E-11	2.87E-07	1784	1634	2340	1957	3492
PMPCB	0.883984	1.059755	0.778365	0.988101	1.091049	1.18E-11	3.94E-14	6.22E-10	7.79E-12	3.71E-12	1785	950	2501	1722	1636
APOC1	-2.18807	-2.632	-2.48966	-2.2886	-3.11628	1.20E-11	2.71E-12	1.08E-14	7.94E-13	1.83E-17	1786	1356	1122	1471	528
ECHS1	1.328999	1.257571	1.579015	1.190641	1.345565	1.22E-11	2.18E-13	2.99E-15	3.52E-11	5.11E-13	1787	1105	1019	1931	1397
CLRN3	1.745309	1.552157	1.73671	1.717902	2.078923	1.22E-11	5.35E-12	5.23E-16	7.61E-12	6.88E-15	1788	1450	883	1718	948
SPP1	-2.43056	-2.50872	-3.01958	-2.41338	-3.34821	1.23E-11	1.26E-10	8.80E-12	2.83E-12	7.32E-15	1789	1861	1855	1620	953
NSUN5	1.261824	1.494269	0.942293	1.210201	1.369116	1.24E-11	1.98E-15	5.43E-08	1.08E-11	1.29E-12	1790	737	3391	1770	1513
DDOST	1.365834	1.136275	1.553934	1.3338	1.500126	1.24E-11	3.68E-10	1.22E-11	2.34E-10	2.03E-15	1791	2030	1898	2170	851
BCAS3	-0.89312	-0.70336	-0.98032	-0.73697	-0.64876	1.28E-11	1.14E-07	2.50E-14	1.07E-07	2.78E-07	1792	3021	1182	3179	3486
CXCL2	1.721066	1.247533	1.638444	1.429841	0.903751	1.29E-11	3.00E-06	4.92E-08	1.70E-07	0.000559	1793	3761	3368	3269	5437
TNFRSF21	1.632308	1.282726	1.532973	1.32669	1.291457	1.29E-11	1.61E-06	2.09E-08	9.59E-08	9.23E-11	1794	3598	3174	3159	2041
POLR1C	1.08967	1.123616	1.034438	0.934429	0.999621	1.34E-11	1.41E-11	2.91E-11	4.00E-10	4.53E-13	1795	1569	2044	2247	1386
SERINC2	2.134323	2.005722	2.782198	2.311592	2.360009	1.34E-11	1.68E-11	2.71E-18	3.02E-17	9.97E-16	1796	1594	539	625	805
UCKL1	1.069625	1.129192	1.185313	1.201223	1.011829	1.39E-11	5.55E-12	6.96E-16	1.36E-12	9.78E-13	1797	1452	902	1540	1481
DDT	1.553794	1.487386	1.74192	1.522558	1.516808	1.39E-11	7.10E-11	1.06E-12	4.24E-13	5.45E-15	1798	1785	1603	1405	926
GZMA	-1.16435	-1.02003	-1.34782	-1.26956	-1.57802	1.40E-11	1.51E-10	8.21E-12	9.23E-12	6.16E-16	1799	1888	1844	1746	767
FAM83F	1.668326	1.840728	1.537035	2.071927	1.545901	1.40E-11	2.10E-13	5.98E-12	4.32E-15	5.62E-13	1800	1102	1807	963	1410
SPRY4	-1.91361	-2.04962	-2.02607	-2.17799	-1.69717	1.40E-11	1.54E-15	4.15E-13	2.25E-16	2.27E-09	1801	725	1493	751	2567
GXYLT2	-1.187	-0.78926	-0.90653	-0.77478	-1.15719	1.42E-11	1.21E-06	8.10E-09	1.49E-06	2.02E-13	1802	3530	2970	3732	1295
TM9SF4	1.198703	1.056012	1.14267	1.368935	0.773728	1.43E-11	2.57E-09	1.39E-11	4.75E-12	7.21E-07	1803	2318	1912	1672	3668
XPO1	0.961497	0.74213	1.159233	0.868998	0.977689	1.45E-11	3.65E-07	2.38E-11	3.07E-07	1.36E-10	1804	3257	2005	3382	2098
PHF23	-0.90746	-0.68747	-0.71354	-0.88121	-1.1366	1.45E-11	2.97E-07	1.92E-09	8.17E-09	1.84E-09	1805	3217	2688	2721	2525
NKG7	-1.73024	-1.89097	-1.93544	-2.10079	-1.81295	1.46E-11	5.71E-11	4.27E-19	6.03E-13	7.92E-09	1806	1763	454	1445	2764
ANKRD16	-0.94564	-0.70066	-0.92994	-0.90975	-1.10423	1.47E-11	7.94E-07	1.34E-11	3.61E-11	4.15E-11	1807	3422	1908	1938	1932
SLC11A1	-1.31166	-1.05043	-1.24533	-1.19425	-1.55536	1.48E-11	2.52E-05	2.90E-08	5.02E-07	3.17E-11	1808	4278	3251	3494	1901
RNPS1	0.977128	1.046911	0.957712	1.148287	1.133149	1.48E-11	2.76E-14	4.82E-12	1.53E-15	2.75E-14	1809	924	1778	874	1089
PHETA2	-1.22323	-1.15625	-1.03089	-1.03422	-1.34414	1.48E-11	1.96E-12	5.89E-13	1.18E-10	2.92E-15	1810	1320	1533	2081	871
SLC2A1	1.822641	1.763051	2.107019	1.814012	1.911815	1.49E-11	3.61E-09	2.21E-12	1.25E-11	2.10E-10	1811	2385	1677	1800	2167
RCN3	-2.04666	-2.10461	-1.79243	-1.97202	-1.94833	1.50E-11	1.48E-10	2.01E-09	1.19E-10	1.08E-10	1812	1887	2704	2084	2061
C3	-3.74763	-3.61394	-2.95638	-4.02631	-4.30597	1.50E-11	1.90E-13	9.21E-12	2.64E-13	1.12E-14	1813	1097	1859	1345	999
SF3B3	1.380553	1.191544	1.307526	1.28843	1.502552	1.51E-11	4.37E-10	9.30E-12	2.15E-11	7.95E-14	1814	2048	1862	1869	1201
PRRC2A	1.077875	0.975251	1.139422	0.893324	1.337028	1.51E-11	5.46E-10	1.20E-12	6.39E-09	2.74E-16	1815	2083	1616	2691	717
SDHC	1.023067	0.917645	1.064542	1.038566	1.070055	1.51E-11	2.38E-11	3.81E-11	1.28E-13	1.31E-12	1816	1640	2073	1261	1516
IDH1	1.407246	1.227252	1.724682	1.480294	1.276994	1.54E-11	6.77E-11	9.15E-14	7.89E-12	6.25E-11	1817	1782	1310	1727	1982
ALYREF	1.573007	1.639381	1.617162	1.656148	1.701533	1.55E-11	1.16E-12	6.24E-12	9.03E-14	3.85E-15	1818	1261	1808	1237	895
SPOCK1	-1.75226	-1.50555	-1.40701	-1.77139	-2.10436	1.58E-11	4.42E-09	1.44E-08	3.77E-11	1.98E-12	1819	2413	3099	1943	1552
CD47	1.408878	1.433859	1.385716	1.607101	1.225937	1.60E-11	1.96E-10	1.14E-08	2.19E-11	1.00E-10	1820	1919	3043	1876	2054
SH3YL1	1.213208	1.377962	1.406733	1.602928	1.804851	1.63E-11	1.71E-13	6.37E-17	2.10E-14	2.15E-16	1821	1087	728	1099	699
SDHA	1.085173	0.935604	0.98178	0.88047	0.938485	1.65E-11	1.28E-06	1.24E-08	3.43E-06	1.78E-09	1822	3546	3067	3924	2521
LIPA	-1.68177	-1.84062	-1.82833	-1.70439	-1.92587	1.66E-11	3.15E-13	1.28E-14	5.03E-12	8.57E-14	1823	1143	1135	1677	1208
ABI3BP	-1.11893	-0.9456	-0.83964	-1.11534	-1.06862	1.67E-11	4.88E-12	1.08E-08	3.49E-10	4.37E-12	1824	1434	3027	2226	1658
OSM	-0.95908	-0.86299	-0.97733	-0.98776	-1.01661	1.70E-11	2.06E-08	2.24E-08	2.87E-10	3.48E-09	1825	2671	3184	2201	2627
ZNF461	-0.97304	-0.57537	-1.12231	-0.77981	-0.65902	1.70E-11	2.81E-08	1.66E-11	1.79E-06	0.000231	1826	2727	1934	3770	5158
UBA1	1.070667	0.728893	0.778635	1.054051	0.728899	1.72E-11	4.19E-07	9.38E-08	3.97E-10	1.27E-06	1827	3277	3497	2245	3786

TRAM2	-1.2679	-1.27221	-1.67662	-1.40641	-1.49971	1.76E-11	3.46E-12	2.35E-17	1.40E-11	3.24E-17	1828	1385	677	1811	563
CTSD	-1.93503	-2.2913	-2.00702	-2.18071	-2.18854	1.76E-11	2.22E-14	2.57E-12	1.36E-12	1.05E-15	1829	902	1696	1541	810
ZC3H15	1.081205	1.031052	1.550359	1.246723	1.032926	1.77E-11	2.23E-09	3.91E-11	2.43E-09	1.53E-09	1830	2295	2075	2549	2493
AHDC1	-1.36532	-1.31895	-1.15414	-1.21363	-1.36207	1.77E-11	7.35E-12	7.96E-10	5.01E-11	1.09E-14	1831	1491	2545	1976	996
CKS2	1.888517	1.967424	1.975585	1.865598	1.67936	1.78E-11	9.35E-12	6.96E-12	4.12E-11	2.07E-10	1832	1521	1828	1952	2164
CYP2S1	1.716491	2.064504	2.506937	2.122488	2.310958	1.79E-11	2.05E-14	2.42E-17	3.00E-14	1.59E-15	1833	888	681	1133	840
LAT	-1.01969	-0.86773	-1.40464	-1.17017	-1.24744	1.79E-11	2.34E-08	1.06E-12	5.36E-10	2.51E-10	1834	2690	1602	2290	2198
CPA3	-1.91777	-1.19929	-1.50186	-1.57349	-1.68094	1.80E-11	4.53E-06	7.89E-07	3.33E-08	3.82E-10	1835	3840	3977	2975	2256
MGST3	1.505204	0.921573	1.415781	1.17685	1.228954	1.84E-11	3.44E-08	4.38E-10	1.61E-09	1.05E-09	1836	2767	2444	2484	2432
FAM90A1	-1.23244	-0.77967	-1.10326	-0.82196	-0.66613	1.85E-11	2.04E-06	9.44E-09	1.25E-05	0.002139	1837	3664	3001	4270	5938
MFNG	-1.13759	-1.18537	-1.63592	-1.33762	-1.42124	1.86E-11	2.51E-11	3.19E-12	3.11E-10	1.30E-10	1838	1650	1732	2209	2087
KREMEN1	-1.20064	-1.00412	-0.81727	-1.30385	-1.25489	1.87E-11	2.04E-10	1.02E-07	9.60E-12	4.23E-09	1839	1927	3522	1753	2661
OBSCN	-0.85405	-0.50208	-0.95365	-0.81981	-0.60119	1.87E-11	1.49E-06	1.86E-08	3.11E-07	0.000165	1840	3582	3152	3387	5067
SSU72	1.020987	0.930942	1.178185	1.063412	0.888484	1.88E-11	8.61E-10	2.30E-13	1.27E-10	1.60E-08	1841	2150	1432	2092	2904
OR8G1	-1.03747	-0.80167	-1.20176	-1.05871	-1.08238	1.88E-11	1.10E-10	5.21E-16	4.32E-12	2.71E-14	1842	1841	882	1663	1084
RPL31	1.970505	2.001408	1.821103	2.174318	2.142367	1.89E-11	5.77E-13	9.47E-11	1.11E-16	2.60E-14	1843	1207	2208	705	1078
NME1	1.999669	1.913346	1.925636	1.862871	2.016034	1.94E-11	1.79E-13	8.02E-12	1.18E-14	8.58E-17	1844	1092	1841	1051	628
ATM	-1.10195	-1.13605	-1.05313	-1.1881	-1.04609	1.95E-11	3.33E-12	1.54E-13	3.75E-13	2.02E-11	1845	1378	1383	1389	1861
CAPRIN1	1.156629	1.026116	1.342423	1.13263	1.28006	1.96E-11	1.66E-10	3.02E-12	4.66E-11	1.26E-11	1846	1899	1723	1965	1794
NET1	1.36914	1.220911	1.73824	1.443826	1.637818	1.96E-11	2.89E-08	2.73E-14	6.11E-11	1.28E-16	1847	2732	1190	2000	662
KCTD12	-1.53524	-1.94727	-1.68885	-1.83875	-1.68176	1.96E-11	9.47E-15	5.93E-13	4.36E-13	5.41E-13	1848	829	1536	1406	1403
MOCS1	-1.12062	-0.71669	-1.12086	-0.81506	-0.90409	1.97E-11	1.31E-08	1.31E-12	8.80E-09	8.42E-09	1849	2602	1623	2738	2773
KRTAP10-:	-1.11898	-0.82979	-1.26233	-1.1281	-1.01478	2.01E-11	1.38E-06	1.49E-10	4.09E-10	3.22E-08	1850	3561	2278	2250	3031
SNX29	-1.16685	-1.02357	-1.21981	-0.87049	-1.07241	2.04E-11	1.43E-10	1.93E-13	9.44E-09	6.88E-11	1851	1882	1412	2751	2002
NPR2	-0.91398	-0.39805	-0.6456	-0.49452	-0.44875	2.06E-11	0.007645	2.83E-06	0.001452	0.000835	1852	6305	4262	5765	5575
MRPS25	1.030934	1.355529	1.248648	1.341038	1.04647	2.06E-11	1.19E-14	1.05E-13	2.56E-14	2.72E-12	1853	845	1322	1116	1603
AURKA	1.608326	1.757031	1.26895	1.826645	1.042505	2.06E-11	1.01E-12	6.74E-12	1.94E-13	5.78E-09	1854	1253	1821	1310	2706
SRC	1.501808	1.207981	1.268716	1.441828	1.132762	2.07E-11	1.21E-06	5.53E-09	1.83E-09	3.68E-08	1855	3529	2896	2504	3057
OR4C3	-0.83016	-0.76283	-0.62757	-0.85071	-0.81674	2.10E-11	8.39E-09	3.84E-08	3.60E-10	1.21E-10	1856	2525	3322	2230	2077
RBBP7	1.301603	1.249542	1.201909	1.462129	1.010253	2.11E-11	4.59E-11	1.64E-09	2.00E-14	4.14E-08	1857	1736	2653	1095	3081
SF3B6	1.274982	1.309379	1.319683	1.273663	1.297276	2.12E-11	1.46E-12	7.32E-16	3.07E-12	4.18E-18	1858	1289	907	1628	441
LINGO1	-1.45217	-1.2509	-1.88298	-1.65208	-1.51178	2.15E-11	1.56E-09	5.38E-13	3.54E-10	7.97E-11	1859	2234	1523	2228	2019
CC2D1A	0.949846	1.214734	1.237084	1.27831	0.97775	2.15E-11	7.62E-11	1.46E-12	9.11E-13	9.37E-09	1860	1790	1636	1486	2800
PIWIL3	-0.7779	-0.50019	-0.83822	-0.6535	-0.65078	2.17E-11	1.04E-05	1.71E-09	3.26E-07	5.09E-07	1861	4038	2660	3395	3605
FBLN2	-2.51317	-2.20181	-1.86867	-2.08774	-2.76386	2.18E-11	1.01E-09	5.02E-09	4.36E-10	2.10E-13	1862	2174	2875	2261	1303
ASCL2	3.097612	3.692386	2.928902	3.747334	3.531167	2.18E-11	2.42E-18	9.04E-11	1.65E-15	3.03E-14	1863	371	2200	884	1101
SYT2	-0.91223	-0.84007	-1.07619	-0.99971	-0.81701	2.18E-11	1.66E-11	1.30E-13	3.51E-11	8.43E-10	1864	1590	1354	1930	2401
PSMB1	1.22049	1.268016	1.402592	1.260968	1.211274	2.20E-11	4.20E-14	2.96E-14	9.74E-14	2.21E-13	1865	955	1194	1242	1308
SRP9	1.009459	1.00824	1.066664	0.966663	1.242814	2.23E-11	2.46E-12	5.26E-12	9.33E-12	3.50E-15	1866	1337	1792	1748	889
CCND1	1.354493	1.17686	1.634033	1.472123	1.040426	2.28E-11	1.51E-08	2.91E-13	7.58E-11	9.72E-07	1867	2627	1459	2033	3729
HOOK1	1.08358	1.153367	0.966985	1.092345	0.956038	2.28E-11	9.56E-15	1.56E-11	2.00E-14	9.26E-10	1868	831	1930	1094	2416
PDLIM4	-1.9165	-1.83233	-1.84449	-1.92508	-2.12739	2.28E-11	1.39E-08	1.10E-11	9.51E-09	5.18E-13	1869	2615	1887	2753	1400
RPS3	2.256744	2.25996	2.062225	2.413881	2.243453	2.29E-11	2.30E-13	1.22E-11	3.61E-15	6.87E-16	1870	1110	1897	943	773
TRANK1	-1.46511	-1.25787	-1.60152	-1.32844	-1.34138	2.29E-11	4.26E-08	1.32E-10	1.06E-08	1.56E-09	1871	2816	2261	2771	2498
C17orf49	-1.02217	-0.93578	-0.7855	-0.90994	-0.74023	2.30E-11	2.73E-10	2.46E-10	3.42E-09	1.09E-07	1872	1970	2354	2607	3295
H4C6	-2.572	-1.97579	-2.23734	-2.20999	-2.54428	2.35E-11	4.15E-09	1.09E-09	1.97E-09	3.97E-14	1873	2407	2593	2514	1131
PRL	-0.82145	-0.72967	-0.76594	-0.90545	-0.8215	2.37E-11	1.70E-09	8.14E-11	2.98E-14	6.35E-12	1874	2248	2175	1131	1699
OR4A15	-0.82463	-0.65147	-0.76122	-0.83535	-0.77036	2.37E-11	4.96E-06	2.66E-07	3.53E-09	6.48E-07	1875	3860	3720	2611	3652
ROCK1	-1.01093	-1.0413	-1.10006	-1.11241	-1.18829	2.41E-11	5.02E-12	4.22E-14	9.71E-13	2.12E-13	1876	1438	1229	1495	1304
MRPL4	0.947581	1.226163	0.869841	1.169652	1.15903	2.47E-11	3.90E-13	1.20E-10	3.07E-14	6.79E-11	1877	1172	2244	1136	1998
BNIP3L	-1.45879	-1.5146	-1.51249	-1.45833	-1.6256	2.52E-11	9.84E-11	8.59E-14	2.42E-11	4.21E-13	1879	1832	1307	1889	1372
DEFB121	-0.76065	-0.41809	-0.59761	-0.5333	-0.70164	2.53E-11	3.27E-05	1.26E-07	4.74E-06	6.92E-09	1880	4356	3567	4005	2736
C1orf43	1.005334	0.666144	0.994966	0.77441	0.605008	2.54E-11	1.42E-07	2.43E-09	1.33E-07	6.05E-05	1881	3074	2741	3218	4768
ERBB2	1.555031	1.656755	1.628173	1.640933	2.121196	2.54E-11	3.83E-12	2.65E-10	5.48E-16	5.70E-13	1882	1398	2363	807	1414
NHSL1	1.266503	0.869302	1.361346	1.061746	1.543841	2.55E-11	3.45E-06	1.27E-10	2.24E-06	6.89E-11	1883	3786	2256	3829	2003
NSMF	1.356476	1.352756	1.581939	1.495328	1.333831	2.55E-11	6.09E-13	1.16E-13	6.90E-12	2.04E-08	1884	1212	1334	1709	2946
CCDC8	-1.21941	-0.80085	-0.96283	-1.13216	-1.33308	2.62E-11	2.05E-07	7.63E-09	4.27E-10	5.35E-12	1885	3156	2955	2254	1679
ARHGAP2:	-1.50605	-1.14255	-1.29431	-1.29539	-1.33659	2.63E-11	8.73E-09	4.43E-13	1.72E-08	6.16E-14	1886	2532	1502	2860	1180
MID1IP1	0.965585	0.745257	0.808656	0.841701	0.69079	2.65E-11	3.95E-08	8.02E-08	2.21E-11	3.00E-07	1887	2796	3461	1881	3504
IGF2	-3.17703	-2.11009	-2.28421	-2.6432	-2.91282	2.66E-11	1.60E-05	5.40E-10	2.40E-07	3.52E-14	1888	4147	2472	3335	1119
TLN1	-1.52745	-1.69462	-1.42141	-1.60497	-1.75945	2.66E-11	5.44E-13	5.28E-11	3.00E-12	2.22E-16	1889	1202	2114	1626	701
SFRP2	-2.29881	-2.56674	-2.12781	-2.55372	-3.25989	2.71E-11	1.25E-09	2.16E-09	5.67E-10	6.03E-13	1891	2206	2717	2295	1422
TSPAN1	2.616374	2.644556	3.219337	2.848196	2.418673	2.76E-11	1.01E-11	1.31E-14	1.80E-15	1.02E-11	1892	1535	1136	892	1765
SELE	-1.51567	-1.53977	-1.72903	-1.62329	-1.25452	2.77E-11	1.54E-10	1.87E-11	5.00E-13	3.11E-06	1893	1892	1966	1422	3988
GOLGA8N	-1.44837	-0.97377	-1.58254	-1.31969	-1.34371	2.82E-11	3.20E-09	2.47E-13	3.93E-10	7.87E-13	1894	2364	1443	2243	1454
SMOC2	-1.75062	-1.09976	-1.43149	-1.09856	-1.0022	2.83E-11	5.77E-07	2.01E-09	2.00E-06	1.86E-06	1895	3344	2705	3804	3866
PTGDS	-1.43642	-0.78737	-1.0739	-1.02438	-1.28835	2.84E-11	2.70E-05	2.49E-08	3.02E-07	1.06E-12	1896	4303	3210	3376	1491
IARS2	1.136755	1.125888	0.963073	1.218792	1.045285	2.85E-11	1.00E-11	1.14E-10	5.55E-16	1.78E-11	1897	1532	2234	809	1841
BCL2A1	-1.86276	-1.77251	-1.59863	-1.80296	-1.87453	2.91E-11	3.63E-08	2.67E-09	2.31E-10	4.39E-11	1898	2779	2763	2169	1939
DENND2B	-1.9407	-1.76195	-1.34614	-1.63878	-1.42171	2.92E-11	1.57E-12	3.66E-08	4.09E-10	8.13E-08	1899	1299	3308	2251	3237
COPZ1	0.982823	0.865906	0.821426	0.803781	0.9384	2.94E-11	6.12E-11	5.62E-11	1.42E-08	9.10E-12	1900	1769	2123	2821	1750
SF3B5	1.062569	0.979188	1.16155	1.08443	1.082529	2.98E-11	6.98E-09	4.51E-12	1.07E-09	2.65E-12	1901	2494	1766	2405	1600
PIK3AP1	-1.52588	-1.6583	-1.46308	-1.69532	-1.61431	3.00E-11	3.10E-10	1.21E-13	3.25E-11	2.72E-12	1902	1997	1341	1918	1602

PSMA3	1.144127	1.019741	1.490958	1.087546	1.420563	3.02E-11	3.81E-11	1.70E-12	1.09E-09	2.66E-14	1903	1708	1654	2411	1082
METTL2B	0.814932	1.033061	1.002024	0.814285	0.663571	3.03E-11	1.51E-12	5.32E-15	2.69E-11	2.93E-05	1904	1292	1071	1901	4552
PTGES2	1.436911	1.504536	1.727165	1.64212	1.596661	3.03E-11	2.99E-11	3.15E-13	8.10E-12	5.11E-14	1905	1668	1465	1729	1155
ETFB	1.097328	0.932379	1.100263	0.938132	1.10625	3.04E-11	3.01E-08	7.09E-11	3.75E-08	1.19E-13	1906	2735	2156	2994	1242
SPDYE3	-0.89408	-0.64216	-1.09066	-0.95501	-0.82026	3.04E-11	2.56E-09	1.55E-12	4.31E-11	4.21E-07	1907	2316	1641	1955	3573
VDAC2	1.47107	1.444969	2.005452	1.511955	1.440904	3.06E-11	3.55E-13	9.69E-19	5.54E-12	5.11E-14	1908	1161	489	1685	1156
PARP4	1.530969	1.317459	1.61394	1.519903	1.677305	3.09E-11	9.09E-09	4.97E-12	1.19E-09	1.11E-10	1909	2538	1783	2420	2067
CRLS1	1.452823	1.741945	1.289624	1.553072	1.182537	3.15E-11	1.68E-13	2.05E-10	2.36E-10	1.12E-07	1910	1082	2327	2174	3302
PANK4	-0.80421	-0.65492	-0.60996	-0.6768	-0.56925	3.17E-11	7.37E-08	1.68E-08	4.80E-07	3.60E-05	1911	2928	3129	3487	4622
HDGFL1	-0.85363	-0.66867	-0.75691	-0.8743	-0.56162	3.18E-11	7.17E-07	1.97E-10	3.39E-10	1.55E-08	1912	3397	2318	2224	2896
HSD11B2	1.663934	1.620802	1.78286	1.924766	1.950204	3.21E-11	1.03E-11	3.18E-12	3.08E-13	5.74E-15	1913	1537	1731	1365	932
RBIS	1.097335	1.133243	1.401712	1.093895	1.043013	3.25E-11	1.82E-11	1.03E-13	2.15E-08	1.69E-08	1914	1603	1318	2902	2911
ARSL	1.437076	1.495258	1.190495	1.534102	1.261053	3.25E-11	1.86E-13	2.91E-11	3.65E-13	9.13E-11	1915	1094	2046	1386	2036
NCL	1.959779	2.014019	2.118343	2.216329	1.736745	3.26E-11	1.18E-12	2.81E-10	3.54E-17	1.05E-11	1916	1266	2374	644	1770
BLOC1S6	-0.75079	-0.85257	-0.76407	-0.89069	-0.82665	3.33E-11	4.36E-11	1.55E-10	5.36E-12	6.11E-10	1917	1729	2286	1682	2341
DOK3	-1.15556	-0.93196	-1.65216	-1.19616	-1.57319	3.34E-11	1.07E-09	2.93E-19	2.93E-09	1.39E-15	1918	2185	437	2578	829
PLD6	-0.8537	-0.65429	-1.01154	-0.79277	-0.96029	3.34E-11	1.04E-08	4.84E-13	4.42E-10	4.42E-13	1919	2559	1508	2264	1382
TMEM100	-1.0835	-0.65989	-0.71913	-0.85206	-0.88171	3.35E-11	1.44E-05	2.76E-07	1.24E-06	3.76E-09	1920	4119	3728	3694	2643
PSMA5	1.197756	0.99767	1.182475	1.322697	1.433965	3.39E-11	6.82E-13	5.13E-11	4.03E-14	2.96E-12	1921	1218	2112	1163	1613
GALNT4	2.010416	1.515125	1.958294	2.201693	1.954466	3.42E-11	1.13E-06	1.51E-10	1.45E-11	6.29E-12	1922	3513	2282	1817	1697
SFRP4	-2.47881	-1.44616	-1.65391	-1.86544	-2.39155	3.44E-11	1.76E-05	1.77E-07	4.75E-07	9.31E-13	1923	4178	3637	3484	1472
AAMP	0.87092	1.018935	0.901189	1.087964	0.977968	3.47E-11	2.32E-10	1.54E-10	1.16E-10	2.92E-09	1924	1948	2285	2078	2600
NUTM2G	-1.13544	-0.9291	-1.17512	-1.08628	-0.79972	3.48E-11	1.33E-07	2.22E-09	2.16E-08	2.20E-06	1925	3057	2725	2904	3905
DENND4C	0.839321	1.03957	1.119301	1.068671	0.847287	3.51E-11	2.94E-11	1.67E-11	9.95E-13	9.82E-07	1926	1667	1935	1503	3733
SLC7A7	-1.38089	-1.32475	-1.28456	-1.49438	-1.29381	3.53E-11	2.08E-10	6.29E-10	8.03E-13	5.85E-07	1927	1932	2502	1473	3635
XRCC6	1.042331	1.014925	1.144279	1.240305	1.259177	3.57E-11	1.76E-09	5.50E-10	3.08E-11	1.88E-11	1928	2251	2474	1909	1852
CSNK2B	1.174216	0.854766	0.906141	0.79085	0.854447	3.58E-11	1.66E-07	1.13E-08	1.45E-06	2.00E-08	1929	3104	3038	3722	2942
BTN3A3	-1.07521	-0.90601	-0.75327	-0.9257	-1.04327	3.60E-11	5.80E-09	2.01E-07	4.25E-12	6.69E-08	1930	2465	3659	1660	3183
TOMM6	1.541831	1.311602	1.579031	1.35685	1.448787	3.60E-11	3.99E-10	2.00E-11	1.78E-10	3.90E-12	1931	2037	1977	2128	1644
MIF	1.752605	1.723024	1.866556	1.771212	1.726658	3.61E-11	1.74E-12	3.45E-12	6.19E-12	1.60E-11	1932	1309	1739	1698	1826
STOML1	-0.9145	-0.53203	-1.02511	-0.73406	-0.73917	3.64E-11	0.000199	1.66E-09	2.25E-05	8.01E-08	1933	4915	2655	4417	3229
MYO19	0.984863	0.901246	1.004915	1.046009	0.894209	3.68E-11	1.58E-10	4.37E-13	1.18E-12	4.07E-13	1934	1895	1499	1523	1371
EIF4H	1.058827	0.956957	1.190678	1.093079	1.310238	3.68E-11	1.75E-11	2.15E-12	1.84E-12	1.19E-15	1935	1597	1676	1577	818
SGCA	-0.9875	-0.62631	-0.95497	-0.90855	-0.93105	3.69E-11	2.67E-05	4.39E-10	4.61E-09	1.03E-09	1936	4300	2445	2650	2427
RPL7A	1.725319	1.767905	1.500858	1.866455	1.782296	3.72E-11	5.07E-12	2.19E-09	3.82E-15	4.04E-11	1937	1440	2720	952	1928
GTF2I	0.850738	0.775846	0.964418	0.82792	1.007294	3.72E-11	1.40E-08	4.46E-09	2.19E-08	2.54E-12	1938	2617	2857	2905	1593
ATRN	-1.17661	-0.67166	-1.03288	-0.86934	-1.40213	3.78E-11	5.94E-05	1.12E-07	1.66E-05	8.68E-09	1939	4533	3544	4352	2784
RDH13	0.878495	1.062563	1.087757	0.860822	0.925431	3.79E-11	1.50E-11	2.56E-13	1.03E-08	4.86E-10	1940	1577	1448	2767	2301
PLOD1	-1.34302	-1.42294	-1.37871	-1.25217	-1.47188	3.79E-11	1.98E-12	7.46E-12	7.10E-11	6.08E-13	1941	1321	1835	2025	1427
OR4C12	-0.90247	-0.42856	-0.73259	-0.66638	-0.69192	3.92E-11	0.000237	2.07E-07	6.45E-07	5.37E-10	1942	4969	3664	3556	2314
MRPL3	1.382585	1.251692	1.374477	1.211484	1.267878	3.93E-11	2.24E-09	9.24E-11	6.37E-10	5.01E-10	1943	2296	2201	2324	2312
GOLGA6L(	-1.12064	-0.60048	-1.15693	-0.89711	-0.75525	3.98E-11	1.49E-05	9.89E-11	1.47E-06	3.66E-05	1944	4131	2216	3724	4626
GPR15	-1.65328	-1.02912	-1.85465	-1.7212	-1.91844	3.98E-11	0.00022	5.18E-08	2.44E-06	2.90E-08	1945	4943	3382	3847	3016
TRNAU1AI	-0.7228	-0.58042	-0.51587	-0.48567	-0.42847	4.00E-11	1.97E-07	1.55E-07	2.34E-05	0.000101	1946	3144	3607	4426	4935
ISCU	-0.751	-0.68175	-0.90453	-0.64769	-0.81335	4.02E-11	2.67E-09	7.52E-14	7.44E-09	2.12E-10	1947	2323	1293	2710	2170
ZDHHC3	1.047561	1.133934	1.249967	1.165678	1.334298	4.04E-11	3.64E-12	8.35E-16	6.62E-13	1.24E-13	1948	1392	922	1457	1246
UBE2Q2	-1.38077	-1.04711	-1.21099	-1.17298	-1.00083	4.07E-11	1.65E-13	2.03E-13	4.04E-14	1.94E-07	1949	1080	1420	1164	3411
ILF3	1.358969	1.143064	1.11291	1.275458	1.219982	4.09E-11	4.19E-08	6.61E-07	1.20E-12	1.79E-09	1950	2813	3933	1524	2524
HMGB1	1.863658	1.787642	1.684467	1.949546	2.377016	4.09E-11	3.96E-11	2.05E-10	6.51E-13	4.70E-15	1951	1714	2326	1454	916
GMNN	1.041011	1.228088	1.178273	1.25575	0.966492	4.09E-11	1.78E-11	6.84E-13	1.79E-11	8.87E-10	1952	1600	1549	1848	2413
IL13RA2	-1.20327	-0.94418	-1.36096	-1.16285	-1.36683	4.15E-11	1.02E-07	1.76E-09	6.04E-08	2.82E-09	1953	3001	2670	3069	2595
PIGK	-0.62948	-0.6202	-0.4727	-0.61098	-0.64685	4.15E-11	9.84E-09	0.000264	2.77E-06	2.40E-07	1954	2551	5534	3879	3456
PTCH2	-1.48453	-1.25993	-1.38616	-1.26935	-1.14295	4.17E-11	1.20E-12	1.98E-12	1.35E-11	4.83E-06	1955	1269	1663	1809	4091
PGAM5	1.195698	1.413538	1.272133	1.400082	1.168262	4.17E-11	1.13E-13	2.37E-13	5.29E-15	4.05E-12	1956	1045	1438	989	1650
VPS35	1.096741	0.974321	1.235233	1.377486	1.126926	4.19E-11	1.16E-09	2.21E-09	2.39E-14	1.74E-10	1957	2195	2723	1112	2131
NOLC1	1.108257	1.361073	1.278162	1.359188	1.180515	4.22E-11	1.94E-13	2.50E-08	1.43E-13	7.48E-10	1958	1098	3213	1275	2378
IRF6	1.488294	1.414839	1.932639	1.758946	1.644534	4.22E-11	5.86E-07	2.80E-11	7.88E-10	7.92E-11	1959	3347	2033	2360	2017
NCOA6	1.029126	1.20081	1.119973	1.267463	0.667517	4.24E-11	8.24E-10	3.40E-12	2.81E-11	1.25E-05	1961	2146	1736	1903	4347
NFKB2	-0.82869	-0.73082	-1.02951	-1.01551	-1.08473	4.29E-11	2.21E-08	2.31E-10	6.24E-11	1.36E-09	1962	2683	2346	2004	2476
RPL39	2.290177	2.252817	1.916347	2.640498	2.266086	4.30E-11	1.57E-12	4.77E-10	6.84E-16	1.92E-13	1963	1297	2458	825	1291
SLCO2B1	-1.75438	-2.07697	-1.98177	-2.13341	-1.89473	4.32E-11	8.34E-13	1.67E-12	3.23E-16	1.04E-10	1964	1237	1652	775	2059
NOP16	1.304952	1.551927	1.59307	1.484623	1.237988	4.33E-11	7.10E-13	3.76E-14	1.92E-11	5.70E-08	1965	1220	1219	1858	3150
RPL35	2.145235	2.003178	1.943037	2.277303	2.247199	4.37E-11	7.29E-12	5.49E-09	1.74E-15	2.84E-12	1966	1490	2892	890	1610
SMTN	-1.17098	-1.18793	-1.32269	-1.11405	-0.9121	4.47E-11	1.51E-09	8.78E-11	1.99E-08	1.24E-05	1967	2231	2193	2888	4345
F2RL1	1.556621	1.729675	1.983721	1.609916	1.519313	4.48E-11	7.21E-12	2.54E-14	5.08E-13	1.75E-11	1968	1487	1185	1424	1838
TMX3	-0.85867	-0.72068	-1.025	-0.92164	-0.70922	4.50E-11	5.59E-07	3.78E-14	1.28E-08	7.67E-07	1969	3335	1223	2805	3684
RNF130	-1.39272	-1.09389	-1.28166	-1.12015	-1.30781	4.51E-11	3.46E-12	1.84E-14	1.73E-11	3.35E-12	1970	1384	1161	1842	1627
ECE1	-1.65677	-1.68362	-1.62889	-1.7059	-1.81404	4.52E-11	1.37E-10	6.78E-12	2.04E-13	6.99E-16	1971	1872	1822	1318	774
ARSD	1.127796	0.973323	1.158319	1.336424	1.129228	4.53E-11	6.81E-07	2.37E-10	7.03E-10	6.11E-09	1972	3382	2350	2338	2715
NINJ2	-0.95149	-0.64755	-0.96169	-0.82599	-1.09298	4.53E-11	5.80E-05	4.94E-08	1.24E-05	4.33E-15	1973	4527	3371	4269	910
SAMHD1	-1.40171	-1.60655	-1.70403	-1.5364	-1.64357	4.53E-11	4.78E-12	8.63E-18	3.53E-13	2.41E-14	1974	1431	619	1382	1073
LIMK1	-0.80601	-0.68776	-0.80196	-0.68714	-0.77315	4.55E-11	4.26E-09	9.85E-10	2.27E-08	1.97E-10	1975	2409	2577	2909	2151
DMAC1	1.287397	1.475378	1.336499	1.540798	1.523284	4.55E-11	2.20E-11	7.38E-10	7.51E-14	1.28E-12	1976	1627	2530	1214	1511

H3C10	2.20599	1.898586	2.344944	2.040548	2.318555	4.56E-11	1.92E-10	2.66E-15	4.78E-11	4.10E-15	1977	1917	1011	1968	904
UMPS	1.334181	1.387201	1.440585	1.445154	1.355377	4.57E-11	2.56E-11	2.43E-11	1.30E-13	6.10E-11	1978	1656	2009	1263	1971
POMP	1.498873	1.250984	1.475907	1.298842	1.787114	4.57E-11	2.51E-09	4.76E-12	3.14E-09	2.32E-12	1979	2313	1771	2595	1579
BTN3A1	-0.96108	-1.05245	-1.21702	-1.00067	-1.20131	4.57E-11	3.41E-13	2.59E-14	1.77E-13	2.18E-12	1980	1156	1188	1305	1569
DNASE1L3	-2.91047	-2.77023	-3.35545	-3.32637	-3.27215	4.59E-11	1.62E-09	9.14E-13	4.48E-10	1.43E-11	1981	2242	1577	2268	1812
MCCC2	1.217099	1.01096	1.108221	1.021251	1.025691	4.60E-11	4.44E-10	8.02E-13	3.89E-11	1.79E-10	1982	2051	1565	1945	2134
PSMC2	0.863628	0.90887	0.789967	1.017499	0.717556	4.62E-11	7.21E-10	2.88E-12	1.76E-11	1.82E-07	1983	2118	1715	1845	3394
PKN3	-2.04861	-1.86825	-2.68679	-2.44146	-2.68412	4.62E-11	1.12E-10	2.07E-12	2.79E-10	9.94E-13	1984	1844	1670	2195	1483
CHD3	-1.58243	-1.33802	-1.15303	-1.45376	-1.45942	4.62E-11	4.01E-10	2.19E-09	1.49E-11	4.00E-10	1985	2038	2722	1822	2267
FRG2	-1.15886	-0.59482	-0.91978	-0.86828	-0.85831	4.64E-11	0.000161	1.23E-08	3.84E-07	3.07E-09	1986	4836	3065	3437	2607
PRDX5	2.166568	2.446068	2.345944	2.583823	2.280309	4.65E-11	1.87E-15	8.65E-17	3.69E-14	2.01E-21	1987	735	754	1153	168
ZNF304	-0.94561	-0.67138	-0.71986	-0.82543	-0.55082	4.65E-11	1.62E-08	6.95E-10	2.53E-11	1.30E-05	1988	2632	2521	1895	4364
TRIM29	2.196929	1.670785	2.365188	1.682551	1.391693	4.67E-11	1.20E-08	6.68E-13	2.64E-08	5.93E-05	1989	2588	1546	2929	4761
ARPC5L	0.945936	1.021073	0.872671	0.987587	0.648394	4.70E-11	1.57E-14	8.72E-14	5.98E-12	3.35E-06	1990	871	1308	1690	4002
SAMD1	0.95025	1.155281	1.03384	1.135141	0.941326	4.72E-11	3.00E-13	1.76E-13	4.58E-13	5.47E-10	1991	1133	1401	1414	2319
FDPS	1.425414	1.487251	1.411784	1.550073	1.904942	4.76E-11	5.67E-12	6.03E-10	2.60E-13	1.48E-15	1992	1456	2499	1343	836
ATP6V1B2	-1.5813	-1.43483	-1.55444	-1.51611	-1.52684	4.77E-11	1.61E-11	5.85E-11	1.77E-11	3.86E-11	1993	1584	2131	1846	1924
RPL14	2.128308	2.258156	2.044935	2.472131	2.221069	4.81E-11	3.13E-13	3.04E-10	4.93E-17	2.81E-13	1994	1139	2386	663	1327
SDHB	1.229139	1.208144	1.40466	1.351397	0.988023	4.82E-11	5.81E-12	5.62E-13	3.42E-14	6.41E-09	1995	1458	1529	1146	2724
DDRGK1	1.018912	1.0261	0.816986	0.977354	0.683664	4.88E-11	1.16E-09	3.29E-11	2.90E-09	1.78E-06	1996	2194	2060	2575	3855
DSTYK	-0.95548	-0.65595	-0.72231	-0.86461	-1.05454	4.89E-11	6.46E-06	1.02E-07	1.92E-08	6.56E-11	1997	3922	3521	2879	1991
NDUFB4	1.04529	0.966607	1.02255	1.049802	1.156675	4.96E-11	1.14E-09	6.39E-11	4.85E-11	5.74E-12	1998	2192	2141	1973	1687
CD200R1L	-0.67095	-0.60636	-0.77459	-0.7997	-0.74467	5.01E-11	5.07E-09	9.73E-12	4.88E-12	4.42E-11	1999	2443	1870	1674	1940
PLPP4	-0.97031	-0.80945	-0.79289	-0.81516	-1.03407	5.02E-11	7.71E-08	2.54E-09	1.86E-08	5.51E-11	2000	2937	2757	2873	1958
TST	1.532157	1.701545	2.068302	1.663643	1.922557	5.02E-11	5.55E-14	3.33E-18	7.43E-13	1.75E-20	2001	983	554	1466	227
HSPA4	0.953761	1.095359	0.890368	1.074364	0.792056	5.04E-11	2.62E-12	1.36E-10	3.57E-15	5.32E-07	2002	1349	2264	942	3612
RPS2	2.334075	2.160517	1.992102	2.476865	2.322811	5.05E-11	3.30E-12	1.93E-09	3.70E-17	9.27E-14	2003	1377	2691	649	1212
HDGFL3	-1.54878	-1.26416	-1.35599	-1.28984	-1.21764	5.14E-11	1.35E-08	1.42E-08	3.29E-08	1.94E-08	2004	2607	3095	2970	2937
IL2RA	-2.13421	-2.07616	-2.13743	-2.61204	-2.29104	5.15E-11	9.02E-09	4.77E-12	6.17E-11	2.59E-10	2005	2536	1772	2002	2205
AKAP10	-0.77545	-0.61388	-0.83528	-0.81279	-0.52636	5.19E-11	1.54E-07	3.53E-10	1.25E-07	1.52E-05	2006	3090	2413	3204	4401
SOCS2	-1.10907	-1.21385	-1.44481	-1.31553	-1.24877	5.31E-11	4.94E-11	2.54E-10	3.27E-09	3.85E-10	2007	1752	2358	2603	2261
SFXN4	0.892206	0.933992	0.914815	0.939785	0.754371	5.32E-11	2.27E-14	6.49E-12	8.98E-14	5.32E-09	2008	904	1811	1235	2696
DMPK	-1.13658	-0.95274	-1.11792	-1.17115	-0.73421	5.51E-11	2.08E-10	3.36E-09	2.57E-11	0.000313	2009	1933	2810	1897	5241
RPS14	1.897432	1.73265	1.658476	1.893758	1.852489	5.69E-11	4.25E-12	1.11E-09	3.33E-14	3.63E-11	2010	1411	2601	1143	1920
GIMAP2	-0.98121	-0.90398	-0.96775	-0.99211	-0.63878	5.92E-11	4.07E-09	7.87E-10	6.90E-11	8.65E-09	2011	2403	2543	2019	2781
EPN1	0.938568	0.82925	1.158501	0.828042	1.008581	5.96E-11	1.92E-07	1.45E-12	3.23E-07	2.67E-11	2012	3139	1634	3394	1882
B3GAT3	-0.73274	-0.61896	-0.82642	-0.64402	-0.77231	5.99E-11	8.70E-09	6.77E-13	1.18E-08	5.62E-11	2013	2531	1548	2793	1960
PSMB4	1.102746	0.845215	1.009647	0.989316	1.065842	6.08E-11	4.84E-09	8.70E-11	9.14E-10	9.50E-12	2014	2435	2191	2382	1754
DVL3	-0.78227	-0.69016	-0.67798	-0.75451	-0.58963	6.13E-11	1.48E-09	3.72E-13	6.97E-11	9.80E-10	2015	2230	1483	2020	2423
PNN	0.867739	0.922929	0.76583	0.900266	0.794321	6.13E-11	2.11E-10	1.16E-08	3.71E-10	6.31E-09	2016	1936	3049	2237	2719
SDC1	1.478564	1.496296	1.351195	1.404139	1.371317	6.16E-11	2.31E-09	1.96E-11	4.98E-10	2.99E-10	2017	2298	1970	2278	2229
CARMIL2	-0.80752	-0.74343	-0.84696	-0.97982	-1.06473	6.28E-11	9.79E-10	1.01E-10	5.09E-15	8.85E-13	2018	2170	2219	986	1467
ARHGAP8	1.750687	1.890093	1.665003	1.873052	1.529218	6.28E-11	1.27E-11	1.88E-09	3.45E-11	7.14E-09	2019	1555	2685	1925	2742
POFUT1	1.401718	1.330669	0.900533	1.507682	0.7343	6.36E-11	2.46E-11	9.64E-08	3.03E-11	1.30E-06	2020	1646	3504	1906	3792
H3C13	2.164781	1.865297	2.225471	2.087906	2.214547	6.56E-11	7.25E-12	1.55E-11	2.16E-14	1.44E-15	2021	1489	1929	1101	833
H2AC13	1.892592	1.977546	2.083905	2.100447	2.145351	6.61E-11	7.23E-13	4.17E-13	1.37E-15	1.74E-15	2022	1223	1495	867	844
SLC2A14	-1.49288	-1.23754	-1.38403	-1.52962	-1.59813	6.61E-11	6.36E-08	2.94E-09	1.86E-10	5.06E-12	2023	2890	2782	2138	1676
MYBL2	2.086019	2.166453	2.205954	2.348629	2.169466	6.63E-11	1.41E-12	2.16E-13	5.93E-14	2.00E-11	2024	1282	1427	1193	1858
EIF2S3B	1.570992	1.532879	1.274098	1.783426	1.596774	6.66E-11	3.58E-11	7.10E-09	2.21E-15	1.28E-12	2025	1691	2945	910	1510
SGSM2	-1.04102	-0.97966	-1.12721	-0.95675	-0.80366	6.68E-11	8.42E-11	1.08E-10	3.57E-07	0.000176	2026	1806	2226	3419	5088
MRPS23	1.024045	1.236428	1.055176	1.195326	0.768253	6.73E-11	3.86E-13	1.00E-11	2.78E-13	2.90E-06	2027	1169	1876	1353	3965
COX20	1.012242	1.27946	1.152526	1.322615	0.99975	6.98E-11	1.20E-13	1.44E-10	1.22E-12	2.17E-10	2028	1051	2272	1525	2179
FBXL5	-1.05336	-1.25011	-0.89448	-1.20947	-1.17401	7.02E-11	2.10E-11	8.38E-09	1.13E-11	1.47E-11	2029	1622	2981	1780	1814
RASEF	1.843919	1.799156	2.192408	1.947734	1.760309	7.18E-11	4.36E-11	9.61E-17	6.08E-12	3.21E-12	2030	1728	765	1694	1624
LTBP1	-2.00787	-1.93649	-1.87356	-2.03165	-2.32494	7.37E-11	3.37E-11	7.37E-14	1.38E-11	4.04E-18	2031	1683	1287	1810	438
UTRN	-1.72353	-1.06486	-1.83731	-1.22395	-1.52533	7.37E-11	6.91E-07	1.17E-14	3.66E-08	1.10E-11	2032	3388	1129	2988	1778
YPEL3	-1.36585	-1.24364	-1.61666	-1.35732	-1.32693	7.37E-11	1.37E-09	6.28E-15	9.84E-09	4.73E-12	2033	2224	1084	2762	1665
MAPKAPK	0.687511	0.753544	0.79236	0.80676	0.838755	7.38E-11	1.83E-12	7.03E-09	1.07E-09	2.72E-08	2034	1314	2942	2406	2998
DEGS1	-1.24911	-1.43456	-1.50423	-1.42125	-1.47222	7.44E-11	1.56E-10	1.02E-12	7.73E-09	8.31E-14	2035	1893	1594	2715	1204
ZBTB16	-1.15683	-0.58001	-1.37122	-0.94778	-1.02283	7.51E-11	0.000804	1.38E-08	6.14E-06	1.60E-08	2036	5423	3088	4069	2903
BTF3	1.446578	1.418388	1.397792	1.614002	1.555963	7.51E-11	1.55E-11	2.99E-10	5.52E-13	2.25E-09	2037	1580	2384	1435	2561
IARS1	1.185598	1.492648	1.533843	1.526585	1.410806	7.54E-11	1.78E-13	2.61E-10	8.71E-15	6.50E-12	2038	1091	2361	1018	1706
NR4A3	-1.6443	-1.72863	-1.75232	-1.99749	-1.80136	7.60E-11	9.81E-09	6.14E-13	5.86E-11	1.94E-09	2039	2549	1538	1995	2533
ATP5ME	1.659932	1.542308	1.674388	1.495477	1.539426	7.60E-11	6.69E-12	1.50E-11	1.70E-12	4.52E-13	2040	1478	1922	1571	1385
GRIA1	-0.89838	-0.64483	-0.82634	-0.93844	-1.06652	7.72E-11	3.15E-06	2.19E-06	3.81E-08	2.03E-10	2041	3770	4198	2998	2156
PDK4	-1.10237	-1.15552	-0.943	-1.21107	-1.12816	7.72E-11	1.06E-10	7.64E-10	7.89E-12	2.19E-10	2042	1838	2537	1726	2181
PTPRG	-1.25319	-1.25764	-1.12799	-1.17025	-0.96417	7.74E-11	4.34E-11	3.19E-09	1.27E-09	3.18E-09	2043	1727	2802	2429	2615
HIGD1A	1.135147	1.232758	1.510886	1.270016	1.090351	7.75E-11	4.59E-11	1.33E-13	1.35E-13	1.49E-09	2044	1737	1361	1271	2488
LYPLA1	1.250706	1.191174	1.287697	1.411299	0.876921	7.85E-11	1.70E-09	1.47E-09	1.03E-11	8.37E-06	2045	2247	2641	1764	4238
ADNP	0.997741	0.88032	0.84222	1.05776	0.972465	7.88E-11	1.58E-09	6.21E-08	2.94E-10	1.98E-11	2046	2238	3414	2204	1857
KHDRBS1	0.713884	0.724196	0.934478	0.879988	0.716738	7.89E-11	4.51E-12	7.89E-11	1.54E-11	8.53E-10	2047	1422	2169	1831	2403
PTPRB	-1.3656	-1.43192	-1.38501	-1.29415	-1.20205	7.93E-11	4.72E-10	2.25E-13	8.58E-09	4.11E-08	2048	2057	1430	2734	3079
GJA1	-2.33213	-2.28338	-1.8488	-2.58745	-2.40888	8.11E-11	9.86E-15	6.02E-08	3.74E-13	7.50E-12	2049	834	3407	1388	1720

HNRNPA1	1.530245	1.411636	1.319353	1.604538	1.580369	8.11E-11	3.90E-10	2.58E-08	1.73E-13	2.86E-12	2050	2035	3222	1300	1611
STT3B	1.201312	1.060872	1.097711	1.178024	1.22485	8.16E-11	9.93E-09	1.68E-09	1.24E-09	2.27E-13	2051	2553	2657	2425	1310
S100A16	1.911945	1.364611	1.858379	1.45588	1.637216	8.27E-11	1.05E-12	1.67E-10	9.12E-11	5.05E-12	2052	1256	2298	2056	1675
TNS4	2.275329	2.520217	2.58327	2.392653	2.405988	8.31E-11	4.53E-13	1.24E-15	8.28E-14	2.69E-16	2053	1190	955	1225	715
H2BC4	1.680737	1.57153	1.747395	1.595715	1.977976	8.33E-11	1.14E-10	7.99E-15	1.08E-11	1.21E-14	2054	1847	1101	1769	1008
ZMAT5	-0.97757	-1.11806	-0.62721	-0.98301	-0.64491	8.60E-11	2.59E-12	2.09E-06	3.84E-09	6.35E-06	2055	1347	4191	2627	4161
RPS15A	1.919776	1.680892	1.671292	1.973594	1.790954	8.64E-11	5.09E-11	4.92E-09	5.13E-16	2.79E-12	2056	1755	2872	799	1608
U2SURP	0.846255	0.872454	0.784778	0.813106	0.779249	8.64E-11	5.34E-11	6.92E-10	1.25E-09	2.07E-10	2057	1757	2519	2426	2162
HLTF	-0.86999	-0.70499	-0.94217	-0.84669	-1.02888	8.71E-11	5.87E-06	1.35E-08	1.15E-06	2.23E-10	2058	3900	3082	3679	2182
WLS	-1.29699	-1.23638	-1.27038	-1.42783	-0.91232	8.74E-11	3.80E-11	5.46E-12	1.72E-11	2.09E-06	2059	1705	1795	1840	3885
EIF4B	1.242169	1.32663	1.075028	1.370984	1.1881	8.84E-11	1.59E-12	2.72E-09	3.25E-13	7.73E-10	2060	1300	2766	1370	2382
GLB1L	-0.83464	-0.56636	-0.92017	-0.69824	-0.71162	9.04E-11	5.99E-05	1.72E-11	4.62E-06	7.30E-06	2061	4536	1941	4004	4197
PRAP1	2.053529	1.87223	1.798429	2.061849	1.934699	9.06E-11	7.21E-13	2.95E-12	3.80E-12	9.70E-10	2062	1222	1719	1644	2422
RTKN	1.01056	1.171957	1.190674	1.2042	1.156468	9.10E-11	8.75E-11	1.61E-13	3.38E-12	4.37E-16	2063	1812	1389	1633	746
OGFRL1	-1.42937	-0.72456	-1.21826	-0.99225	-1.37445	9.16E-11	0.00016	2.78E-08	1.39E-06	4.08E-10	2064	4834	3236	3712	2274
GOLIM4	1.260105	0.924877	1.16511	1.037229	0.896652	9.18E-11	6.78E-07	4.33E-11	1.47E-06	7.36E-06	2065	3381	2090	3728	4202
INPP5B	-0.99332	-0.69561	-0.85658	-0.75786	-0.964	9.22E-11	7.10E-08	2.42E-09	1.44E-07	8.15E-13	2066	2912	2740	3234	1458
SMARCD2	1.275894	1.293925	1.353505	1.342051	1.350822	9.48E-11	1.09E-09	1.85E-09	4.06E-11	3.11E-11	2067	2188	2682	1949	1898
FBXL6	1.023931	1.161458	1.168065	0.935585	0.947832	9.49E-11	2.23E-12	1.60E-12	7.44E-09	6.36E-13	2068	1329	1645	2709	1433
CEBPA	1.55748	1.675592	1.65149	1.401352	1.361101	9.64E-11	1.21E-10	6.43E-13	1.62E-08	4.54E-08	2069	1852	1542	2849	3107
PFKFB3	-1.4659	-1.35314	-1.2053	-1.46685	-1.58303	9.98E-11	2.60E-09	6.78E-09	1.10E-13	3.12E-11	2070	2321	2932	1250	1899
BTN3A2	-0.88504	-0.88242	-0.65891	-0.85359	-0.80995	1.00E-10	3.59E-11	4.28E-08	4.98E-10	9.16E-09	2071	1693	3341	2277	2795
CAPN6	-0.81563	-0.55987	-0.72173	-0.52329	-0.49995	1.01E-10	5.20E-06	4.74E-10	3.53E-05	3.87E-05	2072	3870	2457	4567	4650
RPL27	1.779606	1.548046	1.46274	1.855611	1.667262	1.04E-10	2.36E-10	1.80E-08	1.50E-14	9.22E-11	2073	1950	3142	1068	2040
NDUFA12	0.857048	0.746032	0.995104	0.803493	0.930283	1.04E-10	1.25E-10	1.09E-12	5.75E-10	4.01E-12	2074	1860	1610	2299	1648
CR2	-0.84433	-0.54406	-0.67956	-0.7833	-0.66964	1.04E-10	6.16E-05	2.97E-05	1.09E-06	5.52E-06	2075	4543	4886	3658	4131
SCAP	0.895052	1.029864	0.830749	1.010042	0.947308	1.05E-10	2.47E-14	1.79E-10	1.31E-13	5.94E-08	2076	912	2309	1264	3160
PRDX4	1.26062	1.319097	1.06613	1.399216	0.798212	1.05E-10	5.43E-10	9.05E-09	1.48E-12	4.12E-06	2077	2081	2992	1555	4058
AKNAD1	-0.85963	-0.56677	-0.90027	-0.60301	-0.6098	1.05E-10	2.63E-05	6.49E-08	0.000182	0.000139	2078	4293	3430	5035	5028
BIK	1.265851	1.661836	1.617088	1.687	1.002996	1.06E-10	4.94E-12	1.77E-12	2.85E-12	3.11E-09	2079	1436	1655	1622	2611
TUBB2B	-0.94567	-0.59669	-0.5465	-0.9669	-0.95408	1.07E-10	2.47E-05	3.54E-06	3.47E-09	1.42E-05	2080	4269	4314	2610	4383
ELL	-1.00043	-0.91671	-0.86068	-1.0429	-1.10394	1.08E-10	1.02E-08	4.92E-09	3.73E-12	7.77E-12	2081	2557	2873	1641	1724
ZMYND19	1.087292	1.154525	1.243385	1.379527	1.027618	1.09E-10	1.69E-07	6.47E-11	6.41E-10	2.01E-06	2082	3110	2144	2325	3879
PIAS3	-1.19003	-1.04873	-1.5196	-0.971	-1.12736	1.10E-10	3.15E-09	3.18E-15	1.56E-06	2.39E-07	2083	2357	1029	3742	3455
HRK	-0.98377	-0.67477	-0.88746	-0.82306	-0.64683	1.10E-10	2.54E-06	4.83E-10	6.09E-08	4.15E-07	2084	3716	2460	3070	3570
RBMX	1.068573	1.102129	0.989129	1.29743	1.01218	1.10E-10	1.61E-10	7.55E-09	5.09E-15	2.69E-11	2085	1897	2952	987	1883
LSM3	1.06988	1.170184	0.867541	1.0804	0.90806	1.10E-10	1.19E-13	8.80E-10	1.29E-11	3.49E-09	2086	1050	2559	1805	2629
LACTB2	1.474264	1.22819	1.667722	1.213107	1.493912	1.11E-10	5.72E-09	1.04E-11	8.90E-10	1.38E-09	2087	2463	1879	2374	2480
COBL	1.438621	1.342919	1.465012	1.38187	1.521632	1.11E-10	8.32E-09	2.07E-11	3.04E-09	6.53E-12	2088	2523	1984	2589	1708
XPC	-0.8041	-0.43121	-0.54143	-0.40849	-0.98223	1.11E-10	0.00017	3.52E-06	0.000691	2.28E-09	2089	4859	4312	5488	2568
KIF5B	1.192565	0.804972	1.344711	1.10561	1.677726	1.12E-10	1.05E-05	1.82E-11	9.07E-07	1.34E-13	2090	4041	1956	3612	1250
ZBTB17	-0.94087	-0.63339	-0.60113	-0.72029	-0.85099	1.13E-10	1.07E-06	3.88E-07	1.49E-08	3.11E-09	2091	3496	3813	2832	2612
KRT12	-0.62008	-0.31442	-0.50841	-0.42068	-0.57465	1.14E-10	0.004653	0.000133	0.001767	5.57E-07	2093	6072	5299	5841	3620
ENO2	-1.38454	-1.29247	-1.60827	-1.38234	-1.86995	1.15E-10	1.25E-08	5.22E-12	7.91E-11	1.89E-16	2094	2595	1789	2036	684
HSPA8	1.530186	1.293404	1.606868	1.447889	1.224791	1.15E-10	4.38E-10	1.80E-11	5.20E-11	1.06E-11	2095	2049	1951	1982	1772
UXT	1.10307	1.12643	0.958148	1.195252	1.017304	1.15E-10	5.16E-14	1.09E-09	7.68E-14	3.22E-13	2096	975	2594	1217	1343
SEC31A	-1.32354	-1.54351	-0.91491	-1.41776	-1.08261	1.16E-10	1.20E-14	1.21E-06	3.10E-11	2.59E-08	2097	846	4084	1912	2986
PRPF19	1.206071	0.924553	1.288623	0.960168	1.049879	1.16E-10	2.06E-07	2.77E-10	1.43E-07	3.67E-09	2098	3157	2372	3233	2636
NINJ1	-1.48777	-1.56452	-1.95248	-1.72143	-1.64098	1.16E-10	5.02E-12	1.88E-17	2.87E-14	1.42E-11	2099	1439	663	1122	1810
ERH	0.976012	1.041218	0.983393	1.029565	1.118247	1.17E-10	2.90E-10	5.25E-09	2.88E-12	8.18E-13	2100	1977	2883	1624	1460
GJB1	1.549079	1.846292	1.119072	1.740474	1.699441	1.20E-10	1.43E-13	9.40E-08	4.49E-13	9.92E-12	2101	1064	3499	1413	1763
ZNF281	-0.76497	-0.69533	-0.86068	-0.7227	-0.80775	1.21E-10	3.84E-08	5.14E-12	2.01E-09	7.91E-11	2102	2792	1786	2518	2016
POLR1A	1.071878	1.077447	0.982329	1.287373	0.880188	1.22E-10	3.67E-11	4.18E-09	2.03E-13	1.03E-08	2103	1698	2848	1317	2822
SBDS	0.981779	0.837482	0.979646	0.851305	1.077867	1.24E-10	1.60E-10	1.03E-10	3.04E-11	4.03E-10	2104	1896	2223	1907	2270
ELOC	0.80647	0.894088	1.034946	0.915031	1.027485	1.25E-10	1.39E-12	4.16E-12	4.37E-11	9.12E-13	2105	1281	1758	1956	1469
EEF1B2	1.868464	1.935692	1.787719	2.083795	1.923895	1.26E-10	3.18E-13	4.16E-11	2.91E-15	2.88E-14	2106	1145	2086	923	1097
MYO1A	2.042466	1.675055	2.406109	1.588163	2.017157	1.26E-10	1.17E-11	3.16E-14	4.29E-10	8.54E-14	2107	1549	1199	2256	1207
FOXP4	1.173936	1.181699	1.126552	1.098462	1.319732	1.29E-10	7.41E-12	1.44E-12	1.14E-11	5.18E-17	2108	1494	1633	1781	594
TTC7B	-1.12095	-0.54578	-0.65585	-0.65978	-1.07653	1.30E-10	0.00044	2.97E-06	3.14E-05	4.64E-10	2109	5206	4278	4535	2296
MERTK	-1.57615	-1.22536	-1.84388	-1.33971	-1.88097	1.30E-10	7.13E-08	1.91E-10	1.90E-08	6.10E-11	2110	2915	2316	2877	1972
MRPS33	1.110362	1.446376	1.212727	1.288282	1.292756	1.30E-10	3.94E-12	1.42E-11	2.14E-13	5.66E-13	2111	1404	1917	1323	1413
BUD31	1.04838	1.104299	1.131114	0.860319	1.073872	1.32E-10	1.01E-10	5.16E-14	4.20E-07	4.41E-08	2112	1834	1251	3453	3100
OSBPL8	-1.07796	-1.16067	-0.9898	-1.13683	-0.97145	1.33E-10	4.70E-11	2.06E-10	6.51E-11	2.24E-09	2113	1744	2329	2009	2559
CNOT11	0.842567	0.986961	1.107132	1.158732	1.042741	1.33E-10	1.92E-11	2.69E-12	2.61E-12	1.27E-11	2114	1609	1707	1608	1797
CSTF3	0.657347	0.867355	0.571323	0.831885	0.567526	1.34E-10	9.21E-12	3.44E-08	2.41E-09	4.88E-06	2115	1518	3299	2546	4095
UCP2	-1.76743	-2.06554	-2.07488	-2.04463	-1.83676	1.34E-10	3.60E-12	9.47E-12	4.07E-11	5.13E-09	2116	1390	1866	1950	2685
ZNF484	-0.60867	-0.64882	-0.82963	-0.77867	-0.83267	1.36E-10	7.55E-09	1.52E-11	1.03E-09	1.16E-14	2117	2505	1924	2401	1002
RPS24	2.105885	1.898502	1.897668	2.165548	2.22543	1.37E-10	1.40E-11	5.69E-10	2.58E-13	6.78E-15	2118	1568	2482	1342	947
GFUS	1.547815	1.705252	2.295019	1.818585	1.83626	1.37E-10	3.14E-14	3.83E-17	3.32E-13	6.26E-13	2119	934	697	1374	1430
ZFAND1	1.054778	1.09758	1.522106	1.230405	1.436165	1.38E-10	3.85E-09	2.87E-13	7.27E-09	5.27E-13	2120	2392	1456	2708	1402
TAX1BP1	0.867083	0.734805	1.158035	1.057132	0.951541	1.38E-10	5.30E-08	1.22E-11	1.25E-10	4.29E-10	2121	2853	1900	2090	2282
RPS16	1.461751	1.514044	1.380324	1.459809	1.396975	1.40E-10	4.92E-10	1.97E-09	1.31E-10	1.92E-10	2122	2063	2700	2096	2150
CD5	-1.59438	-1.5766	-1.7245	-1.73349	-1.74519	1.43E-10	9.26E-12	2.23E-13	1.49E-11	1.62E-13	2123	1519	1429	1825	1274

KLHL20	-0.84255	-0.85483	-0.89975	-1.16026	-1.14051	1.44E-10	4.43E-07	8.55E-09	1.17E-11	1.25E-08	2124	3287	2986	1786	2863
SEMA3F	-1.27006	-1.11262	-1.34831	-1.09746	-0.94215	1.44E-10	2.99E-09	3.38E-11	3.67E-09	2.42E-07	2125	2346	2062	2620	3458
SPECC1L	-0.87622	-0.80153	-0.46124	-0.84203	-1.07335	1.47E-10	3.29E-11	9.11E-05	2.88E-10	1.55E-10	2126	1679	5200	2202	2113
KLF2	-2.45556	-2.61703	-2.95947	-2.8125	-3.65759	1.48E-10	3.78E-11	3.03E-14	2.03E-12	1.24E-19	2127	1702	1196	1586	299
UBE2S	1.164317	1.214346	1.150245	1.09012	0.9179	1.48E-10	1.17E-08	3.18E-09	2.32E-08	4.32E-08	2128	2581	2799	2910	3099
STUB1	0.862963	0.858649	0.826191	0.783764	0.838507	1.49E-10	3.50E-10	1.66E-10	6.10E-10	3.73E-09	2129	2020	2297	2314	2640
MINK1	0.936339	0.808078	0.924491	0.95172	0.726456	1.49E-10	1.94E-07	2.40E-08	8.03E-08	2.61E-06	2130	3142	3195	3127	3942
ACP2	-0.98015	-0.92743	-0.88956	-0.88085	-0.97626	1.50E-10	5.05E-09	3.84E-16	3.97E-08	6.30E-13	2131	2440	865	3003	1432
UQCRB	1.441003	1.374486	1.679602	1.439163	1.299514	1.50E-10	2.96E-13	1.10E-11	1.32E-12	4.20E-11	2132	1131	1888	1536	1934
F13A1	-1.16363	-0.77909	-0.93773	-0.82614	-1.16332	1.50E-10	2.51E-06	5.04E-08	1.80E-06	1.83E-11	2133	3711	3376	3772	1849
CLK4	-0.98468	-0.8043	-0.77897	-0.96392	-0.8714	1.50E-10	3.22E-05	2.99E-06	2.89E-06	3.10E-09	2134	4348	4281	3896	2610
GPR160	1.372287	1.610906	1.380367	1.683934	1.7871	1.50E-10	1.05E-13	7.48E-11	1.67E-13	2.49E-14	2135	1041	2162	1296	1075
TOMM34	1.483017	1.608639	1.300646	1.733305	1.73919	1.51E-10	4.37E-13	4.56E-10	7.59E-16	5.03E-13	2136	1182	2452	829	1395
FGD3	-1.06551	-0.65087	-1.18611	-0.86546	-1.19663	1.52E-10	3.75E-05	3.25E-11	9.74E-07	1.69E-11	2137	4395	2057	3636	1831
CHMP1A	1.075974	1.016778	1.234199	1.147467	1.314701	1.52E-10	2.76E-08	5.60E-13	8.00E-11	1.94E-12	2138	2724	1528	2039	1550
DAZAP1	1.195398	1.104996	1.226037	1.211657	1.253059	1.54E-10	9.09E-11	7.89E-09	6.49E-14	8.49E-12	2139	1816	2964	1201	1742
UHRF1BP1	-0.91524	-0.93619	-0.93568	-1.0058	-1.17852	1.56E-10	3.65E-10	1.52E-11	3.15E-11	1.34E-09	2140	2026	1927	1915	2475
SLC25A3	1.32302	1.144686	1.222157	1.248215	1.279194	1.56E-10	6.23E-09	2.03E-10	7.97E-10	1.15E-11	2141	2479	2322	2361	1786
LMO1	-0.84132	-0.35329	-0.81144	-0.69049	-0.58253	1.57E-10	7.21E-05	4.52E-10	3.20E-08	6.07E-06	2142	4595	2450	2964	4153
SRRD	-0.79868	-0.61552	-0.73185	-0.80877	-0.48786	1.57E-10	5.31E-07	3.19E-09	2.48E-09	2.06E-05	2143	3320	2805	2552	4473
RTP5	-0.82839	-0.66571	-0.86138	-0.74472	-0.86725	1.59E-10	3.71E-07	2.25E-10	4.79E-07	1.33E-07	2144	3262	2341	3486	3333
RGS7BP	-0.60884	-0.51551	-0.78226	-0.64756	-0.45112	1.59E-10	3.57E-05	1.56E-08	1.87E-05	0.000358	2145	4373	3109	4382	5283
EMCN	-1.539	-1.39669	-1.9037	-1.50755	-1.70516	1.60E-10	9.95E-08	4.66E-17	1.13E-08	3.59E-11	2146	2994	709	2781	1919
PPIAL4A	1.48086	1.316881	1.32734	1.433687	1.831675	1.60E-10	4.74E-09	6.07E-09	7.32E-11	1.80E-13	2147	2426	2916	2029	1286
SLC35D2	1.193194	1.331026	1.489708	1.372741	1.023428	1.61E-10	1.38E-10	9.34E-13	3.46E-11	3.96E-08	2148	1873	1584	1927	3070
SFT2D1	1.028674	0.846801	1.089916	0.917922	0.863548	1.62E-10	6.03E-09	7.02E-12	1.97E-10	4.25E-13	2149	2473	1830	2146	1374
CCDC141	-1.07762	-0.95317	-0.96695	-0.99186	-0.89081	1.62E-10	1.60E-06	1.24E-08	2.75E-06	1.79E-06	2150	3596	3068	3874	3859
RPL37	1.867521	1.816563	1.614972	1.963951	1.803766	1.63E-10	1.38E-11	4.15E-09	2.64E-14	1.82E-11	2151	1567	2846	1118	1847
RPS18	2.416027	2.30388	1.994325	2.492705	2.447146	1.65E-10	2.41E-11	7.43E-09	6.48E-15	3.13E-13	2152	1644	2949	998	1339
EBI3	-0.84182	-0.70744	-1.19401	-0.88035	-0.67475	1.65E-10	7.15E-08	5.08E-15	1.08E-09	7.11E-05	2153	2917	1068	2410	4829
HOXD9	-0.77812	-0.55494	-0.72791	-0.71978	-0.50173	1.65E-10	3.87E-05	2.80E-08	3.74E-08	0.000328	2154	4404	3241	2993	5255
WSB1	-1.32899	-1.43698	-1.12154	-1.32656	-1.22576	1.66E-10	2.18E-12	1.73E-09	1.44E-11	2.83E-10	2155	1328	2664	1816	2221
NOP56	1.910482	2.042567	1.67473	1.794298	1.650234	1.66E-10	2.10E-11	2.95E-09	3.16E-11	1.67E-07	2156	1623	2783	1916	3377
ERO1A	1.209029	1.230666	1.301389	1.350686	0.73717	1.68E-10	4.03E-08	3.44E-08	1.16E-10	0.000416	2157	2803	3298	2077	5346
NOTCH2N	-1.13623	-0.86772	-1.30343	-0.979	-1.03618	1.70E-10	8.42E-09	7.26E-17	1.02E-09	2.22E-12	2158	2526	744	2399	1572
TINF2	-0.8636	-0.90273	-0.90942	-0.91467	-0.72683	1.70E-10	3.31E-10	7.42E-14	5.58E-09	1.58E-07	2159	2008	1289	2671	3367
LYSMD3	-0.87817	-0.70017	-0.79285	-0.91769	-0.8638	1.70E-10	3.35E-08	3.61E-13	2.73E-10	5.40E-12	2160	2759	1476	2190	1682
ASS1	1.953812	2.110443	1.991746	2.207879	1.828196	1.71E-10	3.66E-14	1.57E-11	5.13E-18	1.53E-11	2161	945	1931	534	1817
SPATA24	-0.7008	-0.45231	-0.74548	-0.73277	-0.96435	1.73E-10	2.25E-06	3.76E-10	5.96E-10	2.10E-06	2162	3690	2422	2307	3890
SIRPG	-0.92205	-0.90132	-0.94509	-1.13868	-0.70213	1.74E-10	2.27E-10	7.84E-09	7.78E-14	7.54E-06	2163	1946	2963	1219	4211
RANBP1	1.891136	1.669807	1.833458	1.959315	2.189111	1.76E-10	3.44E-10	5.11E-10	1.08E-12	2.50E-16	2164	2018	2466	1516	713
CDK16	0.865269	0.773776	0.802894	0.8061	0.925076	1.76E-10	1.96E-09	7.31E-10	8.31E-09	3.71E-11	2165	2272	2529	2726	1922
MRPS26	1.224385	1.091003	0.900461	1.088261	0.988916	1.78E-10	4.82E-09	9.23E-09	4.91E-09	1.35E-08	2166	2432	2996	2655	2876
ANKRD20A	-1.00067	-0.85199	-1.24702	-1.01372	-0.88851	1.78E-10	4.61E-09	5.73E-12	5.27E-09	1.88E-07	2167	2420	1802	2665	3405
WDR81	-1.08872	-0.82105	-1.23388	-0.86706	-0.71789	1.78E-10	6.68E-08	1.42E-11	7.72E-08	0.000459	2168	2898	1916	3115	5383
CDC34	0.801858	1.102728	0.972497	1.121056	0.814702	1.78E-10	4.97E-14	8.47E-14	1.01E-14	4.97E-07	2169	968	1304	1035	3602
CAPN8	1.951791	1.736501	1.887304	1.793098	1.730879	1.78E-10	1.50E-07	1.97E-09	9.27E-09	6.50E-10	2170	3087	2698	2747	2354
S100A11	1.750881	1.055208	1.760228	1.521189	1.512495	1.79E-10	2.49E-09	9.53E-11	6.75E-10	2.96E-12	2171	2308	2209	2332	1614
NUDT21	1.340483	1.014628	1.393879	1.099241	1.334737	1.80E-10	4.85E-07	1.06E-15	1.50E-07	4.60E-12	2172	3306	941	3242	1662
HCFC1	0.929352	0.792292	0.852096	0.757798	0.680238	1.80E-10	4.82E-09	3.71E-08	6.25E-08	7.06E-07	2173	2431	3314	3076	3665
HIVEP2	-1.06559	-0.78106	-0.68828	-0.88319	-0.7923	1.80E-10	6.89E-09	3.29E-08	1.44E-09	3.75E-10	2174	2492	3285	2466	2250
RALGAPA2	1.469531	1.480337	1.545898	1.680715	1.702137	1.87E-10	1.05E-11	5.00E-12	2.09E-13	6.18E-13	2175	1539	1785	1320	1428
RUNX2	-1.27513	-0.80047	-1.26719	-1.06651	-1.52988	1.93E-10	8.87E-07	2.06E-09	1.99E-07	8.77E-15	2176	3451	2710	3301	972
ARSB	-1.12702	-1.07053	-1.39482	-1.29469	-1.10513	1.94E-10	7.38E-09	8.38E-11	9.38E-10	1.12E-08	2177	2500	2182	2388	2844
SGK2	1.509033	1.688693	1.206152	1.641221	1.400256	1.95E-10	1.55E-11	8.73E-08	3.81E-09	1.45E-09	2178	1581	3479	2626	2487
ELOVL5	-1.22923	-0.77557	-1.26751	-0.75856	-0.96412	1.95E-10	3.65E-06	9.01E-10	0.000115	2.71E-05	2179	3800	2563	4895	4534
SNRPD3	1.43021	1.310845	1.535886	1.474558	1.523677	1.96E-10	5.82E-11	1.85E-11	1.17E-11	1.09E-13	2180	1765	1961	1784	1228
RPS28	1.781367	1.749623	1.482533	2.000651	1.6788	1.98E-10	4.60E-11	3.49E-09	7.08E-15	3.12E-10	2181	1738	2817	1004	2233
RNF24	-1.3892	-1.19715	-1.16929	-1.22107	-1.41078	1.99E-10	1.64E-08	7.49E-10	1.33E-07	3.80E-10	2182	2633	2535	3217	2253
SCUBE1	-1.2828	-1.29748	-1.63425	-1.34384	-1.62294	1.99E-10	1.37E-09	1.76E-16	6.57E-11	2.41E-13	2183	2223	808	2012	1315
RPL37A	2.179523	2.094913	1.950893	2.351834	2.15441	2.01E-10	1.67E-11	1.95E-09	4.61E-15	1.24E-12	2184	1592	2695	970	1508
RHOG	-0.9162	-0.97027	-0.8346	-0.95927	-0.88666	2.02E-10	3.72E-13	6.94E-10	9.38E-12	2.18E-12	2185	1165	2520	1749	1568
RAB3IL1	-1.50704	-1.04321	-1.67478	-1.38305	-1.74678	2.02E-10	1.29E-09	7.39E-12	6.06E-10	9.31E-13	2186	2215	1833	2310	1473
ZNF609	-0.769	-1.06807	-0.98155	-1.07168	-0.82871	2.03E-10	4.71E-13	4.73E-11	7.11E-12	8.13E-10	2187	1192	2101	1713	2394
CCT4	1.471265	1.563566	1.588215	1.597505	1.417872	2.08E-10	3.96E-13	7.01E-13	7.48E-14	3.96E-14	2188	1173	1554	1213	1130
SEMA4C	-1.70992	-1.35888	-1.58384	-1.62355	-1.36534	2.10E-10	1.37E-08	1.28E-13	1.62E-12	5.04E-08	2189	2612	1352	1564	3124
EML6	-0.84925	-0.39284	-0.44293	-0.75135	-0.68789	2.10E-10	0.009642	0.002156	1.20E-06	5.29E-06	2190	6412	6232	3691	4125
CYP3A5	2.061369	2.007101	2.51344	2.365706	2.374954	2.12E-10	2.30E-09	1.18E-15	4.41E-13	5.89E-13	2191	2297	951	1411	1418
TBCA	1.072707	0.970309	1.019059	1.157549	1.104319	2.12E-10	3.62E-11	2.34E-10	5.35E-14	6.67E-13	2192	1694	2348	1182	1439
KCMF1	0.595213	0.516059	0.611167	0.513505	0.567293	2.13E-10	3.98E-08	9.73E-10	6.35E-08	5.70E-08	2193	2802	2573	3077	3151
PSMA2	1.076915	1.081347	1.169886	1.166234	1.346107	2.15E-10	5.65E-10	4.71E-11	4.01E-11	4.05E-15	2194	2089	2100	1948	901
SPRY1	-1.37776	-1.29241	-0.85999	-1.17835	-0.64838	2.16E-10	9.24E-09	4.03E-05	1.90E-07	0.001397	2195	2542	4972	3292	5755
MKNK2	0.942252	0.972765	0.86992	1.085338	0.632252	2.17E-10	3.61E-09	5.69E-10	2.89E-10	5.69E-06	2196	2384	2483	2203	4140

RIN3	-1.17084	-0.9186	-1.02987	-0.8989	-1.01145	2.18E-10	4.61E-11	1.04E-13	6.99E-11	1.16E-10	2197	1739	1319	2022	2074
GZMB	-1.21888	-0.9354	-1.24804	-1.48611	-1.06372	2.18E-10	5.19E-05	3.47E-09	2.85E-10	2.83E-06	2198	4498	2816	2200	3961
SNX32	-0.97548	-0.64925	-1.01737	-0.97955	-0.93842	2.22E-10	1.81E-06	8.33E-06	2.54E-08	9.67E-06	2199	3630	4541	2924	4271
H2AC8	2.174108	1.853285	2.218874	2.153093	2.392302	2.23E-10	1.39E-09	3.19E-10	4.89E-13	6.83E-13	2200	2226	2394	1420	1441
H2AC11	2.009118	1.858062	1.944146	2.016715	2.350885	2.27E-10	2.05E-10	7.05E-11	1.86E-13	8.52E-14	2201	1928	2154	1308	1206
CD276	-1.47993	-1.39642	-1.56373	-1.49253	-1.80666	2.28E-10	3.39E-10	2.60E-11	4.39E-11	6.08E-14	2202	2014	2018	1958	1179
DAP3	1.118169	1.101547	1.074594	1.24075	1.308439	2.30E-10	5.62E-10	1.57E-08	1.28E-16	8.32E-12	2203	2088	3110	716	1734
MCM8	1.25137	1.41432	1.211201	1.176345	1.495377	2.32E-10	5.16E-12	4.15E-13	5.67E-10	7.26E-10	2204	1445	1494	2296	2376
SMG7	0.790543	0.79117	0.832292	0.870988	0.678469	2.33E-10	2.15E-10	5.04E-10	6.13E-11	5.37E-08	2206	1940	2465	2001	3143
SPRED1	-1.65145	-1.79735	-1.51494	-1.70979	-1.44115	2.34E-10	1.52E-14	1.20E-08	3.46E-13	2.65E-07	2207	866	3058	1378	3474
EIF5A	1.492663	1.302105	1.587677	1.377031	1.622538	2.37E-10	4.82E-11	6.93E-11	6.59E-11	1.12E-15	2208	1747	2151	2013	812
STIM1	-0.92205	-0.73885	-0.7893	-0.81365	-0.76701	2.37E-10	4.67E-05	8.66E-07	6.11E-06	2.41E-08	2209	4459	3997	4067	2976
CLDN1	1.71164	2.15099	1.631652	2.31661	2.110626	2.46E-10	3.57E-12	4.53E-11	5.08E-12	4.73E-15	2211	1389	2092	1678	918
CENPP	-1.02623	-0.98863	-0.73662	-1.09556	-1.02141	2.46E-10	3.15E-09	1.73E-09	1.78E-10	9.94E-12	2212	2356	2666	2127	1764
BBLN	0.94503	0.921288	1.039721	1.104024	1.064761	2.47E-10	5.11E-10	2.44E-10	1.34E-09	1.43E-10	2213	2069	2353	2449	2105
H2AC20	1.293818	1.077554	1.354261	1.279572	0.88031	2.48E-10	6.86E-10	2.03E-11	9.01E-12	3.28E-07	2214	2111	1979	1743	3522
LRRC1	1.251861	1.232075	1.274761	1.215342	1.104913	2.48E-10	6.86E-10	8.86E-13	4.93E-10	7.72E-10	2215	2110	1575	2274	2381
ADD1	-0.95436	-0.94872	-0.60604	-0.87732	-0.70454	2.49E-10	1.93E-09	5.39E-05	1.98E-08	3.14E-06	2216	2267	5055	2885	3991
MUC1	2.82735	3.315024	3.807315	3.441162	3.647642	2.50E-10	1.30E-14	3.10E-19	2.09E-13	1.12E-13	2217	850	438	1321	1231
CHCHD7	0.906557	1.202261	1.132052	1.350274	0.837424	2.54E-10	2.28E-11	7.77E-12	8.21E-12	7.45E-07	2218	1632	1839	1730	3674
APLN	-2.12108	-2.04526	-2.35269	-2.17924	-2.01634	2.56E-10	6.46E-09	1.82E-14	6.11E-11	2.66E-08	2219	2484	1158	1999	2993
RPL29	1.943021	1.938032	1.857551	2.130278	2.034633	2.58E-10	3.97E-12	2.13E-10	3.09E-15	6.45E-15	2220	1406	2337	928	942
DUSP5	-1.44129	-1.36257	-1.90375	-1.58277	-1.77859	2.61E-10	3.76E-08	7.14E-14	1.10E-09	3.57E-11	2221	2785	1284	2413	1918
GRPEL2	-0.64765	-0.46893	-0.49777	-0.62798	-0.29744	2.62E-10	0.00016	6.18E-05	3.52E-07	0.005105	2222	4832	5090	3415	6238
CARNMT1	0.759077	0.825379	0.852627	0.955516	0.725847	2.63E-10	1.01E-11	2.87E-13	2.00E-13	1.34E-12	2223	1536	1457	1311	1517
RNF111	-0.74694	-0.78518	-0.77686	-0.86925	-0.86613	2.66E-10	1.70E-10	1.27E-10	6.46E-12	7.81E-13	2224	1902	2255	1702	1453
USP17L15	-1.2844	-0.94781	-1.62752	-1.19008	-1.05888	2.67E-10	2.03E-09	7.67E-17	2.97E-09	1.42E-10	2225	2279	749	2581	2104
EIF3M	1.165269	1.239096	1.200015	1.308334	1.37293	2.68E-10	1.34E-10	1.27E-09	3.39E-12	3.64E-11	2226	1868	2625	1634	1921
DGCR6L	-0.79522	-0.53644	-0.54666	-0.49648	-0.66276	2.69E-10	9.71E-06	1.33E-06	3.38E-05	7.86E-07	2227	4024	4106	4559	3689
SNRPG	1.221801	1.358157	1.279583	1.302642	1.577315	2.70E-10	1.26E-13	2.43E-13	9.92E-13	8.62E-20	2228	1053	1441	1502	287
MYADM	-2.69983	-2.72731	-2.66106	-3.04349	-2.96777	2.76E-10	5.27E-13	4.92E-14	1.51E-13	4.97E-13	2229	1199	1245	1280	1393
PRCP	-1.51435	-1.44737	-1.62232	-1.24451	-1.45133	2.76E-10	2.00E-08	2.88E-11	1.55E-08	2.11E-09	2230	2664	2040	2836	2549
ZNF280D	-0.99442	-0.95652	-1.22089	-0.95861	-0.93095	2.77E-10	1.10E-09	4.02E-13	1.96E-09	3.60E-07	2231	2189	1489	2513	3544
STX12	-1.12421	-1.0665	-0.72544	-0.85667	-0.67567	2.77E-10	5.49E-09	1.05E-05	1.03E-06	0.000197	2232	2457	4605	3650	5118
SMARCA4	0.677401	0.818994	0.893622	0.904303	1.0639	2.79E-10	8.77E-11	1.20E-11	2.13E-10	1.04E-09	2233	1813	1895	2160	2428
PTCH1	-1.79218	-1.39794	-1.33032	-1.52908	-1.06173	2.80E-10	1.48E-08	5.77E-09	1.14E-08	9.55E-07	2234	2624	2902	2786	3724
ENGASE	1.487892	1.777414	1.167339	1.880699	1.46827	2.80E-10	1.38E-12	1.30E-07	2.26E-14	4.56E-12	2235	1280	3571	1107	1660
FAM221B	-0.79299	-0.66306	-0.52208	-0.87783	-0.96574	2.81E-10	1.78E-08	4.32E-06	2.38E-12	8.72E-09	2236	2643	4366	1596	2785
SUPT16H	1.113329	1.164848	1.277819	1.24927	0.99925	2.83E-10	8.46E-11	4.42E-10	1.54E-11	2.53E-08	2237	1807	2447	1830	2982
CCDC184	-0.99403	-0.48193	-0.79582	-0.74867	-0.63613	2.86E-10	0.000346	1.34E-07	1.59E-06	6.21E-06	2238	5119	3576	3746	4159
NLRX1	-0.94745	-0.7436	-0.99482	-0.93743	-0.91801	2.87E-10	9.59E-08	2.13E-07	8.27E-10	2.78E-07	2239	2983	3675	2367	3485
ATP5F1D	1.23094	1.313012	1.404514	1.449576	1.337	2.87E-10	1.36E-10	2.40E-10	1.41E-13	1.08E-10	2240	1870	2352	1274	2062
H2BC17	1.684968	1.674844	1.769326	1.887892	2.085688	2.90E-10	2.92E-11	9.50E-13	4.82E-14	6.06E-13	2241	1666	1589	1172	1425
RASSF5	-1.62618	-1.05751	-1.3235	-1.20501	-1.28856	2.92E-10	7.49E-05	1.08E-06	1.02E-05	3.60E-07	2242	4605	4054	4217	3545
ASAP1	-1.39001	-1.02432	-1.27291	-1.12895	-1.3935	2.92E-10	1.16E-08	1.86E-11	1.48E-08	3.24E-06	2243	2578	1963	2829	3997
LPAR1	-1.49562	-1.30323	-1.0914	-1.46492	-1.23667	2.93E-10	1.23E-06	1.13E-06	2.99E-08	5.82E-07	2244	3536	4071	2951	3633
HIPK3	-0.91585	-0.81513	-0.70592	-0.98387	-0.74909	2.95E-10	9.09E-08	1.18E-07	4.85E-11	1.45E-06	2245	2975	3559	1972	3813
KRTAP4-2	-0.93195	-0.99659	-1.15232	-1.10402	-1.19003	2.96E-10	2.24E-11	1.62E-12	1.69E-10	6.57E-11	2246	1630	1647	2119	1992
MRGBP	0.741035	0.85453	0.772951	0.839717	0.436694	2.96E-10	1.23E-09	9.30E-08	7.62E-08	0.002017	2247	2203	3496	3109	5912
DSC2	1.858162	1.526105	2.049249	1.657647	1.827764	2.98E-10	2.35E-09	8.81E-12	3.86E-12	1.92E-13	2248	2301	1856	1649	1290
CALU	-1.56489	-1.82251	-1.4187	-1.66229	-1.82789	2.98E-10	2.76E-13	2.17E-11	2.78E-14	1.35E-13	2249	1125	1992	1120	1251
CHDH	1.246799	1.150028	1.095439	1.407136	1.314089	2.99E-10	3.83E-10	3.02E-10	2.39E-14	1.51E-13	2250	2033	2385	1113	1267
PDAP1	1.077937	0.860682	0.937813	0.785639	0.813357	2.99E-10	2.21E-07	8.86E-08	5.07E-07	2.11E-06	2251	3169	3483	3499	3893
WBP1L	-1.05206	-0.75489	-0.60792	-0.78299	-0.96061	3.04E-10	4.62E-06	2.78E-06	2.30E-06	8.53E-10	2252	3846	4258	3834	2402
AGPAT3	-0.88471	-0.61642	-0.87598	-0.73383	-0.58549	3.07E-10	7.23E-05	7.75E-11	1.08E-05	2.95E-05	2253	4596	2167	4237	4560
CXCR6	-1.2377	-0.78625	-1.28985	-1.00205	-1.41423	3.08E-10	0.000179	2.37E-08	2.77E-05	1.10E-07	2254	4875	3191	4484	3298
ZNF512	-1.01989	-1.08795	-1.1575	-1.2412	-0.91489	3.08E-10	3.34E-12	1.20E-15	8.41E-13	8.34E-06	2255	1379	953	1479	4236
CLSTN3	-0.96504	-0.95479	-1.15951	-1.16389	-1.15345	3.08E-10	1.81E-09	3.43E-12	1.19E-14	3.45E-11	2256	2256	1737	1052	1913
CSRP1	-1.72387	-1.85951	-1.82947	-2.09117	-1.47466	3.09E-10	7.87E-12	2.60E-11	3.55E-12	4.20E-08	2257	1505	2019	1637	3085
KPNB1	1.065027	0.91524	1.022038	1.076932	1.134619	3.12E-10	8.69E-11	4.62E-09	1.65E-13	2.03E-14	2258	1810	2861	1295	1057
NUDT15	1.276849	1.202721	1.175329	1.123592	1.141231	3.12E-10	5.62E-10	2.24E-09	1.94E-09	2.95E-08	2259	2087	2727	2510	3022
HYAL2	-1.2489	-1.61212	-1.54722	-1.26918	-1.33278	3.13E-10	7.37E-13	1.27E-09	3.31E-09	1.44E-08	2260	1226	2624	2605	2887
ZNF625	-0.96365	-0.67843	-0.90432	-0.854	-0.71142	3.14E-10	5.17E-05	8.70E-07	1.32E-06	2.50E-05	2261	4496	4000	3703	4518
SMIM23	-0.69084	-0.66548	-0.786	-0.86583	-0.62752	3.18E-10	4.27E-07	6.48E-08	2.19E-09	2.18E-06	2262	3280	3429	2532	3902
EFNA1	1.334949	1.319555	1.542158	1.63303	1.450734	3.25E-10	2.16E-10	8.44E-09	1.80E-11	1.05E-09	2263	1942	2982	1849	2430
H2AC12	2.073702	1.836028	2.25381	2.028488	2.168479	3.27E-10	5.67E-10	2.96E-13	1.53E-12	6.69E-14	2264	2091	1460	1559	1186
IGHM	-4.39084	-3.91404	-5.06313	-4.44627	-4.53108	3.29E-10	2.97E-10	3.04E-12	1.18E-11	3.49E-10	2265	1988	1725	1787	2241
OSER1	1.390933	1.50487	1.398009	1.714209	1.353826	3.30E-10	8.03E-12	3.54E-10	3.78E-13	2.27E-09	2266	1508	2414	1392	2564
EIF2A	0.90127	0.765168	0.717393	0.727986	0.512906	3.33E-10	8.97E-08	3.78E-09	1.73E-08	9.88E-06	2267	2973	2833	2862	4274
MACC1	1.82562	1.454774	0.979022	1.556354	1.37983	3.36E-10	1.41E-10	1.84E-08	3.20E-10	7.72E-09	2269	1875	3146	2215	2761
ARRDC2	-1.10907	-1.02621	-1.02946	-0.98905	-0.98828	3.41E-10	1.02E-10	2.07E-12	4.54E-09	1.28E-09	2270	1835	1671	2648	2461
SLC29A2	1.139657	1.255565	0.960402	1.023586	0.912335	3.41E-10	1.20E-10	7.01E-08	8.21E-08	2.02E-07	2271	1851	3444	3130	3416
HBB	-1.50063	-0.89918	-1.52376	-1.02661	-1.76478	3.42E-10	0.000104	6.17E-09	1.94E-05	5.71E-11	2272	4708	2922	4394	1963

ZNRF2	1.047698	1.13597	1.304249	1.14049	1.260123	3.43E-10	2.77E-09	3.14E-13	3.11E-10	3.60E-10	2273	2334	1464	2211	2245
DAND5	-0.73403	-0.46482	-0.73297	-0.58223	-0.42931	3.43E-10	1.18E-06	2.83E-09	2.08E-06	0.000446	2274	3522	2774	3812	5369
MSRB3	-1.87862	-1.53374	-1.64832	-1.68797	-1.68333	3.45E-10	8.29E-08	2.89E-10	9.67E-09	3.19E-12	2275	2954	2378	2757	1622
ZNF37A	-0.73568	-0.52166	-0.79136	-0.70774	-0.80133	3.47E-10	1.37E-07	3.51E-12	6.71E-10	2.78E-12	2276	3063	1741	2331	1605
EIF3G	0.995998	0.940265	0.874246	1.117032	0.846625	3.47E-10	4.56E-09	2.92E-07	2.36E-14	2.96E-07	2277	2415	3742	1110	3499
DVL1	0.862096	0.991781	1.0619	0.925446	0.862678	3.48E-10	1.03E-11	2.32E-11	6.03E-11	8.22E-10	2278	1538	2000	1998	2398
HCFC2	-1.21044	-0.98544	-1.2542	-1.05176	-1.21149	3.49E-10	8.75E-10	1.07E-09	2.53E-12	9.71E-11	2280	2154	2588	1603	2046
NECAP2	-1.13621	-0.99147	-0.72602	-0.9192	-0.7987	3.53E-10	7.81E-09	2.94E-07	1.64E-08	1.10E-08	2281	2514	3743	2852	2837
VAV3	1.528922	1.431911	1.469299	1.615168	1.693653	3.56E-10	2.42E-11	2.44E-12	1.71E-11	9.44E-14	2282	1645	1685	1838	1216
NOC2L	0.886844	1.011108	0.854448	1.036881	0.985357	3.56E-10	1.41E-10	4.99E-08	5.01E-14	2.84E-09	2283	1878	3374	1175	2596
IFI27L2	-1.13921	-1.13644	-1.15114	-0.95373	-1.02527	3.61E-10	1.56E-07	6.45E-10	3.17E-08	8.67E-08	2284	3092	2507	2959	3247
NAAA	-0.97325	-0.94064	-1.06001	-1.15666	-1.16134	3.63E-10	5.82E-09	1.67E-10	3.32E-11	6.17E-11	2285	2467	2299	1922	1976
RPL38	2.030018	1.796679	1.656319	1.9476	1.794747	3.63E-10	5.15E-10	7.93E-08	2.58E-13	3.33E-11	2286	2070	3460	1341	1908
JMY	-0.80461	-0.47741	-0.60321	-0.42451	-0.81192	3.64E-10	7.83E-05	7.51E-06	0.000503	2.09E-09	2287	4621	4508	5379	2546
HDAC5	-1.14835	-1.04061	-1.23962	-1.16561	-1.49258	3.68E-10	1.09E-10	3.63E-12	7.59E-10	1.64E-17	2288	1840	1745	2355	527
DNAAF5	1.382774	1.367002	1.274653	1.28778	0.86034	3.69E-10	1.04E-07	9.67E-09	5.51E-07	2.91E-05	2289	3006	3006	3517	4548
PRPS2	1.198723	1.162128	0.766667	1.269931	0.885964	3.70E-10	6.51E-10	8.26E-07	3.98E-10	4.59E-07	2290	2103	3989	2246	3589
CHRDL2	-1.09818	-0.67078	-0.54791	-0.91382	-1.10555	3.72E-10	0.000273	0.000279	7.22E-06	1.79E-06	2291	5021	5550	4116	3857
RBP1	-1.39192	-0.74446	-1.06735	-0.88392	-0.72159	3.77E-10	0.00299	4.36E-06	0.00014	0.005432	2292	5894	4371	4959	6267
SNRPE	1.267695	1.373821	1.272476	1.32168	1.220572	3.81E-10	2.17E-12	3.36E-10	5.96E-15	1.85E-09	2293	1327	2408	994	2526
GAST	-0.93694	-0.64545	-0.87731	-0.7515	-0.98756	3.81E-10	3.86E-05	1.54E-07	1.81E-05	7.12E-09	2294	4403	3605	4373	2740
DMXL1	-0.78674	-0.48555	-0.54112	-0.51378	-0.98259	3.91E-10	0.000943	1.04E-06	0.000528	5.03E-11	2295	5492	4048	5404	1950
PHC3	-0.66049	-0.51479	-0.59007	-0.57941	-0.48582	3.96E-10	9.19E-07	5.64E-08	9.99E-07	8.43E-05	2296	3459	3395	3641	4871
GRTP1	1.653697	1.520706	1.505304	1.498114	1.179885	3.96E-10	5.67E-12	1.29E-13	1.74E-11	2.42E-06	2297	1455	1353	1843	3932
BECN1	0.716403	0.652235	0.699448	0.61049	0.426302	3.98E-10	8.69E-08	3.19E-09	3.81E-08	0.001775	2298	2965	2804	2997	5863
ITGB5	-1.27234	-1.31619	-0.98656	-1.44267	-1.76047	4.00E-10	2.11E-11	1.78E-08	1.46E-09	6.80E-10	2299	1624	3140	2469	2363
ALG1L2	-1.03591	-0.5682	-1.06305	-0.91144	-1.1918	4.02E-10	0.000211	1.84E-08	2.54E-07	1.22E-08	2300	4923	3149	3344	2859
STK17B	-1.55748	-1.39612	-1.75309	-1.64274	-1.89181	4.02E-10	2.97E-08	1.08E-11	1.08E-09	1.66E-09	2302	2734	1882	2409	2506
SMG5	0.71665	0.727161	0.808708	0.826224	0.790639	4.04E-10	2.05E-08	2.68E-09	1.73E-10	8.84E-09	2303	2670	2764	2122	2788
LDHA	1.638791	1.271233	1.974427	1.455763	1.414479	4.08E-10	1.35E-07	3.27E-12	3.62E-10	7.06E-09	2304	3060	1733	2231	2739
HDAC2	0.988265	0.988893	1.064363	1.122279	1.017016	4.09E-10	1.23E-10	1.56E-10	2.11E-12	5.01E-12	2305	1856	2288	1589	1674
RPLP2	1.838474	1.722488	1.558147	1.936005	1.833419	4.11E-10	1.90E-10	6.81E-09	2.78E-13	4.28E-12	2306	1916	2933	1354	1655
TMEM164	1.019162	0.957552	0.779829	0.911233	0.790965	4.12E-10	2.46E-10	3.55E-06	1.76E-07	6.53E-07	2307	1957	4315	3273	3658
CTNNB1	1.371388	1.107298	1.60233	1.42915	1.184084	4.13E-10	2.24E-10	2.70E-12	8.76E-12	8.13E-09	2308	1944	1711	1739	2769
CDCP1	1.159451	1.152173	1.588278	1.280467	1.32768	4.14E-10	3.20E-11	1.42E-14	1.49E-11	9.05E-17	2309	1676	1141	1821	631
PPARG	1.544878	1.662293	1.553811	1.43991	1.428868	4.14E-10	2.95E-13	1.84E-11	3.18E-09	3.87E-10	2310	1128	1960	2599	2262
TRPA1	-2.1779	-2.11538	-2.58484	-2.20492	-2.81097	4.15E-10	7.63E-08	5.68E-12	1.04E-08	3.25E-11	2311	2935	1800	2769	1903
STC1	-2.01756	-1.67347	-2.30038	-2.13408	-2.22392	4.16E-10	1.78E-06	2.80E-09	5.00E-07	1.08E-07	2312	3627	2772	3493	3292
HSD17B10	1.410259	1.576737	1.231785	1.572799	1.457643	4.19E-10	2.04E-11	5.89E-09	1.54E-12	3.62E-13	2313	1615	2908	1561	1358
EPG5	-0.93714	-0.91829	-0.79771	-1.05339	-0.85631	4.26E-10	3.21E-10	1.41E-08	1.40E-10	4.28E-09	2314	2002	3094	2105	2667
KLC1	-1.07107	-0.83216	-0.71782	-0.82303	-0.68687	4.27E-10	1.43E-06	1.90E-06	2.79E-06	2.00E-05	2315	3571	4177	3883	4466
TRAF4	1.019074	0.953371	1.288574	1.151724	1.37119	4.31E-10	2.93E-09	4.11E-17	2.45E-11	5.62E-15	2316	2341	704	1892	931
C10orf99	1.901048	2.552615	1.843065	2.534552	2.12269	4.35E-10	4.95E-15	2.47E-08	8.19E-13	2.91E-10	2317	784	3208	1475	2223
ZNF618	-1.16879	-1.01945	-1.0916	-1.21273	-1.08056	4.39E-10	2.39E-11	7.59E-13	5.73E-13	7.77E-12	2318	1641	1561	1438	1725
GRB10	-1.39531	-1.28984	-1.39331	-1.66451	-1.22048	4.39E-10	7.77E-11	3.35E-10	6.97E-13	9.62E-09	2319	1793	2406	1461	2807
BUD23	1.458345	1.412818	1.401095	1.586827	1.543945	4.41E-10	2.71E-09	6.65E-10	4.78E-13	1.98E-09	2320	2329	2516	1418	2536
TSPAN3	1.697234	1.133003	1.922435	1.295981	1.873795	4.52E-10	8.58E-06	6.59E-11	1.85E-08	5.38E-11	2321	3994	2147	2871	1955
AIMP2	1.189824	1.334392	1.517082	1.138742	1.136947	4.52E-10	4.85E-10	7.37E-15	2.72E-09	6.88E-10	2322	2061	1097	2563	2366
SP110	-1.02104	-1.05044	-0.923	-0.80805	-1.139	4.52E-10	5.06E-09	4.10E-07	5.84E-10	1.34E-08	2323	2441	3825	2304	2874
LHFPL2	-1.32778	-1.66666	-1.43817	-1.44032	-1.33302	4.54E-10	2.26E-12	3.34E-11	1.41E-11	1.82E-07	2324	1331	2061	1812	3395
MSRA	-0.92602	-0.7	-0.83133	-0.69607	-0.68764	4.54E-10	6.54E-08	2.75E-08	1.92E-06	1.28E-06	2325	2895	3233	3796	3789
ORMDL2	0.973543	0.756773	0.756135	0.735132	0.438339	4.55E-10	1.76E-08	1.92E-10	2.20E-07	0.001042	2326	2639	2317	3314	5647
GOLGA6A	-1.14177	-0.7284	-1.20817	-0.9113	-1.13196	4.57E-10	1.10E-06	4.67E-12	5.21E-07	3.20E-14	2327	3507	1770	3501	1108
ZNF428	-0.89181	-0.78534	-0.66627	-0.67527	-0.71913	4.59E-10	9.03E-10	2.03E-07	1.89E-08	1.66E-05	2328	2161	3660	2875	4421
JAGN1	0.781545	0.973939	0.8656	0.849506	0.670783	4.64E-10	1.25E-08	3.79E-08	1.28E-08	2.13E-07	2329	2596	3318	2804	3427
STK24	1.338348	1.215965	1.464439	1.069435	1.709539	4.65E-10	2.62E-08	3.95E-11	3.34E-08	5.78E-13	2330	2713	2078	2976	1415
NAT10	0.834951	1.039711	1.122693	0.881606	0.922689	4.65E-10	1.57E-14	6.33E-16	8.96E-12	3.03E-15	2331	869	890	1742	873
C21orf91	-0.73898	-0.59083	-0.67636	-0.73194	-0.88091	4.67E-10	1.26E-07	1.43E-13	1.69E-09	3.73E-13	2332	3047	1372	2495	1363
PHF1	-1.13835	-0.85363	-1.11817	-1.18134	-1.04947	4.71E-10	6.98E-06	1.12E-07	7.84E-07	7.18E-08	2333	3936	3542	3588	3209
TWIST1	-1.34509	-1.1091	-1.30797	-1.34905	-1.42591	4.74E-10	5.13E-07	9.77E-12	8.14E-09	3.26E-09	2334	3317	1871	2720	2617
OR2T5	-0.89987	-0.55151	-0.81556	-0.692	-0.99682	4.77E-10	1.71E-05	2.93E-09	1.47E-06	2.87E-09	2335	4163	2780	3726	2597
RPL35A	1.782785	1.857972	1.490624	1.899391	1.841878	4.79E-10	6.27E-11	2.43E-08	1.75E-13	4.45E-11	2336	1770	3201	1303	1942
FAM47B	-0.72168	-0.48509	-0.70516	-0.69112	-0.62128	4.80E-10	1.89E-06	5.18E-08	1.81E-09	2.17E-08	2337	3643	3384	2500	2956
HOXB6	1.808547	2.14582	2.460622	2.271923	2.2586	4.82E-10	1.21E-14	8.88E-16	6.11E-16	7.89E-16	2338	847	926	819	789
SLC6A2	-0.67802	-0.41498	-0.59343	-0.65896	-0.65027	4.82E-10	0.00048	1.23E-05	6.53E-08	1.73E-06	2339	5235	4655	3083	3851
RPSA	1.722792	1.940743	1.647763	1.976094	2.05792	4.84E-10	6.68E-12	5.55E-09	2.90E-14	2.59E-13	2340	1477	2898	1125	1319
KLF12	-1.04374	-0.73993	-0.99644	-1.11453	-0.92155	4.87E-10	0.000184	9.26E-07	2.89E-06	1.19E-08	2341	4881	4018	3894	2852
CXCL9	-1.88751	-2.85839	-2.1871	-3.10138	-2.29843	4.93E-10	9.42E-12	1.88E-10	3.61E-13	5.40E-12	2342	1523	2315	1385	1683
MEGF6	-1.0975	-0.52054	-0.64714	-0.57699	-0.58113	4.94E-10	0.000504	1.00E-05	0.000323	0.000255	2343	5257	4592	5240	5192
FBLIM1	-1.51161	-1.5458	-1.31097	-1.48086	-0.75526	5.02E-10	7.64E-11	2.88E-09	1.72E-11	0.000579	2344	1791	2778	1841	5447
PIM1	-1.31484	-1.30522	-1.43919	-1.58734	-1.72955	5.04E-10	1.27E-10	8.42E-10	4.51E-15	6.29E-12	2345	1862	2552	967	1698
BUB3	0.918849	0.855587	0.797587	0.714684	0.850208	5.05E-10	1.48E-08	2.45E-09	1.40E-07	7.82E-11	2346	2625	2748	3229	2015
RBPJ	-0.90253	-0.98053	-0.78969	-1.09729	-0.76718	5.05E-10	9.56E-08	7.63E-07	1.09E-09	2.79E-08	2347	2982	3964	2412	3007

CYTH1	-0.94604	-0.96478	-1.06395	-0.90527	-1.06442	5.10E-10	4.90E-11	1.16E-10	7.84E-08	1.57E-08	2348	1749	2238	3118	2899
NFATC4	-1.72644	-1.56425	-1.4603	-1.59942	-1.67304	5.12E-10	1.05E-09	2.78E-09	1.78E-10	8.05E-13	2349	2179	2771	2126	1456
NAA20	0.93703	0.970134	0.65267	1.072135	1.031973	5.15E-10	5.28E-10	9.17E-07	4.68E-12	3.36E-10	2350	2073	4013	1670	2237
PCNA	1.698618	1.682373	1.726104	1.703001	1.701279	5.15E-10	3.39E-11	1.06E-12	6.54E-13	7.13E-11	2351	1684	1605	1456	2009
ZKSCAN1	0.896923	0.967946	1.14383	1.169573	0.974698	5.15E-10	9.04E-09	7.77E-09	1.00E-09	2.81E-09	2352	2537	2960	2392	2593
PAQR4	1.118039	1.138712	1.047439	1.311367	1.341559	5.15E-10	5.35E-09	1.71E-08	1.69E-09	1.69E-09	2353	2451	3133	2497	2509
KLC4	0.921448	0.94994	0.740119	0.984793	0.853539	5.16E-10	3.93E-09	1.03E-08	8.63E-10	1.99E-10	2354	2395	3019	2368	2153
CDK1	1.236243	1.352946	1.564777	1.242822	1.352204	5.16E-10	3.07E-10	5.89E-14	1.82E-09	3.83E-10	2355	1995	1261	2503	2258
STRAP	0.968467	0.893281	0.88458	1.031054	0.914302	5.34E-10	2.67E-08	1.90E-07	7.59E-10	1.65E-08	2357	2719	3651	2356	2910
SRSF9	0.843803	0.849275	0.926067	1.046597	0.944655	5.38E-10	1.71E-11	5.58E-10	1.05E-12	2.74E-10	2358	1595	2481	1512	2213
LDLR	1.158334	1.132919	1.453091	1.398714	1.23647	5.39E-10	2.54E-08	8.36E-11	1.74E-09	2.78E-12	2359	2706	2180	2498	1606
EEF1D	1.186925	1.473835	1.254673	1.510502	1.111863	5.41E-10	3.22E-13	2.43E-08	1.67E-14	2.72E-10	2360	1146	3203	1077	2212
VTA1	0.708165	0.697313	0.859405	0.547536	0.431619	5.44E-10	1.78E-08	6.89E-12	5.51E-06	0.001137	2361	2641	1825	4048	5683
SPATA2L	-0.84278	-0.54211	-0.92169	-0.74382	-0.53517	5.48E-10	2.66E-05	6.64E-10	5.50E-06	0.000242	2362	4295	2515	4047	5176
PSMC4	0.931497	0.86617	1.253156	0.852537	1.011678	5.49E-10	5.62E-07	2.72E-11	1.64E-07	1.66E-10	2363	3337	2028	3260	2122
RPS19	1.844629	1.930615	1.601973	1.951906	1.857397	5.50E-10	4.82E-11	9.93E-09	9.44E-14	6.01E-13	2364	1746	3011	1239	1420
CHERP	0.807965	0.782211	0.685195	0.838335	0.6513	5.50E-10	3.34E-09	3.81E-07	6.37E-12	5.64E-06	2365	2371	3808	1701	4138
RPL24	1.811242	1.682186	1.522201	1.728199	1.79123	5.50E-10	3.82E-09	1.40E-08	2.71E-10	2.98E-10	2366	2391	3092	2189	2227
SNRNP200	1.137036	1.039493	1.312093	1.159959	1.0717	5.53E-10	4.58E-09	1.87E-11	2.77E-10	6.81E-11	2367	2418	1965	2194	1999
MAIP1	0.768803	0.889947	0.864422	0.914501	0.696561	5.57E-10	3.66E-10	1.68E-09	5.96E-08	4.67E-08	2368	2028	2659	3066	3114
GPAT2	-0.93036	-0.41395	-0.98334	-0.72212	-0.98725	5.61E-10	0.00183	1.84E-08	3.56E-05	1.78E-07	2369	5724	3148	4573	3389
NACA	1.685606	1.397478	1.466997	1.548537	1.61933	5.61E-10	1.20E-09	3.22E-08	7.44E-12	2.05E-12	2370	2197	3275	1717	1560
RNF11	-0.97926	-0.8926	-0.67361	-0.80312	-0.99421	5.64E-10	3.09E-10	2.50E-06	3.25E-08	1.17E-09	2371	1996	4238	2968	2449
ADD3	1.272663	1.36765	1.518276	1.418641	1.882771	5.64E-10	2.64E-10	8.53E-14	5.79E-10	3.29E-14	2372	1966	1306	2301	1109
UBA7	-0.97996	-1.26823	-1.2531	-1.23382	-1.0993	5.67E-10	1.21E-10	1.95E-09	5.58E-11	1.23E-06	2374	1853	2694	1989	3780
NR2F2	-1.3609	-1.39955	-0.95995	-1.28427	-1.05128	5.68E-10	2.05E-10	2.71E-07	5.43E-08	5.23E-08	2375	1929	3725	3048	3137
SLC22A4	-0.97202	-0.67133	-0.9721	-0.75197	-0.90605	5.68E-10	1.99E-06	2.87E-11	1.09E-07	2.40E-10	2376	3655	2039	3184	2191
TFAP4	1.060977	0.977431	0.92676	0.997535	0.994464	5.77E-10	1.68E-09	1.25E-08	2.39E-11	1.56E-09	2377	2244	3071	1888	2496
NRARP	1.24909	1.347151	1.650946	1.329934	1.509001	5.78E-10	8.32E-11	1.12E-15	5.96E-10	3.03E-16	2378	1803	947	2308	725
NHP2	1.470215	1.76716	1.401944	1.749775	1.871305	5.78E-10	3.31E-13	1.15E-09	9.66E-18	1.49E-14	2379	1150	2608	576	1023
SOGA1	-0.87204	-0.83604	-0.93329	-0.6972	-1.21866	5.78E-10	7.38E-10	8.29E-13	1.07E-07	2.87E-14	2380	2125	1566	3180	1096
EDF1	1.037726	0.78768	1.226892	0.89125	1.017442	5.86E-10	3.19E-09	1.63E-14	4.18E-08	5.65E-11	2381	2362	1146	3010	1962
KRTCAP2	0.997123	0.892033	0.629414	0.870961	0.655988	5.93E-10	5.74E-08	7.32E-06	7.40E-08	3.75E-05	2382	2871	4502	3103	4633
CCT8	0.992584	0.845156	1.072533	0.989192	0.930758	5.93E-10	7.77E-09	6.47E-11	1.67E-10	4.39E-10	2383	2512	2143	2116	2286
ANOS1	-1.68733	-1.59593	-1.41349	-1.35718	-1.82846	5.95E-10	1.98E-09	4.47E-08	3.16E-09	1.58E-10	2384	2275	3351	2596	2116
SNRPC	1.053048	0.951002	0.941246	0.933361	0.9767	5.95E-10	9.53E-10	3.66E-08	3.65E-11	2.81E-09	2385	2167	3309	1941	2594
CDC25B	1.505156	1.469614	1.364919	1.669226	1.086225	5.99E-10	3.49E-08	3.05E-08	1.18E-10	1.09E-05	2386	2772	3261	2082	4299
SMAD7	-1.03189	-1.21375	-1.06309	-1.36166	-1.14582	6.00E-10	8.42E-11	2.45E-09	1.33E-12	3.53E-08	2387	1805	2746	1537	3049
IDH3G	1.006769	1.062339	0.953348	1.256085	0.847224	6.06E-10	1.49E-11	5.85E-12	1.12E-13	8.58E-12	2388	1574	1804	1252	1743
LRRC37A2	-0.58906	-0.34405	-0.60228	-0.6442	-0.58332	6.08E-10	0.000291	3.55E-06	9.26E-08	6.94E-05	2389	5049	4316	3153	4822
ST3GAL1	-1.77602	-1.475	-1.77596	-1.49011	-2.22916	6.11E-10	7.39E-09	2.25E-15	8.21E-09	1.19E-12	2390	2501	997	2723	1507
NTRK3	-0.86785	-0.51589	-0.59432	-0.68353	-0.64079	6.12E-10	9.51E-05	1.27E-06	1.43E-06	2.87E-06	2391	4685	4098	3719	3963
EMP2	1.240574	1.186466	1.358873	1.547297	1.049559	6.14E-10	5.55E-10	9.32E-10	5.48E-13	2.48E-06	2392	2086	2567	1433	3937
LACTB	-0.84103	-0.80218	-0.8647	-0.8207	-0.85025	6.14E-10	3.18E-07	8.72E-07	6.73E-08	3.49E-07	2393	3231	4002	3087	3533
H3C15	2.396226	2.115729	2.370431	2.404932	2.6198	6.17E-10	7.61E-10	4.16E-10	1.01E-13	4.26E-13	2394	2129	2437	1246	1377
CHST12	-0.84681	-0.72484	-1.05069	-0.75863	-1.28912	6.18E-10	6.38E-08	2.05E-13	1.83E-08	5.06E-14	2395	2891	1422	2870	1154
RPA3	1.403625	1.405787	1.297329	1.421935	1.304407	6.19E-10	2.46E-09	1.44E-10	1.27E-09	1.13E-09	2396	2306	2273	2431	2446
TMEM258	0.994531	0.854297	1.108096	1.083672	0.772057	6.24E-10	3.15E-08	7.84E-09	9.26E-11	3.69E-07	2397	2744	2962	2058	3553
TINAGL1	1.580656	1.792511	2.026178	1.954875	1.751469	6.25E-10	2.37E-09	8.00E-16	1.66E-11	2.01E-15	2398	2302	917	1837	849
NDUFS7	0.881674	0.955338	0.814397	0.862587	0.651699	6.27E-10	4.15E-11	1.84E-08	7.58E-09	1.94E-05	2399	1721	3150	2713	4457
SH3PXD2A	-1.20771	-1.12791	-1.03805	-1.06905	-1.12873	6.27E-10	1.88E-09	1.10E-09	6.60E-09	1.98E-10	2400	2265	2596	2696	2152
CLCN3	1.123565	0.929844	1.577151	1.08556	1.044554	6.37E-10	2.93E-07	2.32E-13	1.18E-08	1.28E-07	2401	3210	1436	2794	3326
PNRC1	-1.26592	-1.45505	-1.24336	-1.38586	-1.26826	6.42E-10	4.53E-12	2.68E-12	1.64E-10	2.44E-09	2402	1424	1705	2112	2579
PAPOLA	0.789019	0.913041	0.90002	0.845308	0.775218	6.46E-10	6.27E-11	7.49E-10	3.73E-09	6.92E-10	2403	1771	2536	2624	2367
PCBP2	1.321601	0.927329	1.120759	1.075787	0.988937	6.64E-10	1.17E-07	6.54E-08	2.78E-08	1.33E-08	2404	3034	3434	2940	2873
SNX2	-0.64463	-0.69905	-0.43025	-0.64182	-0.50093	6.67E-10	3.38E-09	0.000148	2.35E-06	6.36E-06	2405	2373	5325	3837	4162
FUT2	1.554026	1.685932	1.591745	2.143067	1.61013	6.67E-10	2.02E-09	1.61E-11	1.18E-13	2.18E-11	2406	2278	1932	1256	1867
SFPQ	0.853992	0.702846	0.780976	0.830137	0.864319	6.68E-10	1.62E-07	7.93E-07	1.99E-08	9.32E-09	2407	3096	3978	2887	2798
B4GALT5	1.292448	1.136606	1.412997	1.405377	1.204373	6.75E-10	7.30E-08	8.92E-11	7.86E-11	2.07E-10	2408	2923	2198	2035	2163
H3C2	2.100885	1.758459	2.363081	1.915253	2.58864	6.75E-10	5.67E-10	1.57E-12	7.93E-11	7.55E-15	2409	2092	1643	2038	959
MCTS1	0.997319	1.023345	1.073902	1.243069	0.90568	6.79E-10	5.60E-12	1.21E-08	1.77E-14	2.74E-08	2410	1453	3061	1086	3003
CCL18	-1.74487	-2.22015	-1.60182	-2.0523	-2.24177	6.79E-10	1.67E-10	9.07E-12	2.11E-10	1.80E-11	2411	1900	1858	2157	1842
FAU	1.214483	1.060954	1.162242	1.301511	1.097337	6.84E-10	1.14E-08	7.33E-09	1.19E-11	2.20E-11	2412	2574	2948	1791	1868
H2BC7	1.798708	1.592563	1.947569	1.80238	1.722806	6.87E-10	5.22E-10	1.37E-12	2.64E-12	4.31E-11	2413	2072	1626	1609	1938
ZNF519	-0.90874	-0.55029	-1.03127	-0.71054	-0.69851	6.88E-10	3.07E-06	5.64E-11	5.52E-07	4.66E-08	2414	3767	2124	3518	3112
FBXO8	-0.79418	-0.67935	-0.52622	-0.65939	-0.67084	6.98E-10	7.42E-10	5.83E-07	1.11E-08	2.99E-07	2415	2127	3900	2778	3503
SLC5A10	-0.82876	-0.89714	-1.08708	-1.12886	-0.90626	6.98E-10	2.61E-10	1.54E-12	4.88E-11	2.14E-06	2416	1964	1640	1975	3899
MLXIPL	2.139788	1.96336	2.026391	1.805752	2.070296	7.00E-10	5.57E-14	2.30E-15	9.07E-12	6.41E-12	2417	985	999	1744	1703
H1-2	2.017904	1.510312	2.040984	1.842274	1.961796	7.02E-10	3.42E-08	2.38E-15	2.54E-09	7.40E-13	2418	2765	1004	2555	1447
CDHR5	2.182497	2.160951	2.091655	1.985006	1.427353	7.03E-10	1.18E-09	4.38E-08	3.15E-12	2.77E-05	2419	2196	3346	1631	4540
GTF2F2	1.301552	1.291924	1.194081	1.203691	1.326856	7.27E-10	3.52E-10	2.75E-09	2.17E-11	6.26E-10	2420	2021	2770	1873	2343
CYSTM1	1.446982	1.231046	2.025491	1.526539	1.816774	7.27E-10	1.57E-09	5.07E-16	1.05E-11	3.80E-14	2421	2236	878	1767	1126
SRRM2	0.738294	0.408767	0.423808	0.652634	0.308293	7.28E-10	0.001417	0.000243	1.80E-06	0.003786	2422	5637	5507	3773	6105

NFIX	0.918752	1.301991	1.287809	1.458834	0.943692	7.37E-10	2.29E-12	1.48E-13	3.35E-13	3.60E-07	2423	1333	1374	1377	3546
TCIRG1	-1.25539	-1.48374	-1.32319	-1.37342	-1.52143	7.47E-10	3.79E-11	1.72E-09	4.63E-12	1.73E-11	2424	1704	2662	1669	1835
MRPL15	0.816922	0.900238	1.137421	0.888523	0.678173	7.51E-10	8.82E-13	8.14E-16	5.36E-11	5.91E-05	2425	1243	919	1984	4760
PLK3	-1.00997	-1.04463	-1.17777	-1.09234	-1.38732	7.52E-10	1.72E-10	1.12E-13	2.66E-11	9.56E-19	2426	1904	1330	1900	379
RIN2	-0.98384	-0.76529	-1.17068	-0.73548	-0.88199	7.53E-10	3.39E-05	1.20E-07	8.21E-05	2.15E-05	2427	4363	3560	4805	4483
TPPP3	-1.11482	-0.97422	-0.88517	-0.9328	-1.21458	7.59E-10	2.79E-06	6.33E-06	9.45E-06	1.07E-08	2428	3741	4463	4195	2830
CCDC13	-0.99105	-0.59229	-1.08777	-1.00629	-1.01065	7.61E-10	4.87E-06	1.37E-08	2.06E-08	1.74E-08	2429	3857	3085	2893	2915
MSI2	1.293838	1.253332	0.961513	1.296834	0.816536	7.61E-10	4.98E-10	6.46E-09	5.62E-11	4.68E-06	2430	2065	2926	1991	4080
ANKRD40(	1.131077	1.133467	0.952885	1.134654	1.109366	7.61E-10	2.76E-11	2.61E-12	1.06E-10	3.69E-09	2431	1662	1701	2070	2638
RRM2	1.661538	1.657157	1.954386	1.839197	2.109784	7.62E-10	4.09E-11	6.22E-13	2.17E-14	7.27E-16	2432	1718	1539	1103	777
SMC4	0.919978	0.75441	0.664152	0.634746	0.540937	7.70E-10	2.36E-06	1.06E-05	8.89E-05	0.003032	2433	3699	4607	4825	6037
DIDO1	1.000612	0.919361	0.971837	1.051829	0.693789	7.72E-10	7.24E-09	7.73E-12	1.03E-09	1.36E-06	2434	2498	1838	2400	3803
RAB23	-1.1512	-1.02953	-0.62158	-0.95257	-0.69981	7.75E-10	5.08E-07	0.007242	4.57E-05	0.000171	2435	3315	6718	4648	5075
TK1	1.25796	0.970885	1.5054	1.113176	1.325656	7.75E-10	6.50E-07	9.32E-13	1.10E-07	1.15E-10	2436	3369	1582	3187	2071
RPL27A	1.966675	1.785544	1.655268	2.037341	1.823401	7.84E-10	2.12E-10	8.67E-08	1.25E-14	8.68E-11	2437	1938	3478	1057	2029
KRTAP4-7	-0.86013	-0.66902	-1.16622	-0.89556	-1.02618	7.88E-10	1.04E-09	3.40E-12	5.54E-08	9.84E-09	2438	2178	1735	3049	2810
IFIT5	-0.78177	-0.67552	-0.76115	-0.75633	-0.49932	7.89E-10	1.19E-07	5.12E-08	4.22E-08	0.000162	2439	3036	3378	3012	5063
NPAS2	1.14923	1.011689	1.611008	1.324024	1.177589	7.90E-10	1.44E-08	8.02E-15	3.11E-13	4.48E-11	2440	2621	1103	1367	1943
GOLGA7B	-0.76331	-0.51009	-0.75063	-0.64024	-0.86872	8.00E-10	2.34E-05	4.73E-10	3.01E-07	3.33E-11	2441	4259	2456	3374	1907
ITGA3	1.337911	0.861468	1.434153	0.882575	1.24119	8.01E-10	5.04E-05	3.22E-10	6.00E-05	3.93E-10	2442	4486	2396	4730	2265
FBXL19	0.729796	0.778615	0.824249	0.797339	0.716394	8.03E-10	9.20E-09	3.06E-10	6.38E-11	3.26E-08	2443	2541	2387	2007	3032
CERS5	-0.88058	-0.82565	-1.01544	-0.77835	-0.8244	8.08E-10	4.36E-07	2.55E-10	3.64E-06	2.72E-08	2444	3283	2359	3936	2996
MUC12	2.795564	2.869206	3.31459	2.880189	2.697342	8.18E-10	2.45E-09	5.05E-13	5.60E-11	2.81E-10	2445	2305	1514	1990	2218
TAGLN2	1.17941	0.771382	1.047265	0.984812	0.870605	8.41E-10	2.49E-06	1.51E-07	4.55E-09	4.65E-07	2446	3709	3602	2649	3591
UBLCP1	-0.67812	-0.51903	-0.46422	-0.66082	-0.98008	8.43E-10	0.000136	5.42E-05	3.89E-06	3.66E-10	2447	4784	5057	3959	2246
HNRNPA3	1.036982	1.062639	1.063346	1.203789	1.234318	8.44E-10	3.19E-08	4.84E-09	1.38E-09	2.74E-12	2448	2746	2871	2454	1604
CSF2RA	-1.89277	-1.22024	-1.34114	-1.62784	-1.54752	8.47E-10	0.000258	1.63E-05	3.23E-06	2.94E-06	2449	5004	4726	3910	3971
RPL28	1.436097	1.195159	1.361	1.362629	1.443626	8.52E-10	8.49E-08	1.88E-08	5.16E-09	6.52E-10	2450	2959	3160	2663	2355
ANLN	1.428417	1.48383	1.419734	1.536742	1.161418	8.56E-10	6.04E-09	2.92E-10	7.11E-10	8.80E-09	2451	2474	2381	2342	2786
ARRB2	-1.16553	-1.04882	-1.221	-1.13303	-1.36209	8.60E-10	7.30E-08	1.43E-10	9.82E-09	1.47E-10	2452	2924	2271	2761	2109
BASP1	-0.96849	-0.87454	-1.35117	-1.01706	-1.04392	8.63E-10	2.88E-10	3.16E-15	6.55E-09	2.28E-10	2453	1974	1027	2694	2186
COX16	0.897496	1.176062	1.027435	0.990434	1.234049	8.71E-10	7.54E-12	3.87E-11	4.35E-10	6.54E-15	2454	1499	2074	2260	944
DHX15	1.042625	0.954048	1.267125	1.122755	1.152952	8.87E-10	5.61E-09	3.72E-11	1.45E-11	2.00E-10	2455	2460	2071	1818	2154
PGK1	1.213205	1.01605	1.20372	1.302681	1.23685	8.94E-10	1.85E-08	2.06E-11	8.65E-10	2.25E-12	2456	2647	1983	2369	1576
RGL3	-1.02829	-0.86579	-0.70969	-1.21422	-0.94022	9.04E-10	3.43E-08	3.50E-05	1.13E-11	0.000114	2457	2766	4939	1779	4973
CTSB	-2.47654	-3.23497	-2.65797	-2.67403	-3.21647	9.05E-10	6.02E-14	1.18E-12	7.83E-12	5.88E-16	2458	996	1614	1724	765
DRGX	-0.86532	-0.56321	-0.93284	-0.82173	-0.78723	9.05E-10	1.34E-05	6.16E-08	6.68E-07	0.000172	2459	4107	3413	3567	5078
GDE1	0.838509	0.594697	1.122187	0.741515	0.799966	9.06E-10	0.000586	3.27E-08	0.000234	1.51E-06	2460	5320	3281	5115	3821
TLR4	-1.1713	-0.70177	-1.02276	-0.85038	-0.74342	9.06E-10	0.000656	7.94E-06	0.000139	0.000211	2461	5358	4529	4957	5135
ERN2	1.67004	1.695319	2.287141	1.859452	2.274106	9.36E-10	7.91E-11	7.08E-19	1.89E-10	6.04E-14	2462	1794	471	2142	1177
H3C4	1.528653	1.220655	1.711435	1.359665	1.380556	9.36E-10	1.27E-07	1.22E-10	2.55E-09	2.15E-09	2463	3048	2246	2556	2554
GK5	0.752938	0.705495	0.631315	0.800373	0.452796	9.40E-10	1.25E-07	3.29E-08	3.92E-09	1.09E-06	2464	3045	3283	2630	3758
CYB5R1	-0.64613	-0.44734	-0.39892	-0.43053	-0.422	9.44E-10	2.92E-05	0.000157	3.31E-05	0.001342	2465	4323	5347	4553	5744
PIGR	3.656808	4.052492	4.345302	4.290159	3.308297	9.49E-10	4.15E-11	2.52E-11	2.36E-14	1.35E-08	2466	1720	2011	1111	2875
ZNF423	-0.95885	-0.86117	-1.07621	-1.04759	-0.75535	9.52E-10	3.36E-09	6.44E-11	1.79E-10	2.80E-07	2467	2372	2142	2129	3488
CMAS	1.098178	1.075674	1.269443	1.093266	1.05611	9.53E-10	1.03E-10	1.21E-13	1.99E-10	8.23E-16	2468	1836	1342	2151	793
ARHGEF1(	0.971496	0.936193	1.42308	1.103088	1.410468	9.54E-10	1.41E-08	3.76E-14	1.34E-09	1.49E-17	2469	2619	1221	2448	523
RPS4X	1.928364	1.938557	1.539742	2.256338	1.927165	9.57E-10	1.29E-11	1.58E-08	3.30E-15	1.35E-11	2470	1560	3112	935	1805
AKR1C3	1.368698	1.423521	1.822445	1.534292	1.9372	9.71E-10	1.52E-08	1.69E-12	1.03E-11	5.53E-17	2471	2628	1653	1763	599
IP6K2	0.769123	0.783428	0.855755	0.723604	0.762356	9.79E-10	1.32E-09	1.23E-13	3.74E-08	1.98E-09	2472	2217	1345	2992	2535
F3	-3.38191	-3.63034	-3.11945	-3.41099	-3.34428	9.81E-10	8.33E-14	9.85E-10	4.94E-12	3.89E-12	2473	1023	2575	1676	1643
COA3	0.955519	1.011447	1.019672	1.193754	0.820837	9.93E-10	2.33E-06	8.76E-08	2.94E-09	5.37E-07	2474	3695	3482	2580	3615
CD274	-1.06769	-1.00581	-1.19956	-1.05141	-1.03619	1.00E-09	3.29E-08	5.83E-10	3.04E-07	7.42E-09	2475	2754	2487	3377	2750
LECT2	-0.94826	-0.90295	-0.76297	-1.03769	-0.83082	1.01E-09	9.64E-08	2.76E-07	5.03E-10	9.29E-10	2476	2984	3730	2282	2417
LONRF2	-0.74979	-0.61647	-0.73824	-0.70548	-0.94988	1.02E-09	2.87E-06	7.60E-09	8.18E-07	2.78E-10	2477	3751	2953	3597	2217
EHBP1L1	-1.10541	-1.23841	-1.11139	-1.0678	-1.36458	1.02E-09	3.15E-12	5.17E-10	5.38E-12	1.37E-11	2478	1370	2467	1683	1808
PTGER3	-1.13655	-0.8473	-1.12622	-0.90474	-0.98065	1.02E-09	3.95E-06	9.48E-10	3.77E-06	2.59E-11	2479	3818	2569	3944	1880
EHD1	-0.74537	-0.66035	-0.76571	-0.84199	-0.7828	1.03E-09	3.79E-07	2.40E-08	1.18E-09	3.88E-09	2480	3263	3196	2418	2647
TMC7	0.682033	0.411	0.619211	0.699641	0.510252	1.04E-09	0.000394	1.50E-05	3.83E-06	0.000765	2481	5164	4703	3954	5549
RPL18A	1.697582	1.832545	1.587659	1.934945	1.646529	1.05E-09	1.15E-11	2.31E-08	2.67E-16	2.37E-09	2482	1548	3190	768	2575
IPO4	1.114154	0.876544	1.232241	0.860901	0.839426	1.06E-09	1.11E-07	1.40E-10	2.83E-06	1.99E-07	2483	3019	2268	3885	3413
UVRAG	-0.71314	-0.58895	-0.66752	-0.51423	-0.70328	1.07E-09	1.38E-06	1.08E-07	1.28E-06	8.83E-09	2484	3559	3534	3700	2787
STXBP2	1.162101	1.246666	1.448621	1.369286	1.411618	1.08E-09	3.04E-10	2.07E-12	1.96E-11	1.87E-14	2485	1992	1672	1862	1046
SCAND1	1.057949	1.021414	0.926188	1.332385	0.953671	1.09E-09	8.84E-08	5.63E-08	2.34E-13	1.04E-08	2486	2970	3393	1331	2826
MDH1	1.021935	0.666315	1.067092	0.842115	0.747578	1.10E-09	1.40E-06	1.19E-08	2.89E-08	1.25E-05	2487	3563	3057	2947	4348
GPSM2	1.324109	1.349791	0.999606	1.435983	1.215554	1.11E-09	1.57E-12	1.75E-09	1.93E-12	2.38E-12	2489	1296	2669	1583	1581
SLPI	1.796002	1.677978	2.358087	1.603703	2.208533	1.12E-09	1.85E-06	2.37E-11	9.76E-08	1.71E-13	2490	3638	2004	3162	1279
HTR2C	-0.72679	-0.51603	-0.60899	-0.78105	-0.69288	1.15E-09	6.12E-07	6.40E-08	1.88E-11	3.73E-05	2492	3357	3423	1857	4630
NDUFA13	0.962515	0.980975	1.141028	1.051774	1.030197	1.15E-09	4.52E-12	6.67E-12	5.02E-14	4.28E-11	2493	1423	1815	1176	1937
GNA12	-0.81757	-0.83703	-0.81254	-0.84659	-0.72778	1.15E-09	2.70E-09	8.43E-11	1.64E-08	9.97E-09	2494	2327	2183	2853	2813
MAP4K4	-1.73421	-1.71461	-1.46636	-1.64544	-1.5512	1.16E-09	3.35E-12	2.86E-11	4.54E-11	1.77E-09	2495	1381	2037	1960	2520
UGT8	1.339178	1.489546	1.420427	1.572484	1.259924	1.16E-09	4.03E-09	2.04E-10	1.19E-10	3.74E-09	2496	2400	2324	2083	2642
PSMD2	0.823896	0.476145	0.943262	0.623182	0.766086	1.18E-09	6.90E-05	1.09E-09	6.93E-06	1.14E-09	2497	4582	2592	4106	2448

SLC66A1	-0.6671	-0.57737	-0.89436	-0.76751	-0.7347	1.18E-09	9.40E-08	3.69E-14	9.05E-10	3.52E-10	2498	2979	1216	2379	2242
PGS1	-0.70735	-0.7888	-0.5779	-0.73104	-0.58197	1.18E-09	4.95E-10	7.46E-07	5.66E-08	2.58E-07	2499	2064	3960	3053	3464
COQ9	0.737391	0.706562	0.996819	0.93524	0.97829	1.19E-09	2.74E-09	2.91E-11	9.07E-10	2.65E-14	2500	2331	2045	2380	1081
C1RL	-0.73997	-0.53773	-0.77383	-0.65685	-0.78641	1.22E-09	2.28E-05	1.79E-09	1.03E-07	2.88E-08	2501	4251	2675	3172	3014
NRN1L	-0.7658	-0.68429	-0.96541	-0.91284	-0.46958	1.22E-09	2.56E-08	6.50E-11	6.34E-10	0.000657	2502	2709	2145	2321	5501
RPLP0	1.845809	1.897994	1.561297	2.013284	1.822159	1.23E-09	2.55E-11	2.62E-08	6.30E-15	3.81E-12	2503	1655	3227	997	1639
TMCO1	0.927032	0.550773	0.866543	0.515974	0.725321	1.23E-09	0.000258	3.30E-08	0.000394	5.09E-08	2504	5005	3287	5301	3126
KCTD11	-0.84412	-0.48499	-0.94923	-0.69513	-1.08369	1.23E-09	3.68E-06	5.87E-10	1.60E-07	7.24E-12	2505	3804	2492	3254	1716
CCNB1	1.392449	1.38721	1.621041	1.371267	1.131906	1.23E-09	5.56E-11	1.15E-10	1.06E-12	7.28E-06	2506	1759	2236	1513	4196
BOLA3	0.769922	1.008708	0.907795	0.802648	0.498071	1.23E-09	3.17E-11	1.82E-11	1.33E-08	0.009948	2507	1672	1954	2813	6564
PSMA6	0.912148	0.687894	0.957195	0.895794	0.666814	1.24E-09	9.81E-06	1.68E-07	2.67E-07	3.57E-05	2508	4027	3625	3352	4619
CLN8	-1.08753	-0.76936	-1.22225	-1.03331	-1.22305	1.24E-09	0.000291	1.16E-10	9.29E-07	9.58E-11	2509	5051	2239	3617	2045
CYP4F12	1.414177	1.247188	1.217061	1.386567	1.282063	1.25E-09	6.84E-08	8.70E-07	6.18E-07	1.08E-07	2510	2902	3999	3543	3293
MLF2	1.139889	0.698779	0.974388	0.783784	0.637052	1.25E-09	2.35E-05	6.46E-07	2.11E-06	3.19E-05	2511	4261	3928	3815	4582
USP17L17	-1.36478	-0.76431	-1.13833	-0.96213	-0.80379	1.26E-09	0.000843	6.01E-07	0.000105	7.75E-05	2512	5442	3913	4870	4847
SEC22C	-0.91817	-0.654	-0.93361	-0.78924	-1.04981	1.26E-09	2.66E-06	7.29E-12	6.14E-07	2.92E-13	2513	3725	1831	3542	1331
SLC5A1	1.356374	1.332573	1.652355	1.636327	1.55002	1.26E-09	5.52E-08	8.89E-11	4.05E-12	1.27E-10	2514	2865	2197	1654	2081
ANKRD12	-1.16368	-1.23528	-1.12061	-1.28631	-1.33802	1.28E-09	1.67E-12	1.09E-09	1.24E-12	2.14E-12	2515	1306	2590	1528	1564
ACOT8	0.673802	0.742963	0.886319	0.689538	0.986175	1.29E-09	2.08E-08	4.69E-09	3.98E-08	1.85E-10	2516	2673	2863	3004	2140
TSPAN32	-0.82223	-0.53299	-0.98432	-0.66847	-1.00954	1.29E-09	2.11E-05	1.34E-11	6.90E-06	3.27E-13	2517	4228	1909	4104	1345
HAUS5	-0.69605	-0.50205	-0.65458	-0.66206	-0.55638	1.29E-09	9.37E-06	3.51E-07	8.00E-10	3.77E-05	2518	4015	3790	2362	4639
COL10A1	-1.78337	-1.44331	-1.33342	-1.51316	-2.12031	1.30E-09	2.19E-08	6.43E-08	4.05E-07	9.12E-09	2519	2681	3426	3448	2794
DOK4	1.599148	1.631509	2.219605	1.751748	1.860555	1.31E-09	1.05E-09	2.15E-13	1.01E-10	8.96E-11	2520	2181	1425	2064	2033
CD151	1.276347	1.019374	1.639135	1.36568	1.282523	1.31E-09	6.43E-09	1.22E-11	9.50E-12	1.34E-10	2521	2482	1901	1751	2093
YPEL5	-1.08705	-0.87948	-0.95797	-0.78161	-0.72609	1.31E-09	9.83E-08	1.07E-07	7.99E-06	0.000264	2522	2992	3528	4145	5201
STK38	0.994002	1.233748	1.305336	1.261399	1.174032	1.33E-09	3.86E-11	4.70E-11	1.09E-10	6.23E-11	2523	1710	2099	2071	1979
EIF3K	1.215178	1.049656	1.491717	0.984321	1.385223	1.34E-09	2.80E-08	6.55E-11	5.90E-07	1.72E-14	2524	2726	2146	3533	1039
YWHAE	1.298854	0.994145	1.358433	1.196768	1.836437	1.35E-09	1.04E-06	1.61E-08	2.35E-07	2.10E-11	2525	3490	3116	3325	1863
OR4F21	-0.79612	-0.6874	-1.04588	-0.83556	-0.81094	1.35E-09	2.02E-07	5.87E-12	1.29E-07	1.28E-09	2526	3150	1806	3212	2462
TBC1D1	-0.99637	-1.08184	-0.83676	-1.17972	-1.0455	1.36E-09	3.41E-10	2.10E-06	4.58E-12	5.86E-12	2527	2017	4193	1668	1691
FBXL2	-0.95818	-0.54624	-0.52136	-0.79109	-0.79551	1.36E-09	0.000211	0.000251	3.32E-06	5.19E-06	2528	4922	5516	3915	4118
RBM8A	0.942524	0.676729	0.776997	0.624911	0.90284	1.37E-09	8.36E-06	1.08E-07	1.52E-05	1.12E-10	2529	3983	3533	4329	2070
RALGAPB	1.000973	0.985491	0.740076	1.019692	0.757804	1.39E-09	9.73E-08	1.27E-06	2.51E-09	5.11E-07	2530	2986	4097	2553	3607
IL22RA1	1.419953	1.381556	1.538749	1.567407	0.814948	1.39E-09	6.58E-09	1.03E-12	1.29E-10	2.94E-05	2531	2489	1597	2094	4555
SCUBE2	-0.84712	-0.61609	-0.89361	-0.75502	-0.81609	1.39E-09	3.71E-08	2.03E-09	1.28E-07	1.45E-09	2532	2782	2707	3209	2486
MAP4K2	-0.94739	-0.64664	-1.03996	-0.63395	-0.69045	1.40E-09	2.07E-07	1.58E-10	1.93E-05	0.000119	2533	3160	2291	4389	4987
SLC34A3	-0.78977	-0.34125	-0.66435	-0.66183	-0.58801	1.41E-09	0.009642	1.78E-05	6.00E-07	0.000654	2534	6411	4749	3539	5495
CIB1	1.136531	0.866948	1.177676	1.038694	1.132728	1.42E-09	3.44E-08	4.70E-09	3.45E-09	9.63E-09	2535	2768	2865	2609	2808
RELB	-1.0862	-0.89646	-1.2438	-1.06137	-1.22405	1.43E-09	3.03E-06	2.06E-09	2.70E-07	6.00E-10	2536	3763	2709	3355	2337
ALG3	0.89469	0.868839	0.946083	1.007665	0.713234	1.43E-09	2.15E-10	2.24E-09	9.10E-12	1.10E-05	2537	1941	2728	1745	4302
TAF15	0.920788	0.688303	1.014469	0.757509	0.536342	1.44E-09	7.10E-08	6.59E-10	8.02E-08	1.69E-05	2538	2913	2513	3126	4427
MMAB	1.082782	1.058674	0.839503	1.027823	0.79532	1.44E-09	8.68E-09	3.05E-07	8.53E-08	5.69E-06	2539	2530	3764	3140	4141
RAB13	-1.14991	-1.28916	-1.0905	-1.25297	-1.14412	1.45E-09	1.05E-11	1.45E-09	4.63E-10	5.87E-09	2540	1541	2640	2270	2710
ABRACL	0.943225	0.951778	1.225573	0.989075	1.163195	1.47E-09	5.06E-10	3.92E-15	1.41E-09	1.43E-15	2541	2068	1044	2458	832
DEFA5	-0.92327	-0.99339	-1.01678	-1.18921	-1.29757	1.47E-09	5.36E-10	2.99E-07	8.12E-10	3.51E-11	2542	2078	3756	2363	1914
MBD1	-0.65059	-0.61364	-0.81384	-0.80458	-0.77609	1.47E-09	1.99E-08	1.78E-10	1.12E-09	8.03E-10	2543	2662	2307	2415	2390
KIAA1671	0.780169	0.69272	0.836052	0.709096	0.817783	1.50E-09	1.81E-06	5.40E-11	1.41E-07	1.96E-09	2544	3633	2118	3230	2534
FOXA2	1.536989	1.595315	1.168371	1.677942	1.302216	1.50E-09	4.58E-09	1.37E-07	8.98E-10	7.24E-08	2545	2419	3585	2375	3213
TNFRSF10	-1.07717	-0.75337	-1.17046	-0.85916	-1.19228	1.50E-09	2.32E-06	1.03E-09	5.49E-07	1.89E-11	2546	3694	2583	3516	1853
OR6C1	-0.70842	-0.70607	-0.6593	-0.90028	-0.60859	1.53E-09	1.17E-07	2.90E-08	1.08E-11	2.26E-06	2547	3032	3247	1772	3913
RPL12	1.683827	1.739905	1.4796	1.963441	1.854582	1.54E-09	1.35E-11	4.42E-08	1.32E-15	5.73E-11	2549	1562	3347	862	1966
CXCR3	-0.73011	-0.59316	-0.75159	-0.69187	-0.79495	1.54E-09	1.39E-07	1.93E-09	8.65E-08	1.03E-08	2550	3069	2689	3142	2824
TPMT	1.245743	1.233869	1.39795	1.364027	1.300427	1.55E-09	3.72E-12	1.91E-13	5.52E-11	4.06E-09	2551	1395	1411	1987	2655
RAP1A	-1.12489	-1.31414	-1.11772	-1.21891	-0.96616	1.56E-09	1.50E-12	1.17E-08	2.21E-11	5.18E-08	2552	1291	3051	1879	3134
GPC6	-1.72699	-1.61402	-1.7954	-1.62309	-1.86113	1.57E-09	1.36E-08	5.85E-11	6.30E-10	1.10E-11	2553	2609	2132	2320	1777
MRPL23	1.099891	1.159102	1.264201	1.097051	1.248776	1.59E-09	1.36E-11	3.37E-10	8.44E-12	2.49E-12	2554	1564	2409	1733	1590
HDAC1	1.014909	0.934675	1.263891	1.18414	0.925071	1.60E-09	7.87E-09	1.56E-13	3.16E-10	1.34E-09	2555	2515	1385	2213	2474
NDUFA5	0.794908	0.657951	0.835006	0.644624	0.783606	1.62E-09	7.19E-07	5.10E-11	6.24E-07	5.82E-11	2556	3398	2110	3547	1968
PNPLA5	-0.64781	-0.33701	-0.6651	-0.52655	-0.56008	1.65E-09	0.004037	2.10E-07	0.00022	4.38E-05	2557	6022	3668	5093	4683
USP11	-1.0474	-0.93197	-1.28008	-0.9143	-1.29849	1.66E-09	8.23E-09	1.31E-12	2.69E-07	6.95E-18	2558	2521	1621	3354	474
NAA10	1.015944	1.216656	0.911492	1.359851	0.945643	1.68E-09	4.29E-11	1.41E-07	1.04E-12	1.31E-08	2559	1725	3593	1509	2869
NCBP2	0.714544	0.76122	0.858589	0.857635	0.77076	1.70E-09	1.85E-09	1.35E-07	4.55E-08	2.18E-07	2560	2262	3578	3026	3434
MYO5C	1.168119	1.338263	1.031621	1.361589	1.452349	1.70E-09	4.74E-10	2.56E-09	1.27E-09	9.41E-09	2561	2058	2758	2432	2802
CITED2	-1.33801	-1.50267	-1.2022	-1.5675	-1.26253	1.70E-09	2.04E-09	8.36E-12	2.85E-09	1.01E-07	2562	2280	1846	2573	3277
LSM12	0.73204	0.617078	0.743938	0.657033	0.863225	1.72E-09	7.36E-07	4.55E-09	4.45E-07	3.45E-11	2563	3402	2860	3473	1912
LMAN2	0.929157	0.887929	0.836206	0.881493	0.683438	1.72E-09	1.37E-10	1.88E-08	9.01E-10	1.03E-06	2564	1871	3158	2376	3747
FGF7	-1.64169	-1.15004	-1.47731	-1.4487	-1.43708	1.73E-09	2.50E-05	2.13E-07	6.10E-07	1.35E-08	2565	4275	3672	3541	2878
ATP5PD	1.376212	1.296609	1.777907	1.353048	1.0904	1.74E-09	3.11E-10	3.36E-13	1.97E-08	5.22E-07	2567	1999	1471	2883	3611
H4C12	1.907313	1.464443	1.816401	1.920319	2.00933	1.74E-09	3.18E-09	1.10E-08	1.43E-12	4.94E-12	2568	2359	3031	1548	1673
VPS39	-0.71208	-0.54991	-0.4707	-0.37483	-0.74219	1.75E-09	3.66E-05	2.32E-05	0.003898	1.64E-07	2569	4388	4819	6145	3371
ADGRA3	0.893882	0.869782	0.89749	0.831023	0.678744	1.76E-09	1.72E-07	1.26E-10	2.28E-06	0.000129	2570	3117	2251	3832	5004
PPA2	0.918864	0.785495	1.031543	0.77922	1.065457	1.76E-09	5.07E-07	8.15E-11	1.87E-06	1.16E-06	2571	3313	2176	3790	3767
SLC9A3R1	1.235545	0.922637	1.038757	0.77825	0.94814	1.76E-09	5.08E-05	1.66E-06	0.000243	0.000176	2572	4489	4148	5134	5087

UBE2J1	-1.01826	-0.79446	-1.02646	-0.8102	-0.94459	1.76E-09	3.24E-06	1.12E-08	8.95E-05	2.54E-05	2573	3776	3036	4826	4520
SNX5	1.212886	1.217871	1.098709	1.179588	1.053959	1.76E-09	1.40E-10	2.49E-12	1.14E-10	2.16E-10	2574	1874	1693	2074	2177
URI1	0.911398	0.960982	1.11055	1.028764	1.069132	1.77E-09	1.32E-07	4.03E-08	2.37E-07	2.40E-09	2575	3056	3330	3327	2577
NUP155	0.915762	0.879239	0.833147	0.922244	0.767521	1.77E-09	3.97E-08	2.15E-09	9.14E-10	2.95E-05	2576	2798	2715	2383	4559
SNRPD1	1.472052	1.496287	1.589201	1.362508	1.138726	1.80E-09	5.29E-10	5.47E-11	1.26E-07	2.38E-06	2577	2074	2119	3205	3930
DDX23	0.799466	0.87956	1.037021	0.720583	0.866517	1.81E-09	3.37E-12	1.34E-16	9.03E-10	3.80E-10	2578	1382	788	2378	2254
EIF3D	1.17369	1.124912	1.27779	1.272807	1.340244	1.83E-09	7.93E-09	8.48E-10	4.85E-11	6.45E-13	2579	2516	2553	1971	1435
RPS9	1.517488	1.314847	1.285555	1.508961	1.516026	1.83E-09	1.80E-09	4.04E-08	7.00E-11	2.59E-10	2580	2255	3331	2024	2208
FHIP2B	-1.07707	-0.57708	-0.8428	-0.57235	-0.7767	1.84E-09	1.27E-05	5.80E-08	9.18E-05	2.91E-06	2581	4094	3402	4832	3967
IRF2	-0.77836	-0.5943	-0.46249	-0.44677	-0.46928	1.86E-09	4.05E-05	0.000305	0.001279	0.000122	2582	4420	5574	5714	4995
BAD	-0.67217	-0.47913	-0.50321	-0.54928	-0.53397	1.88E-09	8.97E-06	1.17E-06	1.05E-06	1.27E-06	2584	4011	4080	3653	3788
MKKS	1.114582	1.259043	1.25596	1.294218	1.331478	1.89E-09	3.68E-11	5.20E-16	6.51E-12	2.13E-14	2585	1699	881	1704	1062
RARS1	0.778529	0.764758	0.843516	0.918699	0.567413	1.89E-09	5.07E-09	1.73E-07	2.03E-12	6.41E-05	2586	2442	3629	1587	4791
CIBAR1	-1.24077	-1.10041	-0.83912	-0.95622	-1.18642	1.90E-09	7.83E-10	1.20E-07	3.77E-08	8.19E-10	2588	2135	3561	2995	2396
ARHGEF5	1.026227	1.132449	1.048014	0.916177	1.20653	1.90E-09	5.33E-10	1.02E-09	1.49E-09	5.83E-14	2589	2075	2581	2472	1171
ETV4	1.830227	1.658213	1.478326	1.561659	1.934841	1.90E-09	2.82E-09	1.75E-07	7.02E-08	9.80E-11	2590	2337	3636	3094	2049
PSME2	1.03546	0.588345	0.979038	0.70153	1.052889	1.92E-09	0.000509	1.32E-07	9.22E-05	2.69E-11	2591	5259	3574	4834	1884
CDC42EP5	1.474113	1.252596	1.773176	1.587173	1.709792	1.98E-09	1.76E-06	8.38E-11	2.92E-09	6.39E-08	2592	3621	2181	2577	3172
TMED2	1.224974	0.956971	1.174326	1.157068	1.144354	1.98E-09	1.78E-08	2.51E-08	5.07E-09	1.06E-13	2593	2642	3214	2659	1224
GATAD2A	0.965671	0.835389	0.995204	0.813995	0.927266	1.98E-09	1.70E-07	1.39E-10	1.40E-07	4.82E-10	2594	3114	2266	3228	2299
PLEK2	1.278246	1.318315	1.625712	1.298579	1.33082	2.01E-09	8.31E-13	3.42E-14	5.55E-11	4.43E-10	2595	1235	1210	1988	2288
RPS6	2.14107	2.281053	1.987752	2.410034	2.267684	2.01E-09	2.79E-11	1.36E-08	2.12E-14	1.14E-11	2596	1663	3083	1100	1785
MEDAG	-1.58611	-1.09143	-0.93063	-1.272	-1.18437	2.03E-09	2.12E-05	4.33E-06	2.66E-06	5.29E-09	2597	4232	4368	3866	2692
LNPEP	-0.77778	-0.75166	-0.61217	-0.79172	-0.86949	2.04E-09	3.65E-06	1.37E-06	3.32E-06	2.39E-08	2598	3799	4112	3916	2972
TM2D3	-0.88597	-0.4973	-0.61538	-0.65124	-0.78192	2.04E-09	0.001315	1.45E-05	4.08E-05	6.03E-07	2599	5617	4698	4613	3637
FOXO1	-0.87114	-0.61997	-0.72128	-0.82665	-0.80198	2.05E-09	1.05E-08	2.46E-09	4.39E-13	1.18E-09	2600	2560	2750	1408	2450
SMAD1	-0.85121	-0.83142	-0.78272	-0.71537	-0.95718	2.05E-09	1.31E-05	4.08E-06	6.40E-06	4.64E-06	2601	4101	4350	4080	4079
GLRX3	0.855162	0.808532	0.815298	0.791085	0.704229	2.05E-09	8.37E-10	6.60E-13	4.12E-09	1.78E-08	2602	2147	1544	2636	2922
GAR1	1.205929	1.030465	1.18146	1.011684	1.034924	2.05E-09	1.05E-07	1.52E-13	4.12E-09	6.40E-08	2603	3008	1381	2635	3173
MRPL57	1.322926	1.576731	1.45678	1.543091	1.866806	2.05E-09	1.63E-12	2.89E-12	5.02E-16	3.64E-17	2604	1303	1716	797	569
ANXA5	-2.32344	-3.29581	-2.05801	-2.99859	-2.88616	2.07E-09	1.46E-17	3.23E-08	1.02E-12	8.10E-14	2605	452	3279	1507	1203
EMG1	0.901825	0.753503	0.560079	0.550184	0.672139	2.08E-09	5.89E-07	0.000131	8.48E-05	5.23E-06	2606	3350	5288	4814	4121
STING1	-1.30331	-1.26517	-0.99498	-1.16111	-0.81231	2.10E-09	6.63E-10	1.06E-06	1.69E-07	4.94E-06	2607	2105	4052	3267	4099
C1QBP	1.70589	1.588826	1.863431	1.67257	1.963322	2.10E-09	2.96E-10	2.68E-10	3.55E-12	1.13E-14	2608	1983	2364	1638	1000
TROAP	1.341684	1.233032	1.033958	1.097672	0.973106	2.11E-09	2.64E-07	1.50E-06	3.61E-06	1.87E-07	2609	3191	4128	3932	3404
YBX1	1.461236	1.355622	1.44174	1.594851	1.587381	2.12E-09	1.54E-09	5.10E-08	3.84E-12	1.43E-11	2610	2233	3377	1647	1813
AGMAT	1.34197	1.185249	1.280813	1.06159	1.335731	2.12E-09	4.96E-07	9.85E-10	2.08E-07	4.29E-10	2611	3310	2576	3306	2284
RBCK1	1.357315	1.390418	1.398424	1.286389	1.261305	2.12E-09	2.93E-10	1.35E-11	1.30E-08	1.75E-09	2612	1981	1910	2810	2517
CAPN1	0.933754	0.984466	1.456462	1.272036	1.03208	2.13E-09	6.51E-10	1.33E-13	3.74E-12	2.10E-08	2613	2102	1360	1643	2952
ACYP2	-0.71437	-0.49899	-0.6979	-0.62916	-0.59434	2.13E-09	9.21E-05	8.41E-08	1.25E-06	2.70E-07	2614	4678	3471	3695	3479
EFNA4	0.89045	0.82917	0.949947	0.822431	0.898283	2.14E-09	3.87E-06	1.39E-09	4.98E-07	2.94E-09	2615	3809	2633	3491	2602
WNT8A	-0.88864	-0.54661	-0.90974	-0.73906	-0.68926	2.14E-09	7.78E-05	2.95E-08	9.69E-08	1.84E-06	2616	4616	3256	3161	3865
FBRSL1	0.998186	1.130301	0.764501	0.966548	0.704243	2.14E-09	1.61E-11	3.23E-08	1.94E-08	8.41E-08	2617	1585	3278	2880	3240
COMP	-0.86829	-0.65601	-0.65669	-0.96515	-1.49779	2.15E-09	3.31E-05	6.02E-06	1.53E-05	3.63E-07	2618	4360	4450	4331	3551
UQCC1	1.370755	1.21716	1.206627	1.277656	0.948	2.15E-09	3.45E-08	2.09E-08	2.81E-09	1.32E-05	2619	2769	3172	2571	4368
TMEM68	0.871112	0.757217	1.004951	0.878197	0.620383	2.15E-09	4.86E-07	2.85E-08	1.95E-06	0.000139	2620	3308	3245	3802	5029
AGO2	0.895711	0.972743	1.178915	1.152268	0.789957	2.15E-09	1.69E-09	4.58E-11	1.52E-10	2.05E-09	2621	2246	2095	2110	2540
PNPLA2	1.359047	1.081436	1.262434	1.149564	1.032294	2.18E-09	8.62E-07	1.12E-07	2.37E-10	3.01E-06	2622	3437	3546	2175	3983
FMOD	-2.27867	-2.1438	-1.8191	-2.0734	-2.0219	2.18E-09	1.62E-14	3.07E-12	3.74E-12	1.28E-12	2623	873	1727	1642	1509
CBLB	-1.12749	-0.96434	-0.96418	-0.94824	-0.73711	2.18E-09	4.38E-07	1.12E-09	1.86E-07	6.82E-05	2624	3284	2603	3284	4814
MRPS18A	1.02501	1.001184	1.012194	0.794823	1.338433	2.22E-09	1.72E-07	5.41E-09	4.40E-07	6.30E-11	2625	3118	2889	3468	1985
TXLNA	-0.85675	-0.76979	-0.60019	-0.51668	-0.65532	2.23E-09	8.75E-09	0.000132	0.000195	1.41E-05	2626	2533	5291	5052	4380
PSMD12	0.723162	0.938653	0.826397	0.901775	0.900441	2.23E-09	1.00E-11	1.56E-12	3.95E-11	1.26E-09	2627	1534	1642	1946	2459
RFC3	1.071136	1.206049	1.26019	1.0258	1.491026	2.24E-09	8.22E-11	1.78E-15	6.46E-10	1.21E-14	2628	1800	985	2327	1007
MRPL27	0.71465	0.783348	0.633573	0.943682	0.491416	2.26E-09	7.78E-10	1.74E-10	1.06E-12	3.87E-06	2629	2133	2305	1514	4042
POLR2J	0.764246	0.77857	0.715619	0.733418	0.70559	2.26E-09	4.57E-09	1.74E-10	3.57E-08	8.82E-10	2630	2416	2304	2983	2410
NPC2	-0.96925	-0.8955	-0.90803	-0.96677	-0.93628	2.27E-09	6.27E-07	5.75E-08	1.33E-09	7.60E-08	2631	3362	3400	2445	3223
GNB2	1.230125	0.79565	1.417285	1.033006	1.311684	2.28E-09	9.77E-08	1.06E-13	5.18E-08	2.73E-11	2632	2988	1324	3042	1887
ATP8B1	1.006239	0.860826	1.146823	0.964386	0.896997	2.28E-09	1.23E-07	1.77E-11	2.37E-07	2.25E-05	2634	3044	1945	3330	4493
ARHGAP3	0.938262	0.926969	1.317085	1.07237	0.925279	2.30E-09	1.11E-08	6.47E-14	3.57E-11	2.52E-10	2635	2570	1271	1935	2200
BLOC1S1	0.965136	0.670842	1.020941	0.825619	1.023914	2.31E-09	6.75E-06	4.07E-11	4.05E-08	9.73E-12	2637	3928	2084	3008	1761
SHC1	-1.02803	-1.3019	-1.00698	-1.22518	-1.1975	2.32E-09	7.38E-12	2.37E-09	3.68E-11	2.47E-12	2638	1492	2737	1942	1588
MIPEP	0.975763	0.889248	0.728051	0.835614	0.712082	2.33E-09	9.84E-11	1.40E-09	1.99E-10	1.00E-07	2639	1831	2635	2150	3273
PILRB	0.994607	1.284564	0.952105	1.177958	1.14338	2.37E-09	1.51E-13	1.35E-10	4.29E-12	2.31E-11	2640	1068	2263	1662	1871
SUFU	-0.97624	-0.78111	-1.03836	-0.87668	-1.09011	2.38E-09	3.56E-08	6.51E-14	6.55E-11	4.96E-10	2641	2776	1273	2010	2311
MRPL13	1.09684	1.02788	1.319845	1.089096	1.04934	2.40E-09	3.58E-11	2.95E-12	2.01E-10	1.29E-09	2642	1690	1720	2153	2465
HLA-DQA1	-1.1164	-1.31212	-1.5215	-1.40134	-1.97027	2.42E-09	1.21E-05	5.11E-07	3.80E-06	6.61E-09	2643	4083	3869	3947	2727
U2AF1	1.04357	0.898425	1.260018	0.878181	1.134174	2.44E-09	9.54E-07	1.14E-09	8.86E-07	2.72E-11	2644	3471	2607	3608	1886
NDUFA7	1.038974	1.187221	1.077245	1.273631	0.8948	2.48E-09	4.03E-10	1.45E-09	2.38E-13	6.13E-10	2645	2040	2639	1334	2342
FKRP	-0.72819	-0.50902	-0.78243	-0.55486	-0.69043	2.55E-09	4.53E-05	1.55E-10	1.22E-05	1.16E-05	2646	4450	2287	4266	4323
NPIPA1	0.652943	0.692981	0.759657	0.874774	0.841641	2.55E-09	2.89E-10	3.17E-11	1.17E-11	3.91E-10	2647	1976	2052	1785	2264
NACC1	0.644391	0.717952	0.792641	0.713148	0.467949	2.55E-09	5.53E-10	2.10E-13	2.05E-09	2.77E-07	2648	2085	1423	2519	3484
MTRNR2L	-0.64816	-0.44499	-0.62106	-0.62127	-0.4101	2.57E-09	0.000131	1.31E-05	1.55E-05	0.00135	2649	4771	4673	4336	5745

CSRNP2	-0.79585	-0.76267	-0.65038	-0.74793	-0.60141	2.59E-09	4.57E-07	5.70E-07	9.99E-07	7.49E-06	2650	3298	3893	3642	4209
RPS7	1.931592	1.867433	1.608509	2.139236	2.26241	2.61E-09	2.06E-10	2.82E-08	2.02E-13	4.21E-14	2651	1930	3242	1314	1140
ARMC9	-1.35881	-1.39905	-1.26304	-1.46408	-1.50036	2.61E-09	1.41E-10	2.08E-10	1.53E-09	7.40E-12	2652	1876	2330	2477	1719
CTNND1	1.204145	1.153731	1.611243	1.39933	1.302518	2.61E-09	5.70E-11	1.52E-12	7.51E-13	5.42E-11	2653	1762	1639	1467	1956
CSTB	1.298975	0.886208	1.286068	0.880022	0.896605	2.62E-09	7.06E-07	1.42E-08	7.76E-06	3.53E-07	2654	3392	3097	4137	3539
DDHD2	-0.90169	-0.67828	-1.06535	-0.95125	-1.24623	2.63E-09	8.83E-07	6.11E-09	1.12E-08	5.09E-12	2655	3448	2918	2780	1677
KHDC4	0.9675	0.967694	0.812445	0.95999	1.054575	2.64E-09	2.74E-10	6.89E-09	1.47E-09	9.25E-12	2656	1971	2937	2470	1751
MRPL42	0.868752	0.991043	1.020756	1.061867	0.799844	2.66E-09	4.59E-11	1.34E-08	1.53E-13	1.45E-08	2657	1735	3081	1283	2888
R3HCC1	-0.80604	-0.54835	-0.65188	-0.5753	-0.74321	2.72E-09	3.50E-05	6.51E-07	4.40E-06	1.22E-08	2658	4370	3929	3989	2861
SYNCRIP	1.261472	0.993549	1.374583	1.083638	1.061198	2.73E-09	3.50E-06	8.12E-09	1.86E-06	1.75E-09	2659	3787	2972	3787	2516
DAZAP2	0.881377	0.489929	1.00182	0.702424	0.928929	2.76E-09	6.88E-05	1.88E-10	1.17E-06	1.52E-09	2660	4580	2314	3685	2491
ADAM33	-1.76132	-1.15494	-1.58266	-1.46163	-1.53516	2.76E-09	4.20E-05	3.24E-08	2.83E-06	6.45E-10	2661	4431	3280	3884	2351
LRRC8A	-0.90954	-1.01045	-0.73638	-0.91608	-0.71319	2.77E-09	4.37E-11	4.10E-07	1.50E-11	1.70E-05	2662	1730	3826	1826	4428
ABCC5	-0.75604	-0.52888	-0.64802	-0.50292	-0.77854	2.79E-09	2.16E-06	5.28E-07	0.000296	6.82E-09	2663	3680	3884	5213	2729
MTX3	-0.78214	-0.6992	-0.82615	-0.69393	-0.75505	2.80E-09	1.61E-07	4.94E-11	4.44E-08	6.11E-10	2664	3095	2108	3024	2340
FHIP2A	-1.12372	-0.83032	-0.73388	-0.86666	-0.77808	2.81E-09	2.83E-06	4.66E-07	3.40E-06	3.28E-07	2665	3747	3851	3922	3521
CTPS2	0.70836	0.817495	0.63072	0.777464	0.929696	2.82E-09	2.40E-11	2.64E-08	8.83E-10	6.99E-14	2666	1643	3229	2373	1189
CLEC4E	-1.24052	-1.25615	-1.36293	-1.47398	-1.24038	2.84E-09	1.35E-07	1.73E-06	3.88E-09	1.24E-06	2667	3062	4158	2629	3782
AP3D1	0.885004	0.623602	0.52364	0.767133	0.653008	2.91E-09	1.18E-05	1.20E-05	6.59E-06	1.67E-05	2668	4075	4645	4088	4423
EPHA2	1.462649	1.126947	1.802662	1.320209	1.322591	2.96E-09	1.11E-05	6.72E-11	5.23E-06	4.87E-10	2669	4055	2149	4035	2302
CCL5	-1.38602	-1.41672	-2.0799	-1.67924	-1.84154	2.96E-09	7.10E-13	6.55E-20	4.87E-14	4.26E-13	2670	1221	383	1174	1375
PPIH	0.730239	1.000739	0.66951	0.896327	0.884556	2.97E-09	5.44E-14	6.15E-09	1.11E-11	2.25E-10	2671	982	2920	1778	2184
HOXD12	-0.75363	-0.5498	-0.5946	-0.53906	-0.58938	2.98E-09	1.47E-06	2.64E-06	5.77E-07	4.82E-08	2673	3578	4249	3527	3116
UBR1	-0.67449	-0.60847	-0.53802	-0.68579	-0.46368	2.98E-09	1.69E-07	5.73E-08	4.16E-09	7.03E-06	2674	3111	3399	2638	4184
ALS2CL	1.36481	1.331881	1.431525	1.052973	1.307532	2.99E-09	2.96E-06	5.33E-07	0.000101	4.00E-08	2675	3756	3886	4857	3072
PPM1G	1.010327	0.93675	1.035187	0.933376	0.899401	2.99E-09	1.72E-08	8.49E-10	1.23E-08	4.27E-09	2676	2638	2554	2798	2665
API5	0.827156	1.018212	1.182231	1.123516	0.768896	3.00E-09	1.83E-10	9.30E-14	3.80E-12	1.26E-08	2677	1910	1313	1645	2864
RSAD1	-0.69481	-0.4128	-0.69127	-0.46858	-0.55057	3.01E-09	2.61E-05	1.15E-10	1.32E-05	7.84E-07	2678	4288	2237	4281	3688
SYBU	0.758562	0.575458	1.083623	0.729413	0.558878	3.02E-09	6.85E-06	2.40E-07	0.000155	0.003531	2679	3931	3698	4982	6078
AZGP1	1.871338	1.817022	2.256726	2.360048	1.936062	3.03E-09	6.07E-09	5.34E-13	1.26E-11	7.08E-11	2680	2476	1520	1801	2008
IGSF9B	-0.79116	-0.81139	-0.79551	-0.97026	-0.79184	3.03E-09	3.66E-06	1.67E-06	3.80E-08	1.34E-07	2681	3801	4149	2996	3334
SH3BGRL	-1.40042	-1.68297	-1.74648	-1.5217	-1.5673	3.03E-09	7.79E-13	1.08E-11	2.14E-09	8.04E-12	2682	1230	1884	2525	1730
USP18	-0.8908	-0.46213	-0.90409	-0.6333	-1.12336	3.07E-09	0.000533	1.45E-08	0.000263	9.66E-15	2683	5283	3101	5165	979
ING4	-0.67573	-0.39594	-0.44891	-0.49486	-0.6546	3.08E-09	0.00054	7.76E-05	1.84E-05	3.20E-05	2684	5288	5145	4376	4584
KIF3B	0.832292	0.565569	0.856455	0.854354	0.592034	3.08E-09	7.16E-05	1.36E-07	2.08E-06	4.98E-05	2685	4592	3581	3813	4720
CERK	-1.4183	-1.18042	-1.27817	-1.36796	-1.4303	3.09E-09	3.32E-08	3.11E-09	5.13E-09	8.94E-11	2686	2756	2793	2661	2032
INVS	-0.83357	-0.82913	-0.50793	-0.74163	-0.61393	3.11E-09	1.85E-06	0.000421	5.70E-05	1.06E-05	2687	3637	5683	4715	4289
IDS	-1.22069	-1.17636	-1.16883	-1.16681	-1.45498	3.12E-09	8.22E-11	8.60E-11	1.10E-11	8.84E-11	2688	1799	2187	1777	2030
PICALM	-0.93789	-1.16183	-0.74922	-0.90603	-0.93069	3.12E-09	1.17E-11	6.12E-07	1.56E-08	1.14E-07	2689	1550	3920	2838	3306
SLCO3A1	-0.90692	-0.77847	-0.80936	-0.87807	-1.15731	3.12E-09	1.07E-09	1.45E-09	9.99E-11	3.16E-15	2690	2186	2637	2063	878
ZNF302	-0.80257	-0.75889	-0.77283	-0.79705	-0.39732	3.14E-09	5.58E-07	1.70E-07	6.75E-08	0.000997	2691	3333	3627	3088	5630
VCL	-1.36175	-1.35505	-1.14749	-1.19825	-1.12926	3.15E-09	1.21E-09	1.72E-08	1.64E-08	6.37E-07	2692	2200	3136	2850	3649
PPP6R2	-0.62005	-0.41618	-0.53902	-0.55207	-0.49535	3.16E-09	9.14E-05	9.48E-07	1.31E-05	7.16E-06	2693	4676	4022	4279	4189
VDR	0.881477	0.748727	1.209934	0.795105	0.857626	3.17E-09	1.25E-08	3.14E-15	2.38E-07	2.08E-08	2694	2597	1025	3331	2950
TRIM25	1.044048	1.001695	0.924252	0.904822	0.545623	3.21E-09	1.31E-06	1.25E-07	1.34E-05	0.002287	2695	3551	3566	4285	5957
ZNF580	-0.77745	-0.62787	-0.78084	-0.81538	-0.92007	3.22E-09	1.37E-07	5.54E-09	1.51E-09	3.85E-12	2696	3065	2897	2474	1641
PCARE	-0.84957	-0.6973	-0.74989	-1.10567	-0.79028	3.24E-09	2.78E-06	0.000171	3.08E-11	5.74E-06	2697	3740	5381	1910	4143
CHTOP	0.789909	0.865195	0.738228	0.751851	0.441005	3.24E-09	1.93E-11	3.94E-12	2.75E-08	0.000102	2698	1610	1751	2936	4940
LSMEM1	-0.63756	-0.29812	-0.43979	-0.48345	-0.7357	3.26E-09	0.002406	0.000444	3.31E-05	5.59E-06	2699	5829	5702	4554	4133
PLD2	-0.93372	-0.70163	-0.73743	-0.84298	-0.8955	3.26E-09	6.10E-07	1.37E-06	1.69E-09	5.43E-09	2700	3356	4111	2496	2699
NCLN	0.985692	1.137732	1.281675	1.19801	0.867149	3.30E-09	7.45E-12	4.59E-11	2.08E-14	6.27E-08	2701	1496	2096	1098	3169
DMWD	-0.6515	-0.38678	-0.43646	-0.47793	-0.37904	3.30E-09	0.000217	1.50E-05	2.34E-05	0.00043	2702	4935	4704	4428	5357
PDCD10	0.695861	0.345942	0.686362	0.564363	0.678988	3.37E-09	0.002349	1.68E-06	3.55E-05	3.41E-06	2703	5820	4152	4569	4009
EPAS1	-1.91388	-2.45646	-1.60805	-2.31706	-1.81594	3.48E-09	5.87E-12	8.11E-09	2.04E-11	6.35E-10	2705	1461	2971	1865	2347
H2BS1	1.709325	1.403845	1.80816	1.574713	2.013989	3.49E-09	4.10E-08	8.46E-13	5.58E-09	2.04E-13	2706	2809	1570	2670	1299
ORAI2	-0.90194	-0.77003	-1.36457	-0.85953	-1.15525	3.51E-09	7.60E-09	7.13E-14	8.48E-08	4.94E-11	2707	2506	1283	3139	1949
WDFY3	-0.93841	-0.61058	-0.62355	-0.59758	-0.76203	3.56E-09	0.000179	6.42E-05	0.000252	9.04E-08	2708	4876	5099	5146	3258
RAB37	-0.89939	-0.4272	-0.52714	-0.66385	-0.70668	3.60E-09	0.005306	0.000513	2.92E-05	9.05E-08	2709	6125	5757	4507	3260
RPL22	1.555896	1.360858	1.432259	1.565883	1.743893	3.62E-09	1.92E-08	2.25E-07	1.03E-11	9.17E-13	2710	2652	3682	1762	1470
FKBP10	-2.12305	-2.13017	-2.17273	-1.94511	-2.56443	3.63E-09	1.76E-09	2.36E-11	8.17E-10	2.74E-14	2711	2252	2002	2365	1087
TARDBP	0.863033	0.713939	0.86486	0.754391	0.786797	3.64E-09	1.69E-06	5.52E-09	1.74E-06	1.60E-10	2712	3614	2895	3764	2118
ZNF529	-0.86061	-0.62629	-0.84398	-0.86343	-0.43994	3.64E-09	8.58E-05	3.38E-08	1.35E-07	0.000799	2713	4651	3294	3220	5564
NEUROG2	-0.65617	-0.37059	-0.66123	-0.61616	-0.86313	3.64E-09	0.000293	3.01E-06	2.27E-05	2.07E-09	2714	5054	4282	4419	2544
TAF4	0.920248	1.029912	1.162359	1.182691	1.135257	3.64E-09	1.24E-08	9.58E-11	6.48E-10	4.58E-07	2715	2594	2212	2328	3588
RPS11	1.465871	1.451488	1.343054	1.582719	1.402996	3.65E-09	7.20E-10	4.36E-08	1.26E-11	8.96E-10	2716	2117	3345	1802	2414
CCL15	2.008468	1.63802	2.309679	1.862237	1.168037	3.72E-09	2.98E-10	8.79E-13	4.78E-09	0.000111	2718	1989	1573	2651	4964
DAPK3	-1.05278	-1.01998	-0.96771	-0.85536	-1.11267	3.73E-09	4.88E-08	9.75E-08	1.36E-07	9.32E-09	2719	2844	3509	3223	2797
AAMDC	-0.7396	-0.80304	-0.80349	-0.79661	-0.73063	3.74E-09	2.97E-08	2.21E-08	9.48E-09	1.37E-07	2720	2733	3182	2752	3341
EDEM1	-0.89135	-0.63219	-1.07819	-0.74	-1.26294	3.79E-09	2.25E-06	9.01E-11	1.36E-05	2.00E-12	2721	3689	2199	4289	1558
ITGBL1	-1.89265	-1.3617	-1.46987	-1.57261	-1.88201	3.81E-09	2.66E-08	1.15E-08	8.30E-09	1.62E-13	2722	2717	3045	2724	1275
SNAPC2	-0.93929	-0.88161	-1.12659	-0.84474	-1.08141	3.82E-09	2.82E-08	1.05E-17	4.62E-08	2.85E-17	2723	2728	632	3027	557
PDE3A	-1.07049	-0.9248	-1.27896	-1.23946	-1.0839	3.83E-09	3.42E-07	1.79E-09	8.57E-09	1.20E-08	2724	3241	2674	2732	2855
CLECL1	-0.9234	-0.70772	-0.87459	-0.94335	-1.28918	3.83E-09	4.54E-06	5.64E-08	1.84E-06	4.30E-08	2725	3841	3394	3780	3096

RNF185	-0.74522	-0.57687	-0.50278	-0.64493	-0.5371	3.84E-09	1.69E-06	2.79E-07	4.22E-08	4.72E-08	2726	3616	3732	3011	3115
OR7D4	-0.75456	-0.31048	-0.90641	-0.59719	-0.98223	3.88E-09	0.006157	2.11E-07	0.000222	9.26E-11	2727	6193	3670	5101	2042
CHMP7	-1.09559	-1.13125	-0.81667	-1.02083	-0.60389	3.91E-09	5.43E-08	1.04E-07	9.87E-07	0.000718	2728	2863	3523	3638	5531
NDUFA4	0.904855	0.622283	1.076179	0.878949	1.287892	3.93E-09	2.33E-07	2.22E-11	5.65E-09	5.66E-13	2729	3176	1994	2673	1412
EGFL6	-1.29918	-1.52098	-1.30881	-1.66307	-1.66074	3.95E-09	1.10E-09	7.01E-11	3.07E-11	9.67E-12	2730	2190	2152	1908	1758
HES1	0.983413	0.911233	1.268151	1.005104	1.31563	3.95E-09	2.06E-11	8.65E-13	2.21E-11	2.47E-11	2731	1617	1571	1880	1876
DNAJC1	-0.98372	-0.69703	-0.74094	-0.95341	-0.85435	3.95E-09	9.51E-06	8.53E-07	8.29E-08	7.37E-07	2732	4019	3995	3132	3672
PRR25	-0.94779	-0.47049	-0.75123	-0.74849	-0.7667	3.98E-09	0.004018	2.60E-05	1.51E-05	3.01E-06	2733	6015	4855	4328	3982
RPL11	1.643892	1.625555	1.490577	1.74669	1.835851	3.99E-09	3.35E-10	7.59E-08	7.80E-13	2.67E-12	2734	2012	3450	1469	1601
SYP	-0.6295	-0.36705	-0.63915	-0.50657	-0.6878	4.02E-09	0.001996	4.43E-06	0.000289	2.66E-07	2735	5768	4374	5205	3476
RPS12	1.956262	1.934979	1.758434	1.998792	2.00299	4.02E-09	4.89E-10	1.84E-08	1.22E-11	3.08E-11	2736	2062	3145	1796	1894
PHTF1	-0.90526	-0.85519	-1.19777	-0.87641	-1.04193	4.04E-09	1.32E-08	6.30E-13	5.94E-08	1.69E-11	2737	2603	1540	3063	1832
APRT	1.033092	1.263492	0.887874	1.375769	1.16728	4.11E-09	8.24E-13	2.72E-09	3.66E-15	1.48E-12	2738	1233	2767	948	1530
CCL21	-1.58849	-1.63114	-1.79653	-1.69076	-1.25403	4.12E-09	3.19E-09	1.75E-09	1.64E-08	3.09E-08	2739	2361	2668	2851	3027
ITPK1	1.183479	1.168258	1.625611	0.991207	1.409584	4.12E-09	2.63E-08	9.36E-15	4.14E-07	6.69E-11	2741	2715	1114	3451	1995
SOD1	1.572383	1.28461	1.916831	1.383074	1.238435	4.23E-09	1.29E-08	3.20E-13	1.96E-08	1.82E-08	2742	2600	1468	2882	2928
H2BC9	1.891522	0.825924	1.73453	1.285127	1.895107	4.26E-09	0.009212	2.87E-07	9.37E-05	2.32E-09	2743	6394	3737	4838	2572
EIF5	0.776827	1.021721	1.06351	0.824177	0.880296	4.26E-09	2.49E-09	8.61E-12	8.30E-07	3.09E-09	2744	2309	1848	3601	2608
IMMT	1.060095	1.214288	1.126497	1.191776	1.022139	4.29E-09	4.61E-09	2.48E-09	6.78E-09	1.40E-09	2745	2421	2752	2700	2483
SERINC1	-0.91693	-0.84977	-0.70478	-0.77294	-0.92153	4.30E-09	2.40E-10	7.81E-09	3.30E-09	2.90E-10	2746	1955	2961	2604	2222
PIK3CA	-0.74616	-0.30696	-0.53125	-0.32613	-0.33152	4.30E-09	0.007642	2.98E-06	0.005461	0.002279	2747	6304	4280	6281	5954
SRSF2	1.1883	1.13826	0.941038	1.229851	1.263449	4.31E-09	8.59E-08	1.36E-06	6.22E-09	1.56E-09	2748	2960	4110	2689	2497
ADGRD2	-0.82317	-0.75198	-1.04049	-0.83627	-0.79506	4.33E-09	4.97E-08	1.63E-12	4.82E-08	7.37E-08	2749	2847	1649	3031	3215
SYT5	-0.78014	-0.58219	-0.87077	-0.74548	-1.03746	4.34E-09	6.03E-06	5.94E-09	8.63E-08	2.98E-10	2750	3906	2910	3141	2226
ATP2C2	1.75106	1.317375	1.748449	1.481034	1.950096	4.34E-09	2.02E-07	7.38E-11	1.84E-07	8.26E-13	2751	3153	2159	3282	1462
PRMT1	0.922751	0.960736	0.821526	1.023536	0.985283	4.42E-09	3.65E-07	5.74E-06	1.66E-10	7.45E-09	2752	3256	4438	2113	2751
TMEM154	-0.95955	-0.7796	-1.07571	-0.96296	-1.16862	4.43E-09	6.50E-08	7.37E-14	1.27E-08	4.24E-10	2753	2892	1288	2801	2280
CIAO2B	1.125731	0.892583	1.07078	1.178335	1.023033	4.46E-09	1.06E-08	6.52E-09	2.61E-10	1.12E-07	2754	2563	2927	2184	3303
GALNT6	1.253883	1.54769	0.990191	1.41486	1.043229	4.49E-09	1.26E-13	1.81E-08	8.75E-11	2.00E-05	2755	1054	3144	2052	4465
TRA2B	0.737503	0.672862	0.8593	0.708817	0.924014	4.50E-09	2.34E-08	2.10E-10	2.36E-09	1.97E-12	2756	2691	2332	2543	1551
GRHL2	1.305707	1.782136	1.318537	1.772251	1.464244	4.54E-09	1.32E-14	2.72E-10	3.98E-15	1.04E-12	2757	851	2369	959	1486
THEM6	1.079123	1.454255	1.366269	1.586706	1.110352	4.57E-09	1.04E-09	3.26E-10	1.83E-11	9.36E-10	2758	2176	2402	1852	2421
NDUFV1	1.16778	1.279327	1.405939	1.506405	0.969139	4.59E-09	9.24E-09	1.73E-09	1.25E-11	8.65E-09	2759	2543	2665	1799	2782
C15orf48	2.061136	2.114325	2.402012	2.197878	1.888105	4.61E-09	1.93E-08	1.62E-09	1.18E-12	4.88E-08	2760	2653	2652	1522	3117
U2AF1L4	-0.91776	-0.77548	-0.89699	-0.81573	-1.00584	4.68E-09	1.35E-08	4.40E-12	2.74E-09	1.88E-14	2761	2606	1763	2566	1048
UNC5A	-1.03874	-0.47578	-0.86835	-0.43444	-0.97013	4.74E-09	0.006761	1.03E-06	0.006787	1.02E-08	2762	6236	4045	6374	2819
SLC23A2	-1.48515	-1.3572	-1.5946	-1.46311	-1.72507	4.74E-09	1.46E-09	1.79E-13	5.09E-10	1.41E-13	2763	2228	1404	2284	1262
ARF1	1.095336	0.607878	1.09943	0.69518	1.054861	4.80E-09	0.00017	5.77E-08	6.02E-05	6.08E-08	2764	4861	3401	4731	3163
ZNF397	-0.51342	-0.43578	-0.38015	-0.48037	-0.38799	4.81E-09	6.18E-06	1.50E-05	1.30E-06	9.80E-06	2765	3913	4701	3701	4273
CPNE3	0.694241	0.710457	0.945811	0.911303	0.872964	4.84E-09	1.04E-09	1.19E-08	1.15E-10	4.31E-08	2766	2177	3055	2076	3097
CEP55	0.941819	1.173884	0.9833	1.115694	0.797504	4.85E-09	2.49E-10	1.13E-08	1.85E-11	4.31E-07	2767	1960	3040	1854	3580
MUL1	-0.7455	-0.78219	-0.7475	-0.83613	-0.48881	4.87E-09	6.91E-08	9.76E-07	1.88E-08	0.000238	2768	2906	4028	2874	5174
SLC15A4	-0.81693	-0.55939	-0.46696	-0.56546	-1.05235	4.87E-09	4.00E-06	7.41E-05	6.25E-07	1.00E-08	2769	3819	5131	3548	2816
ABHD14B	1.07273	1.165345	1.534033	1.411115	1.689698	4.90E-09	1.32E-10	2.90E-11	8.11E-11	2.07E-16	2770	1866	2042	2042	694
PHTF2	-0.99162	-0.59063	-0.91196	-0.78824	-0.94346	4.91E-09	0.000771	8.40E-08	3.11E-05	1.54E-09	2771	5409	3469	4530	2495
DNAJB11	0.753932	0.519891	0.578213	0.701468	0.544743	4.91E-09	8.84E-06	7.83E-07	2.84E-08	0.000349	2772	4004	3974	2946	5276
HCCS	0.659575	0.829399	0.841313	0.953589	0.729082	4.91E-09	9.72E-11	2.39E-09	1.98E-09	3.20E-06	2773	1830	2738	2516	3996
YDJC	1.165945	1.532048	1.444312	1.517249	1.155394	4.98E-09	2.72E-09	1.50E-10	1.68E-09	1.18E-09	2774	2330	2281	2492	2451
TMEM184	1.366362	1.186661	1.090828	0.990282	1.410317	4.98E-09	4.82E-09	1.05E-06	2.52E-07	3.77E-10	2776	2433	4051	3341	2252
RALGDS	-0.76822	-0.78654	-0.76261	-0.93268	-0.75239	4.98E-09	1.41E-10	4.80E-12	2.59E-12	2.46E-10	2777	1877	1776	1605	2194
EFCAB13	-0.74818	-0.57872	-0.58163	-0.6986	-0.40064	4.98E-09	1.78E-05	7.92E-06	9.82E-06	0.007436	2778	4181	4526	4208	6405
VEGFB	-0.96455	-0.88358	-0.77601	-0.78464	-1.0431	5.00E-09	3.73E-08	3.66E-06	1.82E-06	2.25E-08	2779	2783	4323	3776	2965
ZNF391	-0.51659	-0.42796	-0.30492	-0.42446	-0.53733	5.00E-09	3.03E-07	0.00294	0.000131	1.10E-05	2780	3222	6351	4943	4305
NUP98	0.853463	0.988326	0.889555	1.05562	0.920635	5.02E-09	1.86E-09	8.67E-10	5.62E-12	1.40E-08	2782	2263	2556	1686	2884
LITAF	-1.22064	-1.07797	-1.1965	-0.82794	-1.38696	5.08E-09	3.49E-11	1.88E-13	4.36E-09	7.71E-14	2783	1686	1410	2643	1194
POR	1.032398	0.991929	0.84648	1.078765	0.976827	5.08E-09	9.57E-10	6.54E-10	2.39E-12	1.68E-10	2784	2168	2511	1598	2124
RABGGTB	0.894082	1.047877	0.833921	1.074417	0.768661	5.10E-09	4.66E-08	5.15E-07	3.37E-10	6.90E-08	2785	2837	3873	2221	3194
ZMIZ2	0.822792	0.992188	0.741214	1.05813	0.821947	5.14E-09	2.66E-08	2.33E-07	5.31E-09	4.44E-08	2786	2718	3690	2667	3102
CHKB	-1.01876	-0.61092	-0.72264	-0.67709	-0.9999	5.15E-09	6.85E-07	2.63E-09	1.77E-06	1.68E-12	2787	3384	2762	3767	1536
PSMD7	1.027424	0.867445	1.023319	1.155941	1.064034	5.16E-09	3.07E-08	1.27E-10	2.06E-11	1.75E-11	2788	2737	2254	1866	1837
COLGALT1	-0.78257	-0.77667	-0.77822	-0.71682	-1.0027	5.19E-09	1.28E-07	6.15E-09	9.30E-07	8.43E-12	2789	3049	2921	3618	1737
NKAPL	-0.82958	-0.71676	-0.57861	-0.92592	-0.90269	5.20E-09	3.34E-06	0.000188	2.19E-11	4.84E-07	2790	3780	5416	1875	3598
STT3A	0.864175	0.589112	0.543389	0.674695	0.362327	5.20E-09	1.72E-05	1.11E-06	4.61E-06	0.001579	2791	4168	4059	4002	5810
ATP5F1C	1.126521	1.02906	1.24635	1.104058	1.151163	5.21E-09	1.98E-08	2.30E-09	1.81E-09	1.93E-11	2792	2658	2732	2501	1855
LYZ	-3.31326	-4.04086	-2.44657	-3.25879	-2.95349	5.24E-09	5.51E-16	2.80E-06	3.66E-09	4.89E-08	2793	665	4260	2619	3118
GATD1	0.883778	1.220454	0.933002	1.249833	0.84597	5.28E-09	4.02E-11	7.68E-09	5.63E-13	1.76E-05	2794	1715	2957	1436	4434
CYP1B1	-0.7799	-0.45693	-0.83955	-0.86863	-1.06732	5.36E-09	0.00058	2.21E-05	0.00013	2.59E-07	2795	5316	4802	4938	3466
RBM14	0.582635	0.651456	0.531782	0.706323	0.419466	5.36E-09	7.84E-10	2.36E-09	1.67E-10	0.007281	2796	2137	2735	2117	6393
NOP2	0.854086	0.842979	0.728684	0.710452	0.475961	5.37E-09	8.52E-07	2.41E-08	1.85E-05	0.000688	2797	3433	3197	4378	5515
H1-0	1.168109	1.13814	1.598872	1.567377	1.01905	5.38E-09	4.71E-08	1.96E-09	1.08E-09	1.26E-05	2798	2840	2696	2408	4355
GABBR1	-1.52366	-0.98384	-1.75135	-1.40634	-1.3236	5.38E-09	1.80E-05	4.05E-13	4.93E-08	4.71E-07	2799	4186	1491	3035	3595
NFKBIA	-0.98036	-1.16432	-0.81238	-1.16893	-1.33862	5.39E-09	9.46E-07	5.44E-05	2.80E-08	8.94E-08	2800	3468	5058	2943	3256
FRMD8	-0.64223	-0.51969	-0.49046	-0.49997	-0.43472	5.42E-09	1.78E-07	1.47E-06	1.47E-07	0.00067	2801	3128	4122	3238	5507

ICOSLG	-1.0561	-0.40512	-0.69777	-0.62009	-0.82284	5.44E-09	0.002547	4.43E-06	1.33E-05	2.65E-07	2802	5848	4378	4282	3473
QARS1	1.106473	0.980055	1.210118	1.238425	1.351704	5.45E-09	3.50E-09	5.36E-10	1.43E-12	1.15E-12	2803	2378	2471	1549	1502
EFNB1	0.870589	1.149171	0.79484	1.081803	1.252026	5.45E-09	3.64E-11	1.18E-05	4.00E-08	4.77E-10	2804	1695	4635	3006	2298
HACD3	0.981835	0.704247	0.838803	0.729775	0.743986	5.47E-09	1.21E-05	6.27E-06	7.82E-07	2.95E-06	2805	4081	4461	3587	3972
COMTD1	1.016777	1.270413	0.881174	1.268627	0.939308	5.47E-09	9.66E-12	1.86E-08	4.40E-13	3.48E-09	2806	1525	3153	1409	2626
FCHO2	-0.7542	-0.94235	-0.77275	-0.60834	-0.96097	5.47E-09	4.65E-10	6.35E-08	1.11E-06	1.76E-07	2807	2053	3421	3661	3387
RNF138	-0.81258	-1.00979	-1.06581	-1.1118	-0.79036	5.48E-09	3.29E-09	2.16E-09	3.25E-09	1.37E-06	2808	2367	2716	2601	3806
ARF3	0.944581	0.545911	0.794211	0.40691	0.976306	5.49E-09	0.000299	1.06E-07	0.002082	7.73E-07	2809	5058	3526	5896	3686
PSME1	0.794471	0.409791	0.885011	0.621338	0.659449	5.52E-09	0.004366	3.86E-07	4.91E-05	5.85E-06	2810	6047	3812	4669	4148
PRSS8	1.644245	1.928141	2.046059	1.872694	1.513048	5.53E-09	5.08E-12	1.79E-11	4.19E-14	1.40E-08	2811	1441	1948	1166	2883
AK3	0.752918	1.117213	0.985927	1.325715	1.004362	5.53E-09	2.16E-09	2.53E-08	7.40E-12	5.67E-10	2812	2289	3216	1716	2326
RPS20	1.525585	1.563408	1.52401	1.717475	1.48887	5.53E-09	6.60E-11	6.88E-08	4.53E-14	1.21E-09	2813	1778	3440	1168	2456
KRTAP9-6	-0.85841	-0.71726	-1.26879	-0.90336	-0.98283	5.60E-09	1.42E-06	2.91E-11	5.37E-06	1.97E-08	2814	3567	2043	4040	2939
RPL32	1.841256	1.830798	1.602513	1.999132	1.876262	5.63E-09	7.04E-10	4.12E-07	8.28E-14	2.14E-10	2815	2113	3828	1224	2174
TENT5C	-2.36368	-2.1449	-3.40858	-2.53927	-2.86308	5.65E-09	2.65E-10	2.23E-14	2.54E-07	6.47E-10	2816	1967	1174	3343	2352
DNPH1	1.219026	1.064596	1.114135	0.918583	0.894917	5.70E-09	6.29E-09	5.40E-08	7.19E-08	3.58E-05	2817	2480	3390	3097	4620
ATP6AP1L	-0.72854	-0.52622	-0.79821	-0.55208	-0.67007	5.76E-09	3.61E-05	5.95E-10	7.48E-06	4.18E-09	2818	4380	2496	4125	2658
NAA50	0.986137	1.038942	1.117504	1.035615	1.295432	5.79E-09	5.33E-09	1.98E-10	1.75E-10	1.80E-10	2819	2449	2319	2124	2137
PMEL	-0.69004	-0.54125	-0.78589	-0.65381	-0.59826	5.80E-09	1.04E-06	6.64E-10	4.83E-09	2.23E-07	2820	3491	2514	2654	3442
CAMK2N2	-0.92079	-0.54305	-0.9451	-0.82757	-0.75744	5.81E-09	0.000528	9.98E-07	5.33E-06	2.13E-06	2821	5272	4036	4039	3897
TTC33	-0.65376	-0.37477	-0.86477	-0.67321	-0.48301	5.81E-09	0.00026	1.37E-09	7.36E-07	0.000338	2822	5006	2630	3576	5266
TOMM70	0.802361	0.95364	0.789541	0.896575	0.922784	5.87E-09	1.32E-10	1.13E-08	3.70E-12	1.71E-10	2823	1865	3039	1640	2128
TWF2	-0.89747	-0.58319	-0.80455	-0.64355	-0.93852	5.90E-09	0.000492	4.10E-07	0.000225	1.40E-13	2824	5246	3827	5104	1257
ELMO3	1.121446	1.313681	1.389274	1.382355	1.700394	5.90E-09	1.27E-11	6.84E-14	7.67E-12	2.23E-18	2825	1556	1275	1721	412
PBX2	-0.87155	-0.82905	-0.84339	-0.9259	-0.66641	5.91E-09	6.14E-08	5.54E-10	1.04E-07	4.24E-07	2826	2888	2478	3174	3576
ZNF557	-0.69279	-0.42421	-0.65244	-0.56973	-0.39948	5.92E-09	5.98E-05	1.56E-08	2.35E-06	0.000341	2827	4535	3108	3836	5268
BRIX1	0.903259	1.038056	1.215406	1.073253	0.781743	5.92E-09	3.49E-09	1.09E-12	7.67E-10	2.28E-06	2828	2377	1609	2357	3920
MTRF1L	-0.58136	-0.30155	-0.37226	-0.48511	-0.65551	5.98E-09	0.004894	0.000498	7.68E-06	2.99E-07	2829	6088	5743	4134	3502
POLR3D	-0.88169	-0.74319	-1.02635	-0.75317	-0.83389	5.99E-09	4.77E-08	1.40E-15	2.62E-08	2.93E-06	2830	2842	963	2927	3970
PLCB1	-1.61784	-0.95467	-1.34682	-1.10784	-1.24152	6.00E-09	0.000832	5.10E-07	0.000104	1.17E-05	2831	5436	3868	4868	4324
DUSP1	-2.22239	-2.49272	-2.3257	-2.46247	-2.73103	6.05E-09	4.76E-10	5.31E-11	4.16E-12	6.54E-11	2832	2059	2115	1657	1989
SMPDL3A	-1.39942	-1.07443	-0.84499	-1.05421	-1.46226	6.06E-09	9.75E-07	5.30E-05	7.51E-08	1.37E-07	2833	3473	5049	3104	3340
NegProbe-	-0.71557	-0.41308	-0.50637	-0.57197	-0.763	6.06E-09	0.000683	4.12E-05	1.91E-05	1.61E-09	2834	5373	4977	4385	2505
CCNC	0.845266	0.661903	1.045617	0.589211	0.681449	6.11E-09	5.12E-05	3.54E-08	0.000655	1.00E-05	2835	4492	3304	5470	4276
LMO4	-1.4234	-1.1893	-1.16069	-1.34772	-1.49176	6.20E-09	2.21E-08	6.73E-08	1.69E-09	1.29E-10	2836	2685	3435	2493	2086
H4C8	1.664536	1.15709	1.871621	1.383267	2.052811	6.25E-09	2.63E-07	2.57E-11	3.82E-08	1.49E-12	2837	3190	2016	2999	1531
BACH1	-1.06523	-1.03003	-1.03983	-0.92099	-1.1255	6.28E-09	8.80E-07	3.08E-08	2.16E-05	1.52E-08	2838	3446	3263	4410	2894
TEAD4	0.816574	0.769213	0.92498	0.734495	0.719702	6.40E-09	1.80E-06	7.80E-07	0.000198	0.000464	2839	3629	3969	5057	5387
CAMTA2	-1.06234	-0.95498	-0.73671	-1.05013	-0.57345	6.43E-09	1.45E-08	2.31E-05	5.27E-09	0.002746	2840	2622	4818	2666	6014
CD8B	-0.83263	-0.462	-0.72246	-0.53695	-0.58181	6.64E-09	0.000614	5.01E-07	0.000119	2.69E-05	2842	5333	3866	4907	4532
PTRH2	0.690216	0.986378	0.835508	0.942601	0.442913	6.66E-09	9.45E-11	1.82E-09	1.28E-08	0.006476	2843	1826	2678	2803	6344
RGS12	-0.82048	-0.69896	-0.75354	-0.88815	-0.76308	6.66E-09	3.82E-05	1.83E-06	3.82E-07	3.27E-07	2844	4401	4174	3436	3520
GNG10	-1.03723	-0.85455	-0.64152	-0.87699	-0.69452	6.71E-09	9.30E-07	2.31E-05	1.25E-06	1.90E-05	2845	3463	4817	3697	4454
SLITRK4	-0.80249	-0.59588	-0.69704	-0.5161	-0.65127	6.72E-09	5.51E-06	5.43E-09	2.75E-05	1.26E-07	2846	3885	2890	4483	3324
RAB17	1.031355	0.925001	0.871822	0.752285	1.045471	6.72E-09	5.31E-08	2.27E-06	3.62E-07	1.33E-06	2847	2854	4210	3422	3799
ZNF775	-0.77178	-0.51296	-0.6569	-0.52693	-0.67778	6.77E-09	1.23E-05	1.91E-08	7.32E-06	1.94E-08	2848	4089	3163	4117	2936
NUTF2	1.18035	1.133924	1.230021	1.375788	1.499758	6.78E-09	3.82E-10	1.16E-09	2.50E-14	5.36E-14	2849	2032	2611	1115	1161
SEPTIN11	-1.61404	-1.49767	-1.09243	-1.50866	-1.47131	6.78E-09	4.33E-12	1.01E-08	1.49E-13	1.83E-08	2850	1415	3013	1279	2930
ZNF483	-0.90322	-0.8076	-1.01363	-0.96848	-0.70149	6.85E-09	5.43E-08	9.51E-10	2.52E-09	5.38E-06	2852	2861	2570	2554	4129
DYNLRB1	1.135699	0.966322	1.319294	1.237589	1.383624	6.86E-09	1.68E-07	7.80E-10	4.36E-08	5.15E-11	2853	3109	2540	3020	1953
HRH1	-0.88138	-0.51012	-0.41208	-0.59701	-0.71123	6.89E-09	0.000141	0.000605	1.87E-05	1.67E-07	2854	4795	5794	4383	3376
SHB	0.846007	0.804634	1.207727	1.074545	1.05917	6.89E-09	4.43E-08	1.20E-11	1.88E-10	4.23E-08	2855	2823	1894	2141	3089
PLOD2	-1.91271	-1.74533	-1.20782	-1.60529	-1.83755	6.99E-09	7.71E-09	1.19E-05	8.32E-08	1.70E-08	2857	2510	4638	3134	2913
SEMA3A	-1.01827	-0.77067	-1.03913	-0.67759	-0.98307	7.04E-09	0.000311	4.97E-07	5.45E-05	3.79E-06	2858	5073	3864	4698	4036
FAT1	1.302576	1.091526	1.624109	1.537038	1.129528	7.22E-09	0.000196	1.74E-07	6.19E-08	4.43E-05	2859	4907	3630	3073	4685
SIVA1	0.741157	0.695535	0.883519	0.604356	0.753721	7.27E-09	1.96E-07	3.23E-10	1.12E-06	1.93E-07	2860	3143	2398	3668	3410
DIS3	1.069536	1.015787	0.885495	1.094058	1.032728	7.28E-09	7.00E-09	6.70E-10	6.36E-10	2.53E-12	2861	2495	2517	2323	1592
CT47B1	-0.65142	-0.42964	-0.80348	-0.56546	-0.63618	7.33E-09	0.00013	7.70E-12	9.82E-06	7.15E-07	2862	4768	1837	4207	3667
PKP4	1.043176	0.95071	1.31648	1.18624	1.533009	7.36E-09	4.65E-08	1.65E-12	5.03E-10	3.02E-17	2863	2835	1650	2281	559
HKDC1	1.867382	1.957113	2.729359	2.006901	1.980607	7.54E-09	2.17E-08	5.55E-17	1.40E-08	2.24E-12	2864	2679	721	2819	1575
SMIM26	1.108717	1.020277	0.888055	1.125567	1.036798	7.55E-09	7.98E-09	8.13E-09	1.89E-08	3.40E-09	2865	2517	2973	2876	2623
PIAS2	-0.77537	-0.72503	-0.85793	-0.73478	-0.54021	7.55E-09	4.52E-08	2.34E-10	2.09E-07	1.29E-05	2866	2827	2347	3307	4360
C12orf57	-1.54088	-1.79157	-1.54576	-1.836	-1.51014	7.62E-09	2.97E-10	3.20E-09	7.37E-11	2.50E-10	2867	1987	2806	2030	2197
DHCR7	1.469128	1.824449	1.779135	1.787534	1.538448	7.69E-09	9.86E-12	2.97E-11	1.39E-10	2.41E-11	2868	1529	2049	2102	1874
STARD13	-0.82587	-0.61299	-0.60371	-0.599	-0.48744	7.79E-09	2.43E-05	1.23E-05	2.97E-05	8.93E-05	2869	4267	4656	4516	4890
WDR43	1.353571	1.616145	1.592563	1.596925	1.256892	7.92E-09	4.67E-11	2.58E-12	4.47E-10	5.79E-10	2870	1740	1699	2267	2332
SCN8A	-0.66318	-0.3254	-0.81171	-0.5662	-0.59086	7.92E-09	0.000917	9.22E-08	2.01E-05	0.000114	2871	5478	3495	4399	4974
RAB7A	0.855197	0.720844	1.139244	0.865766	0.958939	7.99E-09	4.32E-08	4.45E-12	7.76E-09	2.96E-12	2872	2820	1765	2716	1612
PSMD14	0.786604	0.93674	0.997025	0.993676	1.073292	8.02E-09	4.56E-11	4.05E-13	1.40E-12	1.90E-16	2873	1734	1490	1545	685
NXF1	-0.79804	-0.6448	-0.62523	-0.55285	-0.75394	8.07E-09	4.11E-08	5.48E-07	8.24E-07	5.84E-09	2874	2810	3888	3599	2707
SLFNL1	-0.81918	-0.66569	-0.89738	-0.81471	-0.87369	8.09E-09	1.37E-06	7.82E-10	1.23E-08	5.96E-10	2875	3557	2541	2797	2336
SVBP	-0.82208	-0.75086	-1.42483	-1.11701	-1.10026	8.12E-09	5.47E-05	8.26E-06	0.00014	4.07E-05	2876	4514	4540	4958	4665
ZFTA	-0.73812	-0.39371	-0.62146	-0.50974	-0.63856	8.23E-09	0.000946	5.15E-08	2.40E-05	1.09E-06	2877	5493	3379	4436	3757

TECPR2	-0.8343	-0.74231	-0.6639	-0.91515	-0.57094	8.23E-09	1.19E-07	2.81E-07	6.18E-10	8.79E-07	2878	3038	3734	2317	3705
CAPRIN2	-0.64793	-0.3509	-0.51683	-0.46984	-0.78224	8.25E-09	0.000538	1.76E-06	9.42E-06	8.59E-11	2879	5287	4164	4194	2027
NFAM1	-0.738	-0.45727	-0.88145	-0.64997	-0.70389	8.27E-09	0.000178	5.71E-09	1.38E-05	2.90E-08	2880	4873	2900	4296	3017
ZG16B	1.330238	1.374605	1.981776	2.013043	2.110302	8.31E-09	1.64E-07	8.48E-11	6.84E-10	1.62E-14	2881	3099	2184	2334	1031
NUDC	0.841341	0.568948	0.911932	0.631301	1.061631	8.33E-09	3.66E-05	3.63E-07	1.26E-05	5.75E-10	2882	4387	3799	4272	2329
ADK	0.691436	0.692242	0.632676	0.717695	0.629251	8.34E-09	1.99E-07	2.11E-08	6.22E-09	8.39E-06	2883	3146	3176	2690	4239
MNX1	1.028891	1.276896	0.753485	1.156332	1.020514	8.40E-09	4.16E-10	2.18E-05	2.79E-07	4.12E-06	2884	2044	4798	3363	4056
PLOD3	0.837694	0.702058	0.763002	0.964577	0.891605	8.40E-09	1.33E-05	3.08E-09	3.63E-09	1.42E-07	2885	4106	2791	2615	3347
LMNB1	1.097138	0.955123	1.200201	1.201114	1.579176	8.41E-09	1.15E-08	1.39E-10	3.04E-13	1.93E-14	2886	2576	2267	1361	1052
PAFAH1B1	-0.98419	-1.02173	-0.49967	-0.93049	-0.60941	8.42E-09	5.41E-10	0.000362	6.53E-08	1.51E-05	2887	2079	5638	3082	4400
SEMA4G	1.432305	1.634877	1.686723	1.232046	1.400631	8.44E-09	7.78E-09	2.97E-09	1.32E-07	2.15E-09	2888	2513	2784	3215	2552
TFDP1	1.221047	1.281137	1.218328	1.107514	1.660926	8.49E-09	3.85E-09	9.42E-10	2.15E-09	5.17E-14	2889	2393	2568	2526	1159
C2CD2	-0.92765	-0.64164	-0.87782	-0.64173	-0.96322	8.56E-09	0.000116	7.67E-09	7.68E-05	7.12E-09	2890	4736	2956	4796	2741
GIPC2	1.169119	1.073009	0.983671	1.132285	1.183978	8.60E-09	1.46E-10	4.55E-08	1.45E-08	1.34E-10	2891	1886	3353	2824	2095
IPO13	-0.59994	-0.48536	-0.53624	-0.38302	-0.531	8.69E-09	1.17E-05	4.40E-07	0.001034	1.01E-05	2892	4073	3839	5640	4277
EGR3	-1.05678	-0.78685	-1.21753	-0.84794	-1.38161	8.69E-09	1.55E-06	3.20E-15	1.96E-07	2.63E-13	2893	3591	1031	3298	1323
UBE2T	1.189134	1.246973	1.480325	1.139025	1.70149	8.72E-09	3.37E-11	7.15E-15	9.62E-11	1.49E-16	2894	1682	1095	2061	671
PANX1	-0.91198	-0.8787	-1.17515	-0.97139	-0.87492	8.74E-09	1.62E-06	1.10E-11	4.06E-07	3.00E-05	2895	3602	1886	3449	4565
KIF22	1.128256	1.336439	0.869666	1.225667	0.816535	8.86E-09	4.95E-11	4.61E-07	2.98E-09	3.89E-06	2896	1753	3848	2583	4043
NSD2	1.093563	0.819336	1.147762	0.823619	1.106144	8.88E-09	9.94E-07	1.14E-10	1.57E-07	2.01E-10	2897	3476	2232	3251	2155
CCDC32	-0.5606	-0.46307	-0.66737	-0.47624	-0.56176	8.94E-09	3.55E-06	5.98E-09	6.56E-06	1.74E-07	2898	3789	2912	4087	3385
PHLDA1	-2.28662	-2.68942	-2.31172	-2.47451	-1.84444	9.00E-09	7.45E-12	1.40E-09	5.79E-10	5.64E-05	2900	1497	2636	2300	4750
RPS13	1.735458	1.645699	1.519403	1.893135	1.509614	9.09E-09	1.71E-09	5.15E-07	4.08E-14	7.18E-08	2901	2250	3875	1165	3210
ZNF10	-0.75009	-0.3949	-0.73626	-0.39672	-0.78063	9.10E-09	0.001159	4.55E-08	0.00024	4.46E-08	2902	5566	3354	5127	3103
P3H3	-1.7201	-1.49565	-1.50315	-1.59257	-2.26681	9.19E-09	6.98E-07	4.46E-07	1.56E-07	8.05E-12	2903	3389	3843	3249	1732
MARVELD:	1.01085	1.101907	1.043966	1.063039	0.976708	9.25E-09	1.14E-10	1.08E-11	2.34E-10	3.71E-11	2904	1846	1883	2171	1923
SLC2A8	1.180045	1.241535	0.803717	1.282401	0.699229	9.29E-09	3.53E-10	8.17E-07	1.06E-10	7.82E-05	2905	2022	3988	2069	4848
THOP1	0.92634	0.997928	0.806434	1.071165	1.016033	9.30E-09	6.04E-07	4.08E-06	9.11E-09	5.40E-10	2906	3354	4354	2744	2316
FAM168A	-0.94414	-0.85386	-0.65933	-0.75893	-1.08133	9.36E-09	7.49E-09	6.74E-06	3.32E-06	1.04E-08	2907	2502	4477	3917	2825
KRTAP10-:	-0.87898	-0.53707	-1.00395	-0.79632	-0.56257	9.47E-09	4.32E-05	1.20E-10	5.06E-07	7.53E-05	2908	4440	2245	3496	4839
PURA	-0.64195	-0.56472	-0.51373	-0.54672	-0.45775	9.52E-09	2.84E-07	1.78E-06	1.10E-05	5.79E-06	2909	3207	4167	4241	4144
EMC10	0.89036	0.702519	0.975913	0.870466	0.980024	9.52E-09	3.58E-05	8.66E-08	1.86E-06	8.32E-10	2910	4376	3477	3788	2399
VCX3A	-0.85105	-0.42071	-0.91856	-0.65791	-0.8554	9.60E-09	0.000397	7.89E-09	9.12E-06	4.11E-09	2911	5169	2965	4183	2656
KCTD9	-0.95775	-0.86179	-0.88299	-0.85321	-0.68077	9.61E-09	8.46E-09	4.03E-12	5.43E-08	4.94E-05	2912	2528	1753	3047	4717
SEMA4A	-1.08132	-1.09562	-1.59162	-1.4533	-1.48781	9.64E-09	6.61E-09	9.25E-12	2.03E-08	8.24E-11	2913	2490	1861	2889	2020
PTMA	1.463686	1.437758	1.807739	1.554599	1.752915	9.64E-09	1.14E-08	4.68E-11	2.57E-09	6.60E-13	2914	2575	2097	2558	1437
NOL7	1.086536	0.969155	1.28979	1.064243	0.90982	9.68E-09	1.96E-09	4.81E-11	2.16E-09	5.69E-08	2915	2273	2103	2527	3149
PRMT5	0.816422	0.673749	0.811516	0.559352	0.583807	9.68E-09	1.40E-05	2.50E-09	3.09E-05	0.000201	2916	4112	2755	4527	5124
IL11	-1.95174	-1.85238	-2.11263	-2.51795	-2.35573	9.70E-09	9.13E-05	5.83E-07	2.46E-06	8.74E-08	2917	4675	3899	3849	3249
CCNA2	1.121029	1.213073	1.231006	1.208425	1.146504	9.70E-09	2.65E-11	2.74E-13	6.17E-13	3.09E-11	2918	1657	1453	1447	1896
HNRNPR	0.989149	0.879091	1.066883	0.952599	0.489766	9.79E-09	3.30E-06	1.05E-09	1.49E-06	0.007193	2919	3779	2585	3731	6385
E2F6	-0.66891	-0.29954	-0.51739	-0.45414	-0.44317	9.84E-09	0.00062	9.51E-07	1.78E-05	2.16E-05	2920	5335	4024	4369	4485
CRYBG1	1.36383	1.549056	1.692154	1.60375	1.368157	9.93E-09	2.91E-10	4.99E-16	8.95E-11	3.70E-12	2921	1978	876	2055	1635
DDX39B	0.967064	0.806448	0.884943	0.760334	1.157018	9.93E-09	1.69E-06	1.00E-06	1.14E-06	6.15E-11	2922	3615	4038	3673	1975
PUS7	0.954695	0.841676	0.733632	0.859537	0.807378	9.94E-09	1.03E-06	1.73E-06	6.22E-08	7.62E-09	2923	3484	4159	3074	2757
CYB5R3	-1.41013	-1.39299	-0.91018	-1.1459	-1.32187	9.98E-09	3.09E-13	9.23E-07	1.42E-09	3.14E-08	2924	1135	4016	2463	3029
MARVELD	-1.47847	-1.3527	-1.2526	-1.22793	-1.51749	1.01E-08	2.69E-09	5.29E-10	1.44E-08	1.05E-10	2925	2325	2470	2823	2060
SNCA	-1.07276	-0.69452	-0.71229	-0.70768	-1.02262	1.01E-08	0.00043	6.07E-05	0.000235	1.59E-08	2926	5198	5085	5117	2902
TIMM17B	0.897429	1.006906	0.594345	0.982074	0.905467	1.01E-08	7.65E-13	4.27E-08	1.21E-11	6.48E-10	2927	1229	3340	1795	2353
POLR2C	0.709115	0.665576	0.632713	0.609798	0.641329	1.02E-08	9.12E-07	1.10E-09	5.23E-07	7.95E-08	2928	3456	2597	3503	3227
OR5L1	-0.88798	-0.52873	-1.09869	-0.73923	-1.00973	1.02E-08	0.000389	6.02E-10	8.93E-06	6.94E-08	2929	5162	2498	4171	3195
GGCT	1.080254	1.2292	0.988702	1.180369	1.416607	1.02E-08	7.47E-11	1.09E-09	4.03E-10	1.28E-13	2930	1788	2591	2248	1248
SLC6A17	-0.75392	-0.57762	-0.9445	-0.92737	-0.78654	1.03E-08	2.49E-05	1.91E-08	7.98E-08	5.12E-06	2931	4274	3161	3122	4114
EIF4A3	1.360209	1.271739	1.858777	1.3653	1.027826	1.03E-08	1.25E-09	6.56E-12	1.66E-09	1.00E-07	2932	2204	1813	2489	3276
GAGE10	-0.88224	-0.7129	-1.18474	-0.64962	-0.99473	1.04E-08	2.03E-08	1.46E-11	1.85E-05	2.10E-09	2933	2667	1919	4379	2548
YTHDC2	-0.76634	-0.61329	-0.93014	-0.79242	-0.86763	1.04E-08	0.000468	1.37E-09	3.44E-06	1.06E-09	2934	5222	2631	3925	2437
POLD2	1.206602	1.345993	1.385266	1.263179	1.619054	1.04E-08	4.90E-11	2.53E-11	1.36E-10	7.89E-17	2935	1750	2012	2098	624
ATP6V1C1	-0.7729	-0.51528	-0.34525	-0.52133	-0.6295	1.05E-08	0.000139	0.005368	4.14E-05	2.30E-06	2936	4791	6606	4618	3925
OR2A14	-0.61585	-0.47058	-0.95336	-0.80982	-0.74925	1.06E-08	7.82E-07	1.07E-07	4.25E-07	2.32E-05	2937	3415	3532	3457	4501
RBM39	1.393714	1.234152	0.882526	1.488087	1.040254	1.07E-08	5.79E-08	1.43E-05	1.32E-09	7.85E-08	2939	2874	4693	2441	3225
ANAPC11	0.843251	0.763524	0.89137	0.850912	0.875919	1.07E-08	2.95E-07	3.29E-09	3.10E-08	6.40E-09	2940	3214	2808	2956	2721
RAB33B	-0.72541	-0.46521	-0.75829	-0.63162	-0.93934	1.08E-08	8.21E-05	1.04E-09	1.18E-06	5.65E-11	2941	4639	2584	3688	1961
PSMD4	0.671442	0.573966	0.543865	0.627379	0.65586	1.08E-08	2.79E-06	1.04E-06	2.38E-06	3.97E-07	2942	3742	4047	3841	3564
UPP1	-0.96414	-0.83493	-1.1454	-1.08238	-1.1439	1.09E-08	9.90E-06	2.56E-07	9.39E-08	2.45E-08	2943	4028	3710	3155	2978
NFE2L3	1.397852	1.581405	0.991428	1.388081	1.527565	1.09E-08	2.24E-11	2.31E-07	5.01E-10	8.98E-12	2945	1629	3688	2280	1748
CDHR2	1.843511	1.369098	1.542951	1.511076	1.184008	1.09E-08	4.65E-07	3.69E-08	5.92E-09	1.85E-05	2946	3300	3312	2681	4448
AMDHD2	-0.77895	-0.75825	-0.72526	-0.72046	-0.6672	1.10E-08	2.96E-09	2.05E-10	2.81E-07	1.61E-05	2947	2345	2325	3365	4417
MRPL20	0.59145	0.65962	0.79351	0.751396	0.61818	1.10E-08	5.64E-07	2.28E-07	3.69E-09	7.34E-06	2948	3338	3684	2622	4200
B4GAT1	-0.80397	-0.67382	-0.94607	-0.67801	-1.0739	1.11E-08	5.82E-06	4.24E-10	1.25E-06	8.32E-12	2949	3897	2440	3696	1735
SHMT2	1.272945	1.105276	1.006708	1.035871	1.190345	1.11E-08	5.41E-07	9.11E-06	6.81E-09	9.47E-09	2950	3325	4565	2701	2803
CRTC3	-0.77264	-0.72455	-0.79449	-0.87991	-0.84128	1.12E-08	5.32E-08	1.11E-09	3.49E-10	7.26E-11	2951	2856	2599	2227	2010
SGSH	-0.56353	-0.36502	-0.52439	-0.46466	-0.5057	1.13E-08	8.36E-05	1.30E-07	1.61E-06	3.66E-09	2952	4643	3570	3748	2635
NEK2	0.780103	0.872261	0.793227	0.545805	0.423189	1.14E-08	3.03E-09	4.16E-08	5.63E-05	0.006442	2953	2351	3334	4713	6339

LSM2	0.774769	0.727246	0.616668	0.804614	0.798461	1.14E-08	6.71E-07	3.82E-06	1.05E-10	7.50E-09	2954	3378	4328	2067	2754
GNLY	-1.13911	-1.18761	-1.03452	-1.56431	-0.98691	1.14E-08	2.02E-09	1.45E-06	5.26E-15	1.47E-06	2955	2277	4119	988	3816
PF4	-0.85244	-0.65535	-0.86685	-0.75335	-0.89082	1.15E-08	2.18E-07	2.22E-08	7.95E-07	4.00E-08	2956	3164	3183	3589	3073
ANGPTL4	-1.41876	-1.65967	-1.3188	-1.86616	-1.54616	1.15E-08	9.71E-08	1.34E-07	2.15E-08	1.74E-09	2957	2985	3577	2903	2515
TGM2	-1.76716	-2.01338	-1.81436	-1.37354	-1.77777	1.16E-08	1.17E-08	1.03E-08	2.30E-05	5.11E-08	2958	2582	3018	4424	3131
CHIC2	-0.99785	-0.90099	-0.68512	-1.1653	-0.89237	1.16E-08	2.13E-06	0.000266	1.02E-07	3.29E-07	2959	3677	5540	3169	3523
FUS	0.77842	0.848126	0.693274	0.867982	1.202739	1.17E-08	3.98E-09	8.17E-07	1.82E-09	4.57E-11	2961	2399	3987	2502	1945
STRADA	-0.89426	-0.8397	-0.62181	-0.74016	-1.09215	1.18E-08	2.37E-07	6.69E-06	8.46E-06	7.03E-14	2962	3179	4476	4161	1190
SP100	-0.90604	-0.82488	-1.01112	-0.84975	-0.85748	1.19E-08	1.87E-09	2.27E-11	4.81E-11	2.55E-07	2963	2264	1996	1970	3462
CREBL2	-0.74526	-0.92605	-1.008	-1.0151	-0.79335	1.19E-08	1.20E-09	1.04E-12	2.16E-09	7.16E-08	2964	2199	1601	2528	3208
ZNF214	-0.63307	-0.40356	-0.5756	-0.54001	-0.81219	1.19E-08	0.000241	3.06E-07	3.89E-06	1.16E-07	2965	4974	3765	3958	3308
OR4K5	-0.80374	-0.64064	-0.95727	-1.01451	-1.12229	1.20E-08	7.97E-06	1.55E-06	3.63E-09	2.49E-09	2966	3968	4135	2616	2583
MYO15B	1.090116	1.274433	1.094927	1.320373	1.173355	1.20E-08	3.59E-09	1.43E-08	2.34E-09	7.57E-08	2967	2382	3098	2542	3221
H3C7	2.060812	1.408807	2.003673	1.855824	2.171353	1.22E-08	1.73E-05	1.07E-08	1.34E-08	5.16E-10	2968	4170	3025	2815	2313
KLF16	0.700985	0.874054	0.634807	0.925032	0.755321	1.22E-08	3.30E-09	4.02E-07	4.94E-12	1.81E-07	2969	2368	3822	1675	3390
ZSWIM8	-1.06665	-0.81596	-0.74789	-0.90015	-0.87423	1.22E-08	7.12E-06	1.34E-05	1.55E-07	1.06E-07	2970	3940	4675	3248	3289
BDH2	-1.0179	-1.12526	-1.10961	-1.01723	-0.93493	1.22E-08	1.07E-06	5.14E-07	7.68E-06	8.82E-07	2971	3498	3872	4135	3707
GNL3	1.016705	1.377034	1.061104	1.353713	1.350944	1.23E-08	2.53E-11	7.14E-08	1.28E-14	5.24E-12	2973	1653	3445	1060	1678
PRIMPOL	-0.65683	-0.49441	-0.80058	-0.87199	-0.62149	1.23E-08	4.59E-05	7.62E-06	1.29E-07	7.32E-06	2974	4452	4514	3210	4198
PRPF39	-0.60417	-0.38418	-0.61706	-0.56043	-0.45913	1.24E-08	0.000783	1.41E-07	9.82E-06	2.33E-05	2975	5414	3594	4206	4503
PHB2	1.171349	1.075657	0.959108	1.19076	1.186169	1.24E-08	8.17E-08	4.63E-06	1.27E-10	4.62E-08	2976	2951	4386	2093	3109
MTHFD1	1.0889	1.065378	1.262099	1.091406	1.105715	1.24E-08	5.90E-08	3.05E-09	2.50E-10	2.15E-07	2977	2876	2790	2178	3429
DNMBP	0.795962	0.757958	0.702459	0.866753	0.682634	1.24E-08	4.06E-11	2.04E-06	2.19E-09	3.51E-05	2978	1716	4185	2531	4615
PFKFB4	-1.60617	-1.29412	-1.04423	-1.08853	-1.45453	1.25E-08	6.36E-05	8.70E-05	0.000539	7.77E-07	2979	4549	5189	5411	3687
WDR45B	0.909364	0.867625	1.181176	1.126958	0.824635	1.28E-08	4.18E-09	2.69E-08	2.72E-09	1.26E-05	2980	2408	3230	2562	4354
RPS8	1.65016	1.494229	1.310842	1.797072	1.63278	1.28E-08	9.47E-09	9.06E-06	2.29E-13	2.79E-09	2981	2545	4562	1328	2590
MRPL9	0.764212	0.796846	0.519797	0.752616	0.575851	1.29E-08	7.23E-08	1.55E-05	2.63E-09	0.000284	2982	2920	4713	2559	5222
BDKRB2	-1.97746	-1.70777	-1.82309	-2.01197	-2.33461	1.29E-08	3.43E-06	3.04E-07	1.96E-07	4.91E-09	2983	3784	3762	3300	2678
PLEKHA7	0.84898	1.117857	1.179882	0.936043	1.198646	1.32E-08	1.17E-12	1.61E-13	5.77E-11	4.02E-15	2985	1262	1387	1993	898
RPS27A	1.712999	1.762246	1.662741	1.848708	1.956082	1.33E-08	1.99E-10	3.02E-08	6.05E-12	1.90E-13	2986	1924	3259	1693	1288
H2AC16	1.66961	1.630436	1.938746	1.848207	2.083019	1.34E-08	1.09E-08	3.71E-11	5.20E-11	2.96E-13	2987	2565	2070	1981	1335
DPEP1	3.063006	3.230294	1.96449	3.327573	2.970108	1.34E-08	5.28E-12	5.76E-05	3.12E-11	5.72E-10	2988	1447	5069	1914	2328
ACAD9	0.667217	0.849668	0.666321	0.751997	0.744687	1.35E-08	4.39E-10	2.56E-09	1.40E-10	3.82E-10	2989	2050	2759	2103	2255
C1QL4	-0.48256	-0.38594	-0.66525	-0.51786	-0.95786	1.36E-08	0.000218	1.61E-05	0.000243	6.63E-08	2990	4936	4721	5133	3178
ABL2	-0.82642	-0.84236	-0.70894	-0.88491	-0.88449	1.36E-08	1.69E-08	1.35E-07	1.93E-07	1.56E-09	2991	2637	3580	3293	2499
PDIA3	1.16682	0.559487	1.092487	0.908471	0.999929	1.37E-08	0.000741	1.68E-06	1.00E-06	2.06E-06	2992	5392	4151	3643	3882
IDO1	-1.40232	-1.24563	-1.49237	-1.31366	-1.67875	1.37E-08	8.36E-06	1.31E-08	5.06E-07	1.35E-08	2993	3982	3077	3497	2879
DIPK1B	-0.8525	-0.4468	-0.72352	-0.73773	-1.12115	1.37E-08	0.001186	1.35E-06	1.40E-05	7.38E-09	2994	5581	4109	4305	2748
E2F4	0.895871	0.763973	0.767745	0.837096	0.831494	1.37E-08	5.67E-08	1.58E-08	9.71E-09	2.92E-07	2995	2869	3113	2758	3496
TMC4	1.256974	1.222669	1.622129	1.033969	1.524803	1.39E-08	3.87E-07	3.44E-12	5.92E-08	4.49E-11	2996	3266	1738	3060	1944
GLYR1	0.66887	0.53542	0.613644	0.648016	0.733772	1.39E-08	2.36E-06	2.43E-08	1.77E-07	2.40E-08	2997	3700	3202	3274	2974
OR52N2	-0.86228	-0.94432	-0.90482	-1.21854	-1.05431	1.39E-08	7.22E-08	5.88E-09	1.02E-11	6.23E-11	2998	2919	2907	1761	1978
ADRA2B	-0.6933	-0.57888	-0.86908	-0.81872	-0.60239	1.40E-08	6.79E-05	9.15E-08	9.85E-09	0.000246	2999	4579	3490	2763	5183
STX1B	-0.73107	-0.5988	-1.01937	-0.92757	-0.80501	1.41E-08	4.02E-05	5.29E-09	2.98E-08	3.74E-05	3000	4418	2885	2950	4632
THAP6	-0.69728	-0.46158	-0.62339	-0.62764	-0.61675	1.42E-08	9.66E-06	3.20E-08	9.95E-07	5.37E-06	3002	4023	3272	3640	4128
C18orf32	-0.74677	-0.75352	-0.52903	-0.58135	-0.73836	1.42E-08	4.39E-07	3.89E-05	3.28E-05	2.16E-07	3003	3285	4960	4548	3430
STIP1	1.003703	1.090138	1.083431	1.270261	0.964241	1.42E-08	8.13E-11	2.02E-09	1.12E-12	2.18E-09	3004	1796	2706	1520	2555
VPS11	-0.61244	-0.422	-0.54625	-0.47986	-0.68438	1.43E-08	3.78E-05	1.75E-07	1.08E-05	3.26E-08	3005	4397	3633	4238	3034
H2BC11	1.818188	1.612674	2.106187	1.988952	2.062344	1.44E-08	1.11E-08	1.76E-10	4.36E-12	1.15E-12	3006	2568	2306	1665	1501
ABLIM1	0.850734	0.8284	1.127191	0.910254	1.016565	1.44E-08	6.52E-07	8.05E-11	2.12E-07	4.93E-09	3007	3370	2173	3309	2680
PRRC2B	0.592954	0.408639	0.802717	0.62617	0.468773	1.45E-08	4.75E-05	8.98E-07	6.76E-06	0.001159	3008	4468	4008	4095	5688
ZIC3	-0.83307	-0.65925	-1.21887	-0.76692	-0.69088	1.46E-08	6.89E-07	8.01E-13	7.53E-07	6.44E-05	3009	3387	1564	3579	4794
GSN	-1.99885	-2.09026	-1.40087	-1.93147	-1.65196	1.47E-08	6.76E-12	8.79E-07	1.76E-11	2.25E-07	3010	1479	4004	1844	3446
ERC1	-0.76164	-0.59282	-0.52309	-0.56853	-0.86825	1.47E-08	0.000154	0.000109	0.000666	3.73E-09	3011	4822	5245	5479	2641
RPL9	1.575494	1.338838	1.462576	1.435155	1.550169	1.47E-08	4.86E-08	1.38E-06	5.01E-11	7.05E-11	3012	2843	4113	1977	2007
DIAPH1	1.265991	1.209624	1.62838	1.259149	1.321152	1.49E-08	1.28E-07	4.85E-15	1.79E-08	1.17E-11	3013	3051	1063	2866	1787
INPP5D	-0.85779	-0.48429	-1.0105	-0.58285	-0.7131	1.51E-08	0.00603	7.79E-07	0.007085	7.54E-05	3014	6187	3968	6389	4841
DIPK1A	-0.74634	-0.39205	-0.6447	-0.74212	-0.87637	1.52E-08	0.000251	2.11E-07	4.07E-09	4.19E-09	3016	4991	3671	2633	2660
SLC25A6	1.605707	1.456462	1.459422	1.894972	1.688498	1.52E-08	3.00E-09	7.70E-08	1.21E-13	3.19E-11	3017	2347	3454	1258	1902
UQCRFS1	0.87732	0.941195	1.44164	1.026739	1.158109	1.53E-08	4.64E-08	1.59E-15	2.70E-08	3.56E-11	3018	2834	974	2933	1917
IL1B	-2.19262	-2.59029	-2.384	-2.75714	-2.81082	1.53E-08	8.68E-08	4.23E-07	1.58E-08	2.17E-08	3019	2964	3832	2841	2958
XPOT	1.088499	1.01088	1.06058	1.085321	0.902102	1.54E-08	7.48E-07	1.92E-07	7.57E-08	5.61E-07	3020	3404	3653	3105	3622
GCN1	0.775968	0.982741	0.690635	0.920724	1.073449	1.54E-08	5.45E-10	5.99E-07	2.43E-11	4.83E-10	3021	2082	3911	1891	2300
PLK1	1.247699	1.145565	0.85107	1.044805	0.667982	1.54E-08	1.87E-07	2.12E-05	3.82E-07	0.000918	3022	3135	4791	3434	5603
DDX18	0.826754	0.823574	0.725583	0.654759	0.851505	1.55E-08	1.36E-06	5.10E-06	1.07E-05	2.19E-08	3023	3556	4413	4233	2961
DNAJA1	0.736602	0.754666	1.036094	0.818804	0.53071	1.57E-08	1.00E-07	2.78E-10	3.22E-06	3.87E-05	3024	2995	2373	3909	4649
MED13L	-0.65896	-0.54841	-0.52833	-0.43538	-0.42371	1.57E-08	1.44E-07	1.27E-06	5.42E-05	0.000667	3025	3077	4099	4694	5504
PARVA	-1.43441	-1.5975	-1.22276	-1.39061	-1.19326	1.58E-08	6.72E-11	8.49E-08	1.87E-10	2.26E-06	3026	1780	3474	2139	3915
NTMT1	0.795105	1.011754	0.911658	1.08535	0.807645	1.58E-08	1.94E-09	1.33E-09	7.25E-11	1.79E-09	3027	2269	2629	2028	2523
RPS27	1.500335	1.307172	1.171304	1.444634	1.512356	1.59E-08	2.52E-08	4.92E-07	2.99E-09	7.45E-09	3028	2703	3863	2585	2752
NIPA1	-0.64665	-0.43328	-0.56174	-0.50786	-0.53311	1.59E-08	0.002862	1.12E-05	0.000442	1.68E-07	3029	5878	4623	5333	3379
UBA2	0.901155	0.747059	1.026478	0.71903	0.920307	1.60E-08	5.27E-07	3.56E-09	2.76E-06	1.17E-12	3030	3318	2821	3877	1504
INSR	-1.31984	-1.2205	-1.41965	-0.77489	-0.94141	1.60E-08	2.21E-06	2.50E-09	0.000213	0.005644	3031	3686	2754	5085	6292

PAFAH1B3	1.054052	1.229176	1.098257	1.065668	0.79326	1.61E-08	9.34E-11	8.87E-11	4.91E-08	0.000155	3032	1819	2194	3034	5052
IFNA4	-0.75276	-0.50769	-0.76121	-0.72915	-1.20388	1.61E-08	4.23E-05	6.87E-09	1.68E-06	2.05E-13	3033	4436	2936	3753	1300
PSMA1	0.936452	0.807363	0.988289	0.884783	0.803014	1.62E-08	1.14E-07	4.11E-10	1.51E-08	7.70E-09	3034	3023	2435	2833	2760
KLRC2	-0.7591	-0.45532	-0.80465	-0.53805	-0.5219	1.64E-08	1.14E-05	6.01E-09	3.46E-05	1.81E-05	3035	4063	2913	4563	4442
TEAD2	-1.47588	-0.94641	-0.98817	-0.93595	-1.10007	1.68E-08	0.000374	5.80E-05	0.000134	9.30E-06	3036	5149	5070	4949	4261
SGPP2	1.224923	1.331633	1.859842	1.41662	1.142301	1.72E-08	4.26E-10	7.11E-14	2.64E-10	6.52E-07	3037	2047	1282	2186	3657
ETF1	0.704767	0.599174	0.777647	0.575946	0.579638	1.73E-08	8.27E-08	1.68E-11	6.86E-08	1.68E-07	3038	2953	1936	3089	3381
POGLUT3	-0.7941	-0.73264	-0.97985	-0.82912	-1.1392	1.74E-08	3.20E-06	1.74E-10	5.92E-08	4.04E-11	3039	3772	2303	3061	1927
RPS3A	1.616591	1.34164	1.370077	1.562307	1.70802	1.74E-08	9.47E-08	9.82E-07	4.94E-10	1.82E-10	3040	2981	4031	2275	2138
TREX1	-0.71298	-0.5689	-0.56579	-0.54785	-0.59471	1.75E-08	9.73E-06	1.13E-05	2.56E-06	3.46E-06	3041	4026	4624	3855	4018
OR4K15	-0.91297	-0.73865	-0.98798	-1.15874	-0.79444	1.77E-08	3.62E-07	5.32E-08	1.58E-09	4.44E-06	3042	3253	3388	2480	4071
KCNAB2	-0.87864	-0.7775	-1.06703	-0.74339	-1.00341	1.77E-08	8.66E-08	5.58E-10	1.45E-05	5.19E-08	3044	2962	2480	4315	3135
TSPO	0.872948	0.989191	1.258719	0.983954	0.875224	1.77E-08	7.61E-15	3.56E-17	4.49E-15	1.31E-07	3045	821	694	966	3331
CDC6	0.864304	0.919072	0.91842	0.869417	0.462698	1.79E-08	1.22E-09	4.20E-09	9.36E-09	0.005443	3047	2201	2849	2749	6269
KDM5D	-1.23794	-1.23873	-1.31165	-1.25046	-0.99541	1.80E-08	4.88E-09	2.27E-09	3.55E-12	1.43E-08	3048	2437	2731	1639	2886
GADD45A	-0.77136	-0.45531	-0.87709	-0.60857	-0.67587	1.85E-08	0.000864	1.09E-08	7.10E-05	1.14E-05	3049	5454	3029	4773	4316
LUZP1	-0.91271	-1.0211	-0.76386	-1.12464	-0.96005	1.87E-08	1.19E-08	7.15E-06	2.80E-10	1.73E-09	3050	2584	4495	2197	2513
ZMYND11	-0.71384	-0.5587	-0.54189	-0.63213	-0.61033	1.87E-08	1.84E-05	6.77E-06	6.48E-06	3.48E-06	3051	4190	4482	4084	4019
POLE4	0.637763	0.74816	0.666935	0.618513	0.516273	1.87E-08	1.56E-06	9.90E-06	1.26E-06	0.002274	3052	3593	4586	3698	5953
KRTAP19-:	-0.76363	-0.50816	-0.73726	-0.70582	-0.73914	1.87E-08	9.74E-05	3.74E-06	6.76E-06	1.03E-05	3053	4693	4324	4096	4283
UAP1L1	-0.92648	-0.6642	-0.76963	-0.65369	-1.13226	1.87E-08	0.000231	1.93E-06	0.000129	1.47E-10	3054	4959	4179	4936	2108
CFL1	1.226199	0.846624	1.453514	1.172544	1.089015	1.87E-08	2.22E-07	1.13E-09	1.80E-08	7.22E-09	3055	3170	2605	2867	2744
UQCC2	0.804231	0.796906	0.821179	0.712635	0.646063	1.89E-08	2.03E-08	9.17E-08	1.13E-07	1.70E-05	3057	2668	3492	3191	4430
UTP14C	0.822135	0.732759	0.644144	0.594673	0.556574	1.89E-08	2.34E-07	4.42E-07	1.84E-06	0.000223	3058	3178	3840	3781	5146
CHI3L1	-2.39432	-2.15811	-3.32144	-2.63587	-3.53534	1.89E-08	3.29E-08	7.41E-12	9.23E-08	9.58E-12	3059	2752	1834	3152	1756
OBSL1	-1.51813	-1.1229	-1.30722	-1.24082	-1.27014	1.89E-08	8.03E-07	6.78E-07	2.48E-07	5.38E-07	3061	3425	3938	3340	3617
MAP3K8	-0.75372	-0.73857	-0.73789	-0.73924	-0.91915	1.89E-08	9.49E-07	1.15E-08	9.01E-07	1.28E-09	3062	3470	3046	3611	2463
ENO1	1.413714	1.071546	1.703012	1.300553	1.518241	1.89E-08	7.69E-09	1.80E-11	1.02E-07	2.44E-12	3063	2508	1953	3170	1587
ITGA10	-0.71116	-0.60617	-0.79448	-0.67243	-1.10325	1.91E-08	5.86E-05	2.79E-08	8.76E-06	6.16E-12	3064	4531	3239	4165	1694
ANKRD36	-0.8538	-0.31923	-0.86735	-0.5151	-0.65653	1.91E-08	0.006708	7.13E-09	0.000287	2.31E-06	3065	6232	2946	5202	3927
POLR3K	0.934361	0.926124	0.795212	0.885583	0.700584	1.92E-08	1.63E-07	9.22E-07	3.28E-08	4.22E-05	3066	3097	4015	2969	4672
RRAS	-0.86672	-0.81296	-0.77507	-1.02953	-0.88903	1.93E-08	2.18E-08	4.15E-07	1.71E-13	7.98E-09	3067	2680	3829	1298	2766
CD200	-1.20264	-0.97513	-1.1178	-1.10751	-1.35919	1.95E-08	1.75E-05	6.59E-05	6.46E-07	8.57E-09	3068	4173	5108	3557	2780
C6orf132	1.032498	0.92558	1.129782	0.769306	1.164244	1.96E-08	1.44E-06	1.12E-10	3.05E-07	3.81E-12	3069	3575	2231	3381	1638
HMBOX1	-0.98398	-0.48033	-0.84117	-0.67135	-0.76409	1.97E-08	0.001173	1.28E-08	8.01E-05	4.89E-07	3070	5576	3073	4802	3599
RAB24	-0.9446	-0.73876	-0.81901	-0.92606	-0.78102	1.97E-08	4.42E-06	1.63E-07	3.16E-09	2.19E-08	3071	3834	3620	2597	2960
RUVBL1	1.118403	1.113706	0.897899	0.992905	1.0276	1.97E-08	5.84E-09	2.79E-08	2.65E-08	2.74E-08	3072	2469	3238	2931	3001
TOM1	-0.9123	-0.97766	-0.6871	-0.91054	-1.00863	1.99E-08	2.07E-10	4.07E-07	1.66E-09	4.50E-10	3073	1931	3824	2488	2291
GSDMB	1.179001	1.403733	1.451189	1.409469	1.100771	1.99E-08	7.52E-09	1.33E-09	4.90E-13	2.10E-06	3074	2504	2628	1421	3886
NBPF15	-0.73633	-0.43064	-0.97666	-0.66359	-0.6029	2.00E-08	8.54E-05	8.78E-12	1.01E-07	1.53E-06	3075	4650	1853	3166	3828
RPL10A	1.786676	1.621765	1.490278	1.835747	1.90038	2.01E-08	4.71E-09	1.61E-06	2.45E-13	2.44E-11	3076	2425	4141	1339	1875
H2BC18	1.330171	1.221665	1.534188	1.402404	1.330891	2.01E-08	6.62E-10	2.27E-09	7.81E-13	4.71E-09	3077	2104	2730	1470	2676
SMCHD1	-0.73862	-0.82288	-1.08128	-0.88675	-1.05677	2.03E-08	4.02E-10	2.49E-12	2.04E-08	3.56E-11	3078	2039	1691	2890	1916
MOB1B	-0.96793	-0.47413	-0.78241	-0.62714	-1.10904	2.03E-08	0.004454	6.97E-07	0.000421	9.13E-11	3079	6056	3942	5321	2038
H2AZ1	1.341113	1.146094	1.323194	1.240695	1.55411	2.07E-08	2.54E-08	3.00E-08	5.00E-10	3.99E-12	3080	2707	3257	2279	1646
FAM234A	0.881727	0.741983	0.865878	0.86661	0.883669	2.10E-08	4.15E-05	3.45E-07	1.05E-06	1.84E-06	3081	4425	3786	3651	3864
RPL23	1.481594	1.491324	1.308668	1.497337	1.605245	2.15E-08	2.53E-10	1.20E-10	8.65E-10	1.72E-14	3082	1963	2243	2370	1038
GBE1	-0.9315	-0.76632	-0.56874	-0.94119	-0.99856	2.16E-08	2.19E-05	2.64E-06	1.14E-06	3.75E-07	3083	4240	4250	3676	3558
ANP32B	1.080308	1.143682	1.235927	1.046125	1.228892	2.17E-08	4.39E-08	1.79E-09	2.95E-08	1.22E-11	3084	2822	2673	2948	1792
NDUFB6	0.855204	0.731908	0.815457	0.97809	0.720508	2.19E-08	0.000139	1.20E-05	9.50E-07	2.33E-05	3085	4793	4644	3627	4504
COTL1	-1.52761	-1.74567	-1.61533	-1.86414	-1.57176	2.20E-08	2.58E-09	1.42E-08	2.58E-10	3.84E-08	3086	2319	3096	2182	3061
LARP1	0.940295	0.836851	0.822998	0.841004	0.786737	2.26E-08	4.65E-07	4.23E-06	2.47E-08	8.11E-07	3087	3299	4362	2919	3694
LSM10	-0.6894	-0.6169	-0.76031	-0.65363	-0.84821	2.28E-08	3.33E-07	1.72E-09	3.53E-09	1.10E-09	3088	3237	2663	2612	2442
TSPAN2	-1.62924	-1.08054	-1.09153	-1.13351	-1.70315	2.28E-08	0.000538	0.000117	0.000246	3.68E-09	3089	5286	5268	5137	2637
KCNG2	-0.89771	-0.88372	-0.89313	-1.01233	-0.8593	2.29E-08	2.12E-06	5.39E-09	1.60E-07	2.95E-09	3090	3676	2888	3255	2603
ARAP1	-0.96539	-1.03587	-1.03277	-1.1312	-0.98634	2.29E-08	6.04E-09	2.23E-10	1.21E-10	7.26E-10	3091	2475	2339	2085	2375
PET100	0.777744	0.964159	0.775288	0.999472	0.887987	2.30E-08	2.18E-09	3.18E-08	1.08E-11	1.14E-07	3092	2291	3271	1771	3304
NDUFS5	0.998961	0.791059	1.065399	1.085162	0.923208	2.30E-08	1.01E-06	2.72E-07	1.64E-09	8.56E-09	3093	3480	3726	2487	2779
RAP1GAP2	1.147452	1.039735	0.869429	1.070669	0.965805	2.30E-08	8.07E-06	3.75E-06	9.76E-07	1.49E-06	3094	3972	4325	3637	3817
PPP2R2B	-0.70547	-0.57333	-0.87324	-0.81316	-0.86903	2.30E-08	8.55E-06	2.25E-08	1.78E-07	1.06E-08	3095	3992	3186	3276	2828
CFDP1	0.961368	0.676119	0.860183	0.772203	0.897064	2.31E-08	2.41E-05	3.41E-07	1.34E-08	1.56E-09	3096	4266	3784	2814	2500
CEP72	1.07636	1.125124	1.051853	1.018979	0.89185	2.32E-08	1.40E-08	1.18E-10	1.00E-08	2.59E-08	3097	2616	2240	2765	2985
ACTL6A	0.9248	0.988789	1.043456	1.055442	1.218552	2.34E-08	1.15E-07	2.43E-09	1.38E-08	6.30E-11	3098	3027	2743	2818	1986
BYSL	0.7979	0.830857	0.670505	0.524689	0.651602	2.35E-08	8.04E-08	2.12E-08	4.95E-05	7.90E-05	3099	2945	3178	4672	4849
FANCD2OS	-0.66734	-0.34731	-0.71289	-0.61698	-0.5402	2.35E-08	0.000191	7.00E-07	1.02E-06	2.98E-05	3100	4901	3945	3648	4563
SCARB1	1.00054	1.171533	0.750839	1.044679	1.111845	2.35E-08	1.45E-10	5.98E-07	6.63E-11	2.50E-12	3101	1885	3910	2014	1591
SNU13	0.81971	0.89046	1.098676	1.055272	0.753653	2.35E-08	7.31E-08	3.67E-08	1.22E-10	1.45E-06	3102	2925	3311	2087	3814
FAM13B	-0.94413	-0.55832	-0.88468	-0.72343	-0.785	2.37E-08	0.001213	2.24E-07	1.43E-05	1.90E-08	3103	5588	3681	4312	2931
H1-4	1.674473	1.310352	1.513059	1.576153	1.700485	2.40E-08	3.69E-07	9.74E-08	3.33E-10	3.41E-10	3104	3259	3508	2220	2238
VEGFA	1.454747	1.355169	1.46401	1.241641	1.373702	2.40E-08	8.83E-08	4.68E-11	3.17E-07	2.75E-10	3105	2969	2098	3391	2215
MRPL24	0.644853	0.655519	0.791675	0.533052	0.93301	2.41E-08	1.33E-06	6.54E-08	7.51E-07	2.52E-09	3106	3553	3433	3578	2584
KCNK9	-0.8879	-0.49923	-0.90482	-0.62793	-0.48208	2.41E-08	0.000473	2.44E-09	0.000152	0.003348	3107	5224	2745	4977	6065
MLLT6	0.967474	1.037174	0.922187	0.948803	0.978337	2.42E-08	2.31E-09	2.64E-10	2.10E-08	9.19E-11	3108	2299	2362	2897	2039

THBS4	-0.70764	-0.64759	-0.69959	-0.92144	-0.51396	2.42E-08	0.000209	5.07E-05	3.35E-08	0.000224	3109	4920	5043	2978	5150
MRPL52	0.90096	0.766336	0.934466	0.654319	0.633675	2.45E-08	8.82E-07	3.49E-08	2.46E-05	0.001627	3110	3447	3302	4446	5828
YJEFN3	-0.68833	-0.38229	-0.37769	-0.40392	-0.38497	2.46E-08	0.004022	0.00098	0.005473	0.000597	3111	6016	5950	6282	5462
RNF144B	-0.79833	-0.56154	-0.86539	-0.65136	-0.65836	2.48E-08	0.000137	6.41E-10	7.36E-05	0.003578	3112	4787	2505	4788	6083
KLF9	-1.65551	-1.28995	-1.69141	-1.51907	-1.55609	2.50E-08	8.47E-06	4.43E-08	1.20E-06	1.18E-06	3115	3987	3349	3689	3771
NDUFS2	0.830592	0.586489	0.678144	0.552194	0.869383	2.50E-08	0.000537	0.000108	0.0012	8.80E-08	3116	5285	5237	5693	3251
RWDD4	-0.56255	-0.35349	-0.54485	-0.59363	-0.64445	2.50E-08	0.002284	5.93E-07	1.71E-05	3.52E-07	3117	5811	3906	4356	3538
YIPF6	0.646874	0.727148	0.536916	0.823924	0.498695	2.50E-08	1.39E-06	1.65E-05	2.21E-09	0.000339	3118	3562	4731	2534	5267
GART	1.098714	1.316487	1.170845	1.487699	1.179531	2.50E-08	4.86E-09	1.81E-07	2.62E-13	4.88E-10	3119	2436	3640	1344	2306
CDK10	1.027833	0.968273	1.203411	1.183803	0.913371	2.51E-08	3.21E-06	9.47E-12	1.98E-08	1.02E-07	3120	3773	1867	2884	3281
AP1G2	0.80901	0.802242	0.96912	0.711813	1.051227	2.51E-08	1.86E-07	2.39E-11	5.56E-07	2.00E-12	3121	3133	2006	3520	1555
DIO1	-0.70652	-0.4761	-0.55342	-0.6858	-0.70687	2.51E-08	0.000567	9.24E-05	2.57E-06	1.42E-07	3122	5308	5204	3856	3346
POLE3	0.787964	0.867034	0.890296	0.856936	0.683668	2.55E-08	1.01E-08	7.74E-08	1.61E-08	1.03E-05	3124	2555	3456	2848	4282
ACADM	-0.58069	-0.51924	-0.58641	-0.60665	-0.38935	2.55E-08	9.06E-08	9.82E-08	2.58E-08	0.000234	3125	2974	3513	2926	5165
C9orf135	-0.73688	-0.63102	-0.84693	-0.84644	-0.72449	2.56E-08	7.08E-07	5.58E-10	3.23E-08	9.35E-05	3126	3394	2479	2967	4912
VPS29	0.733164	0.739208	0.679537	0.983336	0.740772	2.58E-08	2.90E-09	5.88E-07	5.38E-13	1.75E-08	3127	2339	3904	1429	2918
CLDND1	-0.78089	-0.77554	-0.77871	-0.93921	-0.86581	2.61E-08	1.01E-07	4.82E-09	3.38E-10	4.35E-10	3128	2998	2868	2222	2285
STAP2	1.182848	1.51614	1.608472	1.470818	1.215778	2.62E-08	2.76E-10	9.02E-12	1.02E-11	2.59E-08	3129	1972	1857	1760	2984
OR11G2	-0.86415	-0.78549	-0.72235	-1.10922	-1.22876	2.62E-08	4.52E-08	1.42E-05	4.38E-12	7.21E-08	3130	2825	4691	1666	3212
PCBP1	0.957752	0.605397	0.984931	0.825188	0.77822	2.62E-08	5.60E-06	1.19E-08	1.85E-07	1.53E-07	3131	3889	3056	3283	3356
MSH5	1.261833	1.043991	0.853509	1.165265	0.880523	2.65E-08	1.09E-05	7.31E-05	4.12E-06	1.75E-06	3132	4050	5126	3971	3854
PDZD8	1.353991	1.251108	1.278329	1.209127	1.392705	2.67E-08	1.99E-09	4.55E-08	1.09E-08	1.73E-12	3133	2276	3355	2775	1540
IFNA14	-0.85105	-0.76715	-1.17068	-0.91345	-0.93695	2.68E-08	3.15E-08	1.20E-11	1.58E-07	1.08E-09	3134	2743	1896	3252	2440
LRP12	-1.04448	-0.76175	-1.32506	-1.0356	-1.16571	2.69E-08	1.27E-05	2.60E-11	9.54E-08	2.20E-08	3135	4095	2020	3157	2962
SS18	-0.88116	-0.95406	-0.80721	-1.19642	-0.80809	2.69E-08	5.94E-08	3.98E-07	1.88E-11	2.77E-08	3136	2878	3820	1856	3006
PEBP1	1.278337	1.273554	1.179315	1.464685	1.291351	2.70E-08	1.81E-09	3.81E-07	6.36E-13	1.09E-10	3137	2257	3807	1452	2065
ATAD2	0.848496	1.080979	1.227364	1.1183	1.235333	2.71E-08	6.35E-11	2.34E-12	1.05E-10	3.67E-14	3138	1772	1683	2068	1123
UBAC2	0.96347	0.865076	0.768206	0.871064	0.981741	2.71E-08	2.12E-08	5.15E-07	3.62E-09	2.29E-08	3139	2674	3874	2614	2968
MRPS18B	1.085662	0.921678	1.04844	0.970224	1.337908	2.73E-08	1.45E-06	2.38E-08	7.31E-07	5.64E-12	3140	3576	3194	3575	1686
GPR155	-0.74225	-0.68918	-1.17084	-0.82679	-1.51838	2.73E-08	9.87E-08	6.24E-12	5.93E-07	6.94E-15	3141	2993	1809	3534	950
TANK	-0.69684	-0.72884	-0.60552	-0.79236	-0.86212	2.74E-08	5.70E-09	6.71E-07	4.64E-10	5.77E-12	3142	2462	3936	2271	1688
NTAN1	-1.09958	-0.68888	-0.96533	-0.76861	-1.28649	2.74E-08	5.64E-05	8.45E-09	1.05E-05	4.07E-11	3143	4519	2983	4227	1930
RELT	-1.07913	-0.91644	-1.27038	-1.03221	-1.37658	2.74E-08	1.86E-06	5.82E-11	2.15E-08	1.09E-12	3144	3639	2130	2901	1496
CLNS1A	0.774207	0.934454	0.860591	0.996789	0.796723	2.78E-08	6.74E-11	3.56E-08	3.53E-13	9.81E-09	3145	1781	3305	1383	2809
PUF60	0.805269	0.896777	0.969492	0.834407	0.923789	2.78E-08	2.60E-09	2.21E-09	1.48E-08	2.00E-12	3146	2322	2724	2827	1556
POLDIP2	0.950821	0.926753	1.089768	1.13154	1.069234	2.79E-08	3.32E-08	1.76E-15	1.32E-09	8.84E-14	3147	2755	984	2442	1210
CSMD3	-0.60878	-0.3658	-0.43177	-0.46318	-0.56516	2.79E-08	0.001952	3.60E-05	0.000535	0.000135	3148	5752	4943	5408	5014
CGRRF1	-0.78285	-0.52286	-0.48591	-0.65829	-0.42539	2.79E-08	6.33E-05	2.70E-06	5.75E-08	5.74E-05	3149	4548	4254	3056	4753
PRKAB1	0.970453	1.359882	1.322763	1.387915	1.439755	2.79E-08	2.52E-11	1.38E-10	1.64E-12	1.87E-12	3150	1652	2265	1565	1546
PSMB8	1.045927	0.778712	1.198122	0.913206	1.505247	2.85E-08	2.59E-07	8.64E-11	7.69E-09	3.00E-16	3151	3189	2189	2714	724
XRCC5	0.823655	0.866156	1.123694	1.165718	1.102455	2.87E-08	1.11E-07	1.73E-11	6.19E-11	2.50E-11	3152	3017	1942	2003	1877
REV3L	-0.64269	-0.38225	-0.64823	-0.64247	-0.88303	2.88E-08	0.000465	2.78E-08	1.14E-06	2.32E-08	3153	5221	3235	3674	2970
ABO	1.478776	1.613284	1.445917	1.58008	1.600754	2.92E-08	7.07E-12	1.66E-08	3.60E-11	1.09E-12	3154	1485	3125	1937	1497
ASZ1	-0.78158	-0.60752	-0.794	-0.65633	-0.53041	2.95E-08	0.000121	1.13E-06	9.96E-06	7.06E-05	3155	4747	4069	4213	4828
SNUPN	-0.66203	-0.54893	-0.69592	-0.66108	-0.92024	2.95E-08	1.47E-06	1.38E-07	1.78E-06	5.29E-09	3156	3581	3586	3768	2691
THOC5	-0.71921	-0.4882	-0.45609	-0.47458	-0.68236	2.97E-08	0.000955	0.000245	0.001779	2.91E-08	3158	5495	5509	5843	3018
SLIT2	-1.03522	-0.78398	-0.58801	-0.90732	-0.94944	2.98E-08	3.12E-06	4.75E-05	2.79E-08	4.24E-09	3159	3769	5022	2941	2662
XAF1	-1.85869	-1.94896	-2.16075	-2.10591	-1.83154	3.00E-08	1.36E-08	3.36E-09	1.14E-13	3.62E-07	3160	2608	2809	1253	3547
PARP6	-0.90742	-0.59845	-0.49863	-0.94927	-0.50979	3.03E-08	0.000192	0.000457	6.90E-09	8.49E-05	3161	4902	5706	2704	4876
SRSF10	0.932245	1.189053	1.105582	1.21064	1.099963	3.03E-08	7.31E-10	9.74E-11	6.73E-11	8.75E-10	3162	2121	2214	2015	2408
HMGN1	1.014929	1.014124	1.159505	0.978714	1.596309	3.04E-08	7.43E-06	3.14E-08	4.92E-06	2.09E-13	3163	3950	3267	4017	1302
CXCL13	-1.30269	-0.9185	-0.85608	-1.13767	-1.12951	3.04E-08	0.001979	0.000678	0.000173	9.27E-07	3164	5764	5829	5006	3719
LZTS2	-0.96077	-0.86513	-0.9201	-0.96079	-1.25354	3.05E-08	3.45E-08	9.92E-08	5.81E-09	3.02E-13	3165	2770	3517	2675	1337
PABPN1	0.720813	0.512224	0.834484	0.50469	0.815187	3.07E-08	0.000219	3.06E-07	0.001358	9.58E-09	3166	4939	3766	5742	2806
KRTAP12-3	-0.88475	-0.60415	-1.19737	-0.89767	-0.99414	3.07E-08	0.001171	1.39E-06	0.000316	1.57E-08	3167	5575	4115	5235	2901
TMBIM1	1.091657	0.835445	1.570194	1.129251	0.843801	3.07E-08	2.70E-06	5.28E-11	2.09E-08	5.09E-05	3168	3732	2113	2896	4728
C12orf75	1.273129	1.215752	1.625482	1.367394	1.166249	3.08E-08	4.59E-08	2.10E-10	2.30E-09	5.72E-08	3169	2830	2331	2540	3152
MRPS10	0.777348	0.860571	0.745539	0.817024	0.622251	3.10E-08	2.07E-11	2.13E-10	9.94E-12	1.81E-07	3170	1619	2335	1757	3391
TCF20	0.860233	1.263393	1.019743	1.265466	0.617342	3.15E-08	5.46E-10	8.25E-09	1.99E-10	2.39E-05	3171	2084	2977	2149	4508
HADH	1.257234	0.960662	1.437049	0.848439	1.509212	3.17E-08	2.03E-06	3.96E-11	3.29E-05	2.21E-11	3172	3661	2079	4550	1869
DLG3	0.79829	0.64137	0.529285	0.932206	0.664274	3.20E-08	8.82E-06	0.000355	3.00E-09	0.000119	3173	4002	5627	2586	4990
LYSMD2	-0.65393	-0.46611	-0.558	-0.55425	-0.7997	3.22E-08	0.000332	1.13E-06	2.88E-05	2.43E-08	3174	5103	4068	4502	2977
IL17RE	1.073933	0.926244	0.878834	0.663098	1.279928	3.28E-08	3.14E-07	2.21E-07	2.91E-05	5.78E-10	3176	3226	3678	4504	2331
GOT2	0.902361	0.752721	1.067152	0.88594	1.181183	3.28E-08	2.04E-06	1.26E-10	3.49E-07	8.68E-13	3177	3663	2252	3414	1464
SH2D3A	0.971047	1.336638	1.217972	1.251106	0.898039	3.30E-08	2.93E-10	2.63E-11	7.26E-10	6.61E-08	3178	1982	2024	2346	3177
H2AC14	1.295456	0.937491	1.302091	1.263865	1.311229	3.30E-08	3.58E-07	3.19E-09	6.06E-10	8.46E-09	3179	3249	2800	2311	2775
DNAJC11	0.955239	0.789736	0.805797	0.817894	0.913377	3.31E-08	2.70E-06	1.11E-06	8.38E-09	2.02E-08	3180	3731	4061	2727	2943
CCDC12	0.666072	0.582874	0.667651	0.448993	0.544453	3.32E-08	6.28E-06	3.29E-08	0.000237	2.61E-05	3181	3915	3286	5120	4526
SOD3	-1.78233	-1.63706	-1.26669	-1.47432	-0.97213	3.34E-08	7.78E-10	1.56E-05	8.00E-07	0.005695	3182	2132	4716	3590	6295
FBXO32	-1.00385	-1.0011	-0.96585	-1.16083	-1.52172	3.35E-08	4.88E-08	5.89E-08	5.85E-09	1.13E-13	3183	2845	3406	2678	1234
EEF1E1	1.04449	1.026261	1.005508	0.919083	1.070718	3.35E-08	3.97E-08	3.78E-08	2.47E-09	4.28E-09	3184	2799	3317	2551	2666
CTPS1	0.691545	0.844972	0.640409	0.849259	0.678846	3.35E-08	5.07E-11	7.36E-08	4.23E-12	3.10E-09	3185	1754	3448	1658	2609
H2BC13	1.301924	1.19374	1.661908	1.1909	1.174533	3.35E-08	1.30E-07	4.92E-11	5.75E-08	7.66E-07	3186	3055	2107	3057	3683

NFASC	-0.93916	-0.79867	-1.02699	-0.89198	-0.81385	3.43E-08	3.05E-06	6.06E-13	1.71E-06	7.04E-07	3187	3765	1537	3759	3664
APEX1	0.767234	0.791627	0.874919	0.878257	0.795039	3.43E-08	4.42E-09	8.02E-09	7.27E-10	8.57E-07	3188	2412	2966	2347	3702
SATB2	1.881303	2.371992	1.773285	2.279195	2.062845	3.47E-08	7.75E-12	9.27E-09	7.04E-12	1.58E-11	3189	1504	2997	1710	1823
DBI	1.31852	1.45484	1.731918	1.455074	1.487007	3.49E-08	6.41E-12	3.93E-15	4.17E-11	1.04E-15	3190	1471	1045	1953	808
CPEB4	-1.22062	-0.97743	-1.45562	-1.19512	-1.53117	3.56E-08	4.68E-08	1.88E-11	8.00E-09	1.38E-10	3191	2838	1967	2718	2103
CPSF6	0.646104	0.609787	0.505191	0.631114	0.711892	3.59E-08	1.85E-08	1.45E-07	1.14E-08	1.16E-08	3192	2648	3598	2784	2849
PITRM1	0.744091	0.672751	0.729036	0.618496	0.545343	3.60E-08	3.65E-06	8.44E-08	4.06E-05	0.000251	3193	3798	3473	4610	5187
TSFM	0.930759	1.149746	0.946482	0.934095	1.004572	3.60E-08	3.78E-09	9.16E-08	5.29E-07	3.07E-10	3194	2389	3491	3510	2231
EEF1G	1.686122	1.598792	1.550736	1.931796	1.66092	3.62E-08	1.79E-09	1.52E-06	2.02E-14	2.59E-10	3195	2254	4129	1097	2206
DENND6B	-0.80451	-0.69103	-0.82801	-0.89554	-0.60483	3.63E-08	7.72E-09	5.32E-08	7.55E-11	0.000192	3197	2511	3389	2031	5114
PML	-0.98852	-0.72748	-0.94808	-0.81772	-0.80658	3.66E-08	1.15E-06	1.15E-11	7.47E-07	3.24E-07	3198	3518	1890	3577	3518
CYP20A1	-0.48533	-0.44912	-0.43797	-0.40871	-0.63467	3.67E-08	1.19E-06	7.88E-07	2.41E-05	1.03E-10	3199	3524	3976	4438	2057
OXSR1	0.654171	0.621351	0.79354	0.550575	0.813084	3.67E-08	1.47E-07	2.05E-11	3.91E-06	1.11E-06	3200	3080	1982	3964	3760
C6orf136	0.87084	0.93935	1.187914	0.787302	0.88551	3.76E-08	1.83E-09	6.90E-16	4.38E-08	1.20E-08	3202	2259	900	3021	2854
NIN	-0.82304	-0.46162	-0.5901	-0.49733	-0.54763	3.77E-08	0.002117	0.000211	0.00707	0.001492	3203	5791	5457	6387	5788
DNAJC4	-0.72414	-0.59465	-0.62013	-0.59254	-0.70472	3.82E-08	1.03E-07	4.88E-08	1.02E-06	2.62E-07	3205	3002	3365	3649	3470
NEGR1	-0.66199	-0.5678	-0.74316	-0.70179	-0.59822	3.84E-08	1.12E-05	1.21E-08	2.73E-07	1.70E-07	3206	4056	3062	3358	3383
MRPL2	0.784552	0.722033	0.7485	0.667031	0.641634	3.86E-08	1.55E-07	1.12E-07	4.27E-07	8.41E-07	3207	3091	3545	3460	3698
ZDHHC17	-0.65516	-0.48798	-0.57282	-0.61077	-0.66826	3.86E-08	2.10E-05	4.49E-07	1.04E-07	1.05E-08	3208	4225	3844	3173	2827
ACSL4	-1.4847	-1.43274	-1.60393	-1.40365	-1.65128	3.88E-08	1.98E-08	3.19E-10	2.77E-08	5.72E-09	3209	2659	2393	2938	2703
SOCS5	-0.77215	-0.63336	-0.81253	-0.69318	-0.71785	3.90E-08	1.52E-06	3.46E-10	6.65E-07	2.97E-09	3210	3587	2411	3564	2605
TAS1R3	-0.76984	-0.44079	-0.97851	-0.59729	-0.60274	3.94E-08	4.48E-05	1.99E-12	1.43E-06	2.59E-05	3212	4448	1664	3718	4523
NPM3	0.946677	1.33953	1.138549	1.373836	0.815825	3.95E-08	8.69E-10	1.82E-09	2.14E-12	3.59E-06	3213	2153	2680	1590	4029
HNRNPCL3	-0.78835	-0.44832	-1.07606	-0.77557	-0.62543	3.96E-08	0.000186	4.07E-12	4.02E-07	3.95E-06	3214	4894	1754	3447	4048
TRIM51	-0.9221	-0.67259	-1.07126	-0.83217	-0.88334	3.96E-08	8.17E-06	2.13E-10	1.47E-06	8.98E-09	3215	3976	2334	3727	2791
TOMM40	1.178183	1.368901	1.261101	1.201779	1.496588	3.97E-08	1.37E-07	3.48E-08	3.14E-07	2.44E-12	3216	3064	3301	3389	1584
LAMTOR2	0.673618	0.416821	0.556598	0.540231	0.743585	4.00E-08	0.001913	1.40E-05	0.00035	2.01E-07	3217	5742	4686	5266	3414
KCNK4	-0.64643	-0.44727	-0.88059	-0.56457	-0.43192	4.01E-08	0.000133	3.06E-11	3.75E-05	0.008113	3218	4776	2050	4589	6446
PFDN2	1.051048	1.091547	1.020316	1.269861	1.095421	4.01E-08	4.78E-09	3.00E-07	2.66E-15	6.35E-10	3219	2427	3758	918	2348
NOXA1	0.965089	1.492441	0.99838	1.400992	1.439352	4.07E-08	1.42E-10	1.53E-07	6.97E-09	1.21E-10	3220	1879	3604	2705	2078
HMGCS1	1.410458	1.526348	1.74062	1.535029	1.462908	4.10E-08	3.02E-08	4.33E-12	1.01E-08	1.19E-13	3221	2736	1762	2766	1241
FUT6	1.621797	1.584831	1.881812	1.683715	1.937081	4.11E-08	1.92E-07	5.88E-13	2.05E-08	3.68E-15	3222	3138	1532	2891	893
ZNF264	-1.12873	-0.74638	-0.44301	-0.9001	-0.66198	4.15E-08	8.63E-05	0.008379	2.37E-06	9.64E-06	3223	4652	6785	3840	4269
PDCL	-0.69324	-0.41472	-0.31236	-0.42922	-0.83023	4.15E-08	0.00688	0.001572	0.007306	1.36E-09	3224	6247	6100	6400	2477
UBAP2L	0.817313	0.506704	0.449731	0.571567	0.538134	4.22E-08	0.000438	0.002285	3.21E-05	3.47E-05	3225	5203	6259	4539	4609
FAM193A	-0.64497	-0.50887	-0.47489	-0.6744	-0.42638	4.26E-08	1.40E-05	7.85E-06	2.14E-08	0.000175	3226	4115	4521	2900	5084
PIP4P2	-1.20105	-0.67898	-1.00782	-0.88172	-1.22951	4.27E-08	2.16E-05	8.19E-10	3.01E-07	9.28E-14	3227	4235	2549	3375	1214
CD59	-1.40883	-1.41232	-1.2236	-1.61186	-1.65056	4.28E-08	1.42E-10	1.15E-08	3.68E-16	5.71E-11	3228	1880	3047	783	1964
CHAT	-0.72344	-0.68059	-0.61185	-0.71729	-0.62943	4.29E-08	2.48E-07	5.28E-07	6.67E-07	1.55E-07	3229	3186	3883	3565	3360
PLCB3	1.017974	1.042826	1.295856	1.263387	0.8073	4.31E-08	1.99E-07	5.83E-10	1.23E-08	6.50E-06	3230	3147	2486	2799	4168
DLG1	0.848263	0.853846	0.791333	0.753432	0.661157	4.33E-08	8.63E-07	1.87E-08	2.81E-07	5.77E-07	3231	3440	3156	3364	3631
UCK2	0.793655	0.827294	0.953405	0.845106	0.836107	4.34E-08	1.19E-05	2.58E-09	1.84E-06	9.90E-07	3232	4078	2760	3783	3736
USP54	1.130807	1.275658	1.466433	1.570372	1.104862	4.35E-08	4.68E-10	1.69E-08	3.41E-10	3.19E-07	3233	2055	3130	2225	3517
CPEB3	-0.96152	-1.13776	-1.08727	-1.10876	-0.9361	4.38E-08	1.09E-07	4.71E-08	1.12E-06	7.11E-08	3234	3014	3360	3667	3202
SYNGAP1	-1.08681	-0.88897	-1.16922	-1.15199	-1.40876	4.38E-08	8.40E-06	5.85E-10	2.79E-07	5.91E-09	3235	3985	2488	3361	2712
TASP1	0.906869	1.232472	0.673557	1.102837	0.637197	4.38E-08	3.88E-12	1.66E-06	3.16E-10	7.38E-06	3236	1402	4146	2214	4204
GLO1	1.320246	1.300209	1.395954	1.453288	1.398747	4.38E-08	1.90E-10	1.77E-11	6.54E-12	3.11E-10	3237	1915	1943	1705	2232
AHR	-1.12761	-1.47694	-1.00167	-1.14064	-0.97003	4.39E-08	9.44E-11	1.37E-07	1.77E-08	5.17E-05	3238	1825	3584	2865	4734
PCK2	1.322267	1.557883	1.124181	1.168895	1.418982	4.39E-08	8.07E-09	2.57E-07	3.68E-05	2.64E-07	3239	2518	3712	4580	3471
ITPRIPL2	-0.8793	-1.01961	-0.8455	-0.86439	-1.2291	4.40E-08	2.83E-11	6.48E-10	7.05E-10	4.14E-12	3241	1665	2508	2340	1651
EI24	0.800342	1.168159	1.07528	1.18546	0.587044	4.41E-08	2.64E-07	4.66E-08	3.76E-07	0.00096	3242	3193	3359	3431	5614
METTL26	0.850812	0.879175	0.916427	0.973062	0.951554	4.43E-08	3.02E-09	1.04E-08	1.67E-10	4.22E-10	3243	2348	3020	2118	2278
PAQR5	-0.86528	-0.54482	-0.80413	-1.00641	-0.97265	4.51E-08	2.20E-06	4.43E-08	2.74E-11	2.07E-09	3245	3685	3348	1902	2542
LY6G6D	2.002438	2.644355	1.995501	2.603583	2.102992	4.51E-08	1.66E-10	1.61E-07	1.13E-10	1.44E-09	3246	1898	3616	2073	2485
GAA	-0.86494	-0.55474	-0.60737	-0.67758	-0.76225	4.57E-08	9.56E-06	2.80E-06	2.02E-07	4.24E-08	3247	4020	4261	3304	3091
FAM219B	-0.79841	-0.6034	-0.8277	-0.83648	-0.92678	4.59E-08	1.44E-06	2.44E-08	9.37E-09	4.03E-10	3248	3574	3205	2750	2269
LXN	-1.4302	-1.38307	-0.99707	-1.36919	-1.15816	4.60E-08	2.07E-07	3.36E-05	4.82E-08	9.01E-08	3249	3159	4926	3030	3257
COX17	0.752455	1.039122	0.670056	0.869484	0.906835	4.63E-08	2.01E-10	6.75E-08	7.04E-10	5.66E-09	3250	1925	3436	2339	2702
FOXO4	-0.54109	-0.33696	-0.44866	-0.42838	-0.42036	4.64E-08	0.001412	4.50E-07	2.61E-05	5.09E-05	3251	5636	3845	4465	4729
CNIH4	0.845527	0.886575	0.872922	0.967794	0.688313	4.65E-08	8.83E-08	8.96E-08	2.79E-08	2.05E-07	3252	2968	3487	2942	3419
PPP4C	0.68348	0.413394	0.870233	0.765035	0.950253	4.68E-08	6.46E-05	8.93E-10	1.16E-07	1.73E-09	3254	4555	2562	3194	2514
TMEM175	-0.93983	-0.5751	-0.68208	-0.79395	-0.69849	4.68E-08	0.000226	5.35E-05	1.65E-06	6.11E-05	3255	4950	5054	3752	4777
XPO6	0.777953	0.549618	0.641131	0.846021	0.677994	4.75E-08	6.70E-05	1.12E-07	9.56E-08	0.000499	3256	4572	3548	3158	5404
PLEKHH1	0.929376	1.027273	1.136163	0.946593	1.196336	4.80E-08	3.52E-08	4.84E-11	1.19E-07	3.86E-13	3257	2775	2105	3199	1367
RPS25	1.481812	1.440197	1.194871	1.589229	1.32704	4.81E-08	5.66E-09	1.93E-06	1.86E-12	3.58E-10	3258	2461	4178	1579	2244
SALL4	-1.0155	-0.75412	-0.60765	-0.92215	-0.77466	4.84E-08	2.18E-06	1.95E-05	4.03E-08	2.93E-05	3259	3683	4776	3007	4551
NUDCD3	0.757064	0.886424	0.491787	0.906495	0.811938	4.84E-08	1.23E-10	6.26E-06	5.09E-11	2.07E-08	3260	1855	4460	1979	2948
FKBP3	0.718742	0.721017	0.637428	0.762931	0.667251	4.84E-08	1.37E-06	1.53E-05	8.96E-09	7.26E-06	3261	3558	4710	2740	4195
SLC3A2	1.00353	0.902534	1.015785	0.889401	0.624932	4.87E-08	6.85E-07	8.66E-09	4.40E-07	0.001496	3262	3385	2987	3470	5790
ZNF419	-0.94302	-0.47518	-0.99634	-0.59208	-0.72871	4.90E-08	0.002815	7.47E-10	0.000341	1.19E-06	3263	5871	2533	5256	3773
IPO5	1.464227	1.350292	1.480118	1.286037	1.641954	4.92E-08	9.10E-08	2.56E-08	4.87E-08	1.11E-11	3264	2976	3219	3032	1780
TSN	0.873633	0.927904	1.096742	0.987692	1.061244	4.93E-08	3.55E-09	3.86E-10	8.06E-09	8.12E-13	3265	2380	2425	2719	1457
CANX	1.018379	0.732876	1.049459	0.888041	0.771964	4.94E-08	1.20E-06	1.59E-07	8.07E-08	1.16E-06	3266	3526	3610	3128	3766

C19orf53	0.774039	0.912801	0.871054	1.024627	0.653304	5.01E-08	2.34E-09	2.86E-08	1.25E-12	3.80E-07	3268	2300	3246	1529	3559
RARA	-0.79754	-0.88836	-0.60546	-0.79798	-1.15741	5.04E-08	4.14E-09	2.22E-06	1.11E-07	7.02E-11	3269	2406	4202	3189	2006
MAD2L1	1.076249	0.91007	1.225107	0.989853	0.903693	5.04E-08	1.47E-06	9.61E-11	3.42E-08	1.21E-06	3270	3579	2213	2979	3776
CRIM1	-1.05542	-1.01879	-0.56631	-0.99541	-0.71845	5.07E-08	4.45E-07	0.000349	1.02E-07	5.84E-06	3271	3289	5613	3171	4146
ZNF787	0.629042	0.696591	0.775714	0.562994	0.744852	5.09E-08	2.51E-08	1.23E-10	2.17E-07	1.08E-09	3272	2702	2247	3313	2441
RPS17	1.530319	1.510874	1.335874	1.465184	1.720999	5.14E-08	2.19E-09	6.78E-07	1.50E-10	7.28E-13	3273	2293	3937	2108	1446
THRA	0.751615	0.972448	1.059133	0.988361	1.166369	5.15E-08	4.62E-06	2.92E-09	1.09E-07	5.96E-11	3274	3845	2779	3183	1970
OR2M5	-0.84521	-0.46897	-0.97271	-0.71762	-0.75117	5.27E-08	0.000185	1.77E-11	1.92E-06	2.74E-07	3276	4887	1944	3797	3481
CMTM7	-0.77498	-0.37013	-0.83655	-0.45916	-1.07351	5.28E-08	0.003511	1.39E-07	0.003695	3.14E-12	3277	5963	3588	6124	1619
CASP4	-1.57962	-1.57133	-1.33159	-1.73583	-1.49146	5.29E-08	1.02E-08	1.20E-06	4.31E-08	6.86E-08	3278	2556	4082	3019	3190
DRD5	-0.80204	-0.65841	-0.99886	-0.84353	-0.9492	5.33E-08	3.04E-07	1.96E-09	2.59E-06	9.36E-09	3280	3224	2697	3861	2799
WDR77	0.918426	0.776254	0.660859	0.948659	1.03068	5.38E-08	8.73E-07	2.41E-05	3.74E-10	3.49E-09	3281	3442	4830	2240	2630
MAGEA4	-0.7305	-0.39072	-0.84853	-0.69889	-0.78983	5.42E-08	5.24E-05	4.04E-09	1.90E-06	1.65E-07	3282	4501	2842	3794	3373
EMP1	-2.02592	-2.39309	-2.14559	-2.48498	-2.66463	5.43E-08	1.56E-09	1.33E-07	8.61E-11	8.81E-10	3283	2235	3575	2047	2409
CA5B	-0.72304	-0.54265	-0.598	-0.46891	-0.90336	5.44E-08	1.48E-05	4.64E-06	7.57E-05	1.31E-10	3284	4129	4388	4794	2088
MAVS	1.089368	0.888727	0.893622	0.955932	0.98554	5.49E-08	9.21E-07	7.77E-06	2.37E-07	3.46E-09	3285	3460	4519	3329	2625
NBEAL2	1.167328	0.948151	0.902744	0.97333	1.20993	5.49E-08	1.72E-05	8.72E-06	3.62E-06	2.60E-08	3286	4167	4554	3933	2987
C11orf68	-0.64756	-0.41195	-0.7022	-0.46593	-0.8763	5.55E-08	0.000681	2.05E-10	0.000262	9.39E-11	3287	5372	2328	5164	2043
TMEM14A	0.813251	0.805008	0.666954	0.67857	0.577783	5.59E-08	1.62E-06	4.47E-06	0.000103	0.000103	3288	3604	4380	4863	4945
KIF16B	1.175532	0.903689	1.085177	0.923298	0.783708	5.61E-08	1.51E-06	3.85E-08	5.55E-05	0.000193	3289	3586	3324	4708	5116
MALRD1	-0.5949	-0.47909	-0.42295	-0.66233	-0.70422	5.64E-08	0.000159	0.001302	2.81E-08	7.96E-06	3290	4828	6041	2944	4221
FABP5	1.19569	1.544054	1.68992	1.7336	1.515134	5.65E-08	2.93E-09	4.37E-09	4.41E-13	1.12E-08	3291	2342	2855	1410	2843
CYFIP2	-0.93683	-0.54664	-0.75512	-0.69789	-0.80616	5.69E-08	0.000384	1.56E-10	4.23E-05	1.03E-07	3292	5156	2289	4622	3286
PDRG1	0.760272	0.72439	0.584885	0.718453	0.535662	5.71E-08	4.48E-07	2.96E-06	1.36E-06	0.000213	3293	3293	4277	3707	5136
TMEM184	-0.87411	-0.7888	-0.76676	-0.62109	-0.57661	5.75E-08	9.52E-09	3.73E-09	1.18E-06	0.006879	3294	2546	2831	3687	6366
PAM	-1.54387	-1.51568	-1.25583	-1.72737	-1.71454	5.77E-08	7.52E-09	1.75E-07	4.35E-10	2.39E-09	3295	2503	3632	2259	2576
ESRRA	0.791467	1.17922	0.877749	1.068101	0.716603	5.83E-08	9.90E-14	3.24E-10	4.87E-11	2.49E-06	3296	1034	2399	1974	3938
RAB4B	-0.64407	-0.59027	-0.64942	-0.62856	-0.5016	5.83E-08	9.38E-07	8.13E-07	1.28E-07	0.000119	3297	3467	3982	3208	4983
CUL3	0.579474	0.819147	0.723707	0.935378	0.593849	5.86E-08	3.97E-12	3.19E-10	2.81E-13	3.52E-07	3298	1405	2392	1355	3536
ETFA	0.780736	0.901617	0.878002	0.804802	0.798924	5.89E-08	6.68E-10	7.06E-09	3.29E-08	8.17E-09	3299	2107	2944	2973	2771
DNAJC22	0.857164	0.639482	0.625979	0.653125	0.638148	5.90E-08	0.000182	8.70E-06	0.000202	3.41E-06	3300	4878	4552	5063	4008
NRDE2	-0.58469	-0.48746	-0.56825	-0.49511	-0.61179	5.91E-08	2.61E-05	1.09E-07	1.37E-05	1.16E-08	3301	4286	3535	4291	2848
FAHD1	0.768817	0.801466	0.605638	0.938226	0.392385	5.97E-08	6.60E-07	3.74E-07	1.60E-08	0.001105	3302	3373	3804	2844	5671
IL4I1	-0.83839	-0.54548	-0.80118	-0.7039	-0.83759	5.98E-08	0.0008	5.72E-08	2.46E-05	4.16E-06	3303	5419	3398	4445	4063
KCTD10	-0.92258	-0.74289	-0.95735	-0.82015	-0.83362	6.01E-08	7.85E-05	6.53E-08	4.40E-06	1.07E-06	3304	4623	3432	3988	3755
LMF2	-0.87993	-0.95063	-0.62024	-0.80761	-0.87132	6.01E-08	8.10E-13	2.41E-07	1.29E-08	7.16E-10	3305	1232	3700	2807	2371
OPTN	-1.13705	-0.73844	-0.674	-0.73602	-0.77536	6.03E-08	6.73E-06	5.39E-05	2.38E-05	0.000798	3306	3927	5056	4430	5563
WRNIP1	0.937774	0.958665	1.038978	1.050352	1.203348	6.11E-08	7.03E-08	5.48E-07	9.26E-10	2.24E-10	3308	2909	3887	2387	2183
NR1H3	-1.28686	-1.67703	-1.32433	-1.71097	-1.00837	6.11E-08	1.58E-09	1.91E-09	1.40E-10	0.000272	3309	2237	2686	2104	5212
CES2	2.022101	2.041586	2.396181	1.937061	2.136414	6.18E-08	5.36E-09	5.95E-10	1.88E-07	2.28E-08	3310	2452	2497	3287	2967
CYB561A3	-0.71438	-0.52797	-0.44668	-0.57833	-0.66704	6.18E-08	0.000122	0.000507	0.000225	2.16E-06	3312	4748	5753	5105	3900
FANCC	-0.5592	-0.35244	-0.63054	-0.48909	-0.65132	6.19E-08	0.001936	1.05E-08	6.35E-05	0.000438	3313	5747	3023	4747	5362
NCAPD3	0.804083	0.662784	0.750427	0.725278	0.49733	6.25E-08	6.07E-06	7.68E-09	1.85E-06	0.001126	3314	3908	2958	3784	5681
LRP11	0.889037	0.797436	0.722521	1.026704	0.548511	6.31E-08	1.36E-05	1.67E-08	1.73E-08	0.000246	3316	4110	3128	2861	5180
CSNK2A3	0.700278	0.674007	0.774471	0.594543	0.80542	6.31E-08	1.59E-08	6.13E-11	3.82E-07	2.00E-09	3317	2631	2139	3435	2538
NPM1	1.505627	1.561994	1.286317	1.700453	1.558005	6.31E-08	1.71E-09	3.43E-06	8.63E-14	7.88E-10	3318	2249	4306	1230	2387
UBAP2	0.782466	1.001789	1.062031	0.951738	0.800079	6.34E-08	3.79E-08	3.46E-12	4.90E-08	2.86E-07	3319	2787	1740	3033	3489
PFN2	-0.76019	-0.44861	-0.59518	-0.58681	-0.76279	6.35E-08	0.00358	0.000202	0.000276	8.73E-06	3320	5968	5443	5191	4248
VPS37A	-0.77919	-0.75539	-0.51269	-0.80688	-0.757	6.38E-08	5.79E-08	0.000254	1.82E-08	2.94E-08	3321	2873	5522	2868	3020
TAF1D	0.862134	1.084451	0.935289	1.087502	0.895439	6.38E-08	2.37E-08	1.12E-08	5.34E-08	7.52E-11	3322	2694	3035	3045	2013
TUBA1C	1.366305	0.839401	1.629444	1.085311	1.267032	6.42E-08	2.06E-05	5.06E-09	5.07E-06	6.35E-09	3323	4220	2876	4025	2720
RAB27A	-0.94525	-0.51374	-0.99289	-0.63339	-1.01637	6.43E-08	0.00812	2.33E-06	0.002679	3.09E-07	3324	6337	4217	6000	3509
GDI1	-0.68454	-0.67457	-0.35524	-0.50805	-0.49085	6.48E-08	2.78E-08	0.003581	9.74E-05	1.46E-05	3325	2725	6436	4851	4391
NSMCE3	-0.53869	-0.36041	-0.39878	-0.46385	-0.60377	6.50E-08	0.000233	3.58E-05	3.55E-05	7.16E-09	3326	4961	4941	4570	2743
E2F1	0.934033	1.254476	1.031383	1.102925	0.994387	6.54E-08	1.98E-10	1.23E-08	2.36E-10	8.56E-08	3327	1922	3066	2173	3245
AKAP12	-1.74034	-1.22803	-1.67069	-1.42905	-1.65584	6.56E-08	2.99E-05	3.46E-10	1.30E-05	1.38E-10	3329	4331	2410	4276	2102
OVOL1	1.081849	1.6368	1.399477	2.012279	1.160757	6.67E-08	7.01E-10	2.48E-09	1.20E-13	3.95E-08	3330	2112	2753	1257	3069
TSHZ1	-0.6296	-0.64053	-0.5958	-0.67079	-0.56116	6.68E-08	7.72E-06	6.99E-07	3.82E-06	1.79E-08	3331	3964	3943	3950	2926
LMBRD1	-0.4322	-0.3143	-0.45704	-0.34717	-0.4547	6.68E-08	7.12E-05	1.08E-09	5.36E-05	5.02E-07	3332	4591	2589	4689	3604
CCM2	-0.48372	-0.41904	-0.54729	-0.52743	-0.43023	6.70E-08	1.49E-05	7.17E-09	1.14E-06	0.000174	3333	4130	2947	3678	5081
AATF	0.751605	0.833355	1.027797	0.708803	0.555591	6.75E-08	2.58E-07	1.08E-11	1.83E-06	1.09E-05	3334	3188	1885	3778	4300
FNBP1L	0.918142	0.85926	1.334123	1.230117	0.930884	6.75E-08	1.38E-08	5.89E-09	3.23E-07	4.33E-05	3335	2613	2909	3393	4680
CXCR2	-0.82531	-0.45096	-0.88979	-0.63838	-0.94307	6.80E-08	0.002261	1.20E-07	6.87E-05	2.31E-08	3336	5806	3562	4765	2969
SELENON	-0.79864	-1.15952	-0.61958	-0.9555	-0.50514	6.82E-08	1.28E-09	0.000703	3.71E-06	0.001235	3337	2211	5847	3941	5715
CCZ1	0.615706	0.488056	0.889083	0.7801	0.785507	6.85E-08	5.27E-06	4.28E-08	1.02E-09	1.09E-07	3339	3876	3343	2397	3294
CKS1B	0.910043	1.122045	0.856174	0.996732	1.073814	6.91E-08	2.40E-08	2.30E-08	3.42E-07	4.31E-09	3340	2695	3188	3410	2669
RSU1	-0.92101	-0.84923	-0.6178	-0.99629	-0.71056	6.94E-08	6.09E-08	6.54E-06	2.31E-09	8.16E-05	3341	2884	4470	2541	4858
PABIR2	0.976041	1.125936	0.948965	1.090305	0.692336	6.95E-08	1.97E-10	3.88E-08	7.44E-10	3.18E-06	3342	1920	3325	2350	3994
PFDN6	1.264807	1.264671	1.488198	1.266736	1.17978	7.02E-08	1.42E-08	2.49E-12	1.59E-08	5.75E-09	3344	2620	1692	2842	2704
RPL7L1	0.74602	0.970462	0.8445	1.034672	1.046426	7.05E-08	9.19E-10	7.10E-07	1.62E-10	1.38E-10	3345	2163	3949	2111	2101
FAM83E	1.086128	1.027291	1.341251	1.098981	1.121612	7.05E-08	8.16E-08	5.17E-13	5.47E-10	4.23E-11	3346	2949	1518	2293	1935
CSRNP1	-0.92625	-0.71335	-0.96429	-0.92259	-1.08632	7.05E-08	2.64E-05	1.13E-08	5.82E-07	6.30E-11	3347	4294	3037	3531	1984
SORD	0.840048	1.061207	1.260571	1.192531	1.276127	7.07E-08	1.70E-07	5.85E-08	2.60E-07	5.60E-10	3348	3116	3404	3346	2324

MSH2	0.61794	0.715754	0.567212	0.749248	0.623373	7.08E-08	1.17E-07	1.88E-07	5.27E-07	1.62E-06	3349	3033	3648	3505	3839
FLAD1	0.79066	0.935961	0.782998	0.861725	0.668962	7.10E-08	1.43E-07	5.14E-09	2.72E-07	2.85E-06	3351	3076	2880	3357	3962
LGR4	1.101791	1.33563	1.642389	1.488135	0.997727	7.26E-08	2.11E-09	4.72E-13	5.90E-11	8.21E-06	3352	2285	1506	1996	4230
MTFR1	0.879832	0.939194	1.005149	0.981512	0.433695	7.27E-08	1.98E-08	2.45E-12	4.93E-08	0.009427	3353	2660	1687	3036	6537
ATF6B	0.875223	0.541123	0.694556	0.552456	0.950696	7.27E-08	0.002355	4.79E-06	0.002571	3.14E-07	3354	5821	4400	5978	3515
SRSF3	1.092372	0.747903	0.93564	0.871432	1.194072	7.28E-08	0.00038	3.13E-05	3.24E-05	5.21E-09	3355	5153	4908	4544	2688
BICD2	-0.80474	-0.3783	-0.84636	-0.51251	-0.79884	7.30E-08	0.00812	3.73E-09	0.000388	1.61E-09	3356	6338	2830	5297	2504
HSPA2	-1.80461	-1.56115	-1.66863	-1.09817	-1.25652	7.36E-08	1.14E-07	3.16E-09	0.000601	0.00058	3357	3022	2798	5436	5448
HEBP1	-0.67719	-0.66976	-0.4996	-0.588	-0.41657	7.45E-08	1.57E-07	0.000103	1.75E-05	0.001231	3358	3093	5223	4361	5712
CUL4A	1.101224	0.977103	1.154952	1.073193	1.400955	7.50E-08	8.17E-07	5.53E-10	2.37E-07	3.66E-13	3359	3427	2475	3328	1362
LIPH	1.07415	0.99663	1.105399	1.058375	0.754245	7.55E-08	2.07E-06	4.84E-07	1.13E-08	8.65E-06	3360	3669	3860	2782	4246
TMEM147	0.88998	0.875375	0.834179	0.867098	0.724232	7.60E-08	2.00E-06	7.21E-07	2.62E-07	2.29E-05	3362	3657	3954	3349	4498
LAMTOR4	0.799003	0.790818	0.782823	0.640357	0.670403	7.75E-08	1.07E-07	4.97E-08	5.78E-07	2.10E-06	3363	3010	3372	3528	3887
ILK	-1.24526	-1.66426	-1.00601	-1.32597	-1.50676	7.80E-08	8.01E-12	5.65E-05	5.34E-10	1.22E-08	3364	1507	5063	2287	2860
CCDC6	0.510219	0.780013	0.853394	0.747481	0.497391	7.81E-08	8.68E-06	5.00E-09	5.02E-05	4.85E-06	3365	3996	2874	4676	4093
IGFBP2	1.310231	0.917669	1.460612	1.191366	1.840593	7.96E-08	0.000337	1.78E-10	0.000208	2.60E-07	3368	5106	2308	5075	3469
AP3S2	-0.51698	-0.30854	-0.44641	-0.50534	-0.40099	7.98E-08	0.008013	0.001282	9.80E-06	0.000953	3369	6329	6037	4205	5611
CMSS1	0.886546	1.140628	1.161491	0.976112	0.765102	7.99E-08	7.20E-10	8.71E-12	9.17E-09	2.37E-05	3370	2116	1851	2746	4507
ATP5MG	0.989199	0.801599	0.815083	0.819799	0.765961	8.01E-08	6.79E-06	6.58E-06	6.80E-06	1.68E-07	3371	3929	4471	4100	3378
EPHB4	0.875541	0.89108	0.633531	0.998174	1.092671	8.06E-08	2.50E-08	7.50E-05	1.51E-10	6.20E-08	3372	2700	5136	2109	3168
AFAP1	-0.78972	-0.72918	-0.45853	-0.53736	-0.77442	8.11E-08	2.18E-06	0.004635	0.003583	7.42E-05	3374	3682	6542	6110	4836
PPP1R14D	1.366614	1.777171	1.88404	1.943017	1.697546	8.23E-08	6.07E-08	4.79E-09	2.13E-09	6.54E-11	3375	2883	2867	2524	1990
CCS	-0.56901	-0.34617	-0.70047	-0.37027	-0.5925	8.28E-08	0.000233	1.00E-11	0.000116	2.80E-08	3376	4962	1875	4898	3009
EIF3I	1.131698	1.029292	1.384838	1.254867	1.237285	8.30E-08	6.87E-08	1.58E-09	4.79E-09	3.19E-13	3377	2903	2648	2652	1341
SQLE	1.218476	1.292672	1.517479	1.179116	1.304788	8.30E-08	7.65E-09	1.87E-12	5.36E-08	6.03E-10	3378	2507	1659	3046	2338
GRB7	1.537169	1.717354	2.204003	1.45036	1.870199	8.36E-08	7.03E-09	1.65E-10	2.39E-10	6.76E-10	3380	2496	2294	2176	2361
RUFY2	-0.70301	-0.42789	-0.37331	-0.45979	-0.63571	8.36E-08	0.000478	0.00029	0.000613	1.19E-07	3381	5233	5563	5444	3316
TRDMT1	-0.83238	-0.51849	-0.62017	-0.83663	-0.73464	8.38E-08	0.000269	2.90E-05	2.19E-08	2.55E-06	3382	5016	4877	2906	3941
SUSD1	0.56532	0.605287	0.545637	0.599222	0.543521	8.41E-08	2.71E-08	2.99E-07	2.19E-06	3.01E-06	3383	2723	3754	3822	3980
ANKRD13/	-0.67718	-0.72173	-0.82008	-0.85458	-0.6504	8.42E-08	8.39E-08	2.10E-11	2.65E-09	9.50E-09	3384	2955	1987	2560	2804
PCED1A	-0.91219	-0.80751	-1.2857	-1.04064	-1.41531	8.48E-08	2.22E-06	3.30E-10	2.76E-06	2.27E-09	3385	3687	2404	3876	2565
LRBA	0.726984	0.550848	0.726367	0.583894	0.438743	8.51E-08	0.008295	1.55E-05	0.001403	0.009876	3387	6349	4714	5759	6558
POC1B	1.018985	1.093663	0.972292	1.446265	1.150884	8.51E-08	7.71E-06	1.31E-06	1.38E-09	3.17E-09	3388	3963	4105	2455	2614
SEC24D	-1.22661	-1.32848	-1.36276	-1.22394	-1.52591	8.55E-08	7.38E-08	1.10E-07	1.88E-07	7.89E-12	3389	2929	3537	3288	1726
SNAPC1	-0.82924	-0.75806	-0.73119	-0.83088	-0.84902	8.60E-08	1.58E-05	6.88E-08	1.74E-05	2.39E-08	3390	4144	3441	4360	2973
USP17L12	-1.00813	-0.95584	-1.64578	-1.14705	-0.939	8.73E-08	3.45E-07	4.88E-14	7.72E-08	1.30E-07	3391	3244	1241	3114	3330
PSMB2	0.906323	0.539084	1.002266	0.748845	0.580969	8.75E-08	0.000185	6.98E-09	6.77E-06	0.000649	3392	4889	2940	4097	5490
MAP2K4	-0.90401	-0.6318	-0.53088	-0.73721	-0.67012	8.78E-08	8.11E-05	1.61E-05	7.16E-06	1.60E-09	3393	4636	4722	4114	2503
DRD4	-0.66905	-0.58512	-0.88815	-0.72341	-0.5019	8.86E-08	7.99E-08	6.68E-12	4.47E-08	6.79E-05	3394	2944	1817	3025	4812
HMGCS2	2.268113	2.175618	1.875704	1.782303	2.068754	8.91E-08	2.08E-09	7.61E-08	6.30E-07	3.91E-09	3395	2283	3451	3550	2648
CPLX2	-0.68971	-0.4776	-0.94308	-0.61761	-0.82785	8.91E-08	6.49E-05	1.10E-08	0.000115	5.84E-09	3396	4558	3033	4891	2709
CEP250	0.961766	0.940219	0.801583	1.082496	0.509793	8.91E-08	2.77E-09	1.53E-06	9.19E-10	0.003621	3397	2335	4132	2385	6088
MPPE1	-0.72291	-0.72143	-0.84823	-0.84338	-0.81264	8.91E-08	1.92E-05	7.60E-07	3.58E-07	1.70E-08	3398	4201	3963	3420	2914
NEK7	-0.72247	-0.70829	-0.62656	-0.7482	-0.52544	8.98E-08	5.43E-08	1.17E-06	1.99E-09	0.000464	3399	2860	4078	2517	5386
MIX23	0.802201	1.095924	0.71093	0.902632	0.573806	9.10E-08	8.22E-11	9.16E-07	2.06E-08	0.001127	3400	1801	4012	2892	5682
ZDHHC20	0.910798	0.71719	0.866851	0.630518	1.172611	9.19E-08	4.42E-06	3.73E-08	1.42E-05	3.85E-10	3401	3833	3315	4310	2260
PPP2R3C	-0.73657	-0.78186	-0.76015	-0.93259	-0.59851	9.37E-08	6.22E-09	2.73E-08	1.49E-11	3.07E-05	3402	2478	3232	1823	4574
VPS35L	-1.03911	-0.9352	-0.80438	-0.953	-0.52339	9.37E-08	1.46E-05	5.50E-05	2.42E-05	0.000499	3403	4127	5059	4439	5405
DOC2B	-0.83431	-0.62599	-0.78541	-0.71146	-0.46232	9.40E-08	5.99E-05	7.80E-07	2.18E-06	0.00489	3404	4537	3970	3820	6214
CXXC1	-0.75704	-0.5886	-0.91682	-0.83956	-0.70195	9.47E-08	3.07E-07	6.71E-12	4.33E-10	8.63E-08	3406	3225	1820	2258	3246
BIRC3	-1.02145	-0.8988	-0.67896	-0.92587	-1.26777	9.55E-08	8.16E-08	0.000792	5.75E-07	4.87E-10	3407	2948	5886	3525	2304
MCRIP2	1.276797	1.560542	0.860335	1.554704	1.646688	9.59E-08	7.07E-12	7.52E-06	1.82E-11	4.61E-08	3408	1484	4509	1851	3108
PFDN4	0.870205	0.884195	0.918633	0.946276	0.627007	9.73E-08	2.21E-08	3.30E-08	1.45E-07	6.10E-05	3410	2684	3288	3236	4775
CILP	-0.8636	-0.59601	-0.61257	-0.70773	-1.09294	9.76E-08	0.001321	9.55E-05	0.000208	6.85E-09	3411	5619	5210	5071	2733
KCNK1	1.284154	1.368399	1.816344	1.31114	1.138983	9.85E-08	4.94E-08	8.31E-14	1.33E-07	1.15E-08	3413	2846	1300	3216	2847
TERF2IP	-0.59689	-0.64231	-0.70963	-0.47074	-0.4742	9.90E-08	6.97E-08	5.95E-09	6.99E-06	0.000367	3414	2907	2911	4110	5294
PRAMEF6	-0.86191	-0.71731	-1.14095	-1.03208	-1.05293	1.00E-07	1.50E-06	8.63E-10	1.46E-10	2.90E-09	3415	3585	2555	2107	2599
MRPL11	0.842068	0.916004	1.133352	1.073894	0.940278	1.00E-07	7.74E-10	2.54E-11	1.92E-11	1.50E-10	3416	2131	2014	1860	2110
RPL6	1.452777	1.492665	1.27016	1.753186	1.532995	1.00E-07	3.40E-09	9.47E-06	1.42E-14	1.89E-10	3417	2374	4572	1066	2147
CFB	1.441047	1.411601	1.732803	1.522259	1.486517	1.00E-07	5.54E-07	2.47E-09	1.48E-07	2.93E-08	3418	3331	2751	3239	3019
RAB11A	0.838126	0.753006	1.167865	0.917071	0.852053	1.01E-07	1.89E-07	7.60E-13	8.53E-09	9.70E-08	3420	3136	1562	2731	3269
PROSER1	1.137854	1.251204	1.332529	1.130798	1.558835	1.01E-07	2.14E-07	6.01E-09	4.06E-09	1.32E-10	3421	3162	2914	2632	2089
TNPO3	0.520751	0.803897	0.760401	0.679278	0.54369	1.03E-07	2.08E-07	1.32E-08	1.83E-06	0.000163	3422	3161	3079	3777	5065
P4HA2	-0.96944	-0.94401	-0.56814	-1.00071	-1.05222	1.03E-07	2.69E-08	6.83E-05	3.69E-10	6.18E-07	3423	2720	5118	2235	3647
PRMT3	0.723867	0.826639	0.771087	0.635991	0.738039	1.03E-07	3.86E-08	8.35E-08	5.85E-06	4.54E-07	3424	2794	3468	4059	3585
DYNC2I2	1.070742	1.171982	1.360527	1.161009	1.033729	1.03E-07	1.77E-07	1.23E-09	9.02E-08	5.16E-06	3425	3126	2619	3149	4115
TPP1	-1.07447	-1.21733	-0.82679	-0.91326	-1.34557	1.05E-07	3.48E-09	2.83E-06	2.36E-06	1.58E-11	3426	2376	4263	3839	1824
METTL7A	-0.94201	-0.6169	-0.94522	-0.77121	-1.0993	1.05E-07	0.000366	2.41E-07	6.14E-05	7.38E-09	3427	5139	3699	4739	2749
H2AX	0.981449	1.068624	1.237237	1.168488	1.275166	1.05E-07	3.32E-08	1.78E-09	3.71E-10	7.82E-10	3428	2757	2672	2238	2384
MAPRE1	1.027328	0.815387	1.064511	1.013103	0.839005	1.07E-07	2.73E-05	2.28E-07	6.88E-06	7.49E-07	3429	4308	3686	4102	3677
CENPC	-0.68404	-0.41018	-0.39786	-0.41236	-0.56941	1.08E-07	0.001257	0.000337	0.00117	9.67E-06	3431	5601	5601	5683	4272
PDHX	0.668157	0.817954	0.648999	0.736138	0.788419	1.08E-07	4.72E-08	5.80E-09	4.12E-08	2.10E-06	3432	2841	2903	3009	3889
POLR1D	1.125648	1.140904	1.003232	1.066952	1.301698	1.09E-07	3.12E-08	6.57E-07	2.93E-09	5.94E-08	3433	2741	3931	2579	3159

ARFGEF1	0.648718	0.556137	0.881853	0.665543	0.691341	1.09E-07	0.000444	1.71E-08	0.000177	1.00E-08	3434	5212	3134	5017	2815
SLC38A2	-0.95166	-1.05164	-0.73398	-0.63116	-0.90203	1.10E-07	4.11E-06	0.000598	0.002824	1.29E-05	3435	3824	5791	6022	4361
TRPC4AP	0.794665	0.600395	0.479686	0.694682	0.31176	1.11E-07	1.10E-05	3.38E-05	1.22E-05	0.006099	3436	4051	4928	4264	6317
GTF3A	1.447423	1.433041	1.077713	1.356836	1.675116	1.12E-07	1.88E-10	1.38E-06	1.15E-11	1.27E-09	3437	1914	4114	1782	2460
PICK1	0.792513	0.653474	1.057102	0.710495	1.017093	1.13E-07	7.69E-05	9.10E-09	7.87E-05	3.46E-14	3438	4613	2993	4800	1117
ARHGEF3	-0.66197	-0.63357	-0.74572	-0.67662	-0.58569	1.13E-07	6.51E-06	5.81E-10	2.88E-06	2.19E-07	3439	3923	2485	3893	3436
ZNF616	-0.79193	-0.40257	-0.75729	-0.70462	-0.56059	1.13E-07	0.005004	1.99E-07	2.12E-05	4.86E-05	3440	6096	3657	4408	4714
NOPCHAP	0.805336	0.806315	0.811573	0.803373	0.825178	1.14E-07	1.41E-06	1.17E-05	8.38E-08	1.14E-06	3441	3565	4632	3138	3763
FLCN	-0.85016	-0.87258	-1.07625	-1.02659	-0.99691	1.14E-07	7.66E-08	1.79E-09	6.51E-10	1.79E-10	3442	2936	2676	2329	2135
ARL2BP	-0.651	-0.90555	-0.81574	-0.86103	-0.65388	1.14E-07	5.33E-10	6.41E-09	8.58E-09	4.22E-08	3443	2077	2925	2733	3088
OTUD6B	0.804875	1.12573	1.011196	1.151835	0.724856	1.14E-07	1.82E-09	8.13E-09	1.25E-09	4.40E-07	3444	2258	2974	2427	3581
RPIA	0.864895	1.089046	0.907049	1.010864	1.161445	1.14E-07	9.15E-09	2.32E-09	1.10E-08	1.62E-14	3445	2540	2734	2776	1030
TPST2	-0.7932	-0.54648	-0.64983	-0.67838	-0.99743	1.15E-07	0.000134	1.37E-05	5.25E-05	1.37E-10	3446	4777	4677	4684	2099
DNAJC15	1.745012	1.546951	1.893145	1.675474	2.185841	1.15E-07	5.80E-09	1.71E-10	1.14E-08	5.20E-15	3447	2466	2301	2787	924
HES6	1.210054	1.650807	1.577596	1.657976	1.662189	1.15E-07	4.05E-09	8.71E-10	5.98E-10	5.10E-08	3448	2402	2557	2309	3129
OR2J3	-0.70491	-0.45503	-0.83972	-0.54065	-0.60189	1.16E-07	0.000341	1.18E-09	1.67E-05	3.84E-05	3449	5109	2616	4353	4648
HNF1A	0.668097	0.623075	0.936899	0.743893	0.736878	1.16E-07	2.96E-07	1.24E-09	1.49E-08	1.63E-07	3450	3215	2620	2830	3370
TMEM167	-0.66266	-0.53391	-0.6245	-0.6097	-0.83596	1.17E-07	2.05E-05	4.66E-07	4.52E-06	2.27E-09	3451	4218	3850	3994	2566
MVP	0.879219	0.40299	0.790946	0.903008	0.545499	1.19E-07	0.009482	1.45E-05	3.93E-07	0.000438	3452	6406	4697	3442	5360
COG5	0.67664	0.613967	0.872807	0.711167	0.743871	1.20E-07	2.38E-07	7.25E-11	1.55E-08	2.74E-08	3453	3180	2158	2837	3002
GANC	-0.77397	-0.62235	-0.65055	-0.63591	-0.7379	1.22E-07	0.000576	2.23E-05	0.000423	1.06E-07	3454	5312	4805	5324	3290
CLASP2	-0.47957	-0.33808	-0.64308	-0.64069	-0.41949	1.22E-07	4.62E-05	3.88E-07	3.97E-08	0.001721	3455	4455	3814	3002	5848
CCL3L3	-0.72423	-0.70127	-0.80755	-0.88884	-1.06041	1.25E-07	6.69E-05	9.06E-08	5.97E-06	1.79E-09	3457	4571	3489	4062	2522
RPL5	1.462844	1.442113	1.150934	1.787935	1.629514	1.26E-07	1.23E-08	1.62E-05	2.28E-13	4.34E-09	3458	2592	4723	1327	2672
SGK1	-1.98535	-2.29347	-1.75341	-2.06518	-2.39669	1.27E-07	5.54E-13	1.61E-08	9.47E-10	1.04E-12	3459	1205	3117	2390	1487
CFAP298	0.66788	0.92477	0.862588	0.75217	0.787641	1.27E-07	6.54E-08	2.94E-09	2.38E-06	8.79E-08	3460	2894	2781	3842	3250
MRPS30	0.811371	0.938476	0.875865	0.721746	1.065067	1.28E-07	7.98E-07	2.62E-06	7.55E-07	2.00E-09	3461	3424	4247	3581	2537
SORL1	1.122771	1.087493	0.971438	1.135112	0.803405	1.28E-07	8.89E-07	1.52E-08	2.27E-07	6.00E-05	3462	3452	3105	3320	4765
RECQL4	1.190254	1.592797	1.464909	1.685838	1.066771	1.29E-07	5.12E-11	5.49E-11	1.32E-11	1.30E-09	3463	1756	2121	1808	2468
HUWE1	0.814619	1.011992	0.877512	1.094661	0.946391	1.30E-07	3.15E-12	6.84E-08	1.43E-12	8.69E-13	3464	1372	3438	1550	1465
ARRDC3	-1.13352	-1.25366	-0.9854	-0.77707	-1.14592	1.30E-07	2.94E-07	0.000172	0.000831	1.53E-05	3466	3213	5382	5552	4402
ATP5PF	0.969738	0.826747	1.16633	0.959715	1.124621	1.31E-07	8.97E-06	1.94E-08	3.73E-07	4.14E-10	3467	4010	3164	3426	2276
ASAH1	-1.42086	-1.40537	-1.48804	-1.08748	-1.80124	1.31E-07	1.47E-09	2.17E-09	2.91E-06	6.81E-13	3468	2229	2718	3897	1440
RNF126	0.663414	0.826331	0.69128	0.75722	0.555804	1.31E-07	8.13E-08	6.44E-07	6.96E-08	1.79E-05	3469	2947	3927	3091	4438
SDHD	0.594577	0.602957	0.722404	0.622632	0.61108	1.31E-07	1.65E-06	1.76E-08	1.28E-06	4.57E-07	3470	3608	3139	3699	3587
ZMAT3	-1.00521	-0.59419	-1.0174	-0.83208	-0.96799	1.32E-07	0.004196	9.92E-09	0.000211	6.41E-09	3471	6035	3009	5082	2723
ZBTB7A	0.737103	0.756221	0.963547	0.98066	0.705694	1.32E-07	8.43E-10	1.70E-11	1.69E-13	3.65E-09	3472	2148	1938	1297	2633
CALM2	1.200205	0.633509	1.452133	0.859207	1.226924	1.32E-07	0.00016	2.89E-10	5.26E-05	1.72E-10	3473	4833	2379	4686	2130
INPP4A	-1.00496	-0.57733	-1.31369	-0.67937	-1.13274	1.32E-07	0.00032	3.40E-11	6.43E-05	2.48E-06	3474	5084	2063	4748	3934
CIC	-0.67257	-0.43402	-0.43837	-0.59435	-0.56593	1.33E-07	0.000231	0.000196	7.15E-08	7.54E-07	3475	4958	5434	3096	3681
VAMP8	0.714104	0.77233	1.011929	0.672876	1.17051	1.34E-07	4.46E-05	1.13E-07	5.08E-05	4.29E-09	3476	4445	3549	4678	2668
RBM12	0.813501	0.73451	0.735316	0.975927	0.391805	1.34E-07	5.82E-06	5.00E-05	4.69E-10	0.00609	3477	3898	5036	2272	6316
CSTF1	0.869539	0.922552	0.826975	1.160175	0.489454	1.35E-07	1.27E-05	4.22E-08	8.13E-10	0.005437	3478	4096	3336	2364	6268
DNAJB14	-0.58044	-0.71124	-0.8425	-0.95365	-0.88253	1.35E-07	1.18E-08	9.43E-09	5.05E-11	6.64E-08	3479	2583	3000	1978	3181
MAN1A1	-0.99362	-0.81419	-0.78954	-0.5987	-1.14	1.35E-07	1.03E-05	0.000148	0.00029	2.76E-08	3480	4036	5328	5206	3005
ISG20	-0.98487	-1.03411	-1.25958	-1.10525	-1.36264	1.35E-07	1.27E-09	5.77E-10	1.00E-09	1.15E-10	3481	2210	2484	2391	2072
OPA1	0.710722	0.765721	1.016498	0.807997	0.615664	1.38E-07	1.54E-06	7.48E-10	4.01E-07	8.41E-05	3482	3590	2534	3445	4867
MIB1	-0.82781	-0.79392	-0.84705	-0.89918	-0.93619	1.38E-07	8.87E-06	9.20E-09	3.87E-07	1.30E-08	3483	4006	2995	3439	2868
CCDC14	0.931921	1.126574	0.88002	1.147842	1.064607	1.39E-07	6.37E-11	4.07E-09	6.99E-11	1.06E-09	3485	1774	2843	2023	2436
RNF2	-0.57161	-0.64736	-0.31618	-0.64146	-0.38405	1.41E-07	7.06E-07	0.00386	3.07E-07	0.000536	3486	3391	6460	3383	5423
SCO2	-0.74462	-0.65899	-0.52869	-0.75162	-0.51448	1.41E-07	1.50E-07	2.34E-06	1.38E-07	0.002682	3487	3086	4219	3225	6003
SCX	0.865737	1.047457	1.211665	0.855458	0.971763	1.43E-07	5.65E-08	2.94E-11	5.05E-07	6.43E-08	3488	2867	2047	3495	3174
SLC5A6	1.323734	1.344125	1.275405	1.498578	1.117242	1.46E-07	5.71E-08	4.21E-09	6.85E-10	5.64E-06	3490	2870	2850	2335	4136
RNF207	-0.65892	-0.63508	-0.77214	-0.48073	-0.68646	1.46E-07	5.47E-07	5.85E-10	0.001109	0.000628	3491	3328	2489	5666	5478
ATN1	-0.69125	-0.55671	-0.69946	-0.74247	-0.67941	1.47E-07	2.92E-05	1.18E-08	9.75E-09	2.04E-07	3492	4324	3052	2760	3418
SDCCAG8	-0.70329	-0.87688	-0.7769	-0.85949	-0.80511	1.48E-07	2.68E-09	2.91E-08	1.71E-08	3.47E-10	3493	2324	3252	2859	2240
MMP15	1.318688	1.43684	1.863748	1.385663	1.745951	1.49E-07	8.35E-09	3.36E-14	1.61E-08	2.34E-13	3494	2524	1206	2847	1312
LARGE1	-0.54888	-0.49574	-0.59566	-0.53817	-0.70668	1.49E-07	2.18E-05	9.41E-09	1.88E-06	3.41E-05	3495	4238	2999	3792	4603
SND1	1.096172	1.030022	0.812588	1.197606	1.10383	1.50E-07	5.91E-07	4.04E-05	2.12E-10	4.54E-08	3497	3352	4974	2159	3105
VRK1	0.839106	1.06531	1.031271	0.899623	0.903642	1.50E-07	2.52E-09	1.87E-10	4.99E-08	2.22E-07	3498	2314	2313	3038	3439
TNNC2	1.125044	1.454538	1.119202	1.631736	1.212094	1.51E-07	5.38E-11	8.17E-07	3.46E-11	3.87E-08	3499	1758	3986	1926	3064
CASZ1	0.781251	0.66307	1.056998	0.777361	1.093155	1.52E-07	2.23E-05	3.78E-11	3.68E-07	1.45E-12	3500	4246	2072	3423	1529
TBL1XR1	0.642684	0.717528	0.694391	0.610455	0.659235	1.53E-07	2.48E-07	1.46E-07	1.10E-05	2.60E-08	3503	3185	3599	4242	2988
ANKRD30I	-0.90517	-0.54364	-0.83041	-0.74764	-0.53707	1.54E-07	0.000494	4.68E-07	3.46E-06	0.000841	3504	5247	3853	3927	5577
GNG5	0.655873	0.73972	0.681763	0.80272	0.882392	1.54E-07	8.16E-11	3.31E-08	3.66E-10	1.05E-09	3505	1797	3289	2233	2431
TMEM263	-1.23688	-1.13926	-1.05502	-1.07575	-1.34682	1.56E-07	2.79E-07	5.82E-07	2.06E-06	8.10E-12	3506	3204	3897	3809	1733
ZNF821	-0.69928	-0.45704	-0.85109	-0.60546	-0.94909	1.56E-07	6.95E-05	3.88E-09	1.10E-05	2.33E-10	3507	4585	2837	4244	2189
BAGE5	-1.05182	-0.78506	-1.41475	-1.12252	-1.03182	1.57E-07	2.66E-05	2.98E-13	2.39E-07	1.58E-07	3508	4297	1461	3333	3366
CPNE1	1.465045	1.559991	1.178816	1.708104	1.459692	1.58E-07	1.21E-08	1.94E-05	6.52E-09	1.30E-08	3509	2589	4773	2693	2867
HS6ST1	-1.19753	-1.10092	-0.98089	-0.98095	-1.00817	1.60E-07	1.16E-08	5.19E-09	1.02E-06	7.11E-10	3511	2577	2882	3647	2369
H1-5	2.213161	1.919049	2.546213	2.26159	2.577247	1.60E-07	8.23E-08	4.08E-09	4.24E-10	5.77E-11	3512	2952	2844	2252	1967
TBC1D13	-0.55829	-0.57862	-0.56988	-0.49955	-0.39787	1.62E-07	9.19E-08	6.71E-11	3.06E-05	0.00026	3513	2977	2148	4524	5197
OAZ1	0.876742	0.539587	0.756374	0.80493	0.859044	1.62E-07	0.001013	3.77E-05	1.11E-05	5.03E-06	3515	5513	4952	4246	4108

VPS45	-0.56566	-0.65514	-0.68041	-0.7197	-0.49711	1.63E-07	5.40E-08	8.05E-09	6.88E-08	0.000152	3516	2859	2968	3090	5048
REL	-0.52687	-0.49548	-0.28557	-0.32995	-0.4518	1.63E-07	3.69E-07	0.004207	0.001516	0.000722	3517	3260	6498	5777	5536
UBE2N	0.73836	0.92857	0.848295	0.903606	0.968354	1.63E-07	7.57E-08	1.65E-08	1.12E-07	1.19E-11	3518	2932	3124	3190	1790
C9orf152	0.910037	0.929951	0.8736	1.059254	1.030438	1.64E-07	1.10E-08	1.66E-08	6.49E-09	3.82E-09	3519	2566	3127	2692	2645
SOHLH1	-0.75077	-0.62975	-0.84244	-0.8064	-0.7805	1.65E-07	2.05E-06	4.21E-10	1.51E-08	7.66E-09	3520	3666	2438	2834	2759
MRPL38	0.868482	0.680519	0.747697	0.741887	0.920762	1.65E-07	0.000132	1.26E-05	9.33E-05	7.65E-09	3521	4774	4667	4837	2758
ST6GALNA	-1.79888	-1.64558	-1.65018	-1.71943	-1.92148	1.67E-07	1.16E-07	2.95E-08	1.28E-09	2.96E-09	3522	3030	3255	2433	2604
HNRNPUL	0.733361	0.62612	0.902598	0.596068	0.915442	1.67E-07	3.64E-06	3.45E-09	9.10E-06	7.60E-12	3523	3795	2815	4180	1722
OR6F1	-0.73984	-0.7059	-0.48856	-0.88226	-0.72898	1.68E-07	1.04E-07	3.32E-05	9.77E-13	7.01E-08	3524	3005	4924	1497	3197
LLGL1	-0.63597	-0.63646	-0.83124	-0.65433	-0.80184	1.68E-07	4.72E-06	2.06E-09	1.79E-05	2.12E-06	3525	3851	2711	4370	3896
EPS15	-0.61638	-0.51428	-0.4383	-0.63731	-0.74432	1.69E-07	2.09E-06	6.51E-05	2.30E-05	8.72E-05	3526	3671	5106	4422	4882
GAB2	-0.89513	-0.98771	-0.83139	-0.96781	-1.00908	1.70E-07	4.62E-08	1.84E-08	3.26E-07	7.20E-10	3527	2832	3147	3396	2373
TFE3	-0.85534	-0.77501	-1.0421	-0.76168	-1.00611	1.73E-07	1.28E-06	4.16E-11	1.48E-06	7.60E-09	3528	3543	2087	3729	2756
EPS8	1.003338	0.922074	1.021501	1.061044	1.244068	1.73E-07	2.74E-07	4.90E-11	1.15E-08	6.42E-10	3529	3202	2106	2789	2350
MRPL36	0.505078	0.614171	0.49779	0.66782	0.387473	1.73E-07	8.62E-07	1.40E-05	1.64E-07	0.000975	3530	3438	4684	3261	5620
CHTF18	1.120458	1.176678	0.952031	1.102378	0.912452	1.75E-07	8.11E-09	2.47E-08	3.20E-08	1.36E-06	3531	2520	3207	2965	3804
DCUN1D5	0.809454	0.905035	0.730382	0.755663	0.41266	1.75E-07	1.14E-09	1.01E-07	5.07E-07	0.001187	3532	2193	3518	3498	5694
MORF4L1	-0.87342	-0.97114	-0.89523	-0.93794	-0.87664	1.76E-07	6.59E-08	1.11E-06	2.23E-08	1.24E-06	3533	2897	4060	2908	3783
NCAPG2	0.832351	0.956783	0.881811	0.899115	0.998418	1.76E-07	1.13E-07	4.37E-09	2.72E-08	1.01E-08	3534	3020	2854	2934	2817
SEMA4B	1.195267	1.192657	1.602894	1.223691	1.230577	1.77E-07	4.05E-10	7.89E-11	6.07E-10	2.28E-09	3535	2041	2170	2312	2570
SLC37A1	1.466352	1.294116	1.998424	1.574439	1.636196	1.77E-07	2.20E-07	2.12E-12	1.34E-12	1.22E-11	3536	3168	1675	1538	1791
COX7A2L	0.698645	0.684276	1.098802	0.896924	0.879529	1.79E-07	4.02E-06	9.98E-10	9.63E-08	6.03E-10	3537	3821	2580	3160	2339
OR10A6	-0.62746	-0.44865	-0.76375	-0.54494	-0.84505	1.80E-07	0.000163	1.77E-09	4.70E-05	9.96E-11	3538	4841	2671	4656	2053
ZBTB1	-0.72787	-0.65406	-0.44091	-0.59615	-0.73573	1.82E-07	6.69E-07	0.003481	0.000198	1.16E-07	3539	3377	6422	5053	3310
TMEM82	-0.531	-0.45992	-0.6912	-0.57011	-0.48868	1.82E-07	3.00E-05	7.07E-07	1.16E-05	5.58E-05	3540	4333	3948	4256	4747
RSF1	-0.65759	-0.48304	-0.87627	-0.63151	-0.5822	1.83E-07	2.48E-05	2.11E-10	2.78E-06	1.64E-06	3541	4270	2333	3880	3842
GARS1	0.970494	0.914358	0.993072	0.765865	0.991684	1.83E-07	5.05E-07	5.84E-08	3.87E-06	1.01E-07	3542	3312	3403	3957	3278
NUP205	0.659334	0.860256	0.727559	0.805058	0.640453	1.83E-07	1.10E-10	1.52E-09	8.43E-11	1.16E-06	3543	1842	2644	2045	3768
CARD6	-0.99562	-1.10765	-0.87495	-1.12927	-0.74832	1.85E-07	2.92E-10	3.50E-08	5.45E-10	2.19E-05	3544	1979	3303	2291	4488
NBPF26	-0.65657	-0.59127	-0.79193	-0.71644	-0.78835	1.86E-07	2.06E-05	9.90E-08	1.71E-06	8.66E-10	3545	4219	3515	3760	2406
ELF1	0.961874	0.925545	1.136721	0.741151	0.860597	1.86E-07	1.08E-06	3.19E-09	1.52E-06	9.99E-05	3546	3504	2803	3736	4926
CXCL8	-2.47787	-2.57011	-2.55622	-3.04138	-3.53772	1.87E-07	2.67E-06	2.90E-06	7.99E-08	1.40E-09	3547	3729	4269	3125	2482
HMGB2	1.571672	1.12373	1.533438	1.118421	1.662291	1.87E-07	1.80E-05	6.26E-08	8.94E-06	7.85E-10	3548	4185	3416	4172	2385
SURF6	0.78867	1.063593	1.361489	1.151415	0.966427	1.87E-07	1.26E-09	3.03E-12	1.43E-10	7.80E-10	3549	2208	1724	2106	2383
ABCF1	0.716189	0.71616	0.89801	0.668102	0.823189	1.88E-07	1.05E-05	2.98E-09	9.49E-06	2.10E-09	3550	4040	2785	4197	2547
TRAFD1	-0.75114	-0.52932	-0.59037	-0.40934	-0.51849	1.89E-07	2.49E-05	4.14E-06	0.001296	0.000135	3551	4273	4358	5719	5013
ZBTB37	-0.66131	-0.74798	-0.93549	-0.76361	-0.89204	1.89E-07	6.57E-09	9.44E-11	3.93E-07	1.91E-12	3552	2488	2207	3443	1547
MRPL32	0.729262	0.586498	0.556448	0.626943	0.550763	1.89E-07	6.16E-06	1.03E-05	2.92E-05	1.25E-05	3553	3911	4597	4505	4346
IDH2	1.36905	0.941606	1.464174	1.558782	1.190811	1.91E-07	3.23E-06	2.94E-08	3.53E-11	1.15E-06	3554	3775	3254	1932	3765
STRBP	0.697717	0.940435	0.646774	0.872794	0.861248	1.91E-07	2.67E-11	3.75E-07	2.10E-10	1.95E-08	3555	1659	3805	2156	2938
ATAD2B	-0.62234	-0.5602	-0.5271	-0.7324	-0.59211	1.92E-07	1.44E-06	4.95E-06	2.16E-10	2.20E-06	3556	3573	4409	2162	3906
LYNX1	-0.78836	-0.3898	-0.68894	-0.68772	-0.60445	1.92E-07	0.002863	3.45E-06	3.81E-06	1.80E-05	3557	5879	4307	3948	4440
SDC3	-1.03583	-0.96011	-0.92752	-0.95792	-0.75692	1.94E-07	2.39E-05	6.08E-07	2.41E-05	0.00024	3558	4265	3916	4437	5175
GDA	1.410864	1.562999	1.554279	1.200023	0.86041	1.94E-07	2.06E-09	2.55E-07	7.21E-08	0.000627	3559	2281	3709	3098	5477
ABTB1	-0.92209	-0.64093	-1.00469	-0.7231	-1.06111	1.95E-07	0.000179	2.84E-06	4.41E-05	1.69E-08	3560	4877	4265	4639	2912
RGS14	-0.74892	-0.50371	-1.27202	-0.78088	-1.02391	1.96E-07	7.80E-05	2.77E-11	2.42E-05	7.53E-08	3561	4619	2030	4440	3219
CDCA8	0.532946	0.487509	0.318336	0.37091	0.307751	1.97E-07	7.80E-05	0.007906	0.00312	0.004604	3562	4618	6751	6071	6188
KIAA0513	-0.97408	-0.79858	-0.88493	-0.94113	-0.55112	1.98E-07	4.46E-07	1.56E-06	5.51E-11	0.003485	3563	3291	4136	1986	6076
SATB1	-1.05528	-0.55704	-0.81688	-0.70683	-0.56162	2.01E-07	0.007394	0.000839	0.001483	0.001001	3564	6283	5912	5771	5632
RAD23A	0.798648	0.710608	0.84852	0.844938	0.636855	2.01E-07	7.50E-05	1.54E-06	5.50E-06	3.16E-05	3565	4607	4134	4046	4581
C1QTNF1	-0.8771	-0.69034	-0.67869	-1.00349	-0.66496	2.02E-07	2.41E-06	2.05E-05	6.27E-11	6.85E-05	3566	3702	4783	2005	4816
RPL13	1.885555	1.676567	1.445668	2.07982	2.017198	2.04E-07	5.29E-08	1.65E-05	4.47E-13	4.03E-10	3567	2852	4728	1412	2268
MRPS15	0.646809	0.804744	0.782523	0.811614	0.467613	2.06E-07	2.80E-10	4.03E-11	5.62E-12	0.002065	3568	1973	2081	1687	5918
CHCHD10	1.391298	1.877487	1.947754	1.902675	1.65544	2.07E-07	7.79E-16	5.87E-14	9.89E-16	2.81E-12	3569	680	1260	844	1609
GRIA4	-0.92578	-0.53564	-0.7448	-0.71461	-0.84909	2.08E-07	0.002125	9.33E-05	6.67E-06	1.59E-05	3571	5793	5205	4090	4412
ZNRF3	0.756163	1.100187	1.020047	1.076238	0.887403	2.08E-07	5.19E-09	1.51E-08	2.73E-08	1.05E-09	3572	2445	3103	2935	2434
JAK1	-1.04826	-0.90934	-0.69672	-0.69709	-0.89059	2.08E-07	4.32E-08	2.38E-05	5.45E-05	1.64E-06	3573	2819	4827	4699	3840
UFC1	0.680198	0.687679	0.675872	0.760954	0.990393	2.08E-07	1.57E-05	1.67E-07	9.11E-06	1.59E-09	3574	4142	3624	4181	2502
FAM107B	-1.15135	-0.90432	-1.32184	-1.18803	-1.65901	2.09E-07	7.52E-06	4.04E-07	4.28E-06	3.54E-12	3575	3956	3823	3984	1633
CAPN7	-0.54861	-0.53413	-0.44544	-0.60146	-0.75451	2.09E-07	4.23E-07	1.25E-05	6.87E-09	1.86E-12	3576	3279	4665	2703	1545
WWC3	-0.80413	-0.57273	-0.52939	-0.3985	-0.55949	2.10E-07	4.93E-06	2.29E-05	0.002353	2.03E-05	3578	3859	4816	5948	4470
NBPF14	-0.80766	-0.49039	-0.7937	-0.677	-0.67801	2.11E-07	1.36E-06	3.60E-09	3.13E-08	3.91E-07	3579	3555	2823	2958	3563
MME	-1.80142	-1.21763	-1.43833	-1.79957	-1.72716	2.11E-07	0.000884	8.68E-07	1.40E-05	8.82E-07	3580	5460	3998	4307	3706
EFTUD2	0.884051	0.822248	0.774646	0.770361	0.691099	2.12E-07	2.66E-06	3.26E-06	7.90E-08	7.29E-07	3582	3727	4295	3120	3670
ARHGEF16	0.920277	0.855321	1.340822	0.924005	1.008184	2.13E-07	8.68E-06	2.42E-11	1.69E-06	2.13E-09	3583	3997	2008	3754	2550
ARHGAP5	0.712064	0.553202	1.131271	0.744607	1.029424	2.16E-07	0.000436	5.66E-08	0.000209	4.20E-08	3584	5202	3396	5076	3086
GNA11	0.747125	0.901838	1.168122	0.967814	0.915745	2.17E-07	3.77E-09	7.80E-12	1.71E-11	1.00E-09	3585	2388	1840	1839	2425
DPY30	0.75511	0.85476	0.735467	0.915646	0.822999	2.17E-07	6.66E-10	8.31E-07	7.92E-11	1.11E-10	3587	2106	3990	2037	2066
SATL1	-0.79342	-0.52011	-0.56908	-0.58156	-0.60196	2.18E-07	0.000667	0.000143	0.000275	5.11E-06	3588	5363	5317	5190	4113
PPP1CC	0.837115	0.864834	1.066842	1.070815	0.711512	2.19E-07	4.52E-08	3.16E-07	3.89E-10	1.66E-05	3589	2826	3772	2242	4422
MAP3K6	-0.95648	-1.00476	-0.74092	-0.79473	-0.89884	2.19E-07	1.72E-10	2.73E-05	1.05E-06	5.31E-08	3590	1905	4866	3655	3142
PSMF1	0.77895	0.791134	0.991211	0.734368	0.756981	2.19E-07	4.79E-09	1.41E-11	5.85E-08	6.18E-07	3591	2429	1915	3058	3646
MRPS5	0.720505	1.012373	0.765388	1.051978	0.649007	2.20E-07	2.39E-09	4.28E-08	1.18E-08	1.29E-05	3592	2303	3342	2792	4359

C7	-0.72506	-0.49517	-0.7485	-0.76901	-1.18722	2.20E-07	7.71E-05	4.14E-06	1.16E-07	8.12E-10	3593	4614	4356	3195	2393
XPO5	0.92149	1.194476	1.194301	1.027019	0.807762	2.20E-07	4.05E-08	1.37E-09	1.95E-06	6.40E-05	3594	2804	2632	3801	4790
SLC9A3	1.707337	2.289876	2.078619	2.03956	1.745748	2.22E-07	6.38E-12	1.98E-09	1.17E-08	2.07E-07	3595	1470	2701	2790	3421
GINS1	0.972913	1.207042	1.223377	1.155938	0.976772	2.22E-07	2.48E-11	2.03E-10	1.30E-09	9.33E-08	3596	1649	2323	2434	3263
PTDSS1	0.833144	0.774723	1.176181	0.724004	0.944696	2.27E-07	1.58E-05	1.11E-09	0.000113	2.80E-08	3597	4143	2600	4885	3012
DNAJA4	0.641722	0.786466	0.851627	0.905279	0.936974	2.27E-07	6.65E-07	5.85E-10	1.38E-09	1.04E-07	3598	3375	2491	2453	3287
MAPKAPK2	-0.83582	-1.01769	-1.17391	-1.14944	-0.90268	2.28E-07	1.45E-10	1.33E-11	9.30E-09	1.84E-06	3599	1884	1907	2748	3862
SUMO1	0.555276	0.805506	1.070892	0.906323	0.835314	2.28E-07	2.52E-11	2.69E-11	1.98E-09	5.42E-10	3600	1651	2026	2515	2317
NDUFS3	0.798929	0.841001	0.961221	0.924693	0.70036	2.29E-07	5.83E-07	1.07E-07	4.27E-07	0.000146	3601	3345	3529	3461	5040
VPS13B	-0.58337	-0.30137	-0.45402	-0.34609	-0.47453	2.30E-07	0.005083	4.19E-06	0.002609	1.20E-05	3602	6104	4359	5989	4336
BRCA1	0.992857	1.094991	1.167732	1.236117	0.896135	2.30E-07	6.73E-07	2.44E-08	4.53E-09	1.35E-07	3603	3379	3206	2647	3336
AXIN1	1.006917	1.175971	1.320512	1.213865	1.19127	2.30E-07	1.64E-07	4.77E-12	6.58E-09	1.67E-10	3604	3100	1773	2695	2123
DRG1	0.569685	0.581868	0.660665	0.627944	0.55216	2.30E-07	3.25E-07	2.48E-10	7.15E-07	5.60E-08	3605	3234	2356	3573	3147
CAMSAP2	-0.95313	-1.19458	-1.0551	-1.25393	-1.07378	2.34E-07	3.06E-09	7.69E-09	3.13E-10	7.92E-09	3606	2353	2959	2212	2765
RAB11FIP2	-0.59265	-0.46913	-0.54326	-0.54203	-0.66118	2.34E-07	5.27E-05	4.90E-06	2.42E-06	1.13E-06	3607	4503	4407	3846	3761
G0S2	-1.4461	-1.80329	-1.93464	-1.77088	-2.02099	2.35E-07	4.07E-08	1.49E-12	6.25E-08	1.87E-10	3608	2806	1638	3075	2143
GDF1	-0.91333	-0.63439	-1.34086	-0.96221	-1.01201	2.36E-07	0.000185	1.32E-11	2.77E-06	7.70E-08	3609	4884	1906	3878	3224
RAPGEF2	-0.72386	-1.01509	-0.75672	-0.8494	-0.61586	2.37E-07	8.66E-08	4.92E-06	1.75E-06	7.53E-05	3610	2963	4408	3766	4838
CDHR1	1.530344	2.040781	1.611872	1.916437	1.952053	2.37E-07	2.22E-11	1.96E-07	1.17E-10	2.17E-08	3611	1628	3656	2079	2957
CKAP2	1.171845	1.283628	0.776468	0.809235	0.815735	2.39E-07	4.69E-11	1.91E-05	9.01E-07	0.000954	3612	1743	4768	3610	5612
JUN	0.991014	1.050796	0.85951	1.192172	1.160603	2.39E-07	7.15E-08	2.04E-07	1.33E-07	7.81E-06	3613	2918	3663	3219	4217
GJC2	-0.73481	-0.39981	-0.67147	-0.54838	-0.56362	2.40E-07	0.006187	2.73E-05	0.000938	3.15E-05	3614	6195	4867	5600	4579
SIN3B	-0.70656	-0.44391	-0.56343	-0.59024	-0.53658	2.40E-07	0.000663	3.11E-06	2.65E-05	9.13E-05	3616	5362	4291	4471	4898
ARHGEF38	1.0005	0.643487	1.03474	0.854354	1.110568	2.41E-07	2.22E-05	3.62E-09	1.13E-06	3.33E-11	3617	4244	2824	3671	1906
SPCS1	0.943697	1.009188	0.857103	1.092299	1.077732	2.46E-07	2.50E-08	8.31E-07	2.24E-11	2.24E-09	3618	2701	3991	1883	2560
NELFA	0.606425	0.621897	0.785626	0.374493	0.646402	2.46E-07	3.88E-06	7.89E-11	0.001497	0.000204	3619	3811	2171	5774	5130
MTARC1	0.949975	1.014769	0.973612	0.92852	0.639474	2.48E-07	4.38E-09	1.03E-08	8.14E-07	0.000233	3620	2410	3017	3596	5163
JRK	-0.54687	-0.45819	-0.51571	-0.45887	-0.63377	2.48E-07	7.72E-06	6.10E-07	6.68E-05	1.29E-07	3621	3965	3917	4758	3328
EVI5	-0.67643	-0.60955	-0.73653	-0.67626	-0.50937	2.52E-07	1.11E-06	5.81E-09	2.96E-08	0.00039	3622	3509	2904	2949	5311
SUPT4H1	1.109045	1.339609	1.093458	1.118072	0.973468	2.53E-07	2.97E-10	3.34E-07	1.56E-09	2.89E-06	3623	1986	3779	2478	3964
CAD	0.642597	0.85555	0.728387	0.773379	0.498484	2.55E-07	7.94E-10	2.04E-08	2.28E-10	0.000438	3624	2142	3170	2167	5363
PIGU	1.167636	0.770677	1.035063	0.955725	0.846167	2.56E-07	2.87E-05	4.81E-08	9.13E-07	6.26E-07	3625	4320	3361	3613	3648
YKT6	0.693143	0.660773	0.623272	0.567939	1.026833	2.57E-07	8.04E-06	3.04E-07	0.000122	6.90E-08	3626	3969	3761	4917	3193
RNASE13	-0.6785	-0.39768	-0.48896	-0.68236	-0.67966	2.59E-07	0.000513	0.000172	4.29E-08	2.17E-06	3627	5264	5383	3016	3901
FRZB	-1.84041	-1.98124	-1.74362	-1.95748	-1.18962	2.60E-07	2.54E-09	2.78E-08	3.29E-07	0.001274	3628	2315	3237	3402	5723
KARS1	0.981172	0.822438	0.966044	0.961201	1.090333	2.60E-07	5.99E-06	8.75E-08	1.90E-08	3.22E-08	3629	3905	3481	2878	3030
ADAM8	-1.1845	-1.63012	-1.8287	-1.88305	-1.70174	2.61E-07	2.47E-11	2.46E-12	6.03E-13	2.27E-10	3631	1647	1688	1444	2185
NADK2	0.714466	0.732754	0.444661	0.613818	0.61655	2.62E-07	7.97E-08	0.002368	4.35E-05	5.38E-05	3632	2943	6274	4633	4738
ANKRD13D	-0.7492	-0.52589	-0.79924	-0.68338	-1.01218	2.62E-07	0.000219	5.08E-09	1.62E-05	3.72E-12	3633	4938	2877	4345	1637
RPLP1	1.239485	1.24095	1.0313	1.19884	1.392799	2.67E-07	3.95E-08	2.49E-05	1.79E-09	4.18E-09	3635	2795	4846	2499	2659
ZWINT	0.890827	1.054909	1.548639	1.124875	1.323588	2.68E-07	9.76E-07	2.15E-13	1.85E-06	1.59E-11	3636	3474	1426	3785	1825
ZMYND8	1.000157	1.142096	0.876351	1.453372	0.875108	2.70E-07	4.73E-07	5.31E-05	2.37E-09	5.66E-06	3637	3302	5050	2545	4139
PAOX	-0.69438	-0.77347	-1.19494	-0.94565	-1.26784	2.70E-07	1.39E-07	2.57E-12	8.35E-07	1.28E-09	3638	3068	1698	3602	2464
TFCP2L1	1.090118	1.03788	1.341886	1.164641	1.782621	2.70E-07	4.02E-06	1.03E-08	2.39E-07	7.62E-17	3639	3820	3016	3332	619
GOT1	0.546847	0.710521	0.825219	0.69543	0.806469	2.73E-07	3.60E-08	3.67E-08	1.89E-09	2.03E-09	3640	2778	3310	2508	2539
USP17L11	-1.13414	-1.01782	-1.65884	-1.20719	-1.40299	2.77E-07	1.03E-06	3.34E-10	6.18E-07	1.76E-11	3642	3487	2405	3544	1839
OR6B1	-0.76888	-0.40911	-0.6923	-0.72945	-0.73695	2.77E-07	0.003185	7.92E-05	3.43E-05	0.000543	3643	5920	5157	4562	5425
SNX33	-0.9215	-0.51759	-0.50242	-0.65156	-0.72732	2.78E-07	0.006333	0.002156	0.000894	2.16E-05	3645	6203	6233	5582	4486
TOR1AIP1	-0.73613	-0.84085	-0.8987	-0.98809	-0.65333	2.80E-07	1.00E-07	1.17E-07	9.93E-09	5.37E-06	3647	2997	3557	2764	4127
KDM1B	1.154253	1.054719	1.588408	1.194526	1.159253	2.80E-07	2.47E-06	1.96E-11	1.42E-06	1.14E-06	3648	3708	1971	3716	3762
SPTBN1	1.249616	0.900693	1.538706	0.968634	1.105709	2.81E-07	0.000183	9.93E-09	0.000151	3.00E-08	3649	4880	3010	4976	3024
SLC6A6	-1.41747	-1.23815	-1.27975	-1.10112	-0.81886	2.81E-07	7.77E-07	1.87E-08	0.00015	3.11E-05	3650	3412	3155	4973	4576
CDK4	1.10841	0.954418	0.833281	1.015935	1.174334	2.81E-07	1.30E-06	0.000109	1.51E-09	5.78E-09	3651	3549	5241	2475	2705
CPSF1	0.786922	1.246298	1.024355	1.219281	0.922294	2.83E-07	7.37E-11	1.41E-08	1.28E-11	6.71E-11	3652	1786	3093	1804	1996
RPL34	1.332981	1.155212	1.050467	1.284976	1.445541	2.84E-07	1.09E-06	8.40E-05	3.94E-10	1.09E-08	3653	3505	5176	2244	2833
NCOA1	-0.69467	-0.6675	-0.5039	-0.62948	-0.43956	2.85E-07	2.66E-07	7.90E-06	8.82E-08	3.37E-06	3654	3195	4524	3146	4003
DEPDC7	-0.91822	-0.63646	-0.79639	-0.78068	-0.49526	2.85E-07	4.21E-05	2.48E-06	1.39E-06	0.000925	3655	4433	4235	3711	5607
LTBR	0.877984	0.892417	1.291385	1.159667	0.735731	2.88E-07	1.67E-07	1.86E-10	4.82E-09	5.39E-05	3659	3108	2312	2653	4739
UTF1	-0.855	-0.57773	-1.21195	-0.6989	-1.1024	2.92E-07	2.36E-05	1.13E-11	9.23E-06	1.18E-10	3662	4263	1889	4187	2075
RAB11FIP5	-0.56147	-0.40921	-0.58103	-0.36818	-0.64066	2.92E-07	0.000216	4.21E-07	0.00301	4.69E-07	3663	4933	3831	6054	3593
CALCOCO1	-0.70491	-0.39502	-0.60022	-0.3853	-0.58321	2.92E-07	0.003117	9.16E-06	0.003997	1.98E-06	3664	5911	4567	6158	3876
CXXC5	1.185362	1.387982	0.799214	1.347067	1.189036	2.93E-07	9.40E-11	0.000738	1.12E-08	1.10E-09	3665	1822	5862	2779	2443
TIMM8A	0.825596	0.996216	0.442155	0.900372	0.493842	2.93E-07	2.97E-10	8.54E-05	1.53E-08	8.46E-05	3666	1985	5186	2835	4873
MEX3C	-0.53754	-0.48829	-0.50059	-0.45099	-0.55285	2.97E-07	4.60E-05	3.56E-06	2.79E-05	6.85E-09	3667	4454	4318	4491	2732
SLC44A3	1.170849	1.138318	1.305998	1.434059	1.470547	2.97E-07	1.37E-08	6.52E-08	9.54E-11	1.87E-09	3668	2610	3431	2059	2529
CIAO2A	0.674755	0.598454	0.430404	0.550798	0.413449	2.97E-07	2.87E-06	0.000806	1.24E-06	0.000561	3669	3749	5894	3693	5439
PIKFYVE	-0.59645	-0.56011	-0.53025	-0.47812	-0.63469	2.98E-07	9.25E-07	1.25E-06	2.34E-05	7.27E-09	3670	3462	4093	4425	2745
COMMD6	1.1478	1.316771	1.358422	1.385781	1.533382	2.98E-07	2.36E-10	1.02E-10	6.35E-10	7.51E-15	3671	1951	2222	2322	958
PNPT1	0.915967	0.97629	0.921543	0.9259	0.942772	3.01E-07	2.69E-07	5.14E-07	3.61E-08	8.50E-09	3672	3196	3871	2985	2777
TMEM14C	0.969548	0.742799	0.988729	0.763512	1.125812	3.03E-07	9.20E-06	2.37E-08	8.51E-06	1.82E-12	3673	4013	3192	4162	1544
ATL1	-0.6252	-0.36445	-0.41637	-0.37279	-0.45394	3.05E-07	0.002045	3.41E-05	0.003301	3.49E-05	3676	5776	4931	6092	4613
DMD	-0.8667	-0.47258	-1.06627	-0.79665	-1.19568	3.06E-07	0.000887	1.02E-08	1.39E-05	2.63E-12	3677	5463	3015	4303	1598
HADHA	0.711569	0.720864	0.990861	0.935343	0.931612	3.09E-07	5.07E-08	1.99E-11	1.13E-08	3.41E-12	3679	2848	1975	2783	1630

RASSF1	-0.75362	-0.76757	-0.77956	-0.69927	-0.81	3.10E-07	2.45E-06	3.09E-08	3.67E-06	6.27E-10	3681	3706	3265	3940	2344
SVIL	-1.11104	-0.76156	-0.54892	-0.63709	-0.6885	3.11E-07	5.95E-05	0.001451	0.00161	0.001871	3682	4534	6081	5803	5886
HSD17B11	1.046699	0.979474	1.281975	1.004919	1.059565	3.13E-07	1.04E-07	9.14E-10	4.31E-08	3.24E-09	3684	3004	2565	3017	2616
ISG20L2	0.664188	0.613781	0.589191	0.703586	0.600079	3.16E-07	1.05E-05	1.78E-05	3.47E-07	9.99E-05	3685	4039	4750	3411	4927
B3GALT1	-0.65428	-0.32534	-0.67298	-0.58071	-0.7417	3.16E-07	0.004033	6.79E-05	1.22E-05	7.40E-08	3686	6019	5115	4265	3216
MRPL21	0.798216	0.949589	0.96201	0.91599	0.875833	3.17E-07	2.52E-08	5.88E-09	7.90E-08	1.62E-08	3687	2705	2906	3119	2907
ARL4A	0.87071	0.710811	0.823731	0.752727	0.879544	3.19E-07	2.26E-06	1.89E-07	2.13E-06	6.67E-09	3689	3691	3649	3817	2728
RBM3	0.872552	0.817477	0.834878	1.057396	0.88138	3.24E-07	4.06E-06	1.22E-07	3.32E-08	5.91E-09	3690	3822	3563	2974	2711
TP53BP1	-0.69269	-0.37413	-1.00025	-0.64836	-0.49594	3.24E-07	0.004408	1.99E-10	0.000118	0.000592	3691	6050	2320	4906	5458
FLYWCH2	-0.6199	-0.55672	-0.71495	-0.34945	-0.64547	3.29E-07	1.04E-06	5.35E-09	0.000449	6.61E-06	3692	3489	2887	5338	4171
CIRBP	-0.74602	-0.75558	-0.78426	-0.64739	-0.55399	3.30E-07	2.25E-07	4.60E-08	0.000106	0.001239	3693	3172	3358	4875	5716
ITPA	0.813146	0.582577	0.717861	0.580641	0.928814	3.31E-07	0.000574	5.49E-06	0.000346	1.31E-09	3694	5310	4429	5262	2469
SET	0.934095	0.70822	0.911461	0.74518	0.844777	3.33E-07	0.000138	2.19E-06	5.00E-05	1.53E-07	3695	4790	4199	4675	3355
NPC1	-0.79673	-0.75041	-0.88091	-0.90302	-1.29108	3.34E-07	2.93E-07	2.94E-06	4.47E-10	6.71E-10	3696	3211	4274	2266	2357
UBE2K	0.799682	0.599504	0.880076	0.479564	0.700605	3.37E-07	2.28E-05	5.84E-09	0.000789	1.11E-08	3697	4250	2905	5541	2839
AGFG1	0.694982	0.781702	1.1037	0.762041	0.772547	3.39E-07	2.31E-08	1.29E-11	3.32E-10	2.92E-06	3698	2689	1904	2219	3968
ZNF771	-0.84139	-0.45004	-0.57401	-0.61804	-0.57216	3.41E-07	0.003414	9.85E-06	0.000647	3.29E-06	3700	5948	4585	5467	3999
RTRAF	0.704882	0.725363	0.870281	0.843421	0.613586	3.42E-07	1.24E-05	2.10E-07	1.90E-07	2.60E-05	3701	4091	3667	3290	4525
GLCCI1	-1.08429	-1.06652	-1.89201	-1.43973	-1.53717	3.43E-07	1.17E-07	7.66E-12	3.21E-07	4.95E-08	3702	3031	1836	3392	3121
ITPRID2	0.745744	0.78715	0.996361	0.849105	0.695492	3.45E-07	1.18E-05	4.17E-08	1.92E-06	2.03E-07	3703	4077	3335	3798	3417
RGS2	-1.91574	-1.84777	-1.85555	-2.46266	-2.3626	3.52E-07	5.88E-07	3.36E-07	1.47E-13	1.22E-09	3705	3349	3780	1276	2457
MAGOH	0.683745	0.841282	0.737758	0.746235	0.632925	3.54E-07	3.93E-07	1.48E-06	1.09E-07	2.50E-05	3707	3270	4123	3185	4517
SPPL2B	0.805735	1.041777	1.004246	1.094529	0.63452	3.55E-07	5.82E-10	4.93E-10	1.78E-10	3.10E-05	3708	2095	2463	2125	4575
MORC3	-0.63357	-0.70011	-0.60549	-0.66629	-0.72416	3.55E-07	1.98E-08	4.57E-08	4.27E-06	1.21E-08	3709	2656	3356	3982	2856
MUC20	1.494394	2.097273	1.765797	1.843387	2.331607	3.56E-07	1.12E-11	1.11E-07	6.04E-09	2.70E-14	3710	1547	3539	2685	1083
TMEM63B	0.62208	0.59323	0.831457	0.493961	0.630244	3.56E-07	1.51E-05	6.51E-12	6.88E-06	5.97E-08	3711	4135	1812	4103	3162
CEBPZ	0.741748	0.89158	0.924826	0.97834	0.932424	3.64E-07	2.51E-09	4.36E-09	1.96E-11	1.08E-12	3713	2312	2852	1863	1495
RACK1	1.410451	1.348953	1.309343	1.4854	1.599634	3.65E-07	1.22E-08	2.09E-06	6.07E-10	6.85E-09	3714	2591	4192	2313	2734
LMO7	0.914448	0.884097	1.450736	1.218108	1.235028	3.68E-07	2.89E-07	2.82E-08	3.29E-07	2.49E-07	3716	3209	3243	3403	3460
GIPR	-0.82078	-0.64669	-0.47457	-0.98616	-0.92922	3.73E-07	4.48E-05	0.008821	1.17E-12	1.18E-07	3718	4447	6807	1521	3312
ULK1	-0.65023	-0.36708	-0.43042	-0.37555	-0.81462	3.75E-07	0.001803	0.00069	0.005964	4.82E-12	3719	5715	5839	6306	1668
NPEPPS	0.582473	0.569851	0.680255	0.6854	0.699985	3.76E-07	1.68E-06	1.75E-07	8.74E-08	5.59E-07	3721	3613	3635	3144	3621
CAPNS1	0.863114	0.541298	1.098551	0.781507	0.999515	3.77E-07	0.000263	4.70E-09	8.01E-06	5.11E-08	3722	5011	2864	4148	3130
PRAMEF1	-0.80306	-0.70997	-0.85855	-0.88716	-0.63487	3.77E-07	1.96E-05	7.50E-09	2.62E-07	2.75E-05	3723	4207	2951	3350	4538
CLN6	0.6488	0.522911	0.532419	0.514911	0.522567	3.81E-07	2.58E-06	3.39E-07	1.38E-07	5.47E-05	3724	3718	3781	3226	4741
RBM38	0.980623	0.950798	0.582012	0.974385	0.63656	3.82E-07	3.61E-07	0.003087	1.18E-05	0.001404	3725	3252	6362	4260	5760
ANKS6	0.953405	0.936053	0.660681	1.03623	0.63368	3.84E-07	1.14E-06	1.10E-06	1.89E-06	1.02E-06	3726	3514	4058	3793	3743
BST2	-1.92588	-1.86451	-1.75223	-2.13727	-1.97578	3.93E-07	3.08E-06	2.27E-06	5.57E-08	6.84E-08	3727	3768	4209	3050	3189
ARFIP2	0.853594	0.845691	0.9653	0.851383	0.606698	3.97E-07	1.89E-06	5.25E-08	8.25E-06	0.000149	3728	3644	3387	4156	5044
AGO1	-0.61338	-0.39397	-0.79342	-0.64796	-0.40135	4.03E-07	0.009768	2.29E-06	2.42E-06	0.008918	3729	6420	4212	3845	6497
RPL21	1.71959	1.817678	1.298527	1.922908	2.145224	4.04E-07	5.51E-09	2.22E-05	3.80E-11	8.44E-12	3730	2458	4804	1944	1739
GSTA1	-0.67627	-0.49285	-0.40853	-0.57795	-0.46426	4.07E-07	0.003414	0.002567	0.001077	0.001663	3731	5949	6301	5652	5839
ARSI	-0.84153	-0.50604	-0.85336	-0.69095	-1.13539	4.11E-07	0.001845	3.06E-05	6.24E-05	1.46E-08	3732	5728	4901	4741	2890
CYB5R4	-0.41888	-0.31105	-0.40607	-0.34976	-0.44765	4.11E-07	0.000131	3.73E-07	1.16E-05	1.14E-07	3734	4772	3803	4255	3305
OPRD1	-0.6677	-0.32051	-0.64763	-0.49769	-0.43447	4.13E-07	0.006664	2.84E-06	0.000272	0.003126	3735	6230	4264	5185	6048
ADSS2	0.557746	0.462806	0.46142	0.481596	0.650154	4.19E-07	0.000345	1.52E-05	5.37E-05	6.19E-05	3736	5115	4707	4690	4781
SPDYE5	-0.82167	-0.53279	-1.04334	-0.77523	-0.71489	4.20E-07	0.000716	6.64E-09	1.05E-06	0.000505	3737	5385	2930	3652	5411
CRADD	-0.46878	-0.64262	-0.61036	-0.69458	-0.8041	4.21E-07	1.15E-06	6.12E-07	4.82E-07	1.11E-07	3738	3517	3919	3488	3301
ARFGAP1	0.925058	1.063438	0.87446	1.164625	0.50282	4.22E-07	1.10E-08	1.39E-07	8.20E-09	0.001415	3739	2567	3587	2722	5763
EZH2	0.965577	0.907698	1.04181	0.870103	1.410339	4.24E-07	2.34E-07	1.49E-09	8.14E-08	3.51E-08	3740	3177	2642	3129	3048
DTYMK	0.999968	1.166898	1.360685	1.291983	1.196308	4.27E-07	3.98E-08	8.77E-10	1.38E-09	7.56E-12	3742	2801	2558	2456	1721
TTI1	0.91538	1.019347	0.685025	1.12065	0.658632	4.30E-07	3.96E-08	1.65E-05	1.44E-08	7.76E-06	3743	2797	4730	2822	4215
GABARAP	-0.78438	-1.25258	-0.69065	-0.91286	-0.60666	4.35E-07	2.29E-11	0.000142	1.94E-06	0.000537	3744	1633	5315	3800	5424
GOPC	-0.58361	-0.60758	-0.56322	-0.61154	-0.51148	4.35E-07	6.72E-08	2.79E-07	5.45E-07	1.68E-07	3745	2899	3731	3515	3380
PRDM2	-0.75089	-0.55019	-0.80975	-0.6263	-0.93538	4.39E-07	0.000362	2.50E-08	2.96E-05	6.15E-08	3747	5133	3212	4514	3164
MAN2C1	-0.70249	-0.58491	-0.71755	-0.71208	-0.46589	4.39E-07	2.27E-05	1.06E-07	3.36E-07	0.000225	3748	4249	3525	3407	5153
PRDX3	0.638021	0.537276	0.945201	0.587489	0.655841	4.40E-07	3.56E-05	1.80E-11	4.31E-05	2.50E-06	3749	4372	1952	4629	3939
FAM120C	-0.45754	-0.33516	-0.7196	-0.41102	-0.54609	4.42E-07	0.000322	7.65E-10	0.000273	1.11E-07	3750	5091	2538	5189	3300
HOXB5	1.499799	2.164248	2.043918	2.576547	2.361238	4.44E-07	5.22E-08	7.84E-08	9.07E-09	1.62E-11	3751	2851	3458	2741	1829
HJURP	0.644124	0.959924	1.012118	0.922664	0.546973	4.44E-07	3.14E-09	1.02E-10	5.94E-08	9.56E-06	3752	2354	2221	3064	4266
DDX1	0.658945	0.72313	0.707333	0.954511	0.687969	4.45E-07	1.52E-07	1.22E-06	1.18E-11	5.10E-08	3753	3088	4085	1790	3128
EZH1	-0.54385	-0.44574	-0.59453	-0.56118	-0.62809	4.46E-07	1.06E-05	1.17E-07	8.26E-07	1.44E-07	3754	4042	3558	3600	3348
PRSS22	1.189642	1.202462	1.447948	1.382883	1.66732	4.53E-07	5.36E-08	9.39E-09	2.60E-10	1.27E-10	3755	2858	2998	2183	2083
AP2B1	0.814098	0.46315	0.768833	0.764761	0.448294	4.55E-07	0.005122	7.77E-05	2.26E-06	0.002294	3756	6109	5147	3831	5958
PLEKHG6	0.964285	0.956681	0.869006	0.671203	0.928167	4.57E-07	4.23E-08	4.88E-07	3.84E-05	5.61E-07	3757	2814	3861	4599	3623
BACE2	1.282858	1.114242	1.849609	1.56077	1.964484	4.59E-07	6.04E-06	2.34E-10	8.81E-09	4.89E-10	3758	3907	2349	2739	2307
UBE2A	0.649618	0.402975	0.905281	0.677652	0.413221	4.66E-07	0.003658	4.67E-07	6.84E-05	0.007438	3759	5973	3852	4764	6406
ATPAF2	-0.62591	-0.45892	-0.44543	-0.54865	-0.29077	4.67E-07	0.000342	1.41E-05	3.71E-05	0.001027	3760	5111	4689	4586	5639
NEBL	0.962853	1.240573	1.6102	1.382599	1.917041	4.69E-07	3.51E-08	1.41E-10	7.29E-10	2.27E-16	3761	2774	2269	2348	703
RHOT2	0.650011	0.523646	0.651261	0.47552	0.759037	4.74E-07	0.000417	5.63E-06	0.000816	8.11E-08	3762	5188	4430	5550	3234
SAMD3	-0.66968	-0.41397	-0.50091	-0.66815	-0.72303	4.75E-07	0.002165	0.000148	2.62E-07	2.74E-07	3763	5797	5329	3348	3482
SPR	0.812254	1.038952	1.018015	0.968518	1.082526	4.75E-07	2.14E-09	5.34E-13	5.92E-09	2.24E-12	3764	2287	1519	2680	1574
MYH9	-1.22945	-1.49337	-0.76252	-1.18019	-1.07181	4.79E-07	1.25E-12	0.000232	3.20E-08	8.31E-06	3766	1273	5496	2963	4235

ARL15	-1.01233	-1.06541	-1.0559	-1.01364	-1.19049	4.80E-07	6.77E-08	5.79E-11	7.67E-07	1.80E-13	3767	2900	2128	3583	1287
CCNI2	0.777879	0.749749	0.734345	0.677336	0.885036	4.81E-07	4.09E-08	7.66E-08	2.36E-08	1.70E-09	3769	2808	3453	2915	2511
ROGDI	-0.66415	-0.63255	-0.84641	-0.63937	-0.51695	4.84E-07	6.14E-06	2.50E-11	1.94E-05	8.79E-05	3770	3910	2010	4395	4885
GNS	-0.77266	-1.13986	-0.99775	-0.87961	-1.09145	4.85E-07	1.10E-07	4.02E-07	2.70E-06	2.89E-09	3771	3016	3821	3869	2598
NEK3	1.041098	0.909113	0.862067	0.859065	0.791264	4.86E-07	1.47E-06	7.58E-06	5.59E-05	0.000544	3772	3580	4513	4711	5426
DPAGT1	0.933752	1.017015	0.927751	1.264894	0.630272	4.89E-07	8.87E-07	1.71E-06	2.12E-09	0.001201	3773	3450	4155	2523	5700
OSBPL1A	-0.96732	-0.81951	-1.02236	-0.77331	-0.83783	4.90E-07	4.76E-05	1.29E-06	5.82E-05	3.73E-05	3774	4469	4103	4720	4631
PHF11	-0.7182	-0.75537	-0.72003	-0.88439	-0.46145	4.96E-07	1.88E-05	1.07E-05	1.56E-06	0.001109	3775	4197	4617	3741	5672
RAVER1	0.658674	0.784304	0.907514	0.788485	0.802134	5.05E-07	7.85E-10	1.31E-13	1.06E-09	8.27E-11	3777	2138	1356	2403	2022
CHRFAM7	-0.63573	-0.34011	-0.84252	-0.59315	-0.7956	5.06E-07	0.001358	7.03E-09	2.93E-05	2.05E-09	3778	5623	2941	4509	2541
NOL4L	0.873532	0.768091	0.971371	1.106928	0.908365	5.08E-07	1.46E-05	1.69E-07	1.20E-07	4.21E-07	3779	4125	3626	3200	3572
MRPS17	0.896689	1.139243	0.937673	1.107287	0.914839	5.09E-07	2.42E-08	2.86E-07	2.48E-08	5.39E-09	3780	2697	3735	2920	2698
MRPL41	0.662016	0.659512	0.591037	0.603682	0.46011	5.09E-07	3.64E-06	6.43E-08	3.50E-05	0.00022	3781	3796	3427	4565	5142
ABR	0.857433	0.733221	0.98317	0.660792	1.40042	5.10E-07	3.42E-07	6.84E-07	3.81E-05	6.14E-11	3782	3240	3940	4595	1974
H2AC4	1.177354	0.778837	1.699172	1.129061	1.70658	5.10E-07	0.000853	7.15E-10	6.46E-05	8.73E-14	3783	5448	2525	4751	1209
CTBS	-0.81807	-0.78067	-0.83223	-0.84549	-0.67119	5.17E-07	5.35E-06	1.48E-07	1.11E-06	3.45E-06	3784	3881	3601	3663	4016
ARFRP1	0.868655	0.712803	0.687939	0.78457	0.610698	5.25E-07	5.62E-06	2.98E-07	4.16E-06	5.34E-06	3785	3891	3752	3974	4126
GET4	0.918108	1.07607	0.89368	0.979622	1.117462	5.26E-07	8.08E-09	1.74E-07	5.34E-08	1.32E-09	3786	2519	3631	3044	2472
CHAF1A	0.693315	0.905969	0.828052	0.847175	1.008915	5.26E-07	4.49E-09	2.03E-07	5.01E-08	2.28E-09	3787	2414	3662	3039	2569
PARK7	0.757427	0.471786	0.751858	0.644525	0.843767	5.33E-07	0.001381	1.79E-05	4.20E-05	1.59E-09	3789	5628	4756	4621	2501
KCNH2	-0.6524	-0.47457	-0.84073	-0.67691	-0.68478	5.34E-07	0.000281	3.41E-06	8.75E-05	1.80E-05	3790	5033	4304	4820	4441
JPH1	0.831388	0.780909	0.991459	0.959276	0.653399	5.40E-07	4.13E-06	3.32E-08	1.27E-07	2.90E-06	3794	3825	3290	3206	3966
DGCR2	0.693307	0.747127	0.665912	0.7531	0.567806	5.43E-07	1.53E-07	4.64E-07	1.82E-07	1.23E-06	3796	3089	3849	3281	3779
ARMC10	0.789895	0.819643	0.825338	0.870894	0.767248	5.52E-07	7.36E-08	5.78E-07	2.43E-09	1.51E-06	3798	2926	3894	2548	3822
MYORG	0.98809	0.983217	1.216842	1.013602	0.743439	5.56E-07	1.03E-05	4.93E-08	1.39E-06	0.000708	3801	4035	3369	3710	5526
NOMO3	0.834489	0.785825	0.713332	1.019937	1.030568	5.58E-07	1.03E-06	1.05E-06	9.15E-09	9.40E-09	3802	3488	4050	2745	2801
MRPL51	0.797611	0.529064	0.983628	0.59853	0.63526	5.61E-07	0.000193	6.36E-09	7.07E-05	1.32E-05	3803	4904	2924	4770	4367
ERCC1	-0.70259	-0.4643	-0.32185	-0.59284	-0.72282	5.69E-07	0.000828	0.003856	4.70E-05	2.89E-07	3807	5433	6459	4657	3494
DDX17	-0.69968	-0.71547	-0.72414	-0.67415	-0.62286	5.74E-07	3.14E-07	1.62E-06	2.87E-05	0.00013	3809	3228	4144	4501	5006
TRMT2B	0.595768	0.706091	0.525935	0.773801	0.387126	5.79E-07	1.17E-08	2.15E-06	3.27E-10	0.000686	3811	2580	4195	2217	5514
CENPW	1.297111	1.243127	1.383258	1.245184	1.148261	5.83E-07	1.43E-06	1.54E-07	2.84E-06	1.47E-07	3812	3570	3606	3886	3351
CNPY2	0.818459	0.639561	0.495735	0.773607	0.542377	5.89E-07	0.000129	0.000769	4.59E-07	8.45E-05	3813	4764	5876	3480	4872
NPHP4	-0.71123	-0.9399	-0.38285	-0.66216	-0.35612	5.91E-07	9.03E-11	0.001963	2.97E-05	0.005589	3814	1815	6189	4515	6288
KDELR2	1.061678	0.741357	1.243363	0.946696	0.9189	5.92E-07	0.000125	2.82E-09	9.00E-08	6.11E-07	3815	4755	2773	3148	3642
BAG1	0.823616	0.84964	0.966908	0.948746	0.469415	5.93E-07	6.60E-07	4.77E-10	1.29E-07	0.001732	3816	3374	2459	3211	5852
ARL14	0.806671	0.967918	1.116344	1.041332	0.574276	5.93E-07	3.26E-06	2.15E-09	2.46E-07	0.000242	3817	3777	2714	3339	5177
DNAJC2	0.850908	1.014469	1.121714	0.907721	0.720352	5.97E-07	1.48E-07	8.28E-11	2.15E-06	1.18E-05	3820	3083	2179	3818	4325
ERMP1	0.832954	1.174266	0.789871	0.977171	0.875134	5.98E-07	2.34E-10	4.43E-07	5.27E-07	3.63E-08	3821	1949	3841	3507	3056
HIBADH	0.750062	0.775687	0.715158	0.824597	0.448997	6.07E-07	1.49E-08	6.57E-09	2.71E-10	0.000289	3822	2626	2928	2188	5228
ARF6	0.74649	0.766757	1.068802	0.575481	1.091787	6.10E-07	1.42E-07	1.84E-11	4.27E-05	3.33E-13	3823	3073	1958	4627	1347
ABCC4	-1.3782	-0.81922	-1.52161	-1.19762	-1.26832	6.21E-07	0.00014	3.19E-09	6.53E-06	9.33E-07	3825	4794	2801	4086	3720
TMEM33	0.983884	1.149986	1.211409	1.006651	0.731874	6.21E-07	1.65E-07	8.62E-11	1.04E-05	0.002635	3826	3101	2188	4223	5994
KIAA1958	0.706679	0.959029	0.709612	0.840868	0.547104	6.21E-07	3.79E-09	1.01E-05	9.70E-07	1.46E-05	3827	2390	4595	3634	4393
SERP1	0.649133	0.796015	0.705814	0.896971	0.745178	6.24E-07	6.88E-09	2.96E-07	1.04E-11	1.37E-08	3829	2491	3747	1766	2880
SLC22A3	1.40497	1.472045	2.045832	1.499863	1.482806	6.30E-07	3.92E-06	1.14E-12	3.88E-07	1.05E-09	3831	3815	1612	3440	2435
SREBF1	0.765931	0.940501	1.151865	0.691383	0.991551	6.31E-07	2.88E-08	6.90E-13	3.24E-05	4.05E-10	3832	2731	1551	4542	2271
MED14	0.721326	1.00728	0.512654	1.147784	0.621887	6.37E-07	1.72E-07	0.004216	6.42E-08	4.94E-06	3833	3119	6500	3080	4100
RTF1	-0.60379	-0.63311	-0.7869	-0.85851	-0.82851	6.38E-07	2.09E-06	2.22E-05	2.70E-06	4.92E-06	3834	3672	4803	3868	4098
HEATR5B	-0.60104	-0.52862	-0.71429	-0.63708	-0.44796	6.38E-07	0.000214	3.16E-08	6.77E-06	0.000308	3835	4930	3268	4099	5238
ALDH4A1	0.752587	0.702874	1.012968	0.482947	0.913401	6.41E-07	0.00017	4.74E-07	0.009623	2.83E-05	3836	4858	3857	6516	4545
CRYBB3	-0.58485	-0.46931	-0.46506	-0.46546	-0.41233	6.41E-07	1.78E-05	9.47E-06	3.78E-06	2.09E-05	3837	4182	4571	3945	4476
DNAJC5	0.695867	0.859515	0.806452	0.983048	0.785105	6.44E-07	1.98E-08	1.59E-09	3.69E-10	7.18E-06	3838	2657	2650	2236	4192
GSE1	1.05942	1.060418	0.942564	1.352504	1.276531	6.45E-07	1.01E-07	1.40E-06	5.35E-10	1.54E-11	3839	2999	4116	2288	1820
PDSS1	0.692027	0.859626	0.835735	0.906318	0.803429	6.45E-07	9.63E-09	1.52E-07	3.13E-08	8.34E-06	3840	2548	3603	2957	4237
PYCR1	1.067687	1.073708	1.050005	1.051387	1.107037	6.50E-07	7.81E-08	2.42E-06	1.18E-09	2.91E-07	3841	2939	4228	2419	3495
SH3GLB2	0.710512	0.90187	0.819239	0.768236	0.809334	6.51E-07	5.88E-10	1.50E-13	2.20E-08	1.01E-10	3842	2096	1378	2907	2055
FAM136A	0.889405	0.81783	0.887021	0.900933	0.867868	6.51E-07	5.26E-06	1.58E-07	1.64E-07	5.72E-08	3843	3874	3609	3262	3154
HK1	-0.73091	-0.76186	-0.56888	-0.58739	-0.51674	6.59E-07	9.21E-08	0.000108	2.38E-05	0.001467	3844	2978	5240	4432	5780
TMX4	-0.61743	-0.53025	-0.62186	-0.83245	-0.75117	6.61E-07	1.33E-05	2.35E-05	3.01E-07	3.54E-07	3845	4105	4821	3373	3540
ATP6V1A	-0.40604	-0.4039	-0.47145	-0.30828	-0.5643	6.69E-07	0.000383	3.26E-06	0.001635	6.15E-06	3847	5155	4296	5810	4157
ZNF506	-0.66313	-0.41368	-0.58512	-0.60967	-0.65216	6.70E-07	0.000173	1.22E-05	1.15E-06	7.38E-06	3848	4867	4649	3680	4203
NDUFA3	0.74028	1.142942	0.67724	0.972969	0.987822	6.73E-07	5.89E-12	1.66E-07	1.41E-09	8.24E-11	3849	1462	3622	2462	2021
GPATCH3	-0.69769	-0.57667	-0.77683	-0.72712	-0.66672	6.83E-07	0.000479	1.50E-06	7.69E-07	4.70E-05	3850	5234	4127	3584	4704
PPP1R13L	0.887065	1.070892	1.162224	0.944113	0.676397	6.84E-07	1.98E-06	2.98E-10	8.16E-06	5.13E-05	3851	3653	2383	4154	4732
SOS1	-0.50525	-0.70312	-0.58873	-0.63052	-0.45111	6.87E-07	3.97E-07	4.45E-07	1.41E-05	3.30E-05	3852	3272	3842	4308	4592
RPL15	1.300588	1.344822	1.177279	1.484644	1.520574	6.88E-07	2.22E-08	4.43E-06	2.85E-10	8.14E-10	3853	2686	4375	2199	2395
CFHR1	-0.7369	-0.62971	-0.96796	-0.57087	-0.8128	6.91E-07	0.002752	6.77E-06	4.44E-05	7.06E-05	3855	5863	4481	4640	4827
ACACA	0.675363	0.665436	0.827229	0.634448	0.799065	6.93E-07	7.47E-08	1.97E-09	6.41E-08	1.11E-08	3856	2931	2699	3079	2841
RPS23	1.450219	1.267004	1.140203	1.583247	1.478126	6.93E-07	8.60E-07	0.000124	1.78E-11	3.71E-07	3857	3436	5277	1847	3554
FGFR4	1.202219	1.36362	0.742613	1.144953	1.365428	6.97E-07	4.82E-09	0.000458	7.54E-09	2.33E-09	3858	2434	5709	2712	2574
PGLS	0.730328	0.836703	1.01634	0.749335	1.006553	6.98E-07	1.35E-09	1.79E-11	7.67E-08	7.56E-13	3859	2219	1947	3110	1450
GALR3	-0.55844	-0.28946	-0.66662	-0.42836	-0.38483	6.99E-07	0.005079	1.37E-07	0.000594	0.003204	3860	6103	3583	5429	6054
PPP1R15B	0.616041	0.66422	0.474261	0.515334	0.428612	7.00E-07	2.09E-06	0.000175	0.000399	0.000414	3861	3670	5391	5307	5345

TAS2R60	-0.6162	-0.52207	-0.52309	-0.61751	-0.98945	7.03E-07	0.000373	7.90E-06	0.000273	5.74E-07	3862	5147	4525	5187	3629
FRG1	-0.59787	-0.40089	-0.85312	-0.54427	-0.60122	7.05E-07	0.001107	2.74E-09	2.77E-05	1.20E-05	3863	5549	2769	4486	4331
NKRF	0.496715	0.631601	0.693284	0.485411	0.448256	7.06E-07	7.57E-07	6.87E-09	8.09E-06	0.000549	3864	3409	2935	4150	5431
UBQLN4	0.658416	0.891866	0.674732	1.029916	0.85153	7.14E-07	1.37E-07	2.17E-06	1.55E-11	1.25E-07	3865	3066	4197	1832	3321
PIP5K1C	-0.96754	-1.10766	-1.18929	-0.8319	-1.3425	7.31E-07	5.34E-09	8.49E-11	9.15E-07	4.41E-13	3867	2450	2185	3614	1381
KSR1	-0.8607	-0.65726	-0.83007	-0.59236	-0.85522	7.35E-07	0.00029	1.23E-05	0.000364	7.04E-08	3868	5045	4654	5280	3200
GSAP	-0.66927	-0.65505	-0.85381	-0.71032	-0.81439	7.35E-07	1.97E-08	3.24E-10	1.94E-07	1.87E-10	3869	2655	2400	3295	2142
CLN3	0.698224	0.768375	0.700616	0.918087	0.835005	7.37E-07	1.16E-07	1.39E-07	5.90E-10	5.18E-07	3870	3029	3590	2306	3608
KCNK5	0.934581	0.862045	0.744309	0.816062	0.561584	7.45E-07	5.33E-06	0.000182	6.12E-05	0.001474	3871	3880	5408	4736	5783
IFT57	0.96258	1.367008	0.807889	1.397146	0.983509	7.47E-07	3.36E-10	4.10E-06	9.28E-12	2.14E-09	3872	2013	4355	1747	2551
CREBRF	-0.98582	-0.90586	-0.88894	-1.11162	-1.07846	7.56E-07	0.000112	4.53E-07	1.70E-06	5.27E-09	3873	4733	3846	3756	2690
CRCP	0.687645	0.720233	0.64368	0.735499	0.741035	7.64E-07	1.47E-08	1.41E-07	2.78E-09	6.97E-09	3875	2623	3592	2569	2738
FAM241A	-0.97453	-0.69618	-0.749	-0.84899	-0.71248	7.64E-07	0.00025	3.04E-09	4.27E-06	3.32E-05	3876	4990	2789	3983	4594
INCENP	0.680619	0.733375	0.644212	0.838541	0.502886	7.66E-07	3.84E-08	1.02E-06	1.42E-12	1.27E-05	3878	2791	4041	1546	4358
QPRT	1.479114	1.632914	1.526049	1.766107	1.716601	7.68E-07	2.03E-07	1.71E-06	1.54E-07	3.40E-09	3879	3154	4154	3247	2622
EPHB3	1.338056	2.155958	1.217702	1.817217	2.093664	7.72E-07	1.02E-14	7.75E-07	1.13E-09	1.51E-08	3880	837	3967	2416	2892
MCOLN1	-0.82054	-0.55052	-0.74801	-0.5692	-0.89492	7.73E-07	0.000594	2.40E-06	0.000201	2.50E-08	3881	5326	4227	5062	2980
CDK12	0.933878	0.897437	0.851503	0.716888	0.746288	7.80E-07	2.69E-05	2.08E-05	4.81E-05	1.03E-05	3883	4301	4785	4665	4280
CD79B	-0.91687	-0.53927	-1.11687	-0.81925	-0.96383	7.83E-07	0.007308	2.98E-07	0.000434	4.72E-07	3884	6277	3751	5331	3596
DDB1	0.671986	0.584994	0.804999	0.655334	0.435594	7.84E-07	4.57E-07	1.45E-09	3.59E-07	0.000355	3885	3296	2638	3421	5280
POLA1	0.801482	1.183698	0.688476	1.27999	0.801795	7.89E-07	1.07E-09	3.91E-06	8.88E-12	7.19E-08	3886	2184	4333	1741	3211
FAM153B	-0.7182	-0.5349	-1.03318	-0.71023	-1.07189	7.96E-07	7.83E-05	2.70E-12	4.57E-06	1.41E-13	3887	4620	1709	3998	1261
PPP4R3B	0.568632	0.728739	0.679481	0.620791	0.71576	8.00E-07	2.97E-07	1.25E-07	1.05E-06	5.49E-10	3889	3216	3565	3654	2320
DACT1	-1.387	-1.4168	-0.70677	-1.2871	-0.80703	8.00E-07	4.50E-08	0.000377	1.73E-05	0.000832	3890	2824	5649	4358	5574
TOPBP1	0.583725	0.522852	0.293519	0.482303	0.548108	8.02E-07	7.56E-07	0.00346	5.70E-07	5.82E-07	3891	3408	6420	3523	3632
LAGE3	0.741669	0.938967	1.026429	1.135513	0.606015	8.08E-07	2.75E-09	1.10E-08	4.87E-12	2.93E-05	3892	2332	3032	1673	4553
MCPH1	-0.72922	-0.50601	-0.72324	-0.72352	-0.71313	8.11E-07	0.000973	5.12E-07	4.75E-06	1.12E-05	3893	5497	3870	4006	4308
LZTS3	1.090485	1.380191	0.81149	1.249565	0.845777	8.16E-07	1.76E-09	3.10E-05	9.10E-08	1.26E-05	3895	2253	4904	3151	4351
DGKQ	0.75765	0.759112	0.950843	0.761688	0.99557	8.19E-07	2.32E-05	8.93E-08	1.96E-05	1.04E-09	3896	4257	3485	4397	2429
GNA15	-0.62348	-0.69861	-0.78853	-0.68923	-0.47856	8.19E-07	8.74E-08	1.88E-07	3.34E-06	0.005476	3897	2967	3647	3918	6272
NFATC2	-0.91273	-0.6863	-1.32741	-1.00023	-1.04461	8.23E-07	0.000354	1.20E-09	8.74E-06	6.13E-06	3898	5125	2617	4164	4156
ITM2B	-1.12206	-1.59514	-0.75692	-1.27661	-0.65266	8.26E-07	5.65E-11	0.000467	1.70E-06	0.002942	3899	1761	5715	3757	6034
MECOM	0.979793	0.986689	1.134749	1.256772	1.342391	8.29E-07	9.83E-07	1.49E-08	9.41E-07	5.00E-08	3900	3475	3102	3623	3123
GNL3L	0.96605	1.107568	0.679241	1.017267	1.134425	8.29E-07	5.61E-09	2.08E-05	9.59E-09	1.18E-09	3901	2459	4788	2755	2452
GRIN2D	1.055901	0.878854	1.274124	0.871857	1.014466	8.34E-07	0.0001	6.05E-09	7.00E-05	5.84E-09	3903	4698	2915	4768	2708
GSKIP	1.109135	0.844152	1.794774	0.854026	1.173286	8.41E-07	2.52E-06	9.77E-13	4.88E-05	4.29E-06	3904	3714	1591	4668	4066
SHH	0.944388	0.622167	1.076126	0.97701	1.22727	8.51E-07	0.000925	5.85E-07	1.30E-05	2.93E-09	3908	5482	3902	4277	2601
FAM193B	-0.61669	-0.52562	-0.6294	-0.70383	-0.55817	8.55E-07	1.76E-05	2.43E-08	1.44E-09	7.50E-06	3909	4177	3200	2465	4210
HMGCR	1.068438	1.343495	1.305664	1.265458	1.11295	8.57E-07	2.17E-08	2.18E-09	3.19E-08	2.18E-07	3910	2678	2719	2961	3435
NUDT5	0.791069	0.551889	0.815734	0.435119	0.796879	8.58E-07	0.000516	3.11E-07	0.004331	7.21E-06	3911	5267	3770	6193	4193
GADD45G	0.716722	0.762491	0.951311	0.862995	0.739716	8.61E-07	1.81E-06	5.15E-08	5.10E-09	1.12E-05	3912	3631	3380	2660	4312
PI3	1.79453	1.736241	2.453391	2.022118	1.943942	8.69E-07	1.06E-05	1.93E-09	1.29E-08	1.38E-08	3913	4043	2692	2808	2882
ST7L	-0.61213	-0.52393	-0.62712	-0.52925	-0.55153	8.77E-07	0.000418	4.00E-06	0.000264	8.64E-06	3914	5190	4344	5169	4245
MTMR3	-0.73609	-0.57032	-0.60758	-0.6879	-0.90704	8.83E-07	0.000388	9.01E-06	4.76E-05	2.14E-10	3916	5161	4561	4662	2175
EXOG	-0.64851	-0.40055	-0.69422	-0.56342	-0.82309	8.85E-07	0.005237	2.89E-07	0.000489	1.71E-10	3917	6122	3739	5366	2129
NUP58	0.934715	0.830973	0.799534	0.952732	1.106286	8.90E-07	7.16E-07	1.55E-05	6.17E-09	5.77E-08	3918	3396	4712	2687	3155
CDKN2D	-0.62642	-0.52894	-0.55646	-0.56452	-0.46137	8.90E-07	2.39E-05	2.01E-06	8.24E-05	5.33E-05	3919	4264	4183	4807	4737
SLC7A5	1.582595	2.041886	1.395782	1.834244	1.372758	8.92E-07	4.61E-12	2.96E-07	1.83E-10	8.01E-06	3921	1425	3748	2134	4223
PRKAR1A	-0.80767	-1.20033	-0.66058	-1.00001	-0.90387	8.96E-07	5.52E-12	0.000247	8.87E-08	4.15E-08	3923	1451	5512	3147	3083
PLBD2	-1.11223	-0.72868	-0.89425	-0.79351	-1.27903	9.03E-07	0.001104	3.91E-05	0.000389	2.80E-08	3924	5548	4964	5298	3010
KCNMA1	-0.84229	-0.75004	-0.81161	-0.64488	-0.54067	9.04E-07	3.03E-07	3.27E-08	0.000894	0.00584	3925	3223	3282	5581	6303
PPP2R5A	0.586245	0.721042	0.832228	0.723889	0.905508	9.04E-07	2.66E-06	1.40E-06	3.56E-06	1.00E-07	3926	3724	4117	3931	3274
IFITM1	1.665065	1.805663	1.919962	1.984706	1.939807	9.06E-07	1.29E-09	8.79E-09	7.03E-09	2.00E-12	3927	2214	2988	2706	1557
PLA2G2A	2.216788	2.214523	2.696952	1.947099	1.176025	9.09E-07	4.55E-07	6.22E-09	9.18E-06	0.008104	3928	3295	2923	4185	6443
SIDT2	-0.94812	-1.01725	-0.92663	-0.99115	-1.16544	9.11E-07	9.84E-09	1.15E-07	3.55E-08	4.73E-12	3929	2550	3555	2982	1666
FYTTD1	-0.59272	-0.69354	-0.5633	-0.78061	-0.66527	9.12E-07	7.15E-06	5.49E-06	4.96E-07	1.11E-08	3930	3942	4428	3490	2840
FANCI	0.57534	0.797979	0.59258	0.675256	0.389305	9.21E-07	1.73E-07	9.64E-06	3.06E-05	0.007525	3934	3121	4577	4523	6410
DNAJA3	0.680241	0.624596	0.797902	0.830379	0.840414	9.35E-07	1.73E-07	1.62E-09	5.24E-09	4.30E-07	3935	3120	2651	2664	3579
ZNF598	0.737377	0.802214	0.489408	0.885215	0.644317	9.44E-07	3.07E-10	8.09E-05	1.68E-09	1.69E-06	3936	1994	5167	2490	3848
GNPNAT1	0.830369	1.250259	1.399054	1.342095	0.617046	9.49E-07	4.17E-08	2.54E-11	6.03E-09	0.0001	3937	2811	2015	2684	4931
PPP1R35	0.696292	0.781477	0.399735	0.723239	0.83308	9.49E-07	9.39E-11	0.000657	9.35E-07	1.19E-07	3938	1821	5816	3622	3315
OVOL2	1.025259	1.538733	1.491833	1.349266	1.000337	9.54E-07	4.59E-08	1.13E-09	5.41E-07	1.05E-06	3941	2831	2606	3514	3749
GRIN2A	-0.55469	-0.42898	-0.79087	-0.58259	-0.73995	9.54E-07	0.000103	2.29E-13	1.10E-06	1.90E-10	3942	4705	1431	3660	2148
PSMD13	0.735461	0.476134	0.762278	0.648235	0.81622	9.71E-07	0.00375	3.26E-05	0.000128	7.49E-07	3946	5990	4918	4929	3679
EFNB2	1.205217	0.863621	1.071848	0.77427	1.022815	9.73E-07	0.001012	2.44E-06	0.005591	2.31E-06	3947	5510	4231	6290	3926
GOLGA4	0.64789	0.563399	0.835437	0.611496	0.679661	9.81E-07	0.000236	9.54E-09	7.21E-05	2.66E-06	3948	4968	3002	4780	3947
NBPF12	-0.67982	-0.55536	-0.9028	-0.61178	-0.39034	9.81E-07	5.02E-05	5.60E-13	0.000178	0.006675	3949	4485	1527	5025	6355
PABPC1L	1.283591	1.399324	0.735651	1.358274	1.061271	9.82E-07	2.58E-09	0.000422	9.19E-10	9.04E-08	3950	2320	5684	2386	3259
TMEM30B	0.983163	1.010127	1.425688	1.238594	0.981881	9.98E-07	1.67E-07	1.35E-13	4.26E-10	6.54E-07	3952	3107	1363	2253	3659
KIR3DL1	-0.74621	-0.5913	-1.08642	-0.72923	-0.66906	1.00E-06	2.12E-05	7.45E-11	9.39E-06	4.99E-05	3954	4231	2161	4193	4725
CTNNA1	0.892687	0.462211	1.198982	0.888854	1.003442	1.02E-06	0.000614	7.20E-08	3.08E-07	1.86E-05	3955	5332	3446	3384	4449
COASY	0.746068	0.648628	0.724184	0.709847	0.658116	1.02E-06	3.74E-05	1.22E-06	7.81E-07	2.12E-05	3956	4393	4087	3586	4479
H4-16	1.255705	1.278755	0.914555	1.363062	1.302578	1.02E-06	3.67E-07	1.40E-05	4.42E-08	1.03E-08	3957	3258	4685	3022	2821

SMIM10L1	-0.51699	-0.58893	-0.44849	-0.43508	-0.42335	1.03E-06	5.33E-07	0.000116	0.000158	0.000139	3958	3321	5261	4991	5026
SPC24	0.665314	0.882769	0.722952	0.764393	0.602658	1.03E-06	3.69E-08	6.08E-08	3.00E-06	1.88E-06	3960	2781	3409	3902	3869
PHF14	0.57971	0.58546	0.527906	0.664335	0.678027	1.04E-06	2.51E-06	8.97E-05	5.93E-08	5.26E-08	3962	3712	5196	3062	3141
ACOX3	-0.66919	-0.40804	-0.62187	-0.57174	-1.08761	1.04E-06	0.007064	3.82E-05	0.000752	5.26E-08	3965	6259	4954	5525	3140
NIPAL2	0.909668	0.785353	1.039081	0.8817	1.035031	1.05E-06	2.80E-06	1.10E-09	3.39E-07	6.82E-09	3966	3743	2595	3408	2730
LSM14B	0.963734	0.985487	0.779452	1.103584	0.856163	1.05E-06	7.36E-08	1.23E-06	1.60E-09	5.93E-07	3967	2927	4088	2483	3636
ZNF578	-0.76506	-0.47167	-0.72491	-0.72876	-0.85984	1.05E-06	0.000705	1.33E-06	7.00E-06	7.45E-07	3968	5378	4107	4112	3675
TMEM44	-0.75347	-0.36866	-0.51606	-0.61106	-0.47232	1.05E-06	0.002333	0.000436	2.50E-06	0.000558	3969	5816	5697	3853	5436
OSBPL9	-0.72615	-0.68793	-0.57767	-0.66606	-0.54244	1.06E-06	2.60E-06	3.94E-06	5.96E-06	2.99E-05	3970	3719	4341	4061	4564
RAC1	0.996013	0.586204	1.351097	0.655689	1.11108	1.06E-06	0.001358	3.38E-09	0.000501	6.59E-09	3971	5622	2813	5377	2726
UPF1	0.514149	0.851668	0.478332	0.804623	0.457575	1.06E-06	4.17E-07	0.00292	3.25E-05	0.001039	3972	3276	6349	4545	5646
CARD10	1.178859	1.434536	1.521361	1.590331	1.303474	1.06E-06	2.56E-05	2.05E-09	2.34E-07	2.74E-08	3973	4282	2708	3324	3000
USP7	0.67184	0.709551	0.732216	0.950281	0.885266	1.07E-06	4.83E-06	2.36E-06	1.30E-07	3.72E-09	3975	3855	4220	3213	2639
TSPYL4	-0.53852	-0.42981	-0.64459	-0.5749	-0.62158	1.08E-06	6.15E-05	5.59E-07	1.03E-05	1.01E-06	3976	4542	3891	4219	3739
PNP	0.730221	0.474874	0.84953	0.730599	0.731177	1.08E-06	0.000185	1.37E-08	8.09E-07	1.89E-07	3977	4888	3086	3593	3406
KIAA1109	-0.62856	-0.80981	-0.34801	-0.78637	-0.54898	1.08E-06	3.08E-08	0.00967	2.33E-08	5.63E-05	3978	2738	6833	2913	4748
EPB41L1	1.067605	0.999155	1.09475	1.051266	1.049062	1.08E-06	5.99E-07	1.57E-06	6.18E-07	1.61E-08	3979	3353	4137	3545	2905
PARVB	-1.36878	-1.06137	-0.77069	-0.90212	-1.34264	1.09E-06	2.97E-07	0.002752	0.000237	4.92E-08	3980	3218	6331	5122	3120
PDCD5	0.830737	1.128776	0.892621	0.900696	0.848876	1.09E-06	5.31E-09	8.17E-07	8.44E-09	7.10E-08	3982	2448	3985	2729	3201
CAPZB	-1.00625	-1.34692	-0.8094	-1.15938	-0.57109	1.10E-06	9.72E-10	0.000106	4.49E-07	0.006381	3984	2169	5229	3475	6335
TUBGCP6	-0.65517	-0.3596	-0.42026	-0.33059	-0.55173	1.10E-06	0.000353	0.000379	0.002569	0.000396	3985	5124	5652	5977	5324
PNRC2	0.694489	0.811309	0.887282	0.768221	0.767231	1.10E-06	5.59E-07	6.58E-10	3.62E-08	5.60E-10	3986	3336	2512	2986	2323
TM6SF2	-0.79048	-0.78527	-0.52555	-0.85586	-0.79965	1.12E-06	3.46E-09	0.000289	9.02E-10	2.74E-06	3988	2375	5562	2377	3956
DDC	1.411459	1.564613	1.441076	1.626182	1.426495	1.13E-06	2.19E-08	1.14E-07	3.17E-09	5.31E-09	3990	2682	3553	2598	2693
CMPK1	0.73924	0.805025	1.073125	1.113442	1.015212	1.13E-06	8.17E-08	4.86E-10	7.15E-10	3.32E-09	3991	2950	2461	2343	2620
ASAP2	0.584623	0.521088	0.920715	0.786245	0.862585	1.13E-06	1.20E-06	2.02E-11	3.67E-10	1.53E-09	3992	3525	1978	2234	2492
HPRT1	0.956072	1.185073	1.384897	1.544383	0.783754	1.13E-06	4.27E-06	6.82E-08	1.89E-09	0.000127	3993	3829	3437	2507	5002
SERINC3	0.66265	0.512949	0.585963	0.838284	0.688106	1.13E-06	3.48E-05	1.91E-05	2.63E-08	0.001056	3994	4367	4769	2928	5657
NBPF19	-0.80281	-0.48377	-0.83647	-0.54099	-0.65563	1.14E-06	9.17E-05	1.29E-10	4.81E-05	2.02E-07	3996	4677	2259	4666	3415
PPP1CB	0.86958	0.546055	0.934659	0.78292	1.066483	1.14E-06	0.000511	2.57E-07	1.94E-05	5.58E-12	3997	5262	3715	4390	1684
SNX11	-0.4526	-0.47641	-0.40923	-0.53987	-0.53993	1.16E-06	0.000281	4.65E-05	4.10E-05	2.23E-08	3999	5037	5017	4615	2964
PDPR	-0.63111	-0.63534	-0.7163	-0.67198	-0.38379	1.18E-06	1.67E-05	1.26E-08	1.11E-05	0.009137	4002	4154	3072	4245	6512
HIVEP1	-0.48284	-0.32232	-0.36385	-0.29453	-0.51451	1.18E-06	0.000321	0.000343	0.002441	3.62E-07	4003	5088	5610	5964	3550
D2HGDH	0.804817	0.766277	0.639963	0.879493	1.212351	1.20E-06	1.98E-07	3.05E-05	8.87E-07	1.75E-08	4004	3145	4900	3609	2916
TRA2A	0.467936	0.426972	0.501002	0.437259	0.681012	1.20E-06	5.22E-05	3.60E-07	0.000256	3.62E-09	4006	4500	3795	5153	2632
CFLAR	-0.86544	-0.71427	-1.04116	-0.6136	-1.2332	1.21E-06	2.57E-05	1.29E-08	0.000271	1.91E-09	4008	4284	3075	5182	2530
ARHGAP1	-0.73088	-1.01551	-0.6242	-0.83933	-0.57839	1.22E-06	8.92E-08	5.90E-05	2.67E-05	0.000314	4010	2972	5075	4475	5243
MRPL19	0.589411	0.540738	0.497931	0.563434	0.591978	1.22E-06	0.000208	8.67E-05	6.68E-05	1.70E-06	4011	4919	5188	4760	3849
STAMBP	0.516617	0.716803	0.938081	0.762087	0.636241	1.25E-06	9.78E-08	8.88E-11	4.28E-08	1.26E-07	4013	2989	2196	3015	3325
LPCAT3	0.688183	0.60278	0.575143	0.493763	0.432783	1.26E-06	8.84E-07	8.46E-06	9.48E-05	0.000299	4014	3449	4545	4841	5233
RPL26	1.371376	1.267058	1.125404	1.502974	1.428155	1.29E-06	1.00E-06	4.07E-05	2.73E-09	2.79E-07	4016	3479	4976	2564	3487
CLIP1	-0.51478	-0.33634	-0.51734	-0.35607	-0.52645	1.31E-06	0.000372	1.44E-08	0.000662	1.26E-08	4019	5146	3100	5476	2865
LAMTOR5	0.627673	0.462206	0.707208	0.628263	0.690041	1.31E-06	1.71E-05	1.61E-07	1.63E-06	8.55E-07	4020	4166	3614	3751	3699
SPAG7	-0.78495	-0.54622	-0.92065	-0.77101	-0.92306	1.32E-06	0.000514	7.77E-10	1.70E-06	7.88E-10	4021	5265	2539	3758	2386
DMXL2	-0.95932	-0.85567	-0.96616	-1.1245	-1.09228	1.32E-06	1.21E-05	7.95E-06	1.31E-07	1.48E-08	4022	4082	4530	3214	2891
CHRNB1	-0.89187	-0.66529	-0.96731	-0.74623	-0.97987	1.32E-06	4.75E-05	5.65E-11	1.24E-05	1.38E-09	4023	4466	2126	4268	2478
TMEM106	0.562541	0.563732	0.314693	0.642202	0.604677	1.32E-06	5.27E-06	0.002285	6.77E-06	2.14E-06	4024	3875	6258	4098	3898
OR51S1	-0.6105	-0.37535	-0.87005	-0.5306	-0.60218	1.32E-06	0.000782	2.19E-09	0.000899	8.19E-06	4025	5413	2721	5583	4228
FITM2	0.943391	1.096494	0.88234	1.325666	0.819226	1.33E-06	4.66E-07	3.94E-06	3.72E-09	8.49E-06	4026	3301	4340	2623	4241
EPRS1	0.695365	0.883348	0.71008	0.756351	0.823838	1.34E-06	7.91E-10	2.13E-07	1.60E-08	3.07E-09	4027	2141	3673	2845	2606
NENF	0.674056	0.579593	0.532086	0.610623	0.935066	1.34E-06	3.76E-06	7.24E-06	9.21E-07	4.18E-10	4028	3807	4500	3616	2277
C1QTNF3	-0.84721	-0.76025	-0.57171	-0.94629	-0.95646	1.34E-06	1.80E-05	0.004001	2.82E-09	2.32E-06	4030	4184	6479	2572	3928
CYP4V2	-0.6012	-0.74421	-0.63027	-0.67363	-0.4547	1.35E-06	4.70E-08	2.26E-05	2.21E-06	0.00072	4031	2839	4810	3825	5534
CREB3L2	-0.88992	-0.6584	-0.91295	-0.46364	-1.10608	1.35E-06	0.000258	1.94E-08	0.005136	1.75E-09	4032	5003	3165	6251	2518
NONO	0.699512	0.669984	0.427257	0.792521	0.708968	1.36E-06	3.64E-07	0.000228	2.72E-07	3.41E-07	4033	3254	5485	3356	3528
EXT1	-0.87351	-0.86071	-0.57173	-0.94854	-0.7385	1.36E-06	9.61E-07	0.000711	3.48E-08	5.49E-05	4034	3472	5852	2980	4744
NOB1	0.7615	0.874595	1.007652	1.07068	0.885136	1.37E-06	2.81E-07	4.23E-08	5.07E-09	1.33E-08	4035	3206	3337	2658	2872
CDK2AP1	-1.11828	-1.32205	-0.79771	-1.16857	-0.68437	1.38E-06	2.17E-11	0.000136	3.02E-08	0.001481	4038	1625	5302	2953	5786
TPM1	-1.47664	-1.94258	-1.2852	-1.46474	-1.26539	1.38E-06	1.12E-08	2.26E-05	1.75E-06	0.000845	4039	2572	4809	3765	5581
ATXN1	-1.05632	-1.17981	-1.28491	-1.00576	-1.06593	1.39E-06	2.49E-10	4.06E-11	1.43E-07	1.28E-07	4041	1959	2083	3232	3327
OXER1	-0.66306	-0.72237	-0.55999	-0.65606	-0.609	1.39E-06	2.79E-07	0.000231	2.48E-06	4.85E-06	4042	3203	5493	3852	4092
ISOC2	0.52207	0.384673	0.441255	0.430359	0.36093	1.39E-06	0.001067	0.0001	6.13E-05	0.000906	4043	5537	5218	4737	5599
UCHL5	0.666713	0.853331	0.665396	0.705302	0.608156	1.39E-06	7.79E-08	2.23E-06	3.21E-09	5.57E-05	4044	2938	4203	2600	4746
DDX5	-0.77639	-1.07629	-0.89689	-0.91677	-0.69738	1.40E-06	3.12E-08	6.35E-06	3.90E-06	4.74E-05	4045	2742	4464	3962	4706
ATXN3	-0.62778	-0.61382	-0.42983	-0.68652	-0.36226	1.40E-06	0.000212	0.001546	3.70E-05	0.001629	4046	4925	6096	4584	5829
ACLY	0.847905	0.677471	0.995947	0.923499	0.781786	1.40E-06	0.000984	3.52E-06	1.74E-05	1.42E-07	4047	5501	4311	4359	3344
PIK3CB	0.664909	0.726029	0.603366	0.576843	0.82145	1.40E-06	3.95E-06	9.77E-07	8.86E-06	7.01E-08	4048	3816	4029	4169	3199
CGB2	-0.85331	-0.41962	-1.25513	-0.80034	-1.23075	1.41E-06	0.003373	3.04E-09	0.000167	8.86E-10	4050	5943	2788	5000	2412
PCSK9	1.380069	1.940265	1.823941	1.798311	1.723742	1.42E-06	2.10E-10	2.90E-10	5.35E-10	1.29E-11	4051	1935	2380	2289	1801
NT5C	0.689737	0.573067	0.618415	0.480802	0.799234	1.42E-06	0.000612	6.21E-05	0.004274	3.37E-08	4052	5331	5091	6189	3043
CCNF	0.849273	0.882155	0.90793	0.952421	0.828213	1.42E-06	2.00E-08	1.58E-10	3.87E-09	3.67E-05	4053	2665	2292	2628	4627
MRPL1	0.557728	0.510481	0.618689	0.434461	0.632628	1.43E-06	0.000157	8.55E-06	0.000121	4.79E-06	4054	4825	4546	4916	4087
STAG2	0.662494	0.873254	0.683448	1.024675	0.672938	1.43E-06	9.81E-10	2.33E-06	6.71E-12	1.02E-07	4055	2171	4216	1708	3282

TOMM22	0.860234	0.969395	0.87135	0.95421	0.903403	1.43E-06	1.02E-07	1.58E-06	4.15E-09	3.37E-08	4056	3000	4139	2637	3042
TGOLN2	0.670671	0.56523	0.732338	0.835906	0.846726	1.44E-06	0.000101	3.18E-10	2.18E-06	6.41E-09	4058	4700	2391	3819	2722
TOP1MT	0.789061	1.408552	1.010958	1.329408	1.274797	1.44E-06	2.73E-12	2.37E-08	2.03E-11	8.80E-12	4059	1358	3193	1864	1746
BAIAP2L2	0.697355	0.764084	0.719978	0.67846	0.533118	1.45E-06	7.88E-07	1.82E-05	4.87E-06	0.0001	4060	3419	4760	4014	4930
GABPA	-0.51543	-0.47347	-0.29298	-0.55205	-0.46391	1.46E-06	7.88E-05	0.006488	4.03E-05	8.98E-05	4061	4627	6678	4607	4891
POP7	0.558171	0.751214	0.449699	0.551508	0.617046	1.47E-06	1.97E-09	0.001416	4.68E-05	4.11E-06	4062	2274	6075	4652	4053
MOG	-0.76812	-0.51553	-0.74331	-0.59668	-0.80938	1.47E-06	0.002958	4.60E-05	0.000743	1.18E-07	4063	5891	5014	5522	3313
VDAC3	0.723109	0.726054	0.961651	1.000413	0.78484	1.47E-06	6.83E-07	8.52E-09	8.80E-10	5.56E-06	4064	3383	2985	2371	4132
MANSC1	1.061588	1.305159	1.177354	1.316159	1.204039	1.48E-06	1.97E-06	6.70E-07	6.33E-07	3.12E-08	4065	3649	3934	3551	3028
ERBIN	-0.70893	-0.72493	-0.68803	-0.61756	-0.55716	1.48E-06	5.33E-07	3.42E-08	9.28E-06	0.000509	4066	3322	3297	4190	5413
NPIPB6	0.893628	0.63241	0.45462	0.830175	0.70362	1.50E-06	0.000135	0.002704	4.59E-05	2.29E-06	4068	4781	6328	4649	3923
FUCA2	0.722977	0.632532	1.045704	0.54272	0.663654	1.50E-06	0.000485	1.58E-08	0.000347	3.30E-05	4069	5240	3114	5263	4590
SC5D	0.592244	0.685614	0.854325	0.971369	0.493137	1.56E-06	2.51E-06	3.62E-07	4.26E-09	0.000285	4075	3713	3798	2640	5223
GPD1L	0.61076	0.786751	0.520256	0.763964	0.627831	1.56E-06	2.41E-09	2.59E-07	1.82E-08	3.35E-08	4076	2304	3716	2869	3040
IGBP1	0.62201	0.626916	0.521935	0.790683	0.3782	1.56E-06	2.79E-05	0.000107	3.10E-08	0.007667	4077	4312	5233	2955	6416
ICA1	1.158369	1.156141	1.195483	1.415297	1.494491	1.57E-06	3.54E-07	3.10E-08	1.69E-08	4.24E-11	4078	3248	3266	2858	1936
CDK5RAP2	-0.39798	-0.25936	-0.38018	-0.44069	-0.36243	1.59E-06	0.00262	2.57E-05	2.20E-06	0.00019	4080	5858	4850	3823	5111
THTPA	-0.62718	-0.39498	-0.62182	-0.43637	-0.60141	1.60E-06	0.002846	3.96E-09	0.00109	4.11E-07	4081	5875	2839	5658	3569
LMTK2	0.852046	0.60521	0.656873	0.809146	0.708295	1.62E-06	0.000938	2.27E-05	0.000148	0.000138	4082	5488	4811	4971	5022
ADM	-1.51474	-1.76921	-1.15259	-1.79982	-1.8279	1.62E-06	1.14E-06	6.46E-05	6.21E-09	2.71E-07	4084	3516	5101	2688	3480
FAM25A	-0.86844	-0.40717	-1.12544	-0.52879	-0.66411	1.63E-06	0.003692	1.13E-08	0.002101	0.000448	4085	5980	3041	5901	5373
AFTPH	0.555394	0.662173	0.683538	0.643502	0.461616	1.64E-06	1.13E-06	5.52E-09	1.86E-06	3.02E-05	4086	3510	2894	3786	4570
PRPF6	0.873605	1.063241	0.634881	1.227221	0.595272	1.66E-06	9.46E-08	7.55E-05	1.21E-08	4.15E-06	4087	2980	5138	2796	4061
SLC39A11	0.757691	0.540341	0.536212	0.715107	0.394217	1.68E-06	3.97E-05	1.14E-05	7.05E-06	0.002674	4088	4410	4625	4113	6000
COX14	0.674017	0.448787	0.601673	0.587402	0.624998	1.68E-06	0.000366	1.84E-05	4.92E-05	1.05E-06	4089	5140	4764	4671	3750
ZNF530	-0.61097	-0.4466	-0.5767	-0.57962	-0.57388	1.70E-06	0.000531	2.44E-05	2.55E-05	2.26E-06	4091	5282	4838	4459	3914
RUBCNL	1.5838	2.189436	2.167896	2.004611	2.026182	1.70E-06	5.72E-15	1.46E-10	1.92E-11	2.04E-08	4093	796	2276	1859	2944
GARRE1	-0.67923	-0.37522	-0.61642	-0.4591	-0.50806	1.71E-06	0.000284	3.06E-10	4.34E-05	3.86E-06	4094	5040	2388	4631	4041
KRT34	-0.61722	-0.53992	-1.00485	-0.91322	-0.88292	1.72E-06	1.15E-05	2.79E-07	1.68E-07	5.51E-07	4096	4066	3733	3265	3618
NBAS	-0.36451	-0.25805	-0.31749	-0.31848	-0.47078	1.73E-06	0.007105	0.000847	0.000178	3.14E-06	4098	6264	5918	5021	3992
TMEM14B	0.914435	0.891426	0.963041	0.894881	1.139791	1.74E-06	3.50E-07	8.43E-07	2.76E-08	4.56E-10	4102	3246	3994	2937	2294
ACVR1B	0.878759	0.67106	0.744554	0.638884	0.695657	1.76E-06	3.04E-05	2.61E-06	1.94E-05	1.98E-05	4103	4336	4245	4393	4463
CTNNBL1	0.823066	0.77748	0.633221	0.78835	0.653116	1.77E-06	7.15E-08	5.41E-06	2.95E-06	0.001232	4104	2916	4425	3900	5713
EXOSC4	0.668422	0.771562	0.775168	0.944526	0.555576	1.78E-06	3.39E-08	1.27E-07	8.72E-11	2.66E-06	4105	2762	3569	2049	3945
SVIP	0.587454	0.589101	0.714066	0.55455	0.575795	1.79E-06	2.70E-05	3.08E-08	7.38E-06	8.17E-05	4106	4306	3262	4120	4859
YY1	0.6474	0.554374	0.853842	0.5241	0.634063	1.83E-06	1.91E-05	6.93E-09	0.000134	1.12E-05	4107	4200	2938	4947	4310
SLC16A4	-0.77036	-0.52966	-0.64061	-0.58942	-0.47649	1.84E-06	5.38E-05	1.79E-05	3.65E-06	0.000418	4108	4509	4754	3937	5347
RNPEP	0.527946	0.322565	0.575395	0.325351	0.514163	1.84E-06	0.001857	1.42E-07	0.00214	6.79E-08	4109	5730	3595	5913	3186
SP7	-0.87008	-0.49927	-1.03179	-0.76738	-1.10619	1.85E-06	0.003031	5.24E-08	0.000114	3.27E-08	4110	5900	3386	4888	3036
INTS7	0.4045	0.517942	0.432299	0.510414	0.737437	1.85E-06	4.74E-07	2.41E-05	0.000116	6.06E-06	4111	3304	4832	4897	4152
RPF2	0.728431	0.899531	0.989019	0.935289	1.042593	1.85E-06	9.39E-09	8.18E-08	1.07E-08	1.22E-10	4112	2544	3464	2774	2079
SLC25A25	-0.42619	-0.51722	-0.25546	-0.49972	-0.39226	1.86E-06	1.28E-07	0.002101	1.76E-07	1.35E-05	4113	3050	6219	3272	4371
FBXO9	0.491635	0.629234	0.621513	0.575755	0.434275	1.87E-06	1.67E-06	1.60E-07	5.43E-05	0.001028	4114	3611	3612	4695	5640
MRPS7	0.700193	0.555627	0.724228	0.478568	0.372666	1.89E-06	5.72E-05	1.12E-07	0.000352	0.005444	4117	4521	3547	5270	6270
YBX2	1.096369	1.245068	1.212727	0.827956	0.960185	1.90E-06	2.72E-07	7.48E-09	0.000117	9.65E-06	4118	3198	2950	4902	4270
CRYL1	0.871998	0.672302	0.810631	0.723049	1.184481	1.91E-06	6.36E-06	2.74E-07	9.68E-06	2.18E-10	4119	3916	3727	4201	2180
C8orf74	-0.65459	-0.34991	-0.73999	-0.46688	-0.66381	1.91E-06	0.00334	8.03E-09	0.001083	8.05E-08	4120	5939	2967	5655	3231
CUX1	0.686971	0.802757	0.985174	1.082716	0.653169	1.92E-06	4.23E-06	3.27E-07	1.42E-09	1.60E-05	4121	3828	3776	2464	4413
PNPO	0.859775	0.7669	0.984895	0.807835	0.610397	1.96E-06	1.62E-06	1.25E-11	3.39E-06	1.86E-06	4123	3603	1902	3921	3867
PSMC5	0.579042	0.406256	0.777769	0.66399	0.381643	1.96E-06	0.00556	1.68E-05	8.26E-05	0.004579	4124	6151	4737	4810	6186
MMP3	-2.47427	-2.48084	-2.62424	-3.55166	-4.28716	1.96E-06	3.99E-05	4.31E-05	1.56E-07	2.34E-11	4125	4415	4997	3250	1872
TIMM10B	0.754276	0.568983	0.741788	0.478747	0.564275	2.00E-06	0.000981	1.68E-06	0.002615	0.000157	4128	5499	4153	5991	5055
ARHGEF37	-0.59038	-0.61877	-0.61301	-0.90608	-0.65937	2.00E-06	4.32E-05	2.60E-05	3.68E-09	2.06E-05	4129	4441	4856	2621	4472
TXLNG	0.786039	0.695794	0.645135	0.72659	0.566195	2.02E-06	2.08E-05	0.000488	1.50E-05	0.001445	4130	4224	5734	4324	5770
MVB12B	-0.53774	-0.42436	-0.46929	-0.48517	-0.42317	2.04E-06	0.003975	0.001025	0.001958	0.000499	4132	6011	5960	5871	5402
IP6K1	-0.61483	-0.68427	-0.46406	-0.64701	-0.67002	2.04E-06	7.00E-06	6.73E-05	1.58E-05	5.51E-08	4133	3938	5114	4343	3145
SLC2A9	-0.581	-0.60676	-0.61215	-0.77915	-0.64621	2.07E-06	9.48E-07	7.49E-07	1.27E-08	2.90E-08	4137	3469	3962	2800	3015
INTS8	0.67074	0.580139	0.800936	0.630727	0.54194	2.07E-06	1.16E-05	3.52E-07	0.000176	1.22E-05	4138	4069	3791	5014	4337
UQCR10	0.707879	0.674291	0.836674	0.540168	0.884487	2.07E-06	3.94E-09	2.31E-08	1.64E-05	3.41E-09	4139	2396	3189	4350	2624
POLR2F	0.789865	1.069395	0.815391	1.061256	0.907807	2.07E-06	6.44E-09	2.62E-08	7.52E-10	9.04E-11	4140	2483	3226	2352	2035
PLSCR1	0.848527	0.542861	1.305232	0.955198	0.843582	2.07E-06	0.002017	2.61E-09	3.00E-08	3.51E-06	4141	5772	2761	2952	4020
ARHGEF1	-0.80433	-0.84884	-1.31008	-1.14326	-0.86042	2.07E-06	1.49E-05	9.15E-09	6.38E-07	1.30E-05	4142	4133	2994	3555	4363
AUP1	0.839879	1.032728	1.020122	0.903852	1.016011	2.08E-06	3.82E-07	3.62E-07	6.92E-06	2.79E-08	4143	3265	3797	4105	3008
BIRC2	-0.63527	-0.60405	-0.54117	-0.52649	-0.99191	2.08E-06	4.86E-06	0.000158	0.000595	2.33E-09	4144	3856	5350	5430	2573
NCBP2AS2	0.650344	0.786815	0.603101	0.710401	0.722215	2.08E-06	1.08E-09	8.94E-08	8.34E-08	1.56E-07	4145	2187	3486	3135	3364
TCF7L2	0.952143	0.994766	1.354989	1.333336	1.474592	2.15E-06	3.65E-07	8.05E-11	2.27E-09	2.13E-10	4148	3255	2172	2537	2172
NDUFA11	0.632979	0.632648	0.794026	0.744151	0.634146	2.15E-06	1.10E-05	7.39E-08	6.50E-07	0.000328	4149	4053	3449	3559	5253
DSN1	1.006703	0.88447	0.682718	1.077199	0.646293	2.16E-06	1.08E-05	9.74E-06	9.34E-07	0.001096	4150	4047	4581	3621	5669
ICAM3	-0.76776	-0.58405	-1.09681	-0.75901	-0.98081	2.16E-06	0.000145	6.02E-08	3.66E-05	5.35E-07	4151	4803	3408	4578	3613
RHD	-0.83897	-0.56754	-0.71811	-0.77088	-0.86176	2.16E-06	0.005258	0.000278	0.000277	2.42E-05	4152	6123	5549	5193	4510
GLTP	-0.80259	-0.60031	-0.4855	-0.58734	-0.66703	2.18E-06	0.00192	0.008229	0.000303	6.00E-05	4153	5744	6776	5225	4766
RAB4A	0.784106	0.52316	0.62349	0.523512	0.888606	2.18E-06	0.005445	0.000245	0.005853	3.50E-07	4155	6142	5510	6302	3535
C1GALT1	0.916059	1.037591	1.068761	1.051844	0.799456	2.19E-06	4.42E-07	4.84E-09	9.03E-08	1.45E-06	4156	3286	2870	3150	3811

GCNT3	1.811294	1.364684	2.419564	1.487825	1.682442	2.19E-06	5.01E-05	6.41E-10	5.63E-06	1.15E-07	4157	4484	2504	4053	3307
IFIT2	-0.73574	-0.55481	-0.89761	-0.64569	-1.08041	2.21E-06	0.000484	1.08E-10	0.000156	8.37E-18	4158	5238	2227	4986	486
NUTM2F	-0.71005	-0.42032	-0.84321	-0.70994	-0.89252	2.21E-06	0.000545	2.37E-09	9.71E-06	3.42E-06	4159	5292	2736	4202	4010
C7orf50	0.937397	1.054963	1.072978	1.134597	1.357281	2.21E-06	3.22E-09	3.21E-09	2.17E-10	6.22E-11	4160	2365	2807	2163	1977
SRSF7	0.76345	0.593218	0.735426	0.884698	1.012301	2.22E-06	0.000205	0.000253	3.75E-07	2.20E-08	4161	4917	5519	3430	2963
EMD	0.713641	0.728099	0.714649	0.928412	0.568029	2.24E-06	3.36E-08	7.05E-06	1.15E-09	9.65E-05	4162	2760	4491	2417	4917
IKZF5	-0.41155	-0.35559	-0.53101	-0.4387	-0.54698	2.24E-06	3.22E-05	1.53E-08	1.42E-06	4.44E-08	4163	4349	3106	3715	3101
PHC2	-0.87203	-0.89066	-0.50646	-0.76788	-0.8039	2.26E-06	2.00E-08	0.002878	1.37E-05	3.86E-06	4164	2666	6343	4293	4040
BCCIP	0.732629	0.462781	0.713107	0.527691	0.718588	2.28E-06	0.001427	1.58E-06	0.000519	4.84E-05	4166	5640	4138	5394	4711
SLC37A4	0.801092	0.995494	0.644194	0.880878	0.574044	2.29E-06	4.67E-10	1.54E-05	1.07E-08	0.000559	4167	2054	4711	2772	5438
PLAC8L1	-0.66781	-0.36989	-0.75302	-0.59934	-0.8137	2.32E-06	0.005357	6.70E-07	0.000243	2.13E-08	4168	6130	3935	5132	2954
DAG1	0.733398	0.599753	0.819626	0.837083	0.786897	2.33E-06	0.000104	3.41E-07	3.84E-06	4.73E-06	4170	4710	3785	3955	4083
TSC22D1	1.192836	0.818232	1.354168	1.009304	1.202029	2.33E-06	0.001967	7.64E-08	0.000198	7.11E-08	4172	5759	3452	5056	3205
HNRNPA0	0.608677	0.633189	0.785681	0.699101	0.547304	2.34E-06	6.53E-07	3.22E-08	1.57E-08	4.84E-05	4173	3371	3276	2839	4712
ANKS4B	1.028769	1.145362	1.276127	1.168316	0.720932	2.36E-06	2.19E-07	1.68E-09	7.73E-08	0.000403	4176	3165	2658	3116	5330
VCP	0.635035	0.516856	0.698298	0.740207	0.562994	2.37E-06	0.000807	3.26E-05	3.24E-05	3.01E-06	4177	5425	4917	4543	3981
MICOS13	0.749542	1.080735	1.111857	1.065292	0.792679	2.37E-06	2.57E-09	9.38E-12	1.88E-10	9.45E-08	4178	2317	1864	2140	3265
EIF2S1	0.710489	0.702838	1.016691	0.577041	0.553958	2.38E-06	5.94E-06	4.70E-10	0.000944	0.000985	4179	3901	2455	5605	5626
ZFYVE26	-0.55894	-0.33056	-0.60877	-0.62964	-0.67564	2.40E-06	0.006866	7.36E-06	3.29E-05	1.94E-08	4181	6245	4505	4552	2935
MFSD11	-0.53181	-0.70243	-0.64957	-0.68993	-0.63708	2.41E-06	6.30E-08	1.72E-08	9.67E-07	1.65E-08	4182	2889	3135	3631	2909
TMEM184	-0.66354	-0.47907	-0.30402	-0.48679	-0.58834	2.41E-06	0.000685	0.00691	0.000486	2.97E-06	4183	5374	6699	5363	3975
ZNF845	-0.68855	-0.45061	-0.97786	-0.64501	-0.83304	2.44E-06	0.000196	6.09E-11	5.34E-05	3.57E-08	4185	4906	2136	4688	3052
ANTXR2	-1.45518	-1.2259	-0.85612	-1.30668	-1.10063	2.44E-06	5.37E-06	0.001139	3.13E-09	4.74E-05	4186	3882	5994	2592	4708
ANO1	-1.45307	-1.45576	-1.14533	-1.39097	-1.6222	2.45E-06	1.77E-08	0.000145	7.76E-08	1.92E-08	4187	2640	5322	3117	2933
UBE2L6	-0.89727	-1.14826	-0.61526	-0.8359	-1.15142	2.45E-06	5.11E-06	0.009417	0.000707	1.55E-05	4188	3864	6825	5500	4405
ZNRF1	-0.65478	-0.60282	-0.62843	-0.71785	-0.42016	2.49E-06	6.42E-06	4.83E-06	4.77E-07	0.002002	4190	3918	4404	3485	5910
ATG13	-0.61404	-0.44631	-0.4758	-0.44691	-0.41944	2.52E-06	0.000215	6.38E-07	0.000188	0.000751	4192	4932	3925	5044	5545
XIAP	0.642796	0.686895	0.808408	0.973387	0.439673	2.52E-06	1.73E-07	8.96E-07	1.27E-09	0.000174	4193	3122	4006	2428	5082
NABP1	-1.00093	-0.60488	-1.30569	-0.75682	-1.34924	2.58E-06	0.000709	4.01E-10	0.000249	9.03E-09	4196	5382	2433	5141	2793
G3BP1	0.818124	0.702946	0.901695	0.723366	1.077032	2.59E-06	4.37E-05	1.15E-07	2.70E-05	1.28E-10	4197	4443	3554	4479	2084
ZNF117	-0.61377	-0.28967	-0.69093	-0.53651	-0.4642	2.59E-06	0.008851	1.24E-06	3.99E-05	0.000562	4198	6378	4090	4603	5440
CDC16	0.80437	0.977161	0.933959	0.886072	0.994897	2.60E-06	3.85E-08	1.97E-08	3.63E-09	2.10E-10	4201	2793	3166	2617	2168
TP53RK	0.773585	0.802242	1.034389	0.979077	1.082664	2.62E-06	4.16E-07	1.02E-07	1.78E-07	3.50E-08	4202	3275	3520	3275	3047
EWSR1	0.542234	0.429411	0.559665	0.606474	0.622354	2.62E-06	0.000484	6.44E-05	1.46E-05	1.76E-05	4203	5239	5100	4316	4435
NABP2	0.698592	0.820867	0.529257	0.716944	0.475422	2.63E-06	7.59E-08	8.95E-06	1.12E-06	0.001679	4204	2933	4560	3666	5842
SCYL1	-0.5972	-0.51045	-0.54577	-0.48399	-0.68403	2.66E-06	2.87E-06	7.82E-07	1.32E-05	3.48E-09	4205	3750	3971	4280	2628
LGALS3BP	1.474478	0.930821	1.741919	1.233446	1.388453	2.67E-06	0.000542	9.46E-08	4.50E-06	9.17E-07	4207	5289	3501	3992	3717
DMRTA2	-0.61203	-0.36674	-0.91408	-0.44628	-0.8494	2.69E-06	0.001431	4.52E-09	0.002269	8.42E-09	4208	5641	2858	5934	2772
METAP1	0.695104	0.657875	0.832079	0.510943	0.868688	2.72E-06	8.63E-06	1.58E-10	2.79E-05	2.10E-11	4210	3995	2293	4492	1864
ITGB1	-1.26797	-1.81567	-0.90083	-1.39164	-1.16741	2.72E-06	1.43E-12	0.000177	4.51E-07	1.31E-06	4211	1285	5398	3478	3793
CAPN5	0.859756	0.921956	0.695595	0.943343	0.603476	2.73E-06	3.97E-07	2.88E-05	3.52E-07	7.26E-05	4212	3271	4875	3416	4833
SF3B4	0.694451	0.673398	0.750962	0.770592	0.565324	2.73E-06	0.000816	1.41E-05	8.57E-05	3.05E-05	4213	5429	4688	4816	4573
ACTR3B	0.690814	0.78478	0.798037	0.885247	0.612531	2.73E-06	1.12E-05	7.62E-11	6.63E-07	7.32E-05	4214	4058	2165	3562	4835
METRN	-0.60123	-0.46765	-0.68085	-0.54497	-0.68454	2.75E-06	4.77E-05	1.20E-08	3.82E-06	5.70E-07	4215	4470	3059	3951	3625
SNRPA1	0.583149	0.711198	0.46769	0.684624	0.974039	2.76E-06	1.83E-07	6.17E-05	1.59E-07	1.02E-07	4216	3131	5089	3253	3283
LARP4B	0.754129	0.839875	1.376895	0.706628	1.224884	2.77E-06	5.22E-06	5.93E-10	1.48E-05	6.82E-10	4217	3872	2494	4321	2365
GAL3ST2	1.217238	1.457647	0.976171	1.405714	1.36481	2.78E-06	5.48E-08	7.80E-05	4.50E-06	3.28E-05	4218	2864	5149	3993	4589
DHRS11	0.806123	0.675773	0.987427	0.694739	1.030849	2.83E-06	1.64E-05	5.19E-09	3.54E-06	3.99E-10	4219	4149	2881	3928	2266
SNN	-0.66308	-0.77747	-1.02068	-0.81242	-0.46825	2.91E-06	7.26E-10	2.32E-13	1.62E-09	0.007326	4221	2119	1435	2485	6395
SLC16A10	-0.72613	-0.56658	-0.83858	-0.92292	-0.90248	2.91E-06	0.00016	2.92E-07	3.87E-08	1.82E-07	4222	4831	3741	3001	3393
EPN2	-0.83576	-0.56987	-0.60722	-0.67348	-0.86393	2.95E-06	0.000579	0.000169	0.000129	1.07E-07	4225	5314	5372	4934	3291
IPO9	0.460979	0.562925	0.447905	0.432291	0.40222	2.99E-06	5.83E-07	3.27E-06	9.92E-06	0.000255	4228	3346	4297	4212	5193
HERPUD1	-0.96003	-1.13341	-1.43132	-1.18243	-1.2592	3.00E-06	1.20E-08	1.02E-09	9.25E-06	1.70E-07	4229	2587	2582	4189	3382
IFIH1	-0.64075	-0.78705	-0.95687	-0.71884	-0.85249	3.01E-06	3.16E-07	6.10E-08	7.39E-06	1.19E-08	4230	3230	3411	4122	2853
RRP36	0.498625	0.441164	0.503571	0.474555	0.335734	3.02E-06	0.001001	0.000149	0.00011	0.001855	4231	5506	5332	4882	5881
EFNA2	0.906217	0.878177	1.223656	0.896239	0.77083	3.08E-06	5.31E-06	8.33E-08	8.14E-07	1.64E-05	4234	3879	3467	3595	4419
HOXB8	1.540869	1.755815	1.904749	2.176474	2.287071	3.09E-06	3.20E-08	2.15E-08	2.80E-10	6.37E-12	4235	2747	3181	2196	1701
WWP2	0.679225	0.699732	0.802401	0.7125	0.915481	3.10E-06	2.77E-06	4.74E-07	7.69E-08	6.87E-09	4236	3738	3856	3113	2735
AP1AR	0.760713	0.684716	1.020097	0.80302	1.157862	3.12E-06	8.87E-06	3.21E-11	9.31E-07	5.60E-11	4237	4007	2056	3619	1959
BRCC3	0.768761	1.040882	0.841508	1.293158	0.794252	3.12E-06	1.06E-07	7.89E-06	3.52E-12	5.83E-07	4238	3009	4522	1636	3634
KIAA2013	0.504316	0.553753	0.773061	0.581791	0.858655	3.13E-06	2.62E-06	5.11E-09	3.66E-06	8.13E-09	4239	3720	2878	3939	2770
NCR3LG1	0.921784	0.869636	0.74925	0.677074	0.586639	3.14E-06	0.000136	0.000699	0.00269	0.004807	4240	4785	5844	6003	6208
THOC2	0.63362	0.623732	0.952051	0.792509	0.532014	3.15E-06	2.71E-06	3.08E-07	1.16E-07	0.000411	4241	3733	3767	3193	5339
TOP3A	-0.47949	-0.51415	-0.74967	-0.62616	-0.83076	3.16E-06	3.67E-06	1.09E-08	5.36E-07	1.72E-09	4242	3803	3030	3512	2512
KCNQ1	1.393046	1.412144	1.430749	1.607901	2.170577	3.18E-06	1.28E-06	2.38E-06	2.79E-07	9.31E-13	4245	3542	4221	3362	1474
UTP4	0.750509	0.69462	0.886688	0.686494	0.818699	3.22E-06	2.81E-05	2.54E-08	1.39E-05	1.56E-06	4246	4315	3217	4302	3833
ITSN1	-0.58896	-0.45499	-0.3989	-0.52131	-0.78936	3.23E-06	0.000839	0.00303	7.87E-05	1.24E-05	4247	5439	6357	4799	4343
PCGF2	-0.76942	-0.73912	-0.44266	-0.87232	-0.65167	3.23E-06	7.86E-07	0.00085	1.01E-09	6.43E-05	4248	3417	5920	2395	4793
CHID1	0.908106	1.020282	0.735416	1.099657	0.825508	3.23E-06	4.60E-06	0.00019	3.13E-07	1.04E-06	4249	3844	5419	3388	3748
SPON1	-1.34185	-1.22133	-0.80634	-1.14286	-1.67134	3.24E-06	2.40E-06	0.002135	1.91E-05	2.18E-08	4250	3701	6227	4386	2959
KCNN4	1.040049	0.978334	1.061164	0.906442	0.666191	3.26E-06	0.000185	8.10E-06	2.17E-05	0.003378	4251	4891	4534	4412	6069
CHCHD1	0.57818	0.672073	0.657227	0.702774	0.747934	3.27E-06	1.35E-07	1.40E-07	3.54E-07	6.65E-07	4252	3061	3591	3417	3660
SLC2A5	-0.73798	-0.8129	-1.18376	-1.0842	-1.10228	3.29E-06	7.41E-06	1.88E-09	5.90E-07	1.03E-09	4255	3949	2684	3532	2426

BRD7	0.719573	0.743654	0.601429	0.741994	0.601746	3.29E-06	5.89E-07	3.73E-05	6.63E-08	1.25E-05	4256	3351	4951	3084	4350
MARCHF6	0.740419	0.770138	0.744777	0.736956	0.564218	3.31E-06	1.41E-07	1.19E-08	8.29E-06	0.000237	4257	3072	3054	4157	5170
ZNF326	0.738074	0.773541	0.606306	0.91567	0.559368	3.31E-06	7.54E-07	2.87E-06	3.13E-09	8.05E-08	4258	3406	4266	2593	3232
SMARCC2	0.577059	0.454285	0.302207	0.441487	0.332524	3.31E-06	4.00E-05	0.002836	0.000284	0.005618	4259	4417	6340	5198	6291
BANF1	0.872781	0.978067	0.999338	1.191262	0.795984	3.33E-06	2.70E-08	1.52E-06	7.22E-13	2.11E-06	4260	2722	4131	1462	3894
ECHDC1	0.573947	0.616294	0.652664	0.727633	0.668557	3.33E-06	3.78E-05	1.25E-05	1.07E-05	3.73E-08	4261	4398	4661	4230	3059
MAP3K11	-0.48908	-0.43438	-0.26998	-0.44426	-0.58899	3.35E-06	8.29E-07	0.001789	4.14E-06	6.87E-06	4262	3428	6147	3973	4178
DNASE2	-0.56067	-0.47911	-0.85964	-0.66353	-0.73935	3.36E-06	2.20E-05	1.58E-08	5.33E-06	4.57E-07	4264	4241	3111	4038	3586
RABIF	-0.47911	-0.48313	-0.74739	-0.69091	-0.53952	3.38E-06	3.28E-06	1.08E-10	1.74E-08	0.001335	4265	3778	2228	2864	5742
SPTAN1	0.746988	0.563704	0.875223	0.520547	0.547174	3.39E-06	0.000118	8.75E-08	0.000658	4.20E-05	4266	4740	3480	5474	4671
POLR3E	0.834671	0.915497	0.482361	0.998057	0.643322	3.40E-06	4.73E-07	0.000171	1.80E-07	2.80E-05	4267	3303	5379	3277	4542
BEX3	-1.07295	-0.73205	-1.12316	-0.82795	-0.91255	3.40E-06	2.56E-05	1.86E-08	2.30E-05	9.10E-05	4268	4283	3154	4423	4896
ABHD17A	-0.75194	-0.58031	-0.75511	-0.52891	-0.59338	3.42E-06	0.000407	2.48E-07	0.000761	0.000555	4270	5179	3705	5530	5435
NDUFB1	0.557841	0.454545	0.595537	0.452159	0.364202	3.45E-06	0.000185	3.08E-06	0.000181	0.004507	4272	4886	4288	5033	6180
IER5	-0.70674	-0.61479	-0.85819	-0.63698	-0.63012	3.47E-06	5.83E-06	6.90E-10	0.001136	0.002512	4274	3899	2518	5670	5983
RACGAP1	0.685144	0.762304	0.774689	0.508167	0.584039	3.48E-06	8.92E-06	1.09E-06	0.00038	4.56E-05	4275	4009	4055	5292	4697
STAMBPL1	-0.47484	-0.31173	-0.33191	-0.40432	-0.27983	3.48E-06	0.001921	3.01E-05	4.75E-05	0.005888	4276	5745	4894	4660	6307
NOP9	0.85547	0.562365	0.739453	0.502494	0.544756	3.48E-06	0.00068	6.76E-06	0.001685	0.003899	4277	5371	4480	5823	6112
DDX47	0.652464	0.558874	0.582531	0.592064	0.740093	3.52E-06	0.000143	0.000181	6.14E-05	5.37E-07	4278	4799	5406	4738	3616
CDK5RAP3	0.839605	0.844549	0.538959	0.895354	0.907671	3.53E-06	1.95E-06	5.83E-05	4.32E-06	1.50E-06	4279	3648	5073	3985	3818
H2BC15	1.364263	1.54855	1.122609	1.575959	1.673076	3.53E-06	6.23E-07	0.000174	5.80E-10	4.74E-07	4280	3360	5387	2303	3597
NDEL1	-0.7727	-0.92339	-0.86011	-0.94608	-0.84468	3.54E-06	4.72E-06	1.14E-06	5.21E-06	2.20E-07	4281	3852	4075	4032	3437
INPP5E	-0.6125	-0.56257	-0.85179	-0.88889	-0.58923	3.54E-06	2.81E-05	1.84E-09	9.71E-09	4.96E-06	4282	4314	2681	2759	4101
NOTCH2	-0.87875	-0.74795	-1.14111	-0.92516	-1.07788	3.54E-06	3.42E-07	5.72E-12	1.53E-07	1.03E-10	4284	3242	1801	3246	2058
TMA7	0.509677	0.806173	0.44557	0.6779	0.623484	3.55E-06	4.40E-09	5.72E-05	1.20E-08	4.66E-08	4285	2411	5068	2795	3111
FBXW7	-0.59775	-0.57057	-0.73119	-0.73038	-0.89293	3.56E-06	6.14E-05	2.62E-08	5.99E-07	5.17E-08	4286	4541	3225	3536	3133
CMTM1	-0.56523	-0.54063	-0.40401	-0.48904	-0.55158	3.56E-06	5.28E-05	0.000373	0.000128	5.84E-06	4288	4504	5644	4932	4147
RPL13A	0.991219	0.85035	1.028582	1.004923	0.631649	3.57E-06	3.62E-06	1.15E-05	1.41E-06	0.001399	4289	3792	4630	3714	5757
CENPV	1.351898	1.739417	1.357237	1.61654	0.97572	3.57E-06	2.00E-07	1.43E-06	1.94E-07	0.000303	4290	3148	4118	3294	5236
CMBL	1.000052	0.834774	1.212414	0.863814	0.827122	3.58E-06	4.21E-05	4.26E-08	0.000125	6.23E-05	4291	4432	3339	4922	4784
PPP1R7	0.570757	0.585044	0.666877	0.768485	0.635833	3.58E-06	8.67E-05	7.68E-06	9.83E-06	7.46E-07	4293	4654	4517	4211	3676
ANAPC7	0.752869	0.71533	0.437744	0.895691	0.497278	3.61E-06	5.95E-06	0.000654	6.16E-08	9.90E-05	4294	3902	5814	3072	4924
TIMM17A	0.649778	0.608093	0.669868	0.531758	0.668472	3.63E-06	4.69E-05	8.11E-06	0.000145	3.51E-06	4296	4460	4536	4968	4021
C16orf87	-0.58297	-0.713	-0.80796	-0.81298	-0.84512	3.66E-06	1.03E-06	2.09E-07	5.95E-08	9.91E-09	4298	3486	3666	3065	2811
LY75	1.032361	0.836239	0.773037	1.097611	0.822615	3.67E-06	0.000344	1.93E-05	2.85E-06	2.27E-05	4299	5113	4771	3888	4496
GALNT18	-0.88515	-0.61166	-0.67002	-0.76883	-0.76721	3.70E-06	0.000275	0.000157	1.57E-05	2.93E-05	4300	5026	5346	4340	4554
PRSS23	-1.90017	-1.98874	-2.20703	-1.97378	-2.01681	3.73E-06	8.06E-06	2.72E-10	1.01E-07	1.73E-07	4301	3971	2368	3168	3384
SHFL	-0.64851	-0.34748	-0.66966	-0.41805	-0.71438	3.74E-06	0.009898	4.52E-06	0.000473	3.02E-07	4302	6424	4382	5351	3506
ACSL3	0.790217	0.702291	1.10656	1.012849	0.93481	3.77E-06	1.03E-07	3.72E-10	2.11E-09	2.75E-08	4303	3003	2419	2522	3004
HSPA13	-0.85995	-0.70403	-0.72506	-0.79	-1.21773	3.77E-06	8.18E-05	0.000207	0.000249	1.09E-08	4304	4638	5451	5142	2832
RYK	0.538788	0.746957	0.492708	0.681751	0.700362	3.80E-06	3.23E-07	0.000214	7.02E-07	1.80E-10	4305	3232	5462	3572	2136
P2RY11	-0.52052	-0.3365	-0.67315	-0.42303	-0.3979	3.83E-06	0.000234	9.67E-09	0.000237	0.00103	4306	4965	3007	5123	5642
RPL22L1	0.934535	1.562017	0.92694	1.029378	0.897083	3.91E-06	1.84E-08	7.23E-06	1.17E-05	2.44E-05	4308	2646	4499	4258	4512
BHLHE41	-1.34617	-1.12006	-1.17838	-1.58854	-1.69072	3.92E-06	0.000169	0.000526	9.21E-07	4.63E-08	4309	4856	5762	3615	3110
INPPL1	-0.54142	-0.4929	-0.51132	-0.53986	-0.62701	3.93E-06	0.000378	5.18E-05	3.33E-05	5.77E-05	4310	5150	5045	4555	4756
TVP23B	-0.69538	-0.94118	-0.69166	-0.72384	-0.72324	3.97E-06	2.12E-08	3.66E-06	0.000112	4.28E-05	4311	2675	4322	4883	4675
PPIAL4F	0.719377	0.786269	0.604468	0.748487	1.201092	3.99E-06	9.30E-07	1.16E-05	5.66E-06	6.42E-09	4315	3464	4631	4054	2725
AZIN1	0.759004	0.835973	0.837863	0.906849	0.523166	4.00E-06	6.72E-06	6.40E-06	2.88E-06	0.000438	4316	3926	4466	3892	5361
MFSD3	0.809559	0.989364	1.202923	1.069905	0.730923	4.04E-06	2.93E-09	3.79E-09	1.58E-08	2.92E-05	4317	2344	2834	2840	4549
RBM42	0.553795	0.539907	0.752895	0.375058	0.9187	4.05E-06	0.000555	1.60E-07	0.009389	5.86E-12	4318	5302	3613	6504	1690
BTBD2	0.502159	0.563459	0.753763	0.795581	0.592833	4.11E-06	1.66E-05	3.27E-05	1.69E-07	6.16E-05	4322	4152	4920	3266	4780
TBL3	0.721033	0.6041	0.69664	0.61145	0.503744	4.12E-06	0.000241	5.01E-05	3.33E-05	0.001261	4323	4975	5038	4557	5721
TMEM170	0.639933	0.775149	0.582361	0.795362	0.468776	4.19E-06	4.46E-07	1.07E-05	1.31E-08	6.76E-06	4328	3292	4613	2812	4175
ARSA	-0.67651	-0.57223	-0.81037	-0.63787	-0.9178	4.28E-06	1.35E-05	1.19E-10	1.11E-06	3.11E-12	4331	4108	2241	3664	1618
PSMD1	0.555569	0.645411	0.793465	0.681451	0.565733	4.31E-06	2.64E-08	1.57E-10	1.70E-07	4.91E-08	4332	2716	2290	3270	3119
FGFRL1	0.963633	1.241353	0.832034	1.011787	1.001524	4.32E-06	1.19E-08	1.27E-05	3.77E-07	1.02E-07	4333	2586	4670	3432	3279
LONP1	0.66467	0.703928	0.588027	0.616861	0.721047	4.33E-06	2.94E-06	3.36E-05	1.93E-06	3.89E-10	4334	3754	4925	3799	2263
TMEM248	0.714348	0.530359	0.869293	0.628618	0.839374	4.33E-06	0.000465	1.67E-07	7.12E-05	6.16E-07	4335	5219	3623	4774	3644
NDUFB7	0.71466	1.02605	0.494894	1.108221	0.63198	4.34E-06	1.30E-07	0.003141	4.62E-11	4.07E-05	4336	3054	6372	1963	4666
CBX4	0.697963	0.944463	0.497813	0.767026	0.401394	4.35E-06	8.45E-09	0.000211	9.63E-07	0.009348	4337	2527	5459	3630	6532
LIPG	0.601138	0.807092	1.196129	0.921545	1.216759	4.40E-06	3.00E-07	2.64E-08	1.53E-06	1.35E-07	4339	3219	3228	3738	3337
CASP6	0.623719	0.601833	0.974784	0.517712	1.127654	4.42E-06	0.000172	4.02E-09	0.000975	1.81E-11	4340	4864	2841	5618	1846
TNFRSF1B	-0.88274	-0.89068	-1.15889	-0.79392	-1.32844	4.43E-06	1.14E-07	4.93E-10	1.65E-08	1.92E-10	4341	3025	2462	2854	2149
RAB6C	-0.68897	-0.43728	-0.66399	-0.5293	-0.56937	4.45E-06	0.003149	5.75E-06	0.000668	7.95E-05	4342	5917	4440	5480	4852
WDR90	1.09924	1.400163	1.306507	1.419678	1.297989	4.46E-06	2.48E-10	3.20E-10	4.09E-11	3.94E-09	4343	1958	2395	1951	2650
STAT1	-0.78335	-1.06632	-0.61197	-0.83939	-0.82886	4.46E-06	1.29E-07	0.000564	1.38E-05	4.13E-06	4344	3052	5778	4297	4059
AKR7A3	0.486975	0.404914	0.50147	0.553322	1.049789	4.48E-06	0.003694	0.000272	0.001394	7.52E-08	4345	5982	5545	5754	3218
TMEM30A	-0.53286	-0.34934	-0.72784	-0.47721	-0.45028	4.52E-06	0.005045	2.10E-08	0.001125	0.000354	4347	6102	3175	5668	5277
SLC35B2	0.502329	0.560657	0.522149	0.569958	0.400875	4.53E-06	3.59E-06	0.000597	9.83E-06	0.002059	4348	3791	5790	4210	5917
BNIP3	-1.47894	-1.18504	-0.74663	-0.9779	-1.37914	4.54E-06	0.000471	0.00049	0.005924	3.01E-05	4349	5223	5737	6304	4566
PGP	0.730116	0.623786	0.621403	0.656596	0.681831	4.55E-06	4.82E-05	1.91E-05	3.29E-05	1.86E-05	4350	4474	4767	4551	4450
BNIP2	-0.63425	-0.93405	-0.8159	-0.83326	-0.77016	4.55E-06	4.90E-07	2.33E-07	8.04E-07	3.84E-06	4351	3309	3692	3591	4038
MRPL50	0.540782	0.803692	0.671933	0.741394	0.428682	4.55E-06	2.54E-08	5.88E-07	1.39E-05	3.65E-05	4352	2708	3905	4301	4624

USP9X	0.537015	0.496981	0.303327	0.677079	0.32731	4.57E-06	8.85E-05	0.002576	6.14E-06	0.006358	4353	4665	6306	4070	6332
ARFGAP3	-1.05855	-1.14263	-0.59469	-0.87746	-1.09256	4.57E-06	3.71E-11	0.001797	1.77E-05	1.03E-06	4354	1701	6149	4368	3745
PLAAT3	0.786522	0.742778	0.766891	0.687211	0.759425	4.58E-06	1.15E-05	1.19E-05	1.38E-05	5.77E-05	4355	4067	4639	4295	4755
OGN	-0.89683	-0.57086	-0.76172	-0.76734	-0.74816	4.68E-06	0.006374	0.00017	0.000738	3.14E-05	4358	6211	5376	5520	4578
NPAP1L	-0.59958	-0.41544	-0.80123	-0.57167	-0.46348	4.73E-06	6.95E-05	6.07E-09	8.83E-06	0.000213	4359	4584	2917	4166	5137
HNRNPD	0.939685	0.554698	0.94023	0.672035	1.048949	4.75E-06	0.001513	4.79E-05	4.06E-05	7.01E-08	4362	5657	5027	4609	3198
ATP2A2	0.696411	0.44972	0.748847	0.694844	0.432685	4.80E-06	0.000929	5.66E-06	6.95E-06	0.001906	4365	5483	4433	4107	5892
EIF2S3	0.777562	0.849179	0.619094	0.982648	0.640337	4.85E-06	1.08E-06	3.35E-06	3.34E-08	2.75E-05	4366	3502	4302	2977	4539
S100A9	-1.21852	-1.29283	-1.51022	-1.09678	-1.32515	4.89E-06	2.86E-07	8.48E-09	7.10E-05	1.52E-06	4368	3208	2984	4772	3823
KDM7A	0.59214	0.738028	0.877515	0.922647	0.965976	4.89E-06	1.13E-08	1.14E-10	9.77E-13	4.95E-08	4369	2573	2233	1498	3122
SAP18	0.954145	0.734201	1.010766	0.760243	1.229933	4.90E-06	0.00035	3.55E-06	5.39E-05	5.37E-09	4370	5122	4317	4692	2697
MRPS22	0.718215	0.867084	1.02658	0.89324	0.725421	4.91E-06	1.68E-08	1.10E-10	7.55E-07	7.92E-06	4371	2635	2229	3580	4220
FAM83B	0.708437	0.831346	0.971556	0.938964	0.887382	4.94E-06	7.15E-07	2.34E-07	6.37E-08	3.68E-08	4372	3395	3693	3078	3058
RANGAP1	0.857011	0.85589	1.090745	1.068814	0.871073	4.97E-06	6.38E-05	4.59E-06	1.11E-06	9.17E-06	4373	4552	4384	3665	4259
NEDD4L	0.645034	0.449716	0.584978	0.629318	0.624921	5.11E-06	8.27E-05	2.44E-05	1.50E-05	2.42E-05	4374	4641	4837	4325	4511
RAB2A	0.667488	0.734361	1.204168	0.987406	0.939015	5.17E-06	1.61E-09	5.11E-11	1.67E-08	2.07E-06	4375	2241	2111	2857	3883
AHNAK	-1.42643	-1.26664	-0.79507	-1.21662	-1.64586	5.20E-06	5.11E-08	0.001169	1.73E-06	2.18E-09	4377	2850	6005	3762	2556
CSTA	-1.16008	-0.73265	-1.0077	-0.91072	-1.19273	5.29E-06	0.007048	0.000506	0.001549	4.23E-07	4379	6258	5750	5787	3574
ADARB1	-0.7244	-0.51843	-0.76454	-0.81423	-0.71088	5.30E-06	0.000127	2.39E-07	8.37E-08	2.23E-07	4380	4761	3697	3137	3440
ACOT11	0.564838	0.624482	0.778476	0.510617	0.939662	5.37E-06	1.19E-05	3.41E-08	0.000144	1.27E-10	4383	4079	3296	4966	2082
TNFAIP1	-0.75899	-0.81594	-0.60099	-0.76946	-0.78721	5.40E-06	1.08E-06	1.03E-05	6.05E-06	4.47E-08	4384	3499	4598	4064	3104
ATF5	-0.82406	-0.69177	-0.79883	-1.11227	-1.04776	5.41E-06	0.000192	3.41E-05	3.19E-08	3.62E-07	4385	4903	4932	2960	3549
MBD3L2	-0.7363	-0.63362	-1.33115	-0.77417	-1.18536	5.46E-06	8.02E-05	2.74E-10	0.00026	6.27E-10	4386	4631	2371	5157	2345
NR1H2	-0.61687	-0.57678	-0.63795	-0.5771	-0.60253	5.50E-06	2.23E-05	1.49E-06	2.59E-05	1.26E-05	4388	4245	4125	4462	4352
FUBP3	0.480329	0.624414	0.752662	0.59291	0.354104	5.50E-06	3.06E-05	1.52E-08	5.39E-05	0.001549	4389	4340	3104	4693	5806
PSMG1	0.816859	1.49975	1.418801	1.247838	0.812159	5.54E-06	6.60E-11	1.47E-14	1.87E-09	0.000192	4391	1777	1142	2506	5113
VPS9D1	-0.69835	-0.7352	-0.91716	-0.55171	-0.79676	5.56E-06	6.43E-07	4.14E-09	0.000126	7.73E-07	4392	3367	2845	4924	3685
PRKCA	0.61849	0.565883	1.081343	0.983591	0.758573	5.59E-06	1.51E-05	6.45E-10	8.31E-09	1.09E-07	4393	4136	2506	2725	3296
FAM169A	-0.55856	-0.33254	-0.46044	-0.43092	-0.51696	5.63E-06	0.002538	5.87E-05	0.000509	5.99E-05	4394	5847	5074	5384	4763
NDUFAF1	-0.59314	-0.64154	-0.86865	-0.82235	-0.87895	5.64E-06	1.38E-06	7.30E-07	1.08E-07	8.55E-08	4395	3560	3958	3181	3244
NIPSNAP1	0.872093	0.801864	1.277933	0.750352	0.838247	5.69E-06	6.86E-06	3.26E-10	7.95E-06	2.16E-05	4396	3932	2401	4141	4484
C4orf48	0.738509	0.834897	1.1286	0.740055	1.053738	5.69E-06	1.44E-06	2.81E-10	2.60E-06	7.52E-11	4397	3572	2375	3863	2012
RFK	0.753111	0.741256	0.935633	0.554989	0.647668	5.69E-06	3.18E-05	2.48E-07	0.000519	0.00014	4398	4345	3704	5392	5030
PSMD10	0.505831	0.522537	0.445255	0.598519	0.675238	5.71E-06	7.50E-06	0.000207	0.000133	3.60E-06	4399	3955	5452	4946	4031
SLIT1	-0.71699	-0.4437	-0.76159	-0.61089	-0.63065	5.73E-06	0.000477	9.14E-10	2.99E-05	1.19E-05	4401	5232	2564	4519	4329
CD82	-1.23036	-1.3842	-1.21598	-1.49105	-1.2058	5.74E-06	1.70E-07	7.07E-07	3.02E-09	4.33E-06	4402	3115	3947	2587	4069
TSPAN15	0.838846	0.761744	0.983618	0.827808	1.085195	5.79E-06	2.29E-05	2.61E-07	1.80E-05	6.48E-06	4403	4253	3718	4371	4166
SESN3	-0.96354	-0.67266	-1.04862	-0.80834	-0.60279	5.82E-06	0.000105	8.86E-08	6.42E-06	0.009144	4404	4712	3484	4082	6515
PINK1	-0.72309	-0.75559	-0.4494	-0.76563	-0.54701	5.83E-06	1.77E-06	0.00269	2.24E-07	2.46E-05	4405	3625	6326	3316	4514
MKRN1	0.51238	0.73313	0.946525	0.960757	0.97059	5.87E-06	5.39E-09	8.19E-09	2.10E-09	9.80E-11	4406	2453	2976	2521	2048
THOC7	0.782501	0.811783	0.772122	0.738292	0.943767	5.95E-06	8.67E-05	1.08E-05	3.79E-05	4.27E-08	4409	4655	4619	4594	3094
SEC24A	-0.5964	-0.63405	-0.4852	-0.69104	-1.30662	5.96E-06	8.51E-06	5.33E-05	3.36E-05	7.23E-10	4410	3990	5052	4558	2374
CDC42EP4	0.964826	0.995455	1.519005	1.119405	1.150268	6.00E-06	9.75E-08	4.43E-19	7.77E-09	3.82E-10	4411	2987	457	2717	2257
HNF4G	1.086204	1.117097	1.566287	1.291103	1.553429	6.01E-06	8.76E-07	7.60E-09	4.27E-07	8.51E-11	4412	3444	2954	3459	2025
PTGIS	-0.82484	-0.8688	-1.29837	-1.15995	-1.25766	6.02E-06	1.07E-05	2.71E-11	6.37E-07	4.91E-09	4413	4045	2027	3553	2679
FNDC3B	-0.84524	-0.93399	-1.19004	-0.86863	-1.17153	6.02E-06	1.80E-07	1.40E-12	3.93E-07	6.67E-11	4414	3130	1628	3441	1994
ACOT7	0.56602	0.29613	0.488578	0.423761	0.493598	6.04E-06	0.006594	2.47E-06	0.000212	9.35E-06	4415	6224	4234	5084	4262
EMC8	0.801178	1.074016	0.765223	1.018762	1.021779	6.05E-06	2.44E-08	2.59E-06	5.85E-08	1.62E-10	4416	2698	4242	3059	2119
SIRT2	-0.59871	-0.55213	-0.71169	-0.55633	-0.64743	6.10E-06	6.54E-06	6.78E-09	7.64E-06	1.77E-08	4418	3924	2931	4132	2921
NTHL1	0.81461	0.955216	0.439758	0.762061	0.500831	6.10E-06	3.39E-08	0.008385	8.14E-06	0.004063	4419	2761	6786	4152	6133
VKORC1L1	0.846696	0.796209	0.513594	0.822161	0.715753	6.15E-06	6.36E-06	0.000353	1.59E-05	1.31E-05	4420	3917	5622	4344	4365
ADCY6	0.536343	0.616592	0.632271	0.696899	0.659027	6.18E-06	7.48E-06	6.09E-08	5.25E-07	4.65E-07	4422	3953	3410	3504	3590
FRK	0.620099	0.811945	1.435048	0.805007	0.657519	6.21E-06	1.36E-09	2.26E-10	3.06E-05	0.001749	4423	2222	2342	4526	5855
ZNF254	-0.84364	-0.35352	-0.7474	-0.42557	-0.50145	6.22E-06	0.003851	2.28E-08	0.00328	0.000101	4424	6002	3187	6082	4937
HGS	0.759234	0.600961	0.975706	0.602275	0.702314	6.24E-06	1.88E-05	2.10E-09	7.46E-05	1.51E-08	4425	4198	2713	4789	2893
GPR89A	0.7813	0.995766	0.507197	0.928108	0.569022	6.26E-06	1.21E-07	0.001625	4.18E-07	0.002821	4426	3041	6109	3452	6025
H3C12	1.051698	0.779469	1.379418	1.009162	1.580267	6.29E-06	0.001893	1.13E-07	5.57E-05	7.91E-09	4427	5736	3550	4709	2763
RNF44	0.848851	0.735657	0.665846	0.869678	0.776463	6.29E-06	7.55E-07	0.00026	1.01E-07	1.18E-06	4428	3407	5528	3167	3772
ACTR1A	-0.74631	-0.95232	-0.57501	-0.80903	-0.64386	6.43E-06	4.13E-09	0.000259	5.45E-06	4.56E-05	4429	2405	5527	4042	4698
MARK2	0.764422	0.63115	0.823103	0.811679	0.539762	6.45E-06	0.000191	7.99E-06	3.91E-06	0.000886	4430	4900	4531	3963	5592
DUSP14	-0.61533	-0.70925	-0.53788	-0.8303	-0.87968	6.45E-06	1.55E-06	0.000275	5.62E-09	8.40E-10	4431	3592	5548	2672	2400
ACAA2	0.980225	0.841502	1.241716	0.833211	0.829274	6.51E-06	1.99E-06	7.96E-10	3.98E-06	9.90E-05	4433	3654	2546	3968	4925
CLTB	1.149812	0.764768	1.239872	0.691329	0.897271	6.52E-06	4.90E-05	9.20E-07	0.000253	1.20E-05	4434	4480	4014	5150	4332
APEH	0.725335	0.728243	1.04231	0.719931	1.086255	6.52E-06	2.25E-08	5.86E-12	7.40E-08	7.51E-16	4435	2687	1805	3102	781
CFD	-0.82669	-0.49786	-0.8154	-0.72912	-1.10933	6.55E-06	0.00372	9.80E-08	0.000233	4.84E-12	4437	5987	3511	5113	1670
SGPL1	-0.6864	-0.98046	-0.64373	-0.97552	-0.55566	6.58E-06	6.36E-07	0.000351	1.36E-07	0.000264	4439	3365	5619	3221	5202
DDX4	-1.15982	-1.38007	-1.28446	-1.21367	-1.16873	6.61E-06	6.18E-06	2.32E-05	3.69E-07	5.73E-06	4440	3912	4820	3424	4142
KLHL42	-0.51308	-0.52994	-0.78787	-0.68197	-0.41529	6.62E-06	0.006822	6.21E-07	0.000777	0.002538	4441	6243	3923	5537	5985
PRMT2	-0.65611	-0.55969	-0.37992	-0.61752	-0.55764	6.66E-06	6.95E-06	0.005281	2.09E-06	3.40E-06	4443	3935	6600	3814	4007
ARHGAP1	0.668533	0.612582	0.872735	0.85166	0.683302	6.73E-06	1.20E-05	2.67E-07	2.33E-08	4.44E-06	4444	4080	3721	2912	4072
BIRC5	0.545778	0.599252	0.753313	0.562088	0.569048	6.82E-06	1.13E-05	2.61E-10	4.96E-05	1.58E-06	4445	4059	2360	4674	3835
SCAMP2	0.67642	0.725315	0.875362	0.710346	0.575365	6.93E-06	1.38E-07	1.65E-08	1.57E-09	1.47E-05	4446	3067	3123	2479	4395
NELFE	0.675338	0.584301	0.670566	0.473335	0.55599	6.94E-06	0.000637	1.10E-07	0.00197	0.000177	4447	5341	3536	5879	5092

KAT2A	0.752775	0.820343	0.574518	0.844334	0.786326	7.00E-06	8.75E-07	0.000132	1.27E-07	4.53E-07	4449	3443	5292	3207	3584
CBS	-1.105	-0.81826	-1.03181	-1.05855	-1.08323	7.09E-06	1.66E-07	4.37E-09	3.53E-08	2.28E-10	4453	3105	2853	2981	2187
MTIF3	0.779116	0.617201	0.62582	0.605161	1.026229	7.15E-06	0.000104	6.02E-05	4.26E-05	6.26E-09	4454	4709	5083	4623	2718
ARPC3	0.632389	0.579034	0.777009	0.811823	0.528417	7.17E-06	1.10E-05	6.28E-08	3.74E-07	0.003054	4456	4052	3418	3428	6041
YWHAQ	0.699319	0.56561	0.786132	0.722937	0.955545	7.24E-06	0.000128	1.11E-06	1.28E-05	4.29E-10	4458	4762	4062	4273	2283
ELAVL1	0.538264	0.686809	0.835982	0.843058	0.754265	7.25E-06	6.88E-08	2.94E-08	1.86E-09	5.92E-09	4459	2904	3253	2505	2714
COPS6	0.780606	0.585923	0.733382	0.658709	0.808825	7.27E-06	0.000101	4.63E-05	5.11E-06	3.82E-05	4460	4702	5016	4027	4644
ANXA8	-0.62486	-0.35961	-0.83683	-0.65563	-0.91622	7.27E-06	0.004963	1.21E-05	0.000403	1.39E-05	4461	6093	4646	5311	4376
EIF4E	0.628425	0.381198	0.861389	0.520985	0.545129	7.28E-06	0.003927	3.98E-06	0.002091	0.002102	4462	6008	4343	5898	5929
PODXL2	0.851131	1.095079	0.934344	1.052214	1.134731	7.28E-06	2.27E-06	1.67E-05	6.64E-08	5.92E-08	4463	3692	4736	3085	3158
ETS2	1.268348	1.53734	1.781282	1.570135	1.468004	7.32E-06	6.06E-08	2.32E-13	2.36E-07	4.26E-08	4465	2882	1433	3326	3093
EPS8L1	1.126167	1.492053	1.885428	1.496459	1.685708	7.40E-06	2.98E-06	2.70E-09	4.36E-07	3.37E-11	4467	3759	2765	3467	1910
ACO2	0.67447	0.556856	0.815186	0.701766	0.410665	7.42E-06	8.47E-05	2.70E-08	8.86E-06	0.003651	4468	4648	3231	4168	6091
CRIP1	-1.78506	-1.98213	-1.35854	-2.28145	-1.66601	7.42E-06	5.73E-07	0.000149	7.59E-08	7.97E-07	4469	3343	5331	3107	3692
CTC1	-0.66966	-0.61274	-0.95819	-0.68876	-0.53981	7.45E-06	3.29E-05	1.84E-11	4.40E-05	0.003109	4470	4358	1959	4637	6047
XKR6	-0.8066	-0.40188	-0.76201	-0.5095	-0.62363	7.53E-06	0.005132	2.17E-05	0.000889	0.000395	4471	6112	4796	5579	5318
RABAC1	-0.91208	-0.92304	-0.60944	-0.87428	-0.93902	7.61E-06	1.10E-07	0.000375	3.85E-06	2.28E-07	4472	3015	5648	3956	3449
TNFRSF1A	-0.70658	-0.82593	-0.51569	-0.57412	-0.65763	7.63E-06	1.11E-07	8.41E-05	0.00022	5.16E-06	4474	3018	5178	5097	4116
HBA2	-1.45622	-1.06511	-1.2661	-1.04159	-1.74754	7.72E-06	0.000441	0.000265	0.000152	1.54E-11	4475	5207	5538	4978	1819
HEATR1	0.621445	0.749554	0.306571	0.649757	0.578976	7.86E-06	4.30E-08	0.008637	2.48E-05	0.00265	4476	2818	6796	4449	5996
MARK4	-0.68694	-0.32883	-0.67959	-0.4761	-0.76523	7.90E-06	0.009477	2.69E-07	0.000225	1.10E-08	4477	6404	3723	5106	2836
CLPTM1L	0.645967	0.771158	0.581103	0.811021	0.629414	7.91E-06	1.93E-05	0.000493	5.88E-05	0.000684	4478	4203	5739	4723	5513
MAP3K5	-0.91916	-0.7735	-0.67982	-0.79377	-0.79201	7.97E-06	9.50E-05	0.000149	1.63E-05	8.86E-05	4479	4684	5330	4348	4888
FRAT2	0.805541	1.147186	1.198855	1.052609	1.052389	8.00E-06	4.29E-07	5.28E-09	3.70E-07	3.08E-08	4480	3281	2884	3425	3026
MPRIP	-0.93301	-1.17678	-0.72078	-1.17296	-0.60961	8.16E-06	1.65E-08	0.000253	6.77E-09	0.000364	4482	2634	5518	2699	5290
FNTB	-0.72673	-0.73053	-0.84314	-0.82876	-1.07439	8.17E-06	3.28E-05	2.74E-10	2.94E-06	1.63E-10	4483	4357	2370	3899	2120
RALA	0.50347	0.593886	0.665484	0.533919	0.544356	8.19E-06	1.97E-05	1.12E-06	0.000119	1.16E-05	4484	4208	4065	4910	4322
GPATCH4	0.564551	0.618959	0.48446	0.543776	0.53738	8.28E-06	1.05E-06	0.000208	3.21E-06	0.000371	4485	3492	5454	3908	5298
CHMP2B	0.566325	0.648226	0.90697	0.59794	0.659645	8.45E-06	0.000184	1.11E-08	0.000131	0.00036	4487	4882	3034	4942	5287
ATP1B3	0.739915	0.877644	1.1435	0.783058	1.108836	8.45E-06	4.59E-06	2.29E-11	3.64E-06	1.29E-09	4488	3843	1999	3935	2466
TXNDC11	-0.74867	-0.71658	-1.12928	-0.86565	-1.30325	8.54E-06	5.10E-05	5.18E-08	0.000146	2.12E-10	4490	4490	3383	4970	2171
UBE2D2	0.588347	0.507202	0.502908	0.456094	0.476398	8.56E-06	2.64E-06	2.98E-06	6.84E-05	0.000377	4491	3721	4279	4763	5300
HMGCL	-0.63746	-0.74773	-0.42264	-0.69684	-0.37382	8.58E-06	0.000134	0.009846	0.00041	0.008447	4492	4778	6840	5316	6471
RER1	0.71238	0.56475	1.054431	0.774087	0.748644	8.59E-06	3.10E-05	2.13E-10	2.72E-06	1.20E-07	4493	4342	2336	3870	3317
RRM2B	-0.56766	-0.4231	-0.44421	-0.35008	-0.35572	8.61E-06	0.000525	0.000155	0.00729	0.002786	4494	5270	5341	6396	6016
SNF8	0.521197	0.492861	0.491213	0.502613	0.417853	8.61E-06	4.82E-06	1.04E-05	2.74E-06	3.44E-05	4495	3854	4601	3873	4606
RAB15	0.869772	1.040999	1.237214	1.285708	1.545133	8.61E-06	5.78E-08	4.24E-07	4.44E-09	2.50E-13	4496	2872	3834	2644	1318
PIP4K2C	0.575284	0.552991	0.390274	0.543652	0.421661	8.65E-06	0.00017	0.001212	0.000178	0.001966	4497	4860	6019	5024	5906
SRPRB	0.696601	0.74583	0.495156	0.739642	0.622753	8.67E-06	5.72E-05	0.004158	6.48E-06	0.001262	4498	4522	6491	4085	5722
RPL4	1.214412	1.059969	1.022329	1.304164	1.265042	8.67E-06	8.55E-06	0.000426	2.57E-09	5.19E-07	4499	3993	5688	2557	3609
MROH6	1.212633	1.380114	1.809799	1.221086	0.768632	8.68E-06	4.19E-06	1.22E-13	8.75E-05	0.000689	4500	3827	1344	4821	5516
ATAT1	0.468836	0.562325	0.495299	0.678501	0.466555	8.68E-06	6.99E-07	0.000201	9.12E-10	0.000112	4501	3390	5441	2381	4967
HBA1	-0.93272	-0.61609	-1.27626	-0.79795	-1.05836	8.69E-06	0.000799	7.96E-07	6.44E-05	3.07E-08	4502	5416	3980	4749	3025
FAM114A:	-1.46893	-1.49663	-1.11104	-1.24656	-1.88311	8.70E-06	9.27E-06	0.000378	1.75E-05	2.36E-08	4503	4014	5651	4363	2971
LBHD1	0.582704	0.585487	0.836183	0.80037	0.587028	8.71E-06	0.000411	3.31E-06	9.15E-06	2.03E-05	4504	5183	4300	4184	4469
DPM2	0.469412	0.471158	0.512321	0.537297	0.473619	8.74E-06	4.64E-06	6.78E-06	8.01E-06	1.19E-05	4507	3848	4483	4146	4326
GPHN	0.929364	1.533151	1.120545	1.396856	1.408049	8.85E-06	3.31E-09	3.49E-09	1.46E-08	9.84E-10	4509	2370	2819	2826	2424
FHOD1	-0.7231	-0.68612	-1.06121	-0.68552	-1.05673	9.03E-06	3.97E-05	1.62E-08	0.000158	9.80E-11	4511	4411	3118	4988	2050
EPHX2	0.829306	1.443778	1.028045	1.384344	1.05608	9.11E-06	1.60E-09	5.63E-06	5.39E-09	1.54E-07	4513	2240	4431	2668	3357
WWC1	0.807265	0.942135	0.916275	0.674692	1.085591	9.19E-06	1.25E-07	4.18E-07	2.38E-05	8.86E-11	4514	3046	3830	4433	2031
CARMIL1	0.987235	0.839452	1.324692	1.143143	0.920717	9.21E-06	0.002586	3.04E-06	8.21E-05	2.07E-05	4516	5853	4287	4806	4475
GSK3B	0.559403	0.629456	0.552385	0.617778	0.751658	9.43E-06	2.87E-05	3.73E-05	1.44E-05	5.72E-08	4519	4319	4949	4314	3153
MSLN	1.563672	1.40369	1.515437	1.204193	1.83788	9.43E-06	2.17E-05	8.60E-06	5.57E-05	2.27E-07	4520	4237	4549	4710	3447
BAZ1B	0.532281	0.569646	0.547375	0.738173	0.728347	9.49E-06	3.21E-06	4.31E-07	1.48E-08	1.00E-07	4521	3774	3836	2828	3275
MAPKBP1	-0.44713	-0.35618	-0.62356	-0.52789	-0.38652	9.51E-06	0.001842	2.90E-08	0.000115	0.001757	4522	5727	3248	4896	5856
AMIGO2	-0.76972	-0.59508	-0.77486	-0.56527	-0.69284	9.55E-06	0.001967	1.13E-06	0.002047	6.11E-05	4523	5758	4070	5889	4776
MSMO1	0.972889	0.777188	1.428002	0.988448	0.954826	9.61E-06	0.000387	3.75E-10	4.39E-05	3.91E-05	4524	5160	2421	4636	4654
ZNF816	-0.60075	-0.45937	-0.74046	-0.61085	-0.57981	9.67E-06	2.62E-05	1.39E-08	5.76E-07	3.69E-07	4525	4289	3090	3526	3552
ANXA11	0.761078	0.435331	1.03381	0.658478	1.018754	9.67E-06	0.001101	5.68E-08	0.000487	1.76E-08	4526	5545	3397	5364	2919
PCID2	0.724505	0.979218	0.711752	0.914121	1.01905	9.74E-06	1.99E-08	3.10E-06	2.99E-09	1.29E-09	4529	2661	4289	2584	2467
VCPKMT	-0.56167	-0.35874	-0.28435	-0.51685	-0.79609	9.84E-06	0.001143	0.001669	5.09E-05	4.54E-06	4533	5562	6113	4680	4075
PDCD6IP	0.73528	0.818621	1.339142	0.737641	0.878512	9.98E-06	2.06E-07	3.02E-15	8.20E-07	4.00E-09	4537	3158	1022	3598	2652
CDC27	-0.44405	-0.55123	-0.47718	-0.54509	-0.6633	9.99E-06	2.15E-06	1.00E-05	9.52E-06	1.31E-09	4539	3678	4593	4198	2471
RASA4	-0.67828	-0.67377	-1.09741	-0.83429	-0.70833	1.00E-05	8.73E-06	3.99E-12	1.43E-06	9.70E-05	4541	3999	1752	3717	4920
RHBDD1	-0.51765	-0.43012	-0.60559	-0.5692	-0.70997	1.00E-05	0.000491	1.30E-08	2.77E-05	1.02E-08	4542	5245	3076	4487	2820
AMH	-0.55396	-0.36501	-0.68226	-0.56148	-0.44495	1.01E-05	0.004078	6.55E-07	0.000616	0.002572	4543	6028	3930	5447	5990
GLT8D1	-0.60753	-0.38789	-0.70676	-0.43481	-0.91313	1.03E-05	0.00286	5.63E-09	0.001582	1.11E-09	4547	5877	2899	5797	2445
SRGAP1	-0.76398	-1.03205	-0.78412	-0.91865	-0.68325	1.04E-05	8.45E-08	1.52E-06	5.47E-06	8.12E-06	4551	2957	4130	4045	4225
FLNB	1.061071	1.098533	1.720187	1.267489	1.340356	1.04E-05	4.56E-08	2.64E-12	1.11E-07	8.33E-12	4552	2828	1703	3188	1736
TFAM	0.538752	0.718808	0.63362	0.656842	0.713942	1.04E-05	3.01E-07	1.17E-06	1.64E-07	8.05E-09	4553	3220	4079	3259	2768
MED4	0.57547	0.882302	0.885868	0.723524	1.025485	1.05E-05	3.31E-10	3.60E-09	2.79E-09	2.96E-10	4555	2010	2822	2570	2225
TM7SF3	0.815302	0.753836	0.289777	0.750813	0.461029	1.05E-05	0.000261	0.005035	0.000157	0.004674	4556	5008	6572	4987	6192
ZFX	-0.4169	-0.31361	-0.50327	-0.33052	-0.69641	1.05E-05	0.002962	3.45E-07	0.002127	5.21E-09	4557	5892	3787	5907	2689

PRPF4B	0.585087	0.417308	0.536266	0.469157	0.430632	1.09E-05	0.001724	0.000113	0.001217	0.000419	4559	5699	5258	5700	5348
AURKAIP1	0.478518	0.930476	0.551445	0.702126	0.735	1.09E-05	2.63E-10	5.74E-06	4.98E-07	2.72E-08	4560	1965	4439	3492	2999
NSA2	0.565296	0.770804	0.626681	0.744819	0.641384	1.10E-05	1.28E-06	4.25E-05	5.75E-08	8.78E-06	4563	3541	4990	3055	4250
CHIT1	-0.84243	-0.70728	-0.674	-0.82405	-0.89505	1.12E-05	0.000846	0.001726	0.0002	1.84E-05	4566	5444	6127	5061	4446
KLF13	-0.80501	-0.58639	-0.77961	-0.78033	-0.67944	1.12E-05	3.99E-07	3.85E-05	9.33E-07	0.000127	4567	3274	4957	3620	5001
HOXD11	-0.80618	-0.62102	-1.54047	-1.16273	-1.29909	1.13E-05	0.003376	3.99E-05	0.000448	2.95E-05	4569	5944	4970	5336	4558
TEX264	0.379012	0.414734	0.447346	0.537964	0.434102	1.13E-05	1.98E-06	2.45E-05	1.01E-06	1.81E-05	4570	3652	4839	3645	4443
ENTPD2	0.979207	1.314037	1.301775	1.090572	1.181903	1.14E-05	2.20E-07	5.18E-12	1.10E-05	2.93E-07	4571	3167	1787	4243	3497
ABCF2	0.720907	1.02143	0.700439	0.958022	0.691109	1.14E-05	7.20E-10	9.94E-07	2.52E-10	6.77E-07	4572	2115	4034	2179	3661
SOAT1	-1.08559	-1.3094	-1.35472	-1.34894	-1.63862	1.14E-05	1.93E-07	7.93E-10	3.10E-07	6.60E-14	4575	3140	2544	3386	1185
APMAP	0.510065	0.681829	0.600195	0.751035	0.462843	1.15E-05	4.69E-05	2.92E-06	3.72E-06	1.68E-05	4576	4461	4271	3942	4426
MFF	0.41163	0.608484	0.620467	0.671377	0.448531	1.15E-05	7.13E-06	2.62E-06	4.92E-07	0.000139	4577	3941	4246	3489	5025
CCL20	1.343517	1.677845	1.798575	1.760878	1.476851	1.16E-05	1.29E-07	9.50E-06	2.59E-06	1.74E-06	4579	3053	4574	3860	3852
APOC2	-1.06417	-1.64961	-1.23836	-1.41104	-0.83563	1.17E-05	3.21E-10	3.68E-09	2.65E-08	0.001514	4580	2004	2828	2930	5802
COG2	0.551846	0.742561	0.479022	0.583936	0.559683	1.20E-05	3.10E-08	3.58E-05	1.12E-05	4.73E-05	4583	2739	4942	4247	4705
STUM	-0.66113	-0.60057	-0.62339	-0.982	-0.82875	1.20E-05	0.000314	0.000758	1.45E-09	3.31E-06	4584	5077	5871	2467	4000
ERG28	1.04559	1.230351	1.617381	1.228039	0.885209	1.20E-05	2.97E-05	6.18E-10	9.71E-05	0.000203	4585	4329	2500	4850	5128
GPT2	0.891467	1.001501	0.593705	1.080088	0.616753	1.21E-05	1.32E-08	0.000191	1.60E-08	0.000143	4587	2604	5424	2846	5031
HIGD2A	0.503452	0.741687	0.495445	0.649575	0.649673	1.21E-05	4.81E-09	4.57E-05	3.65E-09	2.66E-07	4588	2430	5010	2618	3477
COX11	0.442488	0.512167	0.515323	0.43143	0.510865	1.23E-05	5.58E-06	0.000111	4.08E-05	6.86E-06	4591	3888	5253	4612	4177
RHPN1	0.883182	1.366244	0.851372	1.223593	0.953598	1.23E-05	2.92E-09	4.82E-06	3.58E-09	2.63E-06	4592	2340	4402	2613	3944
CUTA	0.870395	0.54731	0.682803	0.678621	0.909354	1.24E-05	0.006524	9.79E-05	0.00078	6.00E-06	4593	6220	5215	5539	4150
ARMCX6	-0.48757	-0.55985	-0.8919	-0.59763	-0.90019	1.24E-05	1.18E-05	4.58E-08	0.000303	8.02E-10	4595	4076	3357	5224	2389
TRRAP	0.451874	0.601991	0.557088	0.732475	0.460856	1.25E-05	1.46E-05	1.33E-05	3.98E-08	0.000367	4596	4126	4674	3005	5293
TMX2	0.677878	0.833121	0.742938	0.844556	0.44978	1.25E-05	5.44E-07	4.55E-09	1.60E-07	0.000672	4598	3327	2859	3256	5508
MPZL1	0.530272	0.452993	0.517406	0.646394	0.41727	1.27E-05	0.00053	0.000182	2.24E-07	0.00162	4601	5281	5407	3318	5822
RRS1	0.884252	0.981705	1.477771	0.790823	1.107168	1.27E-05	3.94E-05	2.88E-11	0.000735	5.36E-07	4602	4409	2041	5518	3614
NT5C3A	0.674922	0.727321	0.810387	0.614341	0.78662	1.28E-05	7.35E-07	2.03E-07	2.60E-06	1.77E-07	4603	3401	3661	3864	3388
CORO2A	1.087391	0.801004	1.437696	1.076895	1.548552	1.28E-05	2.08E-05	1.47E-10	1.91E-06	9.47E-13	4604	4223	2277	3795	1476
INF2	0.866109	0.934746	1.317999	0.885003	1.083482	1.28E-05	9.02E-11	7.09E-13	1.14E-08	8.47E-11	4605	1814	1555	2785	2023
YIPF5	-0.54319	-0.63345	-0.53201	-0.81396	-0.74402	1.29E-05	5.98E-06	1.18E-05	3.29E-08	3.31E-09	4606	3904	4634	2971	2619
PPP1R12A	-0.55124	-0.69572	-0.65425	-0.6252	-0.79941	1.30E-05	1.41E-07	3.83E-09	4.54E-06	3.08E-12	4607	3071	2836	3996	1617
CDCA7L	0.725787	0.942538	0.762872	0.750615	0.922872	1.31E-05	1.45E-07	3.29E-07	2.77E-05	7.50E-09	4608	3078	3778	4485	2753
MELK	0.724705	0.730133	0.790334	0.75644	0.622449	1.31E-05	7.23E-06	1.81E-09	1.05E-05	1.56E-06	4609	3944	2677	4226	3832
CDK9	-0.41271	-0.39038	-0.61618	-0.54054	-0.47442	1.31E-05	6.52E-05	9.56E-11	6.48E-07	4.29E-07	4610	4560	2211	3558	3578
SAT2	-0.65952	-0.61254	-0.45725	-0.81902	-0.53239	1.32E-05	1.52E-05	0.001386	3.01E-07	0.000589	4614	4137	6069	3372	5455
PPT1	-0.9793	-1.12965	-1.24628	-1.16742	-1.06007	1.35E-05	2.00E-06	1.88E-07	9.86E-08	2.96E-07	4619	3658	3646	3164	3500
IL13RA1	0.589835	0.414271	0.624593	0.827675	0.442733	1.36E-05	0.00184	2.96E-05	5.25E-06	0.002382	4620	5725	4885	4036	5969
GOLGA1	-0.60777	-0.66352	-0.48912	-0.74346	-0.42817	1.40E-05	9.39E-06	0.00033	1.53E-06	2.16E-05	4623	4016	5597	3740	4487
MELTF	0.848183	0.975969	1.191594	1.193072	1.191017	1.41E-05	5.19E-06	4.08E-06	4.54E-06	2.95E-07	4625	3868	4351	3995	3498
KTN1	0.622015	0.639458	0.794999	0.705242	0.799196	1.41E-05	1.74E-05	1.16E-07	8.57E-06	9.97E-08	4629	4172	3556	4163	3272
CYP2J2	0.500822	0.587106	0.548197	0.490516	0.29501	1.43E-05	1.75E-07	2.79E-08	2.50E-05	0.002179	4630	3124	3240	4454	5945
CAMK1D	-0.83649	-0.48406	-0.70815	-0.61926	-0.61533	1.44E-05	0.008797	1.51E-05	0.000261	0.000103	4631	6374	4705	5159	4944
TIMELESS	0.662267	0.768983	0.98536	0.785406	0.633095	1.44E-05	3.97E-05	3.82E-08	2.07E-05	5.29E-06	4632	4412	3319	4404	4124
DIAPH3	0.670771	0.657531	0.539545	0.562882	0.821272	1.49E-05	1.73E-05	8.73E-05	8.35E-05	5.22E-07	4636	4171	5190	4812	3610
HGD	0.561839	0.640847	0.485992	0.608977	0.406151	1.50E-05	7.50E-07	1.58E-05	1.34E-05	0.000106	4638	3405	4719	4284	4955
SKP1	0.487672	0.525605	0.752749	0.598304	0.509901	1.52E-05	1.00E-06	3.29E-08	1.45E-06	0.003779	4641	3478	3284	3721	6103
SMAD4	-0.7095	-1.01165	-0.65222	-0.84797	-0.63494	1.52E-05	7.79E-10	0.000325	8.59E-07	4.61E-06	4642	2134	5593	3604	4077
ADIPOR2	0.865708	0.763123	1.148068	1.013762	0.842696	1.52E-05	7.52E-05	7.77E-11	4.26E-06	1.32E-08	4643	4608	2168	3981	2871
DDX54	0.728586	1.039232	0.994641	1.209646	0.729312	1.53E-05	1.07E-08	3.93E-06	6.03E-13	3.02E-05	4644	2564	4338	1443	4571
GNPDA1	0.677766	0.753988	0.574829	0.733818	0.468673	1.53E-05	3.69E-06	2.60E-05	3.34E-07	0.002499	4645	3805	4854	3404	5981
SERPINE2	-1.30083	-1.01937	-1.35272	-0.97985	-1.5986	1.53E-05	1.73E-06	2.95E-07	2.72E-06	3.49E-08	4646	3619	3745	3871	3046
KIF18B	0.744027	0.885199	0.780311	0.6954	0.576482	1.55E-05	4.96E-09	2.34E-07	1.18E-05	0.000217	4649	2438	3694	4259	5140
APBB2	-0.62505	-0.49538	-0.85156	-0.82382	-0.77058	1.57E-05	0.001255	5.63E-06	2.81E-05	2.28E-06	4650	5600	4432	4495	3919
CEP43	0.548439	0.467607	0.627968	0.461508	0.746125	1.57E-05	0.000617	8.01E-06	0.002302	1.35E-07	4652	5334	4532	5938	3335
PCYT2	0.742502	0.611908	0.755365	0.527663	0.663759	1.58E-05	2.77E-06	1.64E-08	9.18E-05	1.51E-06	4654	3737	3120	4831	3820
USP10	0.645708	0.765255	0.677987	0.784516	0.836961	1.58E-05	4.46E-07	2.46E-06	7.98E-08	2.28E-08	4655	3290	4232	3121	2966
KIFC2	0.730995	1.383379	1.424528	1.455924	1.118253	1.58E-05	1.37E-08	2.84E-10	1.41E-09	4.02E-11	4656	2611	2376	2459	1926
CLIC3	-0.75347	-0.46158	-0.60943	-0.91529	-0.57074	1.58E-05	0.005578	0.000236	9.67E-09	0.001009	4657	6154	5504	2756	5636
SRA1	-0.39812	-0.53297	-0.42566	-0.69312	-0.36422	1.61E-05	1.83E-05	0.000296	6.20E-07	0.000699	4659	4189	5570	3546	5523
SQSTM1	0.854359	0.794103	0.682097	0.730977	0.548514	1.62E-05	2.66E-06	1.02E-06	7.17E-05	0.000774	4662	3723	4040	4779	5553
SPPL2A	-0.78603	-0.98476	-0.63965	-1.01798	-0.5266	1.63E-05	3.16E-08	2.62E-05	2.44E-09	0.000551	4664	2745	4857	2550	5433
BMP2	-1.57177	-1.54564	-1.78034	-1.95908	-1.91961	1.66E-05	0.000152	1.79E-05	4.19E-06	1.11E-05	4667	4818	4758	3976	4306
BOP1	0.746948	1.135376	1.004418	1.285436	0.951359	1.67E-05	2.49E-09	1.95E-07	1.43E-11	9.73E-08	4668	2307	3655	1814	3270
MCM2	0.784422	0.903411	0.666676	0.943575	0.810691	1.69E-05	4.08E-08	5.31E-06	5.67E-10	3.10E-07	4670	2807	4419	2297	3512
YWHAG	0.469054	0.39879	0.678693	0.589532	0.779281	1.69E-05	0.000449	3.00E-07	3.26E-06	4.54E-08	4671	5213	3757	3911	3106
SRRT	0.646563	0.571094	0.415466	0.567904	0.491734	1.72E-05	0.000528	0.004239	0.00042	0.00033	4674	5275	6503	5320	5257
TCEAL8	-0.66607	-0.59336	-0.62469	-0.55844	-0.64815	1.73E-05	7.45E-06	8.37E-05	0.00012	1.46E-05	4676	3952	5174	4914	4389
RRAGC	-0.57044	-0.47963	-0.44769	-0.62939	-0.47947	1.75E-05	0.000327	0.000641	5.97E-08	0.00016	4678	5093	5809	3067	5061
TAB2	-0.62398	-0.58173	-0.66053	-0.71734	-0.65511	1.76E-05	0.000221	9.37E-07	9.22E-06	6.99E-06	4679	4947	4020	4186	4183
NKD2	1.341539	1.625237	1.315703	1.391014	1.987038	1.77E-05	2.47E-07	8.18E-06	3.92E-06	1.36E-11	4680	3184	4538	3965	1807
YEATS2	-0.46645	-0.40044	-0.46467	-0.40445	-0.43632	1.79E-05	0.000962	8.19E-05	0.000691	8.82E-05	4684	5496	5168	5489	4886
INTS1	0.71723	0.68253	0.62424	0.699689	0.98109	1.80E-05	3.25E-05	2.91E-05	3.27E-05	2.59E-10	4686	4352	4879	4546	2207

KIF2C	0.692216	0.963901	1.248677	1.081692	1.03389	1.80E-05	4.50E-06	2.80E-11	2.28E-07	1.56E-07	4687	3838	2034	3322	3363
COA1	0.588784	0.818718	0.426333	0.814483	0.803184	1.81E-05	5.60E-08	0.000231	2.02E-06	1.36E-07	4688	2866	5491	3808	3338
ORAI3	-0.58108	-0.48453	-0.69606	-0.44977	-0.92141	1.81E-05	0.000516	2.14E-08	0.001617	4.23E-10	4689	5266	3179	5804	2279
BTG2	-1.30231	-0.98814	-2.19791	-1.1636	-1.61071	1.81E-05	0.000185	2.58E-11	0.000776	7.22E-07	4691	4892	2017	5536	3669
NMD3	0.384689	0.478464	0.480405	0.467701	0.310065	1.82E-05	4.85E-07	3.46E-07	2.54E-07	0.000359	4694	3307	3788	3342	5286
LTV1	0.489052	0.613455	0.614202	0.664602	0.688492	1.82E-05	1.21E-06	1.74E-06	7.69E-08	2.59E-07	4695	3528	4160	3111	3467
TACC3	0.862117	0.770705	0.767931	0.565489	1.085481	1.83E-05	0.000285	2.49E-05	0.001812	2.52E-07	4697	5041	4844	5851	3461
CTTN	0.923776	0.840535	1.332723	0.999856	1.025072	1.83E-05	1.14E-06	3.12E-10	1.67E-07	1.61E-08	4698	3515	2389	3264	2906
RAD54L	0.558439	0.456207	0.803929	0.471966	0.674916	1.83E-05	0.005887	3.27E-07	0.001252	0.000345	4699	6176	3777	5711	5274
KDM6B	-0.63851	-0.68587	-0.52088	-0.73483	-0.76017	1.84E-05	1.30E-05	0.000514	2.47E-06	2.37E-06	4700	4099	5758	3850	3929
CDK13	0.490317	0.550478	0.390647	0.610166	0.491364	1.84E-05	6.83E-08	0.000152	2.96E-07	3.82E-05	4702	2901	5334	3370	4645
ACE	-0.81904	-1.20055	-0.84706	-0.92257	-0.69035	1.85E-05	7.69E-10	4.72E-05	5.99E-07	0.000826	4703	2130	5020	3537	5572
BBX	-0.51162	-0.48518	-0.42559	-0.56695	-0.42356	1.85E-05	8.11E-05	0.000651	7.48E-06	1.12E-05	4704	4637	5812	4126	4307
BAG4	-0.46404	-0.31111	-0.59511	-0.48995	-0.89209	1.86E-05	0.001385	5.54E-05	0.000754	8.44E-09	4705	5632	5060	5526	2774
PGAP2	0.560176	0.917304	0.602817	0.786474	0.824064	1.89E-05	6.12E-08	1.12E-06	1.81E-07	4.11E-08	4706	2887	4064	3278	3080
SAMD12	0.729931	1.014358	1.498685	0.946056	1.102804	1.90E-05	1.82E-08	4.49E-15	2.56E-07	3.60E-08	4707	2644	1056	3345	3054
UHRF1	0.713677	0.984015	0.851663	1.252994	0.869822	1.91E-05	0.00011	5.35E-05	6.82E-07	0.000135	4709	4729	5053	3571	5018
DCAF5	-0.64433	-0.5032	-0.63351	-0.65031	-0.95745	1.92E-05	4.20E-05	2.61E-06	1.02E-05	1.35E-10	4710	4430	4244	4216	2097
FGD6	-0.63106	-0.66115	-0.47531	-0.54081	-0.55059	1.92E-05	4.75E-05	0.001294	0.000443	0.002444	4711	4467	6040	5334	5974
DUX4	-0.83285	-0.65835	-1.18141	-0.74476	-0.62645	1.93E-05	2.84E-05	1.10E-09	0.000312	0.00072	4712	4316	2598	5233	5535
TMEM183	0.531989	0.605755	0.713025	0.524511	0.725189	1.93E-05	4.48E-05	9.71E-08	0.000222	3.10E-07	4713	4446	3506	5098	3510
USP39	0.5146	0.620853	0.910052	0.758998	0.746412	1.94E-05	5.21E-06	2.41E-11	1.39E-07	4.28E-10	4714	3871	2007	3227	2281
QRSL1	0.504933	0.610954	0.529297	0.611514	0.477597	1.94E-05	2.38E-07	4.63E-06	4.38E-06	0.001113	4716	3181	4387	3987	5675
PKN2	0.798399	0.737816	0.756965	0.893204	0.551529	1.95E-05	1.36E-05	1.22E-05	2.19E-06	0.001014	4718	4111	4650	3821	5637
ALG5	0.565885	0.636821	0.76965	0.532449	0.571	1.96E-05	1.45E-05	1.56E-07	2.76E-06	0.000121	4719	4123	3608	3875	4993
ARHGAP1[illegible]	-0.63405	-0.86638	-0.66853	-0.65812	-0.67269	1.96E-05	1.25E-09	5.36E-06	1.36E-05	6.63E-08	4720	2207	4422	4290	3179
MMD	-0.75941	-0.8152	-1.07785	-0.87514	-0.74977	1.98E-05	3.34E-05	2.09E-08	1.39E-05	0.000449	4721	4362	3173	4300	5375
MTG2	0.641689	1.155162	0.977191	1.158085	0.4701	1.98E-05	3.61E-07	1.75E-07	2.77E-07	0.004209	4722	3251	3634	3360	6149
ZNF358	-0.69858	-0.43237	-0.47234	-0.46842	-0.5863	2.01E-05	0.001471	0.000398	0.001312	0.000267	4723	5648	5667	5728	5208
SH3BP2	-0.61716	-0.76136	-0.46822	-0.76007	-0.39041	2.02E-05	1.97E-06	0.002887	5.11E-06	0.000653	4724	3651	6345	4026	5492
ZNF195	0.605792	0.929597	0.678344	0.909415	0.943121	2.03E-05	1.06E-06	2.50E-06	4.27E-07	5.10E-07	4725	3495	4237	3463	3606
KIF21A	0.521982	0.670673	0.551767	0.721385	0.404638	2.06E-05	5.71E-07	3.38E-08	4.77E-08	0.000814	4727	3342	3293	3028	5568
PIK3C3	-0.5799	-0.42342	-0.75798	-0.59752	-0.88998	2.06E-05	0.005214	4.89E-06	0.000331	3.29E-09	4728	6121	4406	5246	2618
IBSP	-0.98638	-0.46976	-0.50151	-0.58985	-0.69795	2.06E-05	0.002061	0.001736	6.47E-06	8.35E-05	4729	5778	6134	4083	4863
TXNRD1	0.519466	0.564041	0.710743	0.768219	0.529962	2.08E-05	3.26E-05	4.43E-06	4.33E-07	3.20E-05	4730	4354	4377	3465	4585
AP2A2	-0.40544	-0.64866	-0.42979	-0.40522	-0.41992	2.10E-05	7.67E-07	0.000953	0.002768	0.00048	4732	3411	5942	6010	5393
KIF12	1.012054	1.128003	0.680257	0.923995	1.309888	2.11E-05	1.10E-06	0.000686	0.000166	2.06E-07	4735	3506	5834	4999	3420
UBE3C	0.612813	0.588861	0.842871	0.670533	0.745162	2.13E-05	0.000275	8.41E-08	5.78E-05	1.15E-05	4737	5025	3472	4718	4321
ANKHD1	0.501261	0.449345	0.46984	0.37237	0.414794	2.14E-05	0.000312	2.16E-05	0.000806	0.000224	4738	5075	4795	5545	5149
KCNE3	0.860432	0.730626	0.879833	1.072843	0.530833	2.16E-05	2.96E-06	6.62E-05	2.98E-09	0.003349	4740	3755	5111	2582	6066
CCL28	0.983281	1.006499	1.762445	1.095127	1.075541	2.17E-05	1.96E-05	2.01E-10	2.46E-08	1.51E-05	4742	4206	2321	2918	4399
MCEE	-0.48339	-0.45317	-0.66205	-0.68661	-0.90125	2.20E-05	8.11E-06	3.45E-06	5.74E-07	6.83E-11	4744	3973	4308	3524	2001
KIFBP	-0.6144	-0.59817	-0.49406	-0.53373	-0.69383	2.23E-05	0.000292	0.000192	0.001027	2.98E-07	4747	5053	5426	5638	3501
DPM3	0.512742	0.35499	0.580517	0.469309	0.627615	2.25E-05	0.007216	1.13E-06	0.001245	2.41E-06	4749	6270	4072	5708	3931
PYCR2	0.732783	0.959357	0.524566	0.780624	0.829265	2.27E-05	1.20E-07	0.001968	3.73E-07	3.25E-07	4752	3040	6190	3427	3519
PYM1	0.568684	0.47824	0.497559	0.465659	0.423136	2.28E-05	0.002169	0.000192	0.003263	0.003908	4753	5798	5425	6078	6115
TGFBR2	-0.58141	-0.7866	-0.67847	-0.58441	-0.5154	2.28E-05	7.24E-05	3.10E-05	0.00429	0.001379	4755	4597	4906	6190	5750
PTK7	-1.50795	-1.00557	-1.02867	-0.81543	-0.92082	2.28E-05	6.37E-05	0.000153	0.008698	0.002298	4757	4550	5337	6467	5959
GPBP1L1	0.577491	0.591389	0.665539	0.619447	0.58343	2.29E-05	2.31E-05	3.56E-06	4.68E-05	6.12E-06	4759	4256	4319	4654	4155
PDS5A	0.450895	0.45388	0.594852	0.48821	0.564882	2.30E-05	3.64E-05	1.84E-06	3.29E-07	3.72E-06	4760	4383	4175	3401	4034
HAT1	0.42002	0.298325	0.428216	0.376918	0.535087	2.35E-05	0.003481	8.69E-06	0.000357	5.06E-06	4763	5961	4551	5272	4109
MAGT1	0.596879	0.380659	0.479333	0.55701	0.399674	2.36E-05	0.003833	7.56E-05	5.96E-05	0.002179	4767	6000	5140	4726	5944
GFM1	0.486077	0.627496	0.710258	0.589263	0.595089	2.39E-05	5.71E-07	1.37E-08	5.46E-06	1.03E-06	4769	3341	3084	4043	3746
SLC10A7	-0.46924	-0.38072	-0.48014	-0.46805	-0.66074	2.40E-05	0.001383	0.000226	0.000326	3.81E-09	4770	5630	5482	5245	2644
ZNF511	0.574535	0.724878	0.788515	0.745508	0.818548	2.40E-05	2.61E-05	2.96E-07	7.47E-06	9.56E-07	4771	4287	3749	4124	3725
MRPL33	0.487658	0.555869	0.529332	0.647704	0.599759	2.41E-05	0.00024	4.41E-05	1.13E-05	4.93E-07	4772	4973	5002	4248	3600
SLC41A1	-0.66743	-0.55094	-0.82617	-0.6788	-1.06444	2.43E-05	0.000135	2.69E-10	2.79E-06	1.26E-11	4774	4780	2366	3881	1796
CRACR2B	0.765124	0.672186	0.631206	0.711625	1.039477	2.43E-05	0.000185	0.000655	0.000527	1.05E-06	4775	4893	5815	5402	3751
APOL2	-0.99406	-0.91875	-0.80742	-0.83297	-0.94064	2.44E-05	0.000332	0.000183	0.001066	3.51E-06	4777	5101	5410	5650	4022
SLC39A4	0.60883	0.863061	0.878705	1.000633	0.771969	2.45E-05	6.53E-07	1.29E-06	1.74E-08	1.31E-06	4778	3372	4102	2863	3794
CELF2	-0.67877	-0.74969	-0.78869	-0.8134	-0.76555	2.45E-05	2.29E-08	9.18E-08	8.74E-09	1.94E-09	4780	2688	3493	2736	2532
TCOF1	0.733282	0.628547	0.640463	0.538543	0.578342	2.47E-05	0.000119	4.18E-05	0.000263	0.000512	4781	4744	4982	5166	5415
TRIP13	0.798822	1.163453	1.343992	1.026512	0.90502	2.49E-05	7.89E-07	4.38E-10	8.01E-06	9.93E-06	4783	3420	2443	4147	4275
ZNF518A	0.685898	0.851257	0.598985	0.727558	0.383574	2.52E-05	1.84E-08	9.51E-06	6.67E-07	0.0016	4784	2645	4575	3566	5817
NCOA5	0.559327	0.771745	0.492382	0.723752	0.610424	2.52E-05	1.56E-08	6.31E-05	2.27E-07	4.05E-06	4786	2630	5094	3321	4051
SLC25A13	0.688565	0.891954	0.663185	1.017035	0.697275	2.53E-05	8.16E-06	8.55E-05	1.08E-07	9.00E-06	4787	3975	5187	3182	4255
NSF	0.417551	0.388314	0.541059	0.537357	0.448799	2.56E-05	0.000644	1.76E-06	5.21E-07	1.99E-06	4789	5346	4163	3500	3877
ITPR2	-1.15139	-1.25839	-1.71965	-1.20425	-1.78603	2.57E-05	7.11E-08	1.28E-10	1.34E-05	2.26E-09	4790	2914	2258	4283	2563
NCKAP1	0.776465	0.752566	1.275039	0.886414	0.806631	2.58E-05	8.64E-07	2.66E-11	7.59E-08	3.34E-06	4791	3441	2025	3106	4001
ZNF577	-0.52217	-0.33315	-0.39472	-0.53497	-0.53428	2.59E-05	0.003452	0.003384	4.15E-05	4.36E-05	4792	5956	6404	4619	4681
ATG7	-0.65333	-0.66084	-0.76167	-0.69661	-0.80701	2.62E-05	0.000529	1.05E-06	0.000182	3.58E-06	4795	5280	4049	5034	4028
TMEM9	0.656059	0.85412	0.728846	0.997651	0.819092	2.62E-05	1.61E-06	0.001034	2.77E-08	6.58E-06	4797	3599	5964	2939	4169
NUP88	0.390617	0.457984	0.498953	0.385862	0.355434	2.65E-05	3.34E-06	3.49E-09	4.40E-05	0.003595	4799	3781	2818	4638	6086

SEPHS2	0.818997	0.946999	1.09307	0.919606	0.738081	2.65E-05	1.22E-05	6.94E-09	3.75E-05	0.001233	4800	4085	2939	4590	5714
PAQR8	0.837646	0.598739	0.533207	0.654502	0.902954	2.65E-05	0.004023	0.004579	0.003048	4.36E-06	4801	6017	6536	6059	4070
MBOAT7	0.424457	0.549321	0.500522	0.483896	0.51538	2.66E-05	8.13E-06	1.66E-05	3.66E-05	2.26E-06	4802	3974	4734	4577	3916
IFNAR1	-0.54539	-0.50162	-0.71187	-0.4557	-0.74599	2.68E-05	1.42E-05	2.90E-08	0.000522	2.45E-09	4803	4116	3249	5399	2580
JMJD8	0.527362	0.390037	0.549757	0.648819	0.413999	2.68E-05	0.001167	1.15E-05	5.29E-07	0.00095	4804	5574	4628	3509	5610
REEP6	1.154157	1.139792	0.906825	1.25733	1.058276	2.72E-05	4.66E-05	9.71E-07	6.37E-06	9.58E-07	4805	4457	4027	4078	3726
TBC1D20	-0.75717	-0.65633	-0.62498	-0.62514	-0.68924	2.74E-05	1.23E-05	2.94E-05	0.000258	6.70E-08	4806	4086	4883	5156	3184
CLPP	0.49957	0.476142	0.569882	0.606268	0.473952	2.77E-05	0.000707	2.80E-05	1.75E-05	6.41E-05	4808	5380	4872	4362	4792
ACAP2	-0.37529	-0.35275	-0.35376	-0.51762	-0.30951	2.78E-05	9.11E-05	6.60E-06	1.22E-06	0.000404	4809	4673	4472	3692	5332
MTCH2	0.652693	0.65807	0.886851	0.800005	0.934981	2.83E-05	7.99E-05	2.13E-07	1.03E-05	1.17E-08	4813	4630	3674	4218	2850
MON2	-0.65522	-0.58913	-0.56119	-0.53888	-0.75546	2.83E-05	2.98E-06	3.37E-06	0.000125	4.54E-10	4814	3760	4303	4923	2292
NDUFS8	0.733016	0.701705	0.765167	0.661149	0.51142	2.85E-05	2.76E-06	4.96E-05	2.83E-05	0.004702	4815	3736	5032	4498	6196
FOXK1	0.593029	0.639385	0.704341	0.776223	0.664694	2.87E-05	4.15E-06	3.23E-08	9.69E-07	5.43E-09	4819	3826	3277	3632	2700
CD320	0.900376	1.606996	0.866276	1.466779	1.036	2.91E-05	4.48E-11	3.02E-05	1.43E-11	5.09E-08	4821	1731	4895	1815	3127
CHPF2	-0.69912	-0.53441	-0.40008	-0.48338	-0.50792	2.94E-05	0.000322	0.000838	7.48E-05	0.000447	4823	5090	5911	4790	5370
RIOX1	-0.47469	-0.25642	-0.50069	-0.42763	-0.3581	2.94E-05	0.00792	1.14E-06	2.78E-05	9.17E-05	4824	6324	4073	4489	4901
NUP107	0.527502	0.582866	0.490263	0.610134	0.396665	2.97E-05	1.22E-05	1.41E-05	5.60E-06	0.000463	4826	4084	4687	4052	5385
STK39	0.515276	0.547809	0.684865	0.794913	0.571674	3.02E-05	0.006714	4.21E-05	5.05E-05	6.88E-05	4830	6233	4984	4677	4817
CAPN10	-0.37472	-0.29589	-0.38388	-0.39535	-0.39233	3.06E-05	0.001048	2.50E-06	3.68E-05	1.58E-05	4831	5530	4236	4581	4409
WDR3	0.633213	0.622216	0.618291	0.587811	0.541838	3.09E-05	0.000454	0.000255	0.000676	0.000223	4835	5217	5524	5484	5145
LRIG2	-0.5422	-0.40013	-0.48962	-0.31101	-0.60107	3.10E-05	0.000204	4.25E-05	0.00409	1.64E-06	4836	4916	4989	6167	3841
SPDYE2	-0.58059	-0.41296	-0.92378	-0.68008	-0.67981	3.11E-05	0.001949	8.97E-08	0.000298	0.002921	4838	5749	3488	5219	6029
TCEA1	0.584074	0.762733	0.800956	0.635299	0.561802	3.16E-05	7.20E-07	3.17E-08	7.00E-06	9.23E-05	4839	3399	3270	4111	4904
TFDP2	0.595845	0.531365	0.525138	0.612224	0.78628	3.17E-05	4.45E-05	0.000117	3.09E-05	3.14E-07	4840	4444	5267	4528	3514
HGH1	0.643048	0.921175	0.881982	0.956713	0.935839	3.17E-05	5.03E-09	6.82E-09	2.17E-09	8.21E-13	4841	2439	2934	2529	1461
CCDC47	0.340809	0.280278	0.298024	0.352591	0.522998	3.17E-05	0.001013	0.000202	0.001502	1.95E-05	4842	5512	5442	5776	4461
NDUFA8	0.501472	0.404161	0.844429	0.540201	0.470176	3.18E-05	9.13E-05	3.66E-09	7.29E-05	0.000222	4843	4674	2826	4783	5144
SEPTIN7	-0.46559	-0.62561	-0.42162	-0.56629	-0.5814	3.19E-05	2.03E-06	0.000447	3.74E-07	2.63E-05	4845	3662	5704	3429	4528
VPS72	0.559377	0.699311	0.521876	0.700117	0.51385	3.20E-05	0.00037	0.001033	0.000298	0.000401	4846	5144	5963	5217	5328
TATDN1	0.502374	0.597613	0.711027	0.443637	0.643503	3.21E-05	2.36E-05	7.16E-06	0.000508	1.28E-06	4848	4262	4497	5382	3790
PSD4	0.641739	0.801222	0.666299	0.680728	0.804212	3.22E-05	4.77E-05	7.86E-05	0.000346	9.17E-06	4849	4471	5154	5261	4257
TTC39A	0.865626	1.108537	0.997125	1.135782	1.017343	3.22E-05	3.28E-07	1.99E-09	1.51E-07	2.40E-08	4850	3236	2702	3243	2975
KLHL24	-0.56966	-0.66851	-0.47508	-0.65657	-0.54022	3.23E-05	5.11E-05	0.001325	0.000269	4.46E-05	4851	4491	6048	5178	4688
GAK	0.570614	0.469452	0.875174	0.470859	0.681334	3.27E-05	0.00019	3.92E-10	0.0004	2.07E-10	4852	4899	2428	5308	2161
BCKDHB	0.689315	0.738731	0.604732	0.769212	0.482786	3.28E-05	1.02E-06	5.05E-05	6.64E-06	1.10E-06	4854	3483	5041	4089	3759
NAALADL2	0.912162	1.008173	0.887236	1.05795	1.350643	3.32E-05	1.41E-08	2.58E-05	1.94E-09	1.03E-08	4857	2618	4852	2511	2823
FURIN	-0.99479	-1.43046	-1.03538	-1.24161	-0.99329	3.36E-05	4.66E-09	3.98E-05	3.21E-06	6.61E-06	4858	2424	4969	3907	4170
UBE2L3	0.60857	0.538587	0.621681	0.561028	0.608497	3.36E-05	0.000367	7.32E-05	0.000553	4.11E-07	4859	5141	5127	5415	3568
CTSZ	-0.99	-1.63613	-1.27392	-1.47171	-1.31569	3.37E-05	4.24E-08	3.40E-07	1.21E-07	1.89E-05	4860	2815	3783	3201	4453
DCAF12L2	-0.51695	-0.40664	-0.67602	-0.57215	-0.56792	3.41E-05	0.002792	3.21E-08	0.000151	1.38E-05	4863	5869	3274	4974	4375
ST6GALNA	1.256905	1.402036	1.635576	1.461544	1.809464	3.44E-05	2.83E-08	4.09E-10	2.60E-07	9.53E-08	4865	2730	2434	3347	3266
BMP2K	-0.63454	-0.77012	-0.53829	-0.67376	-0.70434	3.44E-05	6.87E-07	2.36E-05	1.37E-05	1.92E-07	4866	3386	4824	4292	3408
ACTN1	-1.07497	-1.45379	-0.77615	-1.29154	-0.89414	3.50E-05	2.08E-11	0.002122	2.41E-07	0.000169	4868	1620	6224	3336	5073
CCDC124	0.538631	1.150009	1.003397	1.031127	0.701497	3.55E-05	1.49E-11	4.84E-09	6.57E-11	5.00E-06	4870	1575	2869	2011	4105
C3orf52	0.731126	0.881804	0.754773	0.876062	0.98937	3.57E-05	3.92E-07	2.24E-06	9.82E-06	2.23E-06	4872	3269	4205	4209	3911
CYBC1	-0.57194	-0.53668	-0.96668	-0.54532	-0.88825	3.59E-05	3.39E-06	7.09E-11	5.44E-05	4.94E-10	4875	3783	2155	4697	2309
TCEAL9	-1.23387	-1.09453	-0.81906	-1.30216	-1.20446	3.59E-05	5.68E-07	0.000265	4.22E-09	4.96E-06	4876	3339	5536	2639	4102
MRTO4	0.692046	0.911369	1.094969	0.98011	0.65682	3.60E-05	1.28E-06	3.93E-09	9.48E-07	0.000144	4878	3547	2838	3625	5035
OR10AD1	-0.54605	-0.42074	-0.79094	-0.69841	-0.58	3.62E-05	0.001237	0.000123	0.000104	0.000892	4879	5594	5275	4869	5595
FMNL2	-0.6505	-0.56102	-0.55075	-0.55826	-0.59302	3.65E-05	3.88E-07	2.56E-07	4.21E-06	8.74E-06	4881	3268	3711	3979	4249
RAB10	0.51451	0.332008	0.779129	0.539408	0.441164	3.68E-05	0.001012	1.15E-08	0.000115	0.001655	4882	5511	3044	4893	5836
TGFBR3	-0.50182	-0.51114	-0.45975	-0.51589	-0.76836	3.70E-05	1.94E-05	3.41E-05	5.57E-06	4.70E-06	4885	4204	4930	4051	4082
ERLEC1	-0.6558	-0.56298	-0.54188	-0.48864	-0.63462	3.74E-05	7.95E-06	1.06E-05	0.000273	2.23E-05	4890	3967	4609	5186	4492
SMPD2	0.513686	0.402849	0.831315	0.43611	0.4406	3.76E-05	0.006784	2.29E-11	0.003541	0.000332	4891	6239	1998	6108	5258
FH	0.618964	0.481124	0.689326	0.416991	0.800605	3.79E-05	0.000232	2.40E-06	0.00017	3.90E-08	4892	4960	4225	5003	3066
CSNK1D	0.580046	0.48588	0.625813	0.552029	0.392277	3.81E-05	0.000142	1.94E-05	7.12E-05	0.000257	4894	4796	4772	4775	5195
DCLRE1C	-0.36975	-0.33846	-0.68717	-0.55771	-0.52768	3.83E-05	0.000108	1.17E-08	1.63E-05	2.73E-06	4896	4721	3050	4349	3955
H2AC21	1.075394	0.624453	1.391085	0.890795	1.194895	3.84E-05	0.00044	1.64E-07	1.85E-05	6.11E-07	4897	5205	3621	4377	3641
PSG1	-0.62951	-0.47731	-0.78598	-0.6552	-0.49902	3.86E-05	0.000123	3.70E-08	2.35E-05	0.001278	4898	4752	3313	4429	5729
QTRT1	0.61871	0.923239	0.454021	0.882425	0.537094	3.88E-05	5.97E-08	0.00056	5.94E-09	4.74E-05	4899	2880	5777	2682	4709
CTBP2	0.594828	0.668515	0.739687	0.71888	1.070267	3.89E-05	6.99E-05	7.53E-06	6.05E-05	4.26E-09	4900	4587	4510	4732	2664
PROX1	1.012109	1.011634	0.856242	1.081317	1.276536	3.90E-05	6.19E-06	0.000141	7.80E-07	2.72E-08	4901	3914	5311	3585	2997
ACAT2	0.980844	0.785766	1.498905	0.778098	0.796025	3.94E-05	0.000486	4.90E-12	0.000519	0.000154	4905	5242	1781	5395	5051
ATG3	0.606165	0.528427	0.655956	0.640852	0.725882	3.96E-05	0.000669	1.49E-06	1.83E-05	2.23E-07	4907	5366	4124	4375	3443
PES1	0.660857	0.739417	0.9344	0.847667	0.653778	3.98E-05	2.84E-06	1.55E-05	1.16E-06	0.000187	4909	3748	4715	3683	5107
FRYL	0.67758	0.861803	0.902014	1.108178	0.865555	3.98E-05	7.57E-06	5.97E-07	1.04E-08	7.98E-09	4911	3959	3909	2768	2767
CHMP4C	0.589338	0.617089	1.048742	0.798127	0.986184	3.99E-05	0.000164	3.27E-10	2.53E-05	6.44E-12	4912	4842	2403	4458	1705
PLGRKT	0.759478	0.63526	1.08816	0.715004	0.641922	4.03E-05	0.000625	8.56E-11	9.56E-05	2.78E-05	4914	5338	2186	4844	4541
PAK4	0.551792	0.665402	1.067675	0.465592	0.985543	4.04E-05	0.000528	1.14E-08	0.00101	6.33E-08	4915	5274	3042	5629	3171
ETNK1	0.488722	0.429294	0.571224	0.634736	0.705622	4.04E-05	0.000332	6.27E-07	2.43E-05	1.08E-05	4916	5100	3924	4441	4295
BAK1	0.472588	0.691221	0.667785	0.572791	0.669484	4.08E-05	1.34E-07	5.91E-10	7.35E-06	4.45E-09	4920	3059	2493	4119	2673
ASPH	1.121312	1.030365	1.735115	1.648199	1.502485	4.13E-05	6.35E-07	5.53E-10	8.58E-11	3.71E-07	4925	3364	2476	2046	3555
RRP1	0.6419	0.844056	0.798172	0.611886	0.741488	4.14E-05	3.52E-07	1.03E-06	0.000118	4.53E-07	4926	3247	4044	4904	3583

HIP1R	0.813908	0.970088	0.936705	1.165708	1.085533	4.16E-05	1.44E-05	5.04E-06	4.23E-07	3.85E-09	4927	4121	4411	3456	2646
SUGT1	0.587609	0.880613	0.899758	0.762249	0.898459	4.16E-05	5.91E-08	2.25E-08	4.27E-07	7.69E-10	4930	2877	3185	3458	2379
MALSU1	0.589898	0.576489	0.650633	0.493646	0.615968	4.18E-05	1.75E-05	1.75E-06	0.000181	2.10E-06	4932	4174	4161	5032	3888
OST4	0.487076	0.3581	0.495837	0.514742	0.581598	4.24E-05	0.001467	4.06E-06	3.85E-05	3.86E-06	4935	5646	4347	4600	4039
PYCARD	0.503377	0.572555	0.357562	0.540808	0.829476	4.34E-05	4.59E-05	0.005179	3.75E-05	5.84E-08	4939	4453	6591	4588	3156
GRWD1	0.513672	0.574169	0.693251	0.496101	0.680234	4.40E-05	3.32E-05	6.04E-06	0.000495	6.46E-07	4944	4361	4451	5371	3651
DENR	0.714546	0.680397	0.462568	0.887885	0.542693	4.43E-05	5.84E-05	0.000281	7.59E-08	7.11E-08	4945	4530	5553	3108	3203
CDC123	0.53763	0.62415	0.713751	0.662026	0.58972	4.44E-05	5.02E-06	3.96E-08	1.00E-06	2.63E-08	4946	3861	3328	3644	2991
FBXW4	-0.48407	-0.37198	-0.78622	-0.52958	-0.50772	4.45E-05	0.005911	1.90E-07	0.001042	0.000198	4947	6178	3652	5644	5119
RPP38	0.592368	0.737994	0.977191	0.65719	0.674504	4.52E-05	6.55E-05	1.18E-09	3.00E-05	9.49E-05	4951	4562	2615	4520	4914
ESD	0.878574	0.846773	1.157057	0.907763	1.020858	4.54E-05	3.46E-05	8.42E-07	9.57E-07	1.46E-07	4952	4365	3993	3629	3350
OR56A4	-0.41364	-0.3654	-0.49642	-0.49145	-0.34501	4.55E-05	0.00058	7.82E-05	2.89E-05	0.005507	4954	5315	5151	4503	6276
CDC42BPG	0.818988	1.153538	1.095357	1.189699	0.765486	4.56E-05	6.93E-06	1.26E-06	5.67E-07	0.000103	4955	3933	4096	3522	4946
DARS1	0.660522	1.01	0.719246	0.951446	0.653748	4.58E-05	1.48E-11	3.91E-07	4.51E-09	5.64E-06	4956	1572	3816	2645	4137
NDUFB8	0.555966	0.48872	0.499995	0.514192	0.431287	4.64E-05	1.67E-05	0.000403	4.16E-05	0.000157	4958	4155	5669	4620	5053
CHD4	0.431602	0.36649	0.359902	0.310684	0.421569	4.65E-05	0.000793	0.001364	0.003813	0.000114	4959	5415	6061	6136	4972
KDM4B	-0.61834	-0.52681	-0.94497	-0.57966	-0.72304	4.69E-05	0.000648	3.81E-09	0.000553	1.52E-06	4962	5354	2835	5414	3825
UBP1	0.542482	0.698882	0.785006	0.804506	0.715959	4.75E-05	1.06E-08	3.71E-10	2.42E-09	5.23E-08	4966	2562	2416	2547	3139
SPTBN2	0.868421	1.006106	0.584546	1.016078	0.700679	4.75E-05	2.05E-06	3.63E-05	5.13E-06	1.42E-05	4967	3665	4945	4029	4382
ZMPSTE24	0.471671	0.439245	0.527677	0.535408	0.38021	4.78E-05	0.005575	1.71E-05	0.000724	0.003737	4968	6153	4740	5512	6097
ARHGAP4	-1.28668	-1.81734	-2.05808	-2.2611	-1.66642	4.78E-05	3.82E-08	1.25E-09	2.36E-11	1.36E-06	4969	2789	2622	1886	3805
PTGS2	-1.72464	-2.02208	-2.52949	-2.65979	-2.9218	4.85E-05	0.000148	1.95E-05	1.95E-05	3.27E-08	4972	4811	4775	4396	3035
PPP1CA	0.540132	0.409097	0.725216	0.6434	0.42628	4.88E-05	0.000915	4.72E-07	7.62E-07	0.001502	4975	5476	3855	3582	5795
SUCLA2	0.683613	0.692329	1.099539	0.636747	0.854108	4.99E-05	0.000147	1.28E-06	0.002281	1.60E-05	4980	4806	4101	5936	4414
NIFK	0.667094	1.027802	0.817539	0.926808	0.816106	4.99E-05	1.11E-08	5.25E-07	6.97E-08	3.95E-08	4982	2571	3880	3092	3068
CCDC50	-0.5355	-0.62195	-0.84253	-0.76575	-0.59868	5.00E-05	1.20E-06	1.36E-12	1.86E-08	9.53E-07	4983	3527	1625	2872	3723
MYBPHL	-0.60686	-0.45158	-0.62079	-0.69843	-0.79736	5.01E-05	0.002848	0.000696	5.49E-05	3.04E-06	4984	5876	5842	4704	3985
SEMA4D	-0.64064	-0.54425	-0.91442	-0.61944	-0.73844	5.02E-05	0.003235	5.26E-07	0.002751	5.11E-05	4985	5926	3881	6009	4731
VPS36	0.711744	0.794982	0.872546	0.624911	0.953177	5.11E-05	6.66E-06	8.13E-07	2.92E-06	4.32E-08	4986	3925	3983	3898	3098
ACOX1	0.683762	0.819267	0.902169	0.97766	0.770651	5.19E-05	1.96E-05	5.34E-09	1.49E-06	1.02E-06	4989	4205	2886	3730	3744
FGFR1OP2	-0.45934	-0.40055	-0.74396	-0.62756	-0.67946	5.19E-05	0.000772	2.77E-08	1.23E-05	6.86E-08	4990	5410	3234	4267	3191
GORASP2	0.561744	0.608291	0.823631	0.738803	0.89693	5.22E-05	1.33E-05	2.57E-07	1.18E-06	9.34E-07	4992	4104	3713	3686	3721
POT1	-0.4838	-0.42004	-0.52566	-0.44784	-0.55137	5.28E-05	0.000167	5.97E-06	0.000342	6.61E-05	4994	4854	4448	5258	4802
TSEN54	0.617045	0.749287	0.517071	0.639924	0.850651	5.29E-05	1.08E-05	0.000716	6.71E-06	8.83E-06	4996	4046	5854	4093	4252
PRKAR2A	0.640467	0.540222	0.842441	0.604405	0.65291	5.31E-05	0.000884	7.83E-08	0.000263	2.88E-05	4997	5461	3457	5167	4547
PDCD2	0.662761	0.735911	0.537439	0.454902	0.855388	5.32E-05	2.65E-06	8.03E-05	0.001299	8.17E-08	4998	3722	5160	5721	3238
TARBP1	0.81857	1.170414	0.762042	1.147393	1.422666	5.32E-05	8.44E-08	7.67E-06	2.73E-07	6.13E-09	4999	2956	4516	3359	2716
TSPY1	-1.18997	-0.9113	-1.41273	-1.20197	-0.83178	5.41E-05	0.001663	2.92E-06	5.08E-05	0.000104	5001	5679	4272	4679	4948
TADA2B	-0.57531	-0.88333	-0.52809	-0.90534	-0.65813	5.48E-05	1.14E-07	0.000269	4.27E-07	9.67E-07	5002	3024	5543	3462	3728
PRKAB2	-0.4805	-0.36985	-0.69988	-0.53788	-0.46823	5.52E-05	0.000373	3.82E-08	4.11E-05	2.13E-05	5003	5148	3320	4616	4480
SRCAP	0.39891	0.499468	0.754752	0.47967	0.556356	5.55E-05	0.00036	1.14E-06	1.35E-05	0.000126	5006	5129	4074	4287	4999
IRS2	1.083547	1.138239	1.882162	1.414126	1.317241	5.61E-05	1.75E-05	4.19E-12	3.35E-06	9.12E-07	5009	4175	1759	3919	3715
LMAN1	-0.63777	-1.03875	-0.85979	-1.08262	-0.87296	5.69E-05	1.94E-09	5.86E-06	2.92E-09	3.57E-07	5011	2268	4442	2576	3541
KCNJ12	-0.58642	-0.69937	-1.03188	-0.97419	-0.70562	5.69E-05	1.85E-05	1.15E-06	2.02E-06	6.36E-05	5012	4193	4076	3806	4787
ZDHHC13	0.687125	0.808923	0.509626	0.64168	0.747788	5.69E-05	2.44E-06	0.000226	0.000203	1.32E-06	5013	3704	5481	5066	3796
BABAM1	0.426317	0.344858	0.458249	0.490044	0.68773	5.71E-05	0.005374	0.000208	0.001329	6.93E-05	5014	6132	5455	5734	4820
ANKRD49	-0.39319	-0.28945	-0.38434	-0.42029	-0.70628	5.79E-05	0.006259	0.000715	0.002308	1.05E-05	5017	6199	5853	5939	4285
LMBR1	0.525334	0.687188	0.52761	0.490762	0.492865	5.81E-05	1.80E-07	2.09E-05	2.84E-05	0.000282	5018	3129	4790	4500	5220
KIFC1	0.93403	0.947753	1.094232	0.677295	0.758789	5.83E-05	5.74E-05	1.04E-08	0.002036	0.000139	5019	4524	3021	5888	5027
HIRA	0.483773	0.531631	0.561706	0.411815	0.416796	5.88E-05	6.02E-05	4.24E-07	0.001647	0.001512	5021	4539	3833	5812	5799
CEACAM7	1.567558	1.951801	2.352302	2.619413	1.710832	5.88E-05	3.38E-06	9.25E-07	9.19E-10	3.76E-05	5022	3782	4017	2384	4635
PBDC1	0.647709	0.950385	0.503974	1.002503	0.336266	5.88E-05	9.33E-07	0.000924	1.73E-07	0.009211	5023	3465	5933	3271	6519
TMEM65	-0.64023	-0.61599	-0.70129	-0.76261	-0.83159	6.00E-05	3.46E-05	7.83E-07	4.16E-06	1.69E-09	5028	4366	3972	3975	2510
SNAP23	-0.51454	-0.76603	-0.70883	-0.68847	-0.56208	6.00E-05	8.34E-07	1.24E-05	2.21E-05	0.000381	5029	3430	4658	4414	5303
TRPM4	0.811339	0.817903	1.130283	1.055543	1.192524	6.02E-05	7.17E-05	3.84E-08	7.98E-08	1.09E-08	5030	4593	3323	3123	2834
PPP1R11	0.510637	0.420966	0.73676	0.524483	0.658439	6.08E-05	0.005315	3.58E-07	0.001595	1.65E-06	5032	6126	3794	5800	3844
PAIP1	0.582037	0.465502	0.672273	0.651831	0.70216	6.08E-05	0.001549	1.61E-05	1.75E-05	2.00E-06	5033	5663	4720	4365	3878
ANAPC16	0.444007	0.59755	0.651624	0.593748	0.57822	6.13E-05	1.83E-06	2.53E-09	7.38E-06	2.79E-09	5034	3635	2756	4121	2591
LAMP1	0.912955	0.698458	0.969416	0.923857	1.204561	6.15E-05	0.000327	4.36E-06	7.34E-06	2.94E-10	5037	5094	4370	4118	2224
PLCB4	1.199994	1.438452	1.364167	1.523769	1.606928	6.20E-05	6.44E-06	1.95E-05	3.11E-06	3.01E-06	5039	3919	4774	3905	3979
SLC19A1	0.59405	0.986713	0.561794	0.77975	0.608037	6.29E-05	7.09E-10	0.000209	1.67E-08	0.000201	5042	2114	5456	2856	5125
LPP	-0.82106	-0.68746	-0.55873	-0.69051	-0.5953	6.30E-05	0.001688	0.005614	0.001013	0.005105	5043	5686	6627	5632	6239
FKBP1A	-0.56662	-0.82788	-0.75721	-0.87946	-0.7866	6.34E-05	2.35E-06	6.60E-07	1.62E-06	0.000158	5046	3698	3932	3750	5057
AMOTL2	-0.67771	-0.51535	-0.46248	-0.59006	-0.46655	6.40E-05	0.000474	0.000187	0.000658	0.000972	5049	5226	5415	5472	5617
LNX2	0.679821	0.882779	0.843967	0.859231	0.710781	6.43E-05	3.99E-05	2.56E-06	5.94E-05	0.002875	5051	4413	4240	4725	6026
FN3K	0.595077	0.725134	0.930486	0.794278	0.56308	6.60E-05	5.31E-06	1.50E-10	1.13E-06	0.000369	5055	3878	2279	3670	5296
MVD	0.709467	0.654714	0.910202	0.728499	0.881068	6.60E-05	0.000329	9.51E-08	0.000119	8.19E-08	5056	5098	3502	4911	3239
SLC25A23	0.567904	0.707008	0.735558	0.650988	0.465802	6.78E-05	4.66E-06	2.42E-06	7.99E-08	0.001089	5064	3849	4229	3124	5667
TMED10	0.716216	0.574958	0.694234	0.659098	0.592997	6.79E-05	0.000223	5.11E-05	0.0004	0.000457	5066	4948	5044	5309	5381
KCTD5	0.550116	0.389362	0.407218	0.559136	0.508676	6.89E-05	0.007623	0.004356	0.000204	9.99E-05	5069	6301	6519	5067	4928
SPTLC2	0.758474	0.778792	1.330338	0.74499	1.331302	6.92E-05	2.45E-05	7.59E-11	0.000237	3.73E-08	5070	4268	2164	5121	3060
TMEM70	0.591638	0.840606	0.891125	0.839009	0.509609	6.92E-05	8.78E-06	1.46E-06	2.61E-05	0.004044	5071	4001	4121	4466	6131
PROM1	1.170248	1.044392	1.612314	1.680997	1.725988	7.00E-05	7.40E-05	4.70E-07	7.54E-09	1.43E-08	5075	4599	3854	2711	2885

NOL11	0.537792	0.617404	0.648266	0.762725	0.886936	7.03E-05	5.75E-06	2.96E-05	2.58E-06	1.30E-06	5078	3893	4884	3857	3791
ACP6	0.432494	0.50967	0.405901	0.407934	0.470552	7.10E-05	1.71E-05	0.000281	0.00038	3.13E-06	5079	4164	5554	5291	3990
ING2	-0.612	-0.71405	-0.56723	-0.74703	-0.60968	7.12E-05	7.01E-08	5.49E-07	2.07E-07	1.15E-06	5080	2908	3889	3305	3764
MDN1	0.616428	0.703323	0.524067	0.674237	0.303393	7.13E-05	1.02E-05	0.000468	1.80E-06	0.009862	5081	4032	5716	3774	6556
SPTLC1	0.569436	0.675073	1.214191	0.752298	0.541949	7.13E-05	0.003803	2.16E-07	0.002925	0.000257	5082	5996	3676	6041	5194
PEX16	0.3769	0.636521	0.487963	0.703454	0.541525	7.14E-05	3.40E-08	2.39E-05	6.45E-10	4.24E-05	5083	2764	4828	2326	4673
SPC25	0.523636	0.708084	0.608811	0.552286	0.529647	7.25E-05	1.58E-05	5.27E-07	0.000459	0.000524	5086	4145	3882	5344	5418
TRIM59	-0.55613	-0.53949	-0.53174	-0.77971	-0.6817	7.34E-05	0.000511	0.000222	5.81E-07	1.52E-06	5089	5263	5474	3530	3824
TBXAS1	-0.78067	-0.65177	-1.13243	-0.55812	-0.65981	7.36E-05	0.000246	1.94E-09	0.006012	0.00231	5090	4985	2693	6309	5962
HBP1	-0.66076	-0.65057	-0.62151	-0.61478	-0.80746	7.46E-05	2.52E-05	0.000133	0.000498	1.27E-05	5091	4277	5298	5373	4357
KIAA0895L	-0.40966	-0.4276	-0.60766	-0.4683	-0.56502	7.50E-05	0.000585	6.22E-06	0.001203	6.93E-06	5092	5317	4459	5695	4180
ZNF140	0.385571	0.438308	0.429196	0.591492	0.57411	7.52E-05	1.61E-05	1.38E-05	6.64E-07	2.11E-06	5094	4148	4678	3563	3895
DPP3	0.80461	1.160019	1.306734	1.26915	0.790591	7.53E-05	1.22E-06	3.11E-12	3.65E-08	0.000887	5095	3533	1728	2987	5593
TCAIM	0.412734	0.513572	0.496848	0.541583	0.482453	7.63E-05	0.000142	4.56E-05	6.34E-05	1.36E-05	5097	4797	5009	4746	4372
AMT	-0.50895	-0.51583	-0.49164	-0.55166	-0.57144	7.67E-05	9.74E-05	2.89E-06	3.16E-05	1.54E-07	5099	4692	4268	4538	3358
RNASEH2A	0.933451	0.811883	1.185782	0.72584	0.984507	7.68E-05	0.003891	8.76E-06	0.00398	5.95E-06	5101	6004	4555	6154	4149
KRT23	1.28235	1.086744	0.818222	1.358859	1.316415	7.84E-05	1.77E-05	0.000223	9.79E-08	7.15E-08	5106	4179	5475	3163	3207
OAS2	-0.72065	-0.54765	-0.70968	-0.5518	-0.92527	7.87E-05	0.004686	0.000295	0.00022	1.81E-06	5107	6074	5567	5094	3860
RPS19BP1	0.420662	0.578464	0.591172	0.551112	0.519664	7.96E-05	1.62E-06	3.71E-07	4.86E-06	3.82E-06	5110	3601	3802	4013	4037
TGIF1	0.61889	0.515319	0.807744	0.855409	1.107002	7.99E-05	0.000422	8.81E-05	3.06E-06	7.94E-08	5111	5191	5194	3903	3226
BCL7C	0.448688	0.375531	0.424189	0.530784	0.589032	8.02E-05	0.001816	0.000337	3.67E-05	1.87E-06	5112	5719	5602	4579	3868
IRGQ	-0.3527	-0.23932	-0.39851	-0.22652	-0.36823	8.28E-05	0.004508	7.49E-07	0.006166	0.008941	5119	6062	3961	6321	6499
SLF2	-0.5545	-0.52742	-0.62826	-0.40181	-0.77595	8.30E-05	0.000402	0.000233	0.002316	1.06E-05	5120	5174	5497	5942	4288
MRPS16	0.482835	0.607344	0.766032	0.66359	0.820295	8.34E-05	7.82E-06	1.26E-09	4.60E-06	1.87E-10	5121	3966	2623	4001	2144
ZDHHC5	0.363354	0.803014	0.671124	0.729432	0.620295	8.34E-05	8.97E-10	8.74E-11	5.81E-09	2.81E-10	5122	2157	2192	2676	2219
NAT8B	-0.554	-0.43105	-0.80646	-0.62863	-0.61862	8.35E-05	0.001692	3.04E-08	9.00E-05	1.46E-05	5123	5689	3260	4829	4387
RPL23A	0.693922	1.137396	0.977907	1.334376	0.945274	8.40E-05	3.35E-08	5.82E-07	2.09E-10	9.12E-07	5126	2758	3898	2155	3714
ABCG1	-0.78031	-0.77239	-0.91035	-0.71885	-0.63674	8.48E-05	5.94E-05	4.22E-06	0.000178	0.000574	5128	4532	4361	5023	5443
CIZ1	0.43533	0.685994	0.397578	0.610165	0.430681	8.76E-05	2.69E-09	0.000253	3.14E-09	0.000315	5133	2326	5517	2594	5244
FHIP1B	0.469226	0.510022	0.622085	0.612732	0.542727	8.77E-05	2.10E-06	3.40E-07	2.82E-08	1.13E-05	5134	3674	3782	2945	4313
CDCA3	0.809211	0.69769	0.923883	0.618699	0.575726	8.81E-05	0.000227	6.41E-08	0.000588	0.00087	5136	4951	3424	5426	5590
ELP2	-0.44558	-0.50454	-0.50048	-0.47462	-0.47859	8.92E-05	6.55E-05	8.21E-06	0.000269	0.000201	5141	4564	4539	5180	5126
SAPCD2	0.85928	1.471896	1.032953	1.334845	0.810467	8.99E-05	1.88E-08	5.15E-06	4.21E-07	0.000329	5143	2650	4414	3454	5256
RNF6	0.656366	0.422091	0.48091	0.40242	0.677974	9.00E-05	0.009605	0.003208	0.009218	3.94E-05	5144	6409	6378	6493	4658
HSDL2	0.757605	0.690243	0.697605	0.827901	0.959551	9.03E-05	1.84E-05	7.66E-05	1.57E-05	1.07E-05	5146	4192	5143	4342	4293
ATP11A	0.972626	1.049789	1.080802	1.122334	1.462638	9.06E-05	4.17E-05	1.46E-05	7.51E-06	6.76E-10	5147	4428	4700	4127	2360
CTSH	-0.75982	-0.98312	-1.0997	-0.78509	-0.79902	9.09E-05	2.06E-06	4.66E-09	2.44E-05	0.000132	5148	3668	2862	4443	5012
TNRC6A	-0.38708	-0.53799	-0.49194	-0.51516	-0.43999	9.12E-05	2.55E-06	2.21E-06	1.94E-05	0.004052	5150	3717	4201	4392	6132
ATRAID	0.540055	0.546029	0.592973	0.449162	0.429508	9.17E-05	0.000304	2.58E-06	0.002243	0.000356	5153	5061	4241	5929	5281
UBA6	0.626397	0.635603	0.942779	0.760078	0.684556	9.17E-05	0.000312	3.11E-07	3.06E-05	6.52E-05	5154	5074	3769	4525	4798
AAGAB	0.619899	0.788335	0.542655	0.741797	0.637855	9.18E-05	5.10E-06	0.000343	9.24E-06	2.95E-05	5155	3863	5609	4188	4557
ADCK5	0.585004	0.938794	0.966068	0.86011	0.623458	9.25E-05	5.10E-07	1.93E-06	6.28E-05	0.000342	5157	3316	4181	4742	5269
DMBT1	1.902285	1.334068	3.082579	2.416055	2.651863	9.41E-05	9.72E-05	1.28E-08	8.70E-07	7.36E-09	5162	4691	3074	3605	2746
DCAF15	0.450831	0.574435	0.416606	0.398235	0.424592	9.50E-05	1.97E-06	0.000482	0.000868	3.71E-05	5164	3650	5729	5572	4628
SETD7	-0.54832	-0.85078	-0.60874	-0.62368	-0.96463	9.52E-05	3.67E-05	0.001807	1.46E-05	1.13E-05	5167	4389	6154	4317	4315
TYW5	-0.40796	-0.38542	-0.56178	-0.43369	-0.86886	9.87E-05	0.000128	3.51E-05	0.001894	9.03E-09	5175	4763	4940	5864	2792
EIF3F	0.571024	0.663571	0.448591	0.735357	0.751953	9.96E-05	2.75E-06	0.005109	5.22E-08	2.14E-05	5177	3735	6580	3043	4482
PRPF3	0.564402	0.565464	0.503235	0.564664	0.847793	0.0001	9.98E-05	0.000117	0.000351	2.13E-07	5179	4696	5264	5267	3426
RAD51AP1	0.48837	0.625258	0.42842	0.542191	0.412702	0.000103	2.82E-06	0.00154	6.70E-06	0.004794	5186	3746	6095	4092	6205
KIAA1549	0.513303	0.633937	0.541812	0.710491	0.35167	0.000103	1.22E-07	1.20E-06	2.89E-06	0.001292	5187	3043	4081	3895	5730
SLC27A3	-0.6686	-0.70475	-0.81347	-0.5036	-0.61171	0.000104	7.66E-06	1.53E-06	0.008932	0.001169	5189	3960	4133	6482	5693
NCR3	-0.41339	-0.32157	-0.62347	-0.33622	-0.46886	0.000105	0.001331	2.57E-08	0.00309	3.75E-05	5190	5620	3220	6065	4634
FOXA3	1.007759	1.455982	1.288321	1.573119	1.540739	0.000105	1.08E-06	5.48E-09	1.62E-07	9.53E-09	5192	3503	2891	3258	2805
EXOSC8	0.740991	0.968869	0.620915	0.662745	0.769329	0.000105	1.22E-06	0.000637	0.000571	2.84E-05	5193	3532	5805	5422	4546
SIPA1L3	0.672466	0.867558	1.024077	0.905082	0.614501	0.000107	5.43E-09	1.31E-09	2.75E-09	1.75E-06	5194	2456	2626	2568	3853
TMEM168	0.588011	0.641255	0.751572	0.642813	0.988338	0.000107	4.09E-05	1.74E-08	1.65E-05	4.96E-09	5195	4422	3137	4351	2681
BMI1	-0.54365	-0.36435	-0.5229	-0.47452	-0.55437	0.000107	0.008067	0.000212	0.000684	2.30E-06	5196	6332	5460	5486	3924
KIF2A	0.514418	0.539794	0.458431	0.496366	0.254814	0.000109	8.34E-06	6.48E-05	2.68E-05	0.006853	5199	3980	5104	4477	6365
PRKCI	0.399096	0.437559	0.578646	0.561231	0.36001	0.00011	6.44E-06	5.89E-06	4.64E-07	0.000868	5200	3920	4445	3483	5589
NEO1	0.614581	0.58631	0.793569	0.585189	0.70652	0.00011	0.00066	4.43E-06	4.50E-05	3.59E-05	5202	5360	4376	4644	4621
MLXIP	0.668379	1.07219	0.901483	1.145491	0.734788	0.000111	1.15E-07	5.25E-07	1.63E-09	0.000421	5203	3026	3877	2486	5351
GINS2	0.815402	0.957742	0.815515	0.809122	0.823464	0.000111	9.73E-06	8.57E-06	0.00037	0.001341	5204	4025	4548	5287	5743
ALDH9A1	0.3657	0.346847	0.378293	0.382505	0.335506	0.000111	0.005012	0.000129	0.00283	0.000122	5205	6097	5287	6024	4996
NOP14	0.378182	0.43558	0.596839	0.401029	0.714534	0.000111	0.000644	3.85E-07	6.47E-05	4.05E-09	5206	5347	3809	4752	2654
WDR26	-0.59514	-0.5076	-0.3636	-0.5039	-0.53933	0.000112	3.61E-05	0.009799	0.0007	2.55E-05	5209	4382	6837	5495	4521
MEP1A	1.342173	1.181393	1.588533	1.452655	1.701296	0.000113	0.000553	0.000101	3.33E-05	1.19E-06	5210	5300	5219	4556	3774
TELO2	0.519156	0.564865	0.526502	0.567944	0.625684	0.000115	4.00E-05	6.04E-07	6.66E-05	6.95E-06	5213	4416	3915	4756	4182
GLUL	-0.98959	-1.15166	-0.866	-0.74824	-0.7838	0.000115	1.67E-07	1.73E-05	0.001387	0.002884	5214	3106	4745	5751	6028
GPRC5C	0.813247	1.045442	1.30935	1.232552	1.271857	0.000115	0.00022	3.03E-07	9.12E-06	2.69E-09	5217	4941	3760	4182	2587
H4C13	0.936351	0.695286	1.374407	0.909175	1.505352	0.000116	0.000271	9.52E-08	3.82E-05	9.99E-09	5218	5019	3503	4597	2814
HHLA2	1.23735	1.078397	1.279153	0.917159	1.193825	0.000117	2.10E-05	4.75E-06	2.49E-05	8.25E-06	5221	4227	4395	4451	4233
RNPC3	-0.54836	-0.34995	-0.57024	-0.43711	-0.42262	0.000118	0.003382	5.67E-06	0.000174	0.003325	5222	5945	4435	5010	6062
PLEKHG5	-0.95951	-0.81021	-0.75821	-0.75045	-0.90224	0.00012	0.002103	0.002492	0.0048	9.31E-05	5226	5788	6292	6230	4909

CES3	0.595122	0.905297	1.211309	0.991283	0.672536	0.00012	2.50E-06	5.51E-09	4.95E-05	0.003364	5227	3710	2893	4673	6068
NECAB3	0.624986	1.137898	0.842717	1.327584	0.636329	0.00012	1.78E-06	5.20E-05	1.07E-09	4.33E-05	5228	3626	5046	2404	4679
PI4K2B	0.477419	0.470845	0.474427	0.626266	0.477262	0.000125	0.000903	0.00012	5.00E-06	9.05E-05	5234	5470	5272	4021	4895
IGF2BP2	0.693616	0.694982	0.505616	0.844422	0.511253	0.000125	0.000159	0.00105	6.16E-06	0.002719	5235	4827	5969	4071	6010
DDX51	0.496886	0.938371	0.727102	0.787829	0.464712	0.000125	2.17E-09	9.85E-08	2.07E-08	0.00474	5236	2290	3514	2894	6200
EPHA1	0.854604	0.773204	0.702359	0.621808	0.798237	0.000125	3.85E-05	2.23E-05	0.000679	1.05E-05	5237	4402	4806	5485	4287
FBXO15	0.496457	0.774729	0.621714	0.828192	0.650831	0.000125	1.00E-07	3.89E-07	7.36E-08	8.94E-07	5238	2996	3815	3101	3711
UBL4A	0.489831	0.724618	0.466859	0.641113	0.293711	0.000126	3.18E-06	6.46E-06	2.92E-05	0.008282	5239	3771	4468	4508	6460
CRELD2	-0.49754	-0.37815	-0.57212	-0.34199	-0.72244	0.000126	0.001493	4.19E-05	0.001033	4.24E-08	5240	5652	4983	5639	3090
VWA2	0.762084	1.077733	0.568446	1.035377	1.225393	0.000127	1.40E-05	0.001424	2.50E-05	1.61E-07	5242	4114	6076	4456	3369
MAP3K2	-0.61924	-0.55309	-0.40495	-0.56376	-0.64961	0.000128	1.13E-06	0.000544	8.90E-06	5.71E-07	5243	3512	5771	4170	3628
KIFAP3	-0.56267	-0.65338	-0.40733	-0.61752	-0.47862	0.000129	8.48E-07	0.00171	1.21E-05	0.00015	5245	3432	6121	4261	5045
CYB5A	0.830996	0.664859	0.977264	0.684754	1.111357	0.000129	2.15E-05	3.40E-08	0.000113	1.11E-11	5247	4234	3295	4886	1782
ATP2C1	0.308588	0.411748	0.515951	0.417126	0.51063	0.00013	2.96E-05	1.16E-09	2.22E-05	4.28E-07	5249	4326	2610	4415	3577
VPS8	-0.44444	-0.46567	-0.45699	-0.48892	-0.42337	0.000133	0.002092	9.16E-05	0.000912	9.30E-05	5254	5786	5202	5591	4906
IDH3A	0.476247	0.542057	0.608141	0.582906	0.288633	0.000135	2.48E-05	1.11E-07	1.75E-05	0.005344	5260	4271	3541	4364	6258
PHF6	0.478286	0.941611	0.682488	0.887425	0.438171	0.000136	8.64E-08	4.47E-06	4.94E-08	0.000859	5262	2961	4381	3037	5586
FAM102B	-0.6345	-0.75799	-0.59236	-0.65287	-0.74759	0.000136	2.22E-07	0.000198	0.000361	2.99E-06	5264	3171	5439	5278	3977
WDR89	-0.46768	-0.3629	-0.44026	-0.58957	-0.54616	0.000137	0.002336	0.003357	4.21E-06	1.23E-05	5266	5817	6400	3978	4340
ZNF823	0.460743	0.766477	0.709178	0.734222	0.365598	0.00014	5.52E-07	7.21E-07	6.55E-07	0.004508	5273	3330	3953	3560	6181
GNPTAB	-0.74824	-0.61675	-0.92082	-0.66973	-0.93478	0.000142	0.007188	2.24E-06	0.000214	6.50E-06	5277	6267	4207	5089	4167
TSEN2	0.847011	1.218853	0.76679	1.202486	1.046316	0.000142	2.27E-06	6.06E-06	1.52E-06	4.24E-07	5278	3693	4453	3737	3575
JUND	0.485089	0.719967	0.612027	0.613054	0.411759	0.000142	8.31E-07	1.30E-05	4.01E-06	0.003052	5280	3429	4671	3970	6040
MRPL30	0.444736	0.630978	0.563343	0.433991	0.651181	0.000142	5.80E-06	4.98E-08	0.000177	2.09E-08	5281	3895	3373	5015	2951
EXOC6B	-0.65423	-0.48481	-0.83676	-0.55223	-0.83117	0.000143	0.00169	2.25E-09	0.002678	1.80E-08	5283	5688	2729	5999	2927
IMP4	0.5471	0.692326	0.620177	0.649281	0.474036	0.000143	1.23E-05	3.18E-05	2.10E-05	0.00018	5285	4088	4911	4405	5099
PUM2	0.531644	0.694368	0.637662	0.646056	0.716017	0.000145	8.47E-06	1.61E-07	5.70E-05	3.96E-08	5289	3988	3615	4716	3071
PTP4A2	0.559285	0.47095	0.770015	0.501946	0.687582	0.000146	0.00029	2.98E-07	0.000122	1.03E-07	5290	5047	3753	4918	3285
NOP53	0.650977	0.82838	0.462115	0.801721	0.728061	0.000147	2.66E-06	0.006311	2.23E-06	0.000423	5292	3726	6665	3828	5353
PM20D2	0.498239	0.695729	0.740312	0.716825	0.425443	0.000147	3.69E-08	9.83E-06	5.25E-06	0.007425	5293	2780	4583	4037	6404
PRR22	-0.54321	-0.48619	-1.03799	-0.78371	-0.91326	0.000148	0.000817	2.55E-08	2.95E-05	1.96E-07	5294	5430	3218	4513	3412
ZNF282	0.360788	0.435791	0.544326	0.513147	0.674759	0.000154	9.56E-05	1.11E-06	4.07E-05	8.86E-08	5302	4687	4063	4611	3255
CAMK2G	0.54865	0.752134	0.511616	0.565441	0.633482	0.000155	3.39E-08	2.15E-06	1.03E-05	4.05E-08	5303	2763	4196	4221	3077
SMU1	0.431972	0.542585	0.779529	0.824872	0.290657	0.000155	0.000417	4.74E-07	2.16E-07	0.00774	5305	5189	3858	3312	6426
CCDC28A	-0.37248	-0.31976	-0.44243	-0.43197	-0.284	0.000156	0.005019	4.94E-05	0.000734	0.008399	5308	6098	5031	5516	6465
TCIM	-1.11792	-1.74677	-1.69973	-1.55043	-1.79064	0.00016	4.05E-08	3.07E-05	5.27E-07	3.93E-06	5310	2805	4902	3506	4045
MCMBP	0.487538	0.397183	0.575682	0.371188	0.347913	0.00016	0.00196	9.50E-07	0.009156	0.005373	5311	5755	4023	6490	6261
ZNF22	-0.50795	-0.5125	-0.31867	-0.56062	-0.31184	0.00016	0.00013	0.006626	4.58E-06	0.007844	5312	4770	6683	3999	6438
CNOT9	0.37116	0.517777	0.492348	0.474168	0.626012	0.00016	1.77E-06	4.43E-06	6.35E-06	2.49E-07	5313	3624	4379	4077	3459
MAOA	0.829319	1.321281	0.945498	0.851899	0.957514	0.000161	8.79E-07	6.40E-05	0.000319	0.000324	5314	3445	5097	5237	5251
B2M	-0.96647	-1.7986	-0.99694	-1.14179	-1.03119	0.000163	1.21E-07	0.001026	9.55E-06	0.001939	5317	3042	5961	4199	5900
DDX46	0.482086	0.530007	0.339431	0.514295	0.609257	0.000163	6.44E-05	0.003869	0.000109	4.29E-06	5319	4554	6461	4881	4067
DUSP22	-0.61371	-0.51775	-0.82484	-0.72888	-0.66389	0.000164	0.000257	2.18E-05	1.04E-05	0.000299	5320	5001	4797	4225	5234
POFUT2	-0.60735	-0.57337	-0.76753	-0.58027	-0.67653	0.000164	0.000368	1.85E-07	0.001362	2.86E-07	5321	5142	3645	5743	3491
SSRP1	0.585565	0.589476	0.867182	0.65481	0.7623	0.000165	0.004553	3.89E-05	0.001564	6.68E-06	5324	6064	4959	5792	4173
LARGE2	0.831872	0.934991	0.497645	0.759022	0.913366	0.000166	1.02E-05	0.003957	8.98E-05	1.40E-07	5325	4034	6476	4828	3343
TAF13	-0.47536	-0.47443	-0.33787	-0.53865	-0.67581	0.000166	1.56E-05	0.006672	0.000249	3.47E-07	5327	4141	6688	5143	3531
IL4R	-0.8077	-1.07018	-0.7222	-0.74806	-0.57979	0.000167	1.70E-07	6.09E-05	0.000284	0.007578	5328	3113	5086	5197	6412
PRKACB	-0.62177	-0.4867	-0.6619	-0.58982	-0.69849	0.000167	0.000878	2.89E-06	0.000211	1.10E-07	5330	5456	4267	5081	3299
TAF7	0.612645	0.756642	0.679967	0.483567	0.779388	0.000169	1.70E-05	2.49E-05	0.000339	3.27E-06	5332	4160	4843	5251	3998
MRPS14	0.451988	0.695498	0.388547	0.471269	0.625122	0.00017	1.42E-06	0.001246	0.000272	1.27E-06	5337	3566	6027	5183	3787
PIAS1	-0.52848	-0.46607	-0.55369	-0.66188	-0.55082	0.000174	0.001724	1.79E-05	1.04E-05	3.92E-05	5342	5698	4755	4222	4656
NDUFA2	0.584816	0.391087	0.67703	0.370345	0.53694	0.000175	0.002083	7.09E-06	0.004656	4.48E-05	5345	5785	4493	6217	4691
COG3	0.668646	1.151823	0.962123	0.99165	0.949007	0.000175	1.17E-06	6.02E-07	2.79E-06	1.55E-07	5346	3521	3914	3882	3361
MTX1	0.447613	0.675693	0.297967	0.439406	0.51403	0.000176	1.66E-07	0.007298	2.49E-05	3.78E-05	5349	3102	6722	4452	4642
LRP1	-0.96922	-1.27747	-0.97713	-1.13602	-1.22918	0.000177	1.42E-06	6.88E-05	0.000102	8.96E-06	5350	3568	5121	4862	4254
PCLAF	0.430182	0.305814	0.585664	0.281317	0.338542	0.000177	0.00153	1.35E-07	0.005515	0.000629	5351	5661	3579	6288	5479
DDX56	0.537897	0.554367	0.559245	0.383928	0.421882	0.000178	2.54E-05	2.92E-05	0.001012	0.00122	5352	4279	4881	5631	5706
STX16	0.487528	0.840239	0.620712	0.829467	0.643006	0.000178	9.37E-07	3.22E-05	6.02E-08	3.99E-05	5353	3466	4912	3068	4659
SCLT1	-0.44633	-0.43182	-0.59433	-0.49007	-0.42123	0.000178	7.96E-05	2.18E-05	0.000424	0.001505	5355	4629	4800	5326	5796
TRIM16L	0.527351	0.364951	0.539777	0.328108	0.353511	0.000178	0.000817	4.38E-06	0.000694	0.003645	5356	5431	4372	5491	6090
NDUFB5	0.562496	0.723208	0.559531	0.598249	0.82614	0.000179	5.46E-06	5.01E-05	9.02E-06	3.44E-07	5357	3884	5037	4176	3529
KALRN	0.411469	0.567152	0.448398	0.57641	0.87472	0.000181	1.71E-06	0.000381	5.29E-05	0.000505	5358	3617	5653	4687	5412
SLC7A2	-0.61922	-0.55698	-0.52103	-0.65196	-1.12898	0.000181	0.000353	0.000402	0.000138	1.31E-09	5359	5123	5668	4955	2470
RPS6KA1	0.694666	0.633258	1.02285	0.737698	0.903844	0.000183	3.52E-05	1.91E-08	3.29E-07	1.16E-07	5362	4371	3162	3398	3309
CDC45	0.452713	0.460593	0.528015	0.434059	0.61272	0.000184	0.001694	4.57E-05	0.001875	2.75E-06	5366	5691	5012	5862	3958
NUP37	0.639117	0.929756	0.975797	0.918258	0.994162	0.000185	0.000167	2.24E-05	6.70E-05	3.14E-06	5370	4852	4807	4761	3993
NCDN	-0.56454	-0.50587	-0.82023	-0.55077	-0.67396	0.000187	0.000315	1.49E-09	0.00059	1.51E-09	5372	5079	2643	5427	2490
TMEM161	0.475553	0.714966	0.562823	0.862903	0.597693	0.000188	4.44E-07	5.00E-05	6.24E-12	6.43E-06	5374	3288	5035	1699	4163
JAG1	-1.19757	-1.34963	-0.88238	-1.35894	-0.67817	0.00019	0.000486	0.002255	0.000479	0.006299	5377	5243	6253	5357	6329
CARS2	0.814943	1.014333	0.750741	1.006477	1.2947	0.000192	2.15E-06	0.000393	9.49E-07	7.75E-11	5380	3679	5664	3626	2014
AURKB	0.708629	0.654975	0.56942	0.659972	0.697033	0.000192	0.003556	0.005312	0.001974	9.21E-05	5381	5967	6602	5880	4902
EIF4A2	0.372571	0.431942	0.496318	0.392892	0.295639	0.000197	8.03E-05	3.94E-06	0.00078	0.000712	5389	4632	4342	5538	5529

UNC45A	-0.3867	-0.37794	-0.28131	-0.49254	-0.51968	0.000198	0.001288	0.007744	0.00019	4.06E-05	5391	5608	6742	5047	4661
RCC1	0.506567	0.757764	1.068463	0.810266	0.692467	0.000199	4.42E-06	1.07E-07	2.45E-07	7.56E-05	5393	3835	3530	3338	4844
RPS6KA3	0.46627	0.650559	0.399288	0.82813	0.546041	0.000201	7.31E-06	0.002613	8.80E-10	3.43E-05	5396	3945	6316	2372	4604
CDS1	0.75313	0.662207	1.057644	0.700297	0.946341	0.000202	4.78E-05	1.37E-11	4.26E-05	3.35E-08	5397	4473	1911	4625	3041
DNAJC21	0.404715	0.382252	0.260371	0.453309	0.213876	0.000202	0.000219	0.002449	0.000261	0.008119	5398	4940	6283	5158	6447
MCTP2	-0.78359	-0.58721	-0.62999	-0.64649	-0.58774	0.000204	0.001624	0.001567	0.000189	0.005397	5400	5672	6099	5045	6263
PRMT8	-0.64179	-0.58124	-1.20048	-0.67636	-0.65198	0.000206	0.000398	3.38E-09	0.00096	0.001112	5407	5172	2812	5612	5674
HK2	0.709372	0.727702	1.237213	0.935225	1.258317	0.000208	1.93E-05	1.04E-08	4.19E-06	4.46E-10	5408	4202	3022	3977	2290
GNE	0.802024	0.742936	0.606795	0.950673	0.498844	0.000208	0.002469	0.00589	0.000352	0.005791	5410	5838	6643	5269	6299
PIK3C2B	0.72064	0.632483	0.659456	0.562978	0.964139	0.000209	0.002081	0.000235	0.004263	4.77E-06	5411	5783	5500	6186	4084
PHACTR4	-0.42358	-0.55233	-0.38623	-0.58059	-0.35153	0.000211	7.33E-06	0.00106	1.28E-05	0.004092	5415	3947	5972	4274	6139
COL27A1	-1.11744	-1.22774	-1.60801	-1.38752	-1.33468	0.000211	0.000164	4.39E-07	0.000119	5.03E-06	5417	4844	3838	4909	4107
ARGLU1	0.872523	0.857226	0.880234	0.997059	1.55359	0.000214	4.67E-05	0.00012	1.61E-07	8.48E-09	5419	4458	5271	3257	2776
NT5DC2	-0.7351	-0.74747	-0.78265	-0.90016	-0.78997	0.000217	3.75E-05	3.90E-08	1.06E-05	3.46E-07	5420	4394	3326	4228	3530
MUS81	-0.47981	-0.29386	-0.43848	-0.40163	-0.72778	0.000219	0.007743	0.000317	0.000209	1.87E-07	5423	6310	5587	5078	3401
DIP2B	0.462413	0.403487	0.508778	0.485365	0.341821	0.000222	0.000819	3.90E-05	0.000199	0.003929	5425	5432	4961	5058	6117
RHEB	0.4583	0.463843	0.511822	0.44093	0.488905	0.000223	0.000977	0.000506	0.00176	0.000188	5426	5498	5749	5840	5109
NAA25	0.422684	0.656405	0.405969	0.621815	0.44991	0.000224	9.44E-10	0.000473	3.42E-09	7.21E-05	5428	2166	5721	2606	4831
H1-7	-0.44255	-0.55672	-0.41132	-0.55542	-0.79408	0.000224	2.54E-05	0.000358	2.56E-05	0.000312	5429	4280	5632	4460	5239
SAE1	0.468615	0.591459	0.522085	0.505483	0.81828	0.000226	5.01E-05	1.20E-05	0.000403	1.32E-08	5432	4483	4642	5310	2870
HMGN2	0.765576	0.537465	0.958113	0.687162	1.283031	0.000226	0.006562	5.47E-06	0.001631	1.12E-08	5434	6221	4426	5806	2842
DPH3	-0.50224	-0.49508	-0.47362	-0.73693	-0.48976	0.000228	0.007216	0.003952	4.53E-05	0.001445	5435	6271	6475	4647	5771
TMEM87B	0.46763	0.472942	0.673506	0.487619	0.601213	0.000228	0.000107	5.17E-08	0.000199	1.77E-05	5436	4717	3381	5059	4437
MTCH1	0.395245	0.430492	0.583691	0.382038	0.306573	0.000231	0.000103	8.80E-09	0.000631	0.001457	5438	4706	2990	5459	5775
DNHD1	-0.44164	-0.48438	-0.45414	-0.69955	-0.34479	0.000232	0.000107	4.79E-05	7.69E-08	0.007976	5439	4719	5028	3112	6440
MMP1	-1.9752	-2.48539	-2.21409	-2.98971	-4.05253	0.000232	3.99E-05	0.002208	7.65E-06	2.80E-09	5440	4414	6241	4133	2592
SMIM15	0.465843	0.522882	0.460971	0.624164	0.612595	0.000233	3.62E-06	6.46E-05	3.22E-08	5.07E-06	5441	3793	5103	2966	4110
SPDL1	-0.48329	-0.38738	-0.36607	-0.51556	-0.71755	0.000233	0.003796	0.003919	0.000102	9.15E-05	5442	5995	6473	4858	4900
FBXW11	-0.42949	-0.50618	-0.58713	-0.42816	-0.71284	0.000233	8.82E-06	7.31E-07	7.54E-05	8.15E-05	5443	4003	3959	4793	4857
IQGAP2	-0.63437	-0.81003	-0.69914	-0.98003	-0.55794	0.000234	4.43E-06	0.004899	1.02E-06	0.007806	5445	3836	6566	3646	6435
RND3	-0.94474	-0.77554	-0.59382	-1.01746	-1.13518	0.000237	0.003276	0.008992	0.000292	1.46E-05	5446	5933	6813	5208	4392
NUP93	0.53341	0.472838	0.371604	0.50158	0.773083	0.000237	0.000334	0.002693	0.000186	9.15E-05	5447	5105	6327	5041	4899
SS18L1	0.414527	0.628773	0.372248	0.591508	0.352756	0.000237	6.52E-08	0.000181	6.13E-06	0.003106	5449	2893	5405	4068	6046
AMY1A	-0.76471	-0.56262	-0.97318	-0.54498	-0.71795	0.000238	0.006999	4.14E-06	0.006586	7.55E-05	5450	6255	4357	6356	4842
VSIG10	0.529979	0.871147	0.946089	0.98181	1.167884	0.000238	2.93E-06	2.69E-07	1.41E-07	2.82E-11	5451	3753	3724	3231	1889
TESC	0.747499	0.97708	0.849149	0.925315	1.412691	0.000245	1.00E-05	6.15E-05	5.17E-06	3.45E-08	5456	4029	5087	4031	3045
HIBCH	0.42431	0.508753	0.583154	0.719941	0.472966	0.000247	0.000139	3.27E-06	1.51E-06	3.01E-05	5458	4792	4298	3735	4567
SPPL3	0.515739	0.521064	0.470509	0.533794	0.565625	0.000247	0.000117	0.000171	0.000533	3.50E-07	5459	4737	5380	5407	3534
PCSK7	0.592012	0.612901	0.610257	0.754853	0.491014	0.000247	0.00118	1.24E-05	3.42E-05	0.001631	5460	5579	4657	4560	5832
PWWP2A	-0.35025	-0.2999	-0.23882	-0.4635	-0.40689	0.000249	0.004311	0.009045	8.44E-07	0.005076	5463	6042	6815	3603	6232
ELOVL1	0.587984	0.481785	0.80671	0.784547	0.498299	0.000251	0.000693	5.25E-07	5.39E-06	0.000544	5465	5376	3879	4041	5427
COL17A1	0.957662	0.863235	1.632772	0.879192	1.212592	0.000254	0.001481	8.60E-08	4.94E-06	2.62E-06	5469	5650	3476	4018	3943
PXMP2	0.613413	0.526286	0.581873	0.456507	0.449094	0.000254	0.000667	9.54E-06	0.001635	0.003223	5471	5364	4576	5808	6059
LLPH	0.369251	0.345444	0.560055	0.424064	0.545108	0.000259	0.005426	7.58E-05	0.002233	1.31E-06	5474	6137	5141	5926	3795
C4orf46	-0.55395	-0.56522	-1.16331	-0.73017	-0.84872	0.000259	0.000394	1.56E-09	0.000129	1.32E-06	5475	5165	2646	4937	3797
DCTD	-0.29746	-0.41829	-0.53872	-0.47173	-0.34781	0.00026	2.48E-05	1.10E-06	4.37E-05	0.00209	5476	4272	4057	4634	5926
TTC7A	-0.56919	-0.60204	-0.70349	-0.72442	-0.65685	0.000264	8.30E-06	3.94E-06	1.59E-08	1.48E-05	5478	3979	4339	2843	4396
BCL10	0.638479	0.786844	1.010771	1.026497	0.830971	0.000264	0.000341	4.01E-06	2.24E-06	2.72E-06	5479	5108	4345	3830	3954
FAM98B	-0.30513	-0.48673	-0.47718	-0.45168	-0.66576	0.000265	1.14E-05	5.69E-06	0.000214	9.77E-11	5481	4065	4436	5088	2047
RNF181	0.420297	0.556855	0.470982	0.619776	0.774837	0.000265	3.05E-05	0.000807	6.07E-06	2.18E-06	5484	4339	5896	4065	3903
ZNF710	-0.3989	-0.41625	-0.61032	-0.31293	-0.40593	0.000266	0.000158	5.54E-08	0.008182	0.000521	5486	4826	3392	6443	5416
NCEH1	0.74056	0.875392	0.883454	1.012553	0.497338	0.00027	0.000243	3.43E-05	1.39E-05	0.001439	5493	4979	4934	4299	5765
FEN1	0.478517	0.337549	0.420215	0.342167	0.669653	0.000272	0.008448	0.002315	0.003495	7.78E-06	5495	6355	6265	6106	4216
FOXG1	-0.45851	-0.4551	-0.74084	-0.6169	-0.72027	0.000275	0.000156	1.33E-08	3.54E-06	2.21E-07	5499	4824	3080	3930	3438
C8orf82	0.451397	0.705481	0.883338	0.734602	0.735322	0.000275	1.09E-05	8.14E-08	1.50E-05	9.08E-07	5500	4049	3463	4323	3713
C2CD2L	-0.44554	-0.65676	-0.64062	-0.77826	-0.64618	0.000283	0.000484	2.63E-05	2.80E-05	7.33E-06	5505	5237	4859	4493	4199
LARP1B	0.429572	0.655952	0.547047	0.773341	0.368923	0.000283	0.000769	0.002171	9.98E-05	0.000489	5506	5408	6235	4856	5399
AMN	0.795988	1.008435	1.314477	0.806072	1.150334	0.000287	1.88E-06	7.10E-10	2.39E-05	1.09E-08	5511	3640	2524	4434	2835
TACC1	-0.81432	-0.71345	-1.51385	-0.91833	-1.37745	0.000289	0.000846	1.26E-07	0.000277	3.59E-07	5513	5445	3568	5192	3543
RCC2	0.401713	0.435674	0.646609	0.502496	1.023821	0.000291	0.003662	4.33E-06	0.001634	1.05E-09	5514	5975	4367	5807	2433
YIPF2	0.614806	0.610851	0.68252	0.60044	0.499544	0.000296	7.93E-05	1.26E-06	0.00025	4.77E-06	5517	4628	4095	5145	4085
DHFR	0.508448	0.686766	0.464375	0.75532	0.810076	0.000296	7.87E-07	0.000258	1.87E-07	2.25E-07	5519	3418	5526	3286	3445
HILPDA	0.668884	1.014446	0.876973	1.011918	0.947056	0.000297	0.000183	0.00224	0.00024	1.85E-05	5520	4879	6250	5125	4447
RRP1B	0.48138	0.664742	0.505066	0.487891	0.334459	0.000298	6.45E-07	0.000342	9.49E-05	0.002021	5521	3368	5608	4843	5914
EXOC4	0.499872	0.678293	0.422411	0.67301	0.535498	0.000299	1.23E-08	3.47E-05	1.18E-07	0.000723	5522	2593	4938	3198	5537
UBE2E3	-0.58042	-0.5486	-0.37737	-0.47477	-0.48336	0.000302	0.00033	0.000148	0.002344	0.000987	5523	5099	5327	5945	5628
CBX8	0.328123	0.427591	0.30331	0.328729	0.336642	0.000307	0.000189	0.004232	0.000901	0.00086	5527	4896	6501	5585	5587
SLC44A1	0.743179	0.893323	1.137811	0.936599	0.934495	0.000308	0.000624	5.97E-06	0.000183	5.20E-06	5528	5337	4447	5038	4119
REXO4	0.509752	0.707169	0.373761	0.637656	0.886172	0.000308	2.73E-06	0.001282	2.98E-05	2.67E-06	5529	3734	6039	4518	3948
METAP2	0.435205	0.686786	0.572852	0.471785	0.467153	0.00031	1.53E-05	3.00E-05	0.000889	0.001277	5533	4138	4889	5577	5727
FOXO3	0.556357	0.70966	0.582584	0.631043	0.545858	0.000313	0.000115	0.001882	0.00131	0.003957	5534	4735	6173	5727	6120
EXOSC5	0.640856	1.02522	1.056003	0.95929	1.066295	0.000317	1.64E-06	2.59E-07	1.14E-06	1.20E-09	5536	3607	3717	3675	2454
LYPLA2	0.342176	0.434244	0.494391	0.4476	0.578422	0.000319	2.03E-05	3.92E-06	4.55E-07	6.33E-10	5538	4217	4336	3479	2346

CD81	-0.78043	-0.94409	-0.60934	-0.66183	-1.10284	0.000321	1.16E-06	0.005578	0.000368	5.18E-06	5543	3519	6620	5284	4117
POLR2L	0.576134	0.550054	0.777863	0.630527	0.563453	0.000321	4.10E-05	9.83E-06	6.09E-05	2.65E-05	5544	4423	4584	4734	4531
TFF1	1.229881	1.144995	2.088706	1.27019	1.405923	0.000324	0.000142	1.73E-10	2.17E-05	1.71E-05	5547	4798	2302	4411	4431
H2AC7	0.862075	0.702695	1.180573	0.696997	1.085264	0.000325	0.005877	1.03E-06	0.004878	6.14E-05	5549	6175	4046	6234	4779
BARX2	-0.4575	-0.47367	-0.51589	-0.6341	-0.41125	0.00033	0.000386	0.000588	9.66E-06	0.000984	5551	5158	5788	4200	5625
MAGED2	-0.65081	-0.66055	-0.91489	-0.58672	-0.67027	0.00033	0.001755	1.11E-07	0.005473	6.29E-05	5552	5707	3540	6283	4786
FAM207A	0.581608	1.190352	0.893702	0.847535	0.944217	0.000332	8.57E-09	5.81E-07	1.92E-05	1.61E-05	5553	2529	3896	4387	4415
GYS1	-0.44169	-0.4246	-0.70573	-0.50884	-0.61743	0.000333	0.001257	1.79E-06	0.000127	1.91E-05	5554	5602	4170	4926	4456
C6orf223	0.959633	0.923904	1.23694	1.218303	0.967277	0.000335	0.00015	3.03E-06	2.02E-06	2.82E-06	5556	4814	4284	3807	3960
TRIM14	0.48982	0.676791	0.666918	0.754056	0.513007	0.000339	2.74E-07	4.28E-07	9.33E-08	3.19E-05	5561	3200	3835	3154	4583
ZDHHC12	0.517379	0.502978	0.375073	0.547446	0.580044	0.000345	0.000276	0.003406	9.00E-05	1.54E-05	5566	5030	6407	4830	4403
GABPB1	-0.33642	-0.3307	-0.60089	-0.57467	-0.50155	0.000347	0.000308	7.55E-06	4.07E-07	6.57E-05	5569	5071	4511	3450	4800
PDF	0.572915	0.588702	0.540902	0.481067	0.585162	0.000352	0.000815	0.000642	0.003684	0.000246	5571	5428	5810	6122	5181
VPS13A	0.440924	0.580941	0.780537	0.556643	0.489257	0.000352	1.01E-05	3.22E-10	7.27E-05	0.002345	5572	4031	2397	4781	5964
LRG1	0.761929	1.205268	0.899643	1.178516	1.01821	0.000355	5.32E-08	3.87E-06	9.70E-07	2.16E-08	5576	2857	4330	3633	2955
PTPRJ	-0.57923	-0.85236	-0.51042	-0.72853	-0.39721	0.000356	2.03E-06	0.000807	0.000146	0.001384	5577	3660	5895	4969	5753
MTA1	0.455751	0.586978	0.472307	0.511094	0.8183	0.000357	0.000123	0.002084	0.000252	3.60E-08	5579	4751	6211	5147	3055
ALDH1B1	0.586074	0.995219	0.800257	0.840134	0.848428	0.000357	6.49E-09	4.65E-06	3.83E-06	5.71E-07	5580	2486	4390	3953	3627
EXOSC7	0.598846	0.776609	0.820614	0.645759	0.534858	0.000358	0.00027	6.40E-05	0.000867	0.001145	5582	5018	5096	5569	5686
EIF3L	0.530687	0.515751	0.498416	0.675596	0.699845	0.000359	7.88E-05	0.000459	1.07E-05	4.49E-05	5583	4625	5711	4236	4695
CRLF3	-0.35673	-0.4199	-0.61563	-0.3879	-0.94812	0.00037	0.000735	7.95E-07	0.000404	9.55E-11	5592	5390	3979	5313	2044
BROX	0.599798	0.620744	0.947073	0.748515	1.226155	0.00037	0.000275	2.15E-08	1.78E-06	6.19E-13	5593	5028	3180	3769	1429
RPAP3	0.458822	0.609117	0.429465	0.663871	0.368663	0.000373	3.77E-06	9.09E-06	1.16E-07	0.00039	5595	3808	4563	3192	5312
MSRB1	0.543783	0.78011	0.647012	0.956118	0.922984	0.000376	4.36E-08	0.000162	2.56E-10	2.65E-07	5596	2821	5355	2181	3475
ELMOD2	-0.42633	-0.43487	-0.32672	-0.53567	-0.73021	0.000376	0.000701	0.006737	1.54E-05	2.23E-06	5597	5377	6690	4333	3910
MT2A	-1.61068	-1.85308	-1.59563	-1.93504	-2.07434	0.000378	0.000109	0.000865	3.04E-05	1.39E-05	5598	4725	5922	4522	4378
CORO1C	-0.64619	-0.69671	-0.46326	-0.51872	-0.64024	0.000379	6.10E-05	0.006008	0.000875	0.000116	5599	4540	6658	5574	4978
GUCD1	0.664918	0.734351	0.816687	0.795352	0.65293	0.00038	0.000669	5.82E-05	0.000137	6.38E-05	5601	5367	5072	4954	4789
LDLRAP1	0.462207	0.373119	0.314062	0.404728	0.544215	0.000381	0.001732	0.001875	0.003994	0.000607	5602	5703	6170	6157	5468
ADAT2	0.439205	0.828903	0.513781	0.574311	0.600942	0.000382	2.15E-09	8.45E-05	2.98E-05	9.64E-05	5603	2288	5180	4517	4916
DOP1B	0.550626	0.781375	0.794211	0.945262	0.672671	0.000387	8.62E-07	2.07E-07	3.94E-07	4.06E-08	5609	3439	3665	3444	3078
FXR2	-0.42685	-0.44597	-0.29364	-0.5043	-0.45193	0.000403	0.000147	0.004893	0.000192	9.62E-06	5622	4807	6565	5048	4267
TSG101	0.322635	0.447925	0.640673	0.514529	0.710512	0.000404	3.06E-05	1.89E-07	2.51E-06	8.68E-06	5624	4341	3650	3854	4247
URM1	0.39428	0.391145	0.532211	0.358219	0.484278	0.000406	0.000101	4.22E-06	0.00047	9.03E-05	5625	4701	4360	5349	4894
DENND1A	0.501338	0.581728	0.788327	0.579144	0.698968	0.000413	1.77E-06	3.56E-10	1.20E-06	1.61E-06	5631	3622	2415	3690	3838
SEMA3C	0.854825	1.000688	1.590733	1.158558	1.559797	0.000416	4.66E-05	4.59E-10	4.60E-06	2.17E-12	5633	4456	2453	4000	1567
TC2N	0.837482	0.959066	1.598128	1.136163	0.816373	0.000417	0.000249	1.39E-09	3.81E-05	0.000407	5634	4988	2634	4596	5336
DCTN6	-0.51074	-0.56174	-0.41736	-0.5305	-0.47246	0.000422	0.000105	0.000575	0.000137	0.000408	5642	4711	5782	4953	5338
PTPN2	-0.55713	-0.54349	-0.58141	-0.70244	-0.42544	0.000423	0.00124	9.43E-05	7.77E-06	0.006352	5643	5597	5206	4138	6331
CPNE7	0.836359	1.326371	0.954602	1.273107	1.230379	0.000425	6.09E-08	0.000244	1.57E-06	1.82E-07	5647	2886	5508	3743	3396
NELFB	0.620349	0.933258	0.967414	0.787085	0.897087	0.000425	3.95E-06	5.57E-12	2.95E-05	1.49E-07	5648	3817	1798	4512	3352
ZFP36L2	0.715626	0.770968	1.144405	1.19086	1.195859	0.000431	1.83E-06	7.29E-08	1.39E-10	7.67E-13	5654	3636	3447	2101	1452
CPSF3	0.40282	0.408912	0.371053	0.52226	0.595055	0.000434	0.00024	4.01E-05	8.85E-06	3.77E-05	5655	4972	4971	4167	4640
INSIG1	0.853677	0.790532	1.007429	0.7611	1.050188	0.000434	0.00026	1.26E-05	0.000472	3.54E-08	5656	5007	4666	5350	3050
DCBLD2	-0.503	-0.47371	-0.52023	-0.44736	-0.66708	0.000437	0.004675	0.000389	0.006343	3.07E-06	5657	6073	5660	6341	3986
MTDH	0.57455	0.53167	0.774132	0.544806	0.434203	0.000443	0.001031	6.91E-05	0.002769	0.006585	5659	5520	5122	6011	6349
TOX	-0.75546	-0.69413	-0.49842	-0.50807	-0.7818	0.000449	4.25E-05	0.004516	0.008841	7.99E-08	5661	4438	6531	6474	3228
ABCB9	-0.46767	-0.42826	-0.68172	-0.48858	-0.66271	0.000452	0.002322	4.33E-09	0.000815	1.12E-05	5662	5814	2851	5549	4309
KLHDC4	0.719013	0.718988	0.656257	0.852386	0.744791	0.000454	0.000322	0.000721	5.55E-06	0.000125	5664	5089	5859	4050	4998
AHCYL2	0.931304	0.961076	1.352155	1.043343	0.858513	0.000458	8.45E-05	3.54E-07	1.49E-07	1.14E-05	5670	4646	3792	3241	4320
CKB	1.055857	1.773638	1.543041	1.691263	1.412335	0.000463	3.01E-07	4.65E-06	1.06E-07	5.26E-06	5674	3221	4391	3177	4123
NR2C2AP	0.40686	0.722649	0.635438	0.736897	0.817103	0.000465	1.19E-07	5.35E-06	2.90E-07	5.38E-08	5675	3037	4421	3367	3144
RASAL2	-0.61444	-0.60609	-0.68692	-0.57279	-0.55339	0.000488	0.000255	7.06E-10	0.000458	0.001005	5686	4998	2523	5342	5635
PDP1	0.598348	0.824614	0.708647	0.80243	0.488719	0.000497	3.63E-06	6.66E-05	5.01E-06	0.001123	5689	3794	5112	4022	5679
EPB41L5	0.477025	0.573641	0.640593	0.501687	0.644533	0.0005	3.19E-05	9.42E-07	9.66E-05	1.37E-06	5692	4347	4021	4848	3807
NUAK2	0.429247	0.838327	0.800363	0.781564	1.1148	0.000503	2.37E-08	4.87E-06	4.51E-07	6.83E-08	5697	2692	4405	3477	3188
ZNF627	-0.50757	-0.79656	-0.82121	-0.73899	-0.43383	0.000503	1.40E-05	2.91E-06	9.62E-05	0.006503	5698	4113	4270	4845	6346
GTF3C3	0.319378	0.626673	0.444764	0.556173	0.560958	0.000504	8.32E-09	6.75E-06	1.29E-08	2.58E-07	5703	2522	4478	2809	3465
TRIM5	-0.58061	-0.47298	-0.53978	-0.46165	-0.5033	0.000507	0.001026	0.00032	0.002562	0.000844	5707	5516	5589	5974	5580
PTPRO	1.011194	1.578249	0.900636	1.38537	1.115924	0.000518	1.87E-08	0.000283	2.40E-06	1.68E-05	5712	2649	5556	3844	4425
NOM1	0.372216	0.560082	0.407975	0.588668	0.384693	0.000521	7.31E-07	0.00035	2.24E-07	0.001219	5713	3400	5617	3317	5704
BLOC1S2	-0.50097	-0.65219	-0.92713	-0.76711	-0.83556	0.000523	1.18E-05	2.57E-12	2.66E-06	6.65E-08	5715	4074	1694	3865	3182
IRAK2	-0.55069	-0.45227	-0.39498	-0.52328	-0.48936	0.000523	0.005433	0.00535	0.001395	0.006747	5716	6141	6604	5755	6361
REC8	-0.44713	-0.46916	-0.72403	-0.71383	-0.91914	0.000524	0.007288	3.10E-05	0.000193	1.82E-06	5717	6275	4905	5051	3861
SOD2	-1.07046	-0.93421	-0.97622	-0.95647	-1.24022	0.000526	0.00749	0.001323	0.004764	0.000107	5718	6289	6047	6224	4957
CAMTA1	0.356875	0.322302	0.3713	0.466583	0.256215	0.000529	0.000752	9.65E-05	4.56E-06	0.002683	5719	5395	5211	3997	6004
VBP1	0.453055	0.621841	0.660265	0.615905	0.760111	0.00054	0.000344	3.71E-05	0.000222	2.26E-05	5729	5114	4948	5099	4494
SIGIRR	0.43294	0.535711	0.505292	0.482588	0.572711	0.000542	7.88E-05	2.42E-05	0.000359	9.63E-08	5730	4626	4833	5275	3267
SMAD2	-0.56869	-1.01486	-0.48482	-0.85323	-0.49899	0.000543	4.16E-10	0.00083	3.35E-07	0.000204	5731	2045	5906	3406	5129
HERPUD2	-0.41912	-0.44111	-0.41595	-0.45743	-0.51723	0.000544	0.000537	0.000742	0.001371	1.43E-05	5732	5284	5864	5745	4384
CCHCR1	0.503563	0.60634	0.509447	0.390078	0.521778	0.000548	5.40E-05	0.000575	0.001294	0.003657	5733	4510	5781	5718	6092
CMC2	0.450323	0.453326	0.359793	0.525698	0.793269	0.000554	0.000358	0.003285	3.83E-05	6.79E-05	5738	5128	6389	4598	4811
EHMT2	0.51489	0.640585	0.59703	0.461177	0.745959	0.000555	1.67E-05	4.78E-05	0.000108	4.01E-08	5739	4156	5026	4879	3074

GALNT5	0.854885	1.279708	1.290846	1.655321	1.02978	0.000556	1.75E-06	9.44E-08	8.74E-11	1.38E-05	5740	3620	3500	2050	4374
GRINA	0.64037	0.555244	0.70141	0.816736	0.440869	0.000559	0.001761	0.000141	2.03E-05	0.008239	5744	5708	5313	4402	6457
PEDS1	0.48699	0.562388	0.581914	0.687793	0.393646	0.000559	0.000187	4.14E-05	2.85E-06	0.000977	5745	4895	4981	3889	5621
PIGO	0.492104	0.758966	0.393147	0.670524	0.40692	0.000562	1.65E-05	0.004279	0.000142	0.001797	5746	4150	6511	4963	5868
RIDA	0.699606	0.940011	1.028767	0.96803	0.593507	0.000563	8.86E-05	3.91E-08	9.90E-05	0.001098	5747	4666	3327	4854	5670
FANCA	0.575421	0.552736	0.455254	0.681293	0.729473	0.000566	0.004035	0.005153	0.000271	0.000105	5748	6021	6587	5181	4952
GDI2	0.549993	0.340965	0.78964	0.547045	0.770338	0.000566	0.006603	8.13E-07	0.001328	1.17E-06	5749	6225	3981	5733	3769
PLEKHA6	0.753268	1.08349	1.244155	1.179243	0.680371	0.000572	6.61E-05	6.48E-08	2.45E-05	0.000654	5754	4567	3428	4444	5494
PARP12	0.602711	0.664626	0.592749	0.615552	0.417701	0.000579	0.000253	0.000349	2.95E-05	0.007142	5759	4995	5614	4510	6380
NDUFAF2	0.586443	0.923106	0.843409	0.894885	0.736461	0.000589	1.72E-06	5.24E-06	8.71E-07	2.48E-06	5762	3618	4418	3606	3936
RAB20	0.51976	0.700059	0.58197	0.464272	0.776486	0.0006	7.17E-05	2.86E-05	0.000639	0.000101	5765	4594	4873	5464	4934
USP15	-0.3844	-0.41433	-0.6891	-0.37426	-0.50654	0.000605	0.000548	3.17E-08	0.009275	0.000324	5768	5296	3269	6497	5252
CDKN2AIP	-0.29566	-0.26677	-0.38189	-0.37413	-0.39469	0.000616	0.006495	4.23E-05	4.02E-05	4.64E-05	5776	6218	4987	4606	4701
AAR2	0.552222	0.759124	0.502772	0.772883	0.566922	0.000618	2.24E-05	0.000756	3.96E-06	0.001384	5777	4247	5868	3967	5751
MTA3	0.531922	0.67095	0.494363	0.600372	0.456596	0.000621	5.15E-06	2.49E-05	6.33E-06	6.08E-05	5778	3867	4845	4076	4773
POGZ	-0.37838	-0.55338	-0.35605	-0.49595	-0.30386	0.000631	0.000135	0.004169	0.001045	0.003071	5781	4779	6494	5645	6044
ATG4B	0.412322	0.407724	0.556445	0.64258	0.464239	0.000644	0.00136	7.09E-06	5.14E-08	0.000705	5791	5624	4494	3041	5525
H2AW	0.478341	0.681786	0.890806	0.645946	0.567983	0.000644	8.98E-05	3.11E-09	0.000156	0.000812	5792	4667	2795	4985	5566
LYAR	0.669986	0.707022	0.844572	0.464071	0.87656	0.000648	0.000177	1.88E-08	0.006326	4.46E-06	5793	4871	3159	6338	4073
TSR1	0.365592	0.592745	0.502175	0.507979	0.407512	0.00065	1.28E-06	9.27E-06	1.17E-05	0.001651	5795	3544	4570	4257	5834
PSAT1	0.822577	1.311984	0.820264	1.088438	0.756502	0.000656	7.89E-08	7.39E-05	2.82E-07	0.001292	5798	2941	5129	3366	5731
ZYX	-0.63742	-0.97692	-0.57319	-0.8675	-0.6297	0.000659	3.97E-07	0.00295	1.38E-05	3.89E-05	5802	3273	6354	4298	4653
HELLS	0.479489	0.464845	0.636963	0.425647	0.486189	0.000676	0.000443	4.78E-06	0.003292	0.000654	5808	5208	4399	6086	5493
STXBP6	0.688483	0.786297	1.024588	0.76625	0.912471	0.000678	0.002014	3.01E-08	0.002297	2.93E-05	5810	5771	3258	5937	4550
ARRDC4	-0.59259	-0.69403	-0.54778	-0.55909	-0.68828	0.000682	1.97E-05	0.002113	0.002058	1.54E-05	5811	4209	6221	5891	4404
GSR	0.647388	0.855665	0.997878	1.00882	0.835716	0.000683	0.000557	1.39E-08	1.94E-05	7.55E-06	5812	5303	3089	4391	4212
INAVA	0.605445	0.591276	0.508503	0.610417	0.975685	0.0007	0.000306	0.001745	0.001534	3.94E-06	5817	5069	6137	5784	4047
ZNF267	-0.36439	-0.49628	-0.61004	-0.54014	-0.58033	0.000701	0.000164	9.81E-08	8.99E-06	0.000202	5818	4848	3512	4174	5127
SKA3	0.58186	0.780903	0.614336	0.614412	0.845083	0.000719	2.99E-05	6.38E-05	3.56E-05	8.82E-06	5823	4332	5095	4571	4251
PKD1	-0.39037	-0.35804	-0.32263	-0.39292	-0.32273	0.000722	0.001235	0.001348	0.000455	0.001959	5824	5593	6054	5340	5904
ZNHIT3	0.314333	0.435725	0.414437	0.490924	0.252188	0.000724	3.22E-05	1.71E-05	1.84E-06	0.007844	5827	4350	4742	3782	6437
TCF7	0.589486	0.943716	0.934808	1.116089	0.669387	0.000732	0.000429	1.25E-05	3.76E-06	0.00355	5831	5196	4662	3943	6081
LPCAT4	0.555563	0.466252	0.597841	0.429427	0.513709	0.000734	0.001066	0.000992	0.002866	0.001598	5832	5536	5952	6032	5814
IFI44	-0.6133	-0.69016	-0.78635	-0.68317	-1.32882	0.000737	0.000915	0.000125	3.48E-05	3.58E-07	5833	5475	5280	4564	3542
MED7	-0.40466	-0.2774	-0.44788	-0.48526	-0.53425	0.000753	0.006149	9.94E-06	2.85E-06	6.46E-05	5837	6191	4588	3890	4796
TIMM23	0.374803	0.484947	0.551427	0.331526	0.57201	0.00076	5.03E-06	1.39E-08	0.001239	1.25E-07	5844	3862	3091	5703	3322
PCNP	0.400717	0.440574	0.562199	0.459015	0.485087	0.000763	0.000924	1.07E-05	0.000281	4.59E-05	5846	5481	4615	5195	4700
UAP1	0.529713	0.441297	0.498442	0.582271	0.524731	0.000781	0.00225	0.00229	0.000881	0.004248	5849	5804	6260	5576	6154
MLKL	-0.45498	-0.56219	-0.96898	-0.61357	-0.89327	0.000781	0.000274	3.05E-07	0.00035	6.72E-08	5850	5023	3763	5265	3185
MRPL49	0.548646	0.734449	0.958182	0.817254	0.527715	0.000787	1.88E-06	5.00E-08	1.81E-07	0.000104	5852	3642	3375	3279	4947
PYCR3	0.770984	0.914563	0.806901	1.073297	0.523697	0.000803	0.000329	0.000164	6.08E-06	0.007159	5855	5097	5360	4066	6383
CLINT1	0.513224	0.399171	0.658732	0.45081	0.380724	0.00082	0.000453	1.22E-05	0.000269	0.002921	5860	5215	4651	5179	6030
P2RX4	-0.54303	-0.53413	-0.92285	-0.48359	-0.76925	0.000832	0.001788	1.24E-09	0.006082	2.87E-07	5861	5712	2621	6313	3493
LGR5	0.828233	1.086324	0.828443	1.074155	0.952486	0.000842	4.79E-07	0.001918	3.03E-05	0.001667	5863	3305	6180	4521	5840
MRPL47	0.386873	0.571863	0.495693	0.388436	0.393726	0.000843	6.70E-05	5.23E-05	0.002061	0.001626	5864	4574	5047	5893	5827
MOGAT3	1.065577	1.220315	1.937031	1.075779	1.549943	0.000843	0.001238	6.92E-12	0.00309	1.23E-05	5866	5595	1826	6066	4339
LUC7L3	0.540424	0.593711	0.407831	0.61335	0.610163	0.000843	0.000438	0.008065	0.000198	0.000179	5867	5204	6767	5054	5096
EXOSC3	0.439547	0.924439	0.519788	0.902993	0.505067	0.000847	5.62E-06	0.001145	1.04E-05	3.83E-05	5869	3890	5997	4224	4646
MAP3K1	-0.38956	-0.50273	-0.80095	-0.61923	-1.05165	0.000855	6.95E-05	1.74E-09	7.18E-06	4.34E-09	5871	4583	2667	4115	2671
NDUFAF4	0.539003	0.921581	0.910611	0.969099	0.684296	0.000856	6.96E-09	8.07E-09	1.95E-08	9.63E-06	5872	2493	2969	2881	4268
RTEL1	0.740634	1.167545	1.076167	1.287809	0.819327	0.000867	4.13E-05	2.75E-05	4.95E-06	0.000114	5875	4424	4868	4019	4975
MAP4	-0.56891	-0.628	-0.41522	-0.48657	-0.41367	0.00087	0.000149	0.007582	0.003114	0.005146	5876	4812	6739	6069	6240
DUOX2	1.302961	1.687914	2.582311	1.742574	2.048203	0.00087	5.45E-05	1.70E-08	3.75E-05	4.32E-06	5877	4513	3132	4591	4068
RNF5	0.363388	0.521054	0.676175	0.4421	0.38912	0.000885	0.000106	1.25E-08	0.001435	0.000956	5882	4715	3070	5762	5613
MRPL16	0.475926	0.61285	0.743359	0.854916	0.519029	0.000886	3.60E-05	3.00E-05	2.12E-08	0.000401	5883	4378	4890	2899	5327
CLIC5	0.62224	0.91268	0.878314	0.963997	0.657264	0.000889	4.56E-06	1.79E-05	2.01E-06	0.000194	5884	3842	4753	3805	5117
TEX261	0.426758	0.353871	0.47248	0.396252	0.439907	0.000897	0.008058	0.000109	0.002247	4.31E-05	5888	6331	5242	5931	4677
CDKN3	0.540203	0.880076	0.873147	0.798143	0.556957	0.000899	0.000164	4.70E-06	0.000224	0.000891	5889	4845	4393	5103	5594
FBXO38	-0.38411	-0.33284	-0.50261	-0.54712	-0.48291	0.000904	0.001492	8.72E-06	6.67E-06	2.93E-06	5891	5651	4553	4091	3969
NUCB1	-0.47115	-0.74906	-0.55202	-0.51382	-0.5714	0.000904	1.11E-05	0.000834	0.000578	0.000277	5892	4054	5909	5423	5218
CCDC9B	-0.613	-0.53638	-1.04652	-0.69428	-1.04064	0.000913	0.001506	3.13E-09	0.000262	9.96E-09	5895	5656	2797	5162	2812
CRTAP	-0.45875	-0.39329	-0.376	-0.35392	-0.65873	0.000939	0.007934	0.0046	0.009237	1.10E-05	5908	6327	6540	6496	4303
WIPI1	-0.52344	-0.4064	-0.51545	-0.41102	-0.51647	0.00095	0.004559	0.000117	0.006744	0.000221	5913	6067	5263	6369	5143
GRN	-0.69153	-1.16363	-0.66447	-0.78781	-0.92804	0.000951	3.79E-07	0.00018	0.000102	1.02E-06	5914	3264	5404	4859	3741
DGKZ	0.348627	0.678118	0.315591	0.689746	0.480314	0.00096	1.67E-06	0.008506	5.99E-07	0.000153	5920	3612	6790	3535	5049
DHX34	0.363378	0.497464	0.360118	0.382934	0.320824	0.000968	4.16E-05	0.004684	0.00029	0.004685	5922	4427	6548	5207	6193
NXT1	0.482937	0.649416	0.372916	0.733532	0.536012	0.00097	1.90E-06	0.002498	9.95E-08	2.64E-05	5923	3646	6294	3165	4529
YARS1	0.569203	0.596653	0.716044	0.707079	0.606928	0.000974	0.000151	0.000123	4.34E-06	6.80E-05	5924	4815	5274	3986	4813
MEA1	0.395213	0.485514	0.635535	0.446959	0.445655	0.00098	0.000152	0.000182	0.001823	0.002309	5927	4819	5409	5855	5961
RING1	-0.44936	-0.59452	-0.59263	-0.65161	-0.47903	0.000981	7.64E-05	4.42E-05	2.71E-05	0.000255	5929	4612	5004	4482	5191
FADS1	-0.85865	-0.90671	-0.49846	-1.0522	-0.89019	0.000992	1.54E-05	0.007104	2.58E-06	2.96E-06	5932	4140	6710	3858	3974
ISG15	-0.88635	-0.96895	-0.88849	-0.7501	-1.14575	0.001006	0.001042	0.000591	0.00114	3.76E-05	5934	5526	5789	5672	4638
TLE3	-0.38379	-0.41047	-0.57977	-0.57807	-0.59635	0.001012	0.000529	2.33E-07	7.54E-06	2.49E-10	5937	5278	3691	4129	2196

TSPAN14	-0.42947	-0.57713	-0.5722	-0.42392	-0.40211	0.001016	6.70E-05	2.02E-05	0.008333	0.007617	5939	4573	4781	6451	6414
MANBAL	0.416821	0.347974	0.487135	0.461631	0.524103	0.001023	0.001188	0.000315	0.000136	0.000118	5941	5582	5584	4952	4982
FKBP11	-0.5568	-0.54409	-1.07464	-0.79972	-1.18246	0.001029	0.000641	1.79E-06	0.001523	4.90E-06	5942	5344	4168	5779	4097
TPD52L1	0.712747	1.041646	1.018	0.728995	0.796751	0.001034	5.53E-06	4.72E-06	0.00036	1.34E-05	5943	3886	4394	5276	4369
RUSF1	0.443503	0.453663	0.589271	0.727313	0.527976	0.001037	0.006979	8.05E-05	8.96E-05	0.000111	5945	6253	5163	4827	4963
AIFM2	0.408312	0.419246	0.476854	0.431653	0.360152	0.001044	0.00081	1.43E-05	0.001297	0.003843	5949	5426	4692	5720	6109
H2AJ	0.82235	0.629898	0.633788	0.997819	1.064531	0.001046	0.005019	0.000506	5.66E-05	8.24E-07	5951	6099	5751	4714	3696
WWP1	0.421433	0.413088	0.843702	0.701416	0.56123	0.001064	0.009477	9.18E-05	0.000908	0.002709	5955	6405	5203	5589	6007
SLC25A46	-0.53368	-0.85265	-0.71594	-0.9554	-0.5583	0.001072	0.00013	0.000227	2.66E-05	0.000393	5958	4766	5483	4473	5316
ARRB1	-0.61355	-0.6753	-0.69463	-0.70636	-0.93295	0.001085	0.001274	4.07E-06	0.000398	8.41E-07	5960	5605	4348	5305	3697
ANP32A	0.454973	0.537639	0.426765	0.480079	0.527958	0.0011	0.000212	0.003617	0.000122	0.000135	5964	4926	6437	4919	5019
USP47	-0.37792	-0.45716	-0.4933	-0.43603	-0.46301	0.00111	0.000804	0.000104	0.00157	3.47E-05	5970	5422	5226	5793	4610
WFDC2	0.835867	1.031875	1.05965	1.282312	2.037975	0.001111	0.000103	2.75E-06	9.27E-05	2.68E-06	5971	4707	4257	4836	3951
ATXN7	-0.34513	-0.43801	-0.32124	-0.47263	-0.43019	0.001122	0.000558	0.003161	0.000311	5.10E-05	5974	5305	6375	5232	4730
PPAN	0.553303	1.092101	0.663315	0.674278	0.486265	0.001123	1.08E-07	4.23E-05	0.000167	0.004437	5975	3011	4988	5001	6175
SERPINB8	-0.41273	-0.41282	-0.63086	-0.53867	-0.50108	0.001124	0.006374	0.002309	0.003961	0.005283	5976	6212	6263	6152	6256
EBAG9	0.517663	0.760165	0.557146	0.764976	0.441513	0.001126	7.79E-07	1.09E-06	5.39E-07	0.00011	5978	3414	4056	3513	4961
CELSR3	0.576391	1.036152	0.704987	0.788959	0.587254	0.00114	2.12E-05	0.000494	0.000938	0.001857	5980	4230	5741	5601	5883
GPAA1	0.533184	0.600525	0.539392	0.625409	0.459683	0.001144	0.000148	0.000289	5.52E-05	0.000163	5981	4810	5561	4707	5066
HSF1	0.554882	1.159205	0.991838	0.952668	0.767762	0.001168	1.51E-09	1.66E-08	3.10E-07	2.60E-07	5987	2232	3126	3385	3468
MPLKIP	0.413635	0.812404	0.624321	0.774367	0.723396	0.001198	7.00E-06	4.57E-05	7.34E-05	1.50E-09	5992	3937	5011	4786	2489
ANXA7	0.356584	0.338776	0.624472	0.512373	0.616194	0.001251	0.003662	2.96E-06	0.000159	4.15E-06	6001	5976	4276	4993	4062
PLAUR	-0.76886	-0.8133	-0.72709	-0.6672	-1.2998	0.00126	0.000403	0.004162	0.000585	9.78E-07	6003	5175	6493	5425	3730
IRF9	-0.4555	-0.75045	-0.41752	-0.63227	-0.61591	0.00127	6.59E-05	0.007545	6.20E-06	0.000328	6007	4565	6737	4073	5254
RNF13	-0.36045	-0.45899	-0.44559	-0.50811	-0.48903	0.001287	0.000278	3.91E-05	5.98E-05	9.11E-05	6009	5031	4963	4729	4897
TGDS	0.470063	0.658022	0.646608	0.56984	0.616922	0.001288	1.54E-05	1.67E-06	1.00E-05	4.96E-06	6010	4139	4150	4214	4103
ZNF3	0.305962	0.393009	0.387748	0.540549	0.475185	0.001302	0.000415	0.000249	7.90E-06	9.49E-05	6015	5187	5513	4140	4913
NDUFAF3	0.461444	0.537267	0.519042	0.570013	0.454987	0.001325	5.25E-05	5.60E-05	2.61E-05	0.000578	6024	4502	5062	4467	5446
RTL6	-0.47785	-0.43494	-0.42888	-0.33458	-0.3695	0.0014	0.000501	5.65E-05	0.0032	0.007114	6035	5253	5064	6074	6379
MMP7	0.742966	1.503843	1.574735	2.027489	0.973944	0.001416	0.00032	0.000108	2.59E-06	0.005925	6038	5086	5238	3862	6310
ASNS	0.634165	1.005299	0.447785	0.750954	0.769929	0.001428	3.50E-08	0.006972	1.31E-06	0.000199	6041	2773	6705	3702	5120
PANK2	-0.39187	-0.37421	-0.62533	-0.53843	-0.4214	0.00148	0.002261	1.31E-07	5.48E-05	6.77E-05	6045	5807	3572	4701	4809
CCNL2	-0.47528	-0.48889	-0.56234	-0.47913	-0.51242	0.001505	0.003214	0.000103	0.006248	3.52E-05	6051	5924	5222	6330	4617
AARS1	0.471433	0.415705	0.568429	0.561708	0.571516	0.001529	0.001164	0.000127	1.39E-05	0.000127	6055	5571	5283	4304	5000
SEC62	-0.36714	-0.53048	-0.55933	-0.5113	-0.44132	0.001539	5.36E-05	2.88E-07	0.000537	0.000101	6056	4505	3738	5409	4933
MFSD14B	0.375808	0.436813	0.727054	0.422034	0.608878	0.00156	0.002892	6.27E-08	0.005093	3.37E-06	6058	5882	3417	6247	4004
MAF1	0.511015	0.532883	0.58645	0.655251	0.587101	0.001563	0.000258	0.001321	1.03E-05	5.06E-05	6060	5002	6045	4220	4726
RNASEK	-0.53369	-0.93704	-0.65648	-0.83676	-0.70026	0.001566	6.09E-07	0.000586	1.10E-05	1.95E-05	6061	3355	5787	4240	4460
TTC22	0.66248	0.576203	1.062172	0.682388	0.680873	0.001595	0.002258	2.57E-07	1.86E-05	9.44E-06	6069	5805	3714	4381	4264
MRPL34	0.50915	0.494327	0.366246	0.588588	0.412391	0.001621	0.004573	0.006883	0.0009	0.004634	6074	6068	6698	5584	6190
PTGES	-0.5765	-0.58216	-0.52934	-0.61615	-0.92132	0.001628	0.000799	0.005226	0.001958	6.65E-06	6080	5418	6595	5874	4172
TIMM10	0.465862	0.961581	0.861482	0.992492	0.596699	0.001633	5.53E-05	4.66E-05	6.97E-06	0.001582	6081	4516	5018	4109	5811
NAPEPLD	0.472046	0.977663	0.651888	0.950244	0.572844	0.001655	9.48E-05	0.001844	0.000148	0.001259	6088	4683	6161	4972	5719
TMUB1	0.461	0.902845	0.58918	0.811986	0.840401	0.001656	6.84E-06	0.000155	2.43E-05	1.57E-08	6089	3930	5339	4442	2900
B3GNT5	0.708345	0.812771	0.797843	0.864562	0.927398	0.001656	0.000401	4.55E-06	7.52E-05	7.24E-06	6090	5173	4383	4791	4194
MICAL3	0.544673	0.630298	0.733258	0.704201	0.450718	0.001665	0.000225	3.63E-07	0.00022	0.003701	6093	4949	3800	5096	6096
TSR3	0.29467	0.467713	0.518233	0.435178	0.426195	0.001682	9.25E-07	1.36E-07	1.15E-05	1.95E-05	6098	3461	3582	4252	4459
SIRT5	0.359955	0.633831	0.442751	0.478356	0.689905	0.001819	1.68E-05	0.00016	1.47E-05	3.39E-06	6124	4159	5352	4319	4006
PAN3	0.48431	0.723099	0.483906	0.633151	0.598281	0.001822	1.86E-07	0.002519	1.74E-06	0.00025	6125	3134	6297	3763	5186
GMCL1	0.366678	0.479249	0.323443	0.417697	0.423818	0.001835	3.57E-05	0.004294	0.000282	1.74E-05	6128	4374	6513	5196	4433
BZW1	0.361169	0.383638	0.691237	0.469022	0.486395	0.001861	0.001655	1.64E-08	0.000286	5.44E-06	6132	5678	3122	5199	4130
USP53	0.553843	0.644462	1.05498	0.704295	0.456079	0.001866	0.000547	5.86E-07	1.21E-05	0.008391	6134	5295	3903	4263	6464
C1orf116	0.524605	0.72501	0.540644	0.440471	0.72514	0.001899	0.000288	0.000843	0.005213	2.21E-05	6140	5043	5914	6258	4490
ARHGEF18	0.429437	0.631354	0.506426	0.634882	0.391555	0.002074	2.77E-05	0.000355	6.00E-07	0.009248	6159	4311	5625	3538	6523
GSTA4	-0.45769	-0.61107	-0.7813	-0.73082	-0.98351	0.002079	6.64E-05	1.03E-06	1.86E-05	3.52E-06	6160	4568	4043	4380	4024
STX3	0.714369	1.012882	1.09472	0.930577	0.955861	0.002079	7.56E-05	3.01E-06	0.000141	8.82E-07	6161	4609	4283	4961	3708
PPFIBP1	-0.4937	-0.64826	-0.56227	-0.55424	-1.13745	0.00209	0.000499	0.000374	0.004015	5.09E-08	6162	5251	5646	6162	3125
FAM117B	0.290183	0.474754	0.561072	0.53484	0.660105	0.002104	3.40E-05	8.33E-06	4.48E-07	5.07E-06	6166	4364	4542	3474	4111
DENND4B	-0.27364	-0.24686	-0.52636	-0.60827	-0.55322	0.002126	0.008306	4.41E-05	1.44E-06	0.000138	6169	6350	5001	3720	5023
CFAP36	-0.36612	-0.39874	-0.3166	-0.40742	-0.43711	0.00218	0.001863	0.005977	0.001085	0.000374	6176	5733	6656	5656	5299
RO60	-0.38746	-0.41091	-0.39822	-0.75709	-0.64549	0.002197	0.001494	0.003104	4.26E-08	0.000613	6180	5654	6366	3014	5471
RREB1	0.352747	0.37913	0.554766	0.425683	0.569927	0.002213	0.002355	9.36E-07	0.000617	7.14E-08	6181	5822	4019	5449	3206
MTA2	0.419331	0.721843	0.581283	0.829322	0.395553	0.002277	1.80E-06	3.16E-06	1.31E-08	0.002256	6185	3628	4293	2811	5950
RESF1	-0.48	-0.52196	-0.60847	-0.58712	-0.88417	0.002323	0.002535	8.05E-05	0.001521	1.39E-06	6197	5846	5162	5778	3808
SLC6A8	0.880169	0.962607	1.533125	1.02049	1.026108	0.002353	0.000753	1.20E-05	3.29E-05	0.001501	6200	5396	4641	4549	5793
FADS2	-1.28741	-1.37151	-1.11785	-1.18458	-1.36697	0.002404	4.84E-05	0.001195	0.000963	7.44E-06	6204	4475	6014	5615	4207
CDC5L	0.366578	0.352645	0.470587	0.316487	0.347392	0.002407	0.00195	5.98E-05	0.005497	0.000334	6207	5751	5080	6285	5261
NIPSNAP2	0.452821	0.719917	0.787862	0.719533	1.148105	0.002417	0.000194	1.80E-06	0.00012	6.75E-10	6208	4905	4171	4912	2359
SARS1	0.357904	0.445521	0.36146	0.380328	0.477152	0.002437	0.000251	0.000933	0.000103	6.64E-05	6210	4992	5936	4865	4803
SEH1L	0.288713	0.316973	0.482758	0.396496	0.432517	0.002445	0.000214	6.33E-06	8.99E-06	5.25E-06	6211	4929	4462	4175	4122
ENAH	-0.52131	-0.4122	-0.37604	-0.47866	-0.43219	0.002491	0.004925	0.001157	0.002619	0.003327	6216	6089	6001	5992	6063
FAAH	0.447372	0.790038	0.637263	0.56688	0.612179	0.00254	6.44E-05	0.000109	0.002985	0.000743	6227	4553	5247	6049	5543
SLC25A38	0.485462	0.929497	0.867399	0.89123	0.608517	0.002603	2.18E-05	5.02E-05	4.29E-05	0.001034	6232	4239	5039	4628	5644

ARCN1	-0.36485	-0.61462	-0.51154	-0.61986	-0.42835	0.002603	5.08E-05	0.000177	7.79E-05	0.001056	6233	4488	5399	4798	5658
RMND5A	0.418136	0.646469	0.696689	0.677915	0.579607	0.002616	1.61E-06	2.47E-07	6.96E-06	1.43E-06	6236	3597	3702	4108	3810
ATR	0.425133	0.840143	0.393448	0.715486	0.56148	0.002618	3.82E-08	0.00303	1.36E-07	0.000906	6240	2788	6358	3222	5601
USP1	0.368138	0.575171	0.679662	0.480394	0.457992	0.002629	5.87E-07	6.95E-10	0.000134	0.00059	6242	3348	2522	4948	5457
CASP10	0.334464	0.523492	0.613022	0.536402	0.39018	0.002637	0.00013	6.46E-05	0.000205	0.005818	6245	4767	5102	5068	6302
RORC	-0.37428	-0.35262	-0.47	-0.45204	-0.34691	0.002808	0.001803	0.000355	0.001685	0.007065	6262	5714	5626	5822	6376
PTMS	-0.50544	-0.94621	-1.1088	-1.24344	-0.85607	0.002871	0.000308	2.24E-05	9.69E-05	0.000589	6270	5070	4808	4849	5456
ALKBH7	0.317711	0.664162	0.485216	0.646478	0.510218	0.00288	1.49E-06	1.71E-05	4.63E-07	4.97E-06	6272	3583	4741	3481	4104
FAM110C	0.433899	0.714735	0.83675	0.852167	1.124463	0.002911	1.25E-06	5.68E-07	5.13E-06	8.21E-10	6276	3539	3892	4030	2397
KIAA0100	0.390994	0.366089	0.494588	0.61937	0.45047	0.002942	0.00945	0.000175	2.70E-05	6.37E-05	6277	6403	5394	4480	4788
LYPD6B	-0.47595	-0.39001	-0.4582	-0.44955	-0.71205	0.002984	0.007815	0.006313	0.005356	5.52E-07	6287	6316	6666	6271	3619
FUCA1	-0.79963	-0.82191	-0.76745	-1.11844	-0.69031	0.003185	0.000668	0.00128	7.90E-06	0.00401	6307	5365	6036	4139	6126
PCGF5	0.347674	0.458345	0.483772	0.500936	0.56825	0.003185	0.001159	2.19E-05	0.00024	6.14E-05	6308	5567	4801	5128	4778
RRP9	0.552317	0.798146	0.9146	0.647986	0.861042	0.003199	9.98E-05	7.71E-07	0.000987	9.72E-05	6312	4695	3966	5619	4921
IQCE	0.461752	0.476089	0.717746	0.54401	0.552314	0.003257	0.008071	2.94E-06	0.00197	0.000225	6313	6333	4275	5878	5152
MRPS28	0.395507	0.574692	0.754	0.549529	0.426333	0.003269	0.000148	2.21E-06	0.001281	0.005213	6316	4809	4200	5715	6251
XRN2	0.416864	0.603079	0.483326	0.59862	0.662356	0.003315	0.001026	0.002284	0.00054	8.11E-07	6322	5515	6257	5412	3693
NOL3	0.569723	0.829622	0.607964	1.187789	0.597344	0.00332	0.000172	0.00447	1.70E-07	0.000555	6323	4866	6526	3268	5434
TMEM129	0.371439	0.536626	0.630019	0.550925	0.66819	0.003517	0.001292	6.61E-06	0.000627	1.55E-07	6340	5612	4473	5455	3359
MFSD6	0.563355	0.932339	0.828277	0.986067	0.956866	0.003585	1.69E-07	2.47E-07	1.37E-07	2.69E-07	6347	3112	3703	3224	3478
MALL	1.084157	0.879623	1.832051	0.840377	1.539952	0.003733	0.007653	7.20E-07	0.009361	2.06E-06	6359	6306	3952	6501	3881
MAN2A1	-0.28552	-0.474	-0.5016	-0.55666	-0.66568	0.003748	0.00775	0.000255	0.002115	1.41E-06	6361	6312	5523	5905	3809
FZR1	0.339514	0.686594	0.625487	0.636736	0.392252	0.003756	4.11E-06	1.04E-05	1.57E-05	0.000982	6362	3823	4604	4338	5624
TRNT1	0.390362	0.473557	0.440207	0.389953	0.452779	0.003849	0.000753	0.001116	0.002567	0.001847	6369	5397	5987	5975	5878
YME1L1	0.276765	0.318238	0.486966	0.422389	0.713709	0.003898	0.003694	1.15E-05	0.000564	8.83E-07	6377	5981	4627	5418	3709
TIMM23B	0.375059	0.507532	0.462683	0.437436	0.775686	0.003989	0.000658	0.001223	0.001587	9.13E-07	6388	5359	6023	5798	3716
MUC4	0.876773	1.020687	1.55331	1.247669	1.267151	0.004027	0.000214	3.46E-07	1.07E-06	3.08E-06	6392	4931	3789	3657	3987
CAAP1	0.422994	0.686577	0.688502	0.723493	0.590564	0.004114	0.000233	2.08E-05	7.64E-06	0.000105	6402	4964	4786	4131	4950
USP6NL	0.4435	0.819208	0.652535	0.795827	0.736689	0.004117	6.72E-05	0.000169	0.000114	3.53E-06	6403	4575	5374	4890	4025
MTX2	0.421678	0.646042	0.463073	0.659295	0.619301	0.004198	0.000198	0.000105	0.000411	1.55E-05	6407	4911	5228	5317	4406
RNF103	0.478016	0.555184	0.579654	0.592101	0.750044	0.004222	0.002573	0.00015	0.000374	1.35E-06	6410	5851	5333	5289	3802
GTPBP4	0.451279	0.438581	0.704512	0.402865	0.550068	0.004257	0.004983	0.000173	0.006237	0.000174	6414	6094	5385	6329	5079
XPO4	0.368745	0.476905	0.296996	0.419322	0.559113	0.004275	0.000707	0.004191	0.0003	4.45E-05	6417	5381	6497	5220	4687
GPN1	0.360216	0.517662	0.338105	0.626135	0.420874	0.004326	0.000528	0.009694	1.79E-06	0.001927	6420	5273	6835	3771	5894
FOXQ1	0.65123	0.819839	1.104706	1.109238	1.311465	0.004371	0.002269	2.76E-07	6.51E-05	4.07E-07	6422	5809	3729	4753	3565
CLDN2	0.756368	0.895354	1.556287	0.936045	1.153723	0.00445	0.004768	1.43E-07	0.002082	1.19E-05	6427	6082	3596	5897	4328
C8orf33	0.482666	0.69488	0.743569	0.638042	0.633729	0.004483	1.15E-05	2.23E-06	4.61E-05	1.65E-06	6428	4068	4204	4650	3846
RBM26	0.468783	0.565757	0.475278	0.498814	0.956335	0.00449	0.000221	0.007846	0.001012	4.11E-06	6430	4946	6748	5630	4054
COPG1	0.354657	0.36702	0.54607	0.426122	0.48945	0.004527	0.007188	4.63E-05	0.001558	1.40E-05	6432	6268	5015	5790	4379
GOLM1	0.871554	0.948328	1.748117	0.747838	1.284139	0.004551	0.00016	2.32E-09	0.00466	1.90E-07	6438	4835	2733	6219	3407
SOCS6	-0.29799	-0.34662	-0.31196	-0.3888	-0.29175	0.004667	0.002105	0.005393	0.001303	0.004974	6444	5789	6608	5724	6222
TRAPPC6A	0.43987	0.713157	0.65645	0.592153	0.525679	0.004717	2.77E-05	7.72E-05	0.000185	0.000922	6447	4310	5144	5039	5605
NECTIN4	0.583311	0.85024	0.743457	0.858357	0.819962	0.004833	0.000137	2.08E-06	5.51E-05	4.12E-06	6453	4786	4189	4706	4057
NSL1	-0.27975	-0.34876	-0.38892	-0.33233	-0.32006	0.004905	0.000528	7.93E-06	0.001107	0.002667	6458	5276	4527	5665	5999
NUDT14	0.371344	1.063239	0.773821	0.803631	1.017074	0.004929	1.33E-07	3.02E-05	3.89E-05	4.54E-12	6460	3058	4896	4601	1659
AMOT	0.581407	0.47578	0.369759	0.410618	0.585251	0.005044	0.001406	0.007261	0.00271	0.000343	6464	5634	6720	6004	5271
PCMTD2	0.488042	0.707173	0.514332	0.834762	0.372945	0.005105	1.30E-06	0.000632	8.10E-07	0.00706	6466	3550	5801	3594	6375
SMPD4	0.314474	0.508249	0.378758	0.39982	0.267358	0.005108	1.82E-06	0.000263	0.00085	0.007688	6467	3634	5533	5559	6420
ZNF800	0.353751	0.388162	0.520657	0.449504	0.377724	0.005206	0.00064	6.42E-05	0.000649	0.001467	6472	5343	5098	5468	5778
HNF1B	0.589393	0.943723	1.193524	1.022953	0.741533	0.00522	0.001191	1.65E-06	5.98E-05	0.000504	6476	5583	4145	4728	5409
TBCC	-0.28988	-0.26808	-0.463	-0.46223	-0.41963	0.005224	0.006454	2.08E-06	9.74E-06	2.71E-05	6477	6215	4190	4204	4536
OSBP	0.251997	0.378727	0.403673	0.482063	0.381189	0.005431	0.000245	0.000185	2.36E-06	0.000105	6493	4982	5413	3838	4953
ARHGEF2	-0.50172	-0.69681	-0.78146	-0.68901	-0.51581	0.005505	3.65E-05	8.83E-06	0.000465	0.001501	6496	4386	4557	5348	5794
TRAF5	0.359061	0.702039	0.867781	0.822188	1.081976	0.005547	1.50E-06	8.36E-06	4.44E-06	9.11E-08	6501	3584	4543	3990	3261
HSPB6	-0.47911	-0.4462	-0.8496	-0.65392	-0.83072	0.005547	0.005431	3.13E-09	0.000198	1.28E-11	6502	6139	2796	5055	1799
SAA2	0.514365	0.854008	0.875308	0.866759	0.529235	0.005625	8.25E-05	0.001224	0.000498	0.007773	6504	4640	6024	5374	6430
SETX	-0.40518	-0.59294	-0.60377	-0.60359	-0.41497	0.005789	0.001607	0.00042	0.003331	0.006943	6515	5671	5682	6097	6371
CCNG1	0.476992	0.584292	0.588168	0.443475	0.723286	0.005949	6.28E-05	1.78E-05	0.001251	8.70E-08	6527	4547	4752	5710	3248
LRP8	0.597136	1.428061	1.071222	1.362876	0.788654	0.00641	5.44E-05	0.000206	2.38E-05	0.000392	6555	4512	5450	4431	5314
ALKBH5	-0.38259	-0.55039	-0.38607	-0.52639	-0.3819	0.006434	1.06E-05	0.003905	8.60E-05	0.009472	6556	4044	6469	4818	6540
GIT1	0.435522	0.505912	0.483001	0.639226	0.425261	0.006485	0.002877	0.001376	0.000424	0.000253	6559	5881	6065	5328	5188
NIP7	0.43495	0.653764	0.529219	0.535803	0.999768	0.006637	0.000178	0.001399	0.001666	7.11E-08	6568	4872	6072	5815	3204
MAP2K6	0.556052	1.122796	0.941956	1.442311	1.174972	0.006778	6.26E-07	8.83E-06	1.91E-09	7.18E-06	6583	3361	4558	2509	4190
IRF7	-0.6789	-0.78208	-0.88234	-0.7821	-1.12417	0.006967	0.005769	3.69E-07	0.002437	9.32E-09	6591	6166	3801	5963	2796
SYNE4	0.381092	0.936124	0.682903	0.834144	0.689372	0.007114	1.57E-07	6.16E-05	2.86E-06	6.12E-06	6596	3094	5088	3891	4154
TP53INP2	-0.50748	-0.46907	-0.75991	-0.524	-0.73192	0.007124	0.004559	7.20E-06	0.002731	1.01E-06	6598	6066	4498	6006	3740
TOR4A	0.456011	0.863806	0.922247	0.634996	0.701538	0.007197	5.38E-05	7.83E-07	0.002321	2.35E-05	6603	4508	3973	5943	4506
LCE2D	0.629173	0.786733	0.775258	1.089809	1.241142	0.007204	0.001999	6.59E-05	7.10E-05	2.17E-07	6604	5769	5109	4771	3432
GPALPP1	0.464271	0.697628	0.574825	0.501323	0.87948	0.007222	3.80E-05	0.00039	0.000288	1.45E-05	6607	4399	5662	5204	4386
TRIM27	0.332342	0.40737	0.514	0.60943	0.506426	0.007428	0.006248	0.000446	1.61E-06	0.000281	6619	6198	5703	3749	5219
ZMYM2	0.390477	0.432593	0.615888	0.453977	1.034927	0.00781	0.007519	0.000175	0.00359	2.53E-09	6637	6293	5393	6112	2585
RBM25	0.324015	0.657704	0.614673	0.57878	0.68635	0.007826	2.02E-07	2.90E-05	2.05E-05	6.83E-07	6640	3151	4876	4403	3662
STYXL1	0.416583	0.619552	0.478614	0.50946	0.484981	0.007831	0.000372	0.0019	0.004095	0.000635	6641	5145	6177	6168	5483

ZNFX1	0.322948	0.344766	0.511926	0.587922	0.396508	0.007831	0.007897	0.002639	0.000189	0.004569	6642	6323	6320	5046	6184
LCOR	0.353808	0.406972	0.895861	0.487583	0.800677	0.007879	0.006336	6.15E-08	0.002051	9.82E-07	6644	6205	3412	5890	3732
SHROOM1	0.38455	0.75988	0.497012	0.63557	0.402258	0.007968	4.66E-06	0.000729	0.000103	0.006053	6648	3850	5860	4864	6313
RIF1	0.283549	0.808096	0.37477	0.722103	0.621476	0.008222	4.21E-07	0.002241	5.23E-06	1.96E-06	6662	3278	6251	4033	3874
PRKD2	-0.38137	-0.507	-0.95718	-0.96812	-0.81511	0.008294	0.002081	0.000758	0.000258	0.000623	6666	5782	5872	5154	5475
SULT2B1	0.495879	0.819294	1.063285	0.707906	1.029969	0.008316	8.81E-05	2.12E-11	0.000757	6.64E-08	6667	4664	1989	5527	3180
WDR59	0.341422	0.502676	0.40278	0.647038	0.363395	0.008374	0.000804	0.002266	3.48E-07	0.001045	6669	5421	6256	3413	5649
POLR1H	0.343911	0.494104	0.428106	0.305937	0.463981	0.008428	0.00024	0.000146	0.009436	7.05E-05	6672	4971	5323	6507	4826
RAP1B	-0.41286	-0.77009	-0.43795	-0.72611	-0.55322	0.008437	2.01E-05	0.007978	0.000103	0.000181	6674	4214	6758	4866	5100
POLD1	0.342794	0.699786	0.360129	0.599827	0.424038	0.008503	2.25E-07	0.00712	2.61E-05	0.000501	6677	3173	6711	4464	5407
MTIF2	0.395321	0.392135	0.640502	0.448502	0.522482	0.008597	0.004769	0.000117	0.004341	0.001356	6680	6083	5265	6195	5746
TES	0.314983	0.712866	0.782054	0.768557	0.520921	0.008698	2.75E-05	3.22E-07	5.93E-06	0.00049	6685	4309	3774	4060	5400
NOS2	0.669539	1.066727	1.712253	1.333684	1.050062	0.008774	2.69E-05	1.24E-07	2.39E-06	0.000594	6692	4302	3564	3843	5459
TNFRSF11	-0.73475	-0.82961	-1.41677	-1.14998	-1.37349	0.008936	0.007382	0.000157	0.004205	0.000234	6706	6282	5345	6179	5166
ADAM10	-0.30884	-0.60137	-0.49995	-0.52555	-0.42113	0.009142	0.000501	0.001628	0.000403	0.009138	6712	5254	6110	5312	6514
CALML4	0.308972	0.608927	0.538318	0.465038	0.463921	0.009271	0.000393	0.00023	0.004781	6.22E-05	6723	5163	5488	6226	4783
MIEN1	0.465015	0.993934	1.192891	0.789728	0.98321	0.009323	2.90E-05	4.08E-06	1.96E-07	9.83E-05	6726	4322	4349	3299	4923
GALNT12	0.598626	0.626365	0.891957	0.665108	0.491607	0.009398	0.001642	4.77E-06	0.001686	0.005924	6728	5676	4398	5825	6309
LEFTY1	0.871107	1.860319	1.505727	1.89911	2.609297	0.009549	7.67E-07	1.18E-05	3.29E-07	1.55E-08	6735	3410	4636	3399	2897
NAXD	0.335397	0.417763	0.314983	0.462746	0.594009	0.009661	0.003241	0.004678	0.000925	4.11E-06	6743	5927	6546	5593	4055

ID	Description	pvalue	p.adjust	setSize	Count	GeneRatio	.sign	core_enrichment
R-HSA-726	Formation (	1.00E-10	1.24E-09	96	83	0.864583	activated	6227/6132/6173/6222/6170/[illegible]
R-HSA-156	Eukaryotic	1.00E-10	1.24E-09	85	77	0.905882	activated	6227/6132/6173/6222/6170/[illegible]
R-HSA-156	Peptide cha	1.00E-10	1.24E-09	82	74	0.902439	activated	6227/6132/6173/6222/6170/[illegible]
R-HSA-192	Viral mRNA	1.00E-10	1.24E-09	84	74	0.880952	activated	6227/6132/6173/6222/6170/[illegible]
R-HSA-727	Eukaryotic	1.00E-10	1.24E-09	86	75	0.872093	activated	6227/6132/6173/6222/6170/[illegible]
R-HSA-156	L13a-media	1.00E-10	1.24E-09	106	89	0.839623	activated	6227/6132/6173/26986/[illegible]
R-HSA-240	Selenocyste	1.00E-10	1.24E-09	86	74	0.860465	activated	6227/6132/6173/6222/6170/[illegible]
R-HSA-727	GTP hydrol	1.00E-10	1.24E-09	107	88	0.82243	activated	6227/6132/6173/6222/6170/[illegible]
R-HSA-726	Eukaryotic	1.00E-10	1.24E-09	114	89	0.780702	activated	6227/6132/6173/26986/[illegible]
R-HSA-727	Cap-depen	1.00E-10	1.24E-09	114	89	0.780702	activated	6227/6132/6173/26986/[illegible]
R-HSA-179	SRP-depen	1.00E-10	1.24E-09	106	80	0.754717	activated	6227/6132/6173/6222/6170/[illegible]
R-HSA-975	Nonsense M	1.00E-10	1.24E-09	89	76	0.853933	activated	6227/6132/6173/26986/[illegible]
R-HSA-963	Response c	1.00E-10	1.24E-09	95	76	0.8	activated	6227/6132/6173/6222/6170/[illegible]
R-HSA-927	Nonsense-	1.00E-10	1.24E-09	109	83	0.761468	activated	6227/6132/6173/26986/[illegible]
R-HSA-975	Nonsense M	1.00E-10	1.24E-09	109	83	0.761468	activated	6227/6132/6173/26986/[illegible]
R-HSA-168	Influenza V	1.00E-10	1.24E-09	129	80	0.620155	activated	6227/6132/6173/6222/6170/[illegible]
R-HSA-240	Selenoamin	1.00E-10	1.24E-09	106	78	0.735849	activated	6227/191/6132/6173/6222/[illegible]
R-HSA-971	Cellular res	1.00E-10	1.24E-09	131	76	0.580153	activated	6227/6132/6173/6222/6170/[illegible]
R-HSA-901	Regulation	1.00E-10	1.24E-09	152	111	0.730263	activated	6227/6132/6173/26986/[illegible]
R-HSA-168	Influenza In	1.00E-10	1.24E-09	147	97	0.659864	activated	6227/6132/6173/3304/6222/[illegible]
R-HSA-975	SARS-CoV-2	1.00E-10	1.24E-09	46	39	0.847826	activated	6227/6222/6187/6188/6636/[illegible]
R-HSA-886	rRNA proce	1.00E-10	1.24E-09	179	91	0.50838	activated	6227/6132/6173/6222/6170/[illegible]
R-HSA-679	Major path	1.00E-10	1.24E-09	169	83	0.491124	activated	6227/6132/6173/6222/6170/[illegible]
R-HSA-727	Translation	1.00E-10	1.24E-09	271	199	0.734317	activated	6227/6132/6173/26986/[illegible]
R-HSA-726	Formation (	1.00E-10	1.24E-09	51	42	0.823529	activated	6227/6222/6187/6188/6194/[illegible]
R-HSA-973	SARS-CoV-1	1.00E-10	1.24E-09	35	32	0.914286	activated	6227/6222/6187/6188/6194/[illegible]
R-HSA-723	rRNA proce	1.00E-10	1.24E-09	187	92	0.491979	activated	6227/6132/6173/6222/6170/[illegible]
R-HSA-726	Translation	1.00E-10	1.24E-09	58	46	0.793103	activated	6227/26986/6222/6187/[illegible]
R-HSA-726	Activation c	1.00E-10	1.24E-09	59	46	0.779661	activated	6227/26986/6222/6187/[illegible]
R-HSA-727	Ribosomal	1.00E-10	1.24E-09	58	45	0.775862	activated	6227/6222/6187/6188/6194/[illegible]
R-HSA-147	Extracellula	1.00E-10	1.24E-09	194	113	0.582474	suppressed	1300/101/5352/10536/650/[illegible]
R-HSA-712	Metabolism	1.00E-10	1.24E-09	261	155	0.59387	activated	6227/191/6132/6173/1728/[illegible]
R-HSA-693	DNA Replic	1.00E-10	1.24E-09	159	120	0.754717	activated	11065/8339/333932/3017/[illegible]
R-HSA-688	Assembly o	1.00E-10	1.24E-09	125	95	0.76	activated	11065/8339/333932/3017/[illegible]
R-HSA-147	Degradation	1.00E-10	1.24E-09	88	49	0.556818	suppressed	1509/1307/2006/9507/1301/[illegible]
R-HSA-690	DNA Replic	1.00E-10	1.24E-09	138	106	0.768116	activated	11065/8339/333932/3017/[illegible]
R-HSA-300	ECM protec	1.00E-10	1.24E-09	46	31	0.673913	suppressed	2331/3685/5802/3690/3913/[illegible]
R-HSA-142	The citric ac	1.00E-10	1.24E-09	150	92	0.613333	activated	10131/1337/1329/1345/4191/[illegible]
R-HSA-144	Collagen de	1.00E-10	1.24E-09	43	31	0.72093	suppressed	4327/1509/1307/1301/50509/[illegible]
R-HSA-163	Respiratory	1.00E-10	1.24E-09	107	72	0.672897	activated	10131/1337/1329/1345/1327/[illegible]
R-HSA-376	Signaling by	1.00E-10	1.24E-09	186	115	0.61828	activated	6227/6132/6173/26986/[illegible]
R-HSA-737	RNA Polym	1.00E-10	1.24E-09	72	46	0.638889	activated	8339/333932/3017/554313/[illegible]
R-HSA-964	Diseases of	1.00E-10	1.24E-09	86	50	0.581395	activated	8339/333932/3017/554313/[illegible]
R-HSA-737	RNA Polym	1.00E-10	1.24E-09	51	34	0.666667	activated	8339/333932/3017/554313/[illegible]
R-HSA-229	Condensati	1.00E-10	1.24E-09	62	48	0.774194	activated	8339/333932/3017/554313/[illegible]
R-HSA-427	NoRC negat	1.00E-10	1.24E-09	86	48	0.55814	activated	8339/333932/3017/554313/[illegible]
R-HSA-427	SIRT1 negat	1.00E-10	1.24E-09	52	39	0.75	activated	8339/333932/3017/554313/[illegible]
R-HSA-525	Negative ep	1.00E-10	1.24E-09	88	47	0.534091	activated	8339/333932/3017/554313/[illegible]
R-HSA-533	DNA methy	1.00E-10	1.24E-09	52	39	0.75	activated	8339/333932/3017/554313/[illegible]
R-HSA-321	RMTs meth	1.00E-10	1.24E-09	67	49	0.731343	activated	333932/8336/6187/554313/[illegible]
R-HSA-971	Defective p	1.00E-10	1.24E-09	60	41	0.683333	activated	8339/333932/3017/554313/[illegible]
R-HSA-912	Meiotic rec	1.00E-10	1.24E-09	67	42	0.626866	activated	8339/333932/3017/554313/[illegible]
R-HSA-216	Integrin cell	1.00E-10	1.24E-09	60	39	0.65	suppressed	3695/3683/3384/7412/[illegible]
R-HSA-686	Assembly o	1.00E-10	1.24E-09	55	35	0.636364	activated	8339/333932/3017/554313/[illegible]
R-HSA-982	Chromatin	1.00E-10	1.24E-09	55	39	0.709091	activated	8339/333932/3017/554313/[illegible]
R-HSA-212	PRC2 meth	1.00E-10	1.24E-09	61	36	0.590164	activated	8339/333932/3017/554313/[illegible]
R-HSA-321	HDACs dea	1.00E-10	1.24E-09	80	47	0.5875	activated	8339/333932/8336/3017/[illegible]
R-HSA-692	Synthesis o	1.00E-10	1.24E-09	107	77	0.719626	activated	11065/5688/4173/6233/[illegible]
R-HSA-562	Activated P	1.00E-10	1.24E-09	52	34	0.653846	activated	8339/333932/3017/554313/[illegible]
R-HSA-525	B-WICH cor	1.00E-10	1.24E-09	73	56	0.767123	activated	8339/333932/3017/554313/[illegible]
R-HSA-427	ERCC6 (CSB	1.00E-10	1.24E-09	63	43	0.68254	activated	8339/333932/3017/554313/[illegible]
R-HSA-611	Respiratory	1.00E-10	1.24E-09	89	59	0.662921	activated	10131/1337/1329/1345/1327/[illegible]
R-HSA-557	Transcriptic	1.00E-10	1.24E-09	93	54	0.580645	activated	8339/333932/3017/554313/[illegible]
R-HSA-692	S Phase	1.00E-10	1.24E-09	140	94	0.671429	activated	4609/11065/5688/4173/[illegible]
R-HSA-165	Collagen bi	1.00E-10	1.24E-09	41	28	0.682927	suppressed	1300/5352/10536/1307/[illegible]
R-HSA-688	Mitotic Pro	1.00E-10	1.24E-09	127	66	0.519685	activated	8339/333932/3017/554313/[illegible]
R-HSA-961	HCMV Late	1.00E-10	1.24E-09	103	57	0.553398	activated	8339/333932/8336/3017/[illegible]
R-HSA-147	Collagen fo	1.00E-10	1.24E-09	59	36	0.610169	suppressed	1300/5352/10536/4015/[illegible]
R-HSA-525	Positive epi	1.00E-10	1.24E-09	88	51	0.579545	activated	8339/333932/3017/554313/[illegible]
R-HSA-960	HCMV Early	1.00E-10	1.24E-09	111	55	0.495495	activated	8339/333932/10383/8336/[illegible]
R-HSA-981	Maternal to	1.00E-10	1.24E-09	91	53	0.582418	activated	8339/26986/333932/3017/[illegible]
R-HSA-738	RNA Polym	1.00E-10	1.24E-09	90	49	0.544444	activated	8339/333932/3017/554313/[illegible]
R-HSA-201	Formation (	1.00E-10	1.24E-09	76	46	0.605263	activated	4609/8313/8339/333932/[illegible]
R-HSA-202	Assembly o	1.00E-10	1.24E-09	44	28	0.636364	suppressed	4015/4016/1301/50509/[illegible]
R-HSA-381	Regulation	1.00E-10	1.24E-09	67	32	0.477612	suppressed	813/54757/54972/11098/[illegible]
R-HSA-690	Switching o	1.00E-10	1.24E-09	82	59	0.719512	activated	11065/5688/4173/6233/[illegible]
R-HSA-692	G1/S Transi	1.00E-10	1.24E-09	113	77	0.681416	activated	4609/6241/5688/4173/6233/[illegible]
R-HSA-150	Meiosis	1.00E-10	1.24E-09	88	47	0.534091	activated	8339/333932/3017/554313/[illegible]
R-HSA-211	Gene Silenc	1.00E-10	1.24E-09	108	57	0.527778	activated	8339/333932/3017/554313/[illegible]
R-HSA-156	Elastic fibre	1.00E-10	1.24E-09	31	28	0.903226	suppressed	5045/3688/3693/2201/650/[illegible]
R-HSA-969	SARS-CoV-1	1.00E-10	1.24E-09	85	40	0.470588	activated	6227/2810/6222/6187/6188/[illegible]
R-HSA-321	HATs acetyl	1.00E-10	1.24E-09	117	45	0.384615	activated	8339/333932/8336/3017/[illegible]
R-HSA-157	Telomere M	1.00E-10	1.24E-09	97	46	0.474227	activated	8339/3017/554313/3012/[illegible]
R-HSA-894	Collagen ch	1.00E-10	1.24E-09	25	19	0.76	suppressed	1307/1301/50509/7373/[illegible]
R-HSA-453	Mitotic G1	1.00E-10	1.24E-09	126	81	0.642857	activated	4609/4605/6241/5688/4173/[illegible]
R-HSA-694	G2/M Chec	1.00E-10	1.24E-09	141	93	0.659574	activated	8339/2810/3017/554313/[illegible]
R-HSA-895	Post-transl	1.00E-10	1.24E-09	63	29	0.460317	suppressed	813/54757/54972/11098/[illegible]
R-HSA-893	RUNX1 regu	1.00E-10	1.24E-09	109	69	0.633028	activated	8339/333932/3017/554313/[illegible]
R-HSA-901	Estrogen-de	1.00E-10	1.24E-09	125	59	0.472	activated	7033/4609/8339/333932/[illegible]
R-HSA-692	Cell Cycle, M	1.00E-10	1.24E-09	450	241	0.535556	activated	4609/11065/8339/333932/[illegible]
R-HSA-738	Chromosor	1.00E-10	1.24E-09	116	52	0.448276	activated	8339/3017/554313/3012/[illegible]
R-HSA-738	RNA Polym	1.00E-10	1.24E-09	91	49	0.538462	activated	8339/333932/3017/554313/[illegible]
R-HSA-960	HCMV Infec	1.00E-10	1.24E-09	135	59	0.437037	activated	8339/333932/10383/8336/[illegible]
R-HSA-212	Molecules a	1.00E-10	1.24E-09	26	23	0.884615	suppressed	3688/3693/2201/650/8425/[illegible]
R-HSA-688	M Phase	1.00E-10	1.24E-09	339	163	0.480826	activated	11065/8339/333932/10383/[illegible]
R-HSA-212	Epigenetic r	1.00E-10	1.24E-09	157	73	0.464968	activated	8339/333932/3017/554313/[illegible]
R-HSA-721	mRNA Splic	1.00E-10	1.24E-09	195	103	0.528205	activated	6636/25804/51690/6633/[illegible]
R-HSA-721	mRNA Splic	1.00E-10	1.24E-09	203	104	0.512315	activated	6636/25804/51690/6633/[illegible]
R-HSA-696	Cell Cycle C	1.00E-10	1.24E-09	239	132	0.552301	activated	11065/8339/2810/3017/[illegible]
R-HSA-722	Processing	1.00E-10	1.24E-09	267	163	0.610487	activated	6636/25804/51690/6633/[illegible]
